# Social cognition in Parkinson’s disease: a comprehensive systematic review and integrative conceptual framework

**DOI:** 10.3389/fnagi.2026.1863728

**Published:** 2026-06-29

**Authors:** Maor Yeshua, Elise Klein, Martin Südmeyer, Liane Kaufmann

**Affiliations:** 1 Department of Neurology and Clinical Neuropsychology, Klinikum Ernst von Bergmann, Potsdam, Germany; 2 Department of Psychology, Ben-Gurion University of the Negev, Be’er Sheva, Israel; 3 LaPsyDÉ, UMR CNRS 8240, Université Paris Cité, Paris, France

**Keywords:** emotion recognition, neuroimaging, Parkinson’s disease, social cognition, social problem-solving, Theory of Mind (ToM)

## Abstract

**Introduction:**

Parkinson’s disease (PD) is characterized not only by motor symptoms but also by non-motor impairments, including deficits in social cognition (SC), defined as the ability to recognize, interpret, and respond to others’ emotions, intentions, and behaviors. This systematic review synthesizes evidence on SC in PD, focusing on three core aspects: emotion recognition, empathy/Theory of Mind (ToM), and social problem-solving.

**Methods:**

We included 191 behavioral and neuroimaging studies based on a PRISMA-guided search of four databases.

**Results:**

The literature review findings revealed relatively consistent impairments in the recognition of negative emotions, such as fear and anger, and heterogeneous findings for empathy/ToM, with deficits being more pronounced in advanced PD or in patients with mild cognitive impairment (MCI). Social problem-solving remains understudied, though emerging evidence links it to dopaminergic modulation.

**Discussion:**

Across all three aspects, methodological heterogeneity and the scarcity of neuroimaging studies addressing complex SC aspects underscore the need for standardized, multimodal research. Beyond synthesizing the literature, we propose an integrative neurofunctional model of SC in PD that emphasizes its componential nature and highlights the need for future studies to clarify interrelations among SC domains and their neural substrates.

## 1 General introduction

Parkinson’s disease (PD) is the second most common neurodegenerative disease (Poewe et al., 2017) and is known to affect both motor and non-motor functioning (Bloem et al., 2021; Sprenger and Poewe, 2013). Although motor symptoms such as tremor, rigidity, bradykinesia, and postural instability are considered the hallmark symptoms of PD, patients also experience a range of non-motor symptoms affecting, among other domains, cognition and mood [e.g., (Armstrong and Okun, 2020; Pfeiffer, 2016)]. Such non-motor symptoms are reported to substantially reduce patients’ quality of life as well as their responsiveness to treatment (Aarsland et al., 2021; Mole and Prangnell, 2019). Regarding non-motor cognitive functions, patients with PD, particularly those who develop mild cognitive impairment (MCI) in the course of their disease, frequently exhibit executive dysfunctions and memory decline [e.g., (Kalbe et al., 2016)] as well as difficulties regarding visuo-constructional functions (Lehrner et al., 2015). Moreover, accumulating evidence suggests that social cognition (SC) may also be impaired even in the early stages of PD [e.g., (Czernecki et al., 2021; Hipp et al., 2014; Ibañez et al., 2021; McIntosh et al., 2014)]. SC refers to the ability to appropriately recognize, interpret, remember, and respond to other people’s intentions, emotions, and behaviors (Happé et al., 2017). It encompasses several distinct yet overlapping functions: emotion recognition, empathy (including Theory of Mind/ToM) and social problem-solving (Doskas et al., 2024). Importantly, since the publication of the fifth edition of the Diagnostic and Statistical Manual of Mental Disorders [DSM-V; (American Psychiatric Association, 2013)], SC has been recognized as one of the six core neurocognitive domains, alongside attention, learning and memory, language, executive functions and perceptual-motor function. Here, we systematically review the literature on SC in patients with PD.

Social cognition is a complex construct comprising several distinct but interrelated aspects. In this systematic review, we focus on three well-conceptualized and widely studied aspects of SC. First, *emotion recognition* refers to the ability to accurately identify human emotions, either visually (i.e., facial or bodily expressions) or auditorily [i.e., prosody; (Scherer and Scherer, 2011)]. This foundational skill is considered a key building block for more complex aspects of SC (Adolphs, 1999, 2001; Frith and Frith, 2007; Van Overwalle, 2009). Second, *empathy* involves both understanding of others’ emotional experiences (i.e., cognitive empathy) and the affective response to emotions expressed by others [i.e., affective empathy (Cuff et al., 2016)]. In the current literature, the term ToM is frequently used to denote an individual’s empathic skills (Dvash and Shamay-Tsoory, 2014) that include, among others, the understanding of others’ thoughts, beliefs, intentions, and emotions (Baron-Cohen et al., 1997; Henry et al., 2013; Leslie et al., 2004). Broadly speaking, both empathy and ToM include cognitive and affective aspects, namely the ability to rationally understand and affectively grasp others’ feelings, beliefs and desires (Bartochowski et al., 2018; Cuff et al., 2016; Kalbe et al., 2010). Finally, the third aspect of SC addressed in the present systematic review is *social problem-solving/social decision-making*. This process involves resolving problems in naturalistic or “real-world” settings, encompassing inter- or intrapersonal challenges as well as broader community and societal issues (D’Zurilla et al., 2004).

Over the years, numerous reviews and meta-analyses have examined specific aspects of SC in PD [e.g., (Bora et al., 2015; Coundouris et al., 2019; Gray and Tickle-Degnen, 2010; Hazelton et al., 2025; Moonen et al., 2017)]. More recently, Doskas et al. (2024) provided a broader overview addressing all SC aspects specified above simultaneously. However, most of these works, including the latter, were not conducted systematically. Even the existing systematic reviews have been limited by their reliance on a restricted set of databases (such as PubMed and PsychINFO), thereby constraining their ability to comprehensively summarize and evaluate the current state-of-the-art regarding SC in PD. Accordingly, the present review was guided by the following question: In patients with PD, how are social cognition domains, including emotion recognition, ToM/empathy, and social problem-solving, altered at the self-report, behavioral, and neuroimaging levels compared with healthy controls and clinical populations? To address this question and the above-mentioned gaps, the present systematic review provides a large-scale literature search across multiple databases and integrates findings from behavioral, self-report, and neuroimaging studies. Finally, the overarching goal of the present work is to develop an integrative neurofunctional model of SC in PD.

## 2 Methods

### 2.1 Research protocol and scope of review

**FIGURE 1:**
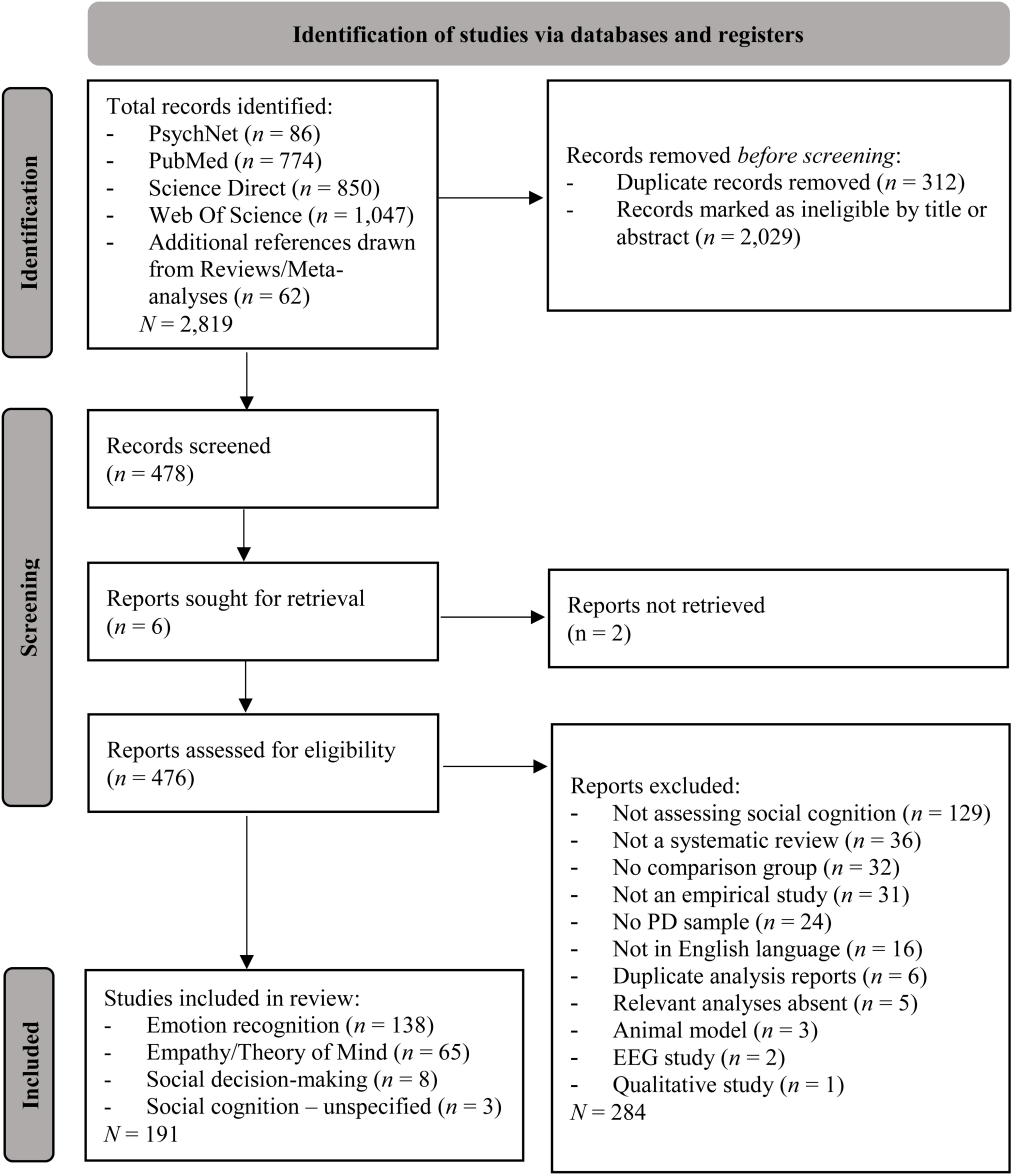
PRISMA flow diagram. PRISMA outline figure drawn from Page et al. (2021). In the included studies, the sum of papers per topic exceeds the total number of studies that were included in the review because some manuscripts examined two or more topics.

This systematic review was conducted in accordance with PRISMA guidelines (see Figure 1) and aims to provide a state-of-the-art synthesis of the literature on SC in PD, integrating findings from both behavioral and neuroimaging studies. In line with the review question stated above, eligibility criteria were defined as follows:

#### 2.1.1 Inclusion criteria

The inclusion criteria for the current systematic review were as follows: (1) Quantitative studies. (2) Reviews, meta-analyses and behavioral/neurocognitive studies (i.e., pencil-paper or PC-administered tests) and self-reports (i.e., questionnaires), in cross-sectional or longitudinal designs. For studies with a longitudinal design, it was required that baseline (T1) data included a relevant comparative analysis between the PD group and at least one other comparison group. (3) Studies including at least one between-group comparison. (4) One of the study groups must be elderly with PD. (5) Studies had to investigate SC [specifically, emotion recognition (including face and prosody), empathy (including ToM) and social problem-solving]. (6) Studies had to be written in English.

#### 2.1.2 Exclusion criteria

The exclusion criteria were as follows: (1) Qualitative studies. (2) Non-empirical studies. (3) Electrophysiological studies. (4) Studies not written in English. (5) Animal models. (6) Longitudinal studies with only one study group, that is, within-subject designs without a comparison group. (7) Non-systematic reviews.

#### 2.1.3 Search terms and databases

The literature search was conducted through July 2025 using four primary databases: PubMed, PsychNet, ScienceDirect, and Web of Science. Database outputs were sorted by relevance using the default ranking options available within each database. Initial screening was conducted at the title level; when eligibility could not be determined from the title alone, the abstract was briefly reviewed to determine whether the record addressed PD and SC. Screening within each search output continued until no further potentially relevant records appeared in the relevance-ranked results. Because databases differed in their ranking algorithms and output structure, the stopping point varied across databases and search combinations. When a review or meta-analysis was identified – regardless of whether it was ultimately included – its reference list was screened for additional studies that might fit the scope of the current systematic review. Across databases and reference-list searches, 2,819 records were identified. After removal of duplicates and title/abstract-based exclusions, 478 records were screened, and 476 reports were assessed for eligibility. Records identified as potentially relevant during the initial screening stage were then reviewed in full against the predefined inclusion and exclusion criteria. Prior to data extraction, two authors (MY and LK) defined the extraction framework and the specific variables to be coded for each study. At the beginning of the screening and extraction process, a pilot calibration procedure was conducted by the first author and a trained research assistant. Approximately 20% of the initially selected papers were jointly reviewed to establish a consistent approach to eligibility decisions, identify relevant data, and standardize how extracted information was entered into the Zotero project. The remaining studies were extracted according to the agreed framework, and ambiguous cases were discussed until consensus was reached. This procedure was used to minimize extraction errors and improve consistency across studies. *N* = 191 studies were included in the final review (see Figure 1 for further details). The search was conducted using the following keywords, which were combined in all relevant permutations:

1. Social cognition, emotion recognition, empathy, Theory of Mind, social problem solving, social decision making
2. Parkinson
3. MRI (Magnetic Resonance Imaging), fMRI (functional MRI), VBM (Voxel Based Morphometry), gray matter, DTI (Diffusion Tensor Imaging), FA (Fractional Anisotropy), white matter

In the first step, combinations were generated without any neuroimaging-related terms. Search strings were as follows: (“Social cognition” OR “emotion recognition” OR “empathy” OR “Theory of Mind” OR “social problem-solving” OR “social decision making”) AND “Parkinson.” In the second step, the search was repeated with the addition of one neuroimaging keyword to each combination. Search strings were as follows: (“Social cognition” OR “emotion recognition” OR “empathy” OR “Theory of Mind” OR “social problem-solving” OR “social decision making”) AND “Parkinson” AND (“MRI” OR “fMRI” OR “VBM” OR “gray matter” OR “DTI” OR “FA” OR “white matter”). Because the databases differed in their search interfaces and syntax, the search terms were entered into the available keyword/search fields of each database while preserving the same Boolean structure across searches. Given that only one empirical study has jointly examined all three aspects of SC, we present the included studies separately according to the following three SC aspects (1) emotion recognition: behavioral vs. neural correlates, (2) empathy/ToM: behavioral vs. neural correlates. (3) social problem-solving: primarily behavioral findings, as the literature on social problem-solving in PD is scarce and only one study has reported neuroimaging findings.

As a result of this systematic methodology, *N* = 191 studies were included in the review; *n* = 3 about SC in general (*n* = 1 at the behavioral level and *n* = 3 at the neuroanatomical/neurofunctional level); *n* = 138 about emotion recognition (*n* = 1 at the self-reported level, *n* = 133 at the behavioral level and *n* = 18 at the neuroanatomical/neurofunctional level); *n* = 65 about empathy and ToM (*n* = 3 at the self-reported level, *n* = 62 at the behavioral level and *n* = 6 at the neuroanatomical/neurofunctional level); and finally, *n* = 8 about social problem-solving (*n* = 2 at the reported level, 6 at the behavioral level and *n* = 1 at the neuroanatomical/neurofunctional level). Note that the sum of papers per topic (i.e., one of the three previously mentioned aspects of SC) exceeds the total number of studies that were included in the review because some manuscripts examined two or more SC aspects at once. For each paper included in the systematic review, the following data were extracted:

(1) Sample characteristics:

(a) Group sizes and matching criteria

(b) PD: type and disease duration

(c) Medication state during the study (ON/OFF medication)

(d) Hoehn and Yahr score

(e) UPDRS-III (Unified Parkinson’s Disease Rating Scale-III)

(f) Gender

(g) Age

(h) Presence of other neurological diseases (e.g., dementia, cerebral tumor, stroke)

(i) Presence of other major psychopathologies (e.g., major depression)

(j) Country of origin and native language

(2) Method:

(a) Study type: review, meta-analysis, behavioral/neurocognitive study, and/or self/others-report study

(b) Instruments used to quantify the following variables:

(i) SC aspect that was measured

(ii) Name of the task

(iii) Type of stimulus (face/voice etc., Ekman faces/other etc.)

(iv) Task conditions

(v) Operationalization

(3) Results: main findings related to the scope of the systematic review

All summarized data (as well as the full reference list) are publicly available in the Zotero file attached as Supplementary Appendix A. At the end of this systematic review, we propose a comprehensive empirical neuroanatomical model of the three SC aspects that are the focus of the present study. Finally, we discuss gaps in the literature on SC in PD, as well as potential directions for future research.

#### 2.1.4 Risk-of-bias assessment and quality of evidence

Risk-of-bias appraisal was conducted by the first author using predefined decision rules. For the included studies the assessment was performed using a set on eight tailored questions that were based on the widely used JBI critical appraisal framework (Barker et al., 2023) developed for comparative studies of SC in PD. Because all included studies were required to include a PD group and a control group, the appraisal focused on methodological domains most relevant to this design: clarity of inclusion and exclusion criteria, characterization of the PD and control samples, validity of PD diagnosis, group comparability or matching, identification and handling of confounding factors, validity and reliability of SC and neuroimaging outcomes, and appropriateness of statistical analyses. Meta-analyses and reviews that were included in the current review were separately assessed using eleven questions that were fully aligned with the JBI guidelines stated in Aromataris et al. (2015). Each item was rated as “Yes,” “Mostly Yes,” “No,” or “Unclear” according to predefined decision rules. The full appraisal criteria and rating definitions are provided in Supplementary Appendix B.

#### 2.1.5 Likelihood of publication bias

Because the present review was designed as a broad integrative systematic review rather than a quantitative meta-analysis, formal statistical assessment of publication bias, such as funnel-plot asymmetry or Egger’s test, was not performed. Nevertheless, publication bias cannot be ruled out, particularly because studies reporting significant group differences in SC may be more likely to be published than those reporting null findings. In addition, the restriction to English-language publications may have introduced language (and thus, cultural) bias. To partially mitigate the risk of missing relevant studies, we searched multiple databases and screened the reference lists of relevant reviews and meta-analyses. In addition, studies were considered eligible even when SC was not the primary outcome, provided that relevant SC data were reported. Findings were extracted based on the statistical results reported in the Results sections rather than on the authors’ interpretations in the Discussion sections, in order to reduce interpretive and selective-emphasis bias. Lastly, we aimed to minimize selective interpretation by reporting both significant and non-significant findings within each domain.

## 3 Results

The results section is structured according to the three SC key aspects defined in the introduction, namely emotion recognition, empathy/ToM, and social problem-solving. Within each aspect, we first present the behavioral findings, followed by the neuroimaging literature aimed at elucidating the neurocognitive correlates of SC in PD.

### 3.1 Emotion recognition

Most of the published literature on SC in individuals with PD focuses on emotion recognition at the behavioral level, making this the most detailed section of our review. Given that emotion recognition is a broad construct – ranging from the widely studied recognition of facial stimuli to the interpretation of emotions conveyed through scenic images and prosody – it is not surprising that the current evidence is mixed. While some behavioral studies reported significant impairments in emotion recognition among individuals with PD compared to healthy controls (HC) [e.g., (Aiello et al., 2014; Beatty et al., 1989; Ciccarelli et al., 2022; Dujardin et al., 2004; Garcia-Rodriguez et al., 2012; Sedda et al., 2017; Seubert-Ravelo et al., 2021; Yip et al., 2003)] or other clinical populations [e.g., (Chiang et al., 2024; Martínez-Corral et al., 2010; Pontieri et al., 2012)], a comparable number of behavioral studies did not find significant group differences in this domain [e.g., (Adolphs et al., 1998; Bediou et al., 2012; Bek et al., 2020; Benke et al., 1998; Martinez et al., 2018; Pell and Leonard, 2005; Péron et al., 2010a; Pietschnig et al., 2016; Wabnegger et al., 2015)]. These discrepancies may be partly attributable to methodological differences, including the wide variety of task designs (e.g., identification, discrimination, naming, and other experimental paradigms) and stimulus types (e.g., visual vs. vocal, gray scale vs. color, 2D vs. 3D, static vs. dynamic, realistic vs. schematic). In addition, substantial heterogeneity in PD sample characteristics and sample sizes further complicates the interpretation of the existing literature (see Table 1). As shown in Table 1, sample sizes were generally small, with particularly high variability in the severity of Parkinson-related motor symptoms {as indexed by the Unified Parkinson’s Disease Rating Scale scores [UPDRS-III; (Movement Disorder Society Task Force on Rating Scales for Parkinson’s Disease, 2003)]}.

**TABLE 1:** Pooled descriptive statistics of Parkinson’s disease (PD) samples from studies investigating emotion recognition.

Variable name	*N*	*M*	SD	Min	Max	Skew
PD sample *N* size	121	30.94	23.51	10	176	3.36
Sex (males %)	116	59%	13%	0%	93%	−0.65
Age	116	63.71	4.42	51.10	74	−0.05
Disease duration	89	7.16	3.04	0.62	17.32	0.56
Hoehn and Yahr	64	1.95	0.50	0.95	3.03	−0.22
UPDRS-III	71	21.60	9.04	4.04	40.10	0.13

Age and disease duration are reported in years. All statistics were calculated based on available data regarding mean values. Manuscripts that did not report mean values were omitted from these calculations. For studies that included comparisons between different PD populations, values were averaged to provide a single estimate per paper.

In the following sections, we differentiate the reviewed behavioral studies according to the paradigms and modalities (visual/facial expression versus vocal/prosody) employed to assess emotion recognition. The most used paradigm is the *emotion identification task*, in which participants are asked to select the expressed emotion from a set of predefined options. Next, we report findings related to the *emotion discrimination or matching paradigms*, in which participants indicate whether two presented faces express the same or different emotions, or alternatively choose, from two options, the face that matches a target face. We then present studies that employed the *emotion naming paradigm*, which requires participants to verbally label the emotion being expressed. Finally, we summarize findings from *other paradigms* that have been applied, such as emotional Stroop tasks combining facial expressions and verbal stimuli, face–body matching tasks, and emotional face memory tasks, among others. In addition, some studies employed dynamic facial/video paradigms, which resembled traditional emotion identification tasks but incorporated dynamic visual stimuli. Other studies employed mixed-modality paradigms, in which facial and prosodic emotional cues were presented simultaneously.

#### 3.1.1 Emotion identification

##### 3.1.1.1 Face identification

The most frequently used paradigm in this field is the identification of static facial expressions of basic emotions [happiness, surprise, anger, disgust, fear, and sadness; (Ekman, 1992)]. Sixty studies examined total scores in comparison with healthy controls (HC). While 32 studies reported significant emotion identification impairments in individuals with PD relative to HC [e.g., (De Risi et al., 2018; Enrici et al., 2015; Hazelton et al., 2023; Herrera et al., 2011; Ibarretxe-Bilbao et al., 2009; Martins et al., 2024; Mattavelli et al., 2021; Palmeri et al., 2020)], 36 studies reported no significant group differences [e.g., (Adolphs et al., 1998; Albuquerque et al., 2016; Assogna et al., 2010; Bauer et al., 2023; Blonder et al., 1989; Chuang et al., 2022; Di Tella et al., 2021; Enrici et al., 2017; Fleury et al., 2014; Le Jeune et al., 2008)]. Studies reporting significant effects tended to include larger samples and patients with longer disease duration and more severe motor symptoms. Notably, the use of total scores may lack sensitivity and thus reduce the likelihood of detecting significant group differences.

Among studies reporting subscores, the most consistent finding (29 reports; 91%) is that individuals with PD in the “on” medication state (i.e., receiving dopaminergic medication) did not differ from HC in identifying *happy* faces (Assogna et al., 2010; Baggio et al., 2012; Bologna et al., 2016; Chen et al., 2019; Chuang et al., 2022; Ciccarelli et al., 2022; Clark et al., 2010; Czernecki et al., 2021; De Risi et al., 2018; Dujardin et al., 2004; Enrici et al., 2015, 2017; Fleury et al., 2014; Jin et al., 2017; Kalampokini et al., 2018; Le Jeune et al., 2008; Mattavelli et al., 2021; Narme et al., 2011, 2013; Palmeri et al., 2020; Péron et al., 2010a; Ricciardi et al., 2015; Saenz et al., 2013; Salfi et al., 2024; Sedda et al., 2017; Sprengelmeyer et al., 2003; Waldthaler et al., 2019) or faces expressing *surprise* (15 reports; 65%) (Czernecki et al., 2021; Ciccarelli et al., 2022; Dujardin et al., 2004; Péron et al., 2010a; Assogna et al., 2010; Baggio et al., 2012; Bologna et al., 2016; Chuang et al., 2022; De Risi et al., 2018; Enrici et al., 2015; Fleury et al., 2014; Le Jeune et al., 2008; Salfi et al., 2024; Sprengelmeyer et al., 2003; Waldthaler et al., 2019). Taken together, these results suggest that the identification of positive facial emotions seems to be relatively preserved in PD patients treated with dopaminergic medication.

Findings regarding negative facial emotions were heterogeneous. Even among studies using black-and-white stimuli, more than half reported null effects, whereas null effects were reported consistently across all studies using colored facial stimuli (Assogna et al., 2010; Chuang et al., 2022; Jin et al., 2017; Waldthaler et al., 2019). Nevertheless, significant group differences (favoring HC) were reported for identification of *disgust* (Assogna et al., 2010; Baggio et al., 2012; Bologna et al., 2016; Chuang et al., 2022; Cousins et al., 2021; Ibarretxe-Bilbao et al., 2009; Mattavelli et al., 2021; Palmeri et al., 2020; Sedda et al., 2017), *sadness* (Czernecki et al., 2021; Sedda et al., 2017; De Risi et al., 2018; Ibarretxe-Bilbao et al., 2009; Mattavelli et al., 2021; Palmeri et al., 2020; Baggio et al., 2012; Bologna et al., 2016; Jin et al., 2017; Narme et al., 2013; Ricciardi et al., 2017; Saenz et al., 2013; Salfi et al., 2024), *fear* (Czernecki et al., 2021; Ciccarelli et al., 2022; Sedda et al., 2017; De Risi et al., 2018; Ibarretxe-Bilbao et al., 2009; Baggio et al., 2012; Cousins et al., 2021; Mattavelli et al., 2021; Narme et al., 2013; Palmeri et al., 2020; Ricciardi et al., 2017; Saenz et al., 2013; Salfi et al., 2024; Sprengelmeyer et al., 2003) and *anger* (Ciccarelli et al., 2022; Sedda et al., 2017; De Risi et al., 2018; Enrici et al., 2015; Ibarretxe-Bilbao et al., 2009; Baggio et al., 2012; Bologna et al., 2016; Clark et al., 2010; Cousins et al., 2021; Mattavelli et al., 2021; Narme et al., 2011; Palmeri et al., 2020; Ricciardi et al., 2017; Salfi et al., 2024; Sprengelmeyer et al., 2003; Vélez Feijó et al., 2008), corresponding to 32%, 41%, 44%, and 50% of the reviewed papers in this section, respectively. Overall, despite substantial heterogeneity, these findings suggest a broader tendency toward impaired recognition of negative emotions in PD (Glozman et al., 2003). Further support for this claim comes from within-subject comparisons, showing that, compared with HC, patients with PD exhibit impaired recognition of negative relative to positive emotions (Adolphs et al., 1998; Aiello et al., 2014; Hipp et al., 2014; Kan et al., 2002; Lawrence et al., 2007; Yip et al., 2003). Notably, studies that included older participants were more likely to find deficiencies in facial emotion recognition in patients with PD. Additional evidence suggests that individuals with PD may be more likely to misidentify emotions as *surprise* (Argaud et al., 2016; Dujardin et al., 2004), and may have greater difficulty with less prototypical stimuli (Buxton et al., 2013; Dujardin et al., 2004).

Subgroup analyses further qualified this pattern. When HC were compared with patients with PD in the “off” medication state, the most consistent impairment was observed in fear recognition (Martins et al., 2008; Mondillon et al., 2012; Sprengelmeyer et al., 2003). In *de novo* or early-stage PD, Slomp et al. (2024) reported impaired identification of disgust and sadness, while other studies reported somewhat different patterns of deficits (Hipp et al., 2014; Peron et al., 2014; Suzuki et al., 2006). Importantly, however, all studies reported significant group differences favoring HC in the identification of negative emotions. Evidence from studies comparing patients with PD and mild cognitive impairment (PD-MCI) with HC is similarly inconclusive. While Chiang et al. (2024) found no significant group differences, other studies reported that patients with PD-MCI had greater difficulty in the recognition of anger (Waldthaler et al., 2019) and identifying surprise, fear, and sadness (Dodich et al., 2022).

The heterogeneous pattern of findings also extends to comparisons involving PD subgroups and other clinical populations. Several studies reported comparable total facial emotion identification scores between patients with PD and patients with PD following deep brain stimulation (DBS) surgery (Enrici et al., 2017; McIntosh et al., 2014), patients with PD-MCI (Pietschnig et al., 2016), patients with PD without cognitive impairment [PD-NC; (Pietschnig et al., 2016)], patients with PD tested in the “off” medication state (Fleury et al., 2014), patients with atypical parkinsonian syndromes such as multiple system atrophy [MSA; (Sidoroff et al., 2024)], and non-Parkinsonian patients with right-brain damage (Borod et al., 1990) or supratentorial infarction (Adamaszek et al., 2019). These findings suggest that facial emotion identification impairments are not uniformly observed across PD subgroups or when PD is compared with other neurological conditions.

One factor that may contribute to this heterogeneity is the inconsistent characterization of cognitive status across studies. Although most studies stated that their PD samples did not present significant cognitive difficulties, some did not provide a clear description of participants’ cognitive status. Consequently, in several studies, it remains unclear whether participants had intact cognitive abilities, PD-MCI, or a more advanced form of cognitive impairment. This ambiguity may partly account for inconsistencies across samples. Indeed, the few studies that directly compared PD-MCI and PD-NC also yielded mixed results. Some reported specific impairments in facial emotion identification among patients with PD-MCI (Chiang et al., 2024; Dodich et al., 2022), with group differences primarily driven by the identification of *sadness* and *anger* (Dodich et al., 2022), whereas others found no significant differences between PD-MCI and PD-NC in this domain (Fernández-Fernández et al., 2024; Pietschnig et al., 2016; Waldthaler et al., 2019).

Comparisons between PD and other clinical populations have likewise produced inconsistent findings. Studies comparing PD with major depressive disorder (MDD), Alzheimer’s disease, or progressive supranuclear palsy reported either no significant group differences (Borod et al., 1990; Martinez et al., 2018; Martins et al., 2024) or poorer emotion identification performance in PD (Bediou et al., 2012; Pontieri et al., 2012). Finally, individual-difference factors may further modulate performance. Sex differences favoring women in emotion identification have been reported in patients with PD (Heller et al., 2018), and this effect may be particularly evident among patients with higher, compared with lower, levels of apathy (Martínez-Corral et al., 2010).

##### 3.1.1.2 Prosody identification

Inconsistent findings also extend to prosody. Six studies reported impaired prosody recognition in PD (Aiello et al., 2014; Benke et al., 1998; Blonder et al., 1989; Clark et al., 2008; Dara et al., 2008; Kan et al., 2002; Péron et al., 2010a), whereas seven others did not (Borod et al., 1990; Buxton et al., 2013; McIntosh et al., 2014; Pell and Leonard, 2003; Saffarian et al., 2019; Schröder et al., 2006). Findings are more consistent when HC are compared with individuals with more advanced PD (Albuquerque et al., 2016; Breitenstein et al., 1998; Peron et al., 2014; Yip et al., 2003), or with those with verbal memory impairments (Benke et al., 1998). Specifically, Breitenstein et al. (2001) demonstrated that compared with HC, individuals with advanced PD tend to rely more on sentence content than on prosody to determine emotional valence.

When focusing on basic emotions, the literature remains relatively limited, with most studies neglecting to examine surprise or disgust. Schröder et al. (2006) reported that, compared to HC, patients with PD exhibited impaired recognition of negative emotions, but not positive or neutral prosody; partially aligning with other findings (Di Tella et al., 2021; McIntosh et al., 2014)). However, Di Tella et al. (2021), McIntosh et al. (2014) reported similar performance levels for disgust and fear in patients with PD and HC, thereby contradicting Schröder et al.’s (2006) broader impairment findings. A moderating role of motor symptom laterality has also been suggested (Stirnimann et al., 2018; Voruz et al., 2020, 2024), with left-PD patients (meaning, predominantly left-sided motor symptoms) showing impaired recognition of happiness and neutral prosody compared to right-PD patients and HC, while right-PD patients (i.e., predominantly right-sided motor symptoms) performed similarly to HC.

#### 3.1.2 Emotion discrimination

##### 3.1.2.1 Facial discrimination

Twenty-two studies employed facial emotion discrimination tasks. Among studies comparing medicated patients with PD with HC, five reported significant emotion discrimination deficits in patients with PD (Aiello et al., 2014; Ariatti et al., 2008; Bauer et al., 2023; Jacobs et al., 1995; Ulusoy et al., 2015), whereas eight studies found no significant group differences (Alonso-Recio et al., 2014a; Blonder et al., 1989; Borod et al., 1990; Burgio et al., 2024; Dan et al., 2019; Delaveau et al., 2009; Pell and Leonard, 2003; Tessitore et al., 2002). Interestingly, even after controlling for disease duration (by differentiating and comparing early-onset, late-onset, or advanced PD to HC) group differences in emotion discrimination remained non-significant (Albuquerque et al., 2016; Haeske-Dewick, 1996). However, disease progression (indexed by bilateral motor symptoms) seems to be a risk factor for developing emotion discrimination deficits (Breitenstein et al., 1998; Yip et al., 2003). When compared with other clinical groups, patients with PD performed better than individuals with schizophrenia (Borod et al., 1990), but similarly to those with right-brain damage or supratentorial infarction (Adamaszek et al., 2019; Borod et al., 1990), as well as to individuals with MDD (Borod et al., 1990) or MCI (Burgio et al., 2024). No group differences in emotion discrimination skills were found between patients with bilateral and unilateral motor symptoms (Breitenstein et al., 1998).

##### 3.1.2.2 Prosody discrimination

Findings are relatively consistent in suggesting that individuals with PD perform similarly to HC on prosody discrimination tasks (Borod et al., 1990; Breitenstein et al., 1998; Pell and Leonard, 2003; Stirnimann et al., 2018; Yip et al., 2003). However, impairments might emerge in more advanced PD (Albuquerque et al., 2016; Breitenstein et al., 1998; Yip et al., 2003). Consistent with findings across other modalities, performance levels of patients with PD resembled that of individuals with MDD or stroke (Adamaszek et al., 2019; Borod et al., 1990; Breitenstein et al., 1998). Similarly, patients with unilateral and bilateral motor symptoms displayed comparable performance levels (Breitenstein et al., 1998).

#### 3.1.3 Emotion naming

##### 3.1.3.1 Facial naming

Only three studies examined emotion naming using facial stimuli. Ariatti et al. (2008) reported general impairments in PD compared with HC, while Breitenstein et al. (1998) found deficits primarily among individuals with bilateral motor symptoms. Emotion-specific effects included impairments in *anger* and *sadness* (Ariatti et al., 2008; Ho et al., 2020), as well as *disgust* and *surprise* (Ho et al., 2020). When compared to other clinical groups, patients with PD outperformed those with cerebellar infarction and performed similarly to those with supratentorial infarction (Adamaszek et al., 2019). Breitenstein et al. (1998) also reported no performance differences in emotion naming between patients with unilateral and bilateral PD motor symptoms.

##### 3.1.3.2 Prosody naming

Only two studies examined prosody naming. Consistent with findings on facial emotion naming, Ariatti et al. (2008) reported a general impairment in PD compared with HC, while Breitenstein et al. (1998) suggested that impairments were specific to patients with bilateral motor symptoms. Emotion-specific effects included reduced recognition of *happiness* (Ariatti et al., 2008).

#### 3.1.4 Other paradigms

All studies using other paradigms focused on facial emotion recognition, except for one study that focused on prosody (Mitchell and Boucas, 2009) and reported no group differences between PD and HC when on n-back or Stroop-like tasks. With regards to facial emotion recognition, it was evident that the performance of patients with PD is highly dependent on task design and sample characteristics. Among the three studies employing an emotional Stroop-like word-face task (Alonso-Recio et al., 2014b; Fleury et al., 2014; Irmen et al., 2017), one study (Fleury et al., 2014) found no group differences in accuracy or Stroop effect size, while the other two studies reported reduced accuracy [i.e., indicating difficulty inhibiting incongruent facial emotional information when participants were asked to read emotional words (Alonso-Recio et al., 2014b)] or selective impairments in processing conflicting information regarding sad – but not happy – faces (Irmen et al., 2017). In a body–face congruency task, patients with PD relied more heavily on body cues than on facial cues, unlike HC or patients with schizophrenia (Foul et al., 2024).

Other cognitive paradigms (e.g., memory paradigm of emotional faces and visual search of emotional faces) largely showed no group differences in accuracy or response patterns (Burgio et al., 2024; Lundqvist et al., 2017). In dual-task conditions, individuals with newly diagnosed PD showed a greater performance cost than HC (i.e., lower accuracy following the addition of a second task) in facial recognition (Garcia-Rodriguez et al., 2012). Most interestingly, eye-tracking studies also revealed subtle group differences upon processing emotional stimuli: compared with HC, patients with PD exhibited a more diffuse gaze distribution, focusing less on diagnostically relevant areas of the face (50,90), despite average performance regarding number of fixations and the fixation durations (Clark et al., 2010). Notably, lateralized effects were also noted (Smith et al., 2010; Thomasson et al., 2022) patients with right-sided motor symptoms showed a bias toward perceiving the left half of the face as happier (Smith et al., 2010).

Similar to the findings reported above, results from dynamic facial/video paradigms (which better approximate real-life emotion recognition) were ambiguous, providing evidence for both non-significant (Anzani et al., 2024; Bauer et al., 2023; Bek et al., 2020; Coundouris et al., 2022; Sidoroff et al., 2024) and significant group differences between PD and HC (Argaud et al., 2016; Bediou et al., 2012; Ho et al., 2020; Kuehne et al., 2023; Martinez et al., 2018; McIntosh et al., 2014; Siquier and Andrés, 2022). Notably, patients with PD were found to exhibit emotion-specific impairments when processing dynamic facial/video paradigms (Kan et al., 2002; McIntosh et al., 2014; Siquier and Andrés, 2022) and required more frames (e.g., more slides of the changing face from neutral to emotional face) to correctly identify emotions (Kuehne et al., 2023), which is consistent with the claim that they perceive authentic emotional expressions as less genuine (Anzani et al., 2024).

Finally, in mixed paradigms requiring face–prosody matching, Breitenstein et al. (1998) reported impaired performance of PD patients with bilateral motor symptoms compared with HC, whereas patients with unilateral motor symptoms showed preserved emotion recognition [however, see (Bauer et al., 2023) for somewhat different findings]. Other studies combined face and prosody measures into a general score and consistently found impairments in PD (Paulmann and Pell, 2010; Shafiei et al., 2020). Eriksson et al. (2022) reported impairments limited to positive emotions, but because their analyses incorporated both basic and complex emotions, further investigation is warranted.

#### 3.1.5 Interim summary on behavioral findings related to emotion recognition

Although findings on emotion recognition in PD are inconclusive, most reviewed papers indicate that patients with PD are more likely to show impairments in negative emotion recognition. However, these deficits are moderated by several factors. First, the type of task and the modality of emotion presentation (facial/prosodic, static/dynamic, mixed, and so forth) are crucial for determining whether impairments are detected. Second, sample characteristics play a key role: Patients with PD at more advanced disease stages or with greater and/or bilateral motor impairments tend to show stronger deficits. Importantly, the reviewed studies highlight the criticality of more detailed reporting of sample characteristics. Moreover, many studies do not provide sufficient detail on patients’ disease duration, motor symptoms severity, or PD type, thereby limiting the generalizability of their findings. Overall, these results suggest the need for careful experimental design, precise sample characterization, and consideration of task modality.

#### 3.1.6 Imaging studies on emotion recognition

The following section includes fifteen empirical papers as well as three systematic reviews/meta-analyses. Of the 15 empirical papers, two did not report any neural correlates for emotion recognition in patients with PD (Hazelton et al., 2023; Trompeta et al., 2023), and two did not include a HC group for comparison (Funghi et al., 2025; Robert et al., 2014). However, given the limited literature on this topic, we included these studies in the present review.

A recent meta-analysis of studies published through 2023 (Hazelton et al., 2025) examined neuroimaging research on interoception, emotion, and social cognition in neurodegenerative diseases, including PD. Although this meta-analysis did not focus specifically on emotion recognition, it reported that neurofunctional activations related to emotion processing (either the recognition of emotions in others or experience of one’s own emotions) in PD involve the frontal poles and central opercular cortices bilaterally, as well as the left planum temporale and postcentral gyrus. Furthermore, two systematic reviews (Ibarretxe-Bilbao et al., 2011; Moonen et al., 2017) indicated that patients with PD and impaired emotion recognition tend to exhibit reduced gray matter volumes in the orbitofrontal cortex (OFC) and amygdala, although these findings are not entirely consistent. Notably, Moonen et al. (2017) proposed that emotional processing relies on two main neural systems: a ventral, bottom-up pathway responsible for emotion perception and automatic affective responses [including the amygdala, ventral striatum, ventral anterior cingulate cortex (ACC), OFC, ventrolateral prefrontal cortex (VLPFC), and hypothalamus] and a dorsal pathway involved in top-down regulation and cognitive control of emotions (including the DLPFC, dorsal ACC, and hippocampus). In PD, the ventral dopaminergic system seems to be primarily compromised (potentially contributing to deficient emotion perception and automatic emotional responses), while the dorsal system may provide compensatory regulation via cognitive control mechanisms (as indicated by increased prefrontal recruitment). Importantly, these theoretical and empirical findings largely correspond with other empirical research on the neural correlates of emotion recognition in PD (see Table 2 and Supplementary Appendix C for a detailed summary).

**TABLE 2:** Summary of neuro-imaging correlations between emotion recognition tasks and functional/anatomical indexes for PD samples.

References	Task	Measure	Region	MNI coordinates			Correlated with
				Z	Y	X	
Total score							
Li et al., 2022	Emotion identification	MRI – cortical thickness	Left caudal middle frontal	−**38.3**	**13.4**	**51.6**	Median RT (−)
		MRI – cortical thickness	Left precentral	−**54.4**	**3.8**	**31.0**	Median RT (−)
		MRI – cortical thickness	Left superior frontal	−**22.3**	**19.4**	**54.4**	Median RT (−)
		MRI – cortical thickness	Right temporal pole	**29.9**	**11.9**	−**36.0**	Median RT (−)
		MRI – cortical thickness	Right superior frontal	**6.9**	**53.4**	**32.5**	Median RT (−)
Robert et al., 2014	Emotion identification	PET - metabolism	Right precuneus	22**.22**	−**61.98**	44**.61**	Accuracy (+)
		PET – metabolism	Left inferior occipital gyrus	−**20.20**	−**92.35**	−**12.49**	Accuracy (+)
		PET – metabolism	Bilateral posterior cingulate cortex	4**.04**	**−45.41**	43**.31**	Accuracy (−)
		PET – metabolism	Right superior frontal gyrus	**20.20**	54**.46**	26**.82**	Accuracy (**−**)
				**38.38**	54**.46**	26**.82**	
				**24.24**	28	**62.42**	
		PET - metabolism	Left superior frontal gyrus	**−16.16**	54**.16**	**−11.17**	Accuracy (**−**)
		PET - metabolism	Left superior frontal gyrus	**−16.16**	52**.20**	31**.05**	Accuracy (**−**)
Tessitore et al., 2002	Emotion matching	fMRI – BOLD	Bilateral amygdala	**−16.16**	**−5.56**	**−14.62**	Accuracy (+)
				**14.14**	**−5.56**	**−14.62**	
		fMRI – BOLD	Bilateral ventral prefrontal cortex	**−48.48**	16**.73**	**−4.99**	Accuracy (+)
				**40.40**	23**.94**	**−4.58**	
		fMRI – BOLD	Bilateral inferior frontal gyrus	**−33.33**	8**.85**	30**.94**	Accuracy (+)
				**33.33**	**8.85**	30**.94**	
		fMRI – BOLD	Anterior cingulate cortex	0	24.61	47.01	Accuracy (+)
		fMRI – BOLD	Bilateral posterior fusiform gyrus	**−36.36**	**−54.98**	**−17.48**	Accuracy (+)
				**36.36**	**−54.98**	**−17.48**	
		fMRI – BOLD	Bilateral inferior occipital gyrus	**−42.42**	**−84.01**	**−14.39**	Accuracy (+)
				**42.42**	**−84.01**	**−14.39**	
Fernández-Fernández et al., 2024	Emotion identification	MRI – cortical thickness	Left superior frontal gyrus	NA	NA	NA	Accuracy (+)
Funghi et al., 2025	Emotion identification	MRI – VBM	Bilateral dorsomedial prefrontal cortex	Brainnetome Atlas’s ROI			Accuracy (+)
		MRI – VBM	Bilateral primary and secondary somatosensory cortex	Brainnetome Atlas’s ROI			Accuracy (+)
		MRI – VBM	Bilateral amygdala	Brainnetome Atlas’s ROI			Accuracy (+)
		MRI – VBM	Right anterior temporal cortex	Brainnetome Atlas’s ROI			Accuracy (+)
Ibarretxe-Bilbao et al., 2009	Emotion identification	MRI – VBM	Bilateral orbitofrontal cortex	**−**30	52	22	Accuracy (+)
				**30**	52	**22**	
Baggio et al., 2012	Emotion identification	MRI – VBM	Dorsal anterior cingulate cortex	2	18	24	Accuracy (+)
Happy							
Li et al., 2022	Emotion identification	MRI – cortical thickness	Left superior temporal cortex	**−41.8**	**9.7**	**−27.3**	Accuracy (+)
Stirnimann et al., 2018	Prosody	PET – metabolism	Right orbitofrontal cortex	**BA10** **NA**	**NA**	**NA**	Discrimination score (**−**)
Fear							
Fleury et al., 2014	Stroop-like task (incongruent – congruent)	fMRI – BOLD	Right middle frontal cortex	45	38	17	NA (+)
		fMRI – BOLD	Left middle temporal cortex	**−63**	**−31**	4	NA (+)
		fMRI – BOLD	Right inferior temporal cortex	39	**−64**	**−8**	NA (+)
Burgio et al., 2024	Emotional face memory task	MRI – VBM	Striatum	**NA**	**NA**	**NA**	LISAS (**−**)
		MRI – VBM	Right parietal regions	**NA**	**NA**	**NA**	LISAS (−)
Wabnegger et al., 2015	–	fMRI – BOLD	Right secondary somatosensory cortex	51	**−**9	21	Intensity and accuracy (+)
Anger							
Fernández-Fernández et al., 2024	Emotion identification	MRI – cortical thickness	Bilateral superior frontal gyrus	NA	NA	NA	Accuracy (+)
		MRI – cortical thickness	Left middle temporal gyrus	NA	NA	NA	Accuracy (+)
		MRI – cortical thickness	Right superior parietal cortex	NA	NA	NA	Accuracy (+)
		MRI – cortical thickness	Right precuneus	NA	NA	NA	Accuracy (+)
Wabnegger et al., 2015	–	fMRI – BOLD	Left inferior parietal cortex	**−**48	**−**69	33	Intensity (+)
Baggio et al., 2012	Emotion identification	MRI – VBM	Bilateral ventral striatum (nuclei accumbens)	6	4	**−**8	Accuracy (+)
				**−**6	4	**−**6	
		MRI – VBM	Right occipital fusiform gyrus	24	**−**64	**−**10	Accuracy (+)
		MRI – VBM	The subgenual cortex	6	10	**−**10	Accuracy (+)
Disgust							
Fernández-Fernández et al., 2024	Emotion identification	MRI – cortical thickness	Left superior frontal gyrus	NA	NA	NA	Accuracy (+)
		MRI – cortical thickness	Right fusiform gyri	NA	NA	NA	Accuracy (+)
Wabnegger et al., 2015	–	fMRI – BOLD	Left secondary somatosensory cortex	**−**36	**−**27	21	Intensity and accuracy (+)
Baggio et al., 2012	Emotion identification	MRI – VBM	Dorsal anterior cingulate cortex	2	**16**	22	Accuracy (+)
Sadness							
Baggio et al., 2012	Emotion identification	MRI – VBM	Right lateral and medial OFC	40	30	**−**20	Accuracy (+)
				4	42	**−**28	
		MRI – VBM	Right amygdala	16	**−**6	**−**12	Accuracy (+)
		MRI – VBM	Right postcentral gyrus	34	**−**32	50	Accuracy (+)
		MRI – FA	Bilateral inferior fronto-occipital fasciculus, including right forceps minor	34	**37**	4	Accuracy (+)
				**−**40	**−**25	**−**5	
		MRI – FA	Left inferior longitudinal fasciculus	**−**43	**−**24	**−**11	Accuracy (+)
		MRI – FA	Corpus callosum to the left centrum semiovale	**−**9	5	17	Accuracy (+)
Di Tella et al., 2021	Emotion identification	MRI – VBM	Bilateral striatum dorsal (caudate and putamen)	NA	NA	NA	Hu scores (+)

+/**−** symbol represents correlation direction between the region and the operational measurement. Hu scores, combined scores of correct identification and incorrect identification; LISAS, combined scores of accuracies and reaction time. Coordinates reported in Talairach space were transformed into MNI space using the tal2mni transformation (Lancaster et al., 2007). BA, Brodmann area; BOLD, blood oxygenation level dependent; FA, fractional anisotropy; OFC, orbitofrontal cortex; PET, positron emission tomography; MRI, magnetic resonance imaging, ROI, region of interest, VBM, voxel-based morphometry.

Converging evidence across studies identifies several brain regions that seem to predominantly modulate emotion recognition in PD. These include the bilateral superior frontal gyrus (Fernández-Fernández et al., 2024; Li et al., 2022; Robert et al., 2014), bilateral amygdala (Baggio et al., 2012; Funghi et al., 2025; Ibarretxe-Bilbao et al., 2011; Tessitore et al., 2002) and orbitofrontal cortex (Baggio et al., 2012; Ibarretxe-Bilbao et al., 2009, 2011). The striatum and its subregions (nucleus accumbens, caudate, putamen) appear particularly relevant for processing negative emotions such as *fear, anger*, and *sadness* (Baggio et al., 2012, Burgio et al., 2024; Di Tella et al., 2021), while the temporal cortex may contribute selectively to processing happy or fearful faces (Fleury et al., 2014; Li et al., 2022). Moreover, the right precuneus and fusiform gyrus have been repeatedly associated with emotion recognition (Robert et al., 2014; Rodríguez-Antigüedad et al., 2024; Tessitore et al., 2002). Finally, the postcentral gyrus may also play a key role in emotion recognition, as reduced gray matter volumes or weaker connectivity between this region and the amygdala have been associated with poorer overall emotion recognition (Baggio et al., 2012; Funghi et al., 2025).

### 3.2 Empathy and ToM

Although the literature on empathy and ToM in patients with PD is sparser than the literature on emotion recognition, it appears relatively more consistent. Six systematic reviews and meta-analyses, each including between 6 and 38 studies, provide converging evidence that empathy and ToM deficits are common in PD (Bodden et al., 2010a; Bora et al., 2015; Coundouris et al., 2020; Desmarais et al., 2018; Doskas et al., 2024; Stafford et al., 2023). Although some of these studies placed greater emphasis on the heterogeneity of ToM manifestations (Bodden et al., 2010a; Stafford et al., 2023), others suggested that empathy and ToM deficits emerge primarily at later disease stages (Desmarais et al., 2018). Although Coundouris et al. (2020) proposed that affective empathy – the ability to resonate emotionally with others – remains preserved, all reviews and meta-analyses report impairments in both cognitive and affective aspects of empathy/ToM. At the behavioral level, however, none of the published studies directly assessed patients’ ability to resonate affectively, highlighting a major gap in the literature on affective empathy. Below, we first present findings based on general ToM scores (beyond affective or cognitive components) and questionnaires, followed by findings related to affective ToM and cognitive empathy as well as cognitive ToM. For the sake of clarity, we use the term affective ToM hereafter to refer to both affective ToM and cognitive empathy.

#### 3.2.1 General scores and questionnaires

Questionnaires appear to be less sensitive for detecting ToM difficulties in patients with PD. With the exception of Narme et al. (2013) who reported that caregivers rated patients with PD as more impaired than HC, studies using caregiver reports (Martinez et al., 2018) or self-reports (Alonso-Recio et al., 2021; Sarasso et al., 2021) yielded null results. Likewise, findings from studies using general scores are inconsistent: while Czernecki et al. (2021) reported significant impairments in PD compared with HC, other studies did not observe such differences (Martins et al., 2024; Yu et al., 2012).

#### 3.2.2 Affective ToM

While 28 reported studies indicate significant impairments in affective ToM in patients with PD compared with HC, 21 studies reported non-significant findings. The most frequently used task to assess affective ToM is the “Reading the Mind in the Eyes” test [RMET (Baron-Cohen et al., 1997)], a set of black-and-white photographs of the eye region in which participants are asked to infer the depicted mental state by choosing the correct answer from four alternatives. Among the reviewed studies comparing PD with HC, twelve studies using the RMET reported no significant differences (Alonso-Recio et al., 2021; Euteneuer et al., 2009; Fernández-Fernández et al., 2024; McKinlay et al., 2013; Péron et al., 2009, 2010b; Roca et al., 2010; Rosen et al., 2013, 2015; Rossetto et al., 2018; Santangelo et al., 2020; Usnich et al., 2023), while fourteen studies found that patients with PD perform worse than HC (Bodden et al., 2010b; Enrici et al., 2015, 2017; Esteves et al., 2018; Foley et al., 2019; Menozzi et al., 2025; Mimura et al., 2006; Orso et al., 2020; Poletti et al., 2013; Raffo De Ferrari et al., 2015; Silveri et al., 2024; Tsuruya et al., 2011; Usnich et al., 2023; Xi et al., 2015).

Importantly, the rather inconclusive findings related to RMET performance in PD may be at least partially explained by potential confounders, among which overall cognitive functioning seems to be the most influential factor. Although Rossetto et al. (2018) reported no effect, most studies indicated that patients with PD and MCI (Adenzato et al., 2019; Dodich et al., 2022; Fernández-Fernández et al., 2024) or amnestic MCI (Maggi et al., 2024) show poorer performance relative to HC, whereas no differences were reported between PD-MCI and PD without MCI, or between PD without MCI and HC (Fernández-Fernández et al., 2024). Likewise, patients with PD-MCI exhibited greater affective ToM impairments than those without cognitive deficits (as indexed by MoCA mean scores of 24.26 and 27.50, respectively) or HC (Alonso-Recio et al., 2021). Importantly, however, McKinlay et al. (2013) demonstrated that overall cognitive functioning accounted more strongly for affective ToM deficiencies than PD diagnosis itself.

Beyond overall cognitive functioning, freezing of gait (Raffo De Ferrari et al., 2015) and the severity of PD motor symptoms also appear to modulate performance on affective ToM tasks (McIntosh et al., 2014; Poletti et al., 2013; Romosan et al., 2019; Trompeta et al., 2023; Yu et al., 2018); for different findings – see (Péron et al., 2009). These findings suggest that advanced – but not early-stage – PD is associated with affective ToM impairments. Finally, some evidence suggests that disease onset may constitute an additional moderator (Seubert-Ravelo et al., 2021; Yu et al., 2018). However, findings remain inconsistent (Seubert-Ravelo et al., 2021; Yu et al., 2018), thus highlighting the need for further investigation.

Studies using the affective component of the Faux-Pas task (Péron et al., 2009; Roca et al., 2010) found no differences between patients with PD and HC. However, studies using the Emotions Attribution Test reported mixed results: while (Santangelo et al., 2012, 2020) reported poorer affective ToM in patients with PD, others (Ciccarelli et al., 2022; Schwartz et al., 2018) found no significant group differences. A possible explanation for this discrepancy may relate to sample characteristics, as the PD and HC groups were matched on MoCA scores (Ciccarelli et al., 2022), thereby effectively controlling for potential differences in cognitive functioning, which may contribute more to group differences than PD-specific characteristics (McKinlay et al., 2013).

The Yoni task (requiring participants to infer a character’s mental states based on verbal cues, facial expressions, and gaze direction) is another tool used in this domain. Consistent with the findings from other ToM tasks summarized in this section, findings on the Yoni task remain inconsistent. Only one out of four studies reported significant affective ToM impairments in PD compared to HC (Bodden et al., 2010b), while others did not (Di Tella et al., 2025; Orduz-Bastidas et al., 2020; Rossetto et al., 2018). However, when asked to judge whether a person was in pain while observing only their limbs (using the Pain Empathy Task), patients with PD performed worse than HC (Han et al., 2025; Hu et al., 2021). By contrast, compared with patients with Huntington’s disease, those with PD were reported to exhibit preserved affective ToM skills (Di Tella et al., 2025). In the absence of other studies specifically designed to investigate whether affective ToM skills in PD differ from or are comparable to those observed in other neurological or neurodegenerative diseases, the findings remain inconclusive. Overall, to better capture the specific nature of affective ToM impairments in patients with PD – and to address the substantial methodological differences across studies that hinder direct comparisons – future research should consider assessing affective ToM by using a composite factor derived from multiple tasks.

#### 3.2.3 Cognitive ToM

The heterogeneity in the measurement of cognitive ToM is substantial, likely reflecting the absence of a well-established and widely used task for assessing cognitive ToM skills. While 24 reports supported cognitive ToM impairments in patients with PD, 15 reports found no effect. A closer look at these findings suggests that impairments in PD were primarily reported by studies employing complex ToM tasks that require substantial cognitive resources and the management of false beliefs – that is, situations in which a person holds an incorrect belief about reality, requiring the ability to recognize such mistaken beliefs in others. Deficits were consistently observed when task demands exceeded basic mental-state attribution, contrasting with preserved performance on simpler tasks. The most consistent deficits in PD were reported in tasks that rely heavily on the integrity of executive functions, and specifically require the inhibition and manipulation of misleading information [the latter of which is a hallmark of advanced cognitive mentalizing and has also been reported to be a key cognitive deficit associated with PD (Kalbe et al., 2016)].

Specifically, most studies reported that, compared to HC, patients with PD exhibited significant difficulties in recognizing and reasoning about False Beliefs (Díez-Cirarda et al., 2015; Eddy et al., 2013; Mengelberg and Siegert, 2003; Saltzman et al., 2000), for different findings, see (Rossetto et al., 2018), as well as in identifying complex social blunders (Faux Pas). Faux Pas deficits in patients with PD have been widely replicated across studies, indicating difficulties in integrating intentionality with social impact (Costa et al., 2013; Del Prete et al., 2020; Esteves et al., 2018; Péron et al., 2009; Silveri et al., 2024; Usnich et al., 2023), with only two studies reporting no effect (Eddy et al., 2013; Roca et al., 2010).

Moreover, patients with PD exhibit performance deficits when required to interpret subtle, non-literal communication or to resolve complex social narratives. These difficulties include understanding irony, deception, and sarcasm (Monetta et al., 2009; Santangelo et al., 2012, 2020), as well as attributing complex communicative intentions, such as recognizing others’ attempts to outsmart or warn someone (Enrici et al., 2017; Pell et al., 2014). Finally, findings regarding irony recognition are somewhat inconsistent. While some studies reported no group differences (Bauer et al., 2023; Benke et al., 1998). Benke et al. (1998) found that ToM performance was modulated by memory function: patients with PD and deficient verbal memory performed worse than HC and patients with PD without verbal memory dysfunction.

Crucially, consistent with the above-mentioned findings on affective ToM, impairments in cognitive ToM were linked to the severity of PD motor symptoms and disease onset. While patients with de novo and early-stage PD did not perform significantly differently from HC (Péron et al., 2009; Trompeta et al., 2023), advanced PD was associated with greater deficits compared with HC (Péron et al., 2009). Moreover, again compared with HC, patients with early onset PD were reported to experience greater cognitive ToM deficits in understanding social causal relations (Seubert-Ravelo et al., 2021). Moreover, co-occurring cognitive impairment – such as PD-MCI, PD-aMCI (PD-amnestic MCI) or naMCI (non-amnestic MCI) manifesting as executive dysfunction (Adenzato et al., 2019; Costa et al., 2013; Dodich et al., 2022; Fernández-Fernández et al., 2024; Maggi et al., 2024) – was found to hamper cognitive ToM performance, particularly in comparison with HC. This pattern underscores the secondary nature of the ToM deficit by reflecting that overall cognitive functioning as well as executive functions are strong modulators of performance on more complex ToM tasks, both affective and cognitive.

When examining trends in non-significant findings, patients with PD generally performed comparably to HC on tasks assessing basic mentalizing capacity or tasks that can be solved using simple, non-executive heuristics. Thus, it is not surprising that tasks requiring only straightforward knowledge attribution or basic first-order mental state attribution (defined as understanding and tracking another person’s mental state without reasoning about what that person thinks regarding someone else’s mental state) did not typically differentiate PD from HC (Rossetto et al., 2018; Saltzman et al., 2000; Yu et al., 2012). Occasionally, more complex tasks, such as the Strange Stories and the Yoni tasks, yield null results as well, which may be explained by sample size or population-specific factors (Del Prete et al., 2020; Orduz-Bastidas et al., 2020; Rossetto et al., 2018).

#### 3.2.4 Imaging studies on ToM

Only six imaging studies examined empathy/ToM in samples of patients with PD. However, despite their conceptual ingenuity, these studies present substantial limitations that warrant consideration. First, two studies examined neural networks underlying ToM and empathy in patients with PD and compared them with HC (Pläschke et al., 2017; Rabini et al., 2024). Rather than using behavioral ToM or empathy tasks, these studies relied on predefined neural networks to infer whether patients with PD differed from HC. Although these studies provide important conceptual groundwork, they also show clear methodological limitations. Likewise, Rabini et al. (2024) examined connectivity patterns within three predefined networks that have not been empirically validated in PD populations:

(1) Cognitive ToM (cToM): dorsomedial prefrontal cortex (dmPFC), dorsolateral prefrontal cortex (dlPFC), dorsal anterior cingulate cortex (dACC), dorsal anterior temporal lobe (dATL), caudate, putamen.

(2) Affective ToM (aToM): orbitofrontal cortex (OFC), ventromedial prefrontal cortex (vmPFC), ventral anterior cingulate cortex (vACC), ventral anterior temporal lobe (vATL), lateral inferior frontal cortex (lIFC), amygdala, nucleus accumbens.

(3) Core ToM (regions that support both affective and cognitive networks): inferior parietal lobule (IPL; BA/Brodmann Area 39/40), posterior superior temporal sulcus (pSTS), precuneus.

They reported a pattern in which core ToM connectivity remained relatively preserved, whereas domain-specific circuits – especially the cToM network – showed reduced connectivity in early-stage PD, including reduced cross-connectivity between cognitive and affective systems. While these findings suggest that ToM-related circuitry may be sensitive to PD-related neurodegeneration, the absence of behavioral ToM or empathy measures makes it difficult to confirm that these networks indeed support the cognitive functions attributed to them. This gap limits construct validity: although the networks may be theoretically plausible, their functional–behavioral grounding remains untested in PD. Similarly, Pläschke et al. (2017) used resting-state fMRI to classify patients with PD versus HC based on connectivity within two predefined networks:

(1) ToM network: the bilateral ventromedial prefrontal cortex (vmPFC), frontal pole (FP), dorsomedial prefrontal cortex (dmPFC), precuneus, temporoparietal junction (TPJ), temporal pole (TP), middle temporal gyrus (MTG), posterior superior temporal sulcus (pSTS), inferior frontal gyrus (IFG), and the right middle temporal visual area (MT/V5)

(2) Empathy network: the bilateral dorsomedial prefrontal cortex (dmPFC), supplementary motor area (SMA), rostral anterior cingulate cortex (rACC), anterior mid-cingulate cortex (aMCC), posterior cingulate cortex (PCC), anterior insula (AI), inferior frontal gyrus (IFG), midbrain, temporoparietal junction (TPJ), left anterior thalamus, amygdala, middle temporal gyrus (MTG), posterior superior temporal sulcus (pSTS), posterior thalamus, hippocampus, and the right pallidum.

They found that the ToM network could differentiate PD from HC, whereas the empathy network did not. While this suggests that the ToM-related circuitry seems to be susceptible to disease-related neurofunctional alterations, the study did not examine which connections were altered or whether these networks correspond to actual deficits in ToM or empathy performance. Taken together, these network-based approaches demonstrate that ToM-related circuits may be altered in PD, but inconsistent definitions of network boundaries and the absence of behavioral validation in patients diagnosed with PD reduce the reliability and interpretability of these findings.

A second group of studies has examined the association between ToM performance and neural measures in PD using PET (Positron Emission Tomography). Though these studies are valuable because they include explicit behavioral assessments, they also share key limitations – most notably, the lack of HC groups. Péron et al. (2010b) assessed PD patients before and after DBS surgery using an adapted RMET task. Improvements in ToM were positively correlated with increased glucose metabolism in widespread regions, including the cingulate gyrus, bilateral middle frontal gyrus, fusiform gyrus, and parietal and occipital cortices. Decrements in ToM skills were associated with increased metabolism in left STG and bilateral IFG. These effects indicate broad metabolic correlates but do not specify a coherent or selectively ToM-related network that corresponds to the network-based analyses mentioned above (Pläschke et al., 2017; Rabini et al., 2024). Orso et al. (2020) also examined RMET performance and PET metabolic measures (as well as transporter binding) with respect to the level of cerebral neurodegeneration. These findings showed that, in the less affected hemisphere, higher ToM scores were associated with higher metabolism in the superior temporal gyrus, whereas in the more affected hemisphere, higher ToM scores were related to higher metabolism in the insula. Furthermore, better RMET scores were associated with lower serotonin transporter (SERT) availability in the thalamus, whereas no such associations were found for dopamine transporter binding. Finally, Trompeta et al. (2023) used PET in combination with a ToM Picture Stories Task and reported that greater dopamine availability in the right thalamus and left putamen was associated with better ToM performance. Although this finding links dopaminergic function to ToM, it does not correspond to the metabolic regions reported in the two studies mentioned above. Across these PET studies, there is minimal convergence in the specific regions implicated. Some variability can plausibly be attributed to methodological differences, including the specific ToM task used (e.g., RMET vs. picture stories), PET tracer type, disease stage, and the presence or absence of DBS. As a result, findings regarding the brain regions found to modulate ToM processing are highly inconsistent.

Only one study to date has examined structural brain correlates of ToM in PD while including a matched HC group (Díez-Cirarda et al., 2015). Using MRI and the Strange Stories Task, the authors found no significant associations between ToM and gray matter in either PD or controls. For white matter, they reported that higher mean diffusivity in the left superior longitudinal fasciculus was associated with higher ToM scores (indicating better ToM performance) in PD patients. This association remained significant after controlling for executive functions and was not observed in HC. However, because elevated mean diffusivity typically reflects reduced white matter integrity, the observed positive association with better ToM performance is counterintuitive. This finding may reflect compensatory mechanisms, measurement issues, or task-specific effects, and hence, it highlights the need for replication.

In summary, the neuroimaging literature linking PD to empathy/ToM remains sparse and methodologically heterogeneous. Although PET studies provide direct ToM–brain correlations, they lack control groups and show highly inconsistent neural substrates. Likewise, network-based fMRI studies demonstrate theoretical relevance but rely on predefined network definitions without behavioral verification. Finally, structural MRI evidence is limited to a single study with mixed and partly counterintuitive results. Overall, although the existing work is innovative and underscores the relevance of neurocognitive dysfunctions in patients with PD, it also raises concerns regarding validity and reliability (as reflected by the absence of behavioral ToM measures in some studies, cross-study inconsistency in the neural regions implicated, and limited ecological validity). These challenges highlight the need for multimodal, task-validated, and methodologically rigorous approaches to clarify how PD affects the neural architecture of ToM and empathy.

### 3.3 Social problem-solving

Across all assessment levels – reported, behavioral, and imaging – social problem-solving is the least studied aspect of SC in PD, likely due to its complexity and reliance on both domain-general (e.g., executive functions) and domain-specific processes (e.g., language). Report-based tools assessing social sensitivity, the ability to adjust behavior according to context, and self-management of social appearance generally fail to capture the full extent of social problem-solving deficits in PD, as no differences were observed between patients with PD without MCI, PD-MCI and HC (Fernández-Fernández et al., 2024; Trompeta et al., 2023). Similarly, using a self-reported problem-solving styles questionnaire, Anderson et al. (2013) found no significant differences among these three groups. A plausible explanation for these null results is that report-based measures may underestimate difficulties in real-world social interactions in patients with PD.

Behavioral studies reveal a clearer pattern: In contrast to social interpretation, which appears to be relatively preserved in PD (Caballero et al., 2022; McNamara et al., 2010), clearer impairments are observed when patients with PD are required to translate social information into decisions and behavior. These impairments become more pronounced with increasing disease severity and cognitive decline. Using a moral decision-making task, Rosen et al. (2013) reported no differences between patients with PD and HC. However, in a later study using the same task, Rosen et al. (2015) found that patients with PD made more selfish decisions, particularly in situations involving high emotional conflict – namely, when another person was present in the social scenario. This discrepancy may reflect differences in sample characteristics, as the later study sample was older, had a longer disease duration, and exhibited more severe motor symptoms. Additional evidence for impaired social problem-solving comes from tasks involving economic choices as well as from evaluations of the quality of proposed solutions to social scenarios in terms of their viability, practicality, and social appropriateness. Specifically, compared to HC, patients with PD allocated more money to counterparts who used polite language or expressed confidence, indicating more biased social behavior (Caballero et al., 2022). Similarly, deficits were observed in problem-solving, as patients with early-onset PD proposed less viable and safe solutions than HC (Seubert-Ravelo et al., 2021). Consistent with the findings related to the SC aspects discussed above (i.e., emotion recognition and empathy/ToM), cognitive impairment was found to hamper social problem-solving skills (Anderson et al., 2013). In particular, Anderson et al. (2013) showed that patients with PD-MCI generated fewer solutions that were both socially sensitive and practical than patients with PD without MCI. At the same time, when HC were compared with patients with PD, with or without MCI, HC generated the highest proportion of solutions that were both socially sensitive and practical. However, somewhat surprisingly, when focusing on the quality of these solutions, no differences were observed among the three groups. Nevertheless, the behavioral literature remains sparse and heterogeneous in terms of sample characteristics, highlighting the need for replication studies.

At the neuroimaging level, only one study to date has investigated the neural correlates of social problem-solving in PD. Using questionnaires and PET imaging, Trompeta et al. (2023) found that patients with PD who had higher self-monitoring in social contexts also had greater dopaminergic activity in the left pallidum. This preliminary finding suggests that intact dopaminergic circuits may support adaptive social behavior, thus highlighting the potential value of neuroimaging to elucidate the neural basis of social problem-solving deficits in PD. Clearly, future neuroimaging studies are needed to further elucidate the neurofunctional and neurostructural correlates of social problem-solving. Upon considering the complex nature of social problem-solving the latter endeavor is especially challenging [i.e., calling for behavioral tasks and imaging methods that are suitable (i) to disentangle the tight interplay between domain-general and domain-specific functions supporting social problem-solving, and (ii) to identify the neural mechanisms implicated in social problem-solving while controlling for disease-related influences].

## 4 Discussion

The main aim of this section is to summarize and discuss the empirical evidence examining SC in patients with PD. Given the complex and componential nature of SC, it is not surprising that the literature investigating its neurocognitive correlates is somewhat inconsistent. Although the research interest in SC is relatively recent, stimulated by the recognition of SC as the sixth cognitive core symptom in the DSM-V, alongside attention, language, memory, executive function, and perceptual/motor skills (American Psychiatric Association, 2013) – our systematic review identified a substantial number of studies investigating SC in PD. However, closer inspection of the published literature reveals that most studies have focused on emotion recognition, whereas more complex aspects of SC – such as empathy/ToM and social problem-solving – have largely been neglected in PD research. The present systematic review is novel in being more comprehensive than recent reviews and meta-analyses [e.g., (Coundouris et al., 2020; Hazelton et al., 2025; Ibarretxe-Bilbao et al., 2011; Moonen et al., 2017)] by explicitly acknowledging the componential nature of SC. Moreover, to our knowledge, this is the first systematic review to include neuroimaging studies on SC in PD, with the aim of identifying, and possibly disentangling, neurofunctional networks underlying the predefined three key aspects of SC: emotion recognition, empathy/ToM, and social problem-solving.

**FIGURE 2:**
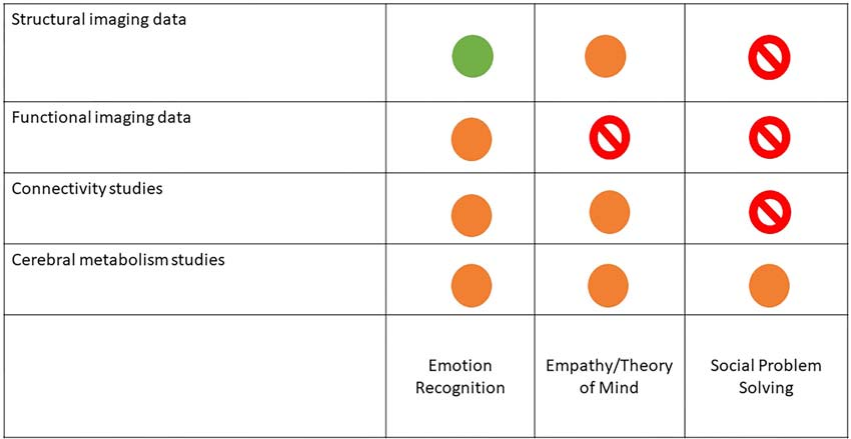
Evidence matrix for the correlates between imaging data and key aspects of social cognition (SC). The Figure depicts the existence and level of (in-)consistency of research in the field of SC, differentiated along the three key aspects of SC and the related imaging studies (as reported in the present systematic review). Red circles with dash = no empirical brain imaging studies; green circle quite = many empirical brain imaging studies with moderate to high consistency; orange circles = few empirical brain imaging studies and low consistency across findings.

**FIGURE 3:**
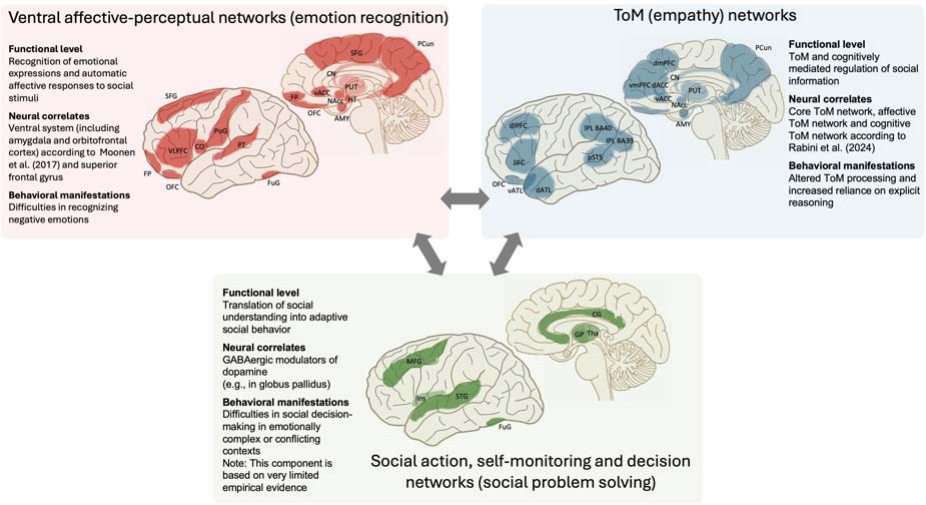
Integrated model of social cognition (SC) networks in Parkinson’s disease (PD). The Figure depicts three conceptually distinct networks of SC (that underlie the three key aspects of SC which are the focus of the present systematic review), namely the (i) ventral affective–perceptual networks (modulating emotion recognition), (ii) ToM networks (modulating empathy/ToM), and (iii) social action and self-monitoring networks (modulating social problem-solving). Each of these networks is supported by partially overlapping neural correlates and moreover, is characterized by unique as well as partly shared behavioral manifestations. AMY, amygdala; CG, cingulate gyrus; CN, caudate nucleus; CO, central operculum; dACC, dorsal anterior cingulate cortex; dATL, dorsal anterior temporal lobe; dlPFC, dorsolateral prefrontal cortex; dmPFC, dorsomedial prefrontal cortex; FuG, fusiform gyrus; FP, frontal pole; GP, globus pallidus; Ins, insula; HT, hypothalamus; lIFC, lateral inferior frontal cortex; IPL BA 39, inferior parietal lobe, Brodmann Area 39; IPL BA 40, inferior parietal lobe, Brodmann Area 40; MFG, middle frontal gyrus; NAcc, nucleus accumbens; OFC, orbitofrontal cortex; PCun, precuneus; PoG, postcentral gyrus; pSTS, posterior superior temporal sulcus; PT, planum temporale; PUT, putamen; SFG, superior frontal gyrus; STG, superior temporal gyrus; Tha, thalamus; vACC, ventral anterior cingulate cortex; vATL, ventral anterior temporal lobe; vlPFC, ventrolateral prefrontal cortex; vmPFC, ventromedial prefrontal cortex; VS, ventral striatum.

To better organize the complex and sometimes inconsistent findings reported in the Results section, we *first* provide a schematic figure summarizing the reviewed literature on SC in PD, differentiating findings according to their level of (in-)consistency across studies (Figure 2). *Second*, we propose a multilayer model depicting the neurostructural and neurofunctional correlates of SC in PD, explicitly acknowledging its componential nature (Figure 3). *Finally*, we discuss potential reasons for the inconclusive findings across studies and outline directions for future research in SC in PD.

### 4.1 A multi-componential model of neurostructural and neurofunctional constructs that underlie SC in PD

To summarize the findings of the reported studies, we propose a tentative framework for understanding alterations of SC in PD by sketching a multi-componential model that incorporates structural and functional imaging data for the three predefined key aspects of SC. Beyond integrating evidence from the behavioral and imaging levels, the tentative model is also based on theoretical models of emotional and social processing, as presented throughout the current systematic review. Rather than assuming a strict temporal sequence, we offer a model that conceptualizes SC as a multi-componential construct supported by partially distinct yet highly intertwined constructs. Each component may be differentially affected by PD-characteristic trajectories – primarily cognitive decline, as well as the onset and severity of motor symptoms – and by potential compensatory mechanisms (see general outline in Figure 3). Importantly, this model should not be interpreted as evidence for a stable or fully convergent neuroimaging signature of SC impairment in PD. Rather, it represents a hypothesis-generating synthesis of currently available findings.

#### 4.1.1 Ventral affective–perceptual networks (emotion recognition)

One central component of SC may rely on ventral neural systems supporting bottom-up perception of socially salient cues and automatic affective responses. Impairments in emotion recognition in PD are often associated with structural alterations in ventral brain regions, particularly the amygdala and orbitofrontal cortex (Baggio et al., 2012; Funghi et al., 2025; Ibarretxe-Bilbao et al., 2011; Moonen et al., 2017; Tessitore et al., 2002), although findings are not entirely consistent across studies. Consistent with Moonen et al. (2017), theoretical accounts further distinguish a ventral pathway involved in emotion perception and automatic affective processing, which is considered especially vulnerable to dopaminergic dysfunction in PD. Behaviorally, ventral system involvement is reflected in frequently reported difficulties in recognizing negative emotions [e.g., (Assogna et al., 2010; Baggio et al., 2012; Czernecki et al., 2021; Sedda et al., 2017)]. These impairments are not uniform across patients, suggesting variability in the extent and functional consequences of ventral system dysfunction, which is most plausibly explained by inter-individual differences in overall cognitive abilities and PD-specific neurodegenerative processes.

#### 4.1.2 Empathy/ToM networks

Social cognition also depends on distributed cortical and subcortical networks supporting perspective-taking and mental state attribution. Although brain imaging evidence to date is limited and sometimes inconsistent, network-based approaches provide the most coherent explanation for the ToM difficulties reported in PD (Pläschke et al., 2017; Rabini et al., 2024). Specifically, structural connectivity within domain-specific ToM networks – particularly the cognitive ToM network – is often reduced, frequently impairing cross-connectivity between cognitive and affective ToM networks (Rabini et al., 2024). At the behavioral level, degeneration of these networks may be associated with poorer performance on both affective and cognitive ToM tasks, especially when task demands are high.

#### 4.1.3 Social action, self-monitoring and decision networks (social problem-solving)

The final component of SC concerns the translation of social understanding into adaptive and adjusted behavior in real-world contexts; however, evidence in PD remains sparse. Findings linking dopaminergic modulation in the left pallidum to social problem-solving suggest a potential role of dopaminergic modulation in basal ganglia–thalamo–cortical loops, particularly via GABAergic neurons in the globus pallidus (Haber, 2014). These preliminary findings raise the possibility that PD-related dopaminergic dysfunction may contribute to difficulties in making socially appropriate decisions. Importantly, this does not necessarily reflect a loss of social knowledge but can be seen as an expression of a suboptimal or disrupted coordination of social understanding, self-monitoring, and action selection.

### 4.2 Future research and limitations of the current study

It is important to emphasize that the tentative framework outlined in section “4.1 A multi-componential model of neurostructural and neurofunctional constructs that underlie SC in PD” should be regarded as explanatory and preliminary in nature. This caution is warranted upon given that the literature collated in this systematic review is largely inconclusive rather than consistent, primarily due to the considerable methodological heterogeneity across studies. In other words, the reported studies are not directly comparable, because they differ widely in terms of conceptual background (i.e., definition and operationalization of SC), sample characteristics (PD with and without MCI, severity of motor symptoms, disease duration, healthy or clinical comparison group etc.), behavioral tasks used to assess SC (no consensus currently exists regarding which task best captures a specific aspect of SC), and brain imaging methods employed to identify the neural correlates of SC in PD (structural vs. functional imaging, connectivity analyses, etc.). A further limitation concerns the risk-of-bias appraisal process. Although the appraisal criteria were predefined and based on established JBI guidance, the full appraisal was not conducted independently by two reviewers for all included studies. Therefore, some degree of subjectivity in item-level judgments cannot be ruled out. To minimize this risk, decision rules were specified in advance.

Another potential limitation is the considerable imbalance regarding the number of included studies related to the three SC aspects under investigation. Most brain imaging studies investigating SC have focused on emotion recognition, considered a relatively simple aspect of SC, while relatively few studies aimed to assess the neurocognitive correlates of ToM or social problem-solving, which reflect more complex aspects of SC. This imbalance can be plausibly explained by the complex nature of ToM and even more so, of social problem-solving. Importantly, ToM and social problem-solving alike draw heavily on domain-general (i.e., attention, executive function) as well as domain-specific (e.g., language) functions, that are tightly intertwined and thus, difficult to disentangle at behavioral and neural levels. However, despite acknowledging the conceptual and clinical relevance of the topic, a detailed discussion of the complex interplay between ToM/social problem-solving and other cognitive domains – including executive and language functions – goes far beyond the scope of this review. Clearly, future research should aim to clarify the componential nature of SC by investigating how different aspects are interrelated at the behavioral level, including the identification of unique and shared processing mechanisms across simple and complex SC components. Moreover, future research is needed to identify the neurofunctional and neurostructural mechanisms underlying both simple and complex SC tasks and how these mechanisms are affected in PD. Clinically, careful patient selection and inclusion of HC are essential to differentiate PD-specific neurodegenerative changes from age-related neurocognitive alterations. Moreover, we would like to emphasize that beyond dopamine, several other neurotransmitters, such as glutamate (Cattaneo et al., 2025), play a significant role in the manifestation of non-motor symptoms of PD. Thus, future research targeted at SC in PD should go beyond the examination of dopamine-related hypotheses. Finally, though being relevant, potential effects of sex and ethnicity have not been considered in the present systematic review, as this goes beyond the scope of the present review article. To summarize, this systematic review highlights the urgent need for conceptually rigorous, multimodal, and behaviorally grounded future research approaches to studying SC in PD.

## Data Availability

The original contributions presented in this study are included in the article/Supplementary material, further inquiries can be directed to the corresponding authors.
