# Supplementary material for: Social cognition in Parkinson’s disease: a comprehensive systematic review and integrative conceptual framework
Source: Front Aging Neurosci. 2026 Jun 29;18:1863728. doi: 10.3389/fnagi.2026.1863728 (PMC13357429; doi:10.3389/fnagi.2026.1863728)
Supplement: Supplementary file 1 [file Data_Sheet_1.pdf]

`I Knew Nothing About Parkinson's': Insights into Receiving a Diagnosis of Parkinson's Disease and the Impact of Self-Management, Self-Care, and Exercise Engagement, from People with Parkinson's and Family Members' Perspectives: Qualitative Study

**Item Type**	Journal Article
**Author**	Leanne Ahern
**Author**	Catriona Curtin
**Author**	Suzanne Timmons
**Author**	Sarah E. Lamb
**Author**	Ruth McCullagh
**Date**	2025-05-25
**Volume**	10
**Publication**	GERIATRICS
**DOI**	10.3390/geriatrics10030073
**Issue**	3
**Date Added**	14.7.2025, 14:48:38
**Modified**	5.9.2025, 14:25:07

Notes:

Not Included: Qualitative Study

Tags: EXCLUDED

A 20-Year Systematic Review of the 'Reading the Mind in the Eyes' Test across Neurodegenerative Conditions.

**Item Type**	Journal Article
**Author**	Owen Stafford
**Author**	Christina Gleeson
**Author**	Ciara Egan
**Author**	Conall Tunney
**Author**	Brendan Rooney
**Author**	Fiadhnait O'Keeffe
**Author**	Garret McDermott
**Author**	Simon Baron-Cohen
**Author**	Tom Burke
**Abstract**	Social cognition has a broad theoretical definition, which includes the ability to mentalise, i.e., recognise and infer mental states to explain and predict another's behaviour. There is growing recognition of the clinical, diagnostic, and prognostic value of assessing a person's ability to perform social cognitive tasks, particularly aspects of theory of mind, such as mentalising. One such measure of mentalising is

the 'Reading the Mind in the Eyes' test (RMET). This systematic review and meta-analysis consider performance on the RMET, applied to people with neurodegenerative conditions in matched control studies, since its publication in 2001. Overall, this review includes 22 papers with data from N = 800 participants with neurodegenerative conditions: Alzheimer's disease, n = 31; Parkinson's disease, n = 221; Lewy body dementia, n = 33; motor neuron disease, n = 218; Huntington's disease n = 80; multiple sclerosis, n = 217; and N = 601 matched typical controls. Our meta-analyses show that deficits in mentalising, as measured by the RMET, are consistently reported across neurodegenerative conditions, with participants in both early and late disease stages being affected. Social cognition is an emerging field of cognitive neuroscience requiring specific and sensitive measurement across each subdomain. Adult-based meta-normative data feature, for which future groups or individuals could be compared against, and hypotheses relating to the source of these mentalising deficits are further discussed. This review was registered with PROSPERO (CRD42020182874).

Date 2023 Aug 31
Language eng
Extra Place: Switzerland
Volume 13
Publication Brain sciences
DOI 10.3390/brainsci13091268
Issue 9
Journal Abbr Brain Sci
ISSN 2076-3425
PMID 37759869
PMCID PMC10526136
Date Added 6.7.2025, 19:09:41
Modified 5.9.2025, 14:57:29

Notes:

Included: Systematic Review & Meta-Analysis

Inclusion Criteria

A systematic literature search of the PubMed, Medline, and PsycINFO databases was carried out in the period extending from February to December 2020. This search strategy included iterative processes, using a combination of keywords, index terms, Boolean Operators, and search strings. Search terms included 'theory of mind' OR 'mentalising' OR 'social cognition', in combination with the required subfield of 'Reading the Mind in the Eyes

Only papers published in English were included for full review. Potentially included studies had their methods section reviewed to ensure that the English-version of the tool was used. Studies that reported objective quantitative investigations using the full 36-item version of the RMET were included. In the initial searches, all clinical groups with matched typical controls (TCs) were required, with neurodegenerative conditions extracted specifically for the primary purpose of this review, and TC data were used as meta-normative data.

Risk of Bias

The risk of bias of the included studies was assessed independently by the two researchers (OS and TB), who assessed the suitability of the full-text articles. These studies were assessed for methodological quality, using a published checklist by Hawker, Payne, Kerr, Hardey, and Powell that identifies nine specific elements of the published report, each evaluated using a 4-point Likert-type scale (4 = good, 3 = fair, 2 = poor, 1 = very poor). The 9 elements included: (1) the abstract and title, (2) introduction and aims, (3) method and data, (4) sampling procedure, (5) data analysis, (6) ethical consideration and approvals, (7) findings and results, (8) transferability/generalizability, and (9) implications and usefulness. Scores for each article were summed with higher scores reflecting higher methodological quality (scores range from 9 (very poor) to 36 (very good).

The Grading of Recommendations Assessment Development and Evaluation (GRADE) approach was further used to assess the overall quality of evidence for each variable considered within this study. Two researchers (OS and TB) rated the factors on the GRADE criteria. The criteria 'dose effect', 'inconsistency', and 'moderate/large effect' were omitted, as these criteria were not applicable to the quality of evidence for the present study.

Results

The search identified a total of 53 potentially relevant articles following title and abstract screening. After the removal of duplicates and full-text screening, a total of 22 studies were included. This yielded a total of n = 800 participants and n = 601 matched TCs. Within the cohort of people with a neurodegenerative condition, the breakdown is as follows: Alzheimer's disease (AD), n = 31; **Parkinson's disease (PD), n = 221;** Lewy body dementia (DLB), n = 33; motor neuron diseases (MNDs), n = 218; Huntington's disease (HD), n = 80; and multiple sclerosis (MS), n = 217.

Overall, the six PD studies included in the present systematic review showed strong evidence of a social cognitive impairment. However, there was also substantial variability among the selected studies, suggesting the need for better and continued research on mentalising abilities in PD populations. The first of these compared RMET performance between a group of non-demented participants with PD and those of a TC group. The findings revealed that the participants with PD scored significantly lower than the TCs. The authors also performed a correlation analysis and showed that neuropsychological test scores, disease duration, disease severity, depressive symptoms, and health-related quality of life were not significant contributors to the impaired ToM performance observed among the PD group.

Poletti and colleagues investigated the impact of PD disease severity on mentalising performance by recruiting participants at both early and moderate stages of PD, along with a group of age-matched TCs. The results showed that when performance on the RMET was analysed according to disease stage (i.e., early and moderate), TCs significantly outperformed both PD groups (both $p < 0.01$), and participants with early PD outperformed the moderate PD group ($p = 0.01$). Similar to the Bodden et al.'s study, these authors also examined the impact of other potential sources of variability in their sample. Here, the lower RMET performance shown among both PD groups combined was not significantly altered when controlling for age, education, the MMSE, BDI, Frontal Assessment Battery (FAB), or the Montreal Cognitive Assessment (MoCA). However, significant ToM differences which were shown between early- and moderate-stage PD groups were not significant when these factors were covaried.

Enrici et al. also found a significant mentalising deficit in participants with both early and moderate PD (mean time since onset, 10.56 ± 3.88 years; mean Hoehn and Yahr (H&Y) scores of 2.81 ± 0.86) when compared to the TC group on the RMET. Similar to the results of Bodden et al., deficits in RMET performance in the PD group remained when factors such as disease severity, duration of illness, and depressive symptomatology were covaried for, alongside an additional examination of dopamine therapy, cognitive status, executive function, anxiety, and apathy. A measure of facial recognition accuracy was also extracted as a control task by asking all participants to judge the gender of the face in an additional trial of target stimuli.

A follow-up paper, Reference, examined the effects of deep brain stimulation (DBS) to the subthalamic nucleus (STN) on social cognition in participants with PD. Participants with PD were divided into two groups (STN-DBS and dopaminergic replacement therapy) and compared to TCs. There was a significant difference between the PD and TC groups when compared on performance on the RMET, although no within group differences were found when PD groups were compared. Enrici et al. suggested that participants with PD undergoing dopaminergic replacement therapy or STN-DBS experience deficits in social cognitive domains, but that STN-DBS does not negatively impact social cognitive performance in isolation. As cognitive impairment is contraindicated for an individual proceeding for DBS at the outset, these findings may have large clinical impact, which is discussed below.

One study presented conflicting evidence whereby mentalising abilities were preserved in participants with early PD diagnosis when their performance on the RMET was compared to TCs ($p = 0.85$). The study analysed data from participants with PD with mild-to-moderate PD, according to the H&Y criteria. Romosan et al. reported that the MoCA total score significantly predicted mentalising performance, suggesting that impaired cognitive functioning was significantly associated with impaired performance on the RMET. Moreover, a multiple regression analysis containing three cognitive domains (specifically, attention, executive function, and visuospatial abilities) explained 64% of the variance and was significantly associated with performance on the RMET. The findings revealed a significant indirect effect of PD on ToM through cognitive status (effect estimate, −4.38; 95% CI, −6.28; −2.67), and cognitive performance appeared to mediate the relationship between PD and affective ToM through the combined effect of attention, executive function, and visuospatial abilities (total effect, −3.63; $p = 0.001$; 95% CI, −5.74; −1.51).

Similar to previous studies, Reference investigated performance on the RMET and MMSE, alongside topographical and neurochemical bases, using multi-tracer molecular neuroimaging and quantitative electroencephalography. Their cohort consisted of 30 individuals with drug-naïve de novo PD ($n = 30$) and matched TCs ($n = 60$), and they also investigated depression. In this study, there was no significant difference in the participants' demographic information (age, education, or gender), cognitive status (based on MMSE), or self-reported depression. There was, however, a statistically significant lower RMET performance observed in the PD group compared to TCs (20.7 ± 5.5 vs. 27.5 ± 3.0, respectively; $p = 0.001$). Reference further investigated the relationship between positive, negative, and neutral outcomes on the RMET. Statistically significant negative correlations between both positive and negative stimuli on the RMET, both cumulatively and individually, were observed in relation to the thalamus on the less-affected brain hemisphere, while controlling specifically for background metabolic uptake on metabolic scanning. This study further shows a direct association between RMET performance and cortical metabolic levels in the superior temporal gyrus and the insula, as well as with a higher subcortical serotoninergic tone.

Considering the meta-analysis, a large effect was observed (SMD, −0.95; 95% CI, −1.16 to −0.74; $p < 0.001$), indicating that the RMET performance was significantly lower in PD as compared with their TC groups. However, there were also high levels of heterogeneity reported in this analysis ($\chi^2 = 13.89$; $I^2 = 71\%$).

Tags: ToM, behavioral

A comparative study of social cognition in epilepsy, brain injury, and Parkinson's disease.

Item Type Journal Article
Author Julia Bauer
Author Bettina Katharina Steiger
Author Lorena Chantal Kegel
Author Marcel Eicher
Author Kristina König
Author Heide Baumann-Vogel
Author Hennric Jokeit
Abstract The brain regions involved in social cognition and the regulation of social behavior form a widely distributed cortico-subcortical network. Therefore, many neurological disorders could affect social cognition and behavior. A persistent lack of valid tests and a rigid neuropsychological focus on language, attention, executive function, and memory have contributed to a long-standing neglect of social cognition in clinical diagnostics, although the DSM-5 recognizes it as one of the six core dimensions in neurocognitive disorders. To assess for the first time the diagnostic yield of a comprehensive social cognition battery (Networks of Emotion Processing [NEmo]), we administered several emotion recognition and theory of mind tests to three incidental clinical samples with different neurological conditions: temporal lobe epilepsy (n = 30), acquired brain injury (n = 24), Parkinson's disease (n = 19), and a healthy control group (n = 67). A multivariate analysis of covariance was performed to test the effect of group on subscales of the NEmo test battery, controlling for age and performance IQ. The results showed statistically significant differences between clinical groups and healthy controls. No differences were found for gender and lateralization of the predominant lesion side. In our incidental samples, 86% of individuals with temporal lobe epilepsy, 57% of individuals with acquired brain lesion, and 14% of individuals with Parkinson's disease underperformed on tests of social cognition compared with controls. These findings suggest a differential impact of neurological disorders on the risk of impaired social cognition and highlight the need to consider social cognition in diagnostics, counselling, therapy, and rehabilitation.
Date 2023 Jun
Language eng
License © 2023 The Authors. PsyCh Journal published by Institute of Psychology, Chinese Academy of Sciences and John Wiley & Sons Australia, Ltd.
Extra Place: Australia
Volume 12
Pages 443-451
Publication PsyCh journal
DOI 10.1002/pchj.650
Issue 3
Journal Abbr Psych J
ISSN 2046-0260 2046-0252
PMID 37127428
Date Added 6.7.2025, 19:09:33
Modified 5.9.2025, 14:27:55

Notes:

Included

sample characteristics

size: 30 temporal lobe epilepsy (TLE), 24 (acquired brain injury) ABI, 19 PD and 67 HC

Parkinson's Disease type and duration: idiopathic PD, Median duration= 7 (1–24)

Medication: NA

Hoehn-Yahr: NA

UPDRS-3: NA

Gender (male): 12 males (64.2%)

averaged ages (SD, range): M= 61.5 (SD=11.9)

other neurological disease (tumor, stroke, etc.): NA

other major psychopathology: NA

origin country (or ethnicity): Switzerland

method observational

instruments used in order to quantify the variables

Social cognition aspect: emotion recognition and ToM

Name of the task: NEmo test battery; four tests of emotion recognition (ER) and three tests of ToM. All but two subtests of the NEmo test battery are newly developed tests (for more details about the stimuli material, see Bauer et al., 2019)

Static Facial ER Task

The Static Facial ER Task assesses ER of static facial expressions with direct and averted gaze direction. Participants are asked to select an emotion from a list with the six basic emotions (fear, anger, disgust, sadness, happiness and surprise) and neutral.

Dynamic Facial ER Task

The Dynamic Facial ER Task is a measure of ER in dynamic facial expressions. Participants are asked to choose the corresponding emotion out of a list of the six basic emotions as quickly as possible after each brief video presentation.

Simultaneous Facial ER Task

The Simultaneous Facial ER Task measures ER in two simultaneously presented dynamic facial expressions. Participants are asked to decide whether the two faces express the same or different emotions.

Prosodic and Facial ER Task

The Prosodic and Facial ER Task involves multimodal ER of simultaneously presented static facial and prosodic expressions. Participants are asked to decide whether the emotions in the visual and auditory modality correspond to, or differ from, each other.

Recognition of Irony Task

The Recognition of Irony Task assesses the participant's ToM ability to identify irony based on prosodic information in the context of spoken short stories. At the end of each short story, participants are asked to decide whether the last utterance was meant to be ironic, neutral, or empathetic/friendly.

Faux-Pas Test

The Faux-Pas Test (FPT; Stone et al., 1998) evaluates affective and cognitive ToM ability using written short stories illustrating a social situation with a faux-pas (i.e., an unintended socially inappropriate behavior). The FPT assesses the participant's ability to detect the faux-pas and to infer the resulting emotional and mental states of others. We used the shortened German adaption, which consists of five stories.

Movie for the Assessment of Social Cognition

The Movie for the Assessment of Social Cognition (MASC; Dziobek et al., 2006) measures the ability to refer to mental states, such as feelings, thoughts and intentions, of actors based on a 15-min movie with multiple-choice questions. The MASC consists of four ToM subscales, one accurate ToM score and three error scores in mental attribution, such as excessive, reduced and absence of ToM.

Main findings related to the review's scope

The PD were sig lower in the Simultaneous FER. compared to HC: significant lower score for PD in comparison to HC. All other tests were with non-sig differences.

No comparisons are reported between the three patients groups.

Tags: emotion recognition, ToM, behavioral

A comparison of facial emotion processing in neurological and psychiatric conditions

Item Type Journal Article
Author Benoit Bediou
Author Jerome Brunelin
Author Thierry d'Amato
Author Shirley Fecteau
Author Mohamed Saoud

Author Marie-Anne Henaff
Author Pierre Krolak-Salmon

Abstract Patients suffering from various neurological and psychiatric disorders show different levels of facial emotion recognition (FER) impairment, sometimes from the early phases of the disease. Investigating the relative severity of deficits in FER across different clinical and high-risk populations has potential implications for the diagnosis and treatment of these diseases, and could also allow us to understand the neurobiological mechanisms of emotion perception itself. To investigate the role of the dopaminergic system and of the frontotemporal network in FER, we reanalyzed and compared data from four of our previous studies investigating FER performance in patients with frontotemporal dysfunctions and/or dopaminergic system abnormalities at different stages. The performance of patients was compared to the performance obtained by a specific group of matched healthy controls using Cohen's d effect size. We thus compared emotion and gender recognition in patients with frontotemporal dementia (FTD), amnestic mild cognitive impairment (aMCI), Alzheimer's disease (AD) at the mild dementia stage, major depressive disorder, Parkinson's disease treated by l-DOPA (PD-ON) or not (PD-OFF), remitted schizophrenia (SCZ-rem), first-episode schizophrenia treated by antipsychotic medication (SCZ-ON), and drug-naive first-episode schizophrenia (SCZ-OFF), as well as in unaffected siblings of patients with schizophrenia (SIB). The analyses revealed a pattern of differential impairment of emotion (but not gender) recognition across pathological conditions. On the one hand, dopaminergic medication seems not to modify the moderate deficits observed in SCZ and PD groups (ON vs. OFF), suggesting that the deficit is independent from the dopaminergic system. On the other hand, the observed increase in effect size of the deficit among the aMCI, AD, and FTD groups (and also among the SIB and SCZ-rem groups) suggests that the deficit is dependent on neurodegeneration of the frontotemporal neural networks. Our transnosographic approach combining clinical and high-risk populations with the impact of medication provides new information on the trajectory of impaired emotion perception in neuropsychiatric conditions, and on the role of the dopaminergic system and the frontotemporal network in emotion perception.

Date 2012
Language English
Extra Place: AVENUE DU TRIBUNAL FEDERAL 34, LAUSANNE, CH-1015, SWITZERLAND Type: Article
Volume 3
Publisher FRONTIERS MEDIA SA
Publication FRONTIERS IN PSYCHOLOGY
DOI 10.3389/fpsyg.2012.00098
ISSN 1664-1078
Date Added 14.7.2025, 14:50:40
Modified 5.9.2025, 14:28:01

Notes:

Included

sample characteristics

size: PD patients ON (PD-ON, N = 12) or OFF (PD-OFF, N = 13) L-DOPA treatment, schizophrenia in remission (SCZ-rem, N=29), 80 patients experiencing their first-episode, in either the drug-naïve state (SCZ-ON, N = 40) or after they had taken medication (SCZ-OFF, N = 40), unaffected siblings of schizophrenia patients (N = 30), patients with MDD (N = 20), patients with mild AD (N = 10), patients with FTD (N = 10) and patients with aMCI (N = 10).

Specifically – the PD sample and the matched HC were retrieved from Lachenal-Chevallet et al., 2006; paper that was excluded here because it was not in English.

Each had respective age- and sex-matched control group.

Parkinson's Disease type and duration: idiopathic PD, Mduration=NA

Medication: off/on medication

Hoehn-Yahr: M=NA

UPDRS-3: M=NA

Gender (male): 10 males (90%)

averaged ages (SD, range): M=61 SD=7

other neurological disease (tumor, stroke, etc.): NA

other major psychopathology: NA

origin country (or ethnicity): France

method observational

instruments used in order to quantify the variables

Social cognition aspect: emotion recognition

Name of the task: NA

type of stimulus [face/voice etc., Ekman faces/other etc.]: photographs of two female and two male faces depicting basic emotions morphed with a neutral face in 10% steps were randomly presented (the duration varied slightly across studies, from 400 ms to 1 s).

task condition: (happiness, fear, anger, and disgust)

operationalization: accuracy

Main findings related to the review's scope

PD patients were impaired relative to controls during either the OFF phase or the ON phase

The PD group performed similar to the SCZ-OFF, SCZ-ON groups, and worse than the AD, SIB, SCZ-rem, MDD, and aMCI

Tags: Emotion recognition, behavioral

A means of measuring facial expressions and a method for predicting emotion categories in clinical disorders of affect

Item Type Journal Article

Author PJ Benson

Abstract In a radical departure from traditional approaches, it was found that only seven measures of facial expression are required to accurately distinguish between normal displays of happiness, sadness, fear, disgust, surprise and anger. Using these prototypical emotion categories it has been shown that particular facial areas in Parkinson's disease (PD) are responsible for communication of blended, rather than the intended expressions. We consider how assessment of facial expressions made in this way can be used to quantify progress of therapeutic intervention in PD. We also show that in gaining a better understanding of the structure of emotion categories, advances in understanding need not be overshadowed by methodological complexity. The simplicity of the approach presented here leads us to propose that the perceptual and behavioural aspects of affective disorders can now be tackled in a unified manner. (C) 1999 Elsevier Science B.V. All rights reserved.

Date 1999-10

Language English

Extra Place: PO BOX 211, 1000 AE AMSTERDAM, NETHERLANDS Type: Article

Volume 55

Publisher ELSEVIER SCIENCE BV

Pages 179-185

Publication JOURNAL OF AFFECTIVE DISORDERS

DOI 10.1016/S0165-0327(99)00005-1

Issue 2-3

ISSN 0165-0327

Date Added 14.7.2025, 14:50:44

Modified 5.9.2025, 14:28:27

Notes:

Not Included: not on PD

Tags: EXCLUDED

A meta-analysis of performance on emotion recognition tasks in Parkinson's disease.

**Item Type**	Journal Article
**Author**	Heather M. Gray
**Author**	Linda Tickle-Degnen
**Abstract**	Individuals with Parkinson's disease (PD) have shown deficits in the ability to recognize emotion. However, these results have been inconsistent. In addition, questions remain about whether any deficit in PD is secondary to depression and broader cognitive impairments, and the effects of stimulus modality, task type, and specific emotion remain unclear. A meta-analysis of 34 comparisons, using data from 1,295 individual participants, was conducted to (a) provide a reliable estimate of the magnitude of the purported deficit in emotion recognition and (b) examine the influence of several potential moderators of emotion recognition abilities in PD. Results show a robust link between PD and specific deficits in recognizing emotion, from both the face and the voice (overall effect size g = 0.52). The deficit extends across stimulus modalities and task types and is particularly acute with respect to negative emotions. Although this deficit does not appear to be secondary to comorbid depression or visuospatial impairments, the potential role of working memory constraints warrants further investigation. We highlight the potential implications of these findings for communication abilities in PD.
**Date**	2010 Mar
**Language**	eng
**License**	Copyright 2010 APA, all rights reserved
**Extra**	Place: United States
**Volume**	24
**Pages**	176-191
**Publication**	Neuropsychology
**DOI**	10.1037/a0018104
**Issue**	2
**Journal Abbr**	Neuropsychology
**ISSN**	1931-1559 0894-4105
**PMID**	20230112
**Date Added**	6.7.2025, 19:09:37
**Modified**	5.9.2025, 14:37:07

Notes:

Included – Meta-analysis

we conducted database searches of PsycINFO and PubMed. We began using the keyword Parkinson in conjunction with each of the following keywords: facial expression, decoding, and prosody. We examined the reference list of these articles to search for more potentially relevant studies. This resulted in 257 potentially eligible papers. The abstracts of these papers were then reviewed. After this review, 203 were excluded for a variety of reasons

N = 34

The 34 papers included in this meta-analysis contributed a total of 74 nonindependent comparisons. Forty-four comparisons com pared the performance of individuals with PD and healthy controls on tasks assessing facial emotion recognition. Of these, roughly half (24) presented participants with the Pictures of Facial Affect stimuli (Ekman & Friesen, 1976), perhaps the most commonly used standard set of facial affect. Other common stimulus sets were the Japanese and Caucasian Facial Expressions of Emotion (JACFEE) series (Matsumoto & Ekman, 1988), used in six com parisons; and a subtest of the Florida Affect Battery (FAB; Bow ers, Blonder, & Heilman, 1991), used in five comparisons. Of the 44 comparisons of facial emotion recognition, 15 involved tests of discrimination (e.g., deciding which of two photographs matches a named expression; deciding whether two posers were expressing the same emotion), 26 involved the identification or labeling of an emotion (generally forced choice rather than open ended); and the remaining three were rating tasks (i.e., rating the extent of each emotion portrayed in a given face).

A total of 1,295 individual participants contributed data to this meta-analysis (701 healthy controls, 594 individuals with PD). Control and PD participants were both, on average, 63 years old (SD 5.39 and 5.34, respectively). PD participants' mean Hoehn and Yahr score was 2.32 (SD 0.62), indicating bilateral symp toms with some balance deficit but physical independence (Hoehn & Yahr, 1967). Patients averaged 6.84 years (SD 2.92) since diagnosis. The majority (roughly 80%) of studies reported that patients were being treated with antiparkinsonian medication; however, many of these did not explicitly indicate whether patients were at their optimally mediated "on" stage during assessment.

Roughly 80% of studies reported screening out patients who had signs of dementia.

Main findings related to the review's scope

We found a robust link between PD and impaired recognition of emotion from faces and voices. Relative to matched control groups, individuals with PD showed significant deficits in the ability to recognize the emotion portrayed in facial and prosodic stimuli. The overall impairment effect size g of 0.52 corresponds to an r of 0.26.

Across studies, the level of emotion recognition deficit does not appear to be related to the level of motor disability. However, the average PD patient included in this meta-analysis exhibited mild to moderate bilateral motor disabil ity, and a different pattern may have emerged if more severely affected patients had been the focus of investigation.

Although beyond the scope of this meta-analysis, the likely cause of this deficit is pathology in neural circuits involved in emotion recognition, particularly within basal ganglia structures including the ventral striatum and STN.

Our data indicate that the deficit in emotion recognition in PD is cross modal, in that it is apparent in

the recognition of emotion from both faces and voices. The cross-modal nature of the impairment provides support for the notion that in PD, basal ganglia pathology produces a decline in emotion recognition independent of stimulus modality.

Individuals with PD were more impaired in recognizing negative emotions (anger, disgust, fear, and sadness) than relatively positive emotions (happiness, surprise).

Tags: Emotion recognition, behavioral

A meta-analytic review of age differences in theory of mind.

**Item Type**	Journal Article
**Author**	Julie D. Henry
**Author**	Louise H. Phillips
**Author**	Ted Ruffman
**Author**	Phoebe E. Bailey
**Date**	09/2013
**Language**	en
**Library Catalog**	DOI.org (Crossref)
**URL**	https://doi.apa.org/doi/10.1037/a0030677
**Accessed**	19.1.2026, 7:32:26
**Volume**	28
**Pages**	826-839
**Publication**	Psychology and Aging
**DOI**	10.1037/a0030677
**Issue**	3
**Journal Abbr**	Psychology and Aging
**ISSN**	1939-1498, 0882-7974
**Date Added**	19.1.2026, 7:32:26
**Modified**	19.1.2026, 7:32:26

A novel framework for understanding reduced awareness of dyskinesias in Parkinson's Disease.

**Item Type**	Journal Article
**Author**	Sara Palermo
**Author**	Leonardo Lopiano
**Author**	Maurizio Zibetti
**Author**	Rosalba Rosato
**Author**	Daniela Leotta
**Author**	Martina Amanzio
**Abstract**	BACKGROUND: Although dyskinesias-reduced-self-awareness (DRSA) in Parkinson's disease (PD) is related to deficit in metacognition, other factors, such as "Theory of Mind" (ToM), could operate. METHODS: Forty-one PD patients were assessed using the Global Awareness of Movement Disorders (GAM) and the

Dyskinesias Subtracted-Index (DS-I). To study whether GAM and DS-I scores could be influenced by second-type ToM or Reading the Mind in the Eyes (RME) tasks, we conducted two multiple logistic regression analyses. RESULTS: The association between the GAM, the DS-I and RME task were highly significant. The association between DS-I and Trail Making Test B-A version was also verified. CONCLUSION: DRSA was related with affective component of ToM and executive functions, thus caused by a complex interplay between specific neuropsychological and motor factors.

Date 2017 Jun
Language eng
License Copyright © 2017 Elsevier Ltd. All rights reserved.
Extra Place: England
Volume 39
Pages 58-63
Publication Parkinsonism & related disorders
DOI 10.1016/j.parkreldis.2017.03.009
Journal Abbr Parkinsonism Relat Disord
ISSN 1873-5126 1353-8020
PMID 28325582
Date Added 6.7.2025, 19:09:41
Modified 5.9.2025, 14:48:43

Notes:

Not Included: No control group. Only PD patients

But comparison with cutoff:

Although the overall neuropsychological assessment reported normal cognitive profiles, 60.98% of patients had scores below the cut-off value on the RME

Tags: EXCLUDED

A Study of Emotional Processing in Parkinson's Disease

Item Type Journal Article
Author Th. Benke
Author S. Bösch
Author B. Andree
Date 10/1998
Language en
Library Catalog DOI.org (Crossref)
URL https://linkinghub.elsevier.com/retrieve/pii/S0278262698910132
Accessed 11.8.2025, 16:50:40
License https://www.elsevier.com/tdm/userlicense/1.0/
Volume 38
Pages 36-52

Publication Brain and Cognition
DOI 10.1006/brcg.1998.1013
Issue 1
Journal Abbr Brain and Cognition
ISSN 02782626
Date Added 11.8.2025, 16:50:40
Modified 11.8.2025, 16:50:40

Notes:

Included

sample characteristics

size: 48 PD (divided into 22 PD1 [comparable to HC in verbal memory] and 26 PD2 [severely impaired on a test of verbal memory] and 18 HC

Parkinson's Disease type and duration: idiopathic PD, Mduration= 9.85 SD= 5.18

Medication: on medication

Hoehn-Yahr: M= 2.39 SD= 0.53

UPDRS-3: M= 17.89 SD= 6.90

Gender (male): 22 males (46%)

averaged ages (SD, range): M= 61.58 SD= 8.70

other neurological disease (tumor, stroke, etc.): None

other major psychopathology: None

origin country (or ethnicity): NA

method observational

instruments used in order to quantify the variables

Social cognition aspect: emotion recognition

Name of the task: Recognition of emotional prosody (JUDGE)

type of stimulus [face/voice etc., Ekman faces/other etc.]: listen to 24 tape-recorded statements with neutral affective content (e.g., Peter is watching television), which were read slowly and with a strong affective expression, 12 by a female, 12 by a male voice

task conditions: angry, surprised, sad, and cheerful

operationalization: accuracy; Correct answers were given 2 points after the first, 1 point after a single repeated presentation, and 0 points were given if a subject failed to choose the correct emotional label for the vocally expressed mood

Social cognition aspect: ToM

Name of the task: Recognition of humorous sketches (PICTURE)

type of stimulus [face/voice etc., Ekman faces/other etc.]: black and white triplets illustrations. Each of the triplets consisted of three similar outline drawings which differed with respect to a few clearly identifiable details giving only one of the pictures a strikingly humorous appearance, whereas the two remaining illustrations were affectively neutral.

operationalization: accuracy.

Main findings related to the review's scope

PD (total) were not different from HC in JUDGE or PICTURE

PD2 patients performed clearly below the two other groups on all tasks, whereas PD1 subjects performed in the range of the controls.

Tags: Emotion recognition, ToM, behavioral

A Study on the Possible Diagnosis of Parkinson's Disease on the Basis of Facial Image Analysis

Item Type Journal Article
Author Jacek Jakubowski
Author Anna Potulska-Chromik
Author Kamila Bialek
Author Monika Nojszewska
Author Anna Kostera-Pruszczyk
Abstract One of the symptoms of Parkinson's disease is the occurrence of problems with the expression of emotions on the face, called facial masking, facial bradykinesia or hypomimia. Recent medical studies show that this symptom can be used in the diagnosis of this disease. In the presented study, the authors, on the basis of their own research, try to answer the question of whether it is possible to build an automatic Parkinson's disease recognition system based on the face image. The research used image recordings in the field of visible light and infrared. The material for the study consisted of registrations in a group of patients with Parkinson's disease and a group of healthy patients. The patients were asked to express a neutral facial expression and a smile. In the detection, both geometric and holistic methods based on the use of convolutional network and image fusion were used. The obtained results were assessed quantitatively using statistical measures, including F1score, which was a value of 0.941. The results were compared with a competitive work on the same subject. A novelty of our experiments is that patients with Parkinson's disease were in the so-called ON phase, in which, due to the action of drugs, the symptoms of the disease are reduced. The results obtained seem to be useful in the process of early diagnosis of this disease, especially in times of remote medical examination.
Date 2021-11
Language English
Extra Place: ST ALBAN-ANLAGE 66, CH-4052 BASEL, SWITZERLAND Type: Article
Volume 10
Publisher MDPI
Publication ELECTRONICS
DOI 10.3390/electronics10222832
Issue 22
Date Added 14.7.2025, 14:50:30
Modified 5.9.2025, 14:40:33

Notes:

Not Included: Not on SC.
Tags: EXCLUDED

A systematic review of diffusion tensor imaging and tractography in dementia with Lewy bodies and Parkinson’s disease dementia

**Item Type**	Journal Article
**Author**	Axel A. S. Laurell
**Author**	Elijah Mak
**Author**	John T. O’Brien
**Abstract**	We reviewed studies using diffusion tensor imaging (DTI) and tractography to characterise white matter changes in Dementia with Lewy Bodies (DLB) and Parkinson’s Disease Dementia (PDD). The search included MEDLINE and EMBASE, and we used a narrative strategy to synthesise the evidence. Data was extracted from 57 studies, of which the majority were considered ‘good quality’. Subjects with DLB and PDD had widespread white matter changes compared to healthy controls and Parkinson’s disease without cognitive impairment, with a relative sparing of the hippocampus. Compared to subjects with Alzheimer’s disease (AD), DLB had greater changes in thalamic connectivity and in the nigroputaminal tract, while AD had greater changes in the parahippocampal white matter and fornix. Cognition was associated with widespread white matter changes, visual hallucinations with thalamic and cholinergic connectivity, and parkinsonism with changes in structures involved in motor control. DTI and tractography may therefore be well suited for discriminating DLB and PDD from other types of dementia, and for studying the aetiology of common symptoms.
**Date**	2025
**URL**	https://www.sciencedirect.com/science/article/pii/S0149763425000077
**Volume**	169
**Pages**	106007
**Publication**	Neuroscience & Biobehavioral Reviews
**DOI**	https://doi.org/10.1016/j.neubiorev.2025.106007
**ISSN**	0149-7634
**Date Added**	6.7.2025, 19:12:35
**Modified**	5.9.2025, 14:42:56

Notes:

Not Included: Not assessing SC
Tags: EXCLUDED

A task-specific cognitive domain decline is correlated with plasma and neuroimaging markers in patients with Parkinson's disease.

**Item Type**	Journal Article

Author Cheng-Hsuan Li
Author Ta-Fu Chen
Author Pei-Ling Peng
Author Chin-Hsien Lin

Abstract BACKGROUND: Cognitive impairment is a disabling non-motor symptom of Parkinson's disease (PD). It remains uncertain whether declines in specific cognitive domains relate to imaging or plasma biomarkers across the disease continuum. OBJECTIVE: We investigated whether neuroimaging and plasma biomarkers correlate with individual task-specific cognitive domain declines evidenced by computerized neuropsychological tests in PD patients. METHODS: A total of 107 participants, including 87 PD patients (30 with normal cognition [PD-NC], 30 with mild cognitive impairment [PD-MCI], 27 with dementia [PDD]), and 20 healthy controls, were recruited. All received the Cambridge Neuropsychological Test Automatic Battery (CANTAB) test, brain MRI, and assays of plasma biomarkers, including α-synuclein, tau, Aβ42, and Aβ40. RESULTS: PD patients had generally poorer cognitive performance than controls. Patients with PD-MCI and PDD had worse performance in visual, verbal, and working memory and executive function than those with PD-NC. After adjusting for covariates, PDD patients had global cortical thinning, especially in the temporal and parietal lobes, and higher plasma α-synuclein levels and tau:Aβ42 ratios than PD-NC group. Plasma α-synuclein level was associated with frontal lobe-mediated tasks, while the tau:Aβ42 ratio was associated with posterior cortical-mediated tasks. Facial emotion recognition tasks and visual pattern recognition associated with frontotemporal cortical thinning. The accuracy of predicting PDD using age alone (area under the curve [AUC] 0.756) increased by incorporating plasma biomarkers (AUC = 0.851, p = 0.025). CONCLUSIONS: Cognitive decline in PD patients has a task-specific correlation with neuroimaging and plasma biomarkers, which may implicate the underlying neuropathological process of PDD.

Date 2022 Dec
Language eng
License © 2022. The Author(s), under exclusive licence to Springer-Verlag GmbH Germany.
Extra Place: Germany
Volume 269
Pages 6530-6543
Publication Journal of neurology
DOI 10.1007/s00415-022-11301-w
Issue 12
Journal Abbr J Neurol
ISSN 1432-1459 0340-5354
PMID 35965282
Date Added 6.7.2025, 19:09:38
Modified 5.9.2025, 14:43:30

Notes:

Included

sample characteristics

size: 107 participants; 20 HC, 87 PD

Parkinson's Disease type and duration: 30 PD-normal cognition (NC) (Mduration=5.9 ± 3.5), 30 PD-MCI (Mduration=5.6 ± 3.3), 27 PD-dementia (PDD) (Mduration=6.7 ± 3.3).

Medication: NA

Hoehn-Yahr: NA

UPDRS-3: PD-NC M= 13.9 ± 7.9, PD-MCI M= 17.0 ± 8.4, PDD M= 23.5 ± 9.1

Gender (male): PD-NC 19 (63%), PD-MCI M= 19 (63%), PDD M= 17 (63%)

averaged ages (SD, range): PD-NC M= 65.2 ± 7.9, PD-MCI M= 70.3 ± 7.2, PDD M= 75.5 ± 8.2

other neurological disease (tumor, stroke, etc.): Excluded atypical parkinsonism syndromes, including multiple system atrophy, progressive supranuclear palsy, corticobasal syndrome, and neuroleptic agent-related parkinsonism. Moreover, excluded severe head injury, encephalitis, inflammatory disease, cerebrovascular disease, or epilepsy.

other major psychopathology: None

origin country (or ethnicity): Taiwan

method Neuroimaging

instruments used in order to quantify the variables

Social cognition aspect: emotion recognition

Name of the task: Emotion Recognition Task from the CANTAB Cambridge Cognition

type of stimulus [face/voice etc., Ekman faces/other etc.]: colored faces

task condition: happiness, sadness, fear, anger, surprise, disgust

operationalization: accuracy and RT

MRI: 1.5- or 3-Tesla scanner. sagittal 3-dimen sional gradient echo T1-weighted sequence (256 × 256 matrix; field of view = 17 cm; slice thickness = 1 mm).

cortical thickness (CTh) was calculated in the surface-based pipeline, of Freesurfer software.

Cortical parcellation was done using the Desikan–Killiany atlas provided in the Freesurfer tool

Main findings related to the review's scope

Similar RT and accuracy in the emotion recognition task between groups.

Beyond groups, the vertex-wise analysis revealed a negative correlation between the overall median reaction time in the emotion recognition task and regional CTh in areas including the left caudal middle

frontal, bilateral superior frontal, and right temporal poles indicating that more prolonged reaction times correlate with thinner CTh in these regions.

Positive correlations were found between the emotion recognition task happiness hit rate and CTh in the left superior temporal area.

ERTOMDRT = emotion recognition task overall median reaction time

ERTUHRH = emotion recognition task unbiased hit rate happiness

Task=ERTOMDRT, Correlated Region=L caudal middle frontal, **Maxa=–8.13**, **Xb=–38.3**, **Yb=13.4**, **Zb=51.6**

Task=ERTOMDRT, Correlated Region=L precentral, **Maxa=–6.56**, **Xb=–54.4**, **Yb=3.8**, **Zb=31.0**

Task=ERTOMDRT, Correlated Region=L superior frontal, **Maxa=–5.50**, **Xb=–22.3**, **Yb=19.4**, **Zb=54.4**

Task=ERTOMDRT, Correlated Region=R temporal pole, **Maxa=–6.09**, **Xb=29.9**, **Yb=11.9**, **Zb=–36.0**

Task=ERTOMDRT, Correlated Region=R superior frontal, **Maxa=–6.12**, **Xb=6.9**, **Yb=53.4**, **Zb=32.5**

Task=ERTUHRH, Correlated Region=L superior temporal, **Maxa=5.34**, **Xb=–41.8**, **Yb=9.7**, **Zb=–27.3**

Tags: Emotion recognition, Imaging

Abnormal functional connectivity density involvement in freezing of gait and its application for subtyping Parkinson's disease

Item Type Journal Article

Author Chaoyang Jin
Author Shouliang Qi
Author Lei Yang
Author Yueyang Teng
Author Chen Li
Author Yudong Yao
Author Xiuhang Ruan
Author Xinhua Wei

Abstract The pathophysiological mechanisms at work in Parkinson's disease (PD) patients with freezing of gait (FOG) remain poorly understood. Functional connectivity density (FCD) could provide an unbiased way to analyse connectivity across the brain. In this study, a total of 23 PD patients with FOG (PD FOG + patients), 26 PD patients without FOG (PD FOG- patients), and 22 healthy controls (HCs) were recruited, and their resting-state functional magnetic resonance imaging (rs-fMRI) images were collected. FCD mapping was first performed to identify differences between groups. Pearson correlation analysis was used to explore relationships between FCD values and the severity of FOG. Then, a machine learning model was employed to classify each pair of groups. PD FOG + patients showed significantly increased short-range FCD in the precuneus, cingulate gyrus, and fusiform gyrus and decreased long-range

FCD in the frontal gyrus, temporal gyrus, and cingulate gyrus. Short-range FCD values in the middle temporal gyrus and inferior temporal gyrus were positively correlated with FOG questionnaire (FOGQ) scores, and long-range FCD values in the middle frontal gyrus were negatively correlated with FOGQ scores. Using FCD in abnormal regions as input, a support vector machine (SVM) classifier can achieve classification with good performance. The mean accuracy values were 0.895 (PD FOG + vs. HC), 0.966 (PD FOG- vs. HC), and 0.897 (PD FOG + vs. PD FOG-). This study demonstrates that PD FOG + patients showed altered short- and long-range FCD in several brain regions involved in action planning and control, motion processing, emotion, cognition, and object recognition.

Date 2023-08
Language English
Extra Place: ONE NEW YORK PLAZA, SUITE 4600, NEW YORK, NY, UNITED STATES Type: Article
Volume 17
Publisher SPRINGER
Pages 375-385
Publication BRAIN IMAGING AND BEHAVIOR
DOI 10.1007/s11682-023-00765-7
Issue 4
ISSN 1931-7557
Date Added 14.7.2025, 14:50:28
Modified 5.9.2025, 14:40:41

Notes:

Not Included: not on SC

Tags: EXCLUDED

Abnormal visual activation in Parkinson's disease patients

Item Type Journal Article
Author Ellison Fernando Cardoso
Author Felipe Fregni
Author Fernanda Martins Maia
Author Luciano M. Melo
Author João R. Sato
Author Antonio Cesário Cruz
Author Edno Tales Bianchi
Author Danilo Botelho Fernandes
Author Mário Luiz Ribeiro Monteiro
Author Egberto Reis Barbosa
Author Edson Amaro

Abstract Abstract Among nonmotor symptoms observed in Parkinson's disease (PD) dysfunction in the visual system, including hallucinations, has a significant impact in their quality of life. To further explore the visual system in PD patients we designed two fMRI experiments comparing 18 healthy volunteers with 16 PD patients without visual complaints in two visual fMRI paradigms: the flickering checkerboard task and a facial perception paradigm. PD patients displayed a decreased activity in the primary visual cortex (Broadmann area 17) bilaterally as compared to healthy volunteers during flickering checkerboard task and increased activity in fusiform gyrus (Broadmann area 37) during facial perception paradigm. Our findings confirm the notion that PD patients show significant changes in the visual cortex system even before the visual symptoms are clinically evident. Further studies are necessary to evaluate the contribution of these abnormalities to the development visual symptoms in PD. © 2010 Movement Disorders Society

Date 2010-08-15
Language en
Library Catalog DOI.org (Crossref)
URL https://movementdisorders.onlinelibrary.wiley.com/doi/10.1002/mds.23101
Accessed 11.8.2025, 19:28:25
License http://onlinelibrary.wiley.com/termsAndConditions#vor
Volume 25
Pages 1590-1596
Publication Movement Disorders
DOI 10.1002/mds.23101
Issue 11
Journal Abbr Movement Disorders
ISSN 0885-3185, 1531-8257
Date Added 11.8.2025, 19:28:25
Modified 11.8.2025, 19:28:25

Notes:

Not Included: not on SC

Tags: EXCLUDED

“Accidental, really?” Attributional bias in patients with Parkinson's disease

Item Type Journal Article

Author Lea Decombe
Author Audrey Henry
Author Rene Decombe
Author Melissa Tir
Author Anne Doe de Maindreville
Author Lou Galland Hairabedian
Author Arthur Kaladjian
Author Delphine Raucher-Chene

Abstract Introduction: Among the cognitive domains impaired in Parkinson's disease (PD), social cognition has received particular attention in recent years. Nevertheless, attributional bias, a social-cognitive subdomain, has not yet been studied in this population, despite its potential relationship with neuropsychiatric symptoms, and despite the possibility that deep-brain stimulation of the subthalamic nucleus, an effective treatment for disabling motor symptoms, worsens cognitive impairment. The present study therefore compared the attributional bias of patients with PD (stimulated and nonstimulated subgroups) with that of controls. It also explored the potential correlations between patients' attributional bias and their clinical scores. Methods: Thirty-two patients with PD (12 stimulated and 20 nonstimulated) were recruited and matched with 32 healthy controls. Attributional bias was assessed using the Ambiguous Intentions Hostility Questionnaire, which yields three subscores: Hostility Bias, Aggression Bias, and Blame. Depressive symptoms (Hamilton Rating Scale for Depression), paranoid thoughts (Paranoia Scale), global cognition (Montreal Cognitive Assessment), and social functioning (Social Functioning Questionnaire) were also assessed. Results: Patients exhibited more hostile and aggressive biases than controls, especially in ambiguous situations. Stimulated patients had greater hostility and aggression biases and a higher blame score than controls in accidental situations. No significant differences were observed between stimulated and nonstimulated patients. Conclusion: To our knowledge, this is the first study to have assessed attributional bias in patients with PD and explored the impact of deep-brain stimulation on this particular subdomain of social cognition. Results suggest that patients exhibit attributional bias, and this impairment may be exacerbated in stimulated patients.

Date 2022-02
Language English
Extra Place: 125 London Wall, London, ENGLAND Type: Article
Volume 95
Publisher ELSEVIER SCI LTD
Pages 18-22
Publication PARKINSONISM & RELATED DISORDERS
DOI 10.1016/j.parkreldis.2021.12.013
ISSN 1353-8020
Date Added 14.7.2025, 14:50:30
Modified 5.9.2025, 14:32:43

Notes:

Not Included: not on SC

Tags: EXCLUDED

Action and emotion perception in Parkinson’s disease: A neuroimaging meta-analysis

Item Type Journal Article
Author Maria Arioli
Author Zaira Cattaneo

Author Maria Luisa Rusconi
Author Fabio Blandini
Author Marco Tettamanti
Abstract Patients with Parkinson disease (PD) may show impairments in the social perception. Whether these deficits have been consistently reported, it remains to be clarified which brain alterations subtend them. To this aim, we conducted a neuroimaging meta-analysis to compare the brain activity during social perception in patients with PD versus healthy controls. Our results show that PD patients exhibit a significantly decreased response in the basal ganglia (putamen and pallidum) and a trend toward decreased activity in the mirror system, particularly in the left parietal cortex (inferior parietal lobule and intraparietal sulcus). This reduced activation may be tied to a disruption of cognitive resonance mechanisms and may thus constitute the basis of impaired others' representations underlying action and emotion perception. We also found increased activation in the posterior cerebellum in PD, although only in a within-group analysis and not in comparison with healthy controls. This cerebellar activation may reflect compensatory mechanisms, an aspect that deserves further investigation. We discuss the clinical implications of our findings for the development of novel social skill training programs for PD patients.
Date 2022
URL https://www.sciencedirect.com/science/article/pii/S2213158222000961
Volume 35
Pages 103031
Publication NeuroImage: Clinical
DOI https://doi.org/10.1016/j.nicl.2022.103031
ISSN 2213-1582
Date Added 6.7.2025, 19:12:35
Modified 5.9.2025, 14:26:56

Notes:

Included – Meta-analysis

Social perception

To this aim, we conducted a neuroimaging *meta*-analysis to compare the brain activity during social perception in patients with PD versus healthy controls.

sample characteristics

We used a set of ALE *meta*-analyses to investigate the neural basis of impaired social perception in PD patients and their specificity with respect to healthy control individuals.

Using ALE, we carried out two different analyses: one on PD patients' data, and one on HC participants' data. After that, we contrasted the respective *meta*-analyses between the two groups.

Our literature selection started by searching on Pubmed (https://www.ncbi.nlm.nih.gov/pubmed/) for the following keyword strings: "social cognition Parkinson *f*MRI", "social cognition Parkinson PET", "theory of mind Parkinson *f*MRI", "theory of mind Parkinson PET", "empathy Parkinson *f*MRI", "empathy Parkinson PET", "emotion Parkinson *f*MRI", "emotion Parkinson PET", "face Parkinson *f*MRI", "face Parkinson PET", "body Parkinson *f*MRI", "body Parkinson PET", "social perception Parkinson *f*MRI", "social perception Parkinson PET", "biological motion Parkinson *f*MRI", "biological motion Parkinson PET", "point light display Parkinson *f*MRI", "point light display Parkinson PET", "action observation Parkinson *f*MRI", "action observation Parkinson PET", "facial Parkinson *f*MRI" and "facial Parkinson PET". The preliminary pool of 870 retrieved studies, after duplicate removal, was evaluated based on title and abstract.

N = 27

Main findings related to the review's scope

Social perception processes in PD patients recruited consistent activation in the right middle temporal gyrus and in the fusiform gyrus bilaterally, alongside the right posterior cerebellum. Further *meta*-analytic activations were found in the left inferior and middle occipital cortex.

Cluster #	Volume (mm^3)	x	y	z	Brain region
1	2088	−44	−74	−8	Left inferior occipital gyrus
		−42	−86	−2	Left middle occipital gyrus
		−40	−64	−14	Left fusiform gyrus
2	1608	48	−72	0	Right inferior occipital gyrus
		52	−62	8	Right middle temporal gyrus
3	776	44	−50	−22	Right fusiform gyrus/Right cerebellum lobule VI

Tags: Imaging, G-SC

Action observation produces motor resonance in Parkinson's disease

Item Type Journal Article
Author Judith Bek
Author Emma Gowen
Author Stefan Vogt
Author Trevor Crawford
Author Ellen Poliakoff
Abstract Observation of movement activates the observer's own motor system, influencing the performance of actions and facilitating social interaction. This motor resonance is demonstrated behaviourally through visuomotor priming, whereby response latencies are influenced by the compatibility between an intended action and an observed (task-irrelevant) action. The impact of movement disorders such as Parkinson's disease (PD) on motor resonance is unclear, as previous studies of visuomotor priming have not separated imitative compatibility (specific to human movement) from general stimulus-response compatibility effects. We examined visuomotor priming in 23 participants with mild-to-moderate PD and 24 healthy older adults, using a task that pitted imitative compatibility against general stimulus-response compatibility. Participants made a key press after observing a task-irrelevant moving human finger or rectangle that was either compatible or incompatible with their response. Imitative compatibility effects, rather than general stimulus-response compatibility effects, were found specifically for the human finger. Moreover, imitative compatibility effects did not differ between groups, indicating intact motor resonance in the PD group. These findings constitute the first unambiguous demonstration of imitative priming in both PD and healthy ageing, and have implications for therapeutic techniques to facilitate action, as well as the understanding of social cognition in PD.
Date 2018-06
Language English
Extra Place: 111 RIVER ST, HOBOKEN 07030-5774, NJ USA Type: Article
Volume 12
Publisher WILEY
Pages 298-311
Publication JOURNAL OF NEUROPSYCHOLOGY
DOI 10.1111/jnp.12133
Issue 2
ISSN 1748-6645
Date Added 14.7.2025, 14:50:34
Modified 5.9.2025, 14:28:05

Notes:

Not Included: not on SC
Tags: EXCLUDED

Advanced Parkinson disease patients have impairment in prosody processing

**Item Type**	Journal Article
**Author**	Luisa Albuquerque
**Author**	Mauricio Martins
**Author**	Miguel Coelho
**Author**	Leonor Guedes
**Author**	Joaquim J. Ferreira
**Author**	Mario Rosa
**Author**	Isabel Pavao Martins
**Abstract**	Background: The ability to recognize and interpret emotions in others is a crucial prerequisite of adequate social behavior. Impairments in emotion processing have been reported from the early stages of Parkinson's disease (PD). This study aims to characterize emotion recognition in advanced Parkinson's disease (APD) candidates for deep-brain stimulation and to compare emotion recognition abilities in visual and auditory domains. Method: APD patients, defined as those with levodopa-induced motor complications (N = 42), and healthy controls (N = 43) matched by gender, age, and educational level, undertook the Comprehensive Affect Testing System (CATS), a battery that evaluates recognition of seven basic emotions (happiness, sadness, anger, fear, surprise, disgust, and neutral) on facial expressions and four emotions on prosody (happiness, sadness, anger, and fear). APD patients were assessed during the ON state. Group performance was compared with independent-samples t tests. Results: Compared to controls, APD had significantly lower scores on the discrimination and naming of emotions in prosody, and visual discrimination of neutral faces, but no significant differences in visual emotional tasks. Conclusion: The contrasting performance in emotional processing between visual and auditory stimuli suggests that APD candidates for surgery have either a selective difficulty in recognizing emotions in prosody or a general defect in prosody processing. Studies investigating early-stage PD, and the effect of subcortical lesions in prosody processing, favor the latter interpretation. Further research is needed to understand these deficits in emotional prosody recognition and their possible contribution to later behavioral or neuropsychiatric manifestations of PD.
**Date**	2016-02-07
**Language**	English
**Extra**	Place: 530 WALNUT STREET, STE 850, PHILADELPHIA, PA 19106 USA Type: Article
**Volume**	38
**Publisher**	TAYLOR & FRANCIS INC
**Pages**	208-216
**Publication**	JOURNAL OF CLINICAL AND EXPERIMENTAL NEUROPSYCHOLOGY
**DOI**	10.1080/13803395.2015.1100279
**Issue**	2
**ISSN**	1380-3395
**Date Added**	14.7.2025, 14:50:36
**Modified**	5.9.2025, 14:25:24

Notes:

Included

sample characteristics

size: 42 advanced Parkinson disease (APD) and 43 HC matched by gender, age, and educational level

Parkinson's Disease type and duration: subset of PD patients with relatively preserved cognitive function, younger than 70 years of age, with levodopa-induced motor complications refractory to best medical treatment, Mduration= 14.6 (SD=6.0)

Medication: NA

Hoehn-Yahr: NA

UPDRS-3: NA

Gender (male): 20 males (48%)

averaged ages (SD, range): M= 62.5 (SD=7.0)

other neurological disease (tumor, stroke, etc.): No dementia

other major psychopathology: NA

origin country (or ethnicity): Portugal

method observational

instruments used in order to quantify the variables

Social cognition aspect: emotion recognition

Name of the task: the Comprehensive Affect Testing System (CATS) [ekman]

type of stimulus [face/voice etc., Ekman faces/other etc.]: faces recognition.

task condition: neutral, happiness, sadness, anger, fear, surprise, and disgust

type of stimulus [face/voice etc., Ekman faces/other etc.]: prosody recognition.

task condition: neutral, happiness, sadness, anger, and fear

operationalization: In "emotional discrimination" tasks, patients were asked to choose whether emotions of a pair of emotional stimuli (faces or sentences) were the "same" or "different."

In "emotional naming" tasks, patients were asked to listen (prosody) or attend (faces) to emotional stimuli and then to name the emotion presented, by choosing among several possible emotion labels depicted on the computer screen.

Answers had no time limits, and correct responses were converted to a percentage of the total.

Main findings related to the review's scope

For visual stimuli, there were no group differences either in the discrimination of emotional faces or in visual emotion naming.

Concerning auditory stimuli, patients had significantly lower scores than healthy controls in all measures: discrimination of emotional prosody, and naming emotional prosody

Tags: Emotion recognition, behavioral

Advanced Theory of Mind in patients at early stage of Parkinson's disease.

Item Type Journal Article

Author Rwei-Ling Yu
Author Ruey-Meei Wu
Author Ming-Jang Chiu
Author Chun-Hwei Tai
Author Chin-Hsien Lin
Author Mau-Sun Hua

Abstract Advanced Theory of Mind (ToM) refers to the sophisticated ability to infer other people's thoughts, intentions, or emotions in social situations. With appropriate advanced ToM, one can behave well in social interactions and can understand the intention of others' behavior. Prefrontal cortex plays a vital role in this ability, as shown in functional brain imaging and lesion studies. Considering the primary neuropathology of Parkinson's disease (PD) involving the frontal lobe system, patients with PD are expected to exhibit deficits in advanced ToM. However, few studies on this issue have been explored, and whether advanced ToM is independent of executive functions remains uncertain. Thirty-nine early non-demented PD patients and 40 normal control subjects were included. Both groups were matched in age, level of education, and verbal intelligence quotient. Each participant received advanced ToM, executive functions, and verbal intelligence quotient tests. We discovered that the performance of the PD patients on the Cartoon ToM task was significantly poorer than that of their normal counterparts. Correlation analysis revealed that performance scores of advanced ToM in PD patients were significantly associated with their executive functions scores; however, this is not the case for normal controls. We conclude that dysfunction of advanced ToM develops in early PD patients, who require more cognitive abilities than their normal counterparts to generate advanced ToM. Our findings might be helpful in developing educational and medical care programs for PD patients in the future.

Date 2012 Jan
Language eng
License Copyright © 2011 Elsevier Ltd. All rights reserved.
Extra Place: England
Volume 18
Pages 21-24
Publication Parkinsonism & related disorders
DOI 10.1016/j.parkreldis.2011.08.003
Issue 1
Journal Abbr Parkinsonism Relat Disord
ISSN 1873-5126 1353-8020
PMID 21868278
Date Added 6.7.2025, 19:09:41
Modified 5.9.2025, 15:02:08

Notes:

Included

Sample characteristics

Size: 39 PD, 40 HC

PD-type: Idiopathic PD

PD-duration: Range = 1-13, M = 4.3, SD = 2.8

Medication: L-Dopa equivalent dose mentioned (no further information). Likely just continuation of medication in ON.

Hoehn-Yahr: Range = 1-2, M = 1.6, SD = 0.5

UPDRS-3: Off-Therapy! Range = 4-39, M = 18.9, SD = 8.6

Gender (male): 25 (64%)

Age: Range = 55-73, M = 62.7, SD = 4.2

Other neurological disease (tumor, stroke, etc.): NA

Other major psychopathology: No dementia (MMSE >23), no depression, no serious systemic or psychiatric disorders

Origin country (or ethnicity): Taiwan

method obversational

instruments used in order to quantify the variables

Social cognition aspect: ToM

Name of the task:

1. Faux Pas Recognition test (FPR)
2. Implication Stories test (IS)
3. Cartoon ToM tasks (CTOM)

Type of stimulus [face/voice etc., Ekman faces/other etc.]:

1. 10 Stories, where the protagonist says something inappropriate in the social situation.
2. 5 short stories which were selected from Happe's set.
3. 10 funny cartoon pictures in which the humor depends upon what a character mistakenly think or does not know

Task condition:

1. After the story, the participants were asked three questions. First, the participants must indicate if anyone in the story said something inappropriate. The second question concerns who made the faux pas and the third concerns how do the other character feel about the situation.
2. Each story was followed by two questions to assess whether participants could understand the implied meaning in the story. The first question requires a judgment about the social situation and the second question requires the inference about the characters' thoughts and intentions.
3. Participants were shown the pictures one at a time, with the instruction to tell the experimenter why each was funny and what the intention of the protagonists in each picture was.

Operationalization: Number of correct answers for all three tasks

Main findings related to the review's scope

A significant group difference in the CTOM scores.

No significant difference in the FPR and IS scores between the two groups.

FPR and IS scores fell below the 5th percentile of NCs in 10.5% and 13.2% of early PD patients, respectively.

(No groups differences in EF, but different correlations of EF and SC between groups!)
Tags: ToM, behavioral

Affective and cognitive Theory of Mind in patients with parkinson's disease.

Item Type Journal Article
Author Maren E. Bodden
Author Brit Mollenhauer
Author Claudia Trenkwalder
Author Nicole Cabanel
Author Karla Maria Eggert
Author Marcus Michael Unger
Author Wolfgang Hermann Oertel
Author Josef Kessler
Author Richard Dodel
Author Elke Kalbe
Abstract Theory of Mind (ToM), which is the ability to infer other people's mental states such as beliefs or desires, is an important prerequisite for social interaction. Affective and cognitive subcomponents of ToM can be impaired selectively in neurological and psychiatric disorders. This study examines ToM in 21 Parkinson's disease (PD) patients and 21 healthy control (HC) subjects, using the computerized "Yoni task" that assesses affective and cognitive ToM abilities and an extensive battery of neuropsychological tests. Furthermore, questionnaires to assess health-related quality of life and depressive symptoms were applied and correlations to ToM were investigated. Compared to the control subjects, PD patients scored lower on both the affective (PD: 76% versus HC: 89%; p = 0.006) and cognitive (PD: 80% versus HC: 92%; p = 0.002) ToM subscales but not on control items (PD: 90% versus HC: 95%; p = 0.077). The ToM abilities were not associated with other cognitive functions, depressive symptoms or clinical data. However, affective ToM was correlated with health-related quality of life (p = 0.01). Parkinson patients are impaired in affective as well as cognitive ToM. These deficits are largely independent from other cognitive impairments, depressive symptoms and motor impairment. The relationship of affective ToM to the health-related quality of life of PD patients points to a clinical relevance of this issue and suggests that ToM dysfunctions must be regarded as an important non-motor feature of Parkinson's disease.
Date 2010 Aug
Language eng
Extra Place: England
Volume 16

**Pages**	466-470
**Publication**	Parkinsonism & related disorders
**DOI**	10.1016/j.parkreldis.2010.04.014
**Issue**	7
**Journal Abbr**	Parkinsonism Relat Disord
**ISSN**	1873-5126 1353-8020
**PMID**	20538499
**Date Added**	6.7.2025, 19:09:40
**Modified**	5.9.2025, 14:29:02

Notes:

Included

sample characteristics

size: 21 PD and 21 HC

Parkinson's Disease type and duration: idiopathic PD, Mduration= 5.1 SD= 2.8

Medication: on medication

Hoehn-Yahr: Md= 2.5, range 1–3

UPDRS-3: M=NA

Gender (male): 15 males (71%)

averaged ages (SD, range): M= 63.7 SD= 10.0

other neurological disease (tumor, stroke, etc.): no dementia

other major psychopathology: NA

origin country (or ethnicity): NA

method observational

instruments used in order to quantify the variables

Social cognition aspect: ToM

Name of the task: The "Reading the Mind in the Eyes Test" (RMET)

type of stimulus [face/voice etc., Ekman faces/other etc.]: photographs of eye regions

operationalization: accuracy

Name of the task: the "Yoni task"

type of stimulus [face/voice etc., Ekman faces/other etc.]: a face named "Yoni" is shown in the middle with four coloured pictures in the corners showing either faces and/or examples of a semantic category (e.g. animals, fruits). Participants have to evaluate which of these four pictures best corresponds to a sentence contemporaneously presented on each screen about which image Yoni is referencing. The items can be subdivided into three types of categories that correspond to affective ToM (e.g. Yoni likes…), cognitive ToM (e.g. Yoni is thinking of…) and control conditions (e.g. Yoni is near by…). While answers for the control condition only require an analysis of the physical attributes of the character, choices in the affective and cognitive ToM items imply mentalizing based on verbal cues contained in the sentences, eye gaze and/or facial expression. Items differ in complexity with both first order (Yoni's mental state should be inferred, examples as described above) and second order items included (Yoni's ToM process should be inferred, such as "Yoni likes the fruit that …dislikes" for affective ToM, "Yoni is thinking of the car that …wants" for cognitive ToM as compared to "Yoni has the flower that …has" in the control condition).

operationalization: accuracy

<u>**Main findings related to the review's scope**</u>

Compared to the HC, the PD group scored lower in the RMET

PD patients scored significantly lower than HC in affective as well as cognitive ToM

PD patients scored lower than HC on affective second order ToM items as well as on cognitive second order ToM items

Tags: ToM, behavioral

Affective judgments by patients with Parkinson’s disease or chronic progressive multiple sclerosis

**Item Type**	Journal Article
**Author**	William W. Beatty
**Author**	Donald E. Goodkin
**Author**	William S. Weir
**Author**	R. Dennis Staton
**Author**	Nancy Monson
**Author**	Patricia A. Beatty
**Date**	4/1989
**Language**	en
**Library Catalog**	DOI.org (Crossref)
**URL**	http://link.springer.com/10.3758/BF03334628
**Accessed**	11.8.2025, 18:56:40
**License**	http://www.springer.com/tdm
**Volume**	27
**Pages**	361-364
**Publication**	Bulletin of the Psychonomic Society
**DOI**	10.3758/BF03334628
**Issue**	4
**Journal Abbr**	Bull. Psychon. Soc.
**ISSN**	0090-5054
**Date Added**	11.8.2025, 18:56:40
**Modified**	11.8.2025, 18:56:40

Notes:

Included

sample characteristics

size: 43 PD and 27 HC

Parkinson's Disease type and duration: idiopathic, Mduration= 4.7

Medication: on medication

Hoehn-Yahr: Mo=2,3 1-4

UPDRS-3: NA

Gender (male): NA

averaged ages (SD, range): M= 65.8

other neurological disease (tumor, stroke, etc.): none

other major psychopathology: none

origin country (or ethnicity): NA

method observational

instruments used in order to quantify the variables

Social cognition aspect: emotion recognition

Name of the task: NA

type of stimulus [face/voice etc., Ekman faces/other etc.]: Ekman faces

task conditions: NA

operationalization: accuracy – total score

Main findings related to the review's scope

PD were less accurate then HC
Tags: Emotion recognition, behavioral

Affective priming of body and facial expressions in Parkinson's disease

**Item Type**	Journal Article
**Author**	Chiara Longo
**Author**	Giulia Mattavelli
**Author**	Alice Beati
**Author**	Maria Pennacchio
**Author**	Bryan Bertoldi

Author Maria Chiara Malaguti
Author Costanza Papagno
Date 2025-03-20
Publication COGNITIVE AFFECTIVE & BEHAVIORAL NEUROSCIENCE
DOI 10.3758/s13415-025-01290-4
ISSN 1530-7026
Date Added 14.7.2025, 14:48:38
Modified 5.9.2025, 14:43:59

Notes:

Included

sample characteristics

size: 25 PD and 25 HC matched in age and education.

Parkinson's Disease type and duration: Mduration = 7.42, SD=5.01

Medication: On

Hoehn-Yahr: M=2.02 SD= 0.64

UPDRS-3: NA

Gender (male): 15 males (60%)

averaged ages (SD, range): mean = 66.24, SD = 6.81

other neurological disease (tumor, stroke, etc.): NA

other major psychopathology: NA

origin country (or ethnicity): NA

method observational

instruments used in order to quantify the variables

Social cognition aspect: emotion recognition

Name of the task: NA

type of stimulus [face/voice etc., Ekman faces/other etc.]:

Bodies: validated database (Borgomaneri et al., 2012, 2015). Color static pictures of bodies performing emotional actions of happiness and fear, neutral postures (i.e., actions which implied movements comparable to emotional body expres sions but with no emotional meaning), and static-neutral postures. Specifically, the neutral postures depict nonemotional actions, whereas the static-neutral stimuli consisted in nonaction postures, i.e., standing positions with arms relaxed alongside the torso. Faces were blanked out in all stimuli to focus on body postures information.

task condition: happy, fear, neutral postures, static-neutral

Faces: taken from the NimStim database, eight colored male faces.

task condition: happy, fearful, and neutral expressions.

operationalization:

Two separate blocks of the subliminal priming task were presented to participants. In the priming Face-Body task, target stimuli were bodies with happy or fearful postures, preceded by a short presentation (17 ms) of happy, fearful or neutral faces combined in congruent (i.e., happy face – happy body / fearful face – fearful body) incongruent (i.e., happy face – fearful body / fearful face – happy body) or

neutral (i.e., neutral face – happy body / neutral face – fearful body) pairs. Prime stimuli were preceded and followed by a random-dot mask (obtained by scram bling the face stimuli) lasting 34 ms and 68 ms. Participants were instructed to select the target emotion by pressing the corresponding one or two keyboard buttons. A total of 192 trials were presented, interleaved by 800-ms fixation cross. In the priming Body-Face task, target stimuli were happy and fearful faces preceded by a short presentation (17 ms) of a body with happy, fearful or static-neutral posture, combined as above in congruent (i.e., happy body – happy face / fearful body – fearful face) incongruent (i.e., happy body – fearful face / fearful body – happy face) or neutral (i.e. static-neutral body – happy face / static-neutral body – fearful face) pairs. Random-dot masks were obtained by scrambling the body stimuli and were presented before and after the primes for 34 ms and 68 ms, respectively. Participants were instructed to select the target emotion by pressing the corresponding one or two keyboard buttons.

After the priming tasks, participants completed two recognition tasks with body or face stimuli separately. The stimuli were the same as those used in the priming tasks, with the addition of nonemotional neutral postures for the body recognition task. Face or body stimuli were presented at the center of the screen and participants were asked to select the emotions that best described the target stimuli by selecting the emotional label provided below the picture (happiness, fear, or neutral)

RTs and Accuracy.

Main findings related to the review's scope

Face-Body task: Accuracy: (1) beyond groups, higher accuracy for fear than happy body emotion. (2) beyond groups, accuracy was higher for congruent than neutral and incongruent. (3) Post hoc for the three-way interaction (emotion by congruency by group) showed that differences between congruency conditions were significant only in the HC group.

RT: (1) RTs were overall longer for happy than fearful. (2) HC were faster than PD. (3) RTs were longer in response to happy than fearful only for PD

Body-Face task: accuracy: high accuracy score for both PD and HC.

RT: (1) RTs were faster for happy than fearful target faces. (2) HC were faster than PD patients. (3) RTs of the PD group were significantly longer for fearful faces than for happy faces in both congruent and incongruent conditions, but not in the neutral condition.

Recognition tasks:

Body: (1) Accuracy significantly differed in all pairwise contrasts between emotional bodies (all ps < .02), with mean accuracy of 0.92 (SD = 0.16) for fearful, 0.87 (SD = 0.21) for happy, 0.63 (SD = 0.28) for neutral postures, and 0.95 (SD = 0.1) for static bodies. (2) The post-hoc analyses on the emotion by group interaction showed no significant differences between HC and PD in the pairwise contrasts for each single emotion. However, the emotional body condition had a different effect on accuracy in the two groups: in PD, all pairwise comparisons between emotions were significant (all ps < .05), except for the fear vs. static body expressions contrast, which were both better recognized than happy and static postures. Differently from PD, in HC the comparison between happy and fearful bodies was not significant.

Simillar findings on RTs.

Faces: (1) beyond groups, higher accuracy for happy faces compared with fearful and neutral faces. (2) beyond groups, faster RTs for happy faces than fearful and neutral. (3) PD were significantly slower

than HC for happy and fearful faces, but not for neutral faces.
Tags: Emotion recognition, behavioral

Affective theory of mind in Parkinson's disease: the effect of cognitive performance.

**Item Type**	Journal Article
**Author**	Ana-Maria Romosan
**Author**	Liana Dehelean
**Author**	Radu-Stefan Romosan
**Author**	Minodora Andor
**Author**	Ana Cristina Bredicean
**Author**	Mihaela Adriana Simu
**Abstract**	PURPOSE: Among other non-motor symptoms, theory of mind (ToM), the ability to recognize, understand and infer others' mental states, beliefs, intents and wishes, has been shown to deteriorate during the course of Parkinson's disease (PD). It has been speculated that ToM impairments could be related to cognitive deficits in PD. However, the current state of literature suggests that there is heterogeneity regarding the involvement of cognitive functioning in the relationship of PD and ToM. The study aimed to measure affective ToM abilities and cognitive performance in a sample of PD patients, to explore the link between affective ToM abilities and cognitive status, and to examine the impact of PD on affective ToM through the mediator effect of cognitive performance. PATIENTS AND METHODS: Sixty-five patients diagnosed with idiopathic PD and 51 healthy controls matched for age, gender and educational level completed a visual affective ToM task (Reading the Mind in the Eyes - RMET), cognitive performance was evaluated with Montreal Cognitive Assessment, and psychiatric symptoms were measured with BPRS-E (Brief Psychiatric Rating Scale). RESULTS: Affective ToM abilities were preserved in early PD patients, declining as the disease progressed. Deficits in cognitive functioning predicted deficiencies in affective ToM. Although attention (AT), executive functions (EF) and visuospatial abilities (VSA) together mediated the relationship between PD and affective ToM, only VSA impairment had a specific negative impact on affective ToM. Moreover, 41% of the total effect of attention and executive functions on affective ToM was mediated by visuospatial skills. CONCLUSION: Cognitive performance may have an impact on the relationship between PD and affective ToM through the involvement of VSA. The influence of AT and EF in this relationship appears to be also exerted by PD patients' VSA.
**Date**	2019
**Language**	eng
**License**	© 2019 Romosan et al.
**Extra**	Place: New Zealand
**Volume**	15
**Pages**	2521-2535
**Publication**	Neuropsychiatric disease and treatment
**DOI**	10.2147/NDT.S219288
**Journal Abbr**	Neuropsychiatr Dis Treat
**ISSN**	1176-6328 1178-2021

PMID 31564879
PMCID PMC6722434
Date Added 6.7.2025, 19:09:41
Modified 5.9.2025, 14:53:43

Notes:

Included

Sample characteristics

Size: 65 PD, 51 HC (matched for age, gender, education)

PD-type: Idiopathic PD

PD-duration: M = 76.43, Sd = 38.04 (months)

Medication: Assessment in ON

Hoehn-Yahr: 1= 7, 1.5 = 17, 2 = 13, 2.5 = 14, 3 = 14

UPDRS-3: NA

Gender (male): 38 (58.5 %)

Age: M = 58.07, Sd = 5.25

Other neurological disease (tumor, stroke, etc.): None

Other major psychopathology: None

Origin country (or ethnicity): Romania

method behavioural

instruments used in order to quantify the variables

Social cognition aspect: Affective ToM

Name of the task: Reading the Mind in the Eyes Test (REMT)

Type of stimulus [face/voice etc., Ekman faces/other etc.]: h 36 photographs depicting the eye region of a Caucasian actor/actress. Each photograph was printed with four possible mental state descriptors around it (eg, "suspicious," "ashamed," "frightened," "serious")

Task condition: Subjects were asked to choose from the four words the one that best describes what the actor illustrated in the photograph might be feeling or thinking

Operationalization: Correct answers: The maximum score that could be obtained was 36

Main findings related to the review's scope

No sig. diff between HC vs mild PD (H&Y between 1-2)

HC sig. better than moderate PD (H&Y between 2.5-3)

mild PD sig. bettern than moderate PD

Tags: ToM, Behavioral

Affective theory of mind in patients with Parkinson's disease: comment.

Item Type Journal Article
Author Gabriella Santangelo
Author Carmine Vitale
Author Domenico Errico
Author Dario Grossi
Author Luigi Trojano
Author Paolo Barone
Date 2014 Mar
Language eng
Extra Place: Australia
Volume 68
Pages 242
Publication Psychiatry and clinical neurosciences
DOI 10.1111/pcn.12116
Issue 3
Journal Abbr Psychiatry Clin Neurosci
ISSN 1440-1819 1323-1316
PMID 24895739
Date Added 6.7.2025, 19:09:41
Modified 5.9.2025, 14:55:15

Notes:

Not included: Comment to a different article

Tags: EXCLUDED

Affective theory of mind in patients with Parkinson's disease.

Item Type Journal Article
Author Michele Poletti
Author Andrea Vergallo
Author Martina Ulivi
Author Alessandro Sonnoli
Author Ubaldo Bonuccelli
Abstract AIM: The aim of this study was to assess the hypothesis that patients with Parkinson's disease (PD) may have difficulties in tasks of affective theory of mind (ToM; the inference on others' feelings) especially in moderate/advanced PD stages. Difficulties of cognitive ToM have already been described in several previous studies. METHODS: Affective ToM was assessed with the Reading the Mind in the Eyes task in 35 PD patients and 35 healthy controls. Depression, global cognitive status and executive functioning were also evaluated. Patients were distinguished in early PD and moderate PD according to their scores in the Hoehn and Yahr Staging Scale. RESULTS: PD patients had more difficulties with affective ToM than healthy controls, also controlling for other variables that resulted in association with this ability. Early PD patients outperformed moderate PD patients, but this difference did

not reach statistical significance when controlling for other variables.
CONCLUSION: These findings confirmed that affective ToM may be impaired in PD, but any conclusion can be made on the effect of disease progression on this ability of social cognition. Therefore, longitudinal studies are needed to investigate this potential effect.

**Date**	2013 May
**Language**	eng
**License**	© 2013 The Authors. Psychiatry and Clinical Neurosciences © 2013 Japanese Society of Psychiatry and Neurology.
**Extra**	Place: Australia
**Volume**	67
**Pages**	273-276
**Publication**	Psychiatry and clinical neurosciences
**DOI**	10.1111/pcn.12045
**Issue**	4
**Journal Abbr**	Psychiatry Clin Neurosci
**ISSN**	1440-1819 1323-1316
**PMID**	23683159
**Date Added**	6.7.2025, 19:09:40
**Modified**	5.9.2025, 14:52:04

Notes:

Included

Sample characteristics

Size: 35 PD, 35 HC (matched for age)

PD-type: Idiopathic PD

PD-duration: M = 6.31, SD = 4.02

Medication: LEDD calculated

Hoehn-Yahr: M = 2.08, SD = 0.72

UPDRS-3: NA

Gender (male): 22 (63%)

Age: M = 68.45, SD = 6.69

Other neurological disease (tumor, stroke, etc.): none (at least for HC)

Other major psychopathology: none (at least for HC)

Origin country (or ethnicity): NA

method behavioural

instruments used in order to quantify the variables

Social cognition aspect: ToM

Name of the task: Reading the Mind in the Eyes (RME)

Type of stimulus [face/voice etc., Ekman faces/other etc.]: 36 black and white photographs of the eye region

Task condition: 4 way foreced choice task

Operationalization: Correct answers. Max socre = 36

Main findings related to the review's scope

HC outperformed PD patients in the RME (P < 0.01).

early PD (H&Y Stages 1, 1.5 and 2) patients outperformed moderate PD patients (H&Y Stage 2.5 and 3)

Tags: ToM, behavioral

Alterations in event-related potential responses to empathy for pain in Parkinson's disease on and off medication

Item Type Journal Article

Author Panpan Hu
Author Ruihua Cao
Author Juan Fang
Author Qian Yang
Author Tingting Liu
Author Fengqiong Yu
Author Kai Wang
Abstract Objective How Parkinson's disease (PD) affects an individual's empathic capacity remains poorly understood. By using the event-related potential (ERP) technique, we sought to: (1) study the temporal dynamics of empathic responses in patients with PD; (2) explore whether dopaminergic medication modulates empathic processing. Methods Twenty-six patients with early-to-moderate PD (13 on– and 13 off-medication) and 14 healthy controls performed an empathy-for-pain paradigm test while we recorded their electroencephalography. The participants responded to neutral or painful pictures during an active empathic condition (pain judgment task) and a control condition that was manipulated by task demands (laterality judgment task). Results The ERP results demonstrated an early automatic frontal response and a late controlled parietal response to pain in healthy elderly controls. The observed early and late ERP responses were detected in the on-medication patients but not in the off-medication patients. Conclusions PD is associated with deficits in both affective and cognitive empathic responses, dopaminergic medication may have the potential to alleviate these deficits. Significance This study helps to understand empathic deficits in patients with PD. Within-subject studies are required to reliably assess the effect of dopaminergic medication on empathic processing.
Date 2021
URL https://www.sciencedirect.com/science/article/pii/S1388245721000262
Volume 132
Pages 914-921
Publication Clinical Neurophysiology
DOI https://doi.org/10.1016/j.clinph.2020.12.020
Issue 4
ISSN 1388-2457
Date Added 6.7.2025, 19:12:35
Modified 5.9.2025, 14:39:37

Notes:

Included

sample characteristics

size: 26 PD and 14 HC sex- and age- matched

Parkinson's Disease type and duration: PD-on Mduration=2.0 SD=1.1, PD-off Mduration=1.6 sd=0.7

Medication: 13 on 13 off

Hoehn-Yahr: PD-on M=1.3 sd= 0.4, PD-off M=1.4 SD= 0.4

UPDRS-3: assessed for both in "on" condition PD-on M=21.0 SD= 7.3, PD-off M=21.2 SD=9.1

Gender (male): PD-on 8 (62%), PD-off 10 (77%)

averaged ages (SD, range): PD-on M=58.0 SD=10.5, PD-off M=58.3 SD=9.2

other neurological disease (tumor, stroke, etc.): No dementia

other major psychopathology: NA

origin country (or ethnicity): China

method (Review, meta-analysis or observational and/or self-reported):

instruments used in order to quantify the variables

Social cognition aspect: Empathy

Name of the task: NA

type of stimulus [face/voice etc., Ekman faces/other etc.]: The visual stimuli consisted of 140 digital pictures that showed hands or feet in painful or neutral conditions (without faces being visible). The pictures were taken from the first-person perspective. The painful pictures depicted 35 incidents that may occur during a normal day (such as a hand cut by scissors or trapped in a door), and each of them contained four conditions: i) the left body part in a painful situation, ii) the left body part in a neutral situation, iii) the right body part in a painful situation, and iv) the right body part in a neutral situation.

task condition: painful-right, painful-left, neutral-right, neutral-lest

operationalization: In the pain judgment block, the subjects were asked to judge if the person in the picture was experiencing pain.

reaction time and accuracy.

Main findings related to the review's scope

patients with PD performed worse on the painful stimuli than the healthy controls (on-medication patients vs. HCs: P = 0.072; off-medication patients vs. HCs: P = 0.048), thus suggesting that the patients tended to judge painful pictures as neutral, compared to the controls.

Tags: empathy, behavioral

Altered emotional recognition and expression in patients with Parkinson's disease.

**Item Type**	Journal Article
**Author**	Yazhou Jin
**Author**	Zhiqi Mao
**Author**	Zhipei Ling
**Author**	Xin Xu
**Author**	Zhiyuan Zhang
**Author**	Xinguang Yu
**Abstract**	BACKGROUND: Parkinson's disease (PD) patients exhibit deficits in emotional recognition and expression abilities, including emotional faces and voices. The aim of this study was to explore emotional processing in pre-deep brain stimulation (pre-DBS) PD patients using two sensory modalities (visual and auditory). METHODS: Fifteen PD patients who needed DBS surgery and 15 healthy, age- and gender-matched controls were recruited as participants. All participants were assessed by the Karolinska Directed Emotional Faces database 50 Faces Recognition test. Vocal recognition was evaluated by the Montreal Affective Voices database 50 Voices Recognition test. For emotional facial expression, the participants were asked to imitate five basic emotions (neutral, happiness, anger, fear, and sadness). The subjects were required to express nonverbal vocalizations of the five basic emotions. Fifteen Chinese native speakers were recruited as decoders. We recorded the accuracy of the responses, reaction time, and confidence level. RESULTS: For emotional recognition and expression, the PD group scored lower on both facial and vocal emotional processing than did the healthy control group. There were significant differences between the two groups in both reaction time and confidence level. A significant relationship was also found between emotional recognition and emotional expression when considering all participants between the two groups together. CONCLUSION: The PD group exhibited poorer performance on both the recognition and expression tasks. Facial emotion deficits and vocal emotion abnormalities were associated with each other. In addition, our data allow us to speculate that emotional recognition and expression may share a common system.
**Date**	2017
**Language**	eng
**Extra**	Place: New Zealand
**Volume**	13
**Pages**	2891-2902
**Publication**	Neuropsychiatric disease and treatment
**DOI**	10.2147/NDT.S149227
**Journal Abbr**	Neuropsychiatr Dis Treat
**ISSN**	1176-6328 1178-2021
**PMID**	29225467
**PMCID**	PMC5708195
**Date Added**	6.7.2025, 19:09:35
**Modified**	5.9.2025, 14:40:45

Notes:

Included

sample characteristics

size: 15 PD and 15 healthy, age- and gender-matched controls

Parkinson's Disease type and duration: NA, Mduration = 7.93 SD=2.31

Medication: on medication

Hoehn-Yahr: M=2.53 SD=0.74

UPDRS-3: M= 29.87 SD=15.63

Gender (male): 11 males (73%)

averaged ages (SD, range): M= 61.73 SD=8.59

other neurological disease (tumor, stroke, etc.): None

other major psychopathology: None

origin country (or ethnicity): China

method (Review, meta-analysis or observational and/or self-reported):

instruments used in order to quantify the variables

Social cognition aspect: emotion recognition

Name of the task: Faces Recognition test

type of stimulus [face/voice etc., Ekman faces/other etc.]: the Karolinska Directed Emotional Faces (KDEF) database

task condition: neutral, happiness, anger, fear, and sadness

operationalization: ACCURACY

Name of the task: Faces Recognition test

type of stimulus [face/voice etc., Ekman faces/other etc.]: the MAV database

task condition: neutral, happiness, anger, fear, and sadness

operationalization: ACCURACY

Main findings related to the review's scope

Total KDEF of PD was lower then HC.

lower scores for the KDEF sub-score of sadness in the PD group than those in the HC group.

Total MAV of PD was lower then HC.

The MAV sub-score of neutral in the PD group was lower than that in the HC group

There were no other significant differences between the two groups
Tags: Emotion recognition, behavioral

Altered Kinematics of Facial Emotion Expression and Emotion Recognition Deficits Are Unrelated in Parkinson's Disease.

Item Type Journal Article
Author Matteo Bologna
Author Isabella Berardelli
Author Giulia Paparella
Author Luca Marsili
Author Lucia Ricciardi
Author Giovanni Fabbrini
Author Alfredo Berardelli
Abstract BACKGROUND: Altered emotional processing, including reduced emotion facial expression and defective emotion recognition, has been reported in patients with Parkinson's disease (PD). However, few studies have objectively investigated facial expression abnormalities in PD using neurophysiological techniques. It is not known whether altered facial expression and recognition in PD are related. OBJECTIVE: To investigate possible deficits in facial emotion expression and emotion recognition and their relationship, if any, in patients with PD. METHODS: Eighteen patients with PD and 16 healthy controls were enrolled in this study. Facial expressions of emotion were recorded using a 3D optoelectronic system and analyzed using the facial action coding system. Possible deficits in emotion recognition were assessed using the Ekman test. Participants were assessed in one experimental session. Possible relationship between the kinematic variables of facial emotion expression, the Ekman test scores, and clinical and demographic data in patients were evaluated using the Spearman's test and multiple regression analysis. RESULTS: The facial expression of all six basic emotions had slower velocity and lower amplitude in patients in comparison to healthy controls (all Ps < 0.05). Patients also yielded worse Ekman global score and disgust, sadness, and fear sub-scores than healthy controls (all Ps < 0.001). Altered facial expression kinematics and emotion recognition deficits were unrelated in patients (all Ps > 0.05). Finally, no relationship emerged between kinematic variables of facial emotion expression, the Ekman test scores, and clinical and demographic data in patients (all Ps > 0.05). CONCLUSION: The results in this study provide further evidence of altered emotional processing in PD. The lack of any correlation between altered facial emotion expression kinematics and emotion recognition deficits in patients suggests that these abnormalities are mediated by separate pathophysiological mechanisms.
Date 2016
Language eng
Extra Place: Switzerland
Volume 7
Pages 230
Publication Frontiers in neurology
DOI 10.3389/fneur.2016.00230
Journal Abbr Front Neurol

ISSN 1664-2295
PMID 28018287
PMCID PMC5155007
Date Added 6.7.2025, 19:09:35
Modified 5.9.2025, 14:29:13

Notes:

Included

sample characteristics

size: 18 PD and 16 HC matched for age, gender, and education

Parkinson's Disease type and duration: idiopathic PD, Mduration=5.9 SD=2.3

Medication: on medication

Hoehn-Yahr: M=NA

UPDRS-3: M=16.9 SD=3.9

Gender (male): 11 males (61%)

averaged ages (SD, range): M= 58.8 SD= 5.5

other neurological disease (tumor, stroke, etc.): None

other major psychopathology: None

origin country (or ethnicity): Italy

method observational

instruments used in order to quantify the variables

Social cognition aspect: Emotion recognition

Name of the task: the Ekman 60 Faces test

type of stimulus [face/voice etc., Ekman faces/other etc.]: Ekman faces, 5 males and 5 females

task condition: anger, disgust, fear, happiness, sadness, and surprise

operationalization: accuracy

Main findings related to the review's scope

The overall Ekman score of the PD group was significantly lower than that of the HC

Significantly lower scores for the sub-scores of disgust, fear, anger and sadness in PD patients than in HC

Tags: Emotion recognition, behavioral

Altered moral decision-making in patients with idiopathic Parkinson's disease.

Item Type Journal Article
Author Jan B. Rosen

Author Elisa Rott
Author Georg Ebersbach
Author Elke Kalbe

Abstract BACKGROUND: Moral decision-making essentially contributes to social conduct. Although patients with Parkinson's disease (PD) show deficits in (non-moral) decision making and related neuropsychological functions, i.e. executive functions, theory of mind (ToM), and empathy, moral decision-making has rarely been examined in PD patients. We examined possible alterations of moral decision-making and associated functions in PD. METHODS: Twenty non-demented PD patients and 23 age- and education-matched healthy control participants were examined with tests that assess reasoning, executive functions (set-shifting and planning), ToM and empathy, decision-making under risk, and moral intuitions. Moral decision-making was assessed with a close-to-everyday moral dilemma paradigm that opposes socially oriented "altruistic" choices to self-beneficial "egoistic" choices in 20 moral dilemma short stories (10 high and 10 low emotional). Concurrently, electrodermal activity was recorded. RESULTS: PD patients made more egoistic moral decisions than healthy controls. Remarkably, while reasoning, planning and empathy correlated with moral decision-making in the control group, in the PD group neuropsychological functions and dopaminergic medication did not correlate with moral decisions. No evidence for reduced skin conductance responses in PD patients and no relationships between skin conductance responses and moral decisions were observed. CONCLUSIONS: This study provides evidence for moral decision-making dysfunctions in PD patients who made more egoistic moral decisions. As a possible underlying mechanism, reduced exercise of attentional control due to a dysfunctional interplay between the prefrontal cortex and the basal ganglia is discussed. Future research will have to determine the impact of PD patients' moral decision-making dysfunctions on everyday life and further determine correlates of the deficits.

Date 2015 Oct
Language eng
License Copyright © 2015 Elsevier Ltd. All rights reserved.
Extra Place: England
Volume 21
Pages 1191-1199
Publication Parkinsonism & related disorders
DOI 10.1016/j.parkreldis.2015.08.016
Issue 10
Journal Abbr Parkinsonism Relat Disord
ISSN 1873-5126 1353-8020
PMID 26318961
Date Added 6.7.2025, 19:09:42
Modified 5.9.2025, 14:53:56

Notes:

Included

Sample characteristics

Size: 21 PD, 23 HC (age, sex, education matched)

PD-type: Idiopathic PD

PD-duration: M = 8.40, SD = 6.92

Medication: LEDD calculated

Hoehn-Yahr: Range = 2-5; M=2.72 SD=0.82

UPDRS-3: M = 34.18, SD = 13.68

Gender (male): 14 (70%)

Age: M = 67.45, Sd = 6.84

Other neurological disease (tumor, stroke, etc.): none

Other major psychopathology: Six PD patients, but none of the HC participants, received antidepressant medication.

Origin country (or ethnicity): Germany

method behavioural

instruments used in order to quantify the variables

Social cognition aspect: Affective ToM

Name of the task: Reading the mind in the eyes test (RMET)

Type of stimulus [face/voice etc., Ekman faces/other etc.]: 36pictures of eyes

Task condition: 4-way forced choice

Operationalization: Correct answer (max = 36)

Type of stimulus [face/voice etc., Ekman faces/other etc.]: 20 short stories describing everyday moral dilemma situations

Task condition: Every short story was presented on one screen, with a subsequent forced choice “yes” or “no” question on whether the subject would choose a proposed behavior, if he or she were confronted with the described situation.

Subdivided into high- and low emotional stories.

On the proposed behaviors in forced-choice questions following the stories, half (n 10) were designed to be “egoistic” while the other half (n 10) were to be “altruistic”

Operationalization: For high emotional (involving a second person that is present), low emotional (involving a second person that is not present), and the whole set of stories, the percentage of altruistic decisions was calculated as the moral decision-making score

Main findings related to the review's scope

No sig. group diff in RMET (p = .126, d = 0.47)

Results show that PD patients made significantly more egoistic decisions in the Moral decision-making (MDM) total and high emotional score, but not in low emotional situations

The results support our main results by significant total moral decision-making differences and medium effect sizes for all MDM scores.

Tags: ToM, social decision-making, behavioral

Altered Prefrontal Theta and Gamma Activity during an Emotional Face Processing Task in Parkinson Disease.

Item Type Journal Article

Author Witney Chen
Author Coralie de Hemptinne
Author Michael Leibbrand
Author Andrew M. Miller
Author Paul S. Larson
Author Philip A. Starr

Abstract Patients with Parkinson disease (PD) often experience nonmotor symptoms including cognitive deficits, depression, and anxiety. Cognitive and affective processes are thought to be mediated by prefrontal cortico-basal ganglia circuitry. However, the topography and neurophysiology of prefrontal cortical activity during complex tasks are not well characterized. We used high-resolution electrocorticography in pFC of patients with PD and essential tremor, during implantation of deep brain stimulator leads in the awake state, to understand disease-specific changes in prefrontal activity during an emotional face processing task. We found that patients with PD had less task-related theta-alpha power and greater task-related gamma power in the dorsolateral pFC, inferior frontal cortex, and lateral OFC. These findings support a model of prefrontal neurophysiological changes in the dopamine-depleted state, in which focal areas of hyperactivity in prefrontal cortical regions may compensate for impaired long-range interactions mediated by low-frequency rhythms. These distinct neurophysiological changes suggest that nonmotor circuits undergo characteristic changes in PD.

Date 2019 Nov
Language eng
Extra Place: United States
Volume 31
Pages 1768-1776
Publication Journal of cognitive neuroscience
DOI 10.1162/jocn_a_01450
Issue 11
Journal Abbr J Cogn Neurosci
ISSN 1530-8898 0898-929X
PMID 31322465

Date Added 6.7.2025, 19:09:39
Modified 5.9.2025, 14:30:32

Notes:

Not Included: no corrected Proof. hence, Not an empirical study
Tags: EXCLUDED

Altered Theory of Mind in Parkinson's Disease and Impact on Caregivers: A Pilot Study.

Item Type Journal Article
Author Ariane Giguère-Rancourt
Author Marika Plourde
Author Eva Racine
Author Marianne Couture
Author Mélanie Langlois
Author Nicolas Dupré
Author Martine Simard
Abstract Mild cognitive impairment (MCI) in Parkinson's disease (PD) includes deficits in theory of mind (ToM). However, associations between ToM and caregiver burden and distress are still unclear. The objective of this pilot study was to preliminarily explore the relation between ToM and caregiver burden and distress in a sample of PD-MCI patients. Twelve PD-MCI patients were evaluated on a ToM task (Faux Pas), whereas their caregivers were assessed on caregiver burden (Zarit Burden Interview-12 items) and distress (Neuropsychiatric Inventory-Distress). Cognitive ToM was significantly associated with caregiver distress, but caregiver burden was associated with the severity of patient psychiatric symptoms.
Date 2022 May
Language eng
Extra Place: England
Volume 49
Pages 437-440
Publication The Canadian journal of neurological sciences. Le journal canadien des sciences neurologiques
DOI 10.1017/cjn.2021.110
Issue 3
Journal Abbr Can J Neurol Sci
ISSN 0317-1671
PMID 33988099
Date Added 6.7.2025, 19:09:40
Modified 5.9.2025, 14:36:41

Notes:

Not Included: not an empirical study (brief communication)
Tags: EXCLUDED

Altruistic Punishment and Impulsivity in Parkinson's Disease: A Social Neuroscience Perspective

**Item Type**	Journal Article
**Author**	Rosalba Morese
**Author**	Sara Palermo
**Abstract**	Non-motor symptoms of Parkinson's disease (PD) are of increasing interest in clinical and psychological research. Disinhibition-the inability to inhibit inappropriate behavior-leads to social and emotional impairments, including impulsive behavior and disregard for social conventions and decision-making behavior. In recent years, the latter has been investigated using economic exchanges during social interactions. Altruistic punishment-to punish someone who violates group norms even if it foresees a personal cost-is one of the most useful and fruitful paradigms; it allows to maintain a cooperation system within social groups. Alterations of this cognitive ability negatively impact the quality of life of the individual and social stability. Social neuroscience has suggested association between impulsive behaviors and altruistic punishment. Neuroimaging research aimed at exploring functional networks and intrinsic functional connectivity went in this direction. To date, little is known about these issues in neurodegenerative diseases such as PD. Dopamine replacement treatment and dopamine-agonists have been associated with impulse-control disorder and impulsive-compulsive behavior able to affect social decision-making. Frontal-executive dysfunction determines an alteration of social functioning through a mechanism of subversion of online action-monitoring, which associates disinhibition with volition. Genetic polymorphisms, alterations of the nigro-striatal substance, and impairment in the medial prefrontal cortex and in the Default mode network (DMN) seem to be able to explain these mechanisms. This theoretical perspective article aims to present these topics in order to encourage an interdisciplinary discussion capable of generating new research and developing rehabilitative intervention to improve social decision-making in PD patients.
**Date**	2020-07-21
**Language**	English
**Extra**	Place: AVENUE DU TRIBUNAL FEDERAL 34, LAUSANNE, CH-1015, SWITZERLAND Type: Article
**Volume**	14
**Publisher**	FRONTIERS MEDIA SA
**Publication**	FRONTIERS IN BEHAVIORAL NEUROSCIENCE
**DOI**	10.3389/fnbeh.2020.00102
**ISSN**	1662-5153
**Date Added**	14.7.2025, 14:50:31
**Modified**	5.9.2025, 14:47:31

Notes:

Not Included: No study (Theoretical perspective article)
Tags: EXCLUDED

Amygdala activation modulated by levodopa during emotional recognition processing in healthy volunteers: a double-blind, placebo-controlled study.

Item Type Journal Article
Author Pauline Delaveau
Author Pilar Salgado-Pineda
Author Joëlle Micallef-Roll
Author Olivier Blin
Abstract A critical role of dopaminergic systems in emotional processing has been revealed by several animal and clinical studies in Parkinson disease and schizophrenia. We conducted a study with functional magnetic resonance imaging (fMRI) in 13 healthy volunteers to test the dopaminergic modulation on amygdala response to emotional processing and to evaluate if it was the result of a direct action on amygdalar nuclei or indirect modulation via medial prefrontal cortex projecting on amygdala.A placebo-controlled crossover experimental design was used. Subjects received either levodopa (100 mg) or placebo in 2 fMRI sessions. Amygdala activation was evaluated during a facial emotion recognition test.The statistical comparison between placebo versus levodopa situations revealed a significant reduction in activation of right amygdala during the levodopa fMRI session. The functional connectivity analysis revealed only a change of correlated activations between right and left amygdala, and not medial prefrontal cortex, after levodopa administration. Our results suggest that administration of levodopa to healthy volunteers impairs the amygdalar activation. It supports the hypothesis that amygdala activation follows an inverted U-shaped curve in relation to dopamine (DA) concentration. The results of the functional connectivity seem to suggest a dopaminergic action on amygdalar nuclei rather than a modulation of medial prefrontal cortex on amygdala.
Date 2007 Dec
Language eng
Extra Place: United States
Volume 27
Pages 692-697
Publication Journal of clinical psychopharmacology
DOI 10.1097/jcp.0b013e31815a444d
Issue 6
Journal Abbr J Clin Psychopharmacol
ISSN 0271-0749
PMID 18004139
Date Added 6.7.2025, 19:09:42
Modified 5.9.2025, 14:32:59

Notes:

Not Included: not on PD
Tags: EXCLUDED

Amygdala and emotionality in Parkinson's disease: An integrative review of the neuropsychological evidence

**Item Type**	Journal Article
**Author**	Radek Trnka
**Author**	Jozef Hasto
**Author**	Inna Cabelkova
**Author**	Martin Kuska
**Author**	Peter Tavel
**Author**	Tomas Nikolai
**Abstract**	Parkinson's disease (PD) is often accompanied by significant changes in emotionality, such as apathy, anhedonia, anxiety and depression. The present review summarizes the empirical evidence, including amygdala changes and psychological changes in emotionality in people suffering from PD. Seventeen empirical full-text articles including research on both amygdala and emotionality in PD were reviewed. The changes in amygdala volumes as well as changes in binding potentials, functional connectivity, regional homogeneity and regional cerebral blood flow were found to have various impacts on emotionality in people with PD. The integration of the results showed that some effects of amygdala changes on emotionality were lateralized. Some of the reviewed studies indicated that the volume loss in the left amygdala was found to be related to increased anxiety, whereas bilateral volume loss in amygdala was linked to increased depressivity. The reviewed results also support a hypothesis of bradylimbic affective disturbance in patients with PD. The disturbed activation of amygdala accompanying the evaluation of negative facial expressions implies that the evaluation of the content of affective stimuli in terms of their affective meanings is disturbed in PD patients. Impaired evaluation of affective attributes given by amygdala-based translational deficits is likely to be related to problems in translating the results of cognitive appraisal into somatomotor, arousal and other changes. This mechanism is suggested to be responsible for apathy as well as for other changes in emotionality accompanying PD.
**Date**	2018
**Language**	English
**Extra**	Place: MAGHIRA & MAAS S A R L, 6C, RUE GABRIEL LIPPMANN, L-5365 MUNSBACH, LUXEMBOURG Type: Review
**Volume**	39
**Publisher**	MAGHIRA & MAAS PUBLICATIONS
**Pages**	105-110
**Publication**	NEUROENDOCRINOLOGY LETTERS
**Issue**	2
**ISSN**	0172-780X
**Date Added**	14.7.2025, 14:50:35
**Modified**	5.9.2025, 14:58:50

Notes:

Not Included: not a systematic review
Tags: EXCLUDED

An ERP study of vocal emotion processing in asymmetric Parkinson's disease

**Item Type**	Journal Article
**Author**	Patricia Garrido-Vasquez
**Author**	Marc D. Pell
**Author**	Silke Paulmann
**Author**	Karl Strecker
**Author**	Johannes Schwarz
**Author**	Sonja A. Kotz
**Abstract**	Parkinson's disease (PD) has been related to impaired processing of emotional speech intonation (emotional prosody). One distinctive feature of idiopathic PD is motor symptom asymmetry, with striatal dysfunction being strongest in the hemisphere contralateral to the most affected body side. It is still unclear whether this asymmetry may affect vocal emotion perception. Here, we tested 22 PD patients (10 with predominantly left-sided [LPD] and 12 with predominantly right-sided motor symptoms) and 22 healthy controls in an event-related potential study. Sentences conveying different emotional intonations were presented in lexical and pseudo-speech versions. Task varied between an explicit and an implicit instruction. Of specific interest was emotional salience detection from prosody, reflected in the P200 component. We predicted that patients with predominantly right-striatal dysfunction (LPD) would exhibit P200 alterations. Our results support this assumption. LPD patients showed enhanced P200 amplitudes, and specific deficits were observed for disgust prosody, explicit anger processing and implicit processing of happy prosody. Lexical speech was predominantly affected while the processing of pseudo-speech was largely intact. P200 amplitude in patients correlated significantly with left motor scores and asymmetry indices. The data suggest that emotional salience detection from prosody is affected by asymmetric neuronal degeneration in PD.
**Date**	2013-12
**Language**	English
**Extra**	Place: GREAT CLARENDON ST, OXFORD OX2 6DP, ENGLAND Type: Article
**Volume**	8
**Publisher**	OXFORD UNIV PRESS
**Pages**	918-927
**Publication**	SOCIAL COGNITIVE AND AFFECTIVE NEUROSCIENCE
**DOI**	10.1093/scan/nss094
**Issue**	8
**ISSN**	1749-5016
**Date Added**	14.7.2025, 14:50:38
**Modified**	5.9.2025, 14:36:17

Notes:

Included

sample characteristics

size: 22 PD and 22 HC (matched the patients for age, sex and education)

Parkinson's Disease type and duration: Ten exhibited rather left-lateralized (LPD) and 12 rather right-lateralized (RPD) motor symptoms according UPDRS. Mduration = 5.52 SD=3.91 (1-15)

Medication: on medication

Hoehn-Yahr: M=2.23 SD=0.65 (1-4)

UPDRS-3: M=14.36 SD=4.70 (6-21)

Gender (male): 11 males (50%)

averaged ages (SD, range): M=66.05 SD=8.06 (44-80)

other neurological disease (tumor, stroke, etc.): None

other major psychopathology: None

origin country (or ethnicity): Germany

method (Review, meta-analysis or observational and/or self-reported):

instruments used in order to quantify the variables

Social cognition aspect: emotion recognition

Name of the task: NA

type of stimulus [face/voice etc., Ekman faces/other etc.]: auditorily presented sentences. Half were presented in lexical German speech and half in unintelligible pseudo-speech matching German phonotactic rules. All sentences had the same syntactic structure and corresponded to one of four emotional intonations. For lexical sentences, semantics matched emotional prosody. Sentences were spoken by a trained male or female speaker, were of approximately 3 s duration, normalized and digitized at a 16-bit/44.1 kHz sampling rate. The material has been successfully used in prior studies.

task condition: angry, disgusted, fearful or happy) or a neutral baseline

operationalization: the question (‘emotional or not emotional?’[ET]/‘German or not German?’[IT]) appeared on the screen for 300 ms. >> accuracy

Main findings related to the review's scope

LPD patients’ performance was significantly below that of HC, while the other group comparisons were not significant

Tags: emotion recognition, behavioral

An fMRI study into emotional processing in Parkinson's disease: Does increased medial prefrontal activation compensate for striatal dysfunction?

Item Type Journal Article
Author Anja J. H. Moonen
Author Peter H. Weiss
Author Michael Wiesing
Author Ralph Weidner
Author Gereon R. Fink
Author Jennifer S. A. M. Reijnders
Author Wim M. Weber
Author Albert F. G. Leentjens
Abstract Background Apart from a progressive decline of motor functions, Parkinson's disease (PD) is also characterized by non-motor symptoms, including disturbed processing of emotions. This study aims at assessing emotional processing and its neurobiological correlates in PD with the focus on how medicated Parkinson patients may achieve normal emotional responsiveness despite basal ganglia dysfunction. Methods Nineteen medicated patients with mild to moderate PD (without dementia or depression) and 19 matched healthy controls passively viewed positive, negative, and neutral pictures in an event-related blood oxygen level-dependent functional magnetic resonance imaging study (BOLD-fMRI). Individual subjective ratings of valence and arousal levels for these pictures were obtained right after the scanning. Results Parkinson patients showed similar valence and arousal ratings as controls, denoting intact emotional processing at the behavioral level. Yet, Parkinson patients showed decreased bilateral putaminal activation and increased activation in the right dorsomedial prefrontal cortex (PFC), compared to controls, both most pronounced for highly arousing emotional stimuli. Conclusions Our findings revealed for the first time a possible compensatory neural mechanism in Parkinson patients during emotional processing. The increased medial PFC activity may have modulated emotional responsiveness in patients via top-down cognitive control, therewith restoring emotional processing at the behavioral level, despite striatal dysfunction. These results may impact upon current treatment strategies of affective disorders in PD as patients may benefit from this intact or even compensatory influence of prefrontal areas when therapeutic strategies are applied that rely on cognitive control to modulate disturbed processing of emotions.
Date 2017-05-09
Language English
Extra Place: 1160 BATTERY STREET, STE 100, SAN FRANCISCO, CA 94111 USA
Type: Article
Volume 12
Publisher PUBLIC LIBRARY SCIENCE
Publication PLOS ONE
DOI 10.1371/journal.pone.0177085
Issue 5
ISSN 1932-6203
Date Added 14.7.2025, 14:50:35
Modified 14.7.2025, 14:50:35

Notes:

not included: not on SC
Tags: EXCLUDED

Analysis of facial expressions in parkinson's disease through video-based automatic methods

**Item Type**	Journal Article
**Author**	Andrea Bandini
**Author**	Silvia Orlandi
**Author**	Hugo Jair Escalante
**Author**	Fabio Giovannelli
**Author**	Massimo Cincotta
**Author**	Carlos A. Reyes-Garcia
**Author**	Paola Vanni
**Author**	Gaetano Zaccara
**Author**	Claudia Manfredi
**Abstract**	Background: The automatic analysis of facial expressions is an evolving field that finds several clinical applications. One of these applications is the study of facial bradykinesia in Parkinson's disease (PD), which is a major motor sign of this neurodegenerative illness. Facial bradykinesia consists in the reduction/loss of facial movements and emotional facial expressions called hypomimia. New method: In this work we propose an automatic method for studying facial expressions in PD patients relying on video-based Methods: 17 Parkinsonian patients and 17 healthy control subjects were asked to show basic facial expressions, upon request of the clinician and after the imitation of a visual cue on a screen. Through an existing face tracker, the Euclidean distance of the facial model from a neutral baseline was computed in order to quantify the changes in facial expressivity during the tasks. Moreover, an automatic facial expressions recognition algorithm was trained in order to study how PD expressions differed from the standard expressions. Results: Results show that control subjects reported on average higher distances than PD patients along the tasks. Comparison with existing methods: This confirms that control subjects show larger movements during both posed and imitated facial expressions. Moreover, our results demonstrate that anger and disgust are the two most impaired expressionS in PD patients. Conclusions: Contactless video-based systems can be important techniques for analyzing facial expressions also in rehabilitation, in particular speech therapy, where patients could get a definite advantage from a real-time feedback about the proper facial expressions/movements to perform. (C) 2017 Elsevier B.V. All rights reserved.
**Date**	2017-04-01
**Language**	English
**Extra**	Place: PO BOX 211, 1000 AE AMSTERDAM, NETHERLANDS Type: Article
**Volume**	281
**Publisher**	ELSEVIER SCIENCE BV
**Pages**	7-20
**Publication**	JOURNAL OF NEUROSCIENCE METHODS
**DOI**	10.1016/j.jneumeth.2017.02.006

ISSN 0165-0270
Date Added 14.7.2025, 14:50:35
Modified 5.9.2025, 14:27:36

Notes:

Not Included: not on SC
Tags: EXCLUDED

Anatomical and neurochemical bases of theory of mind in de novo Parkinson's Disease.

Item Type Journal Article
Author Beatrice Orso
Author Dario Arnaldi
Author Francesco Famà
Author Nicola Girtler
Author Andrea Brugnolo
Author Elisa Doglione
Author Laura Filippi
Author Federico Massa
Author Enrico Peira
Author Matteo Bauckneht
Author Silvia Morbelli
Author Flavio Nobili
Author Matteo Pardini
Abstract Theory of mind (ToM) deficit is a frequent finding in subjects with neurological and psychiatric conditions. While a number of brain regions play a role in ToM, to date the contribution of the diffuse projection systems is less understood. Here, we explored the topographical and neurochemical bases of ToM using multi-tracer molecular imaging and quantitative electroencephalography (qEEG) in a group of 30 drug-naïve, de novo Parkinson's Disease (PD) patients (mean age 73.39 ± 8.93 years, 11 females). ToM was assessed using the "Reading the Mind in the Eyes Task" (RMET), while general cognition with the MMSE. We acquired FDG-PET images (as a marker of regional neurodegeneration), I-123 Ioflupane Single Photon Emission Computed Tomography (123 I-FP-CIT-SPECT, as a marker of dopaminergic impairment in the basal ganglia and in the cortex and as a proxy marker of serotoninergic deafferentation in the thalamus), and qEEG recordings (using the Theta/Alpha power ratio as marker of cholinergic deafferentation). PD presented with a significantly worse RMET score compared to 60 controls (20.7 ± 5.5 vs 27.5 ± 3.0 p = .001) while there was no difference between the two groups in age, education or MMSE. The voxel-wise analysis of total RMET score and regional metabolism showed a positive correlation in the superior temporal gyrus and in the insula. Among the proxy markers of dopaminergic degeneration, serotoninergic and cholinergic deafferentation, ToM presented only an inverse correlation with 123 I-FP-CIT thalamic specific binding ratio (SBR) values -a proxy serotoninergic marker-which remained significant after correction for FDG metabolism in the areas associated with

ToM. On the other hand, MMSE only correlated with qEEG posterior Theta/Alpha power. These findings point to the presence of a specific cortical and neurochemical signature of ToM in PD, to the independence of ToM from general cognition, and suggest possible therapeutic targets to treat social cognition deficits.

Date 2020 Sep
Language eng
License Copyright © 2020 Elsevier Ltd. All rights reserved.
Extra Place: Italy
Volume 130
Pages 401-412
Publication Cortex; a journal devoted to the study of the nervous system and behavior
DOI 10.1016/j.cortex.2020.06.012
Journal Abbr Cortex
ISSN 1973-8102 0010-9452
PMID 32755727
Date Added 6.7.2025, 19:09:40
Modified 5.9.2025, 14:48:17

Notes:

Included

Sample characteristics

Size: 30 PD, 60 HC

PD-type: NA

PD-duration: NA

Medication: OFF state

Hoehn-Yahr: NA

UPDRS-3: M = 20.65 ± 7.6

Gender (male): 19 (63%)

Age: M = : 73.39 SD= 8.93 years; range: 50-81

Other neurological disease (tumor, stroke, etc.): None

Other major psychopathology: None

Origin country (or ethnicity): Italy

method observational

instruments used in order to quantify the variables

Social cognition aspect: ToM

Name of the task: Reading the mind in the eyes task (RMET)

Type of stimulus [face/voice etc., Ekman faces/other etc.]: 36 black and white picture of the eye region

Task condition: The subject has to recognize the emotional state represented in the picture and choose one among four given words.

Operationalization: Each item is scored 1 if the answer is correct and 0 if the answer is wrong (range 0-36). For each patients were calculated both a total score (number of correct responses) and separate subscores for positive, negative and neutral stimuli.

FDG-PET:

Images were acquired by means of a SIEMENS Biograph 16 PET/CT equipment with a total axial field of view of 15 cm and no interplane gap space.

I-FP-CIT SPECT data was acquired by means of a 2-headed Millennium VG camera. Total counts ranged between 2.0 and 2.5 million. The pixel size of the acquisition matrix was 2.4 mm.

Taking into account the frequent asymmetric clinical and biological presentation of PD, 11 of the FDG-PET and ^{123}I-FP-CIT SPECT images were flipped based on the side of the more affected limbs, to have the more affected hemisphere on the right-hand-side of each image. Thus, the right hemisphere in the images represent the More Affected Hemisphere (MAH), and the left hemisphere in the images represent the Less Affected Hemisphere (LAH).

FDG-PET images were subjected to affine and nonlinear spatial normalization into Talairach and Tournoux space using SPM12

All the default choices of SPM were followed with the exception of spatial normalization. For this study, the $H_2{}^{15}O$ SPM-default [redacted] was replaced by an optimized brain FDG-PET template as described by Della Rosa and colleagues (Della Rosa et al., 2014). The spatially normalized set of images was then smoothed with a 10-mm isotropic Gaussian filter to blur individual variations in gyral anatomy and to increase the signal-to-noise ratio. A whole-brain voxel-wise correlation between local FDG uptake and total RMET score was performed in the PD group using a height threshold $p < .001$ (uncorrected) and a minimum cluster size of 100 voxels for significant clusters. At cluster level, the accepted threshold of statistical significance was $p < .05$, family-wise error corrected for multiple comparisons. The FDG signal in the regions found to correlate significantly with RMET was then normalized over the whole brain and used as a covariate in the following analyses of cortical RMET-related volume of interest (VOI).

Main findings related to the review's scope

ToM

PD patients presented with a significantly worse RMET score compared to controls (20.7 ± 5.5 vs 27.5 ± 3.0 p = .001, t = 7.6) while there was no difference between the two groups in age, education or MMSE score.

Imaging - analyzed data included the PD group alone.

FDG-PET: ToM and brain metabolism

The voxel-wise correlation between total RMET score and regional metabolism showed a cluster of significant positive correlation focused in the superior temporal gyrus in the LAH and one focused in the insula in the MAH.

3I-FP-CIT-SPECT: ToM and subcortical serotonergic function

We observed a significant negative correlation between total RMET score and the LAH thalamus SBR (p = .004, r ¼=.620) while there was no correlation between the total RMET score and the MAH thalamus SBR (p = .917, r = .026).

I-FP-CIT-SPECT: ToM and basal ganglia dopaminergic function

we found no correlation between RMET score and SBR in the putamen and caudate nuclei.

I-FP-CIT-SPECT: ToM and dopaminergic function in the cortical RMET-related VOI

There was no significant correlation between SBR of the cortical RMET-related VOI and ToM performance

Tags: ToM, Imaging, behavioral

Anodal Transcranial Direct Current Stimulation over the Cerebellum Enhances Sadness Recognition in Parkinson's Disease Patients: a Pilot Study.

Item Type Journal Article

Author Fabiana Ruggiero

Author Michelangelo Dini
Author Francesca Cortese
Author Maurizio Vergari
Author Martina Nigro
Author Barbara Poletti
Author Alberto Priori
Author Roberta Ferrucci

Abstract Emotional processing impairments, resulting in a difficulty to decode emotions from faces especially for negative emotions, are characteristic non-motor features of Parkinson's disease (PD). There is limited evidence about the specific contribution of the cerebellum to the recognition of emotional contents in facial expressions even though patients with cerebellar dysfunction often lose this ability. In this study, we aimed to evaluate whether the recognition of facial expressions can be modulated by cerebellar transcranial direct current stimulation (tDCS) in PD patients. Nine PD patients were enrolled and received anodal and sham tDCS (2 mA, 20 min), for 5 consecutive days, in two separate cycles at intervals of at least 1 month. The facial emotion recognition task was administered at baseline (T0) and after cerebellar tDCS on day 5 (T1). Our preliminary study showed that anodal cerebellar tDCS significantly enhanced emotional recognition in response to sad facial expressions by about 16%, but left recognition of anger, happiness, and neutral facial expressions unchanged. Despite the small sample size, our preliminary results show that anodal tDCS applied for five consecutive days over the cerebellum modulates the way PD patients recognize specific facial expressions, thus suggesting that the cerebellum plays a crucial role in recognition of negative emotions and corroborating previous knowledge on the link between social cognition and the cerebellum.

Date 2022 Apr
Language eng
License © 2021. The Author(s).
Extra Place: United States
Volume 21
Pages 234-243
Publication Cerebellum (London, England)
DOI 10.1007/s12311-021-01295-y
Issue 2
Journal Abbr Cerebellum
ISSN 1473-4230 1473-4222
PMID 34159563
PMCID PMC8993778
Date Added 6.7.2025, 19:09:38
Modified 5.9.2025, 14:54:29

Notes:

Not Included: Pre-Post Anodal Transcranial Direct Current Stimulation on PD. No HC or other control group

Tags: EXCLUDED

Another Advanced Test of Theory of Mind: Evidence from Very High Functioning Adults with Autism or Asperger Syndrome

**Item Type**	Journal Article
**Author**	Simon Baron-Cohen
**Author**	Therese Jolliffe
**Author**	Catherine Mortimore
**Author**	Mary Robertson
**Abstract**	Previous studies have found a subgroup of people with autism or Asperger Syndrome who pass second-order tests of theory of mind. However, such tests have a ceiling in developmental terms corresponding to a mental age of about 6 years. It is therefore impossible to say if such individuals are intact or impaired in their theory of mind skills. We report the performance of very high functioning adults with autism or Asperger Syndrome on an adult test of theory of mind ability. The task involved inferring the mental state of a person just from the information in photographs of a person's eyes. Relative to age-matched normal controls and a clinical control group (adults with Tourette Syndrome), the group with autism and Asperger Syndrome were significantly impaired on this task. The autism and Asperger Syndrome sample was also impaired on Happe's strange stories tasks. In contrast, they were unimpaired on two control tasks: recognising gender from the eye region of the face, and recognising basic emotions from the whole face. This provides evidence for subtle mindreading deficits in very high functioning individuals on the autistic continuum.
**Date**	10/1997
**Language**	en
**Short Title**	Another Advanced Test of Theory of Mind
**Library Catalog**	DOI.org (Crossref)
**URL**	https://acamh.onlinelibrary.wiley.com/doi/10.1111/j.1469-7610.1997.tb01599.x
**Accessed**	19.1.2026, 7:31:33
**License**	http://onlinelibrary.wiley.com/termsAndConditions#vor
**Volume**	38
**Pages**	813-822
**Publication**	Journal of Child Psychology and Psychiatry
**DOI**	10.1111/j.1469-7610.1997.tb01599.x
**Issue**	7
**Journal Abbr**	Child Psychology Psychiatry
**ISSN**	0021-9630, 1469-7610
**Date Added**	19.1.2026, 7:31:33
**Modified**	19.1.2026, 7:31:33

Apathy and impaired emotional facial recognition networks overlap in Parkinson's disease: a PET study with conjunction analyses.

**Item Type**	Journal Article
**Author**	Gabriel Robert

Author Florence Le Jeune
Author Thibault Dondaine
Author Sophie Drapier
Author Julie Péron
Author Clément Lozachmeur
Author Paul Sauleau
Author Jean-François Houvenaghel
Author David Travers
Author Bruno Millet
Author Marc Vérin
Author Dominique Drapier
Abstract Apathy is a disabling non-motor symptom that is frequently observed in Parkinson's disease (PD). Its description and physiopathology suggest that it is partially mediated by emotional impairment, but this research issue has never been addressed at a clinical and metabolic level. We therefore conducted a metabolic study using (18)fluorodeoxyglucose positron emission tomography ((18)FDG PET) in 36 PD patients without depression and dementia. Apathy was assessed on the Apathy Evaluation Scale (AES), and emotional facial recognition (EFR) performances (ie, percentage of correct responses) were calculated for each patient. Confounding factors such as age, antiparkinsonian and antidepressant medication, global cognitive functions and depressive symptoms were controlled for. We found a significant negative correlation between AES scores and performances on the EFR task. The apathy network was characterised by increased metabolism within the left posterior cingulate (PC) cortex (Brodmann area (BA) 31). The impaired EFR network was characterised by decreased metabolism within the bilateral PC gyrus (BA 31), right superior frontal gyrus (BAs 10, 9 and 6) and left superior frontal gyrus (BA 10 and 11). By applying conjunction analyses to both networks, we identified the right premotor cortex (BA 6), right orbitofrontal cortex (BA 10), left middle frontal gyrus (BA 8) and left posterior cingulate gyrus (BA 31) as the structures supporting the association between apathy and impaired EFR. These results confirm that apathy in PD is partially mediated by impaired EFR, opening up new prospects for alleviating apathy in PD, such as emotional rehabilitation.
Date 2014 Oct
Language eng
License Published by the BMJ Publishing Group Limited. For permission to use (where not already granted under a licence) please go to http://group.bmj.com/group/rights-licensing/permissions.
Extra Place: England
Volume 85
Pages 1153-1158
Publication Journal of neurology, neurosurgery, and psychiatry
DOI 10.1136/jnnp-2013-307025
Issue 10
Journal Abbr J Neurol Neurosurg Psychiatry
ISSN 1468-330X 0022-3050
PMID 24403280
Date Added 6.7.2025, 19:09:37
Modified 5.9.2025, 14:53:05

Notes:

Included

Sample characteristics

Size: 36 PD

PD-type: Idiopathic PD

PD-duration: NA

Medication: Assessment in ON

Hoehn-Yahr: NA

UPDRS-3: M = 7.9, Sd = 5.4, Range = 0-23

Gender (male): NA

Age: M = 58.6, SD = 7.3, Range = 45-74

Other neurological disease (tumor, stroke, etc.): none

Other major psychopathology: Eight patients (22%) were on antidepressant medication.

Origin country (or ethnicity): France

method behavioural

instruments used in order to quantify the variables

Social cognition aspect: Emotional facial recognition (EFR)

Name of the task: NA

Type of stimulus [face/voice etc., Ekman faces/other etc.]: 55 photographs of seven different facial expressions (happiness, sadness, fear, surprise, disgust, anger and no emotion)

Task condition: patients were prompted to give an answer by choosing the most suitable response from the list of seven emotions.

Operationalization: We calculated the percentage of correct emotion identification for each patient and this for each discrete emotion and across all emotions (ie, overall score).

PET-imaging procedure:

PET measurements were performed using a dedicated Discovery ST PET/CT scanner (General Electric Medical System, Milwaukee, WI) in 2D mode. A 222–296 MBq injection of 18F-FDG was administered intravenously. A 20 min 2D scan was performed 30 min postinjection, with participants positioned at the centre of the field of view. X-ray CT-based attenuation correction was performed prior to the emission scan. Following scatter, dead time and random corrections, the PET images were reconstructed by means of 2D filtered backprojection, yielding 47 contiguous transaxial 3.75 mm thick slices

Main findings related to the review's scope

Impaired EFR metabolic network

impaired EFR scores (ie, low scores on the EFR task) were correlated with decreased metabolism within the precuneus and the inferior occipital gyrus.

Impaired EFR was correlated with increased metabolism within an extended limbic network with bilateral posterior cingulate and bilateral superior frontal gyri. Talairach coordinates, BAs, cluster sizes and z scores are provided in table 3.

Decreased metabolism network correlated with poor performances on the EFR task (ie, low overall EFR score) is displayed in figure 1.

Overlap between the two networks highlighted by the conjunction analyses

We found bilateral frontal gyri, right premotor cortex and left posterior cingulate are the structures both involved in the apathy and the impaired EFR networks. Talairach coordinates, BAs, cluster sizes and z scores are provided in table 4

Table 3 Impaired emotional facial recognition (EFR) metabolic networks: positive and negative correlations with low EFR scores (ie, poor recognition abilities)

Metabolism	Anatomical structure	BA	X	Y	Z	Cluster size	*z* score
Positive correlations (decreased metabolism)							
	Right precuneus	7	22	−58	44	43	3.13
	Left inferior occipital gyrus	17	−20	−90	−6	60	3.06
Negative correlations (increased metabolism)							
	Bilateral posterior cingulate cortex	31	4	−42	42	918	3.92
	Right superior frontal gyrus	10	20	54	22	1275	3.73
		9	38	54	22		
		6	24	30	56		
	Left superior frontal gyrus	11	−16	52	−12	141	3.2
	Left superior frontal gyrus	10	−16	52	26	171	3.06

BA, Brodmann area.

Tags: Emotion recognition, Imaging

Apathy and impaired recognition of emotion: are they related in Parkinson's disease?

**Item Type**	Journal Article
**Author**	Kathy Dujardin
**Author**	Renaud Lopes
**Date**	2014 Oct
**Language**	eng
**Extra**	Place: England
**Volume**	85
**Pages**	1061
**Publication**	Journal of neurology, neurosurgery, and psychiatry
**DOI**	10.1136/jnnp-2013-307224
**Issue**	10
**Journal Abbr**	J Neurol Neurosurg Psychiatry
**ISSN**	1468-330X 0022-3050
**PMID**	24403281
**Date Added**	6.7.2025, 19:09:34
**Modified**	5.9.2025, 14:34:37

Notes:

Not Included: Editorial commentary

Tags: EXCLUDED

Apathy Associated With Impaired Recognition of Happy Facial Expressions in Huntington's Disease

**Item Type**	Journal Article
**Author**	Katherine Osborne-Crowley
**Author**	Sophie C. Andrews
**Author**	Izelle Labuschagne
**Author**	Akshay Nair
**Author**	Rachael Scahill
**Author**	David Craufurd
**Author**	Sarah J. Tabrizi
**Author**	Julie C. Stout
**Author**	C. Campbell
**Author**	M. Campbell
**Author**	E. Frajman
**Author**	C. Milchman
**Author**	A. O'Regan
**Author**	A. Coleman

Author R. Dar Santos
Author J. Decolongon
Author A. Sturrock
Author E. Bardinet
Author C. Jauffret
Author D. Justo
Author S. Lehericy
Author C. Marelli
Author K. Nigaud
Author P. Pourchot
Author R. Valabregue
Author N. Bechtel
Author S. Bohlen
Author R. Reilmann
Author A. Hoffman
Author P. Kraus
Author B. Landwehrmeyer
Author S. J. A. van den Bogaard
Author E. M. Dumas
Author J. van der Grond
Author E. P. t'Hart
Author C. Jurgens
Author M. -N. Witjes-Ane
Author N. Arran
Author J. Callaghan
Author C. Stopford
Author C. Frost
Author R. Jones
Author C. Berna
Author H. Crawford
Author N. Fox
Author C. Gibbard
Author N. Hobbs
Author N. Lahiri
Author I. Malone
Author R. Ordidge
Author G. Owen
Author A. Patel
Author T. Pepple
Author J. Read
Author M. Say
Author R. Scahill
Author D. Whitehead
Author E. Wild
Author S. Keenan

Author D. M. Cash
Author S. Hicks
Author C. Kennard
Author T. Acharya
Author E. Axelson
Author H. Johnson
Author D. Langbehn
Author C. Wang
Author S. Lee
Author W. Monaco
Author H. Rosas
Author C. Campbell
Author S. Queller
Author K. Whitlock
Author B. Borowsky
Author A. Tobin
Author TRACK-HD Investigators
Abstract Objectives: Previous research has demonstrated an association between emotion recognition and apathy in several neurological conditions involving fronto-striatal pathology, including Parkinson's disease and brain injury. In line with these findings, we aimed to determine whether apathetic participants with early Huntington's disease (HD) were more impaired on an emotion recognition task compared to non-apathetic participants and healthy controls. Methods: We included 43 participants from the TRACK-HD study who reported apathy on the Problem Behaviours Assessment - short version (PBA-S), 67 participants who reported no apathy, and 107 controls matched for age, sex, and level of education. During their baseline TRACK-HD visit, participants completed a battery of cognitive and psychological tests including an emotion recognition task, the Hospital Depression and Anxiety Scale (HADS) and were assessed on the PBA-S. Results: Compared to the non-apathetic group and the control group, the apathetic group were impaired on the recognition of happy facial expressions, after controlling for depression symptomology on the HADS and general disease progression (Unified Huntington's Disease Rating Scale total motor score). This was despite no difference between the apathetic and non-apathetic group on overall cognitive functioning assessed by a cognitive composite score. Conclusions: Impairment of the recognition of happy expressions may be part of the clinical picture of apathy in HD. While shared reliance on frontostriatal pathways may broadly explain associations between emotion recognition and apathy found across several patient groups, further work is needed to determine what relationships exist between recognition of specific emotions, distinct subtypes of apathy and underlying neuropathology. (JINS, 2019, 25, 453-461)
Date 2019-05
Language English
Extra Place: 32 AVENUE OF THE AMERICAS, NEW YORK, NY 10013-2473 USA
Type: Article
Volume 25
Publisher CAMBRIDGE UNIV PRESS
Pages 453-461
Publication JOURNAL OF THE INTERNATIONAL NEUROPSYCHOLOGICAL SOCIETY
DOI 10.1017/S1355617718001224

Issue 5
ISSN 1355-6177
Date Added 14.7.2025, 14:50:33
Modified 5.9.2025, 14:48:22

Notes:

Not Included: Not of PD

Tags: EXCLUDED

Are dopaminergic pathways involved in theory of mind? A study in Parkinson's disease.

Item Type Journal Article

Author Julie Péron
Author Siobhan Vicente
Author Emmanuelle Leray
Author Sophie Drapier
Author Dominique Drapier
Author Renaud Cohen
Author Isabelle Biseul
Author Tiphaine Rouaud
Author Florence Le Jeune
Author Paul Sauleau
Author Marc Vérin

Abstract The "orbitofrontal" and "cingulate" frontostriatal loops and the mesolimbic dopaminergic system that modulates their function have been implicated in theory of mind (ToM). Parkinson's disease (PD) provides a model for assessing their role in humans. Results of the handful of previous studies of ToM in PD providing preliminary evidence of impairment remain controversial, mainly because the patients included in these studies were not accurately described, making it difficult to determine whether their ToM deficits were due to general cognitive deterioration or to a more specific dopaminergic deficit. The aim of our study was therefore to re-examine previous results highlighting ToM in PD and to explore the involvement of the dopaminergic pathways in ToM. ToM was investigated in 17 newly diagnosed PD patients (early PD group), 27 PD patients in the advanced stages of the disease (advanced PD group) and 26 healthy matched controls (HC), using two ToM tasks: a visual one, which is thought to reflect the "affective" ToM subcomponent ("Reading the Mind in the Eyes"), and a verbal one, which is thought to reflect both the "affective" and the "cognitive" ToM subcomponents (faux pas recognition). Furthermore, the early PD group was studied in two conditions: with and without dopamine replacement therapy (DRT). We failed to find any significant difference in ToM between the early PD patients and the HC group. Furthermore, there was no difference between the early PD patients in the medicated and unmedicated conditions. Conversely, the advanced PD patients scored poorly on the intention attribution question ("cognitive" ToM score) in the faux pas recognition task. The present results suggest that the deficit in ToM only occurs in the more advanced

stages of the disease. In addition, our results would appear to indicate that these advanced PD patients present "cognitive" ToM impairment rather than global ("cognitive" and "affective") ToM impairment. In other words, the ToM deficit would appear to be present in PD patients where the degenerative process has spread beyond the dopaminergic pathways, but not in early PD patients where neuronal loss is thought to be restricted to the nigrostriatal and mesolimbic dopaminergic systems. In conclusion, our results suggest that the dopaminergic pathways are not involved in ToM.

**Date**	2009 Jan
**Language**	eng
**Extra**	Place: England
**Volume**	47
**Pages**	406-414
**Publication**	Neuropsychologia
**DOI**	10.1016/j.neuropsychologia.2008.09.008
**Issue**	2
**Journal Abbr**	Neuropsychologia
**ISSN**	0028-3932
**PMID**	18845171
**Date Added**	6.7.2025, 19:09:41
**Modified**	5.9.2025, 14:50:56

Notes:

Included

Sample characteristics

Size: 17 EPD (early PD), 27 APD (advanced PD), 26 HC

PD-type: Idiopathic PD

PD-duration: EPD: M = 2.5, SD = 1.5; APD: M = 10.5, SD = 4.9

Medication: Assessment in ON and OFF

Hoehn-Yahr (ON): EPD: M = 1.0, SD = 0.9; APD: M = 1.3, SD = 0.9

UPDRS-3: NA

Gender (male): EPD: 5 (29%); APD: 18 (66%)

Age: EPD: EPD: M = 61, SD = 7.1; APD: M = 56.6, SD = 7.8

Other neurological disease (tumor, stroke, etc.): none

Other major psychopathology: none

Origin country (or ethnicity): France

method behavioural

instruments used in order to quantify the variables

Social cognition aspect: ToM (affective and cognitive)

Name of the task: Faux pas recognition task

Type of stimulus [face/voice etc., Ekman faces/other etc.]: a story which may or may not contain a social faux pas. 5 faux pas, 5 control stories

Task condition: Patients had to answers specific questions regarding the stories

Operationalization:

Several composite scores and subscores were also calculated. The correct hits score was broken down into four subscores as follows:

1. Detection score (question 1 × 5 faux pas stories, maximum = 5),
2. Explanation score (questions 2 to 6 × 5 faux pas stories, maximum = 25),
3. Intention attribution score (question 5 × 5 faux pas stories, maximum = 5),
4. Emotion attribution score (question 6 × 5 faux pas stories, maximum = 5).

A composite “total detection” score was calculated as follows: the detection score plus the correct rejects score (maximum = 15).

A composite “total control questions” score was calculated as follows: the correct faux pas story control score plus the correct neutral story control score (maximum = 20). All these additional scores were converted into a percentage of correct responses

---

Social cognition aspect: ToM (affective)

Name of the task: Reading the Min in the eyes test

Type of stimulus [face/voice etc., Ekman faces/other etc.]: 17 photographs of the eye region of the faces of male and female actors

Task condition: Four adjectives corresponding to complex mental state descriptors (e.g. hateful, panicked) were printed on each slide, with one adjective in each corner and the photograph in the middle. One of these words (the target word) correctly described the mental state of the person in the photograph, while the others were included as foils

Operationalization: The maximum ‘‘Emotion score’’ on the test was therefore 17, which was converted into a percentage of correct responses

Main findings related to the review's scope

Fauxpas task

A significant difference was found for the intention attribution score (“cognitive” ToM score) between the early PD patient group in the unmedicated condition, the advanced PD group and the HC group.

advanced PD group was impaired in comparison with the early PD group in the unmedicated condition (U = 105.5, p = 0.03) and the HC group (U = 224.5, p = 0.02). No significant difference, however, was found between the early PD group in the unmedicated condition and the HC group (U = 167.0, p = 0.9).

Paired comparisons for the intention attribution score (“cognitive” ToM score) showed that the advanced PD group was impaired in comparison with the HC group (U = 224.5, p = 0.02). No significant difference, however, was found between the advanced PD group and the early PD group in the medicated condition (U = 186.5, p = 0.3), nor between the early PD group in the medicated condition and the HC group (U = 179.5, p = 0.3).

Paired comparisons for the correct hits score showed that the advanced PD group was impaired in comparison with the HC group (U = 240.5, p = 0.045)

Paired comparisons for the explanation score showed a trend towards significant difference between the advanced PD group and the HC group (U = 262.0, p = 0.09), and between the advanced PD group and the early PD group in the unmedicated condition (U = 115.5, p = 0.07)

No significant difference was found between the three groups, i.e. the early PD group (medicated/ unmedicated), the advanced PD group and the HC group, for any of the other variables of the faux pas recognition test, including the “affective” ToM score, i.e. the emotion attribution score.

no significant difference was found between the medicated and unmedicated conditions in the early PD group for any of the variables of the faux pas recognition test (correct hits score: Z = (1.5, p = .1; correct rejects score: Z = (0.5, p = .5; correct faux pas story control score: Z = (0.8, p = .4; correct neutral story control score: Z = (0.4, p = .6; detection score: Z = (1.1, p = .2; explanation score: Z = (1.5, p = .1; intention attribution score: Z = (0.9, p = .3; emotion attribution score: Z = (1.4, p = .1; total detection score: Z = (0.3, p = .7; total control questions score: Z = (0.5, p = .5).

RMET

No significant difference was found between the three groups, i.e. the early PD group (medicated/ unmedicated), the advanced PD group and the HC group

No significant difference was found between the medicated and unmedicated conditions in the early PD group for any of the variables of the Reading the Mind in the Eyes test (gender score: Z = (0.7, p = .4; emotion score: Z = (0.1, p = .5).

Tags: empathy, ToM, behavioral

[Are non-literal language comprehension deficits related to a theory of mind deficit in Parkinson's disease?].

**Item Type**	Journal Article
**Author**	Julie Vachon-Joannette
**Author**	Christina Tremblay
**Author**	Mélanie Langlois
**Author**	Sophie Chantal
**Author**	Laura Monetta
**Abstract**	Theory of mind (TOM), i.e. the capacity to attribute mental states to oneself and others, would be impaired in Parkinson's disease (PD). Nonliteral language (NLL) comprehension would also be impaired in this disease. The goal of this study was to verify the presence of an association between the TOM and NLL comprehension deficits. We assessed 15 individuals in the early stages of PD and 17 healthy controls (HC), comparable on gender, age and education. Each subject completed a TOM evaluation task and a NLL task (i.e. metaphor comprehension). They also completed executive functioning (mental flexibility, inhibition and working memory) evaluation tasks. Our results showed that patients with PD had significant difficulties in the TOM and NLL comprehension tasks compared to HC participants. A significant relationship was found between TOM and NLL comprehension results. Moreover, NLL scores were associated with a task evaluating mental flexibility. Thus, PD might cause both TOM and NLL comprehension deficit even in the early stages of the disease. Our results showed that there would be a close relationship between TOM and NLL in people with PD.
**Date**	2013 Jun
**Language**	fre
**Extra**	Place: France
**Volume**	11
**Pages**	208-214
**Publication**	Geriatrie et psychologie neuropsychiatrie du vieillissement
**DOI**	10.1684/pnv.2013.0402
**Issue**	2
**Journal Abbr**	Geriatr Psychol Neuropsychiatr Vieil
**ISSN**	2115-8789 2115-7863
**PMID**	23803638
**Date Added**	6.7.2025, 19:09:41
**Modified**	5.9.2025, 14:59:24

Notes:

Not included: French language

Tags: EXCLUDED

Are patients with Parkinson's disease blind to blindsight?

**Item Type**	Journal Article
**Author**	Nico J. Diederich
**Author**	Glenn Stebbins
**Author**	Christine Schiltz
**Author**	Christopher G. Goetz
**Abstract**	In Parkinson's disease, visual dysfunction is prominent. Visual hallucinations can be a major hallmark of late stage disease, but numerous visual deficits also occur in early stage Parkinson's disease. Specific retinopathy, deficits in the primary visual pathway and the secondary ventral and dorsal pathways, as well as dysfunction of the attention pathways have all been posited as causes of hallucinations in Parkinson's disease. We present data from patients with Parkinson's disease that contrast with a known neuro-ophthalmological syndrome, termed 'blindsight'. In this syndrome, there is an absence of conscious object identification, but preserved 'guess' of the location of a stimulus, preserved reflexive saccades and motion perception and preserved autonomical and expressive reactions to negative emotional facial expressions. We propose that patients with Parkinson's disease have the converse of blindsight, being 'blind to blindsight'. As such they preserve conscious vision, but show erroneous 'guess' localization of visual stimuli, poor saccades and motion perception, and poor emotional face perception with blunted autonomic reaction. Although a large data set on these deficits in Parkinson's disease has been accumulated, consolidation into one specific syndrome has not been proposed. Focusing on neuropathological and physiological data from two phylogenetically old and subconscious pathways, the retino-colliculo-thalamo-amygdala and the retino-geniculo-extrastriate pathways, we propose that aberrant function of these systems, including pathologically inhibited superior colliculus activity, deficient corollary discharges to the frontal eye fields, dysfunctional pulvinar, claustrum and amygdaloid subnuclei of the amygdala, the latter progressively burdened with Lewy bodies, underlie this syndrome. These network impairments are further corroborated by the concept of the 'silent amygdala'. Functionally being 'blind to blindsight' may facilitate the highly distinctive 'presence' or 'passage' hallucinations of Parkinson's disease and can help to explain handicaps in driving capacities and dysfunctional 'theory of mind'. We propose this synthesis to prompt refined neuropathological and neuroimaging studies on the pivotal nuclei in these pathways in order to better understand the networks underpinning this newly conceptualized syndrome in Parkinson's disease.
**Date**	2014 Jun
**Language**	eng
**License**	© The Author (2014). Published by Oxford University Press on behalf of the Guarantors of Brain.
**Extra**	Place: England
**Volume**	137
**Pages**	1838-1849
**Publication**	Brain : a journal of neurology
**DOI**	10.1093/brain/awu094
**Issue**	Pt 6
**Journal Abbr**	Brain
**ISSN**	1460-2156 0006-8950
**PMID**	24764573

PMCID PMC4032103
Date Added 6.7.2025, 19:09:41
Modified 5.9.2025, 14:33:39

Notes:

Not Included: not an empirical study
Tags: EXCLUDED

Are patients with Parkinson's disease impaired in the recognition of emotion's authenticity?

Item Type Journal Article
Author Agnese Anzani
Author Stefano Zago
Author Teresa Difonzo
Author Cristina Scarpazza
Author Nadia Bolognini
Author Giulia Franco
Author Alessio Difonzo
Author Maria Cristina Saetti
Abstract In recognising emotions expressed by others, one can make use of both embodied cognition and mechanisms that do not necessarily require activation of the limbic system, such as evoking from memory the meaning of morphological features of the observed face. Instead, we believe that the recognition of the authenticity of an emotional expression is primarily based on embodied cognition, for which the mirror system would play a significant role. To verify this hypothesis, we submitted 20 parkinsonian patients and 20 healthy control subjects to the Emotional Authenticity Recognition test, a novel test using dynamic stimuli to evaluate the ability to recognise emotions and their authenticity. Analysis of variance of the test scores shows that Parkinsonian patients perform worse than controls when they had to recognise the authenticity of emotions, although they are able to identify them. Our results confirm a deficit in the recognition of the authenticity of emotions in patients with Parkinson's disease attributable to the disruption of extrapiramidal limbic circuit between ventral striatum and orbitomesial-prefrontal cortex.
Date 2024 Dec 26
Language eng
License © 2024 The Author(s). Journal of Neuropsychology published by John Wiley & Sons Ltd on behalf of The British Psychological Society.
Extra Place: England
Publication Journal of neuropsychology
DOI 10.1111/jnp.12410
Journal Abbr J Neuropsychol
ISSN 1748-6653 1748-6645
PMID 39726098
Date Added 6.7.2025, 19:09:36

Modified 5.9.2025, 14:26:28

Notes:

Included

sample characteristics

size: 20 PD and 20 HC MATCHED age, gender and years of education

Parkinson's Disease type and duration: , Mduration= 6.05 (SD=5.91) 1–28

Medication: on medication

Hoehn-Yahr: NA

UPDRS-3: M= 15.35 (SD=8.09) 4–31

Gender (male): 12 males (60 %)

averaged ages (SD, range): M= 65.8 (SD=12.5)

other neurological disease (tumor, stroke, etc.): None

other major psychopathology: None

origin country (or ethnicity): Italy

method observational

instruments used in order to quantify the variables

Social cognition aspect: emotion recognition

Name of the task: the Emotional Authenticity Recognition (EAR) test

type of stimulus [face/voice etc., Ekman faces/other etc.]: short videos of faces expressing the six basic emotions. Facial expressions belong to subjects filmed both when conveying a genuine emotional reaction induced by visual or non-visual input and when acting it out

task condition: happiness, sadness, fear, surprise, anger and disgust

operationalization: accuracy in identification and authenticity

Main findings related to the review's scope

Recognition: emotion recognition identification did not differ significantly between the groups

Authenticity: Compared to HC, PD patients scored lower on the emotion authenticity identification. PD patients are more likely to consider authentic expressions actually simulated than vice versa.

Tags: Emotion recognition, behavioral

Are perception and memory for faces influenced by a specific age at onset factor in Parkinson's disease?

**Item Type**	Journal Article
**Author**	H.C Haeske-Dewick
**Date**	4/1996
**Language**	en
**Library Catalog**	DOI.org (Crossref)
**URL**	https://linkinghub.elsevier.com/retrieve/pii/0028393295001093
**Accessed**	11.8.2025, 17:04:23
**License**	https://www.elsevier.com/tdm/userlicense/1.0/
**Volume**	34
**Pages**	315-320
**Publication**	Neuropsychologia
**DOI**	10.1016/0028-3932(95)00109-3
**Issue**	4
**Journal Abbr**	Neuropsychologia
**ISSN**	00283932
**Date Added**	11.8.2025, 17:04:23
**Modified**	11.8.2025, 17:04:23

Notes:

Included

sample characteristics

size: 13 early-onset PD (YPD) 13 late-onset PD (OPD) and 26 HC (two groups, age-matched to the two PD groups)

Parkinson's Disease type and duration: idiopathic PD, YPD Mduration= 8.23 years (S.D. = 5.30). OPD Mduration= 8.38 years (S.D. = 4.97).

Medication: on medication

Hoehn-Yahr: YPD M=2.92 SD=1.04 (1-5) OPD M=3.15 SD=.90 (2-5)

UPDRS-3: NA

Gender (male): YPD 8 males (62%) OPD 9 males (69%)

averaged ages (SD, range): YPD M= 55.80 years (S.D. = 6.24) OPD M= 73.88 years (S.D.=6.15)

other neurological disease (tumor, stroke, etc.): None

other major psychopathology: None

origin country (or ethnicity): NA

method observational

instruments used in order to quantify the variables

Social cognition aspect: emotion recognition

Name of the task: NA

type of stimulus [face/voice etc., Ekman faces/other etc.]: Pairs of photographs of the same person's face with different expressions are presented.

task conditions: (happy, angry, sad)

operationalization: Accuracy

Main findings related to the review's scope

No significant differences.

Tags: Emotion recognition, behavioral

Are there basic emotions?

Item Type Journal Article
Author Paul Ekman
Date 1992

Language en
Library Catalog DOI.org (Crossref)
URL https://doi.apa.org/doi/10.1037/0033-295X.99.3.550
Accessed 19.1.2026, 7:51:32
Volume 99
Pages 550-553
Publication Psychological Review
DOI 10.1037/0033-295X.99.3.550
Issue 3
Journal Abbr Psychological Review
ISSN 1939-1471, 0033-295X
Date Added 19.1.2026, 7:51:32
Modified 19.1.2026, 7:51:32

Are you tuned in to others' mind? A cross-modal evaluation of affective theory of mind in people with Parkinson's disease.

Item Type Journal Article
Author Elisa Menozzi
Author Daniela Ballotta
Author Francesco Cavallieri
Author Stefania Tocchini
Author Sara Contardi
Author Valentina Fioravanti
Author Franco Valzania
Author Paolo F. Nichelli
Author Francesca Benuzzi
Abstract Affective Theory of Mind (ToM) is the ability to understand other peoples' emotional states and feelings. Several studies showed impaired affective ToM abilities in people with Parkinson's disease (PD). However, most studies tested this ability by using single-stimulus modality tasks (visual cues). The aim of the present study was to evaluate whether affective ToM abilities are impaired in PD using multiple stimulus modalities and whether they are related to disease duration and cognitive/emotional abilities. Twenty mid-stage, non-demented people with PD and 20 matched controls were evaluated by means of two tasks requiring subjects to infer others' mental states from the eyes' expression - the Reading the Mind in the Eyes (RME) test, and from the emotional prosody - a modified version of the Reading the Mind in the Voice (RMV) test. In people with PD, cognitive function and emotional processes were assessed through cognitive tasks and batteries of facial and prosodic emotion recognition tests. Compared to controls, the PD group showed significantly impaired performances in both RME and RMV tests ($p < 0.0001$). Affective ToM abilities in people with PD were not correlated with cognitive function or disease duration. Visual recognition of anger and auditory recognition of fear were positively correlated with RME ($p = 0.0028$) and RMV ($p < 0.0001$) test scores, respectively. Cross-modal impairment of affective ToM abilities is a non-motor feature of PD, unrelated to disease stage or cognitive status. A dysfunctional amygdala-centred network might represent the shared bases for impairments in fear and anger

recognition and affective ToM abilities in PD.

**Date**	2025 Feb
**Language**	eng
**License**	Copyright © 2024 The Authors. Published by Elsevier B.V. All rights reserved.
**Extra**	Place: Netherlands
**Volume**	252
**Pages**	104686
**Publication**	Acta psychologica
**DOI**	10.1016/j.actpsy.2024.104686
**Journal Abbr**	Acta Psychol (Amst)
**ISSN**	1873-6297 0001-6918
**PMID**	39733767
**Date Added**	6.7.2025, 19:09:41
**Modified**	5.9.2025, 14:46:27

Notes:

Included

Sample characteristics

Size: 32 PD, 20 HC (asge, sex, education matched healthc HC)

PD-type: NA

PD-duration: M = 6.35, SD = 4.04

Medication: LED reported

Hoehn-Yahr: Median = 2.5

UPDRS-3: M = 20.95, SD = 9.77

Gender (male): 13 (65%)

Age: M = 70.65, SD = 7.08 7.08

Other neurological disease (tumor, stroke, etc.): NA

Other major psychopathology: NA

Origin country (or ethnicity): Italy

method behavioural

instruments used in order to quantify the variables

Social cognition aspect: ToM

Name of the task: modified RMET

Type of stimulus [face/voice etc., Ekman faces/other etc.]: 30 out of 36 items selected

Task condition: 3 forced choice

Operationalization: correct answers

Social cognition aspect: ToM

Name of the task: modified reading the mind in the voice test (RMVT

Type of stimulus [face/voice etc., Ekman faces/other etc.]: 30 short spoken sentences with a semantic content not corresponding to the affective rposody used to pronounce them.

Task condition: 4 forced choice to describe how the speaker was feeling

Operationalization: Errors, Semantic error (SE, the coice of semantic-related answer). Percentage of SD (SD%) was calculated on the toal number of errors.

Main findings related to the review's scope

PD scored significantly worse in both the RME (t = 4.7616, p < 0.0001, and the RMV

The observed difference in ToM abilities remained significant for both tests even after adjustment for

covariates ($p < 0.0001$ for both linear regression models).($t = 4.7173$, $p < 0.0001$,

Most of the errors in the RMV test were sematic-related. However, no differences in the percentage of SE were detected between PD and HC groups, suggesting that the impaired perception of intonation patterns in the PD group was not related to language deficits

Tags: ToM, behavioral

Assessing the Ability to Recognize Facial and Vocal Expressions of Emotion: Construction and Validation of the Emotion Recognition Index

**Item Type**	Journal Article
**Author**	Klaus R. Scherer
**Author**	Ursula Scherer
**Date**	12/2011
**Language**	en
**Short Title**	Assessing the Ability to Recognize Facial and Vocal Expressions of Emotion
**Library Catalog**	DOI.org (Crossref)
**URL**	http://link.springer.com/10.1007/s10919-011-0115-4
**Accessed**	19.1.2026, 7:15:27
**License**	http://www.springer.com/tdm
**Volume**	35
**Pages**	305-326
**Publication**	Journal of Nonverbal Behavior
**DOI**	10.1007/s10919-011-0115-4
**Issue**	4
**Journal Abbr**	J Nonverbal Behav
**ISSN**	0191-5886, 1573-3653
**Date Added**	19.1.2026, 7:15:27
**Modified**	19.1.2026, 7:15:27

Association Between Social Cognition Changes and Resting State Functional Connectivity in Frontotemporal Dementia, Alzheimer's Disease, Parkinson's Disease, and Healthy Controls.

**Item Type**	Journal Article
**Author**	Namita Multani
**Author**	Foad Taghdiri
**Author**	Cassandra J. Anor
**Author**	Brenda Varriano
**Author**	Karen Misquitta
**Author**	David F. Tang-Wai
**Author**	Ron Keren

Author Susan Fox
Author Anthony E. Lang
Author Anne Catherine Vijverman
Author Connie Marras
Author Maria Carmela Tartaglia

Abstract OBJECTIVE: To determine the relationship between alterations in resting state functional connectivity and social cognition dysfunction among patients with frontotemporal dementia (FTD), Alzheimer's disease (AD), Parkinson's disease (PD), and healthy controls (HC). METHODS: Fifty-seven participants (FTD = 10, AD = 18, PD = 19, and HC = 10) underwent structural and functional imaging and completed the Awareness of Social Inference Test-Emotion Evaluation Test (TASIT-EET), Behavioral Inhibition System/Behavioral Activation System (BIS/BAS) scale, Revised Self-Monitoring Scale (RSMS), Interpersonal Reactivity Index (IRI), and Social Norms Questionnaire (SNQ). A multi-variate pattern analysis (MVPA) was carried out to determine activation differences between the groups. The clusters from the MVPA were used as seeds for the ROI-to-voxel analysis. Relationship between social cognition deficits and uncinate integrity was also investigated. RESULTS: BOLD signal activation differed among the four groups of AD, PD, FTD, and HC in the left inferior temporal gyrus-anterior division [L-ITG (ant)], right central opercular cortex (R-COp), right supramarginal gyrus, posterior division (R-SMG, post), right angular gyrus (R-AG), and R-ITG. The BOLD co-activation of the L-ITG (ant) with bilateral frontal pole (FP) and paracingulate gyrus was positively associated with IRI-perspective taking (PT) ($r = 0.38$, $p = 0.007$), SNQ total ($r = 0.37$, $p = 0.009$), and TASIT-EET ($r = 0.47$, $p < 0.001$). CONCLUSION: Patients with neurodegenerative diseases showed alterations in connectivity in brain regions important for social cognition compared with HCs. Functional connectivity correlated with performance on social cognition tasks and alterations could be responsible for some of the social cognition deficits observed in all neurodegenerative diseases.

Date 2019
Language eng
License Copyright © 2019 Multani, Taghdiri, Anor, Varriano, Misquitta, Tang-Wai, Keren, Fox, Lang, Vijverman, Marras and Tartaglia.
Extra Place: Switzerland
Volume 13
Pages 1259
Publication Frontiers in neuroscience
DOI 10.3389/fnins.2019.01259
Journal Abbr Front Neurosci
ISSN 1662-4548 1662-453X
PMID 31824254
PMCID PMC6883726
Date Added 6.7.2025, 19:09:34
Modified 5.9.2025, 14:47:41

Notes:

Not included: No relevant/specific findings linking SC to GM/WM connectivity in PD specifically.

Tags: EXCLUDED

Asymmetric right/left encoding of emotions in the human subthalamic nucleus

Item Type Journal Article
Author Renana Eitan
Author Reuben R. Shamir
Author Eduard Linetsky
Author Ovadya Rosenbluh
Author Shay Moshel
Author Tamir Ben-Hur
Author Hagai Bergman
Author Zvi Israel
Abstract Emotional processing is lateralized to the non-dominant brain hemisphere. However, there is no clear spatial model for lateralization of emotional domains in the basal ganglia. The subthalamic nucleus (STN), an input structure in the basal ganglia network, plays a major role in the pathophysiology of Parkinson's disease (PD). This role is probably not limited only to the motor deficits of PD, but may also span the emotional and cognitive deficits commonly observed in PD patients. Beta oscillations (12-30 Hz), the electrophysiological signature of PD, are restricted to the dorsolateral part of the STN that corresponds to the anatomically defined sensorimotor STN. The more medial, more anterior and more ventral parts of the STN are thought to correspond to the anatomically defined limbic and associative territories of the STN. Surprisingly, little is known about the electrophysiological properties of the non-motor domains of the STN, nor about electrophysiological differences between right and left STNs. In this study, microelectrodes were utilized to record the STN spontaneous spiking activity and responses to vocal non-verbal emotional stimuli during deep brain stimulation (DBS) surgeries in human PD patients. The oscillation properties of the STN neurons were used to map the dorsal oscillatory and the ventral non-oscillatory regions of the STN. Emotive auditory stimulation evoked activity in the ventral non-oscillatory region of the right STN. These responses were not observed in the left ventral STN or in the dorsal regions of either the right or left STN. Therefore, our results suggest that the ventral non-oscillatory regions are asymmetrically associated with non-motor functions, with the right ventral STN associated with emotional processing. These results suggest that DBS of the right ventral STN may be associated with beneficial or adverse emotional effects observed in PD patients and may relieve mental symptoms in other neurological and psychiatric diseases.
Date 2013
Language English
Extra Place: AVENUE DU TRIBUNAL FEDERAL 34, LAUSANNE, CH-1015, SWITZERLAND Type: Article
Volume 7
Publisher FRONTIERS MEDIA SA
Publication FRONTIERS IN SYSTEMS NEUROSCIENCE
DOI 10.3389/fnsys.2013.00069
Date Added 14.7.2025, 14:50:39
Modified 5.9.2025, 14:34:51

Notes:

Not Included: only with a sample after STN-DBS
Tags: EXCLUDED

Automatic and controlled attentional orienting toward emotional faces in patients with Parkinson's disease.

**Item Type**	Journal Article
**Author**	Stefania Righi
**Author**	Giorgio Gronchi
**Author**	Silvia Ramat
**Author**	Gioele Gavazzi
**Author**	Francesca Cecchi
**Author**	Maria Pia Viggiano
**Abstract**	Parkinson's disease (PD) is a neurodegenerative motor disorder that can associate with deficits in cognitive and emotional processing. In particular, PD has been reported to be mainly associated with defects in executive control and orienting attentional systems. The deficit in emotional processing mainly emerged in facial expression recognition. It is possible that the defects in emotional processing in PD may be secondary to other cognitive impairments, such as attentional deficits. This study was designed to systematically investigate the different weight of automatic and controlled attentional orienting mechanisms implied in emotional selective attention in PD. To address our purpose, we assessed drug-naïve PD patients and age-matched healthy controls with two dot-probe tasks that differed for stimuli duration. Automatic and controlled attentions were evaluated with stimuli lasting 100 ms and 500 ms, respectively. Furthermore, we introduced an emotion recognition task to investigate the performance in explicit emotion classification. The stimuli used in both the tasks dot-probe and emotion recognition were expressive faces displaying neutral, disgusted, fearful, and happy expressions.Our results showed that in PD patients, compared with healthy controls, there was 1) an alteration of automatic and controlled attentional orienting toward emotional faces in both the dot-probe tasks (with short and long durations), and 2) no difference in the emotion recognition task. These findings suggest that, from the early stages of the disease, PD can yield specific deficits in implicit emotion processing task (i.e., dot-probe task) despite a normal performance in explicit tasks that demand overt emotion recognition.
**Date**	2023 Apr
**Language**	eng
**License**	© 2023. The Author(s).
**Extra**	Place: United States
**Volume**	23
**Pages**	371-382
**Publication**	Cognitive, affective & behavioral neuroscience
**DOI**	10.3758/s13415-023-01069-5
**Issue**	2
**Journal Abbr**	Cogn Affect Behav Neurosci
**ISSN**	1531-135X 1530-7026
**PMID**	36759426

PMCID PMC10050058
Date Added 6.7.2025, 19:09:36
Modified 5.9.2025, 14:52:46

Notes:

Included

Sample characteristics

Size: 31 PD, 33 HC (age-matched)

PD-type: Idiopathic PD

PD-duration: M = 0.62, SD = 0.46 (0.08-1.67)

Medication: NA

Hoehn-Yahr: M = 1.59, SD = 0.53

UPDRS-3: M = 10.70, SD = 5.51

Gender (male): 15 (48%)

Age: M = 65.85, SD = 7.56

Other neurological disease (tumor, stroke, etc.): none

Other major psychopathology: none

Origin country (or ethnicity): NA

method behavioural

instruments used in order to quantify the variables

Social cognition aspect: Facial Emotion recognition

Name of the task: NA

Type of stimulus [face/voice etc., Ekman faces/other etc.]: Sixteen face identities (8 females) were taken from the Karolinska Directed Emotional Faces (KDEF) database.

Task condition: The 64 faces (16 face identities with 4 expressions: neutral, disgusted, fearful, and happy) were centrally presented in a random way.

Operationalization: Subjects were requested to classify face expression by pressing four buttons on the keyboard. >> Proportion of accuracy and RTs

---

Main findings related to the review's scope

accuracy: No significant differences emerged for the Group variable or for the interaction Emotion x Group.

RT: No significant differences for the Group variable or for the interaction Emotion x Group.

Tags: emotion recognition, behavioral

Basal ganglia contributions to social cognition: evidence from movement disorders

Item Type Journal Article
Author Clare M. Eddy
Date 2025-01-02
Volume 30
Pages 1-14
Publication COGNITIVE NEUROPSYCHIATRY
DOI 10.1080/13546805.2025.2490054
Issue 1
ISSN 1354-6805
Date Added 14.7.2025, 14:48:38
Modified 5.9.2025, 14:34:41

Notes:

Not Included: not a systematic review
Tags: EXCLUDED

Basic and Social Emotion Recognition in Patients With Parkinson Disease

Item Type Journal Article
Author Ana Martins
Author Angelica Muresan
Author Mariline Justo
Author Claudia Simao
Abstract The loss of dopaminergic neurons in Parkinson Disease (PD) patients has been associated with impaired emotion recognition. It has been speculated in previous studies that patients with hypodopaminergic state exhibited weaker amygdala activation in response to facial stimuli. In this study we investigate the processing of social and basic emotions through facial expressions in a sample of seventeen idiopathic PD patients matched by age and education level to a group of twenty healthy controls. Our goal was to assess participants' ability to recognize facial expressions of five basic emotions (happiness, fear, sadness, anger and surprise) and three social emotions (arrogance, guilt and jealousy). The basic emotions' group was assessed through a forced-choice labeling task whereas for the social emotions' group a Go/No-Go task was designed. Our results suggest that PD participants' recognition of basic and social emotions was overall reduced relative to controls, with significant differences for facial expressions of fear and anger (basic emotions' group) and facial expressions of arrogance (social emotions' group). These results were discussed within a framework suggesting that hypodopaminergic state is an influence in basic and social emotion recognition.
Date 2008
Language English
Extra Place: EGE UNIV HOSP. FAC MED, DEPT NEUROSURGERY, BORNOVA-IZMIR, TR35100, TURKEY Type: Article
Volume 25
Publisher JOURNAL NEUROLOGICAL SCIENCES

Pages 247-257
Publication JOURNAL OF NEUROLOGICAL SCIENCES-TURKISH
Issue 4
ISSN 1302-1664
Date Added 14.7.2025, 14:50:42
Modified 5.9.2025, 14:45:22

Notes:

Included

Sample characteristics

Size: 17 PD; 20 HC (matched for age and education).

PD-type: Idiopathic PD

PD-duration: M=3.86, SD = 0.98

Medication: OFF state

Hoehn-Yahr: Range = 1-2; M = 1.05, SD = 0.28

UPDRS-3: NA

Gender (male): 0 (0%) All participants female.

Age: M = 69.39, SD = 3.36

Other neurological disease (tumor, stroke, etc.): No cognitive impairments

Other major psychopathology: Mild depression for patients (as measured by BDI).

Origin country (or ethnicity): Portugal

method observational

instruments used in order to quantify the variables

Social cognition aspect: Basic emotion recognition (facial)

Name of the task: NA

Type of stimulus [face/voice etc., Ekman faces/other etc.]: Black and white photographs. Created their own stimuli using three actors (1 male, 2 female). Disgust stimulus was excluded because was not consistent between judges. Each actor represented all five basic emotions in study, which mean there were displayed fifteen different emotional photographs. Each emotion was presented six times by the same actor (a total of eighteen times between the three actors), participants perceived a total of 90 stimuli, 18 per emotion

Task condition: Forced-choice (5-way; happiness, sadness, anger, fear, surprise). For each emotion, score "1" was considered for each correct answer (minimum score = 0; maximum score = 18).

The neutral facial expression photograph was shown first and available for observation for 1500 ms, followed by emotional photographs, each presented during 500 ms. Decision labels had a forced-choice format and participants could choose one of the five presented emotions during 10000 ms. After the participants' decision or 10000 ms expired, a next trial started automatically

Operationalization: Accuracy and reaction times

Social cognition aspect: Social emotion recognition (facial)

Name of the task: NA

Type of stimulus [face/voice etc., Ekman faces/other etc.]: Black-white photographs. Two actresses were selected to represent three social emotions. (arrogance, jealousy, guilt).

Task condition: Go-NoGo. Three blocks of stimuli presentation (one block for each social emotion on study): one arrogance block, one jealousy block and one guilt block, all applied separately. Between the three blocks of social emotion photographs and one neutral facial photograph, a total of 8 different photographs were combined. Each participant perceived twenty-four photographs per block but only 50% of them were correct: those twelve correct represented emotions from the block and the other 50% of the photographs presented in the same sequence were emotions from the other two blocks. Score "1" was considered for each correct answer (minimum score = 0; maximum score = 12). Were instructed beforehand which emotion they had to identify.

Operationalization: Accuracy and reaction times

Main findings related to the review's scope

Basic emotions:

Superior levels of accuracy for control group comparatively to Parkinson's disease group. Despite of controls' performance superiority detected for all basic emotions represented in the present study, differences were significant only for fear [Z= -2.65270; p = .008] and anger recognition [Z = -2.45423; p = .014].

HC had overall better reaction times for emotion recognition than PD participants, thus they responded more rapidly to stimuli. Still, significant differences were found only for fear recognition [Z = -2.4873; p= .0024].

Social Emotions

The results for accuracy in social emotion recognition indicated inferior accuracy levels in Parkinson's disease participants for all social emotions represented. However, these differences were only significant for arrogance recognition (Z = - 2.86522; p = .004).

controls performed overall inferior reaction times than PD patients for social emotion recognition task. In spite of the tendentious results, participants' performance did not reveal significant differences for any of the three social emotions in study.

Tags: Emotion recognition, Behavioral

Beyond emotion recognition deficits: A theory guided analysis of emotion processing in Huntington's disease

Item Type Journal Article

Author Catarina C. Kordsachia

Author Izelle Labuschagne

Author Julie C. Stout

Abstract Deficits in facial emotion recognition in Huntington's disease (HD) have been extensively researched, however, a theory-based integration of these deficits into the broader picture of emotion processing is lacking. To describe the full extent of emotion processing deficits we reviewed the clinical research literature in HD, including a consideration of research in Parkinson's disease, guided by a theoretical model on emotion processing, the Component Process Model. Further, to contribute to understanding the mechanisms underlying deficient emotion recognition, we discussed the literature in light of specific emotion recognition theories. Current evidence from HD studies indicates deficits in the production of emotional facial

expressions and alterations in subjective emotional experiences, in addition to emotion recognition deficits. Conceptual understanding of emotions remains relatively intact. Impaired recognition and expression of emotion in HD might be linked, whereas altered emotional experiences appear to be unrelated to emotion recognition. A key implication of this review is the need to take all the components of emotion processing into account to understand specific deficits in neurodegenerative diseases. (C) 2016 Elsevier Ltd. All rights reserved.

Date 2017-02
Language English
Extra Place: THE BOULEVARD, LANGFORD LANE, KIDLINGTON, OXFORD OX5 1GB, ENGLAND Type: Review
Volume 73
Publisher PERGAMON-ELSEVIER SCIENCE LTD
Pages 276-292
Publication NEUROSCIENCE AND BIOBEHAVIORAL REVIEWS
DOI 10.1016/j.neubiorev.2016.11.020
ISSN 0149-7634
Date Added 14.7.2025, 14:50:35
Modified 5.9.2025, 14:42:15

Notes:

Not Included: Not on Parkinson

Tags: EXCLUDED

Bias between MNI and Talairach coordinates analyzed using the ICBM-152 brain template

Item Type Journal Article

Author Jack L. Lancaster
Author Diana Tordesillas-Gutiérrez
Author Michael Martinez
Author Felipe Salinas
Author Alan Evans
Author Karl Zilles
Author John C. Mazziotta
Author Peter T. Fox

Abstract Abstract MNI coordinates determined using SPM2 and FSL/ FLIRT with the ICBM-152 template were compared to Talairach coordinates determined using a landmark-based Talairach registration method (TAL). Analysis revealed a clear-cut bias in reference frames (origin, orientation) and scaling (brain size). Accordingly, ICBM-152 fitted brains were consistently larger, oriented more nose down, and translated slightly down relative to TAL fitted brains. Whole brain analysis of MNI/Talairach coordinate disparity revealed an ellipsoidal pattern with disparity ranging from zero at a point deep within the left hemisphere to greater than 1-cm for some anterior brain areas. MNI/Talairach coordinate disparity was generally

less for brains fitted using FSL. The mni2tal transform generally reduced MNI/ Talairach coordinate disparity for inferior brain areas but increased disparity for anterior, posterior, and superior areas. Coordinate disparity patterns differed for brain templates (MNI-305, ICBM-152) using the same fitting method (FSL/ FLIRT) and for different fitting methods (SPM2, FSL/ FLIRT) using the same template (ICBM-152). An MNI-to-Talairach (MTT) transform to correct for bias between MNI and Talairach coordinates was formulated using a best-fit analysis in one hundred high-resolution 3-D MR brain images. MTT transforms optimized for SPM2 and FSL were shown to reduced group mean MNI/Talairach coordinate disparity from a 5-13 mm to 1-2 mm for both deep and superficial brain sites. MTT transforms provide a validated means to convert MNI coordinates to Talairach compatible coordinates for studies using either SPM2 or FSL/ FLIRT with the ICBM-152 template. Hum Brain Mapp 2007. © 2007 Wiley-Liss, Inc.

Date 11/2007
Language en
Library Catalog DOI.org (Crossref)
URL https://onlinelibrary.wiley.com/doi/10.1002/hbm.20345
Accessed 8.2.2026, 18:38:36
License http://onlinelibrary.wiley.com/termsAndConditions#vor
Volume 28
Pages 1194-1205
Publication Human Brain Mapping
DOI 10.1002/hbm.20345
Issue 11
Journal Abbr Human Brain Mapping
ISSN 1065-9471, 1097-0193
Date Added 8.2.2026, 18:38:36
Modified 8.2.2026, 18:38:36

Blunted emotion judgments of body movements in Parkinson's disease

Item Type Journal Article
Author Emmanuelle Bellot
Author Antoine Garnier-Crussard
Author Elodie Pongan
Author Floriane Delphin-Combe
Author Marie-Helene Coste
Author Claire Gentil
Author Isabelle Rouch
Author Marie-Anne Henaff
Author Christina Schmitz
Author Barbara Tillmann
Author Pierre Krolak-Salmon
Abstract Some of the behavioral disorders observed in Parkinson's disease (PD) may be related to an altered processing of social messages, including emotional expressions. Emotions conveyed by whole body movements may be difficult to generate and be detected by PD patients. The aim of the present study was to compare valence

judgments of emotional whole body expressions in individuals with PD and in healthy controls matched for age, gender and education. Twenty-eight participants (13 PD patients and 15 healthy matched control participants) were asked to rate the emotional valence of short movies depicting emotional interactions between two human characters presented with the “Point Light Displays” technique. To ensure understanding of the perceived scene, participants were asked to briefly describe each of the evaluated movies. Patients' emotional valence evaluations were less intense than those of controls for both positive ($p < 0.001$) and negative ($p < 0.001$) emotional expressions, even though patients were able to correctly describe the depicted scene. Our results extend the previously observed impaired processing of emotional facial expressions to impaired processing of emotions expressed by body language. This study may support the hypothesis that PD affects the embodied simulation of emotional expression and the potentially involved mirror neuron system.

Date 2021-09-17
Language English
Extra Place: HEIDELBERGER PLATZ 3, BERLIN, 14197, GERMANY Type: Article
Volume 11
Publisher NATURE PORTFOLIO
Publication SCIENTIFIC REPORTS
DOI 10.1038/s41598-021-97788-1
Issue 1
ISSN 2045-2322
Date Added 14.7.2025, 14:50:30
Modified 14.7.2025, 14:50:30

Notes:

Not Included: not an empirical paper

Tags: EXCLUDED

Brain activity of the emotional circuit in Parkinson’s disease patients with freezing of gait

Item Type Journal Article
Author Elisabetta Sarasso
Author Federica Agosta
Author Noemi Piramide
Author Elisa Canu
Author Maria Antonietta Volontè
Author Massimo Filippi
Abstract Objective Emotional processes might influence freezing of gait (FoG) in Parkinson’s disease (PD) patients. We assessed brain functional MRI (fMRI) activity during a “FoG-observation-task” in PD-FoG patients relative to healthy controls. Methods Twenty-four PD-FoG patients and 18 age- and sex-matched healthy controls performed clinical and neuropsychological evaluations, and fMRI experiments including: i) “FoG-observation-task” consisting of watching a patient experiencing

FoG during a walking task (usually evoking FoG); ii) “gait-observation-task” consisting of watching a healthy subject performing similar walking tasks without experiencing FoG. Results During both tasks, PD-FoG patients showed reduced activity of the fronto-parietal mirror neuron system (MNS) relative to controls. In the “FoG-observation-task” relative to the “gait-observation-task”, PD-FoG patients revealed an increased recruitment of the anterior medial prefrontal cortex and a reduced recruitment of the dorsomedial prefrontal cortex and hippocampus relative to controls. Healthy controls in the “FoG-observation-task” relative to the “gait-observation-task” showed increased recruitment of cognitive empathy areas and decreased activity of the fronto-parietal MNS. Conclusion Our results suggest that when PD-FoG patients observe a subject experiencing FoG, there is an increased activity of brain areas involved in self-reflection emotional processes and a reduced activity of areas related to motor programming, executive functions and cognitive empathy. These findings support previous evidence on the critical role of the emotional circuit in the mechanisms underlying FoG.

Date 2021
URL https://www.sciencedirect.com/science/article/pii/S2213158221000930
Volume 30
Pages 102649
Publication NeuroImage: Clinical
DOI https://doi.org/10.1016/j.nicl.2021.102649
ISSN 2213-1582
Date Added 6.7.2025, 19:12:34
Modified 5.9.2025, 14:55:46

Notes:

Included

Sample characteristics

Size: 24 PD, 18 HC (age, sex matched)

PD-type: Idiopathic PD

PD-duration: NA

Medication: on state

Hoehn-Yahr: < 3; M = 2.25, SD 0 0.36 (ON)

UPDRS-3: M = 25.05, SD = 8.82 (ON)

Gender (male): 17 (71%)

Age: M = 66.54, SD = 8.13

Other neurological disease (tumor, stroke, etc.): none

Other major psychopathology: none

Origin country (or ethnicity): Italy

method behavioural (Task while MRI-recording)

instruments used in order to quantify the variables

Social cognition aspect: Empathy

Name of the task: the Empathy Quotient (EQ) questionnaire

Type of stimulus [face/voice etc., Ekman faces/other etc.]: 40 items on cognitive and affective empathy

Operationalization: sum score

Social cognition aspect: Empathy

Name of the task: FoG-observation-task (FoG = Freezing of Gait) in comparison to the "gait-observation task" (control task).

Type of stimulus [face/voice etc., Ekman faces/other etc.]: 2 short videos.

Task condition: Participants, laying down in the MRI scanner couch, were asked to perform two different tasks: i) the "FoG-observation-task" consisting of watching a video in which a PD patient was experiencing FoG during a walking task (Fig. 1A); ii) the "gait-observation-task" consisting of watching a video of a healthy subject performing similar walking tasks (e.g., turning or walking through narrow spaces) without experiencing FoG to adjust for the mere effect of action observation and the relative involvement of the **Mirror Neurons system** (Fig. 1B).

Operationalization: we asked them to focus their thoughts on the feelings induced by the situation.

Main findings related to the review's scope

the Empathy Quotient (EQ) questionnaire score: No sig dif between the PD-FOG and the HC.

fMRI findings

FoG-observation task

PD-FoG patients relative to healthy controls showed a reduced activity of the [redacted] including right [redacted] pars triangularis and opercularis and of the bilateral [redacted] and right SMA

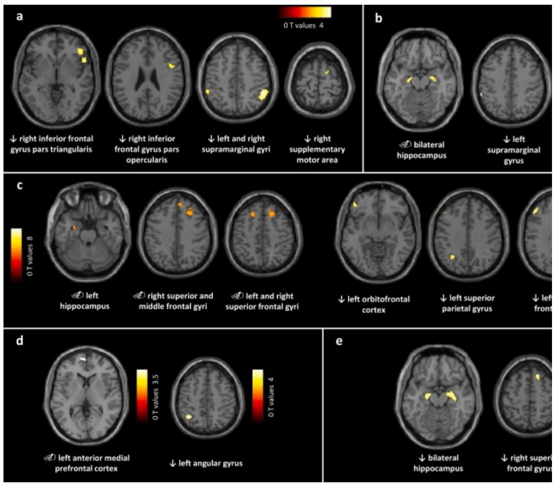

Fig. 2. Task-based functional MRI findings: A. Differences in fMRI patterns of activation between healthy controls and Parkinson's dis
gait (PD-FoG) during the execution of the "FoG-observation-task"; B. Differences in fMRI patterns of activation between healthy con
execution of the "gait-observation-task"; C. Patterns of activation in healthy controls during the comparison between the "FoG-obse
observation-task"; D. Patterns of activation in PD-FoG during the comparison between the "FoG-observation-task" and the "Gait-obser
fMRI patterns of activation in PD-FoG compared to healthy controls performing the "FoG-observation-task" relative to the "gait-obser
shown at $p < 0.001$ uncorrected at the voxel level but only clusters passing a small volume correction for multiple comparisons, 10
significance $p < 0.05$ were presented. Results are shown on axial sections of the Montreal Neurological Institute standard brain. Colo

Table 3
Regions of fMRI activity differences during the execution of the "FoG-observation-task" and "gait-observation-task" in patients with PD-FoG relative to healthy controls, and during the execution "FoG-observation-task" relative to the "gait-observation-task", respectively in patients with PD-FoG and in healthy controls.

	Area	BA	x	y	Z	T
"FoG-observation-task"						
PD-FoG vs HC	↓ R inferior frontal pars triangularis	45	48	34	0	4.21
	↓ R inferior frontal pars opercularis	44	50	6	26	4.16
	↓ R supramarginal	40	52	−44	46	4.77
	↓ L supramarginal	40	−52	−40	46	4.16
	↓ R SMA	6	18	0	68	3.41
"Gait-observation-task"						
PD-FoG vs HC	↓ L supramarginal	40	−60	−40	40	3.71
	↑ L hippocampus	54	−21	−13	−14	3.96
	↑ R hippocampus	54	24	−8	−20	3.86
	↑ R superior frontal	8	18	26	58	4.32
"FoG-observation-task" vs "Gait-observation-task"						
PD-FoG	↑ L anterior medial prefrontal cortex	10	−4	56	1	3.73
	↓ L angular	39	−38	−56	48	4.34
HC	↑ L hippocampus	54	−27	−12	−15	3.74
	↑ R superior frontal	9	8	46	38	4.62
	↑ R SMA	6	16	24	54	8.39
	↑ R middle frontal	8	26	28	40	4.62
	↑ L superior frontal	8	−18	26	48	5.08
	↓ L orbitofrontal	11	−44	43	11	4.29
	↓ L superior parietal	7	−28	−60	38	4.44
	↓ L inferior frontal	45	−46	34	28	5.27
"FoG-observation-task" vs "Gait-observation-task"						
PD-FoG vs HC	↓ L hippocampus	54	−16	−10	−15	4.40
	↓ R hippocampus	54	24	−9	−15	4.32
	↓ R superior frontal	8	18	25	56	3.96
"FoG-observation-task": negative correlation with FoG-Questionnaire						
PD-FoG	↓ L superior medial frontal	8	−2	28	42	4.25
"FoG-observation-task": negative correlation with TMT-B-A						
PD-FoG	↓ R supramarginal	40	48	−46	44	5.32

X, y, and z coordinates referred to the Montreal Neurological Institute (MNI) space.
Abbreviations: BA = Brodmann area; L = left; HC = healthy controls; PD-FoG = Parkinson's disease patients with freezing of gait; R = right; SMA = supplementary motor area; TMT-B-A = Trail Making Test B-A.

Gait-observation task

PD-FoG patients relative to healthy controls showed a reduced recruitment of the left supramarginal gyrus and an increased recruitment of the right [redacted] and bilateral hippocampu

Fog-observation vs gait observation tasks

In the "FoG-observation-task" relative to the "gait-observation-task", healthy controls revealed an increased recruitment of the dorsomedial prefrontal cortex including the right middle frontal cortex and bilateral superior frontal gyri, right SMA and left hippocampus and a reduced activity of left orbitofrontal, inferior frontal and superior [redacted]

FoG patients revealed an increased recruitment of the left anterior medial prefrontal cortex and a decreased activity of the left [redacted]

PD-FoG patients relative to healthy controls showed a reduced recruitment of the dorsomedial prefrontal cortex, including the right superior frontal gyrus, and the bilateral hippocampus

Tags: Empathy, Imaging, behavioral, Questionnaire

Brain functional connectivity patterns for emotional state classification in Parkinson’s disease patients without dementia

**Item Type**	Journal Article
**Author**	R. Yuvaraj
**Author**	M. Murugappan
**Author**	U. Rajendra Acharya
**Author**	Hojjat Adeli
**Author**	Norlinah Mohamed Ibrahim
**Author**	Edgar Mesquita
**Abstract**	Successful emotional communication is crucial for social interactions and social relationships. Parkinson’s Disease (PD) patients have shown deficits in emotional recognition abilities although the research findings are inconclusive. This paper presents an investigation of six emotions (happiness, sadness, fear, anger, surprise, and disgust) of twenty non-demented (Mini-Mental State Examination score >24) PD patients and twenty Healthy Controls (HCs) using Electroencephalogram (EEG)-based Brain Functional Connectivity (BFC) patterns. The functional connectivity index feature in EEG signals is computed using three different methods: Correlation (COR), Coherence (COH), and Phase Synchronization Index (PSI). Further, a new functional connectivity index feature is proposed using bispectral analysis. The experimental results indicate that the BFC change is significantly different among emotional states of PD patients compared with HC. Also, the emotional connectivity pattern classified using Support Vector Machine (SVM) classifier yielded the highest accuracy for the new bispectral functional connectivity index. The PD patients showed emotional impairments as demonstrated by a poor classification performance. This finding suggests that decrease in the functional connectivity indices during emotional stimulation in PD, indicating functional disconnections between cortical areas.
**Date**	2016
**URL**	https://www.sciencedirect.com/science/article/pii/S0166432815302503
**Volume**	298
**Pages**	248-260
**Publication**	Behavioural Brain Research
**DOI**	https://doi.org/10.1016/j.bbr.2015.10.036
**ISSN**	0166-4328
**Date Added**	6.7.2025, 19:12:35
**Modified**	5.9.2025, 15:02:13

Notes:

Not Included: Same behavioural resultsas study
(OEmotion classification in Parkinson's disease by higher-order spectra and power spectrum features using EEG signals: A comparative study)

Tags: EXCLUDED

Brain Networks and Cognitive Impairment in Parkinson's Disease.

Item Type Journal Article
Author Rosaria Rucco
Author Anna Lardone
Author Marianna Liparoti
Author Emahnuel Troisi Lopez
Author Rosa De Micco
Author Alessandro Tessitore
Author Carmine Granata
Author Laura Mandolesi
Author Giuseppe Sorrentino
Author Pierpaolo Sorrentino

Abstract Aim: The aim of the present study is to investigate the relationship between both functional connectivity and brain networks with cognitive decline, in patients with Parkinson's disease (PD). Introduction: PD phenotype is not limited to motor impairment but, rather, a wide range of non-motor disturbances can occur, with cognitive impairment being one of the most common. However, how the large-scale organization of brain activity differs in cognitively impaired patients, as opposed to cognitively preserved ones, remains poorly understood. Methods: Starting from source-reconstructed resting-state magnetoencephalography data, we applied the phase linearity measurement (PLM) to estimate functional connectivity, globally and between brain areas, in PD patients with and without cognitive impairment (respectively PD-CI and PD-NC), as compared with healthy subjects (HS). Further, using graph analysis, we characterized the alterations in brain network topology and related these, as well as the functional connectivity, to cognitive performance. Results: We found reduced global and nodal PLM in several temporal (fusiform gyrus, Heschl's gyrus, and inferior temporal gyrus), parietal (postcentral gyrus), and occipital (lingual gyrus) areas within the left hemisphere, in the gamma band, in PD-CI patients, as compared with PD-NC and HS. With regard to the global topological features, PD-CI patients, as compared with HS and PD-NC patients, showed differences in multi-frequencies bands (delta, alpha, gamma) in the Leaf fraction, Tree hierarchy (Th) (both higher in PD-CI), and Diameter (lower in PD-CI). Finally, we found statistically significant correlations between the Montreal Cognitive Assessment test and both the Diameter in delta band and the Th in the alpha band. Conclusion: Our work points to specific large-scale rearrangements that occur selectively in cognitively compromised PD patients and are correlated to cognitive impairment. Impact statement In this article, we want to test the hypothesis that the cognitive decline observed in Parkinson's disease (PD) patients may be related to specific changes of both functional connectivity and brain network topology. Specifically, starting from magnetoencephalography signals and by applying the phase linearity measurement (PLM), a connectivity metric that measures the synchronization between brain regions, we were able to highlight differences in the

global and nodal PLM values in PD patients with cognitive impairment as compared with both cognitively unimpaired patients and healthy subjects. Further, using graph analysis, we analyzed alterations in brain network topology that were related to cognitive functioning.

**Date**	2022 Jun
**Language**	eng
**Extra**	Place: United States
**Volume**	12
**Pages**	465-475
**Publication**	Brain connectivity
**DOI**	10.1089/brain.2020.0985
**Issue**	5
**Journal Abbr**	Brain Connect
**ISSN**	2158-0022 2158-0014
**PMID**	34269602
**Date Added**	6.7.2025, 19:09:42
**Modified**	5.9.2025, 14:54:21

Notes:

Not Included: Does not study SC

Tags: EXCLUDED

Brain structural MRI correlates of cognitive dysfunctions in Parkinson's disease.

**Item Type**	Journal Article
**Author**	Naroa Ibarretxe-Bilbao
**Author**	Carme Junque
**Author**	Maria J. Marti
**Author**	Eduardo Tolosa
**Abstract**	Cognitive dysfunction occurs at early stages of Parkinson's disease (PD). Initial studies reported that cognitive dysfunction in early PD only affected fronto-striatal circuits, provoking a marked executive dysfunction. Memory impairment in PD was thought to depend on a problem in retrieving stored information, therefore also reflecting a fronto-striatal dysfunction. However, there is increasing structural MRI evidence of medial temporal lobe atrophy in PD, which may be responsible for memory dysfunction. Other neuropsychological functions usually impaired in PD are semantic fluency, visuoperceptual and visuospatial functions, decision-making and recognition of facial emotions; and impairments in these functions are associated with cortical structural changes assessed by MRI. Overall, although the literature on the topic is scarce, there is increasing evidence of brain structural changes, detectable by MRI, which can explain the neuropsychological deficits early in the clinical disease course before dementia develops. In this review, we summarize the papers that have used structural MRI to study the neuroanatomical correlates of cognitive dysfunctions in PD.

Date 2011 Nov 15
Language eng
License Copyright © 2011 Elsevier B.V. All rights reserved.
Extra Place: Netherlands
Volume 310
Pages 70-74
Publication Journal of the neurological sciences
DOI 10.1016/j.jns.2011.07.054
Issue 1-2
Journal Abbr J Neurol Sci
ISSN 1878-5883 0022-510X
PMID 21864849
Date Added 6.7.2025, 19:09:42
Modified 5.9.2025, 14:39:50

Notes:

Included – Systematic review

sample characteristics

A systematic literature search using MEDLINE database was carried out on papers published in English between 1998 and March 2011. We included the studies in PD that performed: i) analyses of T1-weighted structural MRI brain data; ii) neuropsychological assessment; and iii) correlation analyses between the neuropsychological data and structural MRI brain data.

Main findings related to the review's scope

Several studies have reported impairment of recognition of facial expressions of emotions in PD [21], [29], [30], [31], [32], [33]. Specifically, Sprengelmeyer et al. [33] reported that de novo PD patients scored significantly lower in recognizing sadness and fear than healthy controls. These results suggested that recognition of these emotions is impaired very early in the disease and is independent of treatment. In a group of early PD patients we found atrophy of bilateral OFC and amygdala using VBM [21]. The bilateral OFC atrophy correlated with the scores in the Ekman 60 faces test, a measure of recognition of facial emotions, in the PD group. Another MRI study [34] also reported degeneration of the OFC in early PD patients but no significant correlations between the behavioral tests and the imaging data were found.

Tags: Emotion recognition, Imaging

Changes in emotion processing in early Parkinson's disease reflect disease progression.

Item Type Journal Article
Author Allison Eriksson
Author Panagiota Tsitsi
Author Mikkel C. Vinding

Author Martin Ingvar
Author Per Svenningsson
Author Daniel Lundqvist
Abstract OBJECTIVE: Parkinson's disease (PD) is a neurodegenerative disorder which can substantially affect nonmotor functions related to emotional processing. We aimed to examine the underlying differences in emotional processing in PD by comparing how early-stage PD patients recognize, rate, and react to facial, bodily, and vocal emotional stimuli to that of healthy controls (HC). METHOD: We compared emotion recognition, emotional rating bias, and emotional response range between a PD patient group (n = 33) and a HC group (n = 29). Pearson's correlations were conducted to evaluate the relationship between emotion processing measures and clinical outcome measures in each group. RESULTS: PD patients showed an enhanced emotion processing as compared to HC. They were overall more accurate than HC's at identifying correct emotions and furthermore showed an increase in emotional ratings and reactions to both positive and negative stimuli that scaled with increased symptom severity, thereby yielding significant correlations between clinical outcomes and emotional range in the PD patient group. CONCLUSION: Our results suggest that alterations in emotional processing reflect disease progression in early PD. (PsycInfo Database Record (c) 2022 APA, all rights reserved).
Date 2022 Mar
Language eng
Extra Place: United States
Volume 36
Pages 206-215
Publication Neuropsychology
DOI 10.1037/neu0000794
Issue 3
Journal Abbr Neuropsychology
ISSN 1931-1559 0894-4105
PMID 35377692
Date Added 6.7.2025, 19:09:36
Modified 5.9.2025, 14:35:09

Notes:

Included

sample characteristics

size: 33 PD and 29 HC

Parkinson's Disease type and duration: idiopathic PD, NA

Medication: on medication

Hoehn-Yahr: M= 1.58 SD=0.8

UPDRS-3: M= 17.3 SD=10.5

Gender (male): 18 males (55%)

averaged ages (SD, range): M= 63.5 SD=9.5

other neurological disease (tumor, stroke, etc.): None

other major psychopathology: None

origin country (or ethnicity): Sweden

method observational

instruments used in order to quantify the variables

Social cognition aspect: emotion recognition

Name of the task: The Emotion Survey

type of stimulus [face/voice etc., Ekman faces/other etc.]: 46 short video or sound clips from the European Union (EU)-Emotion Stimulus Set (Lassalle et al., 2019; O'Reilly et al., 2016) showing actors portraying positive and negative emotions using three modalities—facial expressions (Face), body language (Body), or sound clips (Voice).

task condition: Positive emotions included happiness, pride, joking, interest, excitement, and surprise. Negative emotions consisted of disgust, shame, fear, sadness, hurt, anger, worry, frustration, and boredom

operationalization: (a) correct identification of the emotion portrayed (b) ratings of valence and arousal in response to the emotion.

tested beyond type of stimuli and grouped emotion into positive/negative

Main findings related to the review's scope

Accuracy: in positive, the mean score for the PD patient group was sig higher than the mean HC group score. in negative, no sig dif

Tags: Emotion recognition, behavioral

Changes in Subjective Cognitive and Social Functioning in Parkinson's Disease from Before to During the COVID-19 Pandemic

**Item Type**	Journal Article
**Author**	Nishaat Mukadam
**Author**	Shraddha B. Kinger
**Author**	Sandy Neargarder
**Author**	Robert D. Salazar
**Author**	Celina Pluim Mcdowell
**Author**	Juliana Wall
**Author**	Rini I. Kaplan
**Author**	Alice Cronin-Golomb
**Date**	2025-01
**Volume**	13
**Publication**	HEALTHCARE
**DOI**	10.3390/healthcare13010070
**Issue**	1
**Date Added**	14.7.2025, 14:48:38
**Modified**	5.9.2025, 14:47:37

Notes:

Not Included: No control group.

Tags: EXCLUDED

Chapter 6 - Facial emotion recognition in Parkinson's disease: methodological, clinical, and pathophysiological factors

**Item Type**	Book Section
**Editor**	Colin R. Martin
**Editor**	Victor R. Preedy
**Author**	Marco De Risi
**Author**	Enrica Olivola
**Author**	Giancarlo Di Gennaro
**Author**	Nicola Modugno
**Abstract**	In the last decade the influence of nonmotor symptoms on the motor performance of Parkinson's disease (PD) patients has been the subject of several studies. In this chapter we focus on the role played by emotional processes that have been studied through research conducted in laboratory or clinical settings. The studies reviewed in this chapter clearly show how PD negatively influences the affective life of patients. More specifically, research on emotional processes and expressions suggests that PD patients may exhibit significant deficits in nonverbal communication, with difficulties in producing emotional facial movements and recognizing the emotions of other people. Several studies demonstrate how patients with PD may display deficits in performing emotion recognition tasks; this seems particularly true for experimental

tasks involving prosodic stimuli with a negative valence. Other studies have shown mood alterations and emotional dysfunctions including depression, apathy, and anxiety as well as alexithymia.

Date 2020
URL https://www.sciencedirect.com/science/article/pii/B9780128159460000065
Extra DOI: https://doi.org/10.1016/B978-0-12-815946-0.00006-5
Publisher Academic Press
ISBN 978-0-12-815946-0
Pages 91-106
Book Title Diagnosis and Management in Parkinson's Disease
Date Added 6.7.2025, 19:12:37
Modified 5.9.2025, 14:52:57

Notes:

No Included: a book chapter

Tags: EXCLUDED

Chapter Seven - Imaging the Nonmotor Symptoms in Parkinson's Disease

Item Type Book Section
Editor K. Ray Chaudhuri
Editor Nataliya Titova
Author Tayyabah Yousaf
Author Heather Wilson
Author Marios Politis
Abstract Parkinson's disease is acknowledged to be a multisystem syndrome, manifesting as a result of multineuropeptide dysfunction, including dopaminergic, cholinergic, serotonergic, and noradrenergic deficits. This multisystem disorder ultimately leads to the presentation of a range of nonmotor symptoms, now appreciated to be an integral part of the disease-specific spectrum of symptoms, often preceding the diagnosis of motor Parkinson's disease. In this chapter, we review the dopaminergic and nondopaminergic basis of these symptoms by exploring the neuroimaging evidence based on several techniques including positron emission tomography, single-photon emission computed tomography molecular imaging, magnetic resonance imaging, functional magnetic resonance imaging, and diffusion tensor imaging. We discuss the role of these neuroimaging techniques in elucidating the underlying pathophysiology of NMS in Parkinson's disease.
Date 2017
URL https://www.sciencedirect.com/science/article/pii/S0074774217300454
Extra DOI: https://doi.org/10.1016/bs.irn.2017.05.001
Volume 133
Publisher Academic Press
Pages 179-257
Series International Review of Neurobiology
Book Title Nonmotor Parkinson's: The Hidden Face

ISSN 0074-7742
Date Added 6.7.2025, 19:12:34
Modified 5.9.2025, 15:01:53

Notes:

Not Included: Not a study; Overview chapter
Tags: EXCLUDED

Chapter Thirteen - Structural MRI in Idiopathic Parkinson's Disease

Item Type Book Section
Editor Marios Politis
Author Rosa De Micco
Author Antonio Russo
Author Alessandro Tessitore
Abstract Among modern neuroimaging modalities, magnetic resonance imaging (MRI) is a widely available, non-invasive, and cost-effective method to detect structural and functional abnormalities related to neurodegenerative disorders. In the last decades, MRI have been widely implemented to support PD diagnosis as well as to provide further insights into motor and non-motor symptoms pathophysiology, complications and treatment-related effects. Different aspects of the brain morphology and function may be derived from a single scan, by applying different analytic approaches. Biomarkers of neurodegeneration as well as tissue microstructural changes may be extracted from structural MRI techniques. In this chapter, we analyze the role of structural imaging to differentiate PD patients from controls and to define neural substrates of motor and non-motor PD symptoms. Evidence collected in the premotor PD phase will be also critically discussed. White matter as well as gray matter integrity imaging studies has been reviewed, aiming to highlight points of strength and limits to their potential application in clinical settings.
Date 2018
URL https://www.sciencedirect.com/science/article/pii/S0074774218300783
Extra DOI: https://doi.org/10.1016/bs.irn.2018.08.011
Volume 141
Publisher Academic Press
Pages 405-438
Series International Review of Neurobiology
Book Title Imaging in Movement Disorders: Imaging Methodology and Applications in Parkinson's Disease
ISSN 0074-7742
Date Added 6.7.2025, 19:12:35
Modified 5.9.2025, 14:46:44

Notes:

Not Included: No SC aspect
Tags: EXCLUDED

Characteristics of facial expression recognition ability in patients with Lewy body disease

**Item Type**	Journal Article
**Author**	Yuriko Kojima
**Author**	Tomohiro Kumagai
**Author**	Tomoo Hidaka
**Author**	Takeyasu Kakamu
**Author**	Shota Endo
**Author**	Yayoi Mori
**Author**	Tadashi Tsukamoto
**Author**	Takashi Sakamoto
**Author**	Miho Murata
**Author**	Takehito Hayakawa
**Author**	Tetsuhito Fukushima
**Abstract**	Background: The facial expression of medical staff has been known to greatly affect the psychological state of patients, making them feel uneasy or conversely, cheering them up. By clarifying the characteristics of facial expression recognition ability in patients with Lewy body disease, the aim of this study is to examine points to facilitate smooth communication between caregivers and patients with the disease whose cognitive function has deteriorated. Methods: During the period from March 2016 to July 2017, we examined the characteristics of recognition of the six facial expressions of “happiness,” “sadness,” “fear,” “anger,” “surprise,” and “disgust” for 107 people aged 60 years or more, both outpatient and inpatient, who hospital specialists had diagnosed with Lewy body diseases of Parkinson's disease, Parkinson's disease with dementia, and dementia with Lewy bodies. Based on facial expression recognition test results, we classified them by cluster analysis and clarified features of each type. Results: In patients with Lewy body disease, happiness was kept unaffected by aging, age of onset, duration of the disease, cognitive function, and apathy; however, recognizing the facial expression of fear was difficult. In addition, due to aging, cognitive decline, and apathy, the facial expression recognition ability for sadness and anger decreased. In particular, cognitive decline reduced recognition of all of the facial expressions except for happiness. The test accuracy rates were classified into three types using the cluster analysis: “stable type,” “mixed type,” and “reduced type”. In the “reduced type”, the overall facial recognition ability declined except happiness, and in the mixed type, recognition ability of anger particularly declined. Conclusion: There were several facial expressions that the Lewy body disease patients were unable to accurately identify. Caregivers are recommended to make an effort to compensate for such situations with language or body contact, etc., as a way to convey correct feeling to the patients of each type.
**Date**	2018-07-18
**Language**	English
**Extra**	Place: ONE NEW YORK PLAZA, SUITE 4600, NEW YORK, NY, UNITED STATES Type: Article

Volume 23
Publisher SPRINGER
Publication ENVIRONMENTAL HEALTH AND PREVENTIVE MEDICINE
DOI 10.1186/s12199-018-0723-2
Issue 1
ISSN 1342-078X
Date Added 14.7.2025, 14:50:34
Modified 5.9.2025, 14:42:09

Notes:

Not Included: not in English
Tags: EXCLUDED

Clinical and structural brain correlates of hypomimia in early-stage Parkinson's disease

Item Type Journal Article
Author Frederic Sampedro
Author Saul Martinez-Horta
Author Andrea Horta-Barba
Author Michel J. Grothe
Author Miguel A. Labrador-Espinosa
Author Silvia Jesus
Author Astrid Adarmes-Gomez
Author Fatima Carrillo
Author Arnau Puig-Davi
Author Florinda Roldan-Lora
Author Miquel Aguilar-Barbera
Author Pau Pastor
Author Sonia Escalante Arroyo
Author Berta Solano Vila
Author Anna Cots-Foraster
Author Javier Ruiz-Martinez
Author Francisco Carrillo-Padilla
Author Mercedes Pueyo-Morlans
Author Isabel Gonzalez-Aramburu
Author Jon Infante-Ceberio
Author Jorge Hernandez-Vara
Author Oriol de Fabregues-Boixar
Author Teresa de Deus Fonticoba
Author Asuncion Avila
Author Juan Carlos Martinez-Castrillo

Author Helena Bejr-Kasem
Author Antonia Campolongo
Author Berta Pascual-Sedano
Author Pablo Martinez-Martin
Author Diego Santos-Garcia
Author Pablo Mir
Author Pedro J. Garcia-Ruiz
Author Jaime Kulisevsky
Author COPPADIS Study Grp
Abstract Background and purpose Reduced facial expression of emotions is a very frequent symptom of Parkinson's disease (PD) and has been considered part of the motor features of the disease. However, the neural correlates of hypomimia and the relationship between hypomimia and other non-motor symptoms of PD are poorly understood. Methods The clinical and structural brain correlates of hypomimia were studied. For this purpose, cross-sectional data from the COPPADIS study database were used. Age, disease duration, levodopa equivalent daily dose, Unified Parkinson's Disease Rating Scale part III (UPDRS-III), severity of apathy and depression and global cognitive status were collected. At the imaging level, analyses based on gray matter volume and cortical thickness were used. Results After controlling for multiple confounding variables such as age or disease duration, the severity of hypomimia was shown to be indissociable from the UPDRS-III speech and bradykinesia items and was significantly related to the severity of apathy (beta = 0.595; p < 0.0001). At the level of neural correlates, hypomimia was related to motor regions brodmann area 8 (BA 8) and to multiple fronto-temporo-parietal regions involved in the decoding, recognition and production of facial expression of emotions. Conclusion Reduced facial expressivity in PD is related to the severity of symptoms of apathy and is mediated by the dysfunction of brain systems involved in motor control and in the recognition, integration and expression of emotions. Therefore, hypomimia in PD may be conceptualized not exclusively as a motor symptom but as a consequence of a multidimensional deficit leading to a symptom where motor and non-motor aspects converge.
Date 2022-12
Language English
Extra Place: 111 RIVER ST, HOBOKEN 07030-5774, NJ USA Type: Article
Volume 29
Publisher WILEY
Pages 3720-3727
Publication EUROPEAN JOURNAL OF NEUROLOGY
DOI 10.1111/ene.15513
Issue 12
ISSN 1351-5101
Date Added 14.7.2025, 14:50:29
Modified 5.9.2025, 14:54:56

Notes:

Not Included: not on SC

Tags: EXCLUDED

Cognition and Other Non-Motor Symptoms in an At-Risk Cohort for Parkinson's Disease Defined by REM-Sleep Behavior Disorder and Hyposmia

**Item Type**	Journal Article
**Author**	Laure Pauly
**Author**	Armin Rauschenberger
**Author**	Claire Pauly
**Author**	Valerie E. Schroder
**Author**	Gilles Van Cutsem
**Author**	Anja K. Leist
**Author**	Rejko Kruger
**Author**	NCER-PD Consortium
**Date**	2024
**Volume**	14
**Pages**	545-556
**Publication**	JOURNAL OF PARKINSONS DISEASE
**DOI**	10.3233/JPD-230285
**Issue**	3
**ISSN**	1877-7171
**Date Added**	14.7.2025, 14:48:39
**Modified**	5.9.2025, 14:49:32

Notes:

Not Included: No study of SC

Tags: EXCLUDED

Cognition and the inhibitory control of saccades in schizophrenia and Parkinson's disease

**Item Type**	Conference Paper
**Author**	TJ Crawford
**Author**	D Bennett
**Author**	G Lekwuwa
**Author**	S Shaunak
**Author**	JFW Deakin
**Editor**	J Hyona
**Editor**	DP Munoz
**Editor**	W Heide
**Editor**	R Radach
**Abstract**	Historically, various lines of evidence have converged on the view that the brain expends much of its neural resources on inhibiting its own activity in a critical step towards the cognitive control of behaviour. The loss of inhibitory control is

widely reported in neurological and psychiatric disorders; however, the consequences of reduced inhibition in terms of wider cognitive effects on cognitive control operations such as planning, abstract thought, working memory and the ability to appreciate the perspective of others ('theory of mind') has been widely overlooked. The antisaccade paradigm examines the conflict between a prepotent stimulus that produces a powerful urge to fixate the target, and the overriding goal to `look' in the opposite direction. In this chapter we illustrate how this paradigm is increasingly used to explore the relationship of inhibitory control and cognition in Parkinson's disease, schizophrenia and healthy participants. Evidence is presented that is consistent with the theory of cognitive inhibition as a distinct process that can be dissociated from working memory. We conclude that the inhibitory control of saccadic eye movement should be studied in the wider context of cognitive operations.

**Date**	2002
**Language**	English
**Extra**	Type: Review
**Volume**	140
**Place**	SARA BURGERHARTSTRAAT 25, PO BOX 211, 1000 AE AMSTERDAM, NETHERLANDS
**Publisher**	ELSEVIER SCIENCE BV
**ISBN**	0-444-51097-4
**Pages**	449-466
**Series**	Progress in Brain Research
**Proceedings Title**	BRAIN'S EYE: NEUROBIOLOGICAL AND CLINICAL ASPECTS OF OCULOMOTOR RESEARCH
**ISSN**	0079-6123
**Date Added**	14.7.2025, 14:50:43
**Modified**	14.7.2025, 14:50:43

Notes:

Not Included: no relevant results

Tags: EXCLUDED

Cognitive and Affective Functioning in Parkinson's Disease Patients with Lateralized Motor Signs

**Item Type**	Journal Article
**Author**	John St. Clair
**Author**	Joan C. Borod
**Author**	Martin Sliwinski
**Author**	Lucien J. Cote
**Author**	Yaakov Stern
**Date**	06/1998
**Language**	en
**Library Catalog**	DOI.org (Crossref)

URL https://www.tandfonline.com/doi/full/10.1076/jcen.20.3.320.820
Accessed 11.8.2025, 15:57:23
Volume 20
Pages 320-327
Publication Journal of Clinical and Experimental Neuropsychology
DOI 10.1076/jcen.20.3.320.820
Issue 3
Journal Abbr Journal of Clinical and Experimental Neuropsychology
ISSN 1380-3395, 1744-411X
Date Added 11.8.2025, 15:57:23
Modified 11.8.2025, 15:57:23

Notes:

Included

sample characteristics

size: 12 LPD 11 RPD and 11 HC (Age, gender, education and MMSE matched)

Parkinson's Disease type and duration: Idiopathic PD, LPD Mduration=3.7 SD=2.1 LPD Mduration=3.6 SD=2.1

Medication: on medication

Hoehn-Yahr: LPD M=2.4 SD=0.5 RPD M=2 SD=0

UPDRS-3: LPD M=3.8 SD=2.0 RPD M=4.3 SD=1.3

Gender (male): LPD 7 males (58%) RPD 5 males (45%)

averaged ages (SD, range): LPD M=72.5 SD=10.2 RPD M=66.6 SD=8.5

other neurological disease (tumor, stroke, etc.): None

other major psychopathology: None

origin country (or ethnicity): NA

method observational

instruments used in order to quantify the variables

Social cognition aspect: emotion recognition

Name of the task: Facial Emotion

type of stimulus [face/voice etc., Ekman faces/other etc.]: NA - Drawn from

Borod, J., Martin, C., Alpert, M., Brozgold, A., & Welkowitz, J. (1993). Perception of facial emotion in schizophrenic and right brain-damaged patients. The Journal of Nervous and Mental Disease, 181, 494-502.

task conditions: NA

operationalization: accuracy

Main findings related to the review's scope

No significant differences between groups

Tags: Emotion recognition, behavioral

Cognitive and affective theory of mind deficits in Parkinson's disease

Item Type Journal Article
Author M. Kralova

Author K. Sujanska
Author Z. Csefalvay
Author J. Markova
Author M. Papayova
Author A. Kusnirova
Author Z. Kosutzka
Author P. Valkovic
Author M. Hajduk
Date 2019
URL https://www.sciencedirect.com/science/article/pii/S0022510X19317125
Volume 405
Pages 232
Publication Journal of the Neurological Sciences
DOI https://doi.org/10.1016/j.jns.2019.10.1238
ISSN 0022-510X
Date Added 6.7.2025, 19:12:37
Modified 6.7.2025, 19:12:37

Notes:

Not Included: Not an empirical ms
Tags: EXCLUDED

Cognitive and Affective Theory of Mind in Mild Cognitive Impairment and Parkinson's Disease: Preliminary Evidence from the Italian Version of the Yoni Task.

Item Type Journal Article
Author Federica Rossetto
Author Ilaria Castelli
Author Francesca Baglio
Author Davide Massaro
Author Margherita Alberoni
Author Raffaello Nemni
Author Simone Shamay-Tsoory
Author Antonella Marchetti
Abstract The aim of the study was to explore cognitive and affective dimensions of ToM using the computerized Yoni task in participants with amnestic Mild Cognitive Impairment (aMCI=16), early stage of Parkinson's Disease (PD=14), and healthy controls (HC=18) Results demonstrated that the Yoni task was effective in discriminating between groups in 1(th) order cognitive dimension (MCI<PD=HC, p(corr)<.05), and in 2(nd) order cognitive and affective dimensions (MCI<HC, p(corr) <.05), highlighting a reduced ToM performance also in people with PD (MCI<PD<HC, p(corr) <.05). Thus, the Yoni task represents a sensitive tool for detecting different dimensions of ToM impairment, across different clinical

conditions.

Date 2018
Language eng
Extra Place: England
Volume 43
Pages 764-780
Publication Developmental neuropsychology
DOI 10.1080/87565641.2018.1529175
Issue 8
Journal Abbr Dev Neuropsychol
ISSN 1532-6942
PMID 30299987
Date Added 6.7.2025, 19:09:41
Modified 5.9.2025, 14:54:01

Notes:

Included

Sample characteristics

Size: 16 aMCI (amnestic MCI), 14 PD, 18 HC (age-matched)

PD-type: NA

PD-duration: NA

Medication: Assessment in ON

Hoehn-Yahr: < 2.5; Median = 1, Range = 1-2.5

UPDRS-3: M = 20.14, SD = 15.17

Gender (male): 13 (93%)

Age: M = 68.21, SD = 7.96

Other neurological disease (tumor, stroke, etc.): none

Other major psychopathology: none

Origin country (or ethnicity): Italy

method behavioural

instruments used in order to quantify the variables

Paper and Pencil Battery (multiple subtests)

Social cognition aspect: ToM

Name of the task: Deceptive Box Task

Type of stimulus [face/voice etc., Ekman faces/other etc.]: A closed box of candies is shown to the participant, the content of which has been previously substituted with staples

Task condition: content of box is shown then close. Participant needes to predict what another person would say if shown box

Operationalization: Each question is scored 1 if the answer is correct and 0 if the answer is wrong (range 0-5)

Social cognition aspect: ToM

Name of the task: Look-Prediction and the Say-Prediction tasks

Type of stimulus [face/voice etc., Ekman faces/other etc.]: The participant has to predict where a character in the story thinks another character would look for a hidden object (look-prediction) or what a character thinks the other one would say about a hidden object (say-prediction

Task condition: Both tasks require participants to answer a total of five questions: two control questions (one memory item and one reality item) and three mentalistic questions

Operationalization: Each question is scored 1 if the answer is correct and 0 if the answer is wrong

(range 0-5).

Social cognition aspect: ToM affective

Name of the task: Reading the Mind in the Eyes test (RME)

Type of stimulus [face/voice etc., Ekman faces/other etc.]: 36 pictures of the eye region taken from different human faces

Task condition: Participants have to infer what the character is feeling and choose a word that describes the character's mental state from four mental states written under each picture

Operationalization:Each item is scored 1 if the answer is correct and 0 if the answer is wrong (range 0-36);

Social cognition aspect: ToM

Name of the task: Strange Stories task

Type of stimulus [face/voice etc., Ekman faces/other etc.]: 4 short stories

Task condition: answer questions to stories

Operationalization: Each question received a score of 0 for wrong answers, 1for partially correct/ incomplete answers and 2 for correct answers (range 0-2 for each question). The global scores of the four "ToM stories" and of the four physical stories ranged from 0 to 8

Social cognition aspect: ToM (cognitive and affective)

Name of the task: Yoni-Task

Type of stimulus [face/voice etc., Ekman faces/other etc.]: 98 trials, each showing a face named "Yoni" ("Gianni" in the Italian version of the task) and four colored pictures surrounding the face, one in each corner of the screen, and referring to various semantic categories (for example, fruit, animals, chairs, means of transport) or faces

Task condition: The participant is required to choose the correct image to which Yoni is referring based on a sentence that appears on the top of the screen and on some available cues, such as Yoni's eye gaze or facial expression or the eye gaze/facial expression of faces around him. Participants were instructed to choose the answer they thought to be correct by pointing to it with the computer mouse as fast as they could. Only one of the four alternatives is correc

Operationalization: Each item was scored 1 if the answer was correct and 0 if the answer was wrong. Thus, the total score on the Yoni task (Yoni TOT) ranged from 0 to 98. For each participant, the scores gained from each sub-category were summed in order to obtain four subtotals: the total of first-order cognitive items (COG1, range 0-12), the total of second-order cognitive items (COG2, range 0-24), the total of first-order affective items (AFF1, range 0-12) and the total of second-order affective items (AFF2, range 0-36). Also RTs

Main findings related to the review's scope

Paper pencil ToM tasks

Our results show no significant differences among groups in the Deceptive Box task, in the Look Prediction task and in the Say-Prediction task

we found significant between group differences in the most advanced ToM tasks, i.e. in the RME test and in the Strange Stories task. In particular, pairwise comparisons revealed that the MCI group had lower performance than the HC group both on the RME test and on the Strange Stories task, while no significant differences emerged between the two clinical groups (MCI and PD) and between the PD group and the HC group

Yoni Task

a significant between-group difference emerged on the total score of the Yoni task. In particular, the MCI group scored lower compared to the HC group, while no differences emerged between the PD group and the HC group and between the two clinical groups

While no between-group differences emerged on the first-order affective items (AFF1), we found significant between-group differences on the second-order affective items. In particular, the MCI group scored lower compared to the HC group, while no significant differences emerged between the two clinical groups and between the PD group and the HC group.

As for the first-order cognitive items (COG1), we found a significant difference across groups. In particular, the MCI group exhibited significantly lower performance compared to both the HC group and the PD group, while no differences emerged between the PD group and the HC group.

The results obtained for second-order cognitive items (COG2) are similar to those for second-order affective items reported above. Significant differences emerged between the groups, with the MCI group scoring lower than the HC group, while no significant differences emerged between the two clinical groups and between the PD group and the HC group.

Our results showed no significant differences in the RTs across groups, both in the affective/cognitive first-order items (AFF1, p=.39; COG1, p=.11), and in the affective/cognitive second-order items (AFF2, p=.14; COG2, p=.30) of the Yoni task

Tags: ToM, behavioral

Cognitive and affective Theory of Mind in neurodegenerative diseases: neuropsychological, neuroanatomical and neurochemical levels.

Item Type Journal Article

Author Michele Poletti

Author Ivan Enrici

Author Mauro Adenzato

Abstract The paper reviews of all of the current evidence on Theory of Mind (ToM) abilities in patients with neurodegenerative diseases. ToM refers to the abilities to attribute mental states to others. Two neural systems are involved in processing other people's beliefs and intentions (cognitive component) and others' emotions and feelings (affective component). We hypothesize that patients with different neurodegenerative diseases may present different patterns of ToM deficits on the basis of how different neuropathological processes affect the neural bases of ToM components during the progression of a disease. The studies we reviewed provided evidence of a deficit of the cognitive ToM component in cortical (Alzheimer's disease and frontotemporal dementia) and frontal-subcortical (amyotrophic lateral sclerosis and basal ganglia disorders) neurodegenerative diseases. As regards the affective ToM component, it resulted markedly impaired in frontotemporal dementia; it also resulted that performances in tasks assessing this process are heterogeneous in Parkinson's disease

and amyotrophic lateral sclerosis. The findings presented support the opportunity to introduce validated ToM tasks in the neuropsychological assessment of neurodegenerative diseases.

Date 2012 Oct
Language eng
License Copyright © 2012 Elsevier Ltd. All rights reserved.
Extra Place: United States
Volume 36
Pages 2147-2164
Publication Neuroscience and biobehavioral reviews
DOI 10.1016/j.neubiorev.2012.07.004
Issue 9
Journal Abbr Neurosci Biobehav Rev
ISSN 1873-7528 0149-7634
PMID 22819986
Date Added 6.7.2025, 19:09:40
Modified 5.9.2025, 14:51:50

Notes:

Not Included: Not a systematic Review (Literature Review)

Tags: Excluded

Cognitive Deficits in Early Parkinson's Disease: New Areas of Research

Item Type Journal Article
Author Sarah L. Mason
Author Roger A. Barker
Abstract Cognitive deficits in Parkinson's disease (PD) are heterogeneous, including in the early stages of the disease. The prognostic value of early cognitive deficits is still under debate, but there is particular interest in identifying deficits that are capable of predicting patients at risk of developing dementia. To date, research has focused on four neuropsychological domains (executive/attention, visuo-spatial, memory, and language), but there is recent evidence that impairment may extend beyond this, in particular into the field of social neuroscience. This review will briefly discuss the cognitive impairments in early PD, including the recent evidence for deficits in theory of mind, before evaluating the diagnostic criteria for, and utility of, the term “mild cognitive impairment” in PD.
Date 2012-03
Language English
Extra Place: 233 SPRING ST, NEW YORK, NY 10013 USA Type: Article
Volume 1
Publisher SPRINGER
Pages 39-44
Publication CURRENT GERIATRICS REPORTS
DOI 10.1007/s13670-011-0003-0

Issue 1
ISSN 2196-7865
Date Added 14.7.2025, 14:50:40
Modified 5.9.2025, 14:45:42

Notes:

Not Included: not a systematic review
Tags: EXCLUDED

Cognitive phenotypes in Parkinson’s disease: A latent profile analysis.

Item Type Journal Article
Author Edoardo Barvas
Author Giulia Mattavelli
Author Francesca Zappini
Author Floriana Giardina
Author Donatella Ottaviani
Author Costanza Papagno
Date 05/2021
Language en
Short Title Cognitive phenotypes in Parkinson’s disease
Library Catalog Crossref
URL https://doi.apa.org/doi/10.1037/neu0000737
Accessed 13.7.2025, 20:21:18
Volume 35
Publisher American Psychological Association (APA)
Pages 451-459
Publication Neuropsychology
DOI 10.1037/neu0000737
Issue 4
ISSN 1931-1559, 0894-4105
Date Added 13.7.2025, 20:21:18
Modified 5.9.2025, 14:27:49

Notes:

Not Included- no comparison group

however - important to address, indicate individual differences in ER.

sample characteristics

size: 65 PD

Parkinson's Disease type and duration: idiopathic PD, Mduration= 7.37 SD= 5.25

Medication: on medication

Hoehn-Yahr: Md=2

UPDRS-3: M= 17.8 SD= 9.33

Gender (male): 42 males (65%)

averaged ages (SD, range): M= 67.94 SD= 7.49

other neurological disease (tumor, stroke, etc.): None

other major psychopathology: None

origin country (or ethnicity): Italy

method observational

instruments used in order to quantify the variables

CLUSTER ANALYSIS – NEUROPSYCHOLOGICAL BUTTERY

Social cognition aspect: emotion recognition

Name of the task: Ekman 60-Faces Test (EK-60 F)

type of stimulus [face/voice etc., Ekman faces/other etc.]: Ekman faces

operationalization: accuracy

Main findings related to the review's scope

Three clusters were extracted, differentiated, among other things, in ER.

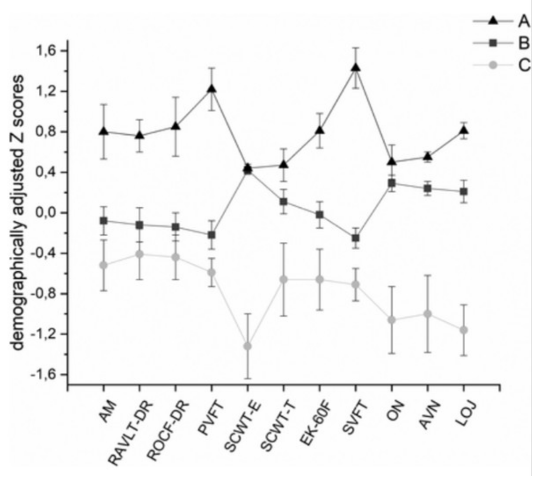

all scores were adjusted for age, education, and, when indicated, gender according to normative Italian data.

Comparisons of clinical and demographical variables across the three clusters revealed differences in the global cognitive functioning as measured by the MoCA (with a higher score for Cluster A than Cluster B and for Cluster B than Cluster C) and ADL-IADL functioning (with Cluster C reaching lower scores than Cluster A).

Cluster A (*n* = 14, 21.54%)

Cluster B (*n* = 35, 53.85%)

Cluster C (*n* = 16, 24.61%)

Tags: EXCLUDED

Cognitive profiles and optimal cut-offs for routine cognitive tests in elderly individuals with Parkinson's disease, Parkinson's disease dementia, Alzheimer's disease, and normal cognition

Item Type Journal Article
Author Harisd Phannarus

Author Weerasak Muangpaisan
Author Pitiporn Siritipakorn
Author Wattanachai Chotinaiwattarakul
Abstract Aim The cognitive impairment seen in Parkinson's disease (PD) results in patient disability and reduced quality of life. However, using cognitive screening scales specific to PD in routine clinical practice is difficult because of limited time, resources, and skills. We studied the ability of routine cognitive tests to differentiate between Parkinson's disease dementia (PDD) and PD and among the neuropsychological profiles of elderly individuals with PD, PDD, Alzheimer's disease (AD), and normal cognition. Methods This cross-sectional study involved 124 subjects. Subjects were 35 cognitively normal elderly and 37 elderly individuals with PD, 22 with PDD, and 30 with AD. All subjects were diagnosed by a specialist using standard criteria. Clinically relevant data and scores from the Montreal Cognitive Assessment and the Thai Mental State Examination were collected. Cognitive test scores were compared among groups. Receiver operating characteristic curves were constructed for a range of cut-off points to explore the sensitivity and specificity of the screening tools to detect PDD. Results There were 74 female subjects (59.7%), and the average age of all subjects was 75.6 years. The median score on the modified Hoehn and Yahr scale was 2.5 in subjects with PD and 4 in those with PDD ($P < 0.001$). The cut-offs for differentiating PDD from PD were 25 on the Thai Mental State Examination and 14 on the Montreal Cognitive Assessment. The sensitivity of the Thai Mental State Examination was 78.4%, and the specificity was 66.7% (area under the curve: 0.828). The sensitivity of the Montreal Cognitive Assessment was 81.1%, and the specificity was 75% (area under the curve: 0.876). There was a significant difference in the memory and language subdomains between AD and PDD ($P < 0.05$). Conclusions The cut-offs used to differentiate PDD from PD were not the same as routine cut-offs in distinguishing AD from normal elderly. The cognitive profile deficit in PDD differed from that in AD. Interpretations of positive screenings test should take this finding into consideration.
Date 2020-01
Language English
Extra Place: 111 RIVER ST, HOBOKEN 07030-5774, NJ USA Type: Article
Volume 20
Publisher WILEY
Pages 20-27
Publication PSYCHOGERIATRICS
DOI 10.1111/psyg.12451
Issue 1
ISSN 1346-3500
Date Added 14.7.2025, 14:50:32
Modified 5.9.2025, 14:51:02

Notes:

Not Included: Doesn’t study SC

Interesting topic though.

Tags: EXCLUDED

Comparison of structural connectivity in Parkinson's disease with depressive symptoms versus non-depressed: a diffusion MRI connectometry study.

**Item Type**	Journal Article
**Author**	Mina Ansari
**Author**	Sahand Adib Moradi
**Author**	Farzaneh Ghazi Sherbaf
**Author**	Abozar Hedayatnia
**Author**	Mohammad Hadi Aarabi
**Abstract**	ABSTRACTObjective:Research on psychobiological markers of Parkinson's disease (PD) remains a hot topic. Non-motor symptoms such as depression and REM sleep behavior disorder (RBD) each attribute to a particular neurodegenerative cluster in PD, and might enlighten the way for early prediction/detection of PD. The neuropathology of mood disturbances remains unclear. In fact, a few studies have investigated depression using diffusion magnetic resonance imaging (diffusion MRI). METHOD: Diffusion MRI of PD patients without comorbid RBD was used to assess whether microstructural abnormalities are detectable in the brain of 40 PD patients with depression compared to 19 patients without depression. Diffusion MRI connectometry was used to carry out group analysis between age- and gender-matched PD patients with and without depressive symptoms. Diffusion MRI connectometry is based on spin distribution function, which quantifies the density of diffusing water and is a sensitive and specific analytical method to psychological differences between groups. RESULTS: A significant difference (FDR = 0.016129) was observed in the left and right uncinate fasciculi, left and right inferior longitudinal fasciculi, left and right fornices, left inferior fronto-occipital fasciculus, right corticospinal tract, genu of corpus callosum, and middle cerebellar peduncle. CONCLUSION: These results suggest the prominent circuits involved in emotion recognition, particularly negative emotions, might be impaired in comorbid depressive symptoms in PD.
**Date**	2019 Jan
**Language**	eng
**Extra**	Place: United States
**Volume**	31
**Pages**	5-12
**Publication**	International psychogeriatrics
**DOI**	10.1017/S1041610218000170
**Issue**	1
**Journal Abbr**	Int Psychogeriatr
**ISSN**	1741-203X 1041-6102
**PMID**	29560834
**Date Added**	6.7.2025, 19:09:42
**Modified**	5.9.2025, 14:26:22

Notes:

Not Included: not on SC

Tags: EXCLUDED

Comparison of visual and auditory emotion recognition in patients with cerebellar and Parkinson´s disease.

**Item Type**	Journal Article
**Author**	Michael Adamaszek
**Author**	Federico D'Agata
**Author**	Christopher J. Steele
**Author**	Bernhard Sehm
**Author**	Cornelia Schoppe
**Author**	Karl Strecker
**Author**	Hartwig Woldag
**Author**	Horst Hummelsheim
**Author**	Kenneth C. Kirkby
**Abstract**	Widespread cortical-subcortical networks are involved in the recognition and discrimination of emotional contents of facial and vocal expression, whereby the cerebellum and basal ganglia are two subcortical regions implicated in these networks with limited evidence to their specific contributions. To investigate this we compared patients with circumscribed cerebellar lesions and patients with Parkinson's disease (PD) on an approved test battery. We studied two groups with subcortical disease, focal cerebellar infarction (n = 22) and PD (n = 22), and a neurological control group with focal supratentorial ischemia (SI) (n = 16) were. Assessments were according to inpatient protocols for neuropsychological routine evaluation, including tests of memory, executive function and attention. Participants completed the Tuebingen Affect Battery, a recognized measure of recognition and discrimination of facial and vocal expression of emotion. As a result, cerebellar lesions were associated with greater impairment than PD and SI in recognition and discrimination of cues of both facial and vocal expressions of differing basic emotions. No confounding effect of other cognitive domains, particularly executive function and attention, was found. Taken together, our findings suggest a specific contribution of the cerebellum to cerebral networks that process facial and vocal emotion expression, related to rapid decisions regulating appropriate behavioral responses in social environments.
**Date**	2019 Apr
**Language**	eng
**Extra**	Place: England
**Volume**	14
**Pages**	195-207
**Publication**	Social neuroscience
**DOI**	10.1080/17470919.2018.1434089
**Issue**	2
**Journal Abbr**	Soc Neurosci
**ISSN**	1747-0927 1747-0919
**PMID**	29375013
**Date Added**	6.7.2025, 19:09:36
**Modified**	5.9.2025, 14:24:39

Notes:

Included

sample characteristics

size: 22 cerebellar infarction, 22 PD, 16 supratentorial infarction within the supply regions of the middle or posterior cerebral artery (All did not differ in age, sex, education or handedness)

Parkinson's Disease type and duration: **NA**

medication: ON state

Hoehn-Yahr: M = 2.14 ± 0.83

UPDRS-3: M = 12.0, SD = 5.7

gender: 13 male (59%)

averaged ages (SD, range): M=57, SD=7

other neurological disease (tumor, stroke, etc.): none

other major psychopathology: None

origin country (or ethnicity): Germany

method observational

instruments used in order to quantify the variables

Social cognition aspect: emotion recognition

Name of the task: Tübingen affect battery (TAB)

type of stimulus [face/voice etc., Ekman faces/other etc.]: face (female faces) and voice (sentences spoken by one female speaker)

task condition: five emotion conditions, namely happiness, sadness, anger, fear, and neutral.

operationalization: accuracy

relevant subtests:

1. TAB2, Facial affect discrimination
2. TAB3, Facial affect naming
3. TAB4, Facial affect selection
4. TAB5, Facial affect matching
5. TAB7, Emotional prosody discrimination
6. TAB8a, Name the emotional prosody
7. TAB8b, Conflicting emotional prosody
8. TAB9, Match emotional prosody to an emotional face
9. TAB10, Match emotional face to the emotional prosody

Subtest order	Subtest issue	
**1**	Facial identity discrimination	Participant has to d
**2**	Facial affect discrimination	Participant has to de
**3**	Facial affect naming	Participant has to n
**4**	Facial affect selection	Participant has to p
**5**	Facial affect matching	Participant has to m card (15 trials)
**6**	Nonemotional prosody discrimination	Participant listens t and has to indica
**7**	Emotional prosody discrimination	Participant listens to has to judge if th
**8a**	Name the emotional prosody	Participant listens to the emotional pr
**8b**	Conflicting emotional prosody	Participant listens to parallel (congruer example (32 trials
**9**	Match emotional prosody to an emotional face	Participant is reques facial emotion of
**10**	Match emotional face to the emotional prosody	Participant is asked sentence (15 trial

Main findings related to the review's scope

CI was significantly lower, attributable to the emotional subtests, but not the perception subtests. By contrast PD and SI had comparable scores.

For the type of emotions the analysis showed a significantly greater number of errors for CI (more than 3 additional errors, CI>SI,PD) and an interaction between group and type of emotion. The post-hoc analyses regarding PD resulted in more errors in fear and happiness, compared to sadness/anger.

Tags: emotion recognition, behavioral

Compensatory premotor activity during affective face processing in subclinical carriers of a single mutant Parkin allele

Item Type Journal Article
Author Silke Anders
Author Benjamin Sack

Author Anna Pohl
Author Thomas Muente
Author Peter Pramstaller
Author Christine Klein
Author Ferdinand Binkofski

Abstract Patients with Parkinson's disease suffer from significant motor impairments and accompanying cognitive and affective dysfunction due to progressive disturbances of basal ganglia-cortical gating loops. Parkinson's disease has a long presymptomatic stage, which indicates a substantial capacity of the human brain to compensate for dopaminergic nerve degeneration before clinical manifestation of the disease. Neuroimaging studies provide evidence that increased motor-related cortical activity can compensate for progressive dopaminergic nerve degeneration in carriers of a single mutant Parkin or PINK1 gene, who show a mild but significant reduction of dopamine metabolism in the basal ganglia in the complete absence of clinical motor signs. However, it is currently unknown whether similar compensatory mechanisms are effective in non-motor basal ganglia-cortical gating loops. Here, we ask whether asymptomatic Parkin mutation carriers show altered patterns of brain activity during processing of facial gestures, and whether this might compensate for latent facial emotion recognition deficits. Current theories in social neuroscience assume that execution and perception of facial gestures are linked by a special class of visuomotor neurons ('mirror neurons') in the ventrolateral premotor cortex/pars opercularis of the inferior frontal gyrus (Brodmann area 44/6). We hypothesized that asymptomatic Parkin mutation carriers would show increased activity in this area during processing of affective facial gestures, replicating the compensatory motor effects that have previously been observed in these individuals. Additionally, Parkin mutation carriers might show altered activity in other basal ganglia-cortical gating loops. Eight asymptomatic heterozygous Parkin mutation carriers and eight matched controls underwent functional magnetic resonance imaging and a subsequent facial emotion recognition task. As predicted, Parkin mutation carriers showed significantly stronger activity in the right ventrolateral premotor cortex during execution and perception of affective facial gestures than healthy controls. Furthermore, Parkin mutation carriers showed a slightly reduced ability to recognize facial emotions that was least severe in individuals who showed the strongest increase of ventrolateral premotor activity. In addition, Parkin mutation carriers showed a significantly weaker than normal increase of activity in the left lateral orbitofrontal cortex (inferior frontal gyrus pars orbitalis, Brodmann area 47), which was unrelated to facial emotion recognition ability. These findings are consistent with the hypothesis that compensatory activity in the ventrolateral premotor cortex during processing of affective facial gestures can reduce impairments in facial emotion recognition in subclinical Parkin mutation carriers. A breakdown of this compensatory mechanism might lead to the impairment of facial expressivity and facial emotion recognition observed in manifest Parkinson's disease.

Date 2012-04
Language English
Extra Place: GREAT CLARENDON ST, OXFORD OX2 6DP, ENGLAND Type: Article
Volume 135
Publisher OXFORD UNIV PRESS
Pages 1128-1140
Publication BRAIN
DOI 10.1093/brain/aws040
Issue 4

ISSN 0006-8950
Date Added 14.7.2025, 14:50:39
Modified 5.9.2025, 14:26:08

Notes:

Not Included: not on PD
Tags: EXCLUDED

Comprehensive review of literature on Parkinson's disease diagnosis

Item Type Journal Article
Author P. Pradeep
Author Kamalakannan J
Abstract PD is one of the neurodegenerative illnesses affects 1–2 individuals per 1000 people over the age of 60 and has a 1 % prevalence rate. It affects both the non-motor and motor aspects of movement, including initiation, execution, and planning. Prior to behavioral and cognitive abnormalities like dementia, movement-related symptoms including stiffness, tremor, and initiation issues may be observed. Patients with PD have substantial reductions in social interactions, quality of life (QoL), and familial ties, as well as significant financial burdens on both the individual and societal levels. The healthcare industry is mostly using ML approaches with the modalities like image, signal, and data as well. Therefore, this survey aims to conduct a review of 50 articles on Parkinson disease diagnosis using different modalities. The survey includes (i) Classifying multimodal articles on Parkinson disease diagnosis (image, signal, data) using various machine learning, deep learning, and other approaches. (ii) Analyzing different datasets, simulation tools used in the existing papers. (iii)Examining certain performance measures, assessing the best performance, and chronological review of reviewed paper. Finally, the review determines the research gaps and obstacles in this research topic.
Date 2024
URL https://www.sciencedirect.com/science/article/pii/S1476927124002160
Volume 113
Pages 108228
Publication Computational Biology and Chemistry
DOI https://doi.org/10.1016/j.compbiolchem.2024.108228
ISSN 1476-9271
Date Added 6.7.2025, 19:12:34
Modified 5.9.2025, 14:52:13

Notes:

Not Included: not a systematic review
Tags: EXCLUDED

COPPADIS-2015 (COhort of Patients with PArkinson's DIsease in Spain, 2015), a global -clinical evaluations, serum biomarkers, genetic studies and neuroimaging- prospective, multicenter, non-interventional, long-term study on Parkinson's disease progression

**Item Type**	Journal Article
**Author**	Diego Santos-Garcia
**Author**	Pablo Mir
**Author**	Esther Cubo
**Author**	Lydia Vela
**Author**	Mari Cruz Rodriguez-Oroz
**Author**	Maria Jose Marti
**Author**	Jose Matias Arbelo
**Author**	Jon Infante
**Author**	Jaime Kulisevsky
**Author**	Pablo Martinez-Martin
**Author**	COPPADIS Study Grp
**Abstract**	Background: Parkinson's disease (PD) is a progressive neurodegenerative disorder causing motor and non-motor symptoms that can affect independence, social adjustment and the quality of life (QoL) of both patients and caregivers. Studies designed to find diagnostic and/or progression biomarkers of PD are needed. We describe here the study protocol of COPPADIS-2015 (COhort of Patients with PArkinson's DIsease in Spain, 2015), an integral PD project based on four aspects/concepts: 1) PD as a global disease (motor and non-motor symptoms); 2) QoL and caregiver issues; 3) Biomarkers; 4) Disease progression. Methods/design: Observational, descriptive, non-interventional, 5-year follow-up, national (Spain), multicenter (45 centers from 15 autonomous communities), evaluation study. Specific goals: (1) detailed study (clinical evaluations, serum biomarkers, genetic studies and neuroimaging) of a population of PD patients from different areas of Spain, (2) comparison with a control group and (3) follow-up for 5 years. COPPADIS-2015 has been specifically designed to assess 17 proposed objectives. Study population: approximately 800 non-dementia PD patients, 600 principal caregivers and 400 control subjects. Study evaluations: (1) baseline includes motor assessment (e.g., Unified Parkinson's Disease Rating Scale part III), non-motor symptoms (e.g., Non-Motor Symptoms Scale), cognition (e.g., Parkinson's Disease Cognitive Rating Scale), mood and neuropsychiatric symptoms (e.g., Neuropsychiatric Inventory), disability, QoL (e.g., 39-item Parkinson's disease Quality of Life Questionnaire Summary-Index) and caregiver status (e.g., Zarit Caregiver Burden Inventory); (2) follow-up includes annual (patients) or biannual (caregivers and controls) evaluations. Serum biomarkers (S-100b protein, TNF-alpha, IL-1, IL-2, IL-6, vitamin B12, methylmalonic acid, homocysteine, uric acid, C-reactive protein, ferritin, iron) and brain MRI (volumetry, tractography and MTAi [Medial Temporal Atrophy Index]), at baseline and at the end of follow-up, and genetic studies (DNA and RNA) at baseline will be performed in a subgroup of subjects (300 PD patients and 100 control subjects). Study periods: (1) recruitment period, from November, 2015 to February, 2017 (basal assessment); (2) follow-up period, 5 years; (3) closing date of clinical follow-up, May, 2022. Funding: Public/Private. Discussion: COPPADIS-2015 is a challenging initiative. This project will provide important information on the natural history of PD and the value of various biomarkers.
**Date**	2016-02-25

Language English
Extra Place: CAMPUS, 4 CRINAN ST, LONDON N1 9XW, ENGLAND Type: Article
Volume 16
Publisher BMC
Publication BMC NEUROLOGY
DOI 10.1186/s12883-016-0548-9
Date Added 14.7.2025, 14:50:36
Modified 5.9.2025, 14:55:31

Notes:

Not Included: Does not study SC
Tags: EXCLUDED

Core mechanisms in ‘theory of mind’

Item Type Journal Article
Author Alan M. Leslie
Author Ori Friedman
Author Tim P. German
Date 12/2004
Language en
Library Catalog DOI.org (Crossref)
URL https://linkinghub.elsevier.com/retrieve/pii/S1364661304002608
Accessed 19.1.2026, 7:33:03
Volume 8
Pages 528-533
Publication Trends in Cognitive Sciences
DOI 10.1016/j.tics.2004.10.001
Issue 12
Journal Abbr Trends in Cognitive Sciences
ISSN 13646613
Date Added 19.1.2026, 7:33:03
Modified 19.1.2026, 7:33:03

Cortical thinning correlates of changes in visuospatial and visuoperceptual performance in Parkinson's disease: A 4-year follow-up

Item Type Journal Article
Author A.I. Garcia-Diaz
Author B. Segura
Author H.C. Baggio

**Author**	C. Uribe
**Author**	A. Campabadal
**Author**	A. Abos
**Author**	M.J. Marti
**Author**	F. Valldeoriola
**Author**	Y. Compta
**Author**	N. Bargallo
**Author**	C. Junque
**Date**	01/2018
**Language**	en
**Short Title**	Cortical thinning correlates of changes in visuospatial and visuoperceptual performance in Parkinson's disease
**Library Catalog**	Crossref
**URL**	https://linkinghub.elsevier.com/retrieve/pii/S1353802017304261
**Accessed**	13.7.2025, 20:22:27
**License**	https://www.elsevier.com/tdm/userlicense/1.0/
**Volume**	46
**Publisher**	Elsevier BV
**Pages**	62-68
**Publication**	Parkinsonism & Related Disorders
**DOI**	10.1016/j.parkreldis.2017.11.003
**ISSN**	1353-8020
**Date Added**	13.7.2025, 20:22:27
**Modified**	13.7.2025, 20:22:27

Notes:

Not Included: not on SC

Tags: EXCLUDED

Crossed functional specialization between the basal ganglia and cerebellum during vocal emotion decoding: Insights from stroke and Parkinson's disease

**Item Type**	Journal Article
**Author**	Marine Thomasson
**Author**	Damien Benis
**Author**	Philippe Voruz
**Author**	Arnaud Saj
**Author**	Marc Verin
**Author**	Frederic Assal
**Author**	Didier Grandjean
**Author**	Julie Peron

Abstract There is growing evidence that both the basal ganglia and the cerebellum play functional roles in emotion processing, either directly or indirectly, through their connections with cortical and subcortical structures. However, the lateralization of this complex processing in emotion recognition remains unclear. To address this issue, we investigated emotional prosody recognition in individuals with Parkinson's disease (model of basal ganglia dysfunction) or cerebellar stroke patients, as well as in matched healthy controls (n = 24 in each group). We analysed performances according to the lateralization of the predominant brain degeneration/lesion. Results showed that a right (basal ganglia and cerebellar) hemispheric dysfunction was likely to induce greater deficits than a left one. Moreover, deficits following left hemispheric dysfunction were only observed in cerebellar stroke patients, and these deficits resembled those observed after degeneration of the right basal ganglia. Additional analyses taking disease duration / time since stroke into consideration revealed a worsening of performances in patients with predominantly right-sided lesions over time. These results point to the differential, but complementary, involvement of the cerebellum and basal ganglia in emotional prosody decoding, with a probable hemispheric specialization according to the level of cognitive integration.

Date 2022-10

Language English

Extra Place: ONE NEW YORK PLAZA, SUITE 4600, NEW YORK, NY, UNITED STATES Type: Article

Volume 22

Publisher SPRINGER

Pages 1030-1043

Publication COGNITIVE AFFECTIVE & BEHAVIORAL NEUROSCIENCE

DOI 10.3758/s13415-022-01000-4

Issue 5

ISSN 1530-7026

Date Added 14.7.2025, 14:50:29

Modified 5.9.2025, 14:58:26

Notes:

Included

Sample characteristics

Size: 24 PD (12 RPD, 12 LPD), 24 patients with focal cerebellar lesions due to ischeamic stroke (12 LCBL, 12 RCBL), 24 HC: all groups matched for age, handedness, sex

PD-type: NA

PD-duration: LPD: *M* = 11.58, *SD* = 4.62; RPD: *M* = 11.47, *SD* = 3.94.

Medication: ON state

Hoehn-Yahr (ON): NA (seem to be incorrect date)

UPDRS-3 (ON): LPD: M = 8.37, SD = 6.66; RPD: M = 6.96, SD = 4.72

Gender (male): NA

Age: LPD: M = 58.75, SD = 7.56; RPD: M = 54.58, SD = 6.87

Other neurological disease (tumor, stroke, etc.): None

Other major psychopathology: None

Origin country (or ethnicity): France

method behavioral

instruments used in order to quantify the variables

Social cognition aspect: emotion recognition

Name of the task: NA

Type of stimulus [face/voice etc., Ekman faces/other etc.]: 60 pseudowords

Task condition:five different emotional prosodies (anger, fear, happiness, neutral, and sadness)

Operationalization: For each pseudoword, they have to indicate the extent to which it expresses different emotions, by moving a cursor along a continuous analog scale (emotion scales display: happiness, anger, fear, and sadness, neutral, and surprise) ranging from “No emotion expressed” to “Emotion expressed with exceptional intensity. >> not clear what was the measured variable.

Main findings related to the review's scope

Concerning patients with PD, only left-lateralized patients exhibited a vocal emotion deficit

RPD performed better than all the other patient subgroups (LPD, RCBL, and LCBL)

LPD made more misattributions than LCBL

LPD (i.e., with greater right hemispheric brain dysfunction) and RCBL were the subgroups with the most pronounced deficit for emotion vocal recognition.

Tags: emotion recognition, behavioral

Decision making in Parkinson's disease: Analysis of behavioral and physiological patterns in the Iowa gambling task.

**Item Type**	Journal Article
**Author**	Mutsutaka Kobayakawa
**Author**	Shinichi Koyama
**Author**	Masaru Mimura
**Author**	Mitsuru Kawamura
**Abstract**	Recent studies suggest that social recognition processes are affected by Parkinson's disease (PD). However, whether PD patients exhibit behavioral changes is still controversial. The purpose of the present study was to examine the decision making of PD patients performing the Iowa Gambling Task (IGT). We recruited a large number of early, nondemented PD patients for the IGT. We also recorded the skin conductance responses (SCRs) during the task as a measure of emotional arousal. Compared with the normal control (NC) subjects, PD patients selected more disadvantageous decks in the IGT, and their SCRs were lower than those of NC subjects before making decisions and after receiving reward or punishment. The tendency toward risky choices was not correlated with age, education, global cognitive function, or the severity of the disease. These results confirmed that the decision making of PD patients was affected by the disease, rather than by other cognitive functions; moreover, such behavior was related to lower emotional responses. Behavioral and SCR patterns of PD patients were similar to those of amygdala-damaged patients. The response bias toward risky choices in PD may be explained by the dysfunction of the amygdala, which is known to be involved in risk evaluation.
**Date**	2008 Mar 15
**Language**	eng
**License**	(c) 2007 Movement Disorder Society.
**Extra**	Place: United States
**Volume**	23
**Pages**	547-552
**Publication**	Movement disorders : official journal of the Movement Disorder Society
**DOI**	10.1002/mds.21865
**Issue**	4
**Journal Abbr**	Mov Disord
**ISSN**	1531-8257 0885-3185
**PMID**	18069681
**Date Added**	6.7.2025, 19:09:39
**Modified**	5.9.2025, 14:42:01

Notes:

<u>**Not Included:**</u> Not assessing SC

Tags: EXCLUDED

Decision-making performance in Parkinson's disease correlates with lateral orbitofrontal volume

Item Type Journal Article
Author Mutsutaka Kobayakawa
Author Natsuko Tsuruya
Author Mitsuru Kawamura
Abstract Background Patients with Parkinson's disease (PD) exhibit poor decision-making, and the underlying neural correlates are unclear. We used voxel-based morphometry with Diffeomorphic Anatomical Registration through Exponentiated Lie algebra to examine this issue. Methods The decision-making abilities of 20 patients with PD and 37 healthy controls (HCs) were measured with a computerized Iowa Gambling Task (IGT). We assessed the local gray matter volumes of the patients and HCs and their correlations with decision-making performance, disease duration, disease severity, and anti-Parkinsonism medication dose. Results Compared with the HCs, the patients with PD exhibited poor IGT performances. The gray matter volumes in the medial orbitofrontal cortex, left inferior temporal cortex, and right middle frontal gyrus were decreased in the patients. Results in the regression analysis showed that lateral orbitofrontal volume correlated with performance in the IGT in PD. Regions that correlated with disease duration, severity, and medication dose did not overlap with orbitofrontal regions. Conclusion Our results indicate that the lateral and medial orbitofrontal cortex are related to decision-making in PD patients. Since the medial orbitofrontal cortex is shown to be involved in monitoring reward, reward monitoring seems to be impaired as a whole in PD patients. Meanwhile, the lateral region is related to evaluation of punishment, which is considered to have an influence on individual differences in decision-making performance in PD patients.
Date 2017
URL https://www.sciencedirect.com/science/article/pii/S0022510X16307493
Volume 372
Pages 232-238
Publication Journal of the Neurological Sciences
DOI https://doi.org/10.1016/j.jns.2016.11.046
ISSN 0022-510X
Date Added 6.7.2025, 19:12:34
Modified 5.9.2025, 14:42:05

Notes:

Not Included: Not assessing SC
Tags: EXCLUDED

Decoding emotional prosody in Parkinson's disease and its potential neuropsychological basis

Item Type Journal Article
Author Rachel L. C. Mitchell
Author Sofia Barbosa Boucas

Abstract Parkinson's disease patients may have difficulty decoding prosodic emotion cues. These data suggest that the basal ganglia are involved, but may reflect dorsolateral prefrontal cortex dysfunction. An auditory emotional n-back task and cognitive n-back task were administered to 33 patients and 33 older adult controls, as were an auditory emotional Stroop task and cognitive Stroop task. No deficit was observed on the emotion decoding tasks; this did not alter with increased frontal lobe load. However, on the cognitive tasks, patients performed worse than older adult controls, suggesting that cognitive deficits may be more prominent. The impact of frontal lobe dysfunction on prosodic emotion cue decoding may only become apparent once frontal lobe pathology rises above a threshold.

Date 2009-07
Language English
Extra Place: 530 WALNUT STREET, STE 850, PHILADELPHIA, PA 19106 USA Type: Article
Volume 31
Publisher TAYLOR & FRANCIS INC
Pages 553-564
Publication JOURNAL OF CLINICAL AND EXPERIMENTAL NEUROPSYCHOLOGY
DOI 10.1080/13803390802360534
Issue 5
ISSN 1380-3395
Date Added 14.7.2025, 14:50:42
Modified 5.9.2025, 14:47:09

Notes:

Included

Sample characteristics

Size: 33 PD, 33 HC

PD-type: Idiopathic PD

PD-duration: M = 8, SD = 4.6

Medication: ON state

Hoehn-Yahr:Range = 1-3

UPDRS-3: NA

Gender (male): 21 (64%)

Age: M = 63.6, SD = 9.9

Other neurological disease (tumor, stroke, etc.): None

Other major psychopathology: None

Origin country (or ethnicity): England

method behavioural

instruments used in order to quantify the variables

Social cognition aspect: Emotion recognition (prosody)/Interference

Name of the task: Auditory emotional Stroop task

Type of stimulus [face/voice etc., Ekman faces/other etc.]: prerecorded sentences in which the emotion conveyed by content matched or conflicted with that conveyed by prosody. The sentences were approximately the same length and were of consistent style and format (in the third person, involving a subject and an action). Participants were instructed to indicate the emotion conveyed by intonation and were told that that they could respond as soon as they had made a decision

Task condition: 60 happy, 60 sad sentences

Operationalization: Correct answers

Social cognition aspect: Emotion recognition (prosody)/Working memory

Name of the task: auditory emotional n-back

Type of stimulus [face/voice etc., Ekman faces/other etc.]: six bisyllabic concrete nouns of neutral content (e.g., carpet), spoken by a female and male speaker.

Task condition: happy, disgusted, angry, sad, and fearful tones

In each condition of the emotional n-back task, 20 stimuli were pseudorandomly selected from the suite of 60 word stimuli and were presented (via E-prime and BOSE headphones) 1 every 4 s. Participants identified the emotion conveyed by intonation, pressing 1 if they thought a word was spoken in a happy tone, 2 for a sad tone (as for the other emotion task), 3 for a disgusted tone, 4 for an angry tone, and 5

for a fearful tone. 0-back to 2-back conditions existed

Operationalization: Correct answers

Main findings related to the review's scope

For the auditory emotional Stroop task, there was no performance difference, $t(65) = 0.76$, $p = .450$.

the between-group difference in performance on the auditory n-back task was not significant, $t(65) = 0.24$, $p = .815$.

Tags: Emotion recognition, behavioral

Deep Brain Stimulation of the subthalamic nucleus does not negatively affect social cognitive abilities of patients with Parkinson's disease.

Item Type Journal Article
Author Ivan Enrici
Author Antonia Mitkova
Author Lorys Castelli
Author Michele Lanotte
Author Leonardo Lopiano
Author Mauro Adenzato

Abstract Bilateral deep brain stimulation (DBS) of the subthalamic nucleus (STN) is a treatment option for patients with advanced idiopathic PD successful at alleviating disabling motor symptoms. Nevertheless, the effects of STN-DBS on cognitive functions remain controversial and few studies have investigated modification of social cognitive abilities in patients with PD treated with STN-DBS. Here we expanded the typically-investigated spectrum of these abilities by simultaneously examining emotion recognition, and both affective and cognitive Theory of Mind (ToM). By means of a cross-sectional study, 20 patients with PD under dopaminergic replacement therapy, 18 patients with PD treated with STN-DBS, and 20 healthy controls performed the Ekman 60-Faces test, the full version of the Reading the Mind in the Eyes test, and the Protocol for the Attribution of Communicative Intentions. There were no differences between the PD groups (treated and not treated with STN-DBS) on any of the social cognitive tests. Our results suggest that patients with PD who are treated with STN-DBS do not experience detrimental effects on their social cognitive abilities. The present study, the first one examining a wide spectrum of social cognitive abilities after DBS of the STN, suggests that this surgical procedure can be considered safe from this standpoint.

Date 2017 Aug 25
Language eng
Extra Place: England
Volume 7
Pages 9413
Publication Scientific reports
DOI 10.1038/s41598-017-09737-6
Issue 1
Journal Abbr Sci Rep

ISSN 2045-2322
PMID 28842656
PMCID PMC5573348
Date Added 6.7.2025, 19:09:42
Modified 5.9.2025, 14:35:02

Notes:

Included

sample characteristics

size: 20 PD under dopaminergic replacement therapy (DRT), 18 PD with STN-DBS, and 20 HC

Parkinson's Disease type and duration: idiopathic PD Mduration = NA

Medication: on medication

Hoehn-Yahr: NA

UPDRS-3: NA

Gender (male): DRT-PD 10 males (50%), DBS-PD 9 males (50%)

averaged ages (SD, range): DRT-PD M=59.75 SD=5.81 (50–70), DBS-PD M=60.89 SD=6.26 (41–70), HC M=60 SD=7.47 (45–71)

other neurological disease (tumor, stroke, etc.): None

other major psychopathology: None

origin country (or ethnicity): Italy

method (Review, meta-analysis or observational and/or self-reported):

instruments used in order to quantify the variables

Social cognition aspect: emotion recognition

Name of the task: the Ekman 60-Faces Test

type of stimulus [face/voice etc., Ekman faces/other etc.]: Ekman faces

task condition: happiness, surprise, anger, disgust, fear, and sadness

operationalization: accuracy

Social cognition aspect: cognitive ToM

Name of the task: the Protocol for the Attribution of Communicative Intentions (PACI)

type of stimulus [face/voice etc., Ekman faces/other etc.]: A series of 18 comic strips was presented to participants, representing two different conceptual categories: communicative intentions (CInt), and physical causality (PhC). The CInt condition consisted of nine stories that each portrayed two characters involved in a communicative interaction (e.g. a person asking for a glass of water and obtaining it from another person). **The PhC condition** consisted of nine stories that each depicted a physical interaction between objects (e.g. a ball blown by a gust of wind knocks over and breaks bottles of water), and **did not require ToM abilities**. Each story consisted of three pictures (development phase) that appeared consecutively on the screen, followed by a set of four options (response phase), and participants selected the most appropriate and logical story ending. The correct picture represented a probable and congruent outcome resulting from the development phase, while the incorrect pictures represented an improbable or incongruent effect.

operationalization: accuracy

Social cognition aspect: affective ToM

Name of the task: the Reading the Mind in the Eyes (RME)

type of stimulus [face/voice etc., Ekman faces/other etc.]: partial pictures of a face depicting only the eye region.

operationalization: accuracy

Main findings related to the review's scope

No sig dif on Ekman 60-Faces total score

Only on Ekman-surprise **DRT-PD performed worse than HC**

Both PD groups perfomed worse than HC on the Communicative intention task as well as on the RMET.
Tags: Emotion recognition, ToM, behavioral

Deep Brain Stimulation of the Subthalamic Nucleus Influences Facial Emotion Recognition in Patients With Parkinson's Disease: A Review.

**Item Type**	Journal Article
**Author**	Caroline Wagenbreth
**Author**	Maria Kuehne
**Author**	Hans-Jochen Heinze
**Author**	Tino Zaehle
**Abstract**	Parkinson's disease (PD) is a neurodegenerative disorder characterized by motor symptoms following dopaminergic depletion in the substantia nigra. Besides motor impairments, however, several non-motor detriments can have the potential to considerably impact subjectively perceived quality of life in patients. Particularly emotion recognition of facial expressions has been shown to be affected in PD, and especially the perception of negative emotions like fear, anger, or disgust is impaired. While emotion processing generally refers to automatic implicit as well as conscious explicit processing, the focus of most previous studies in PD was on explicit recognition of emotions only, while largely ignoring implicit processing deficits. Deep brain stimulation of the subthalamic nucleus (STN-DBS) is widely accepted as a therapeutic measure in the treatment of PD and has been shown to advantageously influence motor problems. Among various concomitant non-motor effects of STN-DBS, modulation of facial emotion recognition under subthalamic stimulation has been investigated in previous studies with rather heterogeneous results. Although there seems to be a consensus regarding the processing of disgust, which significantly deteriorates under STN stimulation, findings concerning emotions like fear or happiness report heterogeneous data and seem to depend on various experimental settings and measurements. In the present review, we summarized previous investigations focusing on STN-DBS influence on recognition of facial emotional expressions in patients suffering from PD. In a first step, we provide a synopsis of

disturbances and problems in facial emotion processing observed in patients with PD. Second, we present findings of STN-DBS influence on facial emotion recognition and especially highlight different impacts of stimulation on implicit and explicit emotional processing.

Date 2019
Language eng
License Copyright © 2019 Wagenbreth, Kuehne, Heinze and Zaehle.
Extra Place: Switzerland
Volume 10
Pages 2638
Publication Frontiers in psychology
DOI 10.3389/fpsyg.2019.02638
Journal Abbr Front Psychol
ISSN 1664-1078
PMID 31849760
PMCID PMC6901782
Date Added 6.7.2025, 19:09:36
Modified 5.9.2025, 15:00:14

Notes:

Not Included: not a systematic review

Tags: EXCLUDED

Deep Brain Stimulation of the Subthalamic Nucleus Selectively Modulates Emotion Recognition of Facial Stimuli in Parkinson's Patients.

Item Type Journal Article
Author Caroline Wagenbreth
Author Maria Kuehne
Author Jürgen Voges
Author Hans-Jochen Heinze
Author Imke Galazky
Author Tino Zaehle
Abstract : Background: Diminished emotion recognition is a known symptom in Parkinson (PD) patients and subthalamic nucleus deep brain stimulation (STN-DBS) has been shown to further deteriorate the processing of especially negative emotions. While emotion recognition generally refers to both, implicit and explicit processing, demonstrations of DBS-influences on implicit processing are sparse. In the present study, we assessed the impact of STN-DBS on explicit and implicit processing for emotional stimuli. METHODS: Under STN-DBS ON and OFF, fourteen PD patients performed an implicit as well as an explicit emotional processing task. To assess implicit emotional processing, patients were tested with a lexical decision task (LTD) combined with an affective priming paradigm, which provides emotional content through the facial eye region. To assess explicit emotional processing, patients additionally explicitly rated the emotional status of eyes and words used in the

implicit task. RESULTS: DBS affected explicit emotional processing more than implicit processing with a more pronounced effect on error rates than on reaction speed. STN-DBS generally worsened implicit and explicit processing for disgust stimulus material but improved explicit processing of fear stimuli. CONCLUSIONS: This is the first study demonstrating influences of STN-DBS on explicit and implicit emotion processing in PD patients. While STN stimulation impeded the processing of disgust stimuli, it improved explicit discrimination of fear stimuli.

Date 2019 Aug 28
Language eng
Extra Place: Switzerland
Volume 8
Publication Journal of clinical medicine
DOI 10.3390/jcm8091335
Issue 9
Journal Abbr J Clin Med
ISSN 2077-0383
PMID 31466414
PMCID PMC6781243
Date Added 6.7.2025, 19:09:37
Modified 5.9.2025, 15:00:18

Notes:

Not Included: Pre-Post DBS in PD-group. No control group

Tags: EXCLUDED

Default Mode Network Functional Connectivity As a Transdiagnostic Biomarker of Cognitive Function.

Item Type Journal Article
Author Vaibhav Tripathi
Author Ishaan Batta
Author Andre Zamani
Author Daniel A. Atad
Author Sneha K. S. Sheth
Author Jiahe Zhang
Author Tor D. Wager
Author Susan Whitfield-Gabrieli
Author Lucina Q. Uddin
Author Ruchika S. Prakash
Author Clemens C. C. Bauer
Abstract The default mode network (DMN) is intricately linked with processes such as self-referential thinking, episodic memory recall, goal-directed cognition, self-projection, and theory of mind. In recent years, there has been a surge in the number of studies examining its functional connectivity, particularly its relationship with frontoparietal

networks involved in top-down attention, executive function, and cognitive control. The fluidity in switching between these internal and external modes of processing, which is highlighted by anticorrelated functional connectivity, has been proposed as an indicator of cognitive health. Due to the ease of estimation of functional connectivity-based measures through resting-state functional magnetic resonance imaging paradigms, there is now a wealth of large-scale datasets, paving the way for standardized connectivity benchmarks. In this review, we explore the promising role of DMN connectivity metrics as potential biomarkers of cognitive state across attention, internal mentation, mind wandering, and meditation states and investigate deviations in trait-level measures across aging and in clinical conditions such as Alzheimer's disease, Parkinson's disease, depression, attention-deficit/hyperactivity disorder, and others. We also tackle the issue of reliability of network estimation and functional connectivity and share recommendations for using functional connectivity measures as a biomarker of cognitive health.

Date 2025 Apr
Language eng
License Copyright © 2025 Society of Biological Psychiatry. Published by Elsevier Inc. All rights reserved.
Extra Place: United States
Volume 10
Pages 359-368
Publication Biological psychiatry. Cognitive neuroscience and neuroimaging
DOI 10.1016/j.bpsc.2024.12.016
Issue 4
Journal Abbr Biol Psychiatry Cogn Neurosci Neuroimaging
ISSN 2451-9030 2451-9022
PMID 39798799
PMCID PMC12207756
Date Added 6.7.2025, 19:09:42
Modified 5.9.2025, 14:58:46

Notes:

Not Included: not a systematic review

Tags: EXCLUDED

Deficits in decoding emotional facial expressions in Parkinson's disease

Item Type Journal Article
Author K Dujardin
Author S Blairy
Author L Defebvre
Author S Duhem
Author Y Noël
Author U Hess
Author A Destée

Abstract Introduction: The basal ganglia have numerous connections not only with the motor cortex but also with the prefrontal and limbic cortical areas. Therefore, basal ganglia lesions can disturb motor function but also cognitive function and emotion processing. The aim of the present study was to assess the consequences of Parkinson's disease (PD) on ability to decode emotional facial expressions (EFEs)-a method commonly used to investigate non-verbal emotion processing. Methods: Eighteen PD patients participated in the study, together with 18 healthy subjects strictly matched with respect to age, education and sex. The patients were early in the course of the disease and had not yet received any antiparkinsonian treatment. Decoding of EFEs was assessed using a standardized, quantitative task where the expressions were of moderate intensity, i.e. quite similar to those experienced in everyday life. A set of tests also assessed executive function. Visuospatial perception, depression and anxiety were measured. Results: Early in the course of the disease, untreated PD patients were significantly impaired in decoding EFEs, as well as in executive function. The deficits were significantly interrelated, although neither was significantly related to severity of the motor symptoms. Visuospatial perception was not impaired, and the patients' impairment was related neither to their depression nor to their anxiety score. The PD patients' impairment in decoding EFEs was related to a systematic response bias. Conclusion: Early in the course of PD, non-verbal emotional information processing is disturbed. This suggests that in PD, nigrostriatal dopaminergic depletion leads not only to motor and cognitive disturbances but also to emotional information processing deficits. The observed correlation pattern does not enable adoption of a clear-cut position in the debate over totally or partially segregated functional organization of the basal ganglia circuits. (C) 2003 Elsevier Ltd. All rights reserved.

Date 2004

Language English

Extra Place: THE BOULEVARD, LANGFORD LANE, KIDLINGTON, OXFORD OX5 1GB, ENGLAND Type: Article

Volume 42

Publisher PERGAMON-ELSEVIER SCIENCE LTD

Pages 239-250

Publication NEUROPSYCHOLOGIA

DOI 10.1016/S0028-3932(03)00154-4

Issue 2

ISSN 0028-3932

Date Added 14.7.2025, 14:50:43

Modified 5.9.2025, 14:34:30

Notes:

Included

sample characteristics

size: 18 PD and 18 HC (Matched in sex, age and education)

Parkinson's Disease type and duration: NA, Mduration = 0.94 SD=0.4 (0.5-2)

Medication: no medication

Hoehn-Yahr: NA

UPDRS-3: M= 17.58 (SD=6.16) [range: 8–28]

Gender (male): 12 males (67%)

averaged ages (SD, range): M= 60.17 (SD=10.31)

other neurological disease (tumor, stroke, etc.): no dementia

other major psychopathology: NA

origin country (or ethnicity): NA

method observational

instruments used in order to quantify the variables

Social cognition aspect: emotion recognition

Name of the task: NA

type of stimulus [face/voice etc., Ekman faces/other etc.]: emotional facial expressions constructed by Hess & Blairy (1995). series of standardized emotional facial expressions (EFEs) produced by two male and two female Caucasian actors (JACFEE) (Matsumoto & Ekman, 1988). Based on the neutral face (0% emotional intensity) and the full-blown EFE (100% emotional intensity) from the same actor and using the Morph 1.0 computer program, the authors constructed a series of intermediate expressions differing in emotional intensity by steps of 10%.

task condition: happiness, anger, sadness, disgust and fear

operationalization: The participants' task was to rate the emotion portrayed by each face and to quantify its intensity. In order to achieve this, they had to rate each expression on seven-point scales for each of seven basic emotions: happiness, sadness, fear, anger, disgust, surprise and shame.

After completion of the emotion scales, participants were also required to rate the task difficulty (i.e. how difficult they found it to deduce the emotion portrayed by that specific facial expression). All the scales were anchored by "not at all" at one extremity and "very intensely" at the other.

>> rating of intensity, difficulty (self-report), and accuracy.

Main findings related to the review's scope

accuracy >> lower decoding accuracy scores in PD patients when compared to healthy controls. Emotion × group and Emotion × group x Intensity not sig

intensity >> For disgust expressions: PD patients scored higher on the surprise scale than the healthy controls, and PD patients scored lower on the disgust scale than the controls.

For sadness expressions: PD patients scored higher on the surprise scale than the healthy controls, and PD patients scored lower on the sadness scale than the controls.

For anger expressions: PD patients scored higher on the surprise scale than the healthy controls, and PD patients scored lower on the anger scale than the controls.

In summary, for each EFE judged, the PD patients rated the target emotion lower than the healthy controls, while evaluating the expressed surprise more highly.

Difficulty >> healthy controls rated 70% intensity EFEs as easier to decode than 30% intensity EFEs ($t_{(17)}$=3.07, P=.006) while no differences emerged for the PD patients ($t_{(17)}$=0.32, n.s.). This suggests that in contrast to healthy controls, the PD patients were not aware of how easy it is to decode 70% intensity EFEs.

Tags: Emotion recognition, behavioral, Questionnaire

Deficits in Emotion Perception and Cognition in Patients with Parkinson's Disease: A Systematic Review

Item Type Journal Article
Author Mohit Gothwal
Author Shyam Sundar Arumugham
Author Ravi Yadav
Author Pramod K. Pal
Author Shantala Hegde
Abstract Non-motor symptoms (NMS) are common among Parkinson's disease (PD) patients and have a significant impact on quality of life. NMS such as deficits in emotion perception are gaining due focus in the recent times. As emotion perception and cognitive functions share certain common neural substrates, it becomes pertinent to evaluate existing emotion perception deficits in view of underlying cognitive deficits. The current systematic review aimed at examining studies on emotion perception PD in the last decade. We carried out a systematic review of 44 studies from the PubMed database. We reviewed studies examining emotion perception and associated cognitive deficits, especially executive function and visuospatial function in PD. This review also examines how early and advanced PD differ in emotion perception deficits and how the presence of common neuropsychiatric conditions such as anxiety, apathy, and depression as well as neurosurgical procedure such as deep brain stimulation affect emotion perception. The need for future research employing a comprehensive evaluation of neurocognitive functions and emotion perception is underscored as it has a significant bearing on planning holistic intervention strategies.
Date 2022-06
Language English
Extra Place: WOLTERS KLUWER INDIA PVT LTD , A-202, 2ND FLR, QUBE, C T S NO 1498A-2 VILLAGE MAROL, ANDHERI EAST, MUMBAI, Maharashtra, INDIA Type: Review

Volume 25
Publisher WOLTERS KLUWER MEDKNOW PUBLICATIONS
Pages 367-375
Publication ANNALS OF INDIAN ACADEMY OF NEUROLOGY
DOI 10.4103/aian.aian_573_21
Issue 3
ISSN 0972-2327
Date Added 14.7.2025, 14:50:29
Modified 5.9.2025, 14:36:58

Notes:

Included – Systematic review

search of literature on PubMed and other sources [refer to Table 1 and Figure 1]. The articles were restricted to English language and published between January 2010 and December 2019. The key terms were Parkinson's disease, emotion, facial expression, emotional prosody and music emotion perception. A total of 44 articles reporting emotion perception and cognitive assessment in PD were selected. Articles related to DBS were also reviewed as they highlight role of this neurosurgical process on emotion perception among PD patients.

Main findings related to the review's scope

This ability to successfully interpret emotions through facial expressions require a complex interplay among brain regions such as amygdala, hypothalamus, mesolimbic, dopaminergic signalling pathways, as well as cortical regions such as prefrontal cortex, and areas of temporal and parietal cortex. The pathophysiological changes in PD such as loss of grey matter in amygdala, orbitofrontal cortex, affected corticostriatal connections[2] contribute to impaired emotion perception and might add to difficulty in pragmatic communication,[21] refer to

Some of the reviewed studies provide evidence in support of specific emotion perception deficits such as decoding negative emotions. The accuracy to perform on emotion perception tasks is moderated by nature of emotion perception tasks

General (non-specific) recognition of prosody deficits has been reported in both mild to moderately PD and advanced PD others reported specific prosody deficits in fear, anger, disgust. In pwPD, deficits in recognizing emotions through prosody are related to executive function.

Tags: Emotion recognition, behavioral

Deficits in Emotion Recognition and Theory of Mind in Parkinson's Disease Patients With and Without Cognitive Impairments.

Item Type Journal Article
Author Alessandra Dodich
Author Giulia Funghi
Author Claudia Meli
Author Maria Pennacchio
Author Chiara Longo

Author Maria Chiara Malaguti
Author Raffaella Di Giacopo
Author Francesca Zappini
Author Luca Turella
Author Costanza Papagno

Abstract BACKGROUND: Emotion recognition and social deficits have been previously reported in Parkinson's disease (PD). However, the extent of these impairments is still unclear and social cognition is excluded from the cognitive domains considered in the current criteria for PD mild cognitive impairment (MCI). This study aims to analyze emotion recognition, affective and cognitive theory of mind in early PD patients classified according to Level II MCI criteria, and to evaluate the prevalence of socio-cognitive deficits in this sample. METHODS: We enrolled 45 participants with PD, classified as cognitively unimpaired (CU; n = 32) or MCI (n = 13) based on a standard neuropsychological assessment. Social cognitive skills were evaluated through validated tests for emotion recognition (i.e., Ekman 60-faces test, Ek60 Test) and mental states attribution (Story-based Empathy Task, SET) and compared to a group of 45 healthy controls (HC). Between-group differences in social tasks were performed, as well as correlation analyses to assess the relationship between social, cognitive, and clinical variables. Finally, the number of patients with social cognitive impairments in both MCI and CU subgroups was computed based on Italian normative data. RESULTS: Statistical comparison revealed significant differences among groups in the Ek60 test, with MCI obtaining significantly lower scores than HC and CU, especially for negative emotions. Significant differences were detected also in the SET, with lower performance in emotion and intention attribution for both PD groups compared to HC. A significant correlation emerged between the Ek60 test and emotion attribution. Nine patients showed poor performance at social tasks, five of them being classified as PD-CU. DISCUSSION: Parkinson's disease cognitive profile was characterized by emotion recognition and attribution deficits. These results, as well as the detection of CU patients with isolated socio-cognitive impairments, underline the importance of assessing social cognition in PD as a possible early marker of cognitive decline.

Date 2022
Language eng
License Copyright © 2022 Dodich, Funghi, Meli, Pennacchio, Longo, Malaguti, Di Giacopo, Zappini, Turella and Papagno.
Extra Place: Switzerland
Volume 13
Pages 866809
Publication Frontiers in psychology
DOI 10.3389/fpsyg.2022.866809
Journal Abbr Front Psychol
ISSN 1664-1078
PMID 35645902
PMCID PMC9138611
Date Added 6.7.2025, 19:09:34
Modified 5.9.2025, 14:33:59

Notes:

Included

sample characteristics

size: 32 PD- cognitively unimpaired (CU) and 13 PD-MCI and 45 HC (matched for demographic variables to the patient group)

Parkinson's Disease type and duration: PD-MCI Md=7 (IQR=4-10) PD-CU Md=7 (IQR=3.8-9.75)

Medication: on medication

Hoehn-Yahr: PD-MCI Md=2.5 (IQR=2-2.75) PD-CU Md=2 (IQR=1-2); max=3

UPDRS-3: NA

Gender (male): PD-MCI males=9 (69%) PD-CU males=17 (53%)

averaged ages (SD, range): PD-MCI M=69 SD=8.8 PD-CU M=67.9 SD=6.6, min=50

other neurological disease (tumor, stroke, etc.): None

other major psychopathology: None

origin country (or ethnicity): NA

method observational

instruments used in order to quantify the variables

Social cognition aspect: emotion recognition

Name of the task: Ekman 60-faces test

type of stimulus [face/voice etc., Ekman faces/other etc.]: Ekman faces; static images expressing six basic emotions

task condition: fear, disgust, anger, happiness, sadness, surprise

operationalization: accuracy

Social cognition aspect: ToM

Name of the task: Story-based Empathy Task, SET

type of stimulus [face/voice etc., Ekman faces/other etc.]: non-verbal task developed to assess mental states attribution

task condition: This test includes a sub-test of emotion attribution (SET-EA), as well as a condition of intention attribution (SET-IA) and causal inference (SET-CI)

operationalization: Each condition has a sub-score of a maximum of six points, with a global score of 18 indicating the best possible performance.

Main findings related to the review's scope

Global emotion recognition abilities were reduced in PD-MCI ($p < 0.001$) compared to PD-CU ($p = 0.009$) and HC

PD-MCI patients showed lower scores than HC in the recognition of fear ($p = 0.02$), surprise ($p = 0.002$) and sadness ($p < 0.001$).

Anger ($p = 0.04$) and sadness ($p < 0.001$) recognition was reduced in PD-MCI compared to PD-CU patients.

PD-MCI showed worse performance than HC in SET scores of emotion (SET-EA, $p < 0.001$) and intention (SET-IA, $p < 0.001$) attribution, as well as in the control condition of causal inference (SET-CI, p = 0.03).

SET-EA ($p = 0.002$) and SET-IA ($p = 0.02$) were also reduced in PD-CU compared to HC, as well as in SET-CI.

Tags: Emotion recognition, ToM, behavioral

Degraded Impairment of Emotion Recognition in Parkinson's Disease Extends from Negative to Positive Emotions.

Item Type Journal Article
Author Chia-Yao Lin
Author Yi-Min Tien
Author Jong-Tsun Huang
Author Chon-Haw Tsai
Author Li-Chuan Hsu
Abstract Because of dopaminergic neurodegeneration, patients with Parkinson's disease (PD) show impairment in the recognition of negative facial expressions. In the present study, we aimed to determine whether PD patients with more advanced motor problems would show a much greater deficit in recognition of emotional facial expressions than a control group and whether impairment of emotion recognition would extend to positive emotions. Twenty-nine PD patients and 29 age-matched healthy controls were recruited. Participants were asked to discriminate emotions in Experiment 1 and identify gender in Experiment 2. In Experiment 1, PD patients demonstrated a recognition deficit for negative (sadness and anger) and positive faces. Further analysis showed that only PD patients with high motor dysfunction performed poorly in recognition of happy faces. In Experiment 2, PD patients showed an intact ability for gender identification, and the results eliminated possible abilities in the functions measured in Experiment 2 as alternative explanations for the results of Experiment 1. We concluded that patients' ability to recognize emotions deteriorated as the disease progressed. Recognition of negative emotions was impaired first, and then the impairment extended to positive emotions.
Date 2016
Language eng
Extra Place: Netherlands
Volume 2016
Pages 9287092
Publication Behavioural neurology
DOI 10.1155/2016/9287092

Journal Abbr Behav Neurol
ISSN 1875-8584 0953-4180
PMID 27555668
PMCID PMC4983334
Date Added 6.7.2025, 19:09:35
Modified 5.9.2025, 14:43:50

Notes:

Included

sample characteristics

size: 29 PD and 29 age-match HC

Parkinson's Disease type and duration: idiopathic, NA

Medication: off medication

Hoehn-Yahr: M=2.84 (SD=0.78)

UPDRS-3: M=36.79 (SD=17.29)

Gender (male): 19 males (66%)

averaged ages (SD, range): M=62.93 (SD=12.78)

other neurological disease (tumor, stroke, etc.): None

other major psychopathology: None

origin country (or ethnicity): Chinese

method observational

instruments used in order to quantify the variables

Social cognition aspect: emotion recognition

Name of the task: Fast Emotion Discrimination Task.

type of stimulus [face/voice etc., Ekman faces/other etc.]: Ekman faces (equal male/female).

task condition: happy, sad, fear, anger

operationalization: Participants were asked to discriminate the valence of the target face, positive or negative. >> accuracy and RT: the Efficiency scores were calculated as the proportion of a given participant's mean accuracy divided by that participant's mean RT across all responses in a given experimental condition.

Main findings related to the review's scope

PD performed worse on happiness, sadness and anger, in comparison to HC.

Both groups performed worse on fear, in comparison to other emotions.

Tags: Emotion recognition, behavioral

Dementia and cognitive impairment in patients with Parkinson's disease from India: a 7-year prospective study.

**Item Type**	Journal Article
**Author**	Jaya Sanyal
**Author**	Tapas Kumar Banerjee
**Author**	Vadlamudi Raghavendra Rao
**Abstract**	Depression and cognitive impairment are frequent manifestations in Parkinson's disease (PD). Although a few longitudinal studies have reported on depression and dementia in PD, there is a yet a lack of such studies in India. This 7-year longitudinal study is a hospital-based prospective case (n = 250)-control (n = 280) study. In all, 36.8% had PD with no cognitive impairment (PD-Normal), 27.2% of the patients with PD were affected by dementia (PDD), and 36% of the remaining patients with PD had mild cognitive impairment (PD-MCI) at baseline. After 7 years of evaluation, 32 new patients, 12 patients from the PD-MCI group and 9 patients from the PD-Normal group, were diagnosed with dementia. The 7-year prevalence rate for dementia was estimated to be 49.28%. In the Indian population, an early onset of dementia is noted among patients with PD, with the age of onset being less than 55 years. Patients with early-onset PDD showed depression symptoms that differed significantly from the controls of the same age-group. There was a major difference in verbal fluency, word list recall, constructional praxis and recall, word list recognition, abridged Boston Naming Test, word list memory with repetition, and Mini-Mental State Examination between PD-MCI and PDD groups. Hallucinations before baseline (odds ratio [OR] = 4.427, 95% confidence interval [CI] = 2.122-9.373), akinetic/tremor dominancy (OR = 0.380, 95%CI = 0.149-0.953), and asymmetrical disease onset (OR = 0.3285, 95%CI = 0.1576-0.685) can be considered as risk factors for patients with dementia. Patients with early-onset PD might be more prone to complex depression and dementia. As the disease progresses, akinetic-dominant PD, early hallucinations, and asymmetrical disease onset are the potential risk factors for the development of dementia in patients with PD.
**Date**	2014 Nov
**Language**	eng
**License**	© The Author(s) 2014.
**Extra**	Place: United States
**Volume**	29
**Pages**	630-636
**Publication**	American journal of Alzheimer's disease and other dementias
**DOI**	10.1177/1533317514531442
**Issue**	7
**Journal Abbr**	Am J Alzheimers Dis Other Demen
**ISSN**	1938-2731 1533-3175
**PMID**	24771763
**PMCID**	PMC10852774
**Date Added**	6.7.2025, 19:09:39
**Modified**	5.9.2025, 14:55:40

Notes:

Not Included: Does not study SC
Tags: EXCLUDED

Detection of emotions in Parkinson's disease using higher order spectral features from brain's electrical activity

Item Type Journal Article
Author R. Yuvaraj
Author M. Murugappan
Author Norlinah Mohamed Ibrahim
Author Kenneth Sundaraj
Author Mohd Iqbal Omar
Author Khairiyah Mohamad
Author R. Palaniappan
Abstract Objective Non-motor symptoms in Parkinson's disease (PD) involving cognition and emotion have been progressively receiving more attention in recent times. Electroencephalogram (EEG) signals, being an activity of central nervous system, can reflect the underlying true emotional state of a person. This paper presents a computational framework for classifying PD patients compared to healthy controls (HC) using emotional information from the brain's electrical activity. Approach Emotional EEG data were obtained from 20 PD patients and 20 healthy age-, gender- and education level-matched controls by inducing the six basic emotions of happiness, sadness, fear, anger, surprise and disgust using multimodal (audio and visual) stimuli. In addition, participants were asked to report their subjective affect. Because of the nonlinear and dynamic nature of EEG signals, we utilized higher order spectral features (specifically, bispectrum) for analysis. Two different classifiers namely K-Nearest Neighbor (KNN) and Support Vector Machine (SVM) were used to investigate the performance of the HOS based features to classify each of the six emotional states of PD patients compared to HC. Ten-fold cross-validation method was used for testing the reliability of the classifier results. Main results From the experimental results with our EEG data set, we found that (a) classification performance of bispectrum features across ALL frequency bands is better than individual frequency bands in both the groups using SVM classifier; (b) higher frequency band plays a more important role in emotion activities than lower frequency band; and (c) PD patients showed emotional impairments compared to HC, as demonstrated by a lower classification performance, particularly for negative emotions (sadness, fear, anger and disgust). Significance These results demonstrate the effectiveness of applying EEG features with machine learning techniques to classify the each emotional state difference of PD patients compared to HC, and offer a promising approach for detection of emotional impairments associated with other neurological disorders.
Date 2014
URL https://www.sciencedirect.com/science/article/pii/S1746809414001116
Volume 14
Pages 108-116
Publication Biomedical Signal Processing and Control
DOI https://doi.org/10.1016/j.bspc.2014.07.005

ISSN 1746-8094
Date Added 6.7.2025, 19:12:36
Modified 5.9.2025, 15:02:16

Notes:

Not Included: Same behavioural resultsas study
(OEmotion classification in Parkinson's disease by higher-order spectra and power spectrum features using EEG signals: A comparative study)

Tags: EXCLUDED

Determinants of coping styles of people with Parkinson's Disease

Item Type Journal Article
Author Eva M. Prins
Author Angelika D. Geerlings
Author Yoav Ben-Shlomo
Author Marjan J. Meinders
Author Bastiaan R. Bloem
Author Sirwan K. L. Darweesh
Abstract Little is known about how people with Parkinson's disease (PD) cope with stressful life events. We examined the determinants of specific coping strategies and whether specific choices have any impact on quality of life (QoL). We recruited patients with PD who had been seen at a neurology outpatient clinic at least once during the past year as part of the PRIME-NL cohort study. Coping was measured using the Ways of Coping Questionnaire (WCQ) and QoL was measured using the Parkinson's Disease Questionnaire (PDQ-39). 977 out of 988 participants completed the questionnaires and 935 participants were diagnosed with PD. Factor analysis was undertaken to test if ways of coping were similar or different to previous findings in a PD population. We used linear regression analyses to examine predictors of coping styles. We then used multivariable linear regression to test how coping style was associated with the domains of QoL conditional on potential confounders. The five coping styles identified by the factor analysis were: “taking action and emphasizing the positive”, “distancing and fantasizing”, “goal oriented and planful problem solving”, “seeking social support” and “avoidance and acceptance”. Age, gender, education and anxiety were associated with the type of coping strategy. For example, higher education was associated with more active coping strategies (e.g. & beta; = 4.39, p < 0.001 for goal oriented). Conditional on other confounders, most coping strategies had little effect on QoL domains. These findings demonstrate that coping behavior of people with PD is influenced by psychological status and personal traits. However, there was only a modest effect of coping behavior on QoL. Future research needs to test whether the enhancement or discouragement of certain coping strategies is feasible and can enhance QoL.
Date 2023-06-27
Language English
Extra Place: HEIDELBERGER PLATZ 3, BERLIN, 14197, GERMANY Type: Article
Volume 9

Publisher NATURE PORTFOLIO
Publication NPJ PARKINSONS DISEASE
DOI 10.1038/s41531-023-00548-3
Issue 1
Date Added 14.7.2025, 14:50:27
Modified 14.7.2025, 14:50:27

Notes:

Not Included: Does not study SC
Tags: EXCLUDED

[Diagnosis and Treatment of Cognitive Impairment in Parkinson's Disease].

Item Type Journal Article
Author Tomotaka Shiraishi
Author Hidetomo Murakami
Author Yasuyuki Iguchi
Abstract Parkinson's disease (PD) patients can present with cognitive impairment, such as deficits in attention and memory processes, visual perception, executive function and social cognition. These dysfunctions negatively affect the activities of daily living and outcome of patients. Some of the risk factors, pathological background and etiology of cognitive dysfunction in PD with dementia (PDD) have been determined. Treatments for such dysfunctions are attracting much attention. Some medications, such as donepezil, rivastigmine, and memantine, have shown to improve motor and/or cognitive functions. Cognitive impairment and motor symptoms in PD can be treated concurrently. Therefore, the correlation between motor and cognitive function should be considered when treating PD.
Date 2019 Aug
Language jpn
Extra Place: Japan
Volume 71
Pages 869-874
Publication Brain and nerve = Shinkei kenkyu no shinpo
DOI 10.11477/mf.1416201367
Issue 8
Journal Abbr Brain Nerve
ISSN 1881-6096
PMID 31346143
Date Added 6.7.2025, 19:09:34
Modified 5.9.2025, 14:56:39

Notes:

Not Included: Does not study SC
Tags: EXCLUDED

Diagnosis and Treatment of Parkinson Disease: A Review

**Item Type**	Journal Article
**Author**	Melissa J. Armstrong
**Author**	Michael S. Okun
**Date**	2020-02-11
**Language**	en
**Short Title**	Diagnosis and Treatment of Parkinson Disease
**Library Catalog**	DOI.org (Crossref)
**URL**	https://jamanetwork.com/journals/jama/fullarticle/2760741
**Accessed**	18.1.2026, 22:54:58
**Volume**	323
**Pages**	548
**Publication**	JAMA
**DOI**	10.1001/jama.2019.22360
**Issue**	6
**Journal Abbr**	JAMA
**ISSN**	0098-7484
**Date Added**	18.1.2026, 22:54:58
**Modified**	18.1.2026, 22:54:58

Diagnostic and Statistical Manual of Mental Disorders

**Item Type**	Book
**Author**	American Psychiatric Association
**Date**	2013
**Publisher**	American Psychiatric Publishing
**Edition**	5
**Date Added**	19.1.2026, 7:12:15
**Modified**	19.1.2026, 7:13:29

Did depressive symptoms affect recognition of emotional prosody in Parkinson's disease?

**Item Type**	Journal Article
**Author**	Adriana Vélez Feijó
**Author**	Carlos R. M. Rieder

Author Márcia L. F. Chaves

Abstract OBJECTIVE: Evaluate the influence of depressive symptoms on the recognition of emotional prosody in Parkinson's disease (PD) patients, and identify types of emotion on spoken sentences. METHODS: Thirty-five PD patients and 65 normal participants were studied. Dementia was checked with the Mini Mental State Examination, Clinical Dementia Rating scale, and DSM IV. Recognition of emotional prosody was tested by asking subjects to listen to 12 recorded statements with neutral affective content that were read with a strong affective expression. Subjects had to recognize the correct emotion by one of four descriptors (angry, sad, cheerful, and neutral). The Beck Depression Inventory (BDI) was employed to rate depressive symptoms with the cutoff 14. RESULTS: Total ratings of emotions correctly recognized by participants below and above the BDI cutoff were similar among PD patients and normal individuals. PD patients who correctly identified neutral and anger inflections presented higher rates of depressive symptoms (p = 0.011 and 0.044, respectively). No significant differences were observed in the normal group. CONCLUSIONS: Depression may modify some modalities of emotional prosody perception in PD, by increasing the perception of non-pleasant emotions or lack of affection, such as anger or indifference.

Date 2008 Jun
Language eng
Extra Place: New Zealand
Volume 4
Pages 669-674
Publication Neuropsychiatric disease and treatment
DOI 10.2147/ndt.s1146
Issue 3
Journal Abbr Neuropsychiatr Dis Treat
ISSN 1176-6328 1178-2021
PMID 18830437
PMCID PMC2526379
Date Added 6.7.2025, 19:09:39
Modified 5.9.2025, 14:59:39

Notes:

Included

Sample characteristics

Size: 35 PD, 65 HC (age and sex-alanced)

PD-type: Idiopathic PD

PD-duration: M=6.94 SD= 4.17 (3–17)

Medication: ON state

Hoehn-Yahr: M=2.06 SD= 0.94 (1–4)

UPDRS-3: NA

Gender (male): 18 (51.4%)

Age: M=63.74 SD= 10.20

Other neurological disease (tumor, stroke, etc.): none

Other major psychopathology: none

Origin country (or ethnicity): Brasil

method (Review, meta-analysis or observational and/or self-reported):

instruments used in order to quantify the variables

Social cognition aspect: emotion recognition

Name of the task: NA

Type of stimulus [face/voice etc., Ekman faces/other etc.]: semantically neutral Portuguese sentence (eg, the table is made of wood) was spoken by a female voice

Task condition: four different prosodic emotional descriptors: anger, sadness, happiness and neutral. Globally, subjects heard the recording play semantically neutral sentences 12 times, with three statements for each descriptor.

Operationalization: Patients and normal participants could study the modalities of emotional prosody and answers given to the examiner for each presentation were 0 (“not at all”) or 1 (“very much”) with respect to the labels “angry”, “happy”, “sad”, and “neutral” >> Frequency of correct detected emotion

Main findings related to the review's scope

PD better recognized anger then HC

PD less recognized happiness then HC

no sig dif in Sadness or neutral

Tags: emotion recognition, behavioral

Differential connectivity of the posterior piriform cortex in Parkinson's disease and postviral olfactory dysfunction: an fMRI study.

**Item Type**	Journal Article
**Author**	Charalampos Georgiopoulos
**Author**	Martha Antonia Buechner
**Author**	Bjoern Falkenburger
**Author**	Maria Engström
**Author**	Thomas Hummel
**Author**	Antje Haehner
**Abstract**	Olfactory dysfunction is a common feature of both postviral upper respiratory tract infections (PV) and idiopathic Parkinson's disease (PD). Our aim was to investigate potential differences in the connectivity of the posterior piriform cortex, a major component of the olfactory cortex, between PV and PD patients. Fifteen healthy controls (median age 66 years, 9 men), 15 PV (median age 63 years, 7 men) and 14 PD patients (median age 70 years, 9 men) were examined with task-based olfactory fMRI, including two odors: peach and fish. fMRI data were analyzed with the co-activation pattern (CAP) toolbox, which allows a dynamic temporal assessment of posterior piriform cortex (PPC) connectivity. CAP analysis revealed 2 distinct brain networks interacting with the PPC. The first network included regions related to emotion recognition and attention, such as the anterior cingulate and the middle frontal gyri. The occurrences of this network were significantly fewer in PD patients compared to healthy controls ($p = 0.023$), with no significant differences among PV patients and the other groups. The second network revealed a dissociation between the olfactory cortex (piriform and entorhinal cortices), the anterior cingulate gyrus and the middle frontal gyri. This second network was significantly more active during the latter part of the stimulation, across all groups, possibly due to habituation. Our study shows how the PPC interacts with areas that regulate higher order processing and how this network is substantially affected in PD. Our findings also suggest that olfactory habituation is independent of disease.
**Date**	2024 Mar 15
**Language**	eng
**License**	© 2024. The Author(s).
**Extra**	Place: England
**Volume**	14
**Pages**	6256
**Publication**	Scientific reports
**DOI**	10.1038/s41598-024-56996-1
**Issue**	1
**Journal Abbr**	Sci Rep
**ISSN**	2045-2322
**PMID**	38491209
**PMCID**	PMC10943068
**Date Added**	6.7.2025, 19:09:42
**Modified**	5.9.2025, 14:36:32

Notes:

Not Included: not on SC
Tags: EXCLUDED

Differential Effects of Disease Duration and Dopaminergic Replacement Therapy on Vocal Emotion Recognition in Asymmetric Parkinson's Disease.

**Item Type**	Journal Article
**Author**	Philippe Voruz
**Author**	Didier Grandjean
**Author**	Sophie Drapier
**Author**	Dominique Drapier
**Author**	Marc Vérin
**Author**	Julie Anne Péron
**Abstract**	INTRODUCTION: Recently, studies have suggested a role of motor symptom asymmetry on impaired emotional recognition abilities in Parkinson's disease with a greater vulnerability in patients with a predominance of left-sided symptoms. However, none of them explored the interaction between motor symptom asymmetry and dopamine replacement therapy in different stages of the disease. METHODOLOGY: We explored the recognition of vocal emotion (i.e., emotional prosody) in 15 newly diagnosed Parkinson's disease patients in the early stages of the disease, 15 patients in the advanced stages of the disease and 15 healthy controls. The early patients were studied in two conditions: ON and OFF dopaminergic replacement therapy and both Parkinson's disease groups (early and advanced) were divided into two subgroups according to the asymmetry of motor symptoms. RESULTS: The analyses revealed two patterns of results. First, as predicted, we observed a reduction in performance for the recognition of vocal emotions in patients with a predominance of left-sided symptoms as compared to both healthy controls and predominantly right-sided symptom patients. Second, in the early stages of the disease, we observed a deleterious effect of dopatherapy on the recognition of vocal emotions for the patients with left-predominant symptoms, and the inverse pattern (i.e., a positive effect of dopatherapy) for the patients with right-predominant symptoms. CONCLUSIONS: Our results bring to knowledge the differential effects of disease duration, dopaminergic replacement therapy and motor symptom asymmetry on vocal emotion recognition in Parkinson's disease.
**Date**	2024
**Language**	eng
**License**	© 2024 S. Karger AG, Basel.
**Extra**	Place: Switzerland
**Volume**	24
**Pages**	129-140
**Publication**	Neuro-degenerative diseases
**DOI**	10.1159/000542337
**Issue**	3-4
**Journal Abbr**	Neurodegener Dis
**ISSN**	1660-2862 1660-2854
**PMID**	39681097

Date Added 6.7.2025, 19:09:35
Modified 5.9.2025, 14:59:55

Notes:

Included

Sample characteristics

Size: 8 early-RPD (early stage < 5 years duration); 7 early-LPD; 8 adv-RPD (advanced PD); 7 adv-LPD; 15 HC

PD-type: Idiopathic PD

PD-duration: early-RPD: M = 2.88, SD = 0.99; early LPD: M = 2.71, Sd = 1.55; adv-RPD: M = 11.13, SD = 3.98; adv-LPD: M = 11.14, SD = 2.91

Medication: ON state

Hoehn-Yahr: early-RPD: M = 057, SD = 0.79; early-LPD: M = 0.71, SD = 0.76; adv-RPD: M = 1.50, SD = 0.9, adv-LPD: M = 1.21, SD = 0.64

UPDRS-3: (only described for adv-groups): adv-RPD: M = 8.38, SD = 4.65, adv-LPD: M = 8.14, SD = 5.81

Gender (male): early-RPD: 6 (75%); early-LPD: 4 (57%); adv-RPPD: 6 (75%); adv-LPD: 4 (57%)

Age: (early-RPD: 63.13 ± 4.27, early-LPD: 56.86 ± 8.90, adv-RPD: 55.13 ± 6.01, adv-LPD: 64.43 ± 8.90

Other neurological disease (tumor, stroke, etc.): none

Other major psychopathology: none

Origin country (or ethnicity): NA

method (Review, meta-analysis or observational and/or self-reported):

instruments used in order to quantify the variables

Social cognition aspect: Emotion recognition

Name of the task: NA

Type of stimulus [face/voice etc., Ekman faces/other etc.]: 60 vocal stimuli, consisting of short pseudowords of meaningless speech composing pseudowords pseudoword.

Task condition: five different prosodies (anger, fear, happiness, neutral, and sadness)

Operationalization: participants rated each stimulus on six scales: one scale for each featured emotion (anger, fear, happiness, and sadness), one for neutral, and one for the surprise emotion >> Rating of each emotion on VAS-scale

Main findings related to the review's scope

LPD patients exhibited greater deficits in emotion recognition compared to both RPD patients and healthy controls, particularly for the recognition of fear and neutral emotions. No other sig dif (anger, happiness, sadness).

early LPD patients showed a decline in performance under dopamine medication, especially for recognizing anger. No other sig dif.

Tags: Emotion recognition, behavioral

Differentiation of claustrum resting-state functional connectivity in healthy aging, Alzheimer's disease, and Parkinson's disease

Item Type Journal Article
Author Sevilay Ayyildiz
Author Halil Aziz Velioglu
Author Behcet Ayyildiz
Author Bernis Sutcubasi
Author Lutfu Hanoglu
Author Zubeyir Bayraktaroglu
Author Suleyman Yildirim
Author Alper Atasever
Author Burak Yulug
Abstract The claustrum is a sheet-like of telencephalic gray matter structure whose function is poorly understood. The claustrum is considered a multimodal computing network due to its reciprocal connections with almost all cortical areas as well as subcortical structures. Although the claustrum has been involved in several neurodegenerative diseases, specific changes in connections of the claustrum remain unclear in Alzheimer's disease (AD), and Parkinson's disease (PD). Resting-state fMRI and T1-weighted structural 3D images from healthy elderly (n = 15), AD (n = 16), and PD (n = 12) subjects were analyzed. Seed-based FC analysis was performed using CONN FC toolbox and T1-weighted images were analyzed with the Computational Anatomy Toolbox for voxel-based morphometry analysis. While we observed a decreased FC between the left claustrum and sensorimotor cortex, auditory association cortex, and cortical regions associated with social cognition in PD compared with the healthy control group (HC), no significant difference was found in alterations in the FC of both claustrum comparing the HC and AD groups. In the AD group, high FC of claustrum with regions of sensorimotor cortex and cortical regions related to cognitive control, including cingulate gyrus, supramarginal gyrus, and insular cortex were demonstrated. In addition, the structural results show significantly decreased volume in bilateral claustrum in AD and PD compared with HC. There were no significant differences in the claustrum volumes between PD and AD groups so the FC may offer more precise findings in distinguishing changes for claustrum in AD and PD.
Date 2023-03
Language English
Extra Place: 111 RIVER ST, HOBOKEN 07030-5774, NJ USA Type: Article
Volume 44
Publisher WILEY
Pages 1741-1750
Publication HUMAN BRAIN MAPPING
DOI 10.1002/hbm.26171
Issue 4
ISSN 1065-9471

Date Added 14.7.2025, 14:50:28
Modified 5.9.2025, 14:27:14

Notes:

Not Included: not on SC
Tags: EXCLUDED

Dimensions of apathy in Parkinson's disease

Item Type Journal Article
Author Nasya Thompson
Author Michael MacAskill
Author Maddie Pascoe
Author Tim Anderson
Author Campbell Le Heron
Abstract IntroductionApathy is one of the most common neuropsychiatric manifestations in Parkinson's disease (PD). Recent proposals consider apathy as a multidimensional construct, which can manifest in behavioral, cognitive, emotional, and/or social dimensions. Apathy also overlaps conceptually and clinically with other non-motor comorbidities, particularly depression. Whether all of these dimensions are applicable to the apathetic syndrome experienced by people with PD is unclear. In the present study, we investigated the multidimensional pattern of apathy associated with PD, using the recently developed Apathy Motivation Index (AMI) which probes behavioral, emotional, and social apathy dimensions. We then examined the relationship between these dimensions and other features of PD commonly associated with apathy, including depression, anxiety, cognition, and motor state. MethodsA total of 211 participants were identified from the New Zealand Brain Research Institute (NZBRI) longitudinal PD cohort. One hundred eight patients and 45 controls completed the AMI, administered as an online questionnaire, and additional assessments including neuropsychiatric, neuropsychological, and motor scores. The pattern of dimensional apathy in PD was assessed using a repeated-measured analysis of variance, while simple linear regressions were performed to evaluate relationships between these dimensions and other variables. ResultsWe found a significant interaction between group (PD versus control) and apathy subscale, driven mainly by higher levels of social and behavioral-but not emotional-apathy in those with PD. This result was strikingly similar to a previous study investigating social apathy in PD. Distinct patterns of dimensional apathy were associated with depression and anxiety, with social and behavioral apathy positively associated with depression, and emotional apathy negatively associated with anxiety. ConclusionThis work provides further evidence for a distinct pattern of apathy in people with PD in which deficits manifest in some-but not all-dimensions of motivated behavior. It emphasizes the importance of considering apathy as a multidimensional construct in clinical and research settings.
Date 2023-06
Language English
Extra Place: 111 RIVER ST, HOBOKEN, NJ 07030 USA Type: Article
Volume 13

Publisher WILEY
Publication BRAIN AND BEHAVIOR
DOI 10.1002/brb3.2862
Issue 6
ISSN 2162-3279
Date Added 14.7.2025, 14:50:28
Modified 5.9.2025, 14:58:29

Notes:

Not Included: Does not study SC
Tags: EXCLUDED

Discriminating facial expressions of emotion and its link with perceiving visual form in Parkinson's disease.

Item Type Journal Article
Author Michelle Marneweck
Author Geoff Hammond
Abstract We investigated the link between the ability to perceive facial expressions of emotion and the ability to perceive visual form in Parkinson's disease (PD). We assessed in individuals with PD and healthy controls the ability to discriminate graded intensities of facial expressions of anger from neutral expressions and the ability to discriminate radial frequency (RF) patterns with modulations in amplitude from a perfect circle. Those with PD were, as a group, impaired relative to controls in discriminating graded intensities of angry from neutral expressions and discriminating modulated amplitudes of RF patterns from perfect circles; these two abilities correlated positively and moderately to highly, even after removing the variance that was shared with disease progression and general cognitive functioning. The results indicate that the impaired ability to perceive visual form is likely to contribute to the impaired ability to perceive facial expressions of emotion in PD, and that both are related to the progression of the disease.
Date 2014 Nov 15
Language eng
License Copyright © 2014 Elsevier B.V. All rights reserved.
Extra Place: Netherlands
Volume 346
Pages 149-155
Publication Journal of the neurological sciences
DOI 10.1016/j.jns.2014.08.014
Issue 1-2
Journal Abbr J Neurol Sci
ISSN 1878-5883 0022-510X
PMID 25179875
Date Added 6.7.2025, 19:09:38

Modified 5.9.2025, 14:45:02

Notes:

Included

Sample characteristics

Size: PD = 24, HC = 18 (well matched for age, sex and MoCA and GDS)

PD-type: NA

PD-duration: Md=8 (2–22)

Medication: OFF medication

Hoehn-Yahr: Md=2 (1–2)

UPDRS-3: Md=40 (19–57)

Gender (male): 16 (66%)

Age: Md=68 (58–82)

Other neurological disease (tumor, stroke, etc.): NA

Other major psychopathology: NA

Origin country (or ethnicity): NA

method (Review, meta-analysis or observational and/or self-reported):

instruments used in order to quantify the variables

Social cognition aspect: Emotion recognition (facial expressions)

Name of the task: NA

Type of stimulus [face/voice etc., Ekman faces/other etc.]: For the measure of emotion discrimination, we selected four models expressing full-blown anger from the NimStim Face Stimulus Set; we graded these expressions in emotional intensity by morphing fullblown expressions of each model with their neutral expression

Task condition:

two-interval forced choice (2IFC): On each trial of the 2IFC procedure, two faces of the same model appeared successively on a computer screen at a duration of 200 ms or 1000 ms with a 200-ms blank interstimulus interval. The face with the neutral expression appeared randomly in either the first or the second interval and the face expressing one of five levels of intensity of anger (set at 5, 9, 16, 29, and 52% of the full-blown expression) appeared in the other interval. On each trial participants signaled the interval containing the angry face by clicking either the left or right button on a mouse to indicate the first or second interval respectively. There were 10 randomized blocks of 40 trials (with a break after the fifth block), with each block containing five intensities of anger each expressed by each of the four models at two stimulus durations, giving 400 trials in total.

Yes-No: For the emotion discrimination measure with the yes– no procedure, each trial showed one face either with a neutral expression or with an expression of anger at one of three emotional intensities (9%, 16%, 29%); each face was presented at a duration of 200 ms or 1000 ms. Participants indicated with mouse-click whether the face was emotional by clicking the left mouse button or neutral by clicking the right mouse button. There were ten randomized blocks of 48 trials (with a break given after the fifth block) with each block containing three neutral expressions and three intensities of anger by

each of four models at two stimulus durations, giving 480 trials in total.

Operationalization: Correct responses

Main findings related to the review's scope

PD performed significantly worse than HC in discriminating angry from neutral expressions.

Sensitivity to expressions of anger increased with increasing intensity of the expression: The PD group showed lower sensitivity than the controls at both stimulus durations.

As was the case for the 2IFC emotion discrimination measure, there was a significant main effect of group (F (1, 40) = 19.94, p b .05), with large effect sizes of the mean group differences across intensity increments for both stimulus durations.

Tags: emotion recognition, behavioral

Discrimination and categorization of emotional facial expressions and faces in Parkinson's disease.

Item Type Journal Article

Author Laura Alonso-Recio

Author Pilar Martín

Author Sandra Rubio

Author Juan M. Serrano

Abstract Our objective was to compare the ability to discriminate and categorize emotional facial expressions (EFEs) and facial identity characteristics (age and/or gender) in a group of 53 individuals with Parkinson's disease (PD) and another group of 53 healthy subjects. On the one hand, by means of discrimination and identification tasks, we compared two stages in the visual recognition process that could be selectively affected in individuals with PD. On the other hand, facial expression versus gender and age comparison permits us to contrast whether the emotional or non-emotional content influences the configural perception of faces. In Experiment I, we did not find differences between groups, either with facial expression or age, in discrimination tasks. Conversely, in Experiment II, we found differences between the groups, but only in the EFE identification task. Taken together, our results indicate that configural perception of faces does not seem to be globally impaired in PD. However, this ability is selectively altered when the categorization of emotional faces is required. A deeper assessment of the PD group indicated that decline in facial expression categorization is more evident in a subgroup of patients with higher global impairment (motor and cognitive). Taken together, these results suggest that the problems found in facial expression recognition may be associated with the progressive neuronal loss in frontostriatal and mesolimbic circuits, which characterizes PD.

Date 2014 Sep

Language eng

License © 2013 The British Psychological Society.

Extra Place: England

Volume 8

Pages 269-288

Publication Journal of neuropsychology
DOI 10.1111/jnp.12029
Issue 2
Journal Abbr J Neuropsychol
ISSN 1748-6653 1748-6645
PMID 23992026
Date Added 6.7.2025, 19:09:37
Modified 5.9.2025, 14:25:48

Notes:

Included

sample characteristics

size: 53 PD and 53 HC matched for sex, educational level, and general cognitive abilities

Parkinson's Disease type and duration: idiopathic PD, Mduration=6.57 years (*SD* = 4.01)

Medication: on medication

Hoehn-Yahr: Md=2

UPDRS-3: NA

Gender (male): 22 males (42%)

averaged ages (SD, range): M= 65.77 SD= 6.71

other neurological disease (tumor, stroke, etc.): None

other major psychopathology: None

origin country (or ethnicity): Spain

method observational

instruments used in order to quantify the variables

Social cognition aspect: emotion recognition

Name of the task: NA

type of stimulus [face/voice etc., Ekman faces/other etc.]: Stimuli were composed of 80 photographs of faces from the FACES Database divided into two sets of 40 images to obtain 20 pairs each. Hair and background were removed from all pictures to provide only facial information for the recognition of emotional facial expressions (EFE) or gender, and the size of each picture was 6.1 × 9 cm. Of all the 80 images used to obtain the 40 pairs, 20 young women/men (between 19 and 44 years) and 20 older women/men (between 46 and 80 years). The number of men and women, as well as young and older ones, was the same in the EFE and gender set.

task condition: 10 of the same EFE pairs showing happiness, sadness, anger, fear, and disgust (two pairs for each emotion), and 10 pairs with different EFEs (happiness-fear, happiness-disgust, happiness-anger, happiness-sadness, fear-disgust, fear-anger, fear-sadness, disgust-anger, disgust-sadness, anger-sadness)

operationalization: number of correct responses

Main findings related to the review's scope

Analyses revealed no statistically significant Group × Stimuli interaction, or main effects of group

No differences were found in correct responses when PD and HC individuals had to judge similarity or differences between the pair presented, nor with EFE or gender judgements

Tags: Emotion recognition, behavioral

Discrimination and recognition of facial expressions of emotion and their links with voluntary control of facial musculature in Parkinson's disease.

Item Type Journal Article
Author Michelle Marneweck
Author Romina Palermo
Author Geoff Hammond
Abstract OBJECTIVE: To explore perception of facial expressions of emotion and its link with voluntary facial musculature control in Parkinson's disease (PD). METHOD: We investigated in 2 sets of experiments in PD patients and healthy controls the perceptual ability to discriminate (a) graded intensities of emotional from neutral expressions, (b) graded intensities of the same emotional expressions, (c) full-blown discrepant emotional expressions from 2 similar expressions and the more complex recognition ability to label full-blown emotional expressions. We tested an embodied simulationist account of emotion perception in PD, which predicts a link between the ability to perceive emotional expressions and facial musculature control. We also explored the contribution of the ability to extract facial information (besides emotion) to emotion perception in PD. RESULTS: Those with PD were, as a group, impaired relative to controls (with large effect sizes) in all measures of discrimination and recognition of emotional expressions, although some patients performed as well as the best performing controls. In support of embodied simulation, discrimination and recognition of emotional expressions correlated positively with voluntary control of facial musculature (after partialing out disease severity and age). Patients were also impaired at extracting information other than emotion from faces, specifically discriminating and recognizing identity from faces (with large effect sizes); identity discrimination correlated positively with emotion discrimination and recognition but not with voluntary facial musculature control (after partialing out disease severity and age). CONCLUSIONS: The results indicate that impaired sensory and sensorimotor processes, which are a function of disease severity, affect emotion perception in PD.
Date 2014 Nov
Language eng
License PsycINFO Database Record (c) 2014 APA, all rights reserved.
Extra Place: United States
Volume 28
Pages 917-928
Publication Neuropsychology
DOI 10.1037/neu0000106
Issue 6
Journal Abbr Neuropsychology
ISSN 1931-1559 0894-4105
PMID 24933489
Date Added 6.7.2025, 19:09:36
Modified 5.9.2025, 14:45:08

Notes:

Included

2 Experiments reported in the paper with different samples.

Expriment 1:

Sample characteristics

Size: 34 PD, 32 HC (well-matched for age and scores on measures of general cognitive functioning and depressive symptoms)

PD-type: NA

PD-duration: Med = 5; Range = 1–19

Medication: OFF medication

Hoehn-Yahr:NA

UPDRS-3: Med = 38; Range = 10-56

Gender (male): 20 (59%)

Age: Med = 66; Range = 46–80

Other neurological disease (tumor, stroke, etc.): NA

Other major psychopathology: Little depressive symptomatology as measured by GDS (both exp)

Origin country (or ethnicity): Australia

method observational

instruments used in order to quantify the variables

Social cognition aspect: emotion recognition

Name of the task: NA

Type of stimulus [face/voice etc., Ekman faces/other etc.]: Faces with neutral/emotional expressions (emotions, anger, disgust, happiness, and sadness). 7 different levels of emotion intensity.

Task condition:

1. Forced-choice. Discriminate neutral from emotional expression.
2. Forced-choice. Discriminate between lower and higher emotional intensity of the same emotion. Choose higher intensity
3. Overall: There were a total of 280 trials with 40 trials for each intensity increment.

Operationalization: Number of correct answers.

Social cognition aspect: emotion recognition

Name of the task: NA

Type of stimulus [face/voice etc., Ekman faces/other etc.]: Discriminating discrepant from similar expressions of fullblown intensity (same for exp1 and exp2). A total of 100 target faces (Karolinska Directed Emotional Faces database, Lundqvist, Flykt, & Öhman, 1998) expressed anger, disgust,

sadness, happiness, fear, and surprise at full-blown emotional intensity.

Task condition: Three-alternative forced choice. Discriminate the discrepant emotional facial expression from two expressions of the same emotion

Operationalization: Number of correct answers.

Social cognition aspect: emotion recognition

Name of the task: NA; Verbally labeling emotional expressions at full-blown intensity (same for exp1 and exp2)

Type of stimulus [face/voice etc., Ekman faces/other etc.]: 144 faces (Lundqvist et al., 1998). 24 for each of 6 emotion categories (anger, disgust, fear, happy, sad, surprise)

Task condition: Six-alternative forced-choice measure. A total of 144 target faces

Main findings related to the review's scope

Patient group performed more poorly than controls on all measures and that the group difference

Effect sizes were substantially greater when comparing control performance with those patients in the high-severity band than those in the low-severity band

Experiment 2

One year after experiment 1. 40 participated in both experiments. 26 unique to Exp. 1, 9 unique to Exp. 2.

Sample characteristics

Size: 25 PD, 24 HC (well-matched for age and scores on measures of general cognitive functioning and depressive symptoms)

PD-type: NA

PD-duration: Md = 7; Range = 1–20

Medication: Test 1-1.5 hours before intake of anit-parkinson medication. L-Dopa-equivalent dose mentioned.

Hoehn-Yahr: NA

UPDRS-3: Md = 41; Range = 19-56

Gender (male): 17 (68%)

Age: Med = 67; Range = 53–81

Other neurological disease (tumor, stroke, etc.): NA

Other major psychopathology: NA

method

instruments used in order to quantify the variables

Social cognition aspect: emotion recognition

Name of the task: NA

Type of stimulus [face/voice etc., Ekman faces/other etc.]: Faces with neutral/emotional expressions (emotions, anger, disgust, happiness, and sadness). 7 different levels of emotion intensity.

Task condition: There were 240 trials with 48 trials for each intensity difference. Higher range of emotional intensity than Exp. 1.

Operationalization: Number of correct answers.

Main findings related to the review's scope

The patient group again performed more poorly than control group, with the group difference increasing with stimulus separation, and with some patients performing as well as some of the best-performing controls.

Patients performed more poorly than controls when discriminating discrepant from similar expressions (g .92) and when verbally labeling emotions (g .96).

Tags: Emotion recognition, Behavioral

Disgust-specific impairment of facial emotion recognition in Parkinson's disease patients with mild cognitive impairment.

Item Type Journal Article

Author Ke-Wei Chiang
Author Chun-Hsiang Tan
Author Wei-Pin Hong
Author Rwei-Ling Yu

Abstract This study investigated the association between cognitive function and facial emotion recognition (FER) in patients with Parkinson's disease (PD) and mild cognitive impairment (PD-MCI). We enrolled 126 participants from Taiwan, including 63 patients with idiopathic PD and 63 matched healthy controls. The PD group was divided into two groups: those with normal cognitive function (PD-NC) and those with MCI (PD-MCI). Participants underwent a modality emotion recognition test and comprehensive cognitive assessment. Our findings reveal that patients with PD-MCI exhibit significantly impaired FER, especially in recognizing "disgust," compared with patients with PD-NC and healthy adults (P = .001). This deficit correlates with executive function, attention, memory, and visuospatial abilities. Attention mediates the relationship between executive function and "disgust" FER. The findings highlight how patients with PD-MCI are specifically challenged when recognizing "disgust" and suggest that cognitive training focusing on cognitive flexibility and attention may improve their FER abilities. This study contributes to our understanding of the nuanced relationship between cognitive dysfunction and FER in patients with PD-MCI, emphasizing the need for targeted interventions.

Date 2024 Nov 5
Language eng
License © The Author(s) 2024. Published by Oxford University Press.
Extra Place: England
Volume 19

Publication Social cognitive and affective neuroscience
DOI 10.1093/scan/nsae073
Issue 1
Journal Abbr Soc Cogn Affect Neurosci
ISSN 1749-5024 1749-5016
PMID 39417289
PMCID PMC11561469
Date Added 6.7.2025, 19:09:36
Modified 5.9.2025, 14:30:40

Notes:

Included

sample characteristics

size: 63 idiopathic PD (divided into PD-MCI and PD-NC [normal cognition]) and 63 HC age-, sex-, and education-matched

Parkinson's Disease type and duration: idiopathic, Mduration=6.14 (SD=4.97)

Medication: mixed; off/on medication

Hoehn-Yahr: M= 1.81 (SD=0.62)

UPDRS-3: M= 32.57 (SD=10.96)

Gender (male): 43 males (68%)

averaged ages (SD, range): M= 62.97 (SD=8.49), 47–78

other neurological disease (tumor, stroke, etc.): None

other major psychopathology: NA

origin country (or ethnicity): Taiwan

method observational

instruments used in order to quantify the variables

Social cognition aspect: emotion recognition

Name of the task: the MMER app

type of stimulus [face/voice etc., Ekman faces/other etc.]: FER subtests (i.e. Subtest 3 and Subtest 4). Subtest 3, participants were presented with a face on a tablet along with seven emotion words. They were required to select the word that best matched the facial expressions.

In Subtest 4, the setup was reversed, displaying an emotion word with seven corresponding faces. Participants were required to identify a face that accurately represented a given emotion.

task condition: neutral, happy, sad, anger, disgust, fear, and surprise

operationalization: accuracy

Main findings related to the review's scope

Comparing the three groups, there was a significant difference in total score of subset 3 and 4, resulting from low performance of the PD-MCI group, while no differences were between HC and PD-NC.

no emotion-specific groups comparisons survived Bonferroni correction
Tags: Emotion recognition, behavioral

Disgust-specific impairment of facial expression recognition in Parkinson's disease.

**Item Type**	Journal Article
**Author**	Atsunobu Suzuki
**Author**	Takahiro Hoshino
**Author**	Kazuo Shigemasu
**Author**	Mitsuru Kawamura
**Abstract**	There is contradictory evidence regarding whether the impairments of the recognition of emotional facial expressions in Parkinson's disease are specific to certain emotions such as disgust and fear. Generally, neurological case reports on emotion-specific impairments have been suspected of being confounded with the factor of task difficulty. Using a refined assessment method in which the difficulty factors were controlled by means of mixed facial expressions and item response theory, we attempted to clarify whether Parkinson's disease disproportionately impaired the recognition of specific emotions. We studied 14 patients with Parkinson's disease and 39 healthy controls who were matched in terms of gender, age, years of education and intelligence quotient. Whereas the refined method revealed that the patients with Parkinson's disease displayed significantly lower scores in disgust recognition alone, conventional methods failed to detect this impairment. In addition, control measures including face recognition abilities did not statistically explain the impairment observed in the patients. The results indicate that Parkinson's disease can indeed selectively impair the recognition of facial expressions of disgust; this provides concrete evidence for emotion-specific impairments that sufficiently withstands criticisms regarding the difficulty artefacts. Furthermore, the results support the proposed role of the basal ganglia-insula system in disgust recognition. This study effectively demonstrates the benefits of refining neuropsychological assessment by taking advantage of the modern psychometric theory.
**Date**	2006 Mar
**Language**	eng
**Extra**	Place: England
**Volume**	129
**Pages**	707-717
**Publication**	Brain : a journal of neurology
**DOI**	10.1093/brain/awl011
**Issue**	Pt 3
**Journal Abbr**	Brain
**ISSN**	1460-2156 0006-8950
**PMID**	16415306
**Date Added**	6.7.2025, 19:09:38
**Modified**	5.9.2025, 14:57:56

Notes:

Included

Sample characteristics

Size: 14 PD, 39 HC (matched for gender, age, education, IQ)

PD-type: Idiopathic PD (early)

PD-duration: M = 4.79, SE = 1

Medication: ON state

Hoehn-Yahr: Range: 1-3; M=1.57 SD=0.65

UPDRS-3: NA

Gender (male): 5 (36%)

Age: M = 67.1, SE = 1.8

Other neurological disease (tumor, stroke, etc.): NA

Other major psychopathology: None

Origin country (or ethnicity): Japan

method behavioral

instruments used in order to quantify the variables

Social cognition aspect: emotion recognition

Name of the task: NA

Type of stimulus [face/voice etc., Ekman faces/other etc.]: 72 grey-scale photographs of facial expressions. 36 of caucasian male, 36 of japanese female. six basic emotion.

Task condition: happiness, surprise, fear, anger, disgust and sadness.

Operationalization:

1. Sensitivity score: rate the emotional intensity. The ratings were made on a 6-point scale from 0 ('not at all') to 5 ('very much'). Each participant's sensitivity to a given emotion was scored by applying the graded-response model (GRM; Samejima, 1969) to the participant's ratings of the 22 stimuli depicting the relevant emotion.
2. Identification accuracy
3. Hexagon morphed faces >> accuracy

Main findings related to the review's scope

in the accuracy scores (method 2+3), no sig dif.

sensitivity analysis: Significant decrease in the sensitivity scores for disgust among the Parkinson's disease patients. None of the other comparisons reached statistical significance

Tags: Emotion recognition, behavioral

Disorders of facial emotional expression and comprehension.

Item Type Journal Article
Author Kenneth M. Heilman
Abstract One of the most important means of communicating emotions is by facial expressions. About 30-40 years ago, several studies examined patients with right and left hemisphere strokes for deficits in expressing and comprehending emotional facial expressions. The participants with right- or left-hemispheric strokes attempted to determine if two different actors were displaying the same or different emotions, to name the different emotions being displayed, and to select the face displaying an emotion named by the examiner. Investigators found that the right hemisphere-damaged group was impaired on all these emotional facial tests and that this deficit was not solely related to visuoperceptual processing defects. Further studies revealed that the patients who were impaired at recognizing emotional facial expressions and who had lost these visual representations of emotional faces often had damage to their right parietal lobe and their right somatosensory cortex. Injury to the cerebellum has been reported to impair emotional facial recognition, as have dementing diseases such as Alzheimer's disease and frontotemporal dementia, movement disorders such as Parkinson's disease and Huntington's disease, traumatic brain injuries, and temporal lobe epilepsy. Patients with right hemisphere injury are also more impaired than left-hemisphere-damaged patients when attempting to voluntarily produce facial emotional expressions and in their spontaneous expression of emotions in response to stimuli. This impairment does not appear to be induced by emotional conceptual deficits or an inability to experience emotions. Many of the disorders that cause impairments of comprehension of affective facial expressions also impair facial emotional expression. Treating the underlying disease may help patients with impairments of facial emotion recognition and expression, but unfortunately, there have not been many studies of rehabilitation.
Date 2021
Language eng
License Copyright © 2021 Elsevier B.V. All rights reserved.
Extra Place: Netherlands
Volume 183
Pages 99-108
Publication Handbook of clinical neurology
DOI 10.1016/B978-0-12-822290-4.00006-2
Journal Abbr Handb Clin Neurol
ISSN 0072-9752
PMID 34389127
Date Added 6.7.2025, 19:09:37
Modified 5.9.2025, 14:38:44

Notes:

Not Included: a book chapter.

Tags: EXCLUDED

Disrupted identities: movement, mind, and memory in Parkinson's disease

Item Type Journal Article
Author Iracema Leroi
Abstract For the many clinicians au fait with the history of the clinical description of Parkinson's disease, they will be aware that the very earliest description of James Parkinson's “Shaking Palsy” in 1817 explicitly excluded the involvement of cognitive and emotional processes as manifestations of the disease. Within a short time following his treatise, it became all too clear to those in the field that Parkinson's disease is more than just a motor disorder, and as was aptly conceptualized by Paul McHugh, Professor of Psychiatry at Johns Hopkins Hospital from 1975 until 2001, Parkinson's disease is closer to being a “triadic disorder,” encompassing motor, cognitive, and psychiatric elements (McHugh, 1989). Even this notion is now outdated, with the triad being accompanied by autonomic, pain, and other non-motor syndromes.
Date 2017
URL https://www.sciencedirect.com/science/article/pii/S1041610224017241
Volume 29
Pages 879-881
Publication International Psychogeriatrics
DOI https://doi.org/10.1017/S1041610217000370
Issue 6
ISSN 1041-6102
Date Added 6.7.2025, 19:12:34
Modified 6.7.2025, 19:12:34

Notes:

Not Included: Editorial letter
Tags: EXCLUDED

Disruption of Inferior Longitudinal Fasciculus Microstructure in Parkinson's Disease: A Systematic Review of Diffusion Tensor Imaging Studies.

Item Type Journal Article
Author Maryam Haghshomar
Author Mahsa Dolatshahi
Author Farzaneh Ghazi Sherbaf
Author Hossein Sanjari Moghaddam
Author Mehdi Shirin Shandiz
Author Mohammad Hadi Aarabi
Abstract Parkinson's disease (PD) is a neurodegenerative disorder accompanied by a series of pathological mechanisms which contribute to a variety of motor and non-motor symptoms. Recently, there has been an increasing interest in structural diffusion tensor imaging (DTI) in PD which has shed light on our understanding of structural

abnormalities underlying PD symptoms or its associations with pathological mechanisms. One of the white matter tracts shown to be disrupted in PD with a possible contribution to some PD symptoms is the inferior longitudinal fasciculus (ILF). On the whole, lower ILF integrity contributes to thought disorders, impaired visual emotions, cognitive impairments such as semantic fluency deficits, and mood disorders. This review outlines the microstructural changes in ILF associated with systemic inflammation and various PD symptoms like cognitive decline, facial emotion recognition deficit, depression, color discrimination deficit, olfactory dysfunction, and tremor genesis. However, few studies have investigated DTI correlates of each symptom and larger studies with standardized imaging protocols are required to extend these preliminary findings and lead to more promising results.

**Date**	2018
**Language**	eng
**Extra**	Place: Switzerland
**Volume**	9
**Pages**	598
**Publication**	Frontiers in neurology
**DOI**	10.3389/fneur.2018.00598
**Journal Abbr**	Front Neurol
**ISSN**	1664-2295
**PMID**	30093877
**PMCID**	PMC6070770
**Date Added**	6.7.2025, 19:09:42
**Modified**	5.9.2025, 14:37:28

Notes:

Not Included – although systematic review, included only one relevant paper in regards to this SR.

We performed a systematic search of the published literature to identify the studies that investigated the involvement of ILF disruptions associated with PD pathology and symptomatology using DTI. We used the broad search terms: "Diffusion Tensor imaging OR Diffusion tensor MRI OR Diffusion MRI OR DTI" AND "Parkinson's disease OR Parkinson disease OR PD." We searched electronic databases including Embace, Scopus, and PubMed from Studies which had investigated diseases other than PD, or had used imaging methods other than DTI were excluded. Full papers were obtained for studies published in English that performed DTI in PD group, and further assessed if they had investigated whole WM microstructure or particularly assessed one or more WM tracts including ILF in PD patients to be included in this systematic review.1980 to May 2018.

N = 45 (??) – practically – only one paper….

Main findings related to the review's scope

emotion recognition relies on numerous neural substrates; In the presence of a target (a facial emotion), the orbitofrontal cortex (OFC) is activated, and it is, on the other hand, associated with the functional connectivity between a particular region of the right OFC and bilateral visual brain regions [i.e., the inferior occipital gyrus (IOG)] (123). Therefore, OFC and IOG connections consisting of some structures like ILF are associated with facial emotion recognition. To support this, DTI studies have shown that in children with object recognition deficit (124) and also in patients with progressive prosopagnosia (isolated deficit in recognition of facial emotions) (125), ILF integrity is compromised. In PD patients, H.C. Baggio et al. have studied the relationship between the capacity to recognize specific emotions in facial expressions and gray and white matter structural parameters (59). They detected a positive correlation between FA in left ILF and sadness scores despite no significant difference in FA values of ILF between PD and HC.

Tags: EXCLUDED

Dissociating cognitive from affective theory of mind: a TMS study

Item Type Journal Article

Author Elke Kalbe
Author Marius Schlegel
Author Alexander T. Sack
Author Dennis A. Nowak
Author Manuel Dafotakis
Author Christopher Bangard
Author Matthias Brand
Author Simone Shamay-Tsoory
Author Oezguer A. Onur
Author Josef Kessler

Abstract INTRODUCTION: "Theory of Mind" (ToM), i.e., the ability to infer other persons' mental states, is a key function of social cognition. It is increasingly recognized to form a multidimensional construct. One differentiation that has been proposed is that between cognitive and affective ToM, whose neural correlates remain to be

identified. We aimed to ascertain the possible role of the right dorsolateral prefrontal cortex (DLPFC) for cognitive ToM as opposed to affective ToM processes. METHODS: 1Hz repetitive transcranial magnetic stimulation (rTMS) was used to interfere offline with cortical function of the right DLPFC in healthy male subjects who subsequently had to perform a computerized task assessing cognitive and affective ToM. RESULTS: RTMS over the right DLPFC induced a selective effect on cognitive but not affective ToM. More specifically, a significant acceleration of reaction times in cognitive ToM compared to affective ToM and control items was observed in the experimental (right DLPFC) compared to the control (vertex) rTMS stimulation condition. CONCLUSIONS: Our findings provide evidence for the functional independence of cognitive from affective ToM. Furthermore, they point to an important role of the right DLPFC within neural networks mediating cognitive ToM. Possible underlying mechanisms of the acceleration of cognitive ToM processing under rTMS are discussed.

Date 2010-06
Language eng
Short Title Dissociating cognitive from affective theory of mind
Library Catalog PubMed
Volume 46
Pages 769-780
Publication Cortex; a Journal Devoted to the Study of the Nervous System and Behavior
DOI 10.1016/j.cortex.2009.07.010
Issue 6
Journal Abbr Cortex
ISSN 1973-8102
PMID 19709653
Date Added 11.9.2025, 11:01:32
Modified 11.9.2025, 11:01:32

Tags:

Humans, Neuropsychological Tests, Cognition, Male, Theory of Mind, Magnetic Resonance Imaging, Reaction Time, Affect, Young Adult, Brain, Prefrontal Cortex, Transcranial Magnetic Stimulation

---

Dissociation of decision-making under ambiguity and decision-making under risk in patients with Parkinson's disease: a neuropsychological and psychophysiological study.

Item Type Journal Article

Author Frank Euteneuer
Author Florian Schaefer
Author Ralf Stuermer
Author Wolfram Boucsein
Author Lars Timmermann
Author Michael T. Barbe
Author Georg Ebersbach

Author Jörg Otto
Author Josef Kessler
Author Elke Kalbe
Abstract Decision-making impairments in Parkinson's disease (PD) are most likely associated with dysfunctions in fronto-striatal loops. Recent studies examined decision-making in PD either in ambiguous situations with implicit rules, using the Iowa Gambling Task (IGT), or in risky situations with explicit rules, using the Game of Dice Task (GDT). Both tasks have been associated with the limbic-orbitofrontal-striatal loop, involved in emotional processing. However, the GDT has additionally been highly associated with the dorsolateral prefrontal-striatal loop, being involved in executive functions. The present study is the first one which examined decision-making in PD patients with both, IGT and GDT. We studied 21 non-demented PD patients on dopaminergic medication and 23 healthy controls with both tasks and a neuropsychological test battery with focus on executive functions. To analyse possible abnormalities in emotional processing, electrodermal responses (EDRs) were assessed while performing the tasks. We found that PD patients were significantly impaired in the GDT, but not in the IGT. Executive dysfunctions correlated with GDT but not with IGT performance. In both tasks, PD patients showed significantly reduced feedback EDRs after losses, but not after gains, indicating a primary decline of sensitivity to negative feedback. Our behavioural data suggest that dysfunctions in the dorsolateral prefrontal loop might be stronger than in the limbic loop, resulting in deficits in executive functions and GDT performance but unimpaired IGT performance. Reduced sensitivity to negative feedback is discussed with regard to dysfunctions in the limbic loop, which may result from pathology of limbic structures or dopaminergic medication.
Date 2009 Nov
Language eng
Extra Place: England
Volume 47
Pages 2882-2890
Publication Neuropsychologia
DOI 10.1016/j.neuropsychologia.2009.06.014
Issue 13
Journal Abbr Neuropsychologia
ISSN 1873-3514 0028-3932
PMID 19545579
Date Added 6.7.2025, 19:09:42
Modified 5.9.2025, 14:35:20

Notes:

Included

sample characteristics

size: 21 PD and 23 HC (MATHCED age, gender, and years of school education)

Parkinson's Disease type and duration: idiopathic PD, Mduration = 7.14 (S.D. = 6.06)

Medication: on medication

Hoehn-Yahr: Md=2.5

UPDRS-3: M=17.7 (S.D. = 9.2)

Gender (male): 7 males (33%)

averaged ages (SD, range): M= 67.60 SD=7.31

other neurological disease (tumor, stroke, etc.): None

other major psychopathology: None

origin country (or ethnicity):

method (Review, meta-analysis or observational and/or self-reported):

instruments used in order to quantify the variables

Social cognition aspect: Theory of mind

Name of the task: the "Reading the mind in Eyes" Test (RMET)

type of stimulus [face/voice etc., Ekman faces/other etc.]: to infer mental states from eye stimuli (black-and-white photographs)

operationalization: Accuracy

Main findings related to the review's scope

No sig dif between PD and HC in RMET
Tags: ToM, behavioral

Does a volume reduction of the parietal lobe contribute to freezing of gait in Parkinson's disease?

**Item Type**	Journal Article
**Author**	Alfonso Rubino
**Author**	Francesca Assogna
**Author**	Fabrizio Piras
**Author**	Maria Elena Di Battista

Author Francesca Imperiale
Author Chiara Chiapponi
Author Gianfranco Spalletta
Author Giuseppe Meco

Abstract BACKGROUND: Freezing of gait (FOG) is as a brief, episodic absence or marked reduction of forward progression of the feet despite the intention to walk. Structural neuroimaging studies on FOG in PD using volumetric techniques yielded variable and partially conflicting findings, probably reflecting the heterogeneity and complexity of the phenomenon. The aim of this study was to further explore the differences in local gray matter (GM) volume in patients with PD with and without FOG by using Voxel-Based Morphometry (VBM). MATERIALS AND METHODS: We enrolled 26 patients (7 women and 19 men) with a diagnosis of PD in stable treatment with dopaminergic therapy. Thirteen patients classified as FOG+ were matched with thirteen non-freezer (FOG-) PD patients. All 26 participants underwent a detailed neuropsychological assessment as well as a VBM analysis derived from T1 weighted 3T MRI. RESULTS: The patient groups did not significantly differ for age, disease duration, H&Y stage, UPDRS part-III or educational attainment. No significant differences of cognitive profile emerged. PD-FOG+ patients showed a pattern of relative GM atrophy in left posterior parietal gyrus compared with PD-FOG-. DISCUSSION: Our results suggest that a specific pattern of cortical volume reduction involving posterior parietal cortex contributes to the occurrence of FOG in PD. These data agree with the growing body of evidence considering the parietal posterior cortex as an associative area involved in spatial control of motor behavior, par-taking in response selection to sensory evaluation.

Date 2014 Oct
Language eng
License Copyright © 2014. Published by Elsevier Ltd.
Extra Place: England
Volume 20
Pages 1101-1103
Publication Parkinsonism & related disorders
DOI 10.1016/j.parkreldis.2014.07.002
Issue 10
Journal Abbr Parkinsonism Relat Disord
ISSN 1873-5126 1353-8020
PMID 25112926
Date Added 6.7.2025, 19:09:42
Modified 5.9.2025, 14:54:17

Notes:

Not Included: Does not study SC

Tags: EXCLUDED

Does Facial Amimia Impact the Recognition of Facial Emotions? An EMG Study in Parkinson's Disease.

**Item Type**	Journal Article
**Author**	Soizic Argaud
**Author**	Sylvain Delplanque
**Author**	Jean-François Houvenaghel
**Author**	Manon Auffret
**Author**	Joan Duprez
**Author**	Marc Vérin
**Author**	Didier Grandjean
**Author**	Paul Sauleau
**Abstract**	According to embodied simulation theory, understanding other people's emotions is fostered by facial mimicry. However, studies assessing the effect of facial mimicry on the recognition of emotion are still controversial. In Parkinson's disease (PD), one of the most distinctive clinical features is facial amimia, a reduction in facial expressiveness, but patients also show emotional disturbances. The present study used the pathological model of PD to examine the role of facial mimicry on emotion recognition by investigating EMG responses in PD patients during a facial emotion recognition task (anger, joy, neutral). Our results evidenced a significant decrease in facial mimicry for joy in PD, essentially linked to the absence of reaction of the zygomaticus major and the orbicularis oculi muscles in response to happy avatars, whereas facial mimicry for expressions of anger was relatively preserved. We also confirmed that PD patients were less accurate in recognizing positive and neutral facial expressions and highlighted a beneficial effect of facial mimicry on the recognition of emotion. We thus provide additional arguments for embodied simulation theory suggesting that facial mimicry is a potential lever for therapeutic actions in PD even if it seems not to be necessarily required in recognizing emotion as such.
**Date**	2016
**Language**	eng
**Extra**	Place: United States
**Volume**	11
**Pages**	e0160329
**Publication**	PloS one
**DOI**	10.1371/journal.pone.0160329
**Issue**	7
**Journal Abbr**	PLoS One
**ISSN**	1932-6203
**PMID**	27467393
**PMCID**	PMC4965153
**Date Added**	6.7.2025, 19:09:38
**Modified**	5.9.2025, 14:26:36

Notes:

Included

sample characteristics

size: 40 PD and 40 HC

Parkinson's Disease type and duration: NA, Mduration=9.7 SD=5.3 1-20

Medication: on medication

Hoehn-Yahr: M=1.2 SD=0.7 0-3

UPDRS-3: M=11.9 SD=8.9 1-33

Gender (male): 20 males (50%)

averaged ages (SD, range): M=61.2 SD=9.6 42-79

other neurological disease (tumor, stroke, etc.): None

other major psychopathology: None

origin country (or ethnicity): France

method observational

instruments used in order to quantify the variables

Social cognition aspect: emotion recognition

Name of the task: NA

type of stimulus [face/voice etc., Ekman faces/other etc.]: dynamic avatar appeared on a black screen for 2000 ms. naturally coloured Caucasian avatars (6 women/6 men). For all stimuli, we used FACSGen [34,35] to generate video clips in which the emotional expression unfolded from a neutral state to its emotional peak in 1000 ms. 3D images.

task condition: anger, joy, neutral

operationalization: accuracy and the participants assessed the emotions portrayed and their intensities on seven visual analogue scales (VAS) from 0 to 100%.

Main findings related to the review's scope

Accuracy: PD patients were overall significantly lower than those of the HC

PD patients were significantly lower than those of the HC for happy and neutral avatars but not for angry faces

Confounding emotions: the PD patients provided globally a similar pattern of confusion than the HC.

Surprise was more often selected by the PD patients than the HC for happy avatar and neutral avatar. For neutral avatars, the PD patients also selected more often sadness than the HC did

Tags: Emotion recognition, behavioral

Does STN-DBS really not change emotion recognition in Parkinson's disease?

**Item Type**	Journal Article
**Author**	Julie Péron
**Date**	2014 May
**Language**	eng
**Extra**	Place: England
**Volume**	20
**Pages**	562-563
**Publication**	Parkinsonism & related disorders
**DOI**	10.1016/j.parkreldis.2014.01.018
**Issue**	5
**Journal Abbr**	Parkinsonism Relat Disord
**ISSN**	1873-5126 1353-8020
**PMID**	24571935
**Date Added**	6.7.2025, 19:09:37
**Modified**	5.9.2025, 14:50:08

Notes:

Not Included: Not a study, but a comment on a different article

Tags: EXCLUDED

Dopamine Boosts Memory for Angry Faces in Parkinson's Disease

**Item Type**	Journal Article
**Author**	Leena Subramanian
**Author**	John Vincent Hindle
**Author**	Margaret Cecilia Jackson
**Author**	David E. J. Linden
**Abstract**	The influence of emotional context on cognitive operations is of fundamental importance for the evolution of higher cognitive functions and their disturbance in neuropsychiatric disorders. The dopamine pathways projecting to prefrontal cortex and the basal ganglia are assumed to play a major role in such emotion-cognition interactions. Here we provide evidence for such a role by studying working memory for emotional faces in patients with Parkinson's Disease. We studied 25 patients with Parkinson's disease during their on and off medication states. Faces with emotional expressions (happy, angry, sad, neutral or fearful) were shown and the participants had to remember and later recall the identity of the faces ignoring the expressions. We found that dopaminergic medication enhances working memory for angry faces and suppresses it for sad faces. The results elucidate neurochemical mechanisms for the saliency of threatening information and support cognitive explanations of the

antidepressant effects of dopamine. They also suggest a role for dopamine in changing emotional-cognitive biases rather than as a generic cognitive enhancer. (C) 2010 Movement Disorder Society

Date 2010-12-15
Language English
Extra Place: 111 RIVER ST, HOBOKEN 07030-5774, NJ USA Type: Article
Volume 25
Publisher WILEY
Pages 2792-2799
Publication MOVEMENT DISORDERS
DOI 10.1002/mds.23420
Issue 16
ISSN 0885-3185
Date Added 14.7.2025, 14:50:41
Modified 5.9.2025, 14:57:49

Notes:

Not Included: Only PD group (no comparison). Also not really about SC.

We found that dopaminergic medication enhances working memory for angry faces and suppresses it for sad faces

Tags: EXCLUDED

Dopamine Modulates the Response of the Human Amygdala: A Study in Parkinson's Disease

Item Type Journal Article
Author Alessandro Tessitore
Author Ahmad R. Hariri
Author Francesco Fera
Author William G. Smith
Author Thomas N. Chase
Author Thomas M. Hyde
Author Daniel R. Weinberger
Author Venkata S. Mattay
Date 2002-10-15
Language en
Short Title Dopamine Modulates the Response of the Human Amygdala
Library Catalog DOI.org (Crossref)
URL https://www.jneurosci.org/lookup/doi/10.1523/JNEUROSCI.22-20-09099.2002
Accessed 11.8.2025, 15:27:39
Volume 22
Pages 9099-9103

Publication The Journal of Neuroscience
DOI 10.1523/JNEUROSCI.22-20-09099.2002
Issue 20
Journal Abbr J. Neurosci.
ISSN 0270-6474, 1529-2401

Date Added 11.8.2025, 15:27:39
Modified 11.8.2025, 15:27:39

Notes:

Included

sample characteristics

size: 10 PD and 10 HC matched for age, gender, and education

Parkinson's Disease type and duration: idiopathic, Mduration= NA

Medication: Off and on medication

Hoehn-Yahr: Md=2 1-2

UPDRS-3: NA

Gender (male): 7 males (70%)

averaged ages (SD, range): M=59 SD=NA

other neurological disease (tumor, stroke, etc.): NA

other major psychopathology: Five of them had a history of depression

origin country (or ethnicity): NA

method observational

instruments used in order to quantify the variables

Social cognition aspect: emotion recognition

Name of the task: NA

type of stimulus [face/voice etc., Ekman faces/other etc.]: Ekman faces

task conditions: angry or afraid

operationalization: select one of two facial expressions (either angry or afraid) that matched that of a simultaneously presented target expression >> accuracy and reaction time

fMRI: BOLD fMRI data were collected, while subjects performed the task, on a General Electric 1.5T Signa scanner (Milwau kee, WI) using a gradient echo echoplanar imaging (EPI) sequence covering 24 axial, interleaved slices (4 mm thick, 1 mm gap), beginning at the cerebral vertex and encompassing the entire cerebrum and the majority of the cerebellum (repetition time/echo time, 2000/28 msec; f ield of view, 24 cm; matrix, 64 64).

Image analysis was completed using SPM99 (www.fil. ion.ucl.ac.uk/spm). These realigned images were then spatially normalized into a standard stereotactic space (Montreal Neurological Institute template) using a 12 parameter affine model. Data sets were then selected for their high quality as demonstrated by small motion correc tion (2 mm) and matched voxel variance across sessions (Mattay et al., 1996).

Main findings related to the review's scope

Behavioral results

There was no significant difference for the emotion task across three groups.

BOLD response

In PD patients during the drug-off state, there was no significant amygdala response, but there were significant responses in the posterior fusiform gyri (Fig. 2*b*). However, there was a significant bilateral amygdala response in these same PD patients during the drug-on state (Fig. 2*c*). The responses of sensory and association cortices identified in NCs were also present in PD patients during both the drug-off and drug-on states (Fig.2*b*,*c*).

regions such as the amygdala and posterior fusiform gyrus showed a greater response in the NCs compared with both PD drug states

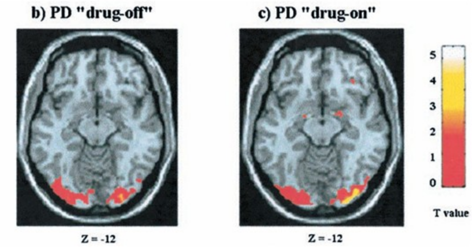

Table 1. Regional BOLD responses to emotion task versus control task in all three groups

Regions	Talairach coordinates (*x*, *y*, *z*)	*Z* score[a]
Normal controls		
Amygdala	−/+14, −8, −12	2.34/2.77
Ventral prefrontal cortex	−/+44, 16, −7	2.00/2.93
Inferior frontal gyrus	−/+37, 6, 28	2.13/2.65
Anterior cingulate cortex	2, 28, 48	2.72
Posterior fusiform gyrus	−/+34, −56, −16	2.30/3.04
Inferior occipital gyrus	−/+42, −82, −8	2.98/3.24
PD drug-off state		
Amygdala	No significant response	
Ventral prefrontal cortex	+42, 33, 18/−50, 29, 18	2.09/2.64
Inferior frontal gyrus	−/+37, 12, 26	2.68/2.00
Anterior cingulate cortex	0, 26, 42	2.05
Posterior fusiform gyrus	−/+34, −50, −16	2.24/2.04
Inferior occipital gyrus	−/+42, −82, −8	2.14/2.43
PD drug-on state		
Amygdala	−16, −6, −12/+14, −6, −12	2.34/3.07
Ventral prefrontal cortex	−48, 16, −5/+40, 23, −5	2.05/2.64
Inferior frontal gyrus	−/+33, 10, 28	2.84/2.23
Anterior cingulate cortex	0, 26, 42	2.60
Posterior fusiform gyrus	−/+36, −54, −12	2.14/2.54
Inferior occipital gyrus	−/+42, −82, −8	2.19/3.05

Coordinates and *Z* scores represent both left (−x) and right (+x) hemisphere responses.

[a] $p < 0.05$ corrected for multiple comparisons across a small volume of interest.

Tags: Emotion recognition, Imaging, behavioral

Dopaminergic modulation of amygdala activity during emotion recognition in patients with Parkinson disease.

Item Type Journal Article
Author Pauline Delaveau
Author Pilar Salgado-Pineda
Author Tatiana Witjas
Author Joëlle Micallef-Roll
Author Eric Fakra
Author Jean-Philippe Azulay
Author Olivier Blin

Abstract Variable findings have been reported for emotional processing in patients with Parkinson disease (PD). These contradictions could be due to differences in the progression of dopamine (DA) depletion. Levodopa treatment could have either beneficial or detrimental effects on brain functions modulated by DA according to disease progression. In healthy subjects, levodopa administration leads to a decreased amygdala activation in response to emotional tasks. Because it is known that there is a link between DA loss in mesolimbic system and depression, we hypothesized that PD patients without depression would have spared limbic DA projections. Consequently, levodopa medication could overdose limbic regions relative to severe dorsal striatal denervation. To evaluate the effect of levodopa on amygdala activation, we conducted a functional magnetic resonance imaging study in nondemented, nondepressed PD patients compared with healthy volunteers. Patients with PD and healthy subjects received either levodopa or placebo in 2 functional magnetic resonance imaging sessions. Amygdala activation was evaluated during a facial emotion recognition task. A similar right-amygdala activity was seen in both healthy subjects and PD patients in the placebo session. After levodopa administration, activity was reduced in both groups. In the patients, the levodopa dose used significantly improved motor dysfunction. Nondemented, nondepressed PD patients thus seem to have relatively preserved DA mesolimbic projections, and consequently, the same dose of levodopa needed to correct the lack of DA in the severely depleted putamen (motor part of striatum) would incidentally overdose the mesolimbic projections toward the amygdala.

Date 2009 Dec
Language eng
Extra Place: United States
Volume 29
Pages 548-554
Publication Journal of clinical psychopharmacology
DOI 10.1097/JCP.0b013e3181bf1c5f
Issue 6
Journal Abbr J Clin Psychopharmacol
ISSN 1533-712X 0271-0749
PMID 19910719
Date Added 6.7.2025, 19:09:42
Modified 5.9.2025, 14:33:06

Notes:

Included

sample characteristics

size: 14 PD and 13 HC

Parkinson's Disease type and duration: NA, Mduration = 11.4 SD=4.4

Medication: off medication and on medication

Hoehn-Yahr: “on” state M=2 SD=0.9

UPDRS-3: placebo, M=22 SD= 6.8; levodopa, M=6.7 SD= 3.1

Gender (male): 11 males (79%)

averaged ages (SD, range): M=61 SD= 8.3 (42-70)

other neurological disease (tumor, stroke, etc.): no dementia

other major psychopathology: None

origin country (or ethnicity): France

method imaging

instruments used in order to quantify the variables

Social cognition aspect: emotion recognition

Name of the task: Emotional Facial Matching Test

type of stimulus [face/voice etc., Ekman faces/other etc.]: target face. color photos were derived from the Karolinska Directed Emotional Faces set.

Condition: fear, anger

operationalization: select which 1 of 2 faces presented on the screen expressed the same emotion (fear or anger). Accuracy

Main findings related to the review's scope

Regarding accuracy (correct responses) on the emotional task, there was no difference between the subject groups (Patients vs Control; whether on placebo or levodopa)

although fMRI measurement was performed, no analysis comparing patients to control in emotion processing on fMRI was conducted.

Tags: emotion recognition, behavioral

Dopaminergic modulation of emotional conflict in Parkinson's disease

Item Type Journal Article
Author Vanessa Fleury

**Author**	Emilie Cousin
**Author**	Virginie Czernecki
**Author**	Emmanuelle Schmitt
**Author**	EugÃ©nie LhommÃ©e
**Author**	Antoine Poncet
**Author**	ValÃ©rie Fraix
**Author**	IrÃ¨ne TroprÃ¨s
**Author**	Pierre Pollak
**Author**	Alexandre Krainik
**Author**	Paul Krack
**Date**	2014-07-23
**Library Catalog**	DOI.org (Crossref)
**URL**	http://journal.frontiersin.org/article/10.3389/fnagi.2014.00164/abstract
**Accessed**	11.8.2025, 15:09:14
**Volume**	6
**Publication**	Frontiers in Aging Neuroscience
**DOI**	10.3389/fnagi.2014.00164
**Journal Abbr**	Front. Aging Neurosci.
**ISSN**	1663-4365
**Date Added**	11.8.2025, 15:09:14
**Modified**	11.8.2025, 15:09:14

Notes:

Included

sample characteristics

size: 10 PD and 12 HC matched age, sex, and education

Parkinson's Disease type and duration: NA, Mduration=9 SD=3.1

Medication: off and on medication

Hoehn-Yahr: NA

UPDRS-3: off M=33.8 SD=10.8 on M=11.4 SD=7.4

Gender (male): 7 males (70%)

averaged ages (SD, range): M=60 SD= 4.2 51–66

other neurological disease (tumor, stroke, etc.): None

other major psychopathology: None

origin country (or ethnicity): France

method observational

instruments used in order to quantify the variables

Social cognition aspect: Emotion recognition

Name of the task: NA, photos from the Montreal Set of Facial Displays of Emotion

type of stimulus [face/voice etc., Ekman faces/other etc.]: black and white happy and fearful facial expression. men and women of European, Asian, Hispanic and African decent. The words "FEAR" or "HAPPY" were prominently written in red bold "Arial" size 45, centered on the middle of faces (i.e., on the "nose" position).

task conditions: The face–word association created either a congruent condition (32 happy faces with the word "HAPPY," 32 fearful faces with the word "FEAR") or an incongruent condition (32 happy faces with the word "FEAR," 32 fearful faces with the word "HAPPY")

operationalization: Participants were instructed to judge as fast and as accurately as possible whether facial expressions represented fear or happiness while ignoring the word. >> accuracy and RT

Name of the task: Ekman Facial Affect Test

type of stimulus [face/voice etc., Ekman faces/other etc.]: Ekman faces

task conditions: happiness, sadness, fear, disgust, surprise, anger, neutral

operationalization: accuracy

fMRI: BOLD fMRI data were collected while subjects performed the ES task in a 3Tesla Bruker

Medspec S300 MRI scanner using a gradient–echo T2*–weighted Echo Planar Imaging (EPI) sequence covering 39 axial, interleaved slices (3.2 mm thick, 0 mm gap), beginning at the cerebral vertex and encompassing the entire cerebrum and the cerebellum (time repetition (TR) = 2500 ms; time echo (TE) = 30 ms; flip angle = 77°; field of view (FOV) = 216 × 216 mm^2; matrix = 72 × 72; voxel size = 3 × 3 × 3.2 mm). Six dummy scans were done before image acquisition to avoid the effects of signal saturation. All scanning parameters were selected to optimize the quality of the BOLD signal while maintaining a sufficient number of slices to acquire whole-brain data. For structural whole-brain images, three-dimensional T1–weighted sagittal images of the whole-brain were also acquired (TR = 2500 ms; TE = 4.3 ms; TI = 908.1 ms; flip angle = 8°; FOV = 256 × 224 × 176 mm^3; voxel size = 1.33 × 1.75 × 1.37 mm^3).

Data analysis was performed by using the general linear model as implemented in SPM8 (Wellcome Department of Imaging Neuroscience, London, UK) where each event is modeled using a hemodynamic function model. Each functional volume was smoothed by an 8-mm Full Width at Half Maximum Gaussian kernel to ameliorate differences in intersubject localization. Time series for each voxel were high-pass filtered (1/128 Hz cutoff) to remove low-frequency noise and signal drift.

Main findings related to the review's scope

No significant differences in the Ekman test (total and subtest).

Stroop task: The mean task accuracy was not statistically different between the three groups (off, on, and HC).

Facial emotions did not influence the percentage of correct responses.

The congruence effect was not statistically different between HC and the drug-on or drug-off patients, whereas it was significantly larger for the drug-on patients compared to the drug-off patients, regardless of the facial emotion.

Brain regions activated during the emotional Stroop task with negative faces in drug-off patients.

Contrast	Cerebral areas	H	BA	MNI coordinates (x, y, z)	k	*T*
[I-C] (negative faces)	**Frontal cortex**					
	Superior medial frontal	L	8	−3, 32, 46	10	5.82
	**Temporal cortex**					
	Inferior temporal	L	37	−51, −55, −8	7	6.02
	Hippocampus	L	28	−24, −37, −8	15	6.28
	**Occipital lobe**					
	Middle occipital	R	19	42, −79, 14	11	8.32
		R	19	30, −85, 20	7	5.09
	Cuneus	R	18	12, −94, 14	8	4.98
[C-I] (negative faces)	*No suprathreshold voxels*					

$p < 0.001$, $k > 5$, $n = 10$, uncorrected. Cerebral areas are defined by the Automatic Anatomical Labeling. BA, Brodmann areas; C, congruent; H, hemisphere; I, incongruent; k, cluster size (number of voxels); L/R, left/right; MNI, Montreal Neurological Institute; x, y, z, mediolateral, rostrocaudal, and dorsoventral.

Brain regions activated during the emotional Stroop task with negative faces in drug-on parkinsonian patients.

Contrast	Cerebral areas	H	BA	MNI coordinates (x, y, z)	k	*T*
[I-C] (negative faces)	**Frontal cortex**					
	Middle frontal	R	46	45, 38, 17	5	5.05
	**Temporal cortex**					
	Middle temporal	L	22	−63, −31, 4	5	4.98
	Inferior temporal	R	19	39, −64, −8	13	7.71
[C-I] (negative faces)	*No suprathreshold voxels*					

$p < 0.001$, $k > 5$, $n = 10$, uncorrected. Cerebral areas are defined by the Automatic Anatomical Labeling. BA, Brodmann areas; C, congruent; H, hemisphere; I, incongruent; k, cluster size (number of voxels); L/R, left/right; MNI, Montreal Neurological Institute; x, y, z, mediolateral, rostrocaudal, and dorsoventral.

fMRI Between-Group Comparisons

For the contrast [I-C] with negative faces, HC displayed significantly greater activation than off-drug patients within the right rACC (x, y, z: 6, 47, 4.4; *BA* = 32; $p < 0.001$; $k > 5$ uncorrected) (Figure 6), the right pre- and post-central gyri and the right thalamus (Table 8A). Drug-off patients vs. HC did not yield any suprathreshold clusters (Table 8B).

Comparisons between HC and drug-on patients (HC vs. drug-on patients and drug-on patients vs. HC) did not yield any suprathreshold clusters. Drug-on patients had significantly greater activation than drug-off patients in the right inferior temporal gyrus (x, y, z: 42, −61, −8; *BA* = 37; $p < 0.001$; $k > 5$ uncorrected), an area involved in visual recognition. No significant activation was found in the ACC for a $p < 0.001$. However, by reducing the threshold to $p < 0.005$, drug-on patients displayed greater activation than drug-off patients in the left postgenual ACC (x, y, z: −3, 23, 19; *BA* = 24; $T = 4.28$; $p < 0.005$; $k > 5$ uncorrected). For a $p < 0.008$, drug-on patients displayed greater activation than drug-off patients in the right rACC (x, y, z: 9, 38, 1; *BA* = 32; $p < 0.008$; $k > 5$ uncorrected) (Table 9). Drug-off patients did not exhibit any suprathreshold clusters when compared with drug-on patients at a $p < 0.001$ threshold. No activation was seen in the ACC when the threshold was increased to $p < 0.008$ (Table 9). No suprathreshold voxels were activated for [C-I] contrast for negative faces in any between-group comparisons.

Tags: Emotion recognition, Imaging, behavioral

Dopaminergic modulation of the default mode network in Parkinson's disease

**Item Type**	Journal Article
**Author**	Pauline Delaveau
**Author**	Pilar Salgado-Pineda
**Author**	Philippe Fossati
**Author**	Tatiana Witjas
**Author**	Jean-Philippe Azulay
**Author**	Olivier Blin
**Date**	11/2010
**Language**	en
**Library Catalog**	Crossref
**URL**	https://linkinghub.elsevier.com/retrieve/pii/S0924977X10001471
**Accessed**	13.7.2025, 20:22:55
**License**	https://www.elsevier.com/tdm/userlicense/1.0/
**Volume**	20
**Publisher**	Elsevier BV
**Pages**	784-792
**Publication**	European Neuropsychopharmacology
**DOI**	10.1016/j.euroneuro.2010.07.001
**Issue**	11
**ISSN**	0924-977X
**Date Added**	13.7.2025, 20:22:55
**Modified**	5.9.2025, 14:32:53

Notes:

Not Included: no relevant results

??

To evaluate the effect of levodopa on DMN deactivation, we conducted a randomized, crossover, placebo-controlled experiment consisting of two fMRI assessments in fourteen non-demented, non-depressed PD patients compared to thirteen healthy volunteers. They received either acute doses of levodopa or placebo in two fMRI sessions. Brain deactivation was evaluated during a facial emotion recognition task.

While the control subjects showed a classical brain deactivation pattern during the emotional task, the PD patients taking placebo only deactivated the ventral medial prefrontal cortex. Patients failed to deactivate the posterior midline and lateral parts of DMN network. After levodopa administration, this network was restored conjointly with the improvement of motor dysfunction in PD patients.

The levodopa effect on DMN is probably the consequence of a beneficial dopamine (DA) medication effect which leads to a fine tuning of the dopamine level in the motor part of striatum, resulting to a global improvement of physical state of PD patients and consequently an increased attentional resource to external stimuli. The absence of medial prefrontal deactivation impairment may suggest a preserved mesocortical DA system in these patients.

Tags: EXCLUDED

Dynamic emotion processing in Parkinson's disease as a function of channel availability.

**Item Type**	Journal Article
**Author**	Silke Paulmann
**Author**	Marc D. Pell
**Abstract**	Parkinson's disease (PD) is linked to impairments for recognizing emotional expressions, although the extent and nature of these communication deficits are uncertain. Here, we compared how adults with and without PD recognize dynamic expressions of emotion in three channels, involving lexical-semantic, prosody, and/or facial cues (each channel was investigated individually and in combination). Results indicated that while emotion recognition increased with channel availability in the PD group, patients performed significantly worse than healthy participants in all conditions. Difficulties processing dynamic emotional stimuli in PD could be linked to striatal dysfunction, which reduces efficient binding of sequential information in the disease.
**Date**	2010 Oct
**Language**	eng
**Extra**	Place: England
**Volume**	32
**Pages**	822-835
**Publication**	Journal of clinical and experimental neuropsychology
**DOI**	10.1080/13803391003596371
**Issue**	8
**Journal Abbr**	J Clin Exp Neuropsychol
**ISSN**	1744-411X 1380-3395

PMID 20336567
Date Added 6.7.2025, 19:09:39
Modified 5.9.2025, 14:49:22

Notes:

Included

Sample characteristics

Size: 11 PD, 11 HC

PD-type: Idiopathic PD

PD-duration: M = 10.3 years SD = 3.2, range = 4–15

Medication: on state

Hoehn-Yahr: Range 2-4, Mode = 3

UPDRS-3: M = 28.5, SD = 9.9

Gender (male): 6 (55%)

Age: M = 68.0, SD = 10.8

Other neurological disease (tumor, stroke, etc.): None

Other major psychopathology: None

Origin country (or ethnicity): Canada

method behavioral

instruments used in order to quantify the variables

Social cognition aspect: emotion recognition

Name of the task: NA

Type of stimulus [face/voice etc., Ekman faces/other etc.]: base stimulus materials were short sentences produced by six English speakers. To allow prosodic information to be isolated in certain conditions, there were two distinct sentence types: "lexical," or well-formed English sentences with emotional semantic content (e.g., I didn't make the team to convey sadness); and pseudosentences, which were semantically anomalous (e.g, Someone migged the pazing). Five unique lexical sentences were constructed to convey each emotion type (5 items × 6 emotion types = 30 total), whereas the same five pseudosentences could be emotionally inflected by speakers to convey the six emotion types strictly through prosody

Task condition: five basic emotions (anger, disgust, sad, happiness, pleasant surprise) and neutral affect

There were three unimodal conditions where only one communication channel was available (face, prosody, semantics), three bimodal conditions where two channels were available (face + prosody, face + semantics, prosody + semantics), and one multimodal condition, which presented cues in all three information channels (face + prosody + semantics)

Operationalization: Correct answers

Main findings related to the review's scope

A 7 (channel) × 6 (emotion) × 2 (group) ANOVA performed. the ANOVA on these data revealed a significant main effect of group, $F(1, 20) = 7.42$, $p = .01$, $r = .52$. When viewed overall, recognition of

emotions was more accurate for the healthy control participants than for the PD patients (79% vs. 65%). There was no evidence that group interacted with either emotion or channel in the form of significant two- or three-way interactions (all Fs < 1.22, ps > .30)

Tags: Emotion Recognition, behavioral

Early Sociability and Social Memory Impairment in the A53T Mouse Model of Parkinson's Disease Are Ameliorated by Chemogenetic Modulation of Orexin Neuron Activity

**Item Type**	Journal Article
**Author**	Milos Stanojlovic
**Author**	Jean Pierre Pallais, Jr. Yllescas
**Author**	Aarthi Vijayakumar
**Author**	Catherine Kotz
**Abstract**	Parkinson's disease (PD) is a multi-layered progressive neurodegenerative disease. Signature motor system impairments are accompanied by a variety of other symptoms such as mood, sleep, metabolic, and cognitive disorders. Interestingly, social cognition impairments can be observed from the earliest stages of the disease, prior to the onset of the motor symptoms. In this study, we investigated age-related reductions in sociability and social memory in the A53T mouse model of PD. Since inflammation and astrogliosis are an integral part of PD pathology and impair proper neuronal function, we examined astrogliosis and inflammation markers and parvalbumin expression in medial pre-frontal cortex (mPFC), part of the brain responsible for social cognition regulation. Finally, we used DREADDs (Designer Receptors Exclusively Activated by Designer Drugs) for the stimulation and inhibition of orexin neuronal activity to modulate sociability and social memory in A53T mice. We observed that social cognition impairment in A53T mice is accompanied by an increase in astrogliosis and inflammation markers, in addition to loss of parvalbumin neurons and inhibitory pre-synaptic terminals in the mPFC. Moreover, DREADD-induced activation of orexin neurons restores social cognition in the A53T mouse model of PD.
**Date**	2019-12
**Language**	English
**Extra**	Place: ONE NEW YORK PLAZA, SUITE 4600, NEW YORK, NY, UNITED STATES Type: Article
**Volume**	56
**Publisher**	SPRINGER
**Pages**	8435-8450
**Publication**	MOLECULAR NEUROBIOLOGY
**DOI**	10.1007/s12035-019-01682-x
**Issue**	12
**ISSN**	0893-7648
**Date Added**	14.7.2025, 14:50:32
**Modified**	5.9.2025, 14:57:33

Notes:

Not Included: Study in mice
Tags: EXCLUDED

EEG-based emotion charting for Parkinson's disease patients using Convolutional Recurrent Neural Networks and cross dataset learning.

Item Type Journal Article
Author Muhammad Najam Dar
Author Muhammad Usman Akram
Author Rajamanickam Yuvaraj
Author Sajid Gul Khawaja
Author M. Murugappan
Abstract Electroencephalogram (EEG) based emotion classification reflects the actual and intrinsic emotional state, resulting in more reliable, natural, and meaningful human-computer interaction with applications in entertainment consumption behavior, interactive brain-computer interface, and monitoring of psychological health of patients in the domain of e-healthcare. Challenges of EEG-based emotion recognition in real-world applications are variations among experimental settings and cognitive health conditions. Parkinson's Disease (PD) is the second most common neurodegenerative disorder, resulting in impaired recognition and expression of emotions. The deficit of emotional expression poses challenges for the healthcare services provided to PD patients. This study proposes 1D-CRNN-ELM architecture, which combines one-dimensional Convolutional Recurrent Neural Network (1D-CRNN) with an Extreme Learning Machine (ELM), robust for the emotion detection of PD patients, also available for cross dataset learning with various emotions and experimental settings. In the proposed framework, after EEG preprocessing, the trained CRNN can use as a feature extractor with ELM as the classifier, and again this trained CRNN can be used for learning of new emotions set with fine-tuning of other datasets. This paper also applied cross dataset learning of emotions by training with PD patients datasets and fine-tuning with publicly available datasets of AMIGOS and SEED-IV, and vice versa. Random splitting of train and test data with 80 - 20 ratio resulted in an accuracy of 97.75% for AMIGOS, 83.20% for PD, and 86.00% for HC with six basic emotion classes. Fine-tuning of trained architecture with four emotions of the SEED-IV dataset results in 92.5% accuracy. To validate the generalization of our results, leave one subject (patient) out cross-validation is also incorporated with mean accuracies of 95.84% for AMIGOS, 75.09% for PD, 77.85% for HC, and 84.97% for SEED-IV is achieved. Only a 1 - sec segment of EEG signal from 14 channels is enough to detect emotions with this performance. The proposed method outperforms state-of-the-art studies to classify EEG-based emotions with publicly available datasets, provide cross dataset learning, and validate the robustness of the deep learning framework for real-world application of psychological healthcare monitoring of Parkinson's disease patients.
Date 2022 May
Language eng
License Copyright © 2022. Published by Elsevier Ltd.
Extra Place: United States
Volume 144
Pages 105327

Publication Computers in biology and medicine
DOI 10.1016/j.compbiomed.2022.105327
Journal Abbr Comput Biol Med
ISSN 1879-0534 0010-4825
PMID 35303579
Date Added 6.7.2025, 19:09:36
Modified 5.9.2025, 14:32:25

Notes:

Not Included: EEG study
Tags: EXCLUDED

Effect of Dopamine Therapy on Nonverbal Affect Burst Recognition in Parkinson's Disease

Item Type Journal Article
Author Julie Peron
Author Didier Grandjean
Author Sophie Drapier
Author Marc Verin
Abstract Background: Parkinson's disease (PD) provides a model for investigating the involvement of the basal ganglia and mesolimbic dopaminergic system in the recognition of emotions from voices (i.e., emotional prosody). Although previous studies of emotional prosody recognition in PD have reported evidence of impairment, none of them compared PD patients at different stages of the disease, or ON and OFF dopamine replacement therapy, making it difficult to determine whether their impairment was due to general cognitive deterioration or to a more specific dopaminergic deficit. Methods: We explored the involvement of the dopaminergic pathways in the recognition of nonverbal affect bursts (onomatopoeias) in 15 newly diagnosed PD patients in the early stages of the disease, 15 PD patients in the advanced stages of the disease and 15 healthy controls. The early PD group was studied in two conditions: ON and OFF dopaminergic therapy. Results: Results showed that the early PD patients performed more poorly in the ON condition than in the OFF one, for overall emotion recognition, as well as for the recognition of anger, disgust and fear. Additionally, for anger, the early PD ON patients performed more poorly than controls. For overall emotion recognition, both advanced PD patients and early PD ON patients performed more poorly than controls. Analysis of continuous ratings on target and nontarget visual analog scales confirmed these patterns of results, showing a systematic emotional bias in both the advanced PD and early PD ON (but not OFF) patients compared with controls. Conclusions: These results i) confirm the involvement of the dopaminergic pathways and basal ganglia in emotional prosody recognition, and ii) suggest a possibly deleterious effect of dopatherapy on affective abilities in the early stages of PD.
Date 2014-03-20
Language English

**Extra**	Place: 1160 BATTERY STREET, STE 100, SAN FRANCISCO, CA 94111 USA<br>Type: Article
**Volume**	9
**Publisher**	PUBLIC LIBRARY SCIENCE
**Publication**	PLOS ONE
**DOI**	10.1371/journal.pone.0090092
**Issue**	3
**ISSN**	1932-6203
**Date Added**	14.7.2025, 14:50:38
**Modified**	14.7.2025, 14:50:38

Notes:

Included

Sample characteristics

Size: 15 EPD (early PD), 15 APD (advanced PD), 15 HC) (matched for age and education)

PD-type: Idiopathic PD

PD-duration: EPD: M = 2.8, SD = 1.2; APD: M = 11.1, Sd = 3.4

Medication: EPD tested ON and OFF, APD always medicated

Hoehn-Yahr: EPD: 0.6, SD = 0.7 (ON), APD: M = 1.3, SD = 0.8

UPDRS-3: NA

Gender (male): 10 (66%) for each group

Age: EPD: M = 60.3, SD = 7.3, APD: M = 59.5, SD = 8.6

Other neurological disease (tumor, stroke, etc.): None

Other major psychopathology: None

Origin country (or ethnicity): France

method behavioural

instruments used in order to quantify the variables

Social cognition aspect: Vocal emotion recognition

Name of the task: NA

Type of stimulus [face/voice etc., Ekman faces/other etc.]: stimuli were taken from the Montreal Affective Voices (MAV) database. a total of 70 vocal stimuli

Task condition: seven different prosodies (anger, fear, happiness, neutral, disgust, surprise, and sadness)

Operationalization: Ratings an visual analogue scale for each emotion. A response was deemed to be correct when the subject rated the “Target” scale (e.g. the “Anger” scale when the stimulus was “Anger”) higher than all the other scales

Main findings related to the review's scope

overall recognition score, both the advanced PD and early PD ON patients performed more poorly than the HC

whereas there was no significant difference between the advanced PD and early PD ON patients

for the anger recognition subscore, impairment seemed to be restricted to the early PD ON group, with pairwise comparisons revealing that the early PD ON patients performed more poorly than the HC.

there were no Group×Scale interaction effects for
the happiness, sadness, surprise, disgust and neutral prosodies ($F<1$ for all comparisons). There were, however, significant interactions for anger, $F(12, 252)=3.10$, $p<.001$, and fear, $F(12, 252)=2.38$, $p<.01$.

No sig. diff for EPD OFF vs HC
Tags: emotion recognition, behavioral

Effect of Parkinson Disease on Emotion Perception Using the Persian Affective Voices Test

**Item Type**	Journal Article
**Author**	Arezoo Saffarian
**Author**	Yunes Amiri Shavaki
**Author**	Gholam Ali Shahidi
**Author**	Zahra Jafari
**Abstract**	Background and Objectives: Emotion perception plays a major role in proper communication with people in different social interactions. Nonverbal affect bursts can be used to evaluate vocal emotion perception. The present study was a preliminary step to establishing the psychometric properties of the Persian version of the Montreal Affective Voices (MAV) test, as well as to investigate the effect of Parkinson disease (PD) on vocal emotion perception. Methods: The short, emotional sound made by pronouncing the vowel "a" in Persian was recorded by 22 actors and actresses to develop the Persian version of the MAV, the Persian Affective Voices (PAV), for emotions of happiness, sadness, pleasure, pain, anger, disgust, fear, surprise, and neutrality. The results of the recordings of five of the actresses and five of the actors who obtained the highest score were used to generate the test. For convergent validity assessment, the correlation between the PAV and a speech prosody comprehension test was examined using a gender- and age-matched control group. To investigate the effect of the PD on emotion perception, the PAV test was performed on 28 patients with mild PD between ages 50 and 70 years. Results: The PAV showed a high internal consistency (Cronbach's alpha = 0.80). A significant positive correlation was observed between the PAV and the speech prosody comprehension test. The test-retest reliability also showed the high repeatability of the PAV (intraclass correlation coefficient = 0.815, P <= 0.001). A significant difference was observed between the patients with PD and the controls in all subtests. Conclusion: The PAV test is a useful psychometric tool for examining vocal emotion perception that can be used in both behavioral and neuroimaging studies.
**Date**	2019-07
**Language**	English
**Extra**	Place: 360 PARK AVENUE SOUTH, NEW YORK, NY 10010-1710 USA Type: Article
**Volume**	33
**Publisher**	MOSBY-ELSEVIER
**Publication**	JOURNAL OF VOICE
**DOI**	10.1016/j.jvoice.2018.01.013
**Issue**	4
**ISSN**	0892-1997
**Date Added**	14.7.2025, 14:50:33
**Modified**	5.9.2025, 14:54:44

Notes:

Included

Sample characteristics

Size: 28 PD, 28 HC (matched for age, gender, education)

PD-type: Idiopathic PD

PD-duration: M = 4.67, SD = 1.24

Medication: ON state

Hoehn-Yahr: Range = 1-2

UPDRS-3: NA

Gender (male): 16 (57%)

Age: M = 56.83, Sd = 4.23

Other neurological disease (tumor, stroke, etc.): None

Other major psychopathology: None

Origin country (or ethnicity): Persia as mother tongue

method (Review, meta-analysis or observational and/or self-reported):

instruments used in order to quantify the variables

Social cognition aspect: Vocal emotion perception

Name of the task: Persian Affective Voices (PAV)

Type of stimulus [face/voice etc., Ekman faces/other etc.]: Short emotional sounds using the Persion vowel "a".

Task condition: happiness, sadness, anger, pain, pleasure, disgust, fear, surprise, neutral emotions

Operationalization: The patients were requested to evaluate all the 90 voices (10 actors and actresses × 9 categories of emotion) based on a 10-point rating scale (e.g. (from "not at all angry" to "extremely angry,"). The total scores of the PAV test were given based on the 10-point rating scale of intensity. >> Correct answers in percent

Main findings related to the review's scope

People with PD score significantly lower on the PAV compared with subjects without PD.
Tags: emotion recognition, behavioral

Effectiveness of telerehabilitation plus virtual reality (Tele-RV) in cognitive e social functioning: A randomized clinical study on Parkinson's disease.

Item Type Journal Article

Author Maria Grazia Maggio
Author Antonina Luca
Author Calogero Edoardo Cicero
Author Rocco Salvatore Calabrò
Author Filippo Drago
Author Mario Zappia
Author Alessandra Nicoletti
Abstract INTRODUCTION: Telemedicine could represent an emerging and innovative approach to support cognitive and behavioral rehabilitation reducing the overload of healthcare facilities, favoring home care therapy. The present study aimed to assess the potential efficacy of Tele-VR apps in enhancing cognitive performance and improving social skills in patients with Parkinson's disease (PD). METHODS: Thirty-four patients with PD were included in the study. Patients were assigned to one of the following treatment groups: Experimental Group 1 (EG1) underwent a Tele-VR program using two cognitive rehabilitation applications (app) on smartphones (Neuronation-Brain Training and Train your Brain); Experimental Group 2 (EG2) received a Tele-VR program through one cognitive rehabilitation app (Neuronation-Brain Training) and one socio-cognitive rehabilitation App (The Sims) on smartphones; Active Control Group (aCG) performed a conventional training using pencil and paper exercises (Not-VR). RESULTS: At the end of the study, the aCG and EG1 presented an improvement in the executive, attentional and visuospatial cognitive domains. Mood and subjective memory also improved in the EG1. Moreover, in the EG2 group, a significant improvement was found in all cognitive domains, including social cognition skills (theory of mind). The inter-group comparison showed that both EG1 and EG2 had significantly greater improvements than aCG in MoCA score. Finally, both EG1 and EG2 showed a higher improvement in the FAB score, as compared to the aCG. CONCLUSION: Rehabilitation with smartphone apps could be more useful than conventional rehabilitation in improving cognitive and social cognition skills in patients with PD. Combining cognitive and social cognition training could improve the cognitive and affective domains, also aiding in the long-term maintenance of cognitive outcomes.
Date 2024 Feb
Language eng
License Copyright © 2023 Elsevier Ltd. All rights reserved.
Extra Place: England
Volume 119
Pages 105970
Publication Parkinsonism & related disorders
DOI 10.1016/j.parkreldis.2023.105970
Journal Abbr Parkinsonism Relat Disord
ISSN 1873-5126 1353-8020
PMID 38142630
Date Added 6.7.2025, 19:09:41
Modified 5.9.2025, 14:44:47

Notes:

Not Included: Training with only PD group

Tags: EXCLUDED

Effects of facial biofeedback on hypomimia, emotion recognition, and affect in Parkinson's disease.

Item Type Journal Article
Author Sarah Roßkopf
Author Theresa Friederike Wechsler
Author Stefanie Tucha
Author Andreas Mühlberger
Abstract OBJECTIVES: Facial expressions are a core component of emotions and nonverbal social communication. Therefore, hypomimia as secondary symptom of Parkinson's disease (PD) has adverse effects like social impairment, stigmatization, under-diagnosis and under-treatment of depression, and a generally lower quality of life. Beside unspecific dopaminergic treatment, specific treatment options for hypomimia in PD are rarely investigated. This quasi-randomized controlled trial evaluated the short-term effects of facial electromyogram (EMG) based biofeedback to enhance facial expression and emotion recognition as nonverbal social communication skills in PD patients. Furthermore effects on affect are examined. METHOD: A sample of 34 in-patients with PD were allocated either to facial EMG-biofeedback as experimental group or non-facial exercises as control group. Facial expression during posing of emotions (measured via EMG), facial emotion recognition, and positive and negative affect were assessed before and after treatment. Stronger improvements were expected in the EMG-biofeedback in comparison to the control group. RESULTS: The facial EMG-biofeedback group showed significantly greater improvements in overall facial expression, and especially for happiness and disgust. Also, overall facial emotion recognition abilities improved significantly stronger in the experimental group. Positive affect was significantly increased in both groups with no significant differences between them, while negative affect did not change within both groups. CONCLUSIONS: The study provides promising evidence for facial EMG-biofeedback as a tool to improve facial expression and emotion recognition in PD. Embodiment theories are discussed as working mechanism.
Date 2024 May
Language eng
Extra Place: England
Volume 30
Pages 360-369
Publication Journal of the International Neuropsychological Society : JINS
DOI 10.1017/S1355617723000747
Issue 4
Journal Abbr J Int Neuropsychol Soc
ISSN 1469-7661 1355-6177
PMID 38017615
Date Added 6.7.2025, 19:09:35
Modified 5.9.2025, 14:54:08

Notes:

Not Included: No comp. group
Tags: EXCLUDED

Effects of happy and sad facial expressions on the perception of time in Parkinson's disease patients with mild cognitive impairment

**Item Type**	Journal Article
**Author**	Giovanna Mioni
**Author**	Simon Grondin
**Author**	Lucia Meligrana
**Author**	Francesco Perini
**Author**	Luigi Bartolomei
**Author**	Franca Stablum
**Abstract**	Introduction: Parkinson's disease (PD) is a movement disorder caused by deterioration of the dopaminergic system. Previous studies have demonstrated temporal as well as emotional facial recognition impairment in PD patients. Moreover, it has been demonstrated that emotional facial expressions alter temporal judgments. In the present study, we investigate the magnitude of temporal distortions caused by the presentation of emotional facial expressions (happiness, sadness, and neutral) in PD patients with mild cognitive impairment (PD-MCI) and controls.Method: Seventeen older adults with PD-MCI and 22 healthy older adults took part in the present study. Participants were tested with a time bisection task with standard intervals lasting 400 ms and 1600 ms. Moreover, a complete neuropsychological evaluation was conducted to characterize the sample.Results: Differences between groups were observed indicating a general underestimation of time in PD-MCI patients. Temporal impairments in PD-MCI patients seem to be caused mainly by a dysfunction at the level of reference memory. The effect of emotional facial expressions on time perception was evident in both PD patients and controls, with an overestimation of perceived duration when happiness was presented and an underestimation when sadness was presented.Conclusion: Overall, our results indicate that reduced cognitive abilities might be responsible for the lower temporal ability observed in PD-MCI patients. Moreover, similar effects of emotional stimuli were observed in both PD-MCI patients and controls.
**Date**	2018
**Language**	English
**Extra**	Place: 530 WALNUT STREET, STE 850, PHILADELPHIA, PA 19106 USA Type: Article
**Volume**	40
**Publisher**	TAYLOR & FRANCIS INC
**Pages**	123-138
**Publication**	JOURNAL OF CLINICAL AND EXPERIMENTAL NEUROPSYCHOLOGY
**DOI**	10.1080/13803395.2017.1324021
**Issue**	2
**ISSN**	1380-3395
**Date Added**	14.7.2025, 14:50:34
**Modified**	5.9.2025, 14:47:03

Notes:

Not Included: Does not study SC

Tags: EXCLUDED

Effects of Subthalamic Nucleus Deep Brain Stimulation on Facial Emotion Recognition in Parkinson's Disease: A Critical Literature Review.

**Item Type**	Journal Article
**Author**	S. Kalampokini
**Author**	E. Lyros
**Author**	P. Lochner
**Author**	K. Fassbender
**Author**	M. M. Unger
**Abstract**	Deep brain stimulation (DBS) of the subthalamic nucleus (STN) is an effective therapy for Parkinson's disease (PD). Nevertheless, DBS has been associated with certain nonmotor, neuropsychiatric effects such as worsening of emotion recognition from facial expressions. In order to investigate facial emotion recognition (FER) after STN DBS, we conducted a literature search of the electronic databases MEDLINE and Web of science. In this review, we analyze studies assessing FER after STN DBS in PD patients and summarize the current knowledge of the effects of STN DBS on FER. The majority of studies, which had clinical and methodological heterogeneity, showed that FER is worsening after STN DBS in PD patients, particularly for negative emotions (sadness, fear, anger, and tendency for disgust). FER worsening after STN DBS can be attributed to the functional role of the STN in limbic circuits and the interference of STN stimulation with neural networks involved in FER, including the connections of the STN with the limbic part of the basal ganglia and pre- and frontal areas. These outcomes improve our understanding of the role of the STN in the integration of motor, cognitive, and emotional aspects of behaviour in the growing field of affective neuroscience. Further studies using standardized neuropsychological measures of FER assessment and including larger cohorts are needed, in order to draw definite conclusions about the effect of STN DBS on emotional recognition and its impact on patients' quality of life.
**Date**	2020
**Language**	eng
**License**	Copyright © 2020 S. Kalampokini et al.
**Extra**	Place: Netherlands
**Volume**	2020
**Pages**	4329297
**Publication**	Behavioural neurology
**DOI**	10.1155/2020/4329297
**Journal Abbr**	Behav Neurol
**ISSN**	1875-8584 0953-4180
**PMID**	32724481
**PMCID**	PMC7382738
**Date Added**	6.7.2025, 19:09:36

Modified 5.9.2025, 14:40:50

Notes:

__Not Included__: Not a systematic review

Tags: EXCLUDED

Eliciting clinical empathy via transmission of patient-specific symptoms of Parkinson's disease

Item Type Journal Article

Author Adam Palanica
Author Anirudh Thommandram
Author Yan Fossat

Abstract Clinical empathy can have numerous benefits for patients, clinicians, and health-care providers. Traditional empathy training techniques (e.g. storytelling, videos, or disease simulators) are centered on the health condition rather than the individual. This condition-centric approach perpetuates the belief that the disease, rather than the patient, is at the core of the experience. This process can be ineffective in generating the ability to understand and accurately acknowledge the feelings of another. A more effective means of eliciting empathy can be through technology-mediated symptom transference for transmitting an individual patient's actual experience, rather than a simulation, to the usera process termed tele-empathy. We developed an investigational digital tele-empathy device for use toward patients with Parkinson's disease (PD), known as SymPulse. The device plays back muscle tremors using an armband, giving the wearer a replication of the involuntary muscle activity that a patient with PD feels. The purpose of the current study was to determine whether the SymPulse device could enhance feelings of empathy in test participants (wearing the device) versus control participants (not wearing the device). A sample of 45 participants (22 test; 23 control) reported their level of empathy via self-report questionnaires. Results revealed significantly higher empathy scale scores for test compared to control participants, demonstrating the effectiveness of the SymPulse for use in tele-empathy. The use of such technology for eliciting tele-empathy may have practical and clinical implications for providing effective training to health-care providers.

Date 2018-09-25
Language English
Extra Place: KARL JOHANS GATE 5, NO-0154 OSLO, NORWAY Type: Article
Volume 5
Publisher TAYLOR & FRANCIS AS
Pages 1-13
Publication COGENT PSYCHOLOGY
DOI 10.1080/23311908.2018.1526459
Issue 1
ISSN 2331-1908
Date Added 14.7.2025, 14:50:34
Modified 5.9.2025, 14:48:35

Notes:

Not Included: no comp. group.
Tags: EXCLUDED

Embodied cognition and emotion, two variables improving memory abilities in Parkinson's and Alzheimer's diseases.

**Item Type**	Journal Article
**Author**	Romane Croze
**Author**	Domitille Dilly
**Author**	Marielle Godeau
**Author**	Zineb Bouazza
**Author**	Jean-Claude Getenet
**Author**	Hanna Chainay
**Author**	Céline Borg
**Abstract**	OBJECTIVE: This study investigated whether emotions and enactment can jointly increase memory performance in nondemented Parkinson's disease (PD) and Alzheimer's disease (AD) patients. METHOD: Actions' drawings with negative, positive, or neutral valence were presented to 17 PD patients, 17 AD patients, and 37 elderly controls, matched to age. Two conditions of intentional encoding were proposed to each participant: one verbal, in which participants had to name the represented actions; and one motor, in which they had to mime the displayed actions. Thereafter, participants were submitted to an immediate free recall task and a delayed recognition task. RESULTS: The enactment effect was found in all three groups. The effect of emotion was also observed in that all three groups recalled negative actions better than both neutral and positive (PD patients), only neutral (AD patients), or only positive actions (elderly controls). Positive actions were not recalled better than neutral actions in any group. CONCLUSIONS: These results constitute an evidence for the preservation of the enactment effect and of the emotion effect on memory in AD and PD patients. However, they do not support the hypothesis of the combined effect of emotion and enactment on memory, neither in AD and PD patients nor in normal aging. (PsycInfo Database Record (c) 2022 APA, all rights reserved).
**Date**	2022 Oct
**Language**	eng
**Extra**	Place: United States
**Volume**	36
**Pages**	614-625
**Publication**	Neuropsychology
**DOI**	10.1037/neu0000826
**Issue**	7
**Journal Abbr**	Neuropsychology
**ISSN**	1931-1559 0894-4105
**PMID**	35786959
**Date Added**	6.7.2025, 19:09:35
**Modified**	5.9.2025, 14:32:02

Notes:

Not Included: not on SC
Tags: EXCLUDED

Emotion and basal ganglia (I): What can we learn from Parkinsons disease?

**Item Type**	Journal Article
**Author**	T. Dondaine
**Author**	J. Peron
**Abstract**	Parkinson's disease provides a useful model for studying the neural substrates of emotional processing. The striato-thalamo-cortical circuits, like the mesolimbic dopamine system that modulates their function, are thought to be involved in emotional processing. As Parkinson's disease is histopathologically characterized by the selective, progressive and chronic degeneration of the nigrostriatal and mesocorticolimbic dopamine systems, it can therefore serve as a model for assessing the functional role of these circuits in humans. In the present review, after a definition of emotional processing from a multicomponential perspective, a synopsis of the emotional disturbances observed in Parkinson's disease is proposed. Note that the studies on the affective consequences of subthalamic nucleus deep brain stimulation in Parkinson's disease were excluded from this review because the subject of a companion paper in this issue. This review leads to the conclusion that several emotional components would be disrupted in Parkinson's disease: subjective feeling, neurophysiological activation, and motor expression. We then discuss the functional roles of the striato-thalamo-cortical and mesolimbic circuits, ending with the conclusion that both these pathways are indeed involved in emotional processing. (C) 2012 Elsevier Masson SAS. All rights reserved.
**Date**	2012-09
**Language**	French
**Extra**	Place: 21 STREET CAMILLE DESMOULINS, ISSY, 92789 MOULINEAUX CEDEX 9, FRANCE Type: Article
**Volume**	168
**Publisher**	MASSON EDITEUR
**Pages**	634-641
**Publication**	REVUE NEUROLOGIQUE
**DOI**	10.1016/j.neurol.2012.06.013
**Issue**	8-9
**ISSN**	0035-3787
**Date Added**	14.7.2025, 14:50:39
**Modified**	5.9.2025, 14:34:07

Notes:

Not Included: not in English
Tags: EXCLUDED

Emotion and basal ganglia (II): What can we learn from subthalamic nucleus deep brain stimulation in Parkinson's disease?

Item Type Journal Article
Author J. Peron
Author T. Dondaine
Abstract The subthalamic nucleus deep-brain stimulation Parkinson's disease patient model seems to represent a unique opportunity for studying the functional role of the basal ganglia and notably the subthalamic nucleus in human emotional processing. Indeed, in addition to constituting a therapeutic advance for severely disabled Parkinson's disease patients, deep brain stimulation is a technique, which selectively modulates the activity of focal structures targeted by surgery. There is growing evidence of a link between emotional impairments and deep-brain stimulation of the subthalamic nucleus. In this context, according to the definition of emotional processing exposed in the companion paper available in this issue, the aim of the present review will consist in providing a synopsis of the studies that investigated the emotional disturbances observed in subthalamic nucleus deep brain stimulation Parkinson's disease patients. This review leads to the conclusion that several emotional components would be disrupted after subthalamic nucleus deep brain stimulation in Parkinson's disease: subjective feeling, neurophysiological activation, and motor expression. Finally, after a description of the limitations of this study model, we discuss the functional role of the subthalamic nucleus (and the striato-thalamo-cortical circuits in which it is involved) in emotional processing. It seems reasonable to conclude that the striato-thalamo-cortical circuits are indeed involved in emotional processing and that the subthalamic nucleus plays a central in role the human emotional architecture. (C) 2012 Elsevier Masson SAS. All rights reserved.
Date 2012-09
Language French
Extra Place: 21 STREET CAMILLE DESMOULINS, ISSY, 92789 MOULINEAUX CEDEX 9, FRANCE Type: Article
Volume 168
Publisher MASSON EDITEUR
Pages 642-648
Publication REVUE NEUROLOGIQUE
DOI 10.1016/j.neurol.2012.06.012
Issue 8-9
ISSN 0035-3787
Date Added 14.7.2025, 14:50:39
Modified 5.9.2025, 14:50:02

Notes:

Not Included: Not a systematic Review

Tags: EXCLUDED

Emotion and object processing in Parkinson's disease.

**Item Type**	Journal Article
**Author**	Henri Cohen
**Author**	Marie-Hélène Gagné
**Author**	Ursula Hess
**Author**	Emmanuelle Pourcher
**Abstract**	The neuropsychological literature on the processing of emotions in Parkinson's disease (PD) reveals conflicting evidence about the role of the basal ganglia in the recognition of facial emotions. Hence, the present study had two objectives. One was to determine the extent to which the visual processing of emotions and objects differs in PD. The other was to assess the impact of cognitive load on the processing of these types of information. Thirty-one patients with idiopathic PD (IPD) under dopamine replacement therapy (DRT) were compared to 30 control subjects on emotion and object recognition tasks. Recognition of objects was more accurate and faster than recognition of facial expressions of emotion, for both groups of subjects. In a second experiment using an N-back procedure with the same stimuli-a more demanding task with a higher cognitive load-patients with IPD were as accurate as control subjects in detecting the correct sequential presentation of stimuli, but were much slower in their decision responses. This indicates that IPD patients under DRT are not impaired in encoding emotion or object information, but that they have difficulty with the processing demands of the N-back task. Thus, patients with IPD appear to be more sensitive to cognitive load than to type of information, whether facial emotions or objects. In this perspective, one must consider that a deafferented dopaminergic system has problems processing more complex information before one can posit the existence of deficits affecting a specific type of information.
**Date**	2010 Apr
**Language**	eng
**License**	Copyright 2010 Elsevier Inc. All rights reserved.
**Extra**	Place: United States
**Volume**	72
**Pages**	457-463
**Publication**	Brain and cognition
**DOI**	10.1016/j.bandc.2010.01.001
**Issue**	3
**Journal Abbr**	Brain Cogn
**ISSN**	1090-2147 0278-2626
**PMID**	20167412
**Date Added**	6.7.2025, 19:09:37
**Modified**	5.9.2025, 14:31:24

Notes:

Included

sample characteristics

size: 31 PD and 30 HC (matched eduation)

Parkinson's Disease type and duration: idiopathic PD, Mduration= 4.79 (SD=2.85)

Medication: on medication

Hoehn-Yahr: Range=1-2.5

UPDRS-3: NA

Gender (male): 16 males (52%)

averaged ages (SD, range): M= 65.37 (SD=7.04)

other neurological disease (tumor, stroke, etc.): None

other major psychopathology: NA

origin country (or ethnicity): Canada

method observational

instruments used in order to quantify the variables

Social cognition aspect: Emotion recognition

Name of the task: NA

type of stimulus [face/voice etc., Ekman faces/other etc.]: Twenty individual faces (10 men, 10 women), displaying expressions were taken from standardized sets of pictures (MSFDE, NimStim). black and white on a computer screen.

task condition: anger, fear, sadness, happiness and disgust

operationalization: RT and accuracy

Main findings related to the review's scope

RT: recognition of happiness was faster for all subjects

Accuracy: no difference

Tags: Emotion recognition, behavioral

Emotion and ocular responses in Parkinson's disease

Item Type Journal Article
Author J. Dietz

Author M. M. Bradley
Author M. S. Okun
Author D. Bowers
Abstract Parkinson's disease (PD) is a neurodegenerative disease that affects motor, cognitive, and emotional functioning. Previous studies reported reduced skin conductance responses in PD patients, compared to healthy older adults when viewing emotionally arousing pictures. Attenuated skin conductance changes in PD may reflect peripheral autonomic dysfunction (e.g., reduced nerve endings at the sweat gland) or, alternatively, a more central emotional deficit. The aim of the current study was to investigate a second measure of sympathetic arousal-change in pupil dilation. Eye movements, a motor-based correlate of emotional processing, were also assessed. Results indicated that pupil dilation was significantly greater when viewing emotional, compared to neutral pictures for both PD patients and controls. On the other hand, PD patients made fewer fixations with shorter scan paths, particularly when viewing pleasant pictures. These results suggest that PD patients show normal sympathetic arousal to affective stimuli (indexed by pupil diameter), but differences in motor correlates of emotion (eye movements). (C) 2011 Elsevier Ltd. All rights reserved.
Date 2011-10
Language English
Extra Place: THE BOULEVARD, LANGFORD LANE, KIDLINGTON, OXFORD OX5 1GB, ENGLAND Type: Article
Volume 49
Publisher PERGAMON-ELSEVIER SCIENCE LTD
Pages 3247-3253
Publication NEUROPSYCHOLOGIA
DOI 10.1016/j.neuropsychologia.2011.07.029
Issue 12
ISSN 0028-3932
Date Added 14.7.2025, 14:50:40
Modified 5.9.2025, 14:33:43

Notes:

Not Included: not on SC
Tags: EXCLUDED

Emotion classification in Parkinson's disease by higher-order spectra and power spectrum features using EEG signals: A comparative study

Item Type Journal Article
Author R. Yuvaraj
Author M. Murugappan
Author Norlinah Mohamed Ibrahim
Author Mohd Iqbal Omar
Author Kenneth Sundaraj

Author Khairiyah Mohamad
Author R. Palaniappan
Author M. Satiyan
Abstract Deficits in the ability to process emotions characterize several neuropsychiatric disorders and are traits of Parkinson's disease (PD), and there is need for a method of quantifying emotion, which is currently performed by clinical diagnosis. Electroencephalogram (EEG) signals, being an activity of central nervous system (CNS), can reflect the underlying true emotional state of a person. This study applied machine-learning algorithms to categorize EEG emotional states in PD patients that would classify six basic emotions (happiness and sadness, fear, anger, surprise and disgust) in comparison with healthy controls (HC). Emotional EEG data were recorded from 20 PD patients and 20 healthy age-, education level- and sex-matched controls using multimodal (audio-visual) stimuli. The use of nonlinear features motivated by the higher-order spectra (HOS) has been reported to be a promising approach to classify the emotional states. In this work, we made the comparative study of the performance of k-nearest neighbor (kNN) and support vector machine (SVM) classifiers using the features derived from HOS and from the power spectrum. Analysis of variance (ANOVA) showed that power spectrum and HOS based features were statistically significant among the six emotional states ($p < 0.0001$). Classification results shows that using the selected HOS based features instead of power spectrum based features provided comparatively better accuracy for all the six classes with an overall accuracy of 70.10% +/- 2.83% and 77.29% +/- 1.73% for PD patients and HC in beta (13-30 Hz) band using SVM classifier. Besides, PD patients achieved less accuracy in the processing of negative emotions (sadness, fear, anger and disgust) than in processing of positive emotions (happiness, surprise) compared with HC. These results demonstrate the effectiveness of applying machine learning techniques to the classification of emotional states in PD patients in a user independent manner using EEG signals. The accuracy of the system can be improved by investigating the other HOS based features. This study might lead to a practical system for noninvasive assessment of the emotional impairments associated with neurological disorders.
Date 2014-03
Language English
Extra Place: RM 19C, LOCKHART CTR, 301-307 LOCKHART RD, WAN CHAI, 00000, HONG KONG Type: Article
Volume 13
Publisher IMR PRESS
Pages 89-120
Publication JOURNAL OF INTEGRATIVE NEUROSCIENCE
DOI 10.1142/S021963521450006X
Issue 1
ISSN 0219-6352
Date Added 14.7.2025, 14:50:38
Modified 5.9.2025, 15:02:19

Notes:

Not Included: not on SC

Tags: EXCLUDED

Emotion Detection Deficits and Decreased Empathy in Patients with Alzheimer's Disease and Parkinson's Disease Affect Caregiver Mood and Burden.

**Item Type**	Journal Article
**Author**	Maria Martinez
**Author**	Namita Multani
**Author**	Cassandra J. Anor
**Author**	Karen Misquitta
**Author**	David F. Tang-Wai
**Author**	Ron Keren
**Author**	Susan Fox
**Author**	Anthony E. Lang
**Author**	Connie Marras
**Author**	Maria C. Tartaglia
**Abstract**	Background: Changes in social cognition occur in patients with Alzheimer's disease (AD) and Parkinson's disease (PD) and can be caused by several factors, including emotion recognition deficits and neuropsychiatric symptoms (NPS). The aims of this study were to investigate: (1) group differences on emotion detection between patients diagnosed with AD or PD and their respective caregivers; (2) the association of emotion detection with empathetic ability and NPS in individuals with AD or PD; (3) caregivers' depression and perceived burden in relation to patients' ability to detect emotions, empathize with others, presence of NPS; and (4) caregiver's awareness of emotion detection deficits in patients with AD or Parkinson. Methods: In this study, patients with probable AD (N = 25) or PD (N = 17), and their caregivers (N = 42), performed an emotion detection task (The Awareness of Social Inference Test-Emotion Evaluation Test, TASIT-EET). Patients underwent cognitive assessment, using the Behavioral Neurology Assessment (BNA). In addition, caregivers completed questionnaires to measure empathy (Interpersonal Reactivity Index, IRI) and NPS (Neuropsychiatric Inventory, NPI) in patients and self-reported on depression (Geriatric Depression Scale, GDS) and burden (Zarit Burden Interview, ZBI). Caregivers were also interviewed to measure dementia severity (Clinical Dementia Rating (CDR) Scale) in patients. Results: The results suggest that individuals with AD and PD are significantly worse at recognizing emotions than their caregivers. Moreover, caregivers failed to recognize patients' emotion recognition deficits and this was associated with increased caregiver burden and depression. Patients' emotion recognition deficits, decreased empathy and NPS were also related to caregiver burden and depression. Conclusions: Changes in emotion detection and empathy in individuals with AD and PD has implications for caregiver burden and depression and may be amenable to interventions with both patients and caregivers.
**Date**	2018

Language eng
Extra Place: Switzerland
Volume 10
Pages 120
Publication Frontiers in aging neuroscience
DOI 10.3389/fnagi.2018.00120
Journal Abbr Front Aging Neurosci
ISSN 1663-4365
PMID 29740312
PMCID PMC5928197
Date Added 6.7.2025, 19:09:37
Modified 5.9.2025, 14:45:13

Notes:

Included

Sample characteristics

Size: PD = 17; AD (Alzheimers) = 25 (very mild to moderate dementia). Groups differed by gender. and Caregivers group (one per patient)

PD-type: NA

PD-duration: NA

Medication: ON state

Hoehn-Yahr: NA

UPDRS-3: NA

Gender (male): 15 (88%)

Age: 69.35 ± 8.2 years

Other neurological disease (tumor, stroke, etc.): None

Other major psychopathology: None

Origin country (or ethnicity): Canada

method (Review, meta-analysis or observational and/or self-reported):

instruments used in order to quantify the variables

Social cognition aspect: Emotion Recognition

Name of the task: The Awareness of Social Inference Test-Emotion Evaluation Test (TASIT-EET)

Type of stimulus [face/voice etc., Ekman faces/other etc.]: several short video clips, enacted by professional actors demonstrating seven emotions (happy, surprised, sad, angry, anxious, disgusted and neutral

Task condition: 7-alternative forced-choice.

Operationalization: Correct answers

Social cognition aspect: Empathy

Name of the task: Interpersonal Reactivity Index (IRI). caregivers'-report.

28 item caregivers' report questionnaire on a five point Likert scale. The scale measures both the cognitive and emotional aspects of empathy. The cognitive aspects include Perspective Taking (PT) and Fantasy (F) subscales.

Main findings related to the review's scope

TASIT-EET: patients performed worse compared to their caregivers. however, no difference between PD and AD

IRI: no difference between PD and AD

Tags: Emotion recognition, Empathy, Behavioral, Questionnaire

Emotion processing in Parkinson's disease: a blood oxygenation level-dependent functional magnetic resonance imaging study.

**Item Type**	Journal Article
**Author**	Mohammed Benzagmout
**Author**	Saïd Boujraf
**Author**	Badreeddine Alami
**Author**	Hassane Ali Amadou
**Author**	Halima El Hamdaoui
**Author**	Amine Bennani
**Author**	Mounir Jaafari
**Author**	Ismail Rammouz
**Author**	Mustapha Maaroufi
**Author**	Rabia Magoul
**Author**	Driss Boussaoud
**Abstract**	Parkinson's disease is a neurodegenerative disorder caused by loss of dopamine neurons in the substantia nigra pars compacta. Tremor, rigidity, and bradykinesia are the major symptoms of the disease. These motor impairments are often accompanied by affective and emotional dysfunctions which have been largely studied over the last decade. The aim of this study was to investigate emotional processing organization in the brain of patients with Parkinson's disease and to explore whether there are differences between recognition of different types of emotions in Parkinson's disease. We examined 18 patients with Parkinson's disease (8 men, 10 women) with no history of neurological or psychiatric comorbidities. All these patients underwent identical brain blood oxygenation level-dependent functional magnetic resonance imaging for emotion evaluation. Blood oxygenation level-dependent functional magnetic resonance imaging results revealed that the occipito-temporal cortices, insula, orbitofrontal cortex, basal ganglia, and parietal cortex which are involved in emotion processing, were activated during the functional control. Additionally, positive emotions activate larger volumes of the same anatomical entities than neutral and negative emotions. Results also revealed that Parkinson's disease associated with emotional disorders are increasingly recognized as disabling as classic motor symptoms. These findings help clinical physicians to recognize the emotional dysfunction of patients with Parkinson's disease.
**Date**	2019 Apr
**Language**	eng
**Extra**	Place: India
**Volume**	14
**Pages**	666-672
**Publication**	Neural regeneration research
**DOI**	10.4103/1673-5374.247470
**Issue**	4
**Journal Abbr**	Neural Regen Res
**ISSN**	1673-5374 1876-7958

PMID 30632507
PMCID PMC6352597
Date Added 6.7.2025, 19:09:37
Modified 5.9.2025, 14:28:32

Notes:

Not Included: not in English
Tags: EXCLUDED

Emotion processing in Parkinson's disease: a three-level study on recognition, representation, and regulation.

Item Type Journal Article
Author Ivan Enrici
Author Mauro Adenzato
Author Rita B. Ardito
Author Antonia Mitkova
Author Marco Cavallo
Author Maurizio Zibetti
Author Leonardo Lopiano
Author Lorys Castelli
Abstract BACKGROUND: Parkinson's disease (PD) is characterised by well-known motor symptoms, whereas the presence of cognitive non-motor symptoms, such as emotional disturbances, is still underestimated. One of the major problems in studying emotion deficits in PD is an atomising approach that does not take into account different levels of emotion elaboration. Our study addressed the question of whether people with PD exhibit difficulties in one or more specific dimensions of emotion processing, investigating three different levels of analyses, that is, recognition, representation, and regulation. METHODOLOGY: Thirty-two consecutive medicated patients with PD and 25 healthy controls were enrolled in the study. Participants performed a three-level analysis assessment of emotional processing using quantitative standardised emotional tasks: the Ekman 60-Faces for emotion recognition, the full 36-item version of the Reading the Mind in the Eyes (RME) for emotion representation, and the 20-item Toronto Alexithymia Scale (TAS-20) for emotion regulation. PRINCIPAL FINDINGS: Regarding emotion recognition, patients obtained significantly worse scores than controls in the total score of Ekman 60-Faces but not in any other basic emotions. For emotion representation, patients obtained significantly worse scores than controls in the RME experimental score but no in the RME gender control task. Finally, on emotion regulation, PD and controls did not perform differently at TAS-20 and no specific differences were found on TAS-20 subscales. The PD impairments on emotion recognition and representation do not correlate with dopamine therapy, disease severity, or with the duration of illness. These results are independent from other cognitive processes, such as global cognitive status and executive function, or from psychiatric status, such as depression, anxiety or apathy. CONCLUSIONS: These results may contribute to better understanding of the emotional problems that are often seen in patients with PD and the measures used to test these problems, in

	particular on the use of different versions of the RME task.
**Date**	2015
**Language**	eng
**Extra**	Place: United States
**Volume**	10
**Pages**	e0131470
**Publication**	PloS one
**DOI**	10.1371/journal.pone.0131470
**Issue**	6
**Journal Abbr**	PLoS One
**ISSN**	1932-6203
**PMID**	26110271
**PMCID**	PMC4482447
**Date Added**	6.7.2025, 19:09:35
**Modified**	5.9.2025, 14:34:56

Notes:

Included

sample characteristics

size: 32 PD and and 25 HC

Parkinson's Disease type and duration: NA

Medication: on medication

Hoehn-Yahr: M=1.60 SD=0.67

UPDRS-3: M=33.69 SD=9.26

Gender (male): 17 (53%)

averaged ages (SD, range): M=57.97 sd=7.20

other neurological disease (tumor, stroke, etc.): None

other major psychopathology: None

origin country (or ethnicity): Spain

method (Review, meta-analysis or observational and/or self-reported):

instruments used in order to quantify the variables

Social cognition aspect: emotion recognition

Name of the task: the Ekman 60-Faces Test

type of stimulus [face/voice etc., Ekman faces/other etc.]: Ekman faces

task condition: happiness, surprise, anger, disgust, fear, and sadness

operationalization: accuracy

Social cognition aspect: affective ToM

Name of the task: the Reading the Mind in the Eyes (RME)

type of stimulus [face/voice etc., Ekman faces/other etc.]: partial pictures of a face depicting only the eye region.

operationalization: accuracy

Main findings related to the review's scope

In total, PD perfomed worse than HC in Ekman, specifically anger. Other NS.

Also, worse in RMET.

Tags: Emotion recognition, ToM, behavioral

Emotion processing in Parkinson's disease: an EEG spectral power study.

**Item Type**	Journal Article
**Author**	R. Yuvaraj
**Author**	M. Murugappan
**Author**	Mohd Iqbal Omar
**Author**	Norlinah Mohamed Ibrahim
**Author**	Kenneth Sundaraj
**Author**	Khairiyah Mohamad
**Author**	M. Satiyan
**Abstract**	OBJECTIVE: Although an emotional deficit is a common finding in Parkinson's disease (PD), its neurobiological mechanism on emotion recognition is still unknown. This study examined the emotion processing deficits in PD patients using electroencephalogram (EEG) signals in response to multimodal stimuli. METHOD: EEG signals were investigated on both positive and negative emotions in 14 PD patients and 14 aged-matched normal controls (NCs). The relative power (i.e., ratio of EEG signal power in each frequency band compared to the total EEG power) was computed over three brain regions: the anterior (AF3, F7, F3, F4, F8 and AF4), central (FC5 and FC6) and posterior (T7, P7, O1, O2, P8 and T8) regions for theta (4-8 Hz), alpha (8-13 Hz), beta (13-30 Hz) and gamma (30-60 Hz) frequency sub-bands, respectively. RESULTS: Behaviorally, PD patients showed decreased performance in classifying emotional stimuli as measured by subjective ratings. EEG power at theta, alpha, beta, and gamma bands in all regions were significantly different between the NC and PD groups during both the emotional tasks, with p-values less than 0.05. Furthermore, an increase of relative spectral powers in the theta and gamma bands and a decrease of relative powers in the alpha and beta bands were observed for PD patients compared with NCs during emotional information processing. CONCLUSION: The results suggest the possibility of the existence of a distinctive neurobiological substrate of PD patients during emotional information processing. Also, these distributed spectral powers in different frequency bands might provide meaningful information about emotional processing in PD patients.
**Date**	2014 Jul
**Language**	eng
**Extra**	Place: England
**Volume**	124
**Pages**	491-502
**Publication**	The International journal of neuroscience
**DOI**	10.3109/00207454.2013.860527
**Issue**	7
**Journal Abbr**	Int J Neurosci
**ISSN**	1563-5279 0020-7454
**PMID**	24168328
**Date Added**	6.7.2025, 19:09:38
**Modified**	5.9.2025, 15:02:34

Notes:

Not Included: not on SC

Tags: EXCLUDED

Emotion processing in Parkinson's disease: Dissociation between early neuronal processing and explicit ratings

**Item Type**	Journal Article
**Author**	Matthias J. Wieser
**Author**	Andreas Mühlberger
**Author**	Georg W. Alpers
**Author**	Michael Macht
**Author**	Heiner Ellgring
**Author**	Paul Pauli
**Abstract**	Objective Patients suffering from Parkinson's disease (PD) have a diminished ability to discriminate facial expressions of emotion. We investigated early emotion discrimination deficits in PD by means of event-related potentials (ERPs). Methods Emotional pictures were presented to 14 PD patients and 14 healthy controls in a rapid serial visual presentation paradigm (three frames per second) while EEG was recorded. In addition, valence and arousal ratings were obtained for a representative subsample of 54 pictures. Results PD patients rated pictures of highly arousing content as less exciting than did healthy controls. Pictures of high compared to low emotional arousal were associated with a pronounced relative negative shift in the ERP waveform over parietal and occipital sites developing about 220ms after picture onset. This early posterior negativity (EPN) did not differ between PD and control group. Conclusions This dissociation of affective ratings and early ERP components supports the view that PD is associated with blunted emotional responses, but there is no evidence for a deteriorated early visual processing of emotional stimuli. Significance Frequently reported deficits in emotion discrimination are likely not due to deficits in early emotion processing.
**Date**	2006
**URL**	https://www.sciencedirect.com/science/article/pii/S1388245705003792
**Volume**	117
**Pages**	94-102
**Publication**	Clinical Neurophysiology
**DOI**	https://doi.org/10.1016/j.clinph.2005.09.009
**Issue**	1
**ISSN**	1388-2457
**Date Added**	6.7.2025, 19:12:36
**Modified**	5.9.2025, 15:01:02

Notes:

Not included: not on SC

Tags: EXCLUDED

Emotion Recognition and Alexithymia in Parkinson's Disease Cross-Sectional Study

**Item Type**	Journal Article
**Author**	K. Disselkamp
**Author**	H. J. M. von Piekartz
**Author**	G. Mohr
**Abstract**	Background: Basic emotions are expressed through facial expressions and are an important communicator to the outside world. Patients with Parkinson's disease lose this skill due to the often apparent mask-like face. Objective: The aim of this study was to identify differences in recognition and perception of emotion between patients with Parkinson's disease and healthy subjects. Method: 34 medically controlled patients with Parkinson's disease were examined regarding their recognition by using the facially expressed emotion labeling test and regarding their own emotion perception by using the Toronto alexi-thymia scale-26. The results were compared to data of already existing studies. Results: Patients with Parkinson's disease had significant problems in recognising mimic facial expressions in comparison to healthy subjects ($p = 0.001$). In addition, they needed significantly more time to correctly attribute the expressions to the emotions ($p = 0.001$). The final results of the FEEL test correlated highly with the response time ($p = 0.001$; $r = -0.665$). Their perception of own emotions (TAS-26) was also constricted ($p = 0.001$). Correlation between the results of the FEEL test and the TAS-26 was poor ($p = 0.020$; $r = -0.404$). Conclusions: Patients with Parkinson's disease showed deteriorations in recognising facial expressions and in attributing them to the appropriate emotions. However, alexithymia features were not found. Patients with good emotion recognition seem to have fewer problems in recognising their own emotions.
**Date**	2014-12
**Language**	German
**Extra**	Place: RUDIGERSTR 14, D-70469 STUTTGART, GERMANY Type: Article
**Volume**	10
**Publisher**	GEORG THIEME VERLAG KG
**Pages**	149-155
**Publication**	PHYSIOSCIENCE
**DOI**	10.1055/s-0034-1385472
**Issue**	4
**ISSN**	1860-3092
**Date Added**	14.7.2025, 14:50:37
**Modified**	5.9.2025, 14:33:56

Notes:

Not Included: not in English

Tags: EXCLUDED

Emotion recognition impairment and apathy after subthalamic nucleus stimulation in Parkinson's disease have separate neural substrates.

**Item Type**	Journal Article
**Author**	D. Drapier
**Author**	J. Péron
**Author**	E. Leray
**Author**	P. Sauleau
**Author**	I. Biseul
**Author**	S. Drapier
**Author**	F. Le Jeune
**Author**	D. Travers
**Author**	A. Bourguignon
**Author**	C. Haegelen
**Author**	B. Millet
**Author**	M. Vérin
**Abstract**	OBJECTIVE: To test the hypothesis that emotion recognition and apathy share the same functional circuit involving the subthalamic nucleus (STN). METHODS: A consecutive series of 17 patients with advanced Parkinson's disease (PD) was assessed 3 months before (M-3) and 3 months (M+3) after STN deep brain stimulation (DBS). Mean (+/-S.D.) age at surgery was 56.9 (8.7) years. Mean disease duration at surgery was 11.8 (2.6) years. Apathy was measured using the Apathy Evaluation Scale (AES) at both M-3 and M3. Patients were also assessed using a computerised paradigm of facial emotion recognition [Ekman, P., & Friesen, W. V. (1976). Pictures of facial affect. Palo Alto: Consulting Psychologist Press] before and after STN DBS. Prior to this, the Benton Facial Recognition Test was used to check that the ability to perceive faces was intact. RESULTS: Apathy had significantly worsened at M3 (42.5+/-8.9, p=0.006) after STN-DBS, in relation to the preoperative assessment (37.2+/-5.5). There was also a significant reduction in recognition percentages for facial expressions of fear (43.1%+/-22.9 vs. 61.6%+/-21.4, p=0.022) and sadness (52.7%+/-19.1 vs. 67.6%+/-22.8, p=0.031) after STN DBS. However, the postoperative worsening of apathy and emotion recognition impairment were not correlated. CONCLUSIONS: Our results confirm that the STN is involved in both the apathy and emotion recognition networks. However, the absence of any correlation between apathy and emotion recognition impairment suggests that the worsening of apathy following surgery could not be explained by a lack of facial emotion recognition and that its behavioural and cognitive components should therefore also be taken into consideration.
**Date**	2008 Sep
**Language**	eng
**Extra**	Place: England
**Volume**	46
**Pages**	2796-2801
**Publication**	Neuropsychologia
**DOI**	10.1016/j.neuropsychologia.2008.05.006
**Issue**	11
**Journal Abbr**	Neuropsychologia
**ISSN**	0028-3932
**PMID**	18579165

Date Added 6.7.2025, 19:09:35
Modified 5.9.2025, 14:34:20

Notes:

Not Included: not comparing HC to PD, only longitudinal effect of DBS

Tags: EXCLUDED

Emotion recognition impairment in Parkinson's disease patients without dementia.

Item Type Journal Article
Author Elena Herrera
Author Fernando Cuetos
Author Javier Rodríguez-Ferreiro
Abstract PURPOSE: Previous research has shown dementia and mild cognitive impairment to be present in some Parkinson's disease (PD) patients. Nevertheless, it is still not clear whether PD patients are also impaired on facial emotion recognition, nor it is whether this possible deficit is independent other cognitive impairment. The aim of this study is to assess the presence of emotion recognition deficits in a sample of PD patients with normal cognitive abilities, evaluated with several cognitive tasks widely used to detect cognitive impairment in this patient group. METHOD: 40 non-demented (MMSE scores>25) PD patients and 19 healthy older adults matched on demographic characteristics took part in the study. All of them were evaluated with a neuropsychological battery including tests aimed to assess the cognitive domains mainly affected by PD, as well as a facial emotion recognition task. RESULTS: t-test analysis showed significant differences between PD and control groups in 6 cognitive tasks which were introduced in a sequential logistic regression analysis. The results confirmed the existence of a facial emotion recognition deficit in PD patients after controlling for demographic and cognitive characteristics of the participants. CONCLUSION: Although none of the PD patients fulfilled criteria for dementia, many of them appeared to present deficits on recognition of facial emotions. This task should therefore be incorporated into future research to study the full range of early cognitive dysfunctions and non-motor symptoms presents in PD patients, and inclusion of this task in assessment protocols should be considered.
Date 2011 Nov 15
Language eng
License Copyright © 2011 Elsevier B.V. All rights reserved.
Extra Place: Netherlands
Volume 310
Pages 237-240
Publication Journal of the neurological sciences
DOI 10.1016/j.jns.2011.06.034
Issue 1-2
Journal Abbr J Neurol Sci
ISSN 1878-5883 0022-510X
PMID 21752398

Date Added 6.7.2025, 19:09:37
Modified 5.9.2025, 14:39:08

Notes:

Included

sample characteristics

size: 40 "regular" PD and 19 HC (matched in sex, age, education and MMSE)

Parkinson's Disease type and duration: Mduration = 7.22 SD=5.47

Medication: on medication

Hoehn-Yahr: M= 1.56 SD=0.65

UPDRS-3: NA

Gender (male): 25 males (63%)

averaged ages (SD, range): M= 69.50 sd=5.47

other neurological disease (tumor, stroke, etc.): None

other major psychopathology: None

origin country (or ethnicity): Spain

method observational

instruments used in order to quantify the variables

Social cognition aspect: emotion recognition

Name of the task: NA

type of stimulus [face/voice etc., Ekman faces/other etc.]: 18 photos of faces (half male, half female) selected from the MacBrain Face Stimulus Set

task condition: happiness, sadness, anger, surprise, disgust or fear.

operationalization: Accuracy

Main findings related to the review's scope

PD performed worse on the task then HC.

There was a significant effect of the emotion recognition task even after controlling for all the other characteristics. The model was able to accurately distinguish between healthy and PD participants in 91.5% of the cases.

Tags: Emotion recognition, behavioral

Emotion recognition in early Parkinson's disease patients undergoing deep brain stimulation or dopaminergic therapy: a comparison to healthy participants.

Item Type Journal Article
Author Lindsey G. McIntosh
Author Sishir Mannava
Author Corrie R. Camalier
Author Bradley S. Folley
Author Aaron Albritton
Author Peter E. Konrad
Author David Charles
Author Sohee Park
Author Joseph S. Neimat
Abstract Parkinson's disease (PD) is traditionally regarded as a neurodegenerative movement disorder, however, nigrostriatal dopaminergic degeneration is also thought to disrupt non-motor loops connecting basal ganglia to areas in frontal cortex involved in cognition and emotion processing. PD patients are impaired on tests of emotion recognition, but it is difficult to disentangle this deficit from the more general cognitive dysfunction that frequently accompanies disease progression. Testing for emotion recognition deficits early in the disease course, prior to cognitive decline, better assesses the sensitivity of these non-motor corticobasal ganglia-thalamocortical loops involved in emotion processing to early degenerative change in basal ganglia circuits. In addition, contrasting this with a group of healthy aging individuals demonstrates changes in emotion processing specific to the degeneration of basal ganglia circuitry in PD. Early PD patients (EPD) were recruited from a randomized clinical trial testing the safety and tolerability of deep brain stimulation (DBS) of the subthalamic nucleus (STN-DBS) in early-staged PD. EPD patients were previously randomized to receive optimal drug therapy only (ODT), or drug therapy plus STN-DBS (ODT + DBS). Matched healthy elderly controls (HEC) and young controls (HYC) also participated in this study. Participants completed two control tasks and three emotion recognition tests that varied in stimulus domain. EPD patients were impaired on all emotion recognition tasks compared to HEC. Neither therapy type (ODT or ODT + DBS) nor therapy state (ON/OFF) altered emotion recognition performance in this study. Finally, HEC were impaired on vocal emotion recognition relative to HYC, suggesting a decline related to healthy aging. This study supports the existence of impaired emotion recognition early in the PD course, implicating an early disruption of fronto-striatal loops mediating emotional function.
Date 2014
Language eng
Extra Place: Switzerland
Volume 6
Pages 349
Publication Frontiers in aging neuroscience
DOI 10.3389/fnagi.2014.00349
Journal Abbr Front Aging Neurosci
ISSN 1663-4365
PMID 25653616

PMCID PMC4301000
Date Added 6.7.2025, 19:09:36
Modified 5.9.2025, 14:45:57

Tags:

levodopa

Notes:

Included

Sample characteristics

Size: 7 PD-ODT (optimal drug therapy), 9 PD-DBS (medication + deep brain stimulation), 21 HYC (matched healthy young controls), 23 HEC (matched healthy elderly controls). Matched by age, pre morbid estimated IQ and education

PD-type: All early PD patients

PD-duration: Treated for more than 4 months and less than 6 years (no ON-OFF fluctuations)

Medication: ON state

Hoehn-Yahr: 1-2

UPDRS-3: PD-ODT (ON): M=21.43 SD= 9.88; PD-DBS (ON): M=26.13 SD= 14.43

Gender (male): PD-ODT: 5 (71%), PD-DBS: 8 (88%)

Age: PD-ODT: M=62.29 SD= 9.09; PD-DBS: M=62.22 SD= 7.97

Other neurological disease (tumor, stroke, etc.): NA

Other major psychopathology: NA

Origin country (or ethnicity): USA

method observational

instruments used in order to quantify the variables

Social cognition aspect: Emotion Recognition (voice recognition/prosody)

Name of the task: Montreal Affective Voices Task (MAV)

Type of stimulus [face/voice etc., Ekman faces/other etc.]: 90 nonverbal affect bursts. 5 mal, 5 female actors.

Task condition: anger, disgust, fear, pain, sadness, surprise, happiness, pleasure, neutral

Operationalization: After hearing each stimulus, participants chose between the nine options on the screen. Correct answers

---

Social cognition aspect: Affective ToM

Name of the task: Readin the Mind in the Eyes Task (RMET)

Type of stimulus [face/voice etc., Ekman faces/other etc.]: 36 black and white images of the eye region of faces.

Task condition: Each stimulus is presented with four adjective answer choices. Participants are instructed to choose the word that best describes what the person in the image is thinking or feeling

Operationalization: Correct answers

Social cognition aspect: Emotion Recognition

Name of the task: Awareness of Social Inference Test (TASIT) / Subset used, the Emotion Evaluation Test (EET)

Type of stimulus [face/voice etc., Ekman faces/other etc.]: 28 audiovisual vignettes. recognition of spontaneous emotional expression

Task condition: emotions: happy, surprised, sad, anxious, angry, disgusted, and neutral

Operationalization: seven forced choice response. Correct answers

Main findings related to the review's scope

There was no significant effect of treatment type ($p > 0.4$ for all tasks) or treatment state ($p > 0.5$ for all tasks) on emotion recognition task.

(PD-ODT and PD-DBS here combined, cause they did not differ regarding emotion recognition performance)

TASIT: early stage PD (EPD) patients performed worse than HEC on TASIT overall ($t(33) = 3.70$, $p = 0.001$). Specific affect impairment was found for disgusted, anxious, and neutral. Statistical trends for specific affect impairments were found for sad ($p = 0.07$), happy ($p = 0.06$), and surprised ($p = 0.05$). No other sig effects.

RMET: EPD patients also performed worse than HEC on RMET ($t(31) = 2.98$, $p = 0.006$). BFRT performance unrelated

MAV: EPD patients performed worse than HEC on MAV ($t(35) = 2.02$, $p = 0.05$). The groups also diverged in performance for angry ($t(35) = 2.57$, $p = 0.02$), and surprised ($t(35) = 3.24$, $p = 0.003$) conditions. no other sig dif.

Tags: ToM, Emotion Recognition, behavioral

Emotion Recognition in Multiple System Atrophy: An Exploratory Eye-Tracking Study.

Item Type Journal Article
Author Victoria Sidoroff
Author Federico Carbone
Author Philipp Ellmerer
Author Stefanie Bair
Author Alexandra Hoffmann
Author Thomas Maran
Author Florian Krismer
Author Philipp Mahlknecht
Author Katherina Mair
Author Cecilia Raccagni
Author Jean-Pierre Ndayisaba
Author Klaus Seppi

Author Gregor K. Wenning
Author Atbin Djamshidian

Abstract OBJECTIVE: Emotional processing is a core feature of social interactions and has been well studied in patients with idiopathic Parkinson's disease (PD), albeit with contradictory. RESULTS: . However, these studies excluded patients with atypical parkinsonism, such as multiple system atrophy (MSA). The objective of this exploratory study was to provide better insights into emotion processing in patients with MSA using eye tracking data. METHODS: We included 21 MSA patients, 15 PD patients and 19 matched controls in this study. Participants performed a dynamic and a static emotion recognition task, and gaze fixations were analyzed in different areas of interest. Participants underwent neuropsychological testing and assessment of depression and alexithymia. RESULTS: MSA patients were less accurate in recognizing anger than controls ($p = 0.02$) and had overall fewer fixations than controls ($p = 0.001$). In the static task, MSA patients had fewer fixations ($p < 0.001$) and a longer time to first fixation ($p = 0.026$) on the eye region. Furthermore, MSA patients had a longer fixation duration overall than PD patients ($p = 0.004$) and longer fixations on the nose than controls ($p = 0.005$). Alexithymia scores were higher in MSA patients compared to controls ($p = 0.038$). CONCLUSION: This study demonstrated impaired recognition of anger in MSA patients compared to HCs. Fewer and later fixations on the eyes along with a center bias suggest avoidance of eye contact, which may be a characteristic gaze behavior in MSA patients.

Date 2024 Jan
Language eng
Extra Place: Korea (South)
Volume 17
Pages 38-46
Publication Journal of movement disorders
DOI 10.14802/jmd.23090
Issue 1
Journal Abbr J Mov Disord
ISSN 2005-940X 2093-4939
PMID 37748924
PMCID PMC10846972
Date Added 6.7.2025, 19:09:37
Modified 5.9.2025, 14:56:43

Notes:

Included

Sample characteristics

Size: 15 PD, 21 multiple system atrophy (MSA), 19 HC

PD-type: Idiopathic PD

PD-duration: M = 4, Range = 3-9

Medication: ON state

Hoehn-Yahr: M = 2, Range = 2-2

UPDRS-3: total-score: M = 38.7, Sd = 19.1

Gender (male):10 (66%)

Age: M = 64.4, SD = 8.8

Other neurological disease (tumor, stroke, etc.): NA

Other major psychopathology: None

Origin country (or ethnicity): Austria

method (Review, meta-analysis or observational and/or self-reported):

instruments used in order to quantify the variables

Social cognition aspect: Emotion recognition

Name of the task: modified Geneva Emotion Recognition Test (emotion recognition dynamic task; ER-D)

Type of stimulus [face/voice etc., Ekman faces/other etc.]: 24 3,000 ms video clips presenting male and female actors demonstrating eight different emotions

Task condition: participants have to choose between the primary emotions of joy, anger, fear and disgust and the secondary emotions of surprise, amusement, pride and despair.

Operationalization: Correct answers

Social cognition aspect: Emotion recognition

Name of the task: Emotion recognition–static test (ER-S)

Type of stimulus [face/voice etc., Ekman faces/other etc.]: 63 faces selected from the NimStim-Set of Emotional Facial Expression Pictures. Photos of faces expressing three different emotions (joy, anger and fear) are shown for 6,000 ms. Every emotion is displayed at three different intensities (100% vs. 66% vs. 33%) by morphing the expression. Also neutral included.

Task condition: participants are asked to choose among neutral, joy, anger, fear, disgust, surprise or despair; this choice includes more options than the emotions displayed.

Operationalization: Correct answers

Main findings related to the review's scope

ER-D

the three groups exhibited similar recognition rates (p = 0.236)

ER-S

the three groups exhibited similar recognition rates (p = 0.66), without differences according to emotion type or intensity.

The reaction time (from stimulus onset to participant response) did not differ among the groups,

Tags: Emotion recognition, behavioral

Emotion recognition in Parkinson's disease after subthalamic deep brain stimulation: differential effects of microlesion and STN stimulation.

Item Type Journal Article

Author Marilena Aiello
Author Roberto Eleopra
Author Christian Lettieri
Author Massimo Mondani
Author Stanislao D'Auria
Author Enrico Belgrado
Author Antonella Piani
Author Luca De Simone
Author Sara Rinaldo
Author Raffaella I. Rumiati

Abstract Deep brain stimulation of the subthalamic nucleus (STN-DBS) has acquired a relevant role in the treatment of Parkinson's disease (PD). Despite being a safe procedure, it may expose patients to an increased risk to experience cognitive and emotional difficulties. Impairments in emotion recognition, mediated both by facial and prosodic expressions, have been reported in PD patients treated with such procedure. However, it is still unclear whether the STN per se is responsible for such changes or whether others factors like the microlesion produced by the electrode implantation may also play a role. In this study we evaluated facial emotions discrimination and emotions recognition using both facial and prosodic expressions in 12 patients with PD and 13 matched controls. Patients' were tested in four conditions: before surgery, both in on and off medication, and after surgery, respectively few days after STN implantation before turning stimulator on and few months after with stimulation on. We observed that PD patients were impaired in discriminating and recognizing facial emotions, especially disgust, even before DBS implant. Microlesion caused by surgical procedure was found to influence patients' performance on the discrimination task and recognition of sad facial expression while, after a few months of STN stimulation, impaired disgust recognition was again prominent. No impairment in emotional prosody recognition was observed both before and after surgery. Our study confirms that PD patients may experience a deficit in disgust recognition and provides insight into the differential effect of microlesion and stimulation of STN on several tasks assessing emotion recognition.

**Date**	2014 Feb
**Language**	eng
**License**	Copyright © 2013 Elsevier Ltd. All rights reserved.
**Extra**	Place: Italy
**Volume**	51
**Pages**	35-45
**Publication**	Cortex; a journal devoted to the study of the nervous system and behavior
**DOI**	10.1016/j.cortex.2013.11.003
**Journal Abbr**	Cortex
**ISSN**	1973-8102 0010-9452
**PMID**	24342106
**Date Added**	6.7.2025, 19:09:35
**Modified**	5.9.2025, 14:25:14

Notes:

Included

sample characteristics

size: 12 PD and 13 age-matched HC.

PD-type: NA

PD-duration: M = 10.9 (SD = 4.1)

Medication: Patients were tested ON and OFF medication

Hoehn-Yeahr: NA

UPDRS-3: ON-medication (M = 15.2, SD = 11.1), OFF-medication (M = 34.7, SD = 15.6)

Gender: 8 males (67%)

Age: M = 61.7, SD = 7.4

other neurological disease (tumor, stroke, etc.): none

other major psychopathology: None

origin country (or ethnicity): Italy

method observational

instruments used in order to quantify the variables

Social cognition aspect: emotion recognition

Name of the task: NA

type of stimulus [face/voice etc., Ekman faces/other etc.]:

Faces: colored expressions from eight individuals (four females) taken from the NimStim set, as well as 6 elderly models (three females)

Prosody: Twenty-four sentences were uttered by four different actors (two females) and controlled for length

Facial emotion discrimination: Participants were asked to judge whether two facial stimuli express the same or a different emotion.

Facial emotion recognition: Participants were asked to identify which emotion was presented and rate its intensity.

task condition: happiness, sadness, fear, surprise, disgust, and anger.

operationalization:

Faces and prosody: For every stimulus, they were asked to choose the expressed emotion among six labels and then rate their intensity using a continuous scale from 0 (not at all) to 7 (very much).

Facial emotion discrimination: Accuracy of the same/different judgment was recorded.

Main findings related to the review's scope

PD 'ON Med' versus controls: facial emotion discrimination and recognition tasks PD patients performed less well than controls. emotional prosody recognition task, no difference.

PD patients differed from controls only for the ability to recognize disgust

There was no difference in the error patterns between PD patients and the controls regarding single emotions, except for sadness. Relative to controls, patients selected more often the neutral instead of the sadness expression

The two groups did not differ either for accuracy or for the intensity ratings when single prosodic emotions were analyzed

PD 'OFF Med' versus controls: significant difference in the facial emotion discrimination. No differences were observed in facial emotion recognition, emotion prosody recognition**.** The patients' ability to recognize facial expressions and prosodic stimuli did not differ across types of emotions. The error analysis revealed that, compared with controls, the PD patients sometimes selected the neutral instead of disgust expression

Tags: Emotion recognition, behavioral

Emotion recognition in Parkinson's disease: Static and dynamic factors.

Item Type Journal Article

Author Cory I. Wasser
Author Felicity Evans
Author Clare Kempnich
Author Yifat Glikmann-Johnston
Author Sophie C. Andrews
Author Dominic Thyagarajan
Author Julie C. Stout

Abstract OBJECTIVE: The authors tested the hypothesis that Parkinson's disease (PD) participants would perform better in an emotion recognition task with dynamic (video) stimuli compared to a task using only static (photograph) stimuli and compared performances on both tasks to healthy control participants. METHOD: In a within-subjects study, 21 PD participants and 20 age-matched healthy controls performed both static and dynamic emotion recognition tasks. The authors used a 2-way analysis of variance (controlling for individual participant variance) to determine the effect of group (PD, control) on emotion recognition performance in static and dynamic facial recognition tasks. RESULTS: Groups did not significantly differ in their performances on the static and dynamic tasks; however, the trend was suggestive that PD participants performed worse than controls. CONCLUSIONS: PD participants may have subtle emotion recognition deficits that are not ameliorated by the addition of contextual cues, similar to those found in everyday scenarios. Consistent with previous literature, the results suggest that PD participants may have underlying emotion recognition deficits, which may impact their social functioning. (PsycINFO Database Record

Date 2018 Feb
Language eng
License (c) 2018 APA, all rights reserved).
Extra Place: United States

Volume 32
Pages 230-234
Publication Neuropsychology
DOI 10.1037/neu0000400
Issue 2
Journal Abbr Neuropsychology
ISSN 1931-1559 0894-4105
PMID 29035069
Date Added 6.7.2025, 19:09:34
Modified 5.9.2025, 15:00:48

Notes:

Included

Sample characteristics

Size: 21 PD (six had DBS), 20 HC (similar in age)

PD-type: NA

PD-duration: NA

Medication: ON state

Hoehn-Yahr: Range = 1-3; Median = 2.00

UPDRS-3: M = 18.81, Sd = 8.87

Gender (male): 15 (71%)

Age: M = 63.39, SD = 8.16, Range = 44-74

Other neurological disease (tumor, stroke, etc.): none

Other major psychopathology: none

Origin country (or ethnicity): Australia

method behavioural

instruments used in order to quantify the variables

Social cognition aspect: emotion recognition

Name of the task: NA

Type of stimulus [face/voice etc., Ekman faces/other etc.]: series of images of faces (Karolinska Directed Emotional Faces dataset)

Task condition: six emotions (neutral, surprised, sad, angry, fearful, disgusted)

Operationalization: 6 way forced choice. >> accuracy

Social cognition aspect: emotion recognition

Name of the task: Part 1 of TASIT-R (Form A), the Emotion Evaluation Test.

Type of stimulus [face/voice etc., Ekman faces/other etc.]: 28 video scenes of actors portraying emotions. Designed to emulate real life scenarios, in which emotions are expressed dynamically over time.

Task condition: happiness, disgust, surprise, anger, fear, sadness, neutral.

Operationalization: 6-way forced choice >> accuracy

Main findings related to the review's scope

Group (PD, control) as well as interactions with group were not significantly significant in both tasks.

Tags: Emotion recognition, behavioral

Emotion recognition in patients with idiopathic Parkinson's disease.

Item Type Journal Article
Author James T. H. Yip
Author Tatia M. C. Lee
Author Shu-Leong Ho
Author Kin-Lun Tsang
Author Leonard S. W. Li
Abstract Emotion recognition (ER) was examined in 64 patients with idiopathic Parkinson's disease (PD; 56 bilateral and 8 right-sided) and 64 matched healthy volunteers. Participants were administered an ER battery, consisting of the following subscores: overall ER (OER), overall facial ER, facial emotion identification (FEI) and discrimination, overall prosodic ER, and prosodic emotion identification (PEI) and discrimination. Measures of visuospatial functions, auditory attention, and depression were also administered. After controlling for visuospatial functions, auditory attention and depression, results indicated that patients with bilateral PD had poorer performance on all ER subscores, regardless of the modality and type of experimental task involved, relative to healthy volunteers. However, patients with right-sided PD had difficulty on FEI and PEI only. Whereas none of the clinical variables examined in this study predicted any of the ER subscores, visual organization and auditory attention positively predicted OER in patients with PD. In addition, visual organization also positively predicted FEI in these patients. Implications are discussed in terms of the neural substrates underlying ER.
Date 2003 Oct
Language eng
License Copyright 2003 Movement Disorder Society
Extra Place: United States
Volume 18
Pages 1115-1122
Publication Movement disorders : official journal of the Movement Disorder Society
DOI 10.1002/mds.10497
Issue 10
Journal Abbr Mov Disord
ISSN 0885-3185
PMID 14534914
Date Added 6.7.2025, 19:09:37
Modified 5.9.2025, 15:01:38

Notes:

Included

Sample characteristics

Size: 64 PD (56 bilateral - PD-b, 8 right-sided - PD-r) , 64 HC (matched on chronological age, sex, years of formal education, and performance on the Test of Nonverbal Intelligence)

PD-type: Idiopathic PD

PD-duration: PD-b: M = 7.21, SD = 4.00; PD-r: M = 2.78, SD = 1.61

Medication: ON state

Hoehn-Yahr: M=2.84 SD=0.88 (1-5)

UPDRS-3: NA

Gender (male): PD-b: 33 (59%); PD-r: 4 (50%)

Age: PD-b: M = 64.47, SD = 11.78; PD-r: M = 65.34, SD = 14.48

Other neurological disease (tumor, stroke, etc.): None

Other major psychopathology: none

Origin country (or ethnicity): China

method (Review, meta-analysis or observational and/or self-reported):

instruments used in order to quantify the variables

Social cognition aspect: Emotion Recognition

Name of the task: NA

Type of stimulus [face/voice etc., Ekman faces/other etc.]: Photographs of 6 male and 6 female Japanese were selected from the Japanese and Caucasian Facial Expressions of Emotion (JACFEE) compilation

Task condition: . Each photograph conveyed one of the six basic emotions (e.g., happy, sad, anger, surprise, disgust, and fear) and was posed by a different individual.

Operationalization: These photographs were taken together and administered in an identification task (12 test items) and a discrimination task (30 test items).

For the former, these photographs were presented individually for 10 seconds on a personal computer. Participants were asked to indicate which of the six basic emotions was conveyed in each photograph by pointing to one of the six emotion labels or verbally referring to the emotion labels (or the associated numerical reference).

For the discrimination task, all possible pairs of photographs were formulated and placed side by side on the computer screen. Each pair was presented for 10 seconds and participants were asked to indicate which of the two photographs conveyed a specified emotion (e.g., happy) by pointing to or verbally referring to “1” or “2” on the screen. Only photographs of the same gender were paired in the discrimination task.

>> accuracy

An overall facial emotion recognition (OFER) score was calculated by adding together the scores on individual test items. Separate scores were calculated for facial emotion identification (FEI) and facial emotion discrimination (FED).

Social cognition aspect: Emotion recognition

Name of the task: NA

Type of stimulus [face/voice etc., Ekman faces/other etc.]: Spoken sentence. Neutral sentence "I want to go to see a movie" and one neutral Chinese character (word), each verbally produced with each of the six basic emotions, 48 with a male and female speaker).

Task condition: Neutral + six basic emotions

As in facial emotion-recognition, identification + discrimination tasks

Operationalization: same as in the facial emotion-recognition

Main findings related to the review's scope

Emotion Recognition in Patients With PD:

patients with bilateral PD performed significantly worse than their corresponding healthy volunteers in terms of emotion recognition, regardless of the modality (facial or prosodic) or the type of experimental tasks involved (identification or discrimination).

Although patients with bilateral PD were impaired in recognizing all six basic emotions, the recognition of fear, followed by sadness is relatively more impaired than other basic emotions.

In contrast, patients with right-sided PD were impaired (relative to their respective healthy volunteers) on facial and prosody identification only.

Furthermore, the recognition of all emotions in patients with right-sided PD was significantly impaired, except for happiness, relative to healthy volunteers.

Specifically, patients with right-sided PD were most impaired on the recognition of sadness, followed by disgust

Tags: emotion recognition, behavioral

Emotion Recognition in Patients With Parkinson Disease.

**Item Type**	Journal Article
**Author**	Hai-Bo Chen
**Author**	Dong-Dong Wu
**Author**	Jing He
**Author**	Shu-Hua Li
**Author**	Wen Su
**Abstract**	BACKGROUND: Individuals with Parkinson disease (PD) display cognitive dysfunction. However, few studies have investigated how facial and musical emotion recognition are affected in individuals with PD. OBJECTIVE: To explore the relationship between facial and musical emotion recognition and executive functions in Chinese individuals with PD. METHODS: We showed 40 Chinese individuals with

PD and 40 Chinese healthy controls 24 black-and-white portraits and 24 musical excerpts that were designed to express happiness, sadness, fear, and anger. Then, we used four tests to assess the participants' executive functions, including the Trail Making Test (TMT), Clock Drawing Test (CDT), semantic Verbal Fluency Test (VFT), and Digit Span Test (DST). RESULTS: The PD group showed significant impairment in recognizing anger from facial expressions, although their emotion recognition from musical excerpts was similar to that of the control group. Recognition of an angry face was significantly correlated to scores on the TMT and DST. Recognition of happy music was significantly correlated to the Hamilton Rating Scale for Depression score, whereas recognition of angry music was significantly correlated to musical knowledge background. Recognition of happy, sad, or angry music was significantly correlated to tests of executive function, whereas recognition of fearful music was not. CONCLUSIONS: The PD group showed impaired recognition of angry faces, which may be related to executive dysfunction. However, the PD group did not show any difficulties in recognizing emotions in music. This dissociation indicates that the mechanisms underlying the recognition of emotions in faces and music are partly independent.

Date 2019 Dec
Language eng
Extra Place: United States
Volume 32
Pages 247-255
Publication Cognitive and behavioral neurology : official journal of the Society for Behavioral and Cognitive Neurology
DOI 10.1097/WNN.0000000000000209
Issue 4
Journal Abbr Cogn Behav Neurol
ISSN 1543-3641 1543-3633
PMID 31800485
Date Added 6.7.2025, 19:09:35
Modified 5.9.2025, 14:30:13

Notes:

Included

sample characteristics

size: 40 PD and 40 HC

Parkinson's Disease type and duration: NA, Median duration=8 (3-12)

Medication: on medication

Hoehn-Yahr: M= 2.31 SD=0.84

UPDRS-3: M= 34.38 SD=13.23

Gender (male): 27 males (68%)

averaged ages (SD, range): M= 60.50 (SD=6.76) 46-69

other neurological disease (tumor, stroke, etc.): None

other major psychopathology: None

origin country (or ethnicity): China

method observational

instruments used in order to quantify the variables

Social cognition aspect: emotion recognition

Name of the task: NA. facial emotion task system developed by the neurology department of Beijing Hospital, which was derived from *Facial Expressions: A Visual Reference for Artists*

type of stimulus [face/voice etc., Ekman faces/other etc.]: black-and-white portraits — six photos for each of the four facial expressions — sorting them so that each adjacent picture showed a different emotion

task condition: happiness, sadness, fear, and anger

operationalization: accuracy

Name of the task: NA.

Main findings related to the review's scope

Faces: The angry face recognition score in the PD group was significantly lower than that in the HCs group. No other sig dif

Tags: Emotion recognition, behavioral

Emotion-Specific Affective Theory of Mind Impairment in Parkinson's Disease.

**Item Type**	Journal Article
**Author**	Rwei-Ling Yu
**Author**	Po See Chen
**Author**	Shao-Ching Tu
**Author**	Wei-Chia Tsao
**Author**	Chun-Hsiang Tan
**Abstract**	The neuropathology of Parkinson's disease (PD) involves the frontal-subcortical circuit, an area responsible for processing affective theory of mind (ToM). Patients with PD are expected to experience deficits in the affective ToM. This study aims to investigate whether the ability to infer emotion in others is affected in either young-onset Parkinson's disease (YOPD) or middle-onset PD (MOPD) patients and to test whether the impairments in affective ToM are associated with the motor symptoms. The affective ToM, global mental abilities, and clinical symptoms were assessed in a total of 107 MOPD, 30 YOPD, and 30 normal controls (NCs). The MOPD patients exhibited deficits in affective ToM to the negative and neutral valences, when compared to the participants in the NCs and YOPD group. By conducting gender-stratified analysis, the deficits in affective ToM was only found in female participants. After adjusting for demographic variables, the multiple linear regression model revealed that affective ToM predicted motor symptoms, especially in female MOPD patients. The present study may aid in the development of medical care programs by advocating for a more comprehensive therapeutic plan that includes continuous disease progression monitoring and social skills training for female MOPD patients or their caregivers.
**Date**	2018 Oct 30
**Language**	eng
**Extra**	Place: England
**Volume**	8
**Pages**	16043
**Publication**	Scientific reports
**DOI**	10.1038/s41598-018-33988-6
**Issue**	1
**Journal Abbr**	Sci Rep
**ISSN**	2045-2322
**PMID**	30375420
**PMCID**	PMC6207749
**Date Added**	6.7.2025, 19:09:40
**Modified**	5.9.2025, 15:01:58

Notes:

Included

Sample characteristics

Size: 30 young-onset PD (YOPD), 30 middle-onset PD (MOPD), 30 HC

For primary study: 60 patients with PD (30 YOPD and 30 MOPD) and 30 NCs were included for the purpose of matching for sex, education level, mental state, disease severity, and levodopa equivalent daily dose.

(Further recruited 77 MOPD patients to explore the relationship between affective ToM and PD-related clinical characteristics) -> Relationship between RMET and PD-related clinical variables (motor and non-motor symptoms)

PD-type: No atypical PD; YOPD = onset < 50 years old; MOPD after 50 and before 70

PD-duration: YOPD: M = 8.13, SD = 4.58, Range = 0.66-17; MOPD: M = 6.43, SD = 3.79, Range = 2-16

Medication: L-dopa equivalent dose mentioned. No further information.

Hoehn-Yahr: YOPD: M = 2.30, SD = 0.95, Range = 1-4; MOPD: M = 2.30, SD = 0.83, Range = 1-4

UPDRS-3: YOPD: M = 12.46, SD = 8.7, SD = 0-29; MOPD: M = 10.86, SD = 7.32, Range = 0-30

Gender (male): YOPD: 17 (57%); MOPD: 16 (53%)

Age: YOPD: M = 53.22, SD = 5.29, Range = 41-63; MOPD: M = 61.40, SD = 5.43, Range = 54-75

Other neurological disease (tumor, stroke, etc.):

Other major psychopathology: No psychiatric disorders

Origin country (or ethnicity): Taiwan

method observational

instruments used in order to quantify the variables

Social cognition aspect: Affective ToM

Name of the task: Rading the mind in the eye test (RMET)

Type of stimulus [face/voice etc., Ekman faces/other etc.]: 36 black-and-white photographed pairs of eyes that each depict a particular emotion : 8 positive, 12 negative, and 16 neutral valences

Task condition: The participants were asked to pick an adjective (out of 4) that best fit the emotion expressed by the photograph (e.g., which of the following adjectives best describes the eye region shown: excited, relieved, shy, or despondent).

Operationalization: Number of correct answers (Separate analysis for total score, positiv/negative/ neutral emotions.

Main findings related to the review's scope

A significant main effect of group in the total score of RMET. Post hoc tests indicated that the MOPD group had the worst performance on the RMET. YOPD and HC had similar performance.

Out of the three emotional valences of the RMET (positive, negative, and neutral), the MOPD group performed significantly poorly on decoding negative and neutral valences compared to the YOPD or the NC group. No significant difference was found in the positive valence among the three study groups.

Effect only found in female population. Not the case for male PD patients.

Tags: ToM, behavioral

Emotional and cognitive social processes are impaired in Parkinson's disease and are related to behavioral disorders.

Item Type Journal Article

Author Pauline Narme
Author Harold Mouras
Author Martine Roussel
Author Cécile Duru
Author Pierre Krystkowiak
Author Olivier Godefroy

Abstract OBJECTIVE: Parkinson's disease (PD) is associated with behavioral disorders that can affect social functioning but are poorly understood. Since emotional and cognitive social processes are known to be crucial in social relationships, impairment of these processes may account for the emergence of behavioral disorders. METHOD: We used a systematic battery of tests to assess emotional processes and social cognition in PD patients and relate our findings to conventional neuropsychological data (especially behavioral disorders). Twenty-three PD patients and 46 controls (matched for age and educational level) were included in the study and underwent neuropsychological testing, including an assessment of the behavioral and cognitive components of executive function. Emotional and cognitive social processes were assessed with the Interpersonal Reactivity Index caregiver-administered questionnaire (as a measure of empathy), a facial emotion recognition task and two theory of mind (ToM) tasks. RESULTS: When compared with controls, PD patients showed low levels of empathy ($p = .006$), impaired facial emotion recognition (which persisted after correction for perceptual abilities) ($p = .001$), poor performance in a second-order ToM task ($p = .008$) that assessed both cognitive ($p = .004$) and affective ($p = .03$) inferences and, lastly, frequent dysexecutive behavioral disorders (in over 40% of the patients). Overall, impaired emotional and cognitive social functioning was observed in 17% of patients and was related to certain cognitive dysexecutive disorders. In terms of behavioral dysexecutive disorders, social behavior disorders were related to impaired emotional and cognitive social functioning ($p = .04$) but were independent of cognitive impairments. CONCLUSIONS: Emotional and cognitive social processes were found to be impaired in Parkinson's disease. This impairment may account for the emergence of social behavioral disorders.

Date 2013 Mar
Language eng
License PsycINFO Database Record (c) 2013 APA, all rights reserved.
Extra Place: United States
Volume 27

**Pages**	182-192
**Publication**	Neuropsychology
**DOI**	10.1037/a0031522
**Issue**	2
**Journal Abbr**	Neuropsychology
**ISSN**	1931-1559 0894-4105
**PMID**	23527646
**Date Added**	6.7.2025, 19:09:37
**Modified**	5.9.2025, 14:48:00

Notes:

Included

Sample characteristics

Size: 23 PD, 46 HC (matched for age and education)

PD-type: Idiopathic PD

PD-duration: M = 5.9, SD = 5.6

Medication: on medication

Hoehn-Yahr: M=2.48 SD=0.56 (2-4)

UPDRS-3: M = 18.1, SD = 7.7

Gender (male): 14 (61%)

Age: M = 62.8 SD = 12.2

Other neurological disease (tumor, stroke, etc.): None

Other major psychopathology: None

Origin country (or ethnicity): France

method behavioral

instruments used in order to quantify the variables

Social cognition aspect: Empathy

Name of the task: Interpersonal Reactivity Index (IRI)

Type of stimulus [face/voice etc., Ekman faces/other etc.]: Questionnaire

Task condition: Estimated by caregivers

Operationalization: Sum-score of total empathy

Social cognition aspect: Facial emotion recognition

Name of the task: NA

Type of stimulus [face/voice etc., Ekman faces/other etc.]: Faces of 10 people expressing one of five emotions or a neutral expression from Ekman database

Task condition: happiness, fear, anger, disgust, or sadness, neutral

Operationalization: Participants were required to choose the target emotion. accuracy.

Social cognition aspect: ToM

Name of the task: Faux Pas test

Type of stimulus [face/voice etc., Ekman faces/other etc.]: Twenty stories (10 of which featured a faux pas) were read aloud to participants, who were then asked to indicate whether a character had said

something that they should not have said

Task condition: One of the questions tested cognitive ToM (was the faux pas intentional?) and another assessed affective ToM (how did the recipient feel?

Operationalization: We awarded one point for each correctly identified faux pas story (maximum score for "hits": 10). When participants identified a faux pas story, five explanatory questions were then posed (maximum score: 50)

The maximum score was 10 for questions on each type of ToM (i.e., cognitive ToM and affective ToM). A total score (of 60 at most) was computed by adding the number of hits to the explanation score

---

Social cognition aspect: ToM

Name of the task: Yoni

Type of stimulus [face/voice etc., Ekman faces/other etc.]: Fifty-four on-screen trials consisted of Yoni's face surrounded by four pictures (e.g., animals, fruits, or faces)

Task condition: By assessing Yoni's gaze and facial expression, participants had to complete a sentence by choosing the picture to which the character was referring. There were two ToM-based conditions (to test cognitive ToM ["Yoni is thinking of . . ."] and affective ToM ["Yoni likes . . ."]) and a control condition requiring the analysis of physical attributes. The items also differed in complexity, with first-order and second-order inferences (e.g., for cognitive ToM: "Yoni is thinking of the car that . . . wants").

Operationalization: Accuracy

Main findings related to the review's scope

Empathy Questionnaire

The overall IRI score was significantly lower in PD patients (53.1 12.9) than in HCs (62.3 11.4; p .006), suggesting lower levels of empathy in PD.

Facial Emotion Recognition

The ANCOVA revealed (i) a main effect of group due to worse performance in PD patients than in HCs; (ii) the lack of a significant effect of emotion, and (iii) a significant group emotion interaction, due to worse performance in PD patients than HCs for fear, sadness, and neutral expressions.

ToM abilities

Faus-pas

A one-way ANOVA indicated lower scores in the PD patients than in HCs for the total composite score (F(1) 10.76; p .002) and the explanation score (F(1) 11.97; p .001).

Aspects related to cognitive and affective inferences were also impaired in PD (cognitive ToM score: F(1) 7.32; p .009; affective ToM score: F(1) 5.04; p .028). However, PD patients also had a lower comprehension score than HCs (F(1) 35.12; p .001), suggesting that this result might be due to the impairment of non-empathic processes.

The intergroup differences disappeared after correcting for impaired understanding. Hence, these findings indicate that the PD patients' poor results in the faux pas task were **attributable to impaired understanding.**

Yoni task

An ANOVA with repeated measures on order (first, second) and condition (cognitive, affective, physical), and with group (PD, HC) as between-subjects factor revealed (i) a main group effect (F(1) 6.06; p .02), with lower scores in the PD group (83.2 1.5) than in the HC group (88.6 1.1); (ii) a significant effect of order (F(1) 87.673; p .001), due to lower scores for second-order inferences (75.8 1.6) than first-order inferences (95.97 0.8); and (iii) a significant group order interaction (F(1, 66) 7.49; p .008), due to worse performance in PD patients than HCs for second-order inferences.

PD patients had lower scores than HCs for both cognitive (62.7 4.2 vs. 76.8 2.9; F(1) 8.66; p .004) and affective ToM (70.3 3.7 vs. 79.2 2.6; F(1) 5.08; p .03) but not for the control condition (F(1) 1.91; p .18.

This finding suggests that both cognitive and affective second order ToM abilities were impaired in PD

A further ANCOVA was performed with the DRS score as covariate. The result was not statistically significant, suggesting that ToM impairment on the Yoni task is not attributable to cognitive status.

Tags: Emotion recognition, Empathy, ToM, behavioral, Questionnaire

---

Emotional atypical arousal ratings for unpleasant stimuli in patients with Parkinson's disease

**Item Type**	Journal Article
**Author**	Simone Migliore
**Author**	Stefano Toro
**Author**	Francesca Proietti
**Author**	Alessandro Magliozzi
**Author**	Gaia Anzini
**Author**	Francesca Pistoia
**Author**	Vincenzo Di Lazzaro
**Author**	Giuseppe Curcio
**Author**	Massimo Marano
**Date**	2024-08
**Volume**	45
**Pages**	3785-3790
**Publication**	NEUROLOGICAL SCIENCES
**DOI**	10.1007/s10072-024-07434-7
**Issue**	8
**ISSN**	1590-1874
**Date Added**	14.7.2025, 14:48:39
**Modified**	5.9.2025, 14:46:48

Notes:

<u>**Not Included: not on SC**</u>

Tags: EXCLUDED

Emotional dysfunctions in neurodegenerative diseases.

Item Type Journal Article
Author Leonie A. K. Löffler
Author Sina Radke
Author Carmen Morawetz
Author Birgit Derntl
Abstract Neurodegenerative diseases are characterized primarily by motor signs but are also accompanied by emotional disturbances. Because of the limited knowledge about these dysfunctions, this Review provides an overview of emotional competencies in Huntington's disease (HD), Parkinson's disease (PD), and multiple sclerosis (MS), with a focus on emotion recognition, emotion regulation, and depression. Most studies indicate facial emotion recognition deficits in HD and PD, whereas data for MS are inconsistent. On a neural level, dysfunctions of amygdala and striatum, among others, have been linked to these impairments. These dysfunctions also tap brain regions that are part of the emotion regulation network, suggesting problems in this competency, too. Research points to dysfunctional emotion regulation in MS, whereas findings for PD and HD are missing. The high prevalence of depression in all three disorders emphasizes the need for effective therapies. Research on emotional disturbances might improve treatment, thereby increasing patients' and caregivers' well-being.
Date 2016 Jun 1
Language eng
License © 2015 Wiley Periodicals, Inc.
Extra Place: United States
Volume 524
Pages 1727-1743
Publication The Journal of comparative neurology
DOI 10.1002/cne.23816
Issue 8
Journal Abbr J Comp Neurol
ISSN 1096-9861 0021-9967
PMID 26011035
Date Added 6.7.2025, 19:09:37
Modified 5.9.2025, 14:43:55

Notes:

Not Included: not a systematic review

Tags: EXCLUDED

[Emotional facial expression recognition impairment in Parkinson disease].

Item Type Journal Article
Author Karine Lachenal-Chevallet

Author Benoit Bediou
Author Martine Bouvard
Author Stéphane Thobois
Author Emmanuel Broussolle
Author Alain Vighetto
Author Pierre Krolak-Salmon
Abstract INTRODUCTION: some behavioral disturbances observed in Parkinson's disease (PD) could be related to impaired recognition of various social messages particularly emotional facial expressions. METHOD: facial expression recognition was assessed using morphed faces (five emotions: happiness, fear, anger, disgust, neutral), and compared to gender recognition and general cognitive assessment in 12 patients with Parkinson's disease and 14 controls subjects. RESULTS: facial expression recognition was impaired among patients, whereas gender recognitions, visuo-perceptive capacities and total efficiency were preserved. Post hoc analyses disclosed a deficit for fear and disgust recognition compared to control subjects. CONCLUSION: the impairment of emotional facial expression recognition in PD appears independent of other cognitive deficits. This impairment may be related to the dopaminergic depletion in basal ganglia and limbic brain regions. They could take a part in psycho-behavioral disorders and particularly in communication disorders observed in Parkinson's disease patients.
Date 2006 Mar
Language fre
Extra Place: France
Volume 4
Pages 61-67
Publication Psychologie & neuropsychiatrie du vieillissement
Issue 1
Journal Abbr Psychol Neuropsychiatr Vieil
ISSN 1760-1703
PMID 16556519
Date Added 6.7.2025, 19:09:39
Modified 5.9.2025, 14:42:46

Notes:

Not Included: Not in English
Tags: EXCLUDED

Emotional facial imagery, perception, and expression in Parkinson's disease.

Item Type Journal Article
Author D. H. Jacobs
Author J. Shuren
Author D. Bowers
Author K. M. Heilman

Abstract Patients with Parkinson's disease (PD) may be impaired at expressing emotional faces and perceiving emotional facial affect. We tested the hypothesis that patients with PD may be impaired at imaging emotional faces. We first compared 12 patients with PD and 30 control subjects on perceptual and imagery tasks. Patients were significantly impaired on a task of emotional facial imagery but not on a control task of object imagery. Patients were also impaired on a task of perceiving emotional faces. Subsequently, we found that PD patients were impaired relative to controls on making emotional faces. Performance on both the perceptual and motor tasks of facial expression significantly correlated with performance on the emotional facial imagery task. We suggest that the basal ganglia, together with the right hemisphere, are part of a neural network subserving emotional facial tasks.

Date 1995 Sep
Language eng
Extra Place: United States
Volume 45
Pages 1696-1702
Publication Neurology
DOI 10.1212/wnl.45.9.1696
Issue 9
Journal Abbr Neurology
ISSN 0028-3878
PMID 7675229
Date Added 6.7.2025, 19:09:39
Modified 5.9.2025, 14:40:30

Notes:

Included

sample characteristics

size: 12 PD and 30 HC

Parkinson's Disease type and duration: NA

Medication: NA

Hoehn-Yahr: NA

UPDRS-3: NA

Gender (male): 5 males (42%)

averaged ages (SD, range): M= 70.6 SD= 8.0

other neurological disease (tumor, stroke, etc.): NA

other major psychopathology: NA

origin country (or ethnicity): USA

method (Review, meta-analysis or observational and/or self-reported):

instruments used in order to quantify the variables

Social cognition aspect: emotion recognition

Name of the task: the Florida Affect Battery-revised (FAB-r)

type of stimulus [face/voice etc., Ekman faces/other etc.]: subtest 2 - (facial affect discrimination: subjects had to determine whether two faces depicted the same or different emotional expressions) and subset 5 - (facial affect matching: subjects were asked to match the picture of an emotional face to another face with the same emotional expression. The subjects were shown a stimulus slide consisting of multiple photographs. On the left side of the slide, there was a single photograph of a target emotional face. To the right of the slide, there were pictures of five people, expressing different emotional expressions. Their task was to match the target expression with its counterpart on the right of the slide.)

task condition: NA

operationalization: Accuracy

Main findings related to the review's scope

PD patients who were better at subset 2 were also better in subset 5.

PD were less accurate in affect discrimination (subset2) then HC.

Tags: Emotion recognition, behavioral

Emotional impairment in Parkinson's disease

Item Type Journal Article
Author Mitsuru Kawamura
Author Mutsutaka Kobayakawa
Abstract Patients with Parkinson's disease (PD) may show emotional impairment in the early stages of the disease. PD patients show disadvantageous decision-making. which is related to decreased emotional responses, its measured by skin conductance responses (SCRs). This pattern of decreasing SCRs is similar to that observed in amygdala-damaged patients. In facial expression recognition, PD patients did not show amygdala activation. In another study, PD patients did not show amygdala activations to unpleasant olfactory Stimuli, which were observed in normal controls. Emotional impairment in PD patients may reflect amygdala dysfunction in early PD. (c) 2008 Elsevier Ltd. All rights reserved.
Date 2009-01
Language English
Extra Place: THE BOULEVARD, LANGFORD LANE, KIDLINGTON, OXFORD OX5 1GB, OXON, ENGLAND Type: Article; Proceedings Paper
Volume 15
Publisher ELSEVIER SCI LTD
Pages S47-S52
Publication PARKINSONISM & RELATED DISORDERS
DOI 10.1016/S1353-8020(09)70013-6
Issue 1
ISSN 1353-8020
Date Added 14.7.2025, 14:50:42
Modified 5.9.2025, 14:41:19

Notes:

Not Included: Mostly no SC, and when examined emotion recognition, used EEG.
Tags: EXCLUDED

Emotional Processing following Cortical and Subcortical Brain Damage: Contribution of the Fronto-Striatal Circuitry

Item Type Journal Article
Author Caterina Breitenstein
Author Irene Daum
Author Hermann Ackermann
Abstract The present study examined the differential contribution of cortical and subcortical brain structures in emotional processing by comparing patients with focal cortical lesions (n = 32) to those with primarily subcortical dysregulation of the basal ganglia (Parkinson's disease n = 14). A standardized measure of emotional perception (Tübingen Affect Battery) was used. Only patients in the more advanced

stages of Parkinson's disease and patients with focal damage to the (right) frontal lobe differed significantly from controls in both facial expression and affective prosody recognition. The findings imply involvement of the fronto-striatal circuitry in emotional processing.

**Date**	01/1998
**Language**	en
**Short Title**	Emotional Processing following Cortical and Subcortical Brain Damage
**Library Catalog**	DOI.org (Crossref)
**URL**	https://onlinelibrary.wiley.com/doi/10.1155/1998/579029
**Accessed**	11.8.2025, 18:13:03
**Volume**	11
**Pages**	29-42
**Publication**	Behavioural Neurology
**DOI**	10.1155/1998/579029
**Issue**	1
**Journal Abbr**	Behavioural Neurology
**ISSN**	0953-4180, 1875-8584
**Date Added**	11.8.2025, 18:13:03
**Modified**	11.8.2025, 18:13:03

Notes:

Included

sample characteristics

size: 14 PD and 12 HC matched for age, sex, and IQ

Parkinson's Disease type and duration: NA, Mduration= 4.43 SD= 2.47

Medication: on medication

Hoehn-Yahr: M=1.5 SD=0.5 (1-2)

UPDRS-3: NA

Gender (male): 8 males (57%)

averaged ages (SD, range): M= 51.1 SD= 12.43

other neurological disease (tumor, stroke, etc.): None

other major psychopathology: None

origin country (or ethnicity): Germany

method observational

instruments used in order to quantify the variables

Social cognition aspect: emotion recognition

Name of the task: the 'Florida Affect Battery – Revised'

type of stimulus [face/voice etc., Ekman faces/other etc.]: The battery includes ten subtests (see Table 4): five subtests require discrimination, naming, pointing to, or matching of FACIAL STIMULI; in three subtests the subjects are asked to discriminate or name LINGUISTIC (1 subtest)and AFFECTIVE PROSODIC (2 subtests) sentences; in the remaining two subtests the subjects are instructed to MATCH a facial expression to one of three affective prosodic sentences or vice versa (crossmodal matching).

task conditions: happiness, anger, sadness, fear, and neutral

operationalization: accuracy

Main findings related to the review's scope

PD-II (bilateral symptoms) patients scored significantly lower in all subtests (except linguistic prosody) compared to HC and performed significantly worse than the PD-I (unilateral symptoms) group in the matching task. PD-I patients did not differ from HC in any of the measures

Tags: Emotion recognition, behavioral

Emotional processing in Parkinson's disease and schizophrenia: evidence for response bias deficits in PD.

**Item Type**	Journal Article
**Author**	Ilona P. Laskowska
**Author**	Ludwika Gawryś
**Author**	Szymon Łęski
**Author**	Dariusz Koziorowski
**Abstract**	Deficits in facial emotion recognition in Parkinson's disease (PD) patients has been well documented. Nevertheless, it is still not clear whether facial emotion recognition deficits are secondary to other cognitive impairments. The aim of this study was to answer the question of whether deficits in facial emotion recognition in PD result from impaired sensory processes, or from impaired decision processes. To address this question, we tested the ability to recognize a mixture of basic and complex emotions in 38 non-demented PD patients and 38 healthy controls matched on demographic characteristics. By using a task with an increased level of ambiguity, in conjunction with the signal detection theory, we were able to differentiate between sensitivity and response bias in facial emotion recognition. Sensitivity and response bias for facial emotion recognition were calculated using a d-prime value and a c index respectively. Our study is the first to employ the EIS-F scale for assessing facial emotion recognition among PD patients; to test its validity as an assessment tool, a group comprising schizophrenia patients and healthy controls were also tested. Patients with PD recognized emotions with less accuracy than healthy individuals (d-prime) and used a more liberal response criterion (c index). By contrast, patients with schizophrenia merely showed diminished sensitivity (d-prime). Our results suggest that an impaired ability to recognize facial emotions in PD patients may result from both decreased sensitivity and a significantly more liberal response criteria, whereas facial emotion recognition in schizophrenia may stem from a generalized sensory impairment only.
**Date**	2015
**Language**	eng
**Extra**	Place: Switzerland
**Volume**	6
**Pages**	1417
**Publication**	Frontiers in psychology
**DOI**	10.3389/fpsyg.2015.01417
**Journal Abbr**	Front Psychol
**ISSN**	1664-1078
**PMID**	26441788
**PMCID**	PMC4585298
**Date Added**	6.7.2025, 19:09:38
**Modified**	5.9.2025, 14:42:50

Notes:

Included

sample characteristics

size: 38 PD and 38 HC (matched for sex, age, and education)

Parkinson's Disease type and duration: Idiopathic Parkinson's disease, Mduration = 8.63 SD=5.09

Medication: on medication

Hoehn-Yahr: mean = 2.34, SD = 1

UPDRS-3: NA

Gender (male): 24 males (63%)

averaged ages (SD, range): M= 61.42 SD=8.52

other neurological disease (tumor, stroke, etc.): non-demented, MMSE score below 24 were excluded

other major psychopathology: NA

origin country (or ethnicity): Poland

method behavioral

instruments used in order to quantify the variables

Social cognition aspect: emotion recognition

Name of the task: Facial Emotion Recognition Task - EIS-F (Matczaket al., 2005)

type of stimulus [face/voice etc., Ekman faces/other etc.]: 18 color photographs, nine featuring male faces and nine featuring female faces.

task condition: four photographs depict positive emotions and five depict negative emotions. The emotions depicted in the photographs include both basic emotions (positive: joy, surprise; negative: sadness, anxiety, anger, disgust), and complex emotions (positive: tenderness, self-contentment, pride, satisfaction, admiration, hope, coquetry, composure, self-confidence, curiosity, expectation, interest, astonishment; negative: unpleasant surprise, confusion, aversion, distrust, resignation, regret, disappointment, insecurity, disregard, feeling of superiority, indignation, envy, hate, contempt, unease, jealousy, disbelief).

operationalization: The subject must determine which of the six emotions are shown in each photograph, and which are not, by choosing one of three possible responses: "shown," "not shown," and "hard to say." Perfect score in the test requires the identification of 45 "shown" emotions and correct rejection of 63 "not shown" emotions.

Main findings related to the review's scope

The hit rate was considerably higher in the PD group than in the HC group (Table 2), although this was accompanied by a higher rate of false alarm responses. Despite the higher rate of hits, the d' sensitivity index (which indicates the accuracy of recognition) showed no difference between the groups. For both

groups, a large number of hits were accompanied by an equally large number of false alarms. Both groups employed a liberal response strategy (as indicated by a negative c index). At the same time, the response bias was significantly higher (i.e., larger deviation from zero) among PD patients, which shows that there is a greater tendency to give positive responses in this group.

All ROC curves lie relatively close to the diagonal dotted line representing performance of random choice strategy. This suggests that the difficulty level of the task was relatively high. However, all groups perform above chance level. Note that younger HCs performed better than older HCs. This would indicate that the age of test subjects significantly affects facial emotion recognition ability.

Tags: Emotion recognition, behavioral

Emotional processing in parkinson's disease: A systematic review

Item Type Journal Article
Author Julie Peron
Author Thibaut Dondaine
Author Florence Le Jeune
Author Didier Grandjean
Author Marc Verin
Abstract Parkinson's disease provides a useful model for studying the neural substrates of emotional processing. The striato-thalamo-cortical circuits, like the mesolimbic dopamine system that modulates their function, are thought to be involved in emotional processing. As Parkinson's disease is histopathologically characterized by the selective, progressive, and chronic degeneration of the nigrostriatal and mesocorticolimbic dopamine systems, it can therefore serve as a model for assessing the functional role of these circuits in humans. In the present review, we begin by providing a synopsis of the emotional disturbances observed in Parkinson's disease. We then discuss the functional roles of the striato-thalamo-cortical and mesolimbic circuits, ending with the conclusion that both these pathways are indeed involved in emotional processing. (C) 2012 Movement Disorder Society
Date 2012-02
Language English
Extra Place: 111 RIVER ST, HOBOKEN 07030-5774, NJ USA Type: Review
Volume 27
Publisher WILEY
Pages 186-199
Publication MOVEMENT DISORDERS
DOI 10.1002/mds.24025
Issue 2
ISSN 0885-3185
Date Added 14.7.2025, 14:50:40
Modified 5.9.2025, 14:50:21

Notes:

Included: Systematic Review

Search Criteria

The databases were selected using PubMed services with the following keywords: Parkinson's disease, emotion, facial expression, emotional prosody, subjective feeling, arousal.

Forty-three articles were identified as being relevant to the question of emotional processing in PD

Results

Whereas researchers have fairly consistently reported a deficit in the recognition of emotion conveyed by the human voice (ie, emotional prosody) in PD,19 studies of emotional facial expression (EFE) recognition have yielded some particularly ambivalent results. Whereas some authors have reported diminished EFE recognition in Parkinsonian individuals compared with HC,19, 24, 30 others have failed to demonstrate any difference at all between the two.17, 22, 25, 32, 33

Tags: emotion recognition, behavioral

Emotional, cognitive and neurochemical alterations in a premotor stage model of Parkinson's disease.

Item Type Journal Article

Author M. T. Tadaiesky
Author P. A. Dombrowski
Author C. P. Figueiredo
Author E. Cargnin-Ferreira
Author C. Da Cunha
Author R. N. Takahashi

Abstract In addition to classic motor symptoms, Parkinson's disease (PD) is characterized by cognitive and emotional deficits, which have been demonstrated to precede motor impairments. The present study addresses the question of whether a partial degeneration of dopaminergic neurons using 6-hydroxydopamine (6-OHDA) in rats is able to induce premotor behavioral signs. The time-course of nigrostriatal damage was evaluated by tyrosine hydroxylase immunohistochemistry and the levels of dopamine, noradrenaline, and 5-HT in various brain regions were analyzed by high performance liquid chromatography (HPLC). Behavioral tests that assessed a variety of psychological functions, including locomotor activity, emotional reactivity and depression, anxiety and memory were conducted on 6-OHDA lesioned rats. Bilateral infusion of 6-OHDA in the striatum of rats caused early (1 week) damage of dopaminergic terminals in striatum and in cell bodies in substantia nigra pars compacta. The nigrostriatal lesion was accompanied by early loss of dopamine in the striatum, which remained stable through a 3-week period of observation. In addition, a late (3 weeks) loss of dopamine in the prefrontal cortex, but not in the hippocampus, was seen. Additional noradrenergic and serotonergic alterations were observed after 6-OHDA administration. The results indicated that 6-OHDA lesioned rats show decreased sucrose consumption and an increased immobility time in the forced swimming test, an anhedonic-depressive-like effect. In addition, an anxiogenic-like activity in the elevated plus maze test and cognitive impairments were observed on the cued version of the Morris water maze and social recognition tests. These findings suggest that partial striatal dopaminergic degeneration and

parallel dopaminergic, noradrenergic and serotonergic alterations in striatum and prefrontal cortex may have caused the emotional and cognitive deficits observed in this rat model of early phase PD.

**Date**	2008 Oct 28
**Language**	eng
**Extra**	Place: United States
**Volume**	156
**Pages**	830-840
**Publication**	Neuroscience
**DOI**	10.1016/j.neuroscience.2008.08.035
**Issue**	4
**Journal Abbr**	Neuroscience
**ISSN**	0306-4522
**PMID**	18817851
**Date Added**	6.7.2025, 19:09:38
**Modified**	5.9.2025, 14:58:12

Notes:

Not Included: Does not Study SC

Tags: EXCLUDED

Empathy and theory of mind in Parkinson's disease: A meta-analysis.

**Item Type**	Journal Article
**Author**	Sarah P. Coundouris
**Author**	Alexandra G. Adams
**Author**	Julie D. Henry
**Abstract**	In contrast to well-documented deficits in the core social cognitive domains of social perception and theory of mind (ToM), how Parkinson's disease (PD) affects one's empathic capacity remains poorly understood. The current study provides the first meta-analytic review of both ToM and empathy as broad constructs, and also breaks these constructs down to clearly differentiate their overlapping (affective ToM and cognitive empathy) and distinct (affective empathy and cognitive ToM) components. A total of 38 studies contributed to these analyses, with results revealing that, relative to controls, PD is associated with significant and substantial deficits in the domain of cognitive ToM (g = -0.78), as well as the overlapping domains of affective ToM/ cognitive empathy (g = -0.69). However, no group differences were identified for affective empathy (g = -0.08). These data speak to there being a potential preservation of affective empathic processing in PD, but because of the relatively limited research base on this topic, recommendations for future research are highlighted.
**Date**	2020 Feb
**Language**	eng
**License**	Copyright © 2019 Elsevier Ltd. All rights reserved.
**Extra**	Place: United States
**Volume**	109

Pages 92-102
Publication Neuroscience and biobehavioral reviews
DOI 10.1016/j.neubiorev.2019.12.030
Journal Abbr Neurosci Biobehav Rev
ISSN 1873-7528 0149-7634
PMID 31899300
Date Added 6.7.2025, 19:09:39
Modified 5.9.2025, 14:31:47

Notes:

Included – meta-analysis

sample characteristics

The meta-analysis was conducted in line with PRISMA guidelines (Moher et al., 2009). A systematic literature search of electronic databases (PsycINFO, MEDLINE, PubMed, Web of Science) was completed in May 2019. The terms searched were: [redacted], social perception, emotion perception, emotion recognition, facial expression*, prosody, [redacted], mentalising, pragmatic impairment, non-literal language, sarcas*, lie*, joke*, empath*, perspective taking, Frontal Systems [redacted] Scale, Frontal Behavioral Inventory, Socioemotional Dysfunction Scale, Peer-Report Social Functioning Scale, Social Impairment Rating Scale, Iowa Scales of Personality Change, Frontal Lobe Personality Scale; in combination with, hypokinetic [redacted], Parkinson's disease, [redacted], Parkinson

N = 38

To ensure that the studies had comparable outcomes, the following criteria were applied:

· 1)

For empathy, tasks were considered eligible if they assessed one's emotional response to the perceived situation of another (affective empathy) and/or assessed a participant's understanding of another's emotional state (i.e. what another was feeling; cognitive empathy; Bartochowski et al., 2018; Henry et al., 2016; Wondra and Ellsworth, 2015).

· 2)

For ToM, studies that assessed a participant's understanding of another's emotional state also contributed to analyses as affective ToM (Preckel et al., 2018; Shamay-Tsoory et al., 2009, 2005). Tasks involving the attribution of mental states to another were included as measures of cognitive ToM (Dvash and Shamay-Tsoory, 2014; Shamay-Tsoory et al., 2007).

Thirty-eight studies published between 2000 and 2019 met the inclusion criteria, and in total, data from 1014 PwPD, and 921 controls contributed to analyses. Participants were broadly equivalent in terms of age (M_{PD} = 65.09, SD_{PD} = 4.28, *n* = 38, and $M_{Control}$ = 64.57, $SD_{Control}$ = 4.89, *n* = 36). The mean disease duration of PwPD was 7.08 years (*SD* = 2.49, *n* = 30), while the mean motor severity was 1.91 (*SD* = 0.54. *n* = 23) using Hoehn and Yahr's (1967) scale, and 21.83 (*SD* = 6.80, *n* = 20) with the UPDRS. PD participants' average MMSE score was 28.32 (*SD* = 0.67, *n* = 21).

Main findings related to the review's scope

Highlights

•People with Parkinson's disease show comparable difficulties in understanding others' mental states and emotional experiences.

•Deficits in cognitive empathy/affective theory of mind only evident for performance based tasks.

•Preliminary support for preserved affective empathy in people with Parkinson’s disease.

relative to controls, PwPD exhibit significant and moderate sized difficulties understanding others’ mental states (cognitive ToM), and others’ emotional experiences (affective ToM/cognitive empathy). However, the current study also provides preliminary support for the possibility that the ability to resonate affectively with others (affective empathy) is preserved, pointing to a potentially important disconnect between the affective and cognitive components of empathic responding in this group.

Tags: empathy, ToM, behavioral

Empathy changes in neurocognitive disorders: A review.

Item Type Journal Article

Author Zachary Bartochowski
Author Shravan Gatla
Author Rita Khoury
Author Roula Al-Dahhak
Author George T. Grossberg

Abstract BACKGROUND: Empathy can be broadly defined as the ability to understand what others feel (cognitive empathy) and feel what others feel (affective empathy). The capacity to empathize may be impaired in certain major neurocognitive disorders (MNCDs), affecting not only the patient, but also the caregivers. METHODS: PubMed and Google Scholar databases were searched for studies investigating empathy changes, using an objective scale, in patients with MNCDs. RESULTS: The Interpersonal Reactivity Index was most commonly used to evaluate empathy in this population. Impairments in cognitive but not affective empathy were found in patients with Alzheimer's disease (AD), and may be attributable to overall cognitive decline. Patients with frontotemporal dementia (FTD) have demonstrated severe deficits in empathy, correlating with greater caregiver burden. Empathy changes in patients with dementia with Lewy bodies, vascular dementia, and Parkinson's disease dementia have not yet been studied. Intranasal oxytocin has emerged as a promising therapeutic approach for empathy loss, but it has not been explored yet in patients with MNCDs. CONCLUSIONS: Caregivers need to be educated about empathy loss, which is an important part of the disease process in AD and FTD. Future research should further assess empathy changes in other MNCDs, as well as explore novel treatment options in this field.

Date 2018 Aug
Language eng
Volume 30
Pages 220-232
Publication Annals of clinical psychiatry :, official journal of the American Academy of Clinical Psychiatrists..
Issue 3
Journal Abbr Ann Clin Psychiatry

ISSN 1547-3325 1040-1237
PMID 30028897
Date Added 6.7.2025, 19:09:40
Modified 5.9.2025, 14:27:45

Notes:

Not Included: not a systematic review
Tags: EXCLUDED

Empathy design method based on immersive interactive experiential spaces: A case study of Parkinson's disease in China

Item Type Journal Article
Author Xinyu Yang
Author Jianfang Guan
Author Dongjun Han
Author Xipei Ren
Date 2024-11-01
Volume 27
Pages 1270-1291
Publication DESIGN JOURNAL
DOI 10.1080/14606925.2024.2405775
Issue 6, SI
ISSN 1460-6925
Date Added 14.7.2025, 14:48:38
Modified 5.9.2025, 15:01:23

Notes:

Not Included: Case-Study
Tags: EXCLUDED

Empathy: A Review of the Concept

Item Type Journal Article
Author Benjamin M.P. Cuff
Author Sarah J. Brown
Author Laura Taylor
Author Douglas J. Howat
Abstract The inconsistent definition of empathy has had a negative impact on both research and practice. The aim of this article is to review and critically appraise a range of

definitions of empathy and, through considered analysis, to develop a new conceptualisation. From the examination of 43 discrete definitions, 8 themes relating to the nature of empathy emerged: “distinguishing empathy from other concepts”; “cognitive or affective?”; “congruent or incongruent?”; “subject to other stimuli?”; “self/other distinction or merging?”; “trait or state influences?”; “has a behavioural outcome?”; and “automatic or controlled?” The relevance and validity of each theme is assessed and a new conceptualisation of empathy is offered. The benefits of employing a more consistent and complete definition of empathy are discussed.

Date 04/2016
Language en
Short Title Empathy
Library Catalog DOI.org (Crossref)
URL https://journals.sagepub.com/doi/10.1177/1754073914558466
Accessed 19.1.2026, 7:28:32
Volume 8
Pages 144-153
Publication Emotion Review
DOI 10.1177/1754073914558466
Issue 2
Journal Abbr Emotion Review
ISSN 1754-0739, 1754-0747
Date Added 19.1.2026, 7:28:32
Modified 19.1.2026, 7:28:32

Executive dysfunction in Parkinson's disease: A review

Item Type Journal Article
Author Georg Dirnberger
Author Marjan Jahanshahi
Abstract Executive dysfunction can be present from the early stages of Parkinson's disease (PD). It is characterized by deficits in internal control of attention, set shifting, planning, inhibitory control, dual task performance, and on a range of decision-making and social cognition tasks. Treatment with dopaminergic medication has variable effects on executive deficits, improving some, leaving some unchanged, and worsening others. In this review, we start by defining the specific nature of executive dysfunction in PD and describe suitable neuropsychological tests. We then discuss how executive deficits relate to pathology in specific territories of the basal ganglia, consider the impact of dopaminergic treatment on executive function (EF) in this context, and review the changes in EFs with disease progression. In later sections, we summarize correlates of executive dysfunction in PD with motor performance (e.g., postural instability, freezing of gait) and a variety of psychiatric (e.g., depression, apathy) and other clinical symptoms, and finally discuss the implications of these for the patients' daily life.
Date 2013-09
Language English
Extra Place: 111 RIVER ST, HOBOKEN 07030-5774, NJ USA Type: Review
Volume 7

Publisher WILEY
Pages 193-224
Publication JOURNAL OF NEUROPSYCHOLOGY
DOI 10.1111/jnp.12028
Issue 2, SI
ISSN 1748-6645
Date Added 14.7.2025, 14:50:38
Modified 5.9.2025, 14:33:52

Notes:

Not Included: not a systematic review
Tags: EXCLUDED

Executive dysfunction in Parkinson's disease: From neurochemistry to circuits, genetics and neuroimaging

Item Type Journal Article
Author Shuyan Tong
Author Ruiwen Wang
Author Huihua Li
Author Zhu Tong
Author Deqin Geng
Author Xiangrong Zhang
Author Chao Ren
Abstract Cognitive decline is one of the most significant non-motor symptoms of Parkinson's disease (PD), with executive dysfunction (EDF) being the most prominent characteristic of PD-associated cognitive deficits. Currently, lack of uniformity in the conceptualization and assessment scales for executive functions impedes the early and accurate diagnosis of EDF in PD. The neurobiological mechanisms of EDF in PD remain poorly understood. Moreover, the treatment of cognitive impairment in PD has progressed slowly and with limited efficacy. Thus, this review explores the characteristics and potential mechanisms of EDF in PD from multiple perspectives, including the concept of executive function, commonly used neuropsychological tests, neurobiochemistry, genetics, electroencephalographic activity and neuroimaging. The available evidence indicates that degeneration of the frontal-striatal circuit, along with mutations in the Catechol-O-methyltransferase (COMT) gene and Leucine-rich repeat kinase 2 (LRRK2) gene, may contribute to EDF in patients with PD. The increase in theta and delta waves, along with the decrease in alpha waves, offers potential biomarkers for the early identification and monitoring of EDF, as well as the development of dementia in patients with PD. The PD cognition-related pattern (PDCP) pattern may serve as a tool for monitoring and assessing cognitive function progression in these patients and is anticipated to become a biomarker for cognitive disorders associated with PD. The aim is to provide new insights for the early and precise diagnosis and treatment of EDF in PD.
Date 2025
URL https://www.sciencedirect.com/science/article/pii/S0278584625000260

Volume 137
Pages 111272
Publication Progress in Neuro-Psychopharmacology and Biological Psychiatry
DOI https://doi.org/10.1016/j.pnpbp.2025.111272
ISSN 0278-5846
Date Added 6.7.2025, 19:12:36
Modified 5.9.2025, 14:58:37

Notes:

Not Included: not on SC
Tags: EXCLUDED

Eye-gaze Strategies During Facial Emotion Recognition in Neurodegenerative Diseases and Links With Neuropsychiatric Disorders

Item Type Journal Article
Author Kevin Polet
Author Solange Hesse
Author Adeline Morisot
Author Benoit Kullmann
Author Sandrine Louchart de la Chapelle
Author Alain Pesce
Author Galina Iakimova
Abstract Background: Facial emotion recognition (FER) is primarily and severely impaired in individuals with the behavioral variant of frontotemporal dementia (bvFTD) and is often mildy impaired in individuals with Alzheimer disease (AD) or Parkinson disease (PD). Such impairment is associated with inappropriate social behaviors. Objective: To determine whether FER impairment is linked to the use of inappropriate eye-gaze strategies to decode facial emotions, leading to misinterpretation of others' intentions and then to behavioral disorders. Method: We assessed FER in 9 individuals with bvFTD, 23 with AD, and 20 with PD, as well as 22 healthy controls (HC), using the Reading the Mind in the Eyes (RME) Test and the Ekman Faces Test. Eye movements (number and duration of fixations) were recorded with an eye-tracking device. Behavior was assessed using the Neuropsychiatric Inventory. Results: FER was mildly impaired in the AD and PD groups and severely impaired in the bvFTD group. FER impairment was accompanied by an increase in the number of fixations and a more attracted gaze toward the lower part of one's face. FER impairment and an increase in the number of fixations were positively correlated with behavioral disorders. Conclusion: Our study demonstrated a link between FER impairment, modification of eye-gaze strategies during the observation of emotional faces, and behavioral disorders in individuals with bvFTD and those with AD or PD. These results suggest that an eye-gaze strategy rehabilitation program could have beneficial effects on emotion recognition and behavioral disorders in individuals with these diseases.
Date 2022-03
Language English

Extra Place: TWO COMMERCE SQ, 2001 MARKET ST, PHILADELPHIA, PA 19103 USA Type: Article
Volume 35
Publisher LIPPINCOTT WILLIAMS & WILKINS
Pages 14-31
Publication COGNITIVE AND BEHAVIORAL NEUROLOGY
DOI 10.1097/WNN.0000000000000288
Issue 1
ISSN 1543-3633
Date Added 14.7.2025, 14:50:29
Modified 5.9.2025, 14:51:38

Notes:

Not included: no access

Tags: EXCLUDED

Face-to-trait inferences in patients with Parkinson's disease

Item Type Journal Article
Author Masahiro Hirai
Author Takeshi Sakurada
Author Shin-ichi Muramatsu
Abstract Introduction: Parkinson's disease is a progressive neurological disorder characterized by the preferential loss of dopaminergic neurons in the substantia nigra, which project to the striatum. The disease is characterized by prominent motor symptoms, which are its cardinal features. Consequently, Parkinson's disease has been primarily considered a disorder of movement. However, increasing evidence has indicated that Parkinson's disease affects not only the motor domain but also the cognitive domain. Increasing evidence indicates that patients with Parkinson's disease have an impaired ability to recognize emotional facial expressions. Recent studies have reported that other socially relevant information from faces, including face-to-trait inferences for traits such as dominance, competence, and trustworthiness, may be processed in subcortical regions, including the amygdala and caudate nucleus. However, the mechanism underlying the processing of face-to-trait inferences for these traits in patients with Parkinson's disease is still unknown. This study aimed to assess the face-to-trait inference ability in patients with Parkinson's disease. Method: Face-to-trait inference ability was assessed using a forced-choice method in patients with Parkinson's disease and age- and sex-matched healthy controls. Results: Overall correct face-to-trait inferences occurred significantly less frequently in the Parkinson's disease group than in the control group. Further analysis revealed a significant interaction between groups and the extent to which facial features were exaggerated. Conclusions: The present results suggest that the sensitivity of face-to-trait processing was linear in the Parkinson's disease group but not in the healthy controls. These deficits may have resulted from dysfunction in subcortical regions, which may also lead to impairment in other social inferential abilities in patients with Parkinson's disease.
Date 2019-02-07

**Language**	English
**Extra**	Place: 530 WALNUT STREET, STE 850, PHILADELPHIA, PA 19106 USA Type: Article
**Volume**	41
**Publisher**	TAYLOR & FRANCIS INC
**Pages**	170-178
**Publication**	JOURNAL OF CLINICAL AND EXPERIMENTAL NEUROPSYCHOLOGY
**DOI**	10.1080/13803395.2018.1513452
**Issue**	2
**ISSN**	1380-3395
**Date Added**	14.7.2025, 14:50:33
**Modified**	5.9.2025, 14:39:19

Notes:

Included

sample characteristics

size: 24 PD and 24 HC (age (within 3 years), sex, and hand dominance matched with patients with PD).

Parkinson's Disease type and duration: Mduration = 6.4 SD= 5.0

Medication: on medication.

Hoehn-Yahr: M=2.7 SD= 0.7

UPDRS-3: M= 24.4 SD= 11.8

Gender (male): 7 males (29%)

averaged ages (SD, range): M= 67.8 SD= 6.5

other neurological disease (tumor, stroke, etc.): None exhibited atypical symptoms, such as severe gaze palsy or symptomatic dysautonomia. None showed atrophy of the putamen or pons on magnetic resonance imaging.

other major psychopathology: NA

origin country (or ethnicity): Japan

method observational

instruments used in order to quantify the variables

Social cognition aspect: emotion recognition

Name of the task: NA

type of stimulus [face/voice etc., Ekman faces/other etc.]: computer-generated faces that had been selected to have high or low perceived trust worthiness, dominance, or competence. These extensively validated faces were generated using FaceGen (https:// facegen.com) based on data-driven computational models of the respective traits (Todorov, Dotsch, Porter, Oosterhof, & Falvello, 2013; Todorov & Oosterhof, 2011; Todorov, Said, Engell, & Oosterhof, 2008). Herein, we used three sets of faces, each of which included 24 distinct face identities.

task condition: dominant/competent/trustworthy, number of std of a stimuli from the standard

operationalization: accuracy

Main findings related to the review's scope

Beyond group, Performance was significantly better under the dominance condition than under the competence condition. differences in performance levels between the trustworthiness and dominance conditions and between the trustworthiness and competence conditions were not significant.

Performance was significantly better in the HC group than in the PD group

Beyond group, it was most easy to identify 3sd than 2sd than 1sd. This sig trend was also within the PD group, however not the HC (which was simillar in 3sd and 2sd).

Tags: Emotion recognition, behavioral

Facial emotion decoding in patients with Parkinson's disease.

**Item Type**	Journal Article
**Author**	Marco De Risi
**Author**	Giancarlo Di Gennaro
**Author**	Angelo Picardi
**Author**	Sara Casciato
**Author**	Liliana G. Grammaldo
**Author**	Alfredo D'Aniello
**Author**	Deborah Lanni
**Author**	Stefano Meletti
**Author**	Nicola Modugno
**Abstract**	PURPOSE: In line with the growing attention on non-motor symptoms and disturbance of affective and emotional processing in Parkinson's disease, we aimed to study the different aspects of facial emotion expression evaluation in a group of Parkinson's disease without cognitive decline in treatment with common antiparkinsonian drugs, matched for sex, age and education with healthy subjects. MATERIALS AND METHODS: The study was conducted on 30 patients (13 male; mean age: 63.3 ± 6.7; mean age of disease onset: 56.5 ± 7.1; mean duration of the disease: 6.7 ± 2.6) with a diagnosis of Parkinson's disease and receiving dopaminergic therapy, as compared with 30 healthy controls. Different tasks of facial expression evaluation were used. All patients were assessed for neuropsychological and psychological profiles during optimized medication-on condition. RESULTS: The total number of errors in facial emotion recognition task is higher ($p < 0.001$) in patients than controls and it is due to errors in identifying sadness ($p < 0.001$), anger ($p = 0.01$) and fear ($p < 0.001$). No differences in the total amount of activation, valence and intensity ratings were found. The difference between patients and controls in emotion recognition appears to be independent by the severity of depressive symptoms. CONCLUSIONS: The present study provides further evidence of altered non-verbal emotional information processing in Parkinson's disease patients, suggesting that nigrostriatal dopaminergic depletion leads also to emotional information processing dysfunction. The consequences of these emotional encoding disturbances in daily living and their relationship to mood and behavioural disorders remain to be clarified.
**Date**	2018 Jan
**Language**	eng
**Extra**	Place: England
**Volume**	128
**Pages**	71-78
**Publication**	The International journal of neuroscience
**DOI**	10.1080/00207454.2017.1366475
**Issue**	1
**Journal Abbr**	Int J Neurosci
**ISSN**	1563-5279 0020-7454
**PMID**	28796560

**Date Added**	6.7.2025, 19:09:37
**Modified**	5.9.2025, 14:32:40

Notes:

Included

sample characteristics

size: 30 PD and 30 HC (matched for sex, age range and education.)

Parkinson's Disease type and duration: idiopathic PD, Mduration = 6.7 SD= 2.6

Medication: on medication

Hoehn-Yahr: M= 1.6 SD= 0.5

UPDRS-3: M= 23.7 SD= 11.9

Gender (male): 13 males (43%)

averaged ages (SD, range): M= 63.3 SD=6.7

other neurological disease (tumor, stroke, etc.): None

other major psychopathology: None

origin country (or ethnicity): Italy

method (Review, meta-analysis or observational and/or self-reported):

instruments used in order to quantify the variables

Social cognition aspect: emotion recognition

Name of the task: NA

type of stimulus [face/voice etc., Ekman faces/other etc.]: facial affect taken from the Ekman and Friesen series

task condition: happiness, fear, sadness, disgust and anger

operationalization: emotion-labelling task, and to rate the intensity, the arousal and the valence of facial expressions.

Main findings related to the review's scope

PD patients displayed a significantly worse performance in ER task with higher total and specific emotions errors, compared to HC

Specifically, errors in identifying sadness ($p < 0.001$), anger ($p = 0.01$) and fear ($p < 0.001$

There were not significant differences between the two groups in the total amount of activation, valence and intensity.

Tags: Emotion recognition, behavioral

Facial Emotion Recognition and Discrimination Deficit in Idiopatic Parkinson Patients

**Item Type**	Journal Article
**Author**	Ersin Kasim Ulusoy
**Author**	Emre Ayar
**Author**	Deniz Bayindirli
**Abstract**	Objective: Motor symptoms are the primary focus in diagnosis and treatment of idiopathic Parkinson disease (IPD). But facial emotion recognition disorder, one of non-motor symptoms of the disease, reduces quality of life significantly by disrupting social interaction. Facial emotion recognition and discrimination ability is an important part of social interaction. Neuroimaging studies highlight amigdala as the locus of facial emotion recognition disorder in IPD. The aim of this study is to investigate the relationship between clinical features and impairments in facial emotion recognition and discrimination ability. Materials and Methods: This study involves 41 patients followed with IPD in neurology outpatient clinic and 38 healthy controls. Facial Emotion Identification Test (FEIT) and Facial Emotion Discrimination Test (FEDT) were carried out for both groups. Clinical and demographic features of patient and control groups were recorded. Hoehn-Yahr (H and Y) scale was used for staging of disease and Unified Parkinson's Disease Rating Scale (UPDRS) was used for assessment of clinical severity. The results of both groups were compared with Kruskal Wallis and Pearson's Chi Square tests. Results: Average of FEIT and FEDT in patients with IPD are 12.64 +/- 5.55 and 17.84 +/- 4.94, respectively. When these values were compared with control group, they were worse than control group (p<0.01). This impairment was correlated with H and Y and UPDRS stages. The most impaired one among facial exogenous sensations was fear sensation with 2.29 +/- 1.26. Conclusion: This study shows that patients with IPD have more difficulty than normal population in recognition and discrimination of facial exogenous emotions. This difficulty was correlated with stage and clinical severity of disease. We hope that these findings will be an important step in regulation of impaired social intercourse and functionality in IPD and will help determining rehabilitation targets.
**Date**	2015-03
**Language**	English
**Extra**	Place: MESRUTIYET CADDESI 48-7, ANKARA, 06650, TURKEY Type: Article
**Volume**	21
**Publisher**	TURKISH NEUROLOGICAL SOC
**Pages**	16-21
**Publication**	TURKISH JOURNAL OF NEUROLOGY
**DOI**	10.4274/tnd.44227
**Issue**	1
**ISSN**	1301-062X
**Date Added**	14.7.2025, 14:50:37
**Modified**	5.9.2025, 14:59:11

Notes:

Included

Sample characteristics

Size: 41 PD; 38 HC (age and sex matched)

PD-type: idiopathic

PD-duration: M=6.51 SD=4.11 years

Medication: NA

Hoehn-Yahr: M=2.22 SD=0.85 (1-4)

UPDRS-3: M=19.12 SD=8.38

Gender (male): 26 (63%)

Age: M=69.92 SD=8.08

Other neurological disease (tumor, stroke, etc.): none

Other major psychopathology: none

Origin country (or ethnicity): Turkey

method observational

instruments used in order to quantify the variables

Social cognition aspect: Emotion Recognition

Name of the task: Facial Emotion Identification Test (FEIT)

Type of stimulus [face/voice etc., Ekman faces/other etc.]: 19 black and white photos showing different emotional facial expressions.

Task condition: Photos convey six main emotions (joy, sadness, anger, fear, confusion, shame)

Operationalization: The subject is given an answer sheet containing 6 options for each of the 19 trials. The subject is asked to mark the most appropriate emotion that corresponds to each image >> sum correct answers

Social cognition aspect: emotion recognition.

Name of the task: Facial emotion Discrimination Test (FEDT)

Type of stimulus [face/voice etc., Ekman faces/other etc.]: 30 black and white photo pairs

Task condition: six main emotions (joy, sadness, anger, fear, confusion, shame).

Operationalization: Photo pairs show either the same or different emotions. >> Number of correct answers

Main findings related to the review's scope

In both tasks, PD were less accurate then HC.

When the patient group was compared to the control group in terms of the correct answers they gave for facial emotion recognition (happiness, anger, fear, etc.), performance for all emotions was statistically lower. Among these emotions, the most impaired one was fear

Tags: emotion recognition, behavioral

Facial Emotion Recognition and Expression in Parkinson's Disease: An Emotional Mirror Mechanism?

Item Type Journal Article
Author Lucia Ricciardi
Author Federica Visco-Comandini
Author Roberto Erro
Author Francesca Morgante
Author Matteo Bologna
Author Alfonso Fasano
Author Diego Ricciardi
Author Mark J. Edwards
Author James Kilner

Abstract BACKGROUND AND AIM: Parkinson's disease (PD) patients have impairment of facial expressivity (hypomimia) and difficulties in interpreting the emotional facial expressions produced by others, especially for aversive emotions. We aimed to evaluate the ability to produce facial emotional expressions and to recognize facial emotional expressions produced by others in a group of PD patients and a group of healthy participants in order to explore the relationship between these two abilities and any differences between the two groups of participants. METHODS: Twenty non-demented, non-depressed PD patients and twenty healthy participants (HC) matched for demographic characteristics were studied. The ability of recognizing emotional facial expressions was assessed with the Ekman 60-faces test (Emotion recognition task). Participants were video-recorded while posing facial expressions of 6 primary emotions (happiness, sadness, surprise, disgust, fear and anger). The most expressive pictures for each emotion were derived from the videos. Ten healthy raters were asked to look at the pictures displayed on a computer-screen in pseudo-random fashion and to identify the emotional label in a six-forced-choice response format (Emotion expressivity task). Reaction time (RT) and accuracy of responses were recorded. At the end of each trial the participant was asked to rate his/her confidence in his/her perceived accuracy of response. RESULTS: For emotion recognition, PD reported lower score than HC for Ekman total score ($p<0.001$), and for single emotions sub-scores happiness, fear, anger, sadness ($p<0.01$) and surprise ($p = 0.02$). In the facial emotion expressivity task, PD and HC significantly differed in the total score ($p = 0.05$) and in the sub-scores for happiness, sadness, anger (all $p<0.001$). RT and the level of confidence showed significant differences between PD and HC for the same emotions. There was a significant positive correlation between the emotion facial recognition and expressivity in both groups; the correlation was even stronger when ranking emotions from the best recognized to the worst ($R = 0.75$, $p = 0.004$). CONCLUSIONS: PD patients showed difficulties in recognizing emotional facial expressions produced by others and in posing facial emotional expressions compared to healthy subjects. The linear correlation between recognition and expression in both experimental groups suggests that the two mechanisms share a common system, which could be deteriorated in patients with PD. These results open new clinical and

	rehabilitation perspectives.
**Date**	2017
**Language**	eng
**Extra**	Place: United States
**Volume**	12
**Pages**	e0169110
**Publication**	PloS one
**DOI**	10.1371/journal.pone.0169110
**Issue**	1
**Journal Abbr**	PLoS One
**ISSN**	1932-6203
**PMID**	28068393
**PMCID**	PMC5221788
**Date Added**	6.7.2025, 19:09:34
**Modified**	5.9.2025, 14:52:42

Notes:

Included

Sample characteristics

Size: 20 PD, 20 HC (matched for age, gende)

PD-type: NA

PD-duration: M = 7.3, SD = 4.1

Medication: Assessment in ON

Hoehn-Yahr: NA

UPDRS-3: M = 21.8, SD = 8.7

Gender (male): 8 (40 %)

Age: M = 69.3, SD = 6.6

Other neurological disease (tumor, stroke, etc.): none

Other major psychopathology: none

Origin country (or ethnicity): NA

method behavioral

instruments used in order to quantify the variables

Social cognition aspect: Facial emotion recognition

Name of the task: NA

Type of stimulus [face/voice etc., Ekman faces/other etc.]: 60 Ekman faces (happy, sad, surprise, angry, disgust, fear)

Task condition: sex-forced-choice

Operationalization: Correct answers. maximum of 60 correct for recognition of all six emotions, or scores out of 10 for recognition of each basic emotion

---

Main findings related to the review's scope

Post-hoc t-tests revealed a statistical difference for happiness, anger, sadness, surprise and fear ($p<0.05$), which were less recognized by the PD group

Tags: Emotion recognition, behavioral

Facial emotion recognition and its relationship to cognition and depressive symptoms in patients with Parkinson's disease.

Item Type Journal Article

Author J. Pietschnig
Author L. Schröder
Author I. Ratheiser
Author I. Kryspin-Exner
Author M. Pflüger
Author D. Moser
Author E. Auff
Author W. Pirker
Author G. Pusswald
Author J. Lehrner
Abstract BACKGROUND: Impairments in facial emotion recognition (FER) have been detected in patients with Parkinson disease (PD). Presently, we aim at assessing differences in emotion recognition performance in PD patient groups with and without mild forms of cognitive impairment (MCI) compared to healthy controls. METHODS: Performance on a concise emotion recognition test battery (VERT-K) of three groups of 97 PD patients was compared with an age-equivalent sample of 168 healthy controls. Patients were categorized into groups according to two well-established classifications of MCI according to Petersen's (cognitively intact vs. amnestic MCI, aMCI, vs. non-amnestic MCI, non-aMCI) and Litvan's (cognitively intact vs. single-domain MCI, sMCI, vs. multi-domain MCI, mMCI) criteria. Patients and controls underwent individual assessments using a comprehensive neuropsychological test battery examining attention, executive functioning, language, and memory (Neuropsychological Test Battery Vienna, NTBV), the Beck Depression Inventory, and a measure of premorbid IQ (WST). RESULTS: Cognitively intact PD patients and patients with MCI in PD (PD-MCI) showed significantly worse emotion recognition performance when compared to healthy controls. Between-groups effect sizes were substantial, showing non-trivial effects in all comparisons (Cohen's ds from 0.31 to 1.22). Moreover, emotion recognition performance was higher in women, positively associated with premorbid IQ and negatively associated with age. Depressive symptoms were not related to FER. CONCLUSIONS: The present investigation yields further evidence for impaired FER in PD. Interestingly, our data suggest FER deficits even in cognitively intact PD patients indicating FER dysfunction prior to the development of overt cognitive dysfunction. Age showed a negative association whereas IQ showed a positive association with FER.
Date 2016 Jul
Language eng
Extra Place: United States
Volume 28
Pages 1165-1179
Publication International psychogeriatrics
DOI 10.1017/S104161021600034X
Issue 7
Journal Abbr Int Psychogeriatr
ISSN 1741-203X 1041-6102
PMID 26987816
Date Added 6.7.2025, 19:09:35
Modified 5.9.2025, 14:51:11

Notes:

Included

Sample characteristics

Size: 26 PD-CI (no cognitive decliine), 57 PD-non-aMCI, 14 PD-aMCI

PD-type: NA

PD-duration: M = 6, Range = 0.25,-19 (only overall reported)

Medication: ON state

Hoehn-Yahr: NA

UPDRS-3: M = 25.1, SD = 12.4 (only overall reported)

Gender (male): PS-CI 18 males (68%); PD-non-aMCI: 52 males (91%); PD-aMCI: 9 males (61%)

Age: PD-CI M = 65.46; SD = 9.58; PD-non-aMCI M = 67.32, SD = 9.93; PD-aMCI M = 68.71, SD = 5.72

Other neurological disease (tumor, stroke, etc.): none

Other major psychopathology: none

Origin country (or ethnicity): Austria

method behavioural

instruments used in order to quantify the variables

Social cognition aspect: Facial emotion recognition (FER)

Name of the task: Vienna emotion recognition task – short form (VERT-K)

Type of stimulus [face/voice etc., Ekman faces/other etc.]: 36 colored pictures of faces

Task condition: either expressing anger, disgust, fear, happiness, sadness or neutral (6 each)

Operationalization: Select correct emotions>> Total number of correct responses

Main findings related to the review's scope

healthy controls outperformed patients in FER performance, yielding large significant effects when compared to non-aMCI and aMCI PD patients (Cohen ds = 0.84 and 1.22, respectively) and a medium-sized albeit non-significant effect when compared to cognitively intact PD patients (Cohen *d* = 0.58). Although differences between patient groups did not reach nominal significance, non-trivial small- to medium-sized effects were observed for all pairwise comparisons. Cognitively intact patients showed higher scores than both non-aMCI and aMCI patients (Cohen *d*s = 0.28 and 0.57, respectively) and non-aMCI patients scored higher than aMCI patients (Cohen *d* = 0.31).

Tags: emotion recognition, behavioral

Facial emotion recognition and judgment of affective scenes in Parkinson's disease.

Item Type Journal Article
Author Federico Salfi
Author Stefano Toro
Author Gennaro Saporito
Author Patrizia Sucapane
Author Massimo Marano
Author Gianluca Montaruli
Author Angelo Cacchio
Author Michele Ferrara
Author Francesca Pistoia
Abstract Emotional dysfunctions in Parkinson's disease (PD) remain a controversial issue. While previous investigations showed compromised recognition of expressive faces in PD, no studies evaluated potential deficits in recognizing the emotional valence of affective scenes. This study aimed to investigate both facial emotion recognition performance and the ability to judge affective scenes in PD patients. Forty PD patients (mean age ± SD: 64.50 ± 8.19 years; 27 men) and forty healthy subjects (64.95 ± 8.25 years; 27 men) were included. Exclusion criteria were previous psychiatric disorders, previous Deep Brain Stimulation, and cognitive impairment. Participants were evaluated through the Ekman 60-Faces test and the International Affective Picture System. The accuracy in recognizing the emotional valence of facial expressions and affective scenes was compared between groups using linear mixed models. Pearson's correlation was performed to test the association between accuracy measures. The groups did not differ in sex, age, education, and Mini-Mental State Examination scores. Patients showed a lower recognition accuracy of facial expressions (68.54 % ± 15.83 %) than healthy participants (78.67 % ± 12.04 %; $p < 0.001$). Specifically, the PD group was characterized by lower recognition of faces expressing fear, sadness, and anger than the control group (all $p < 0.020$). No difference was detected for faces expressing disgust, surprise, and happiness (all $p \geq 0.25$). Furthermore, patients showed lower accuracy in recognizing the emotional valence of affective scenes (66.75 % ± 14.59 %) than healthy subjects (74.83 % ± 12.65 %; $p = 0.010$). Pearson's correlations indicated that higher accuracy in recognizing the emotional facial expressions was associated with higher accuracy in classifying the valence of affective scenes in patients ($r = 0.57$, $p < 0.001$) and control participants ($r = 0.57$, $p < 0.001$). Our study suggested maladaptive affective processing in PD, leading patients to misinterpret both facial expressions and the emotional valence of complex evocative scenes.
Date 2024 Jun 30
Language eng
License © 2024 The Authors.
Extra Place: England
Volume 10
Pages e32947
Publication Heliyon
DOI 10.1016/j.heliyon.2024.e32947
Issue 12
Journal Abbr Heliyon

ISSN 2405-8440
PMID 38975139
PMCID PMC11226888
Date Added 6.7.2025, 19:09:34
Modified 14.7.2025, 15:19:31

Notes:

Included

Sample characteristics

Size: 40 PD, 40 HC (matched for age, sex, education)

PD-type: Idiopathic PD

PD-duration: M = 5.68, SD = 3.17

Medication: ON state

Hoehn-Yahr: Md = 1, Range = 1-3

UPDRS-3: M = 22.63, SD = 10.53

Gender (male): 27 (68%)

Age: M = 64.50, SD = 8.19, Range = 44-77

Other neurological disease (tumor, stroke, etc.): None

Other major psychopathology: None

Origin country (or ethnicity): Italy

method (Review, meta-analysis or observational and/or self-reported):

instruments used in order to quantify the variables

Social cognition aspect: Facial emotion recognition

Name of the task: Ekman 60-Faces task

Type of stimulus [face/voice etc., Ekman faces/other etc.]: 60 Ekman faces. Ten photographs were selected for each emotion (10 faces x 6 emotions) and presented, one at a time.

Task condition: (happiness, sasness, anger, fear, sigust, surprise)

Operationalization: Participants were required to name the expressed emotion for each stimulus by selecting one of six emotional labels. Then, they had to rate the intensity of the emotion expressed on a 1–9 Likert scale (1 = none, 5 = moderate, 9 = extreme) >> Correct answers and intensity rating

Main findings related to the review's scope

Facial expressions recognition

PD patients showed lower accuracy than healthy control participants.

The interaction effect between “experimental group” and “emotional valence” factors was also significant.

PD patients showed lower accuracy for faces expressing fear, sadness, and anger then the control group. No difference was detected for faces expressing disgust, surprise, and happiness.

Facial expressions intensity ratings

the analysis did not identify significant differences in the intensity ratings of recognized facial emotion.

Emotional responses to affective scenes

PD patients demonstrated lower accuracy (mean ± standard deviation, 66.75 % ± 14.59 %) than control participants (74.83 % ± 12.65 %), ($F_{1,78} = 7.01$, $p = 0.01$).

The main effect of "emotional valence" was also significant ($F_{4,2312} = 57.77$, $p < 0.001$). e. Holm post hoc comparisons indicated that all the emotions differed between them (all $p \leq 0.001$), except for the comparison between fear and disgust ($p = 0.59$).

The interaction effect between "experimental group" and "emotional valence" factors was not significant ($F_{4,2312} = 0.67$, $p = 0.62$).

the analysis did not detect significant differences in the intensity ratings of recognized affective scenes (PD: 7.53 ± 1.40, Control: 7.62 ± 0.86; $F_{1,77.01} = 0.12$, $p = 0.73$)
Tags: emotion recognition, behavioral

Facial emotion recognition deficits are associated with hypomimia and related brain correlates in Parkinson's disease.

Item Type Journal Article
Author Jon Rodríguez-Antigüedad
Author Saül Martínez-Horta
Author Andrea Horta-Barba
Author Arnau Puig-Davi
Author Antonia Campolongo
Author Frederic Sampedro
Author Helena Bejr-Kasem
Author Juan Marín-Lahoz
Author Javier Pagonabarraga
Author Jaime Kulisevsky
Abstract Hypomimia is a frequent manifestation in Parkinson's disease (PD) that can affect interpersonal relationships and quality of life. Recent studies have suggested that hypomimia is not only related to motor dysfunction but also to impairment in emotional processing networks. Therefore, we hypothesized that the severity of hypomimia could be associated with performance on a task aimed at assessing facial emotion recognition. In this study, we explored the association between hypomimia, recognition of facial expressions of basic emotions using the Ekman 60 Faces Test (EF), and brain correlates of both hypomimia and performance on the EF. A total of 94 subjects underwent clinical assessments (neurological and neuropsychological examinations), and 56 of them participated in the neuroimaging study. We found significant correlation between hypomimia, EF Disgust ($r = -0.242$, $p = 0.022$) and EF Happiness ($r = -0.264$, $p = 0.012$); an independent reduction in Cortical Thickness (Cth) in the postcentral gyrus, insula, middle and superior temporal gyri, supramarginal gyrus, banks of the superior temporal sulcus, bilateral fusiform gyri, entorhinal cortex, parahippocampal gyrus, inferior and superior parietal cortex, and right cuneus and precuneus; and multiple correlations between negative emotions such as EF Disgust or EF Anger and a reduced Cth in fronto-temporo-parietal regions. In conclusion, these results suggest that the association between hypomimia and emotion recognition deficits in individuals with PD might be mediated by shared

circuits, supporting the concept that hypomimia is not only the result of the dysfunction of motor circuits, but also of higher cognitive functions.

**Date**	2024 Dec
**Language**	eng
**License**	© 2024. The Author(s), under exclusive licence to Springer-Verlag GmbH Austria, part of Springer Nature.
**Extra**	Place: Austria
**Volume**	131
**Pages**	1463-1469
**Publication**	Journal of neural transmission (Vienna, Austria : 1996)
**DOI**	10.1007/s00702-023-02725-3
**Issue**	12
**Journal Abbr**	J Neural Transm (Vienna)
**ISSN**	1435-1463 0300-9564
**PMID**	38206439
**Date Added**	6.7.2025, 19:09:34
**Modified**	5.9.2025, 14:53:30

Notes:

Included

Sample characteristics

Size: 94 PD, no comparison group; however, it was an imaging correlational study

PD-type:

PD-duration: M = 6.4, SD = 3.4

Medication: LEDD calculated

Hoehn-Yahr: M = 22, SD = 0.4

UPDRS-3: M = 27.9, SD = 8.6

Gender (male): 34 (36.2%)

Age: M = 69.3, SD = 7.6

Other neurological disease (tumor, stroke, etc.): NA

Other major psychopathology: NA

Origin country (or ethnicity): Spain

method behavioural

instruments used in order to quantify the variables

Social cognition aspect: emotion recognition

Name of the task: 60 Ekman faces test

Type of stimulus [face/voice etc., Ekman faces/other etc.]: 60 Ekman photographs

Task condition:

Operationalization: Correct answers

MRI: MRI scans were acquired using a 3-Tesla Philips Achieva scanner in all participants who agreed to participate in the neuroimaging study. T1-weigthed images were obtained using a specific axial T13D–MPRAGE MRI (TR/TE 500/50 ms, flip angle=8°, field of view (FOV) 23 cm, with in-plane resolution of 256×256- and 1-mm slice thickness).

measure: cortical thickness (Cth)

Main findings related to the review's scope

multiple correlations stood out, particularly between negative emotions such as Disgust or Anger and a reduced Cth relative to the severity of hypomimia in frontotemporo-parietal regions

the correlations between negative emotions such as disgust or anger and the superior frontal gyrus, middle temporal gyrus, superior parietal gyrus, right fusiform gyrus and right precuneus remained significant (after applying the FDR correction

Facial emotion recognition deficits are associated with hypomimia and related brain correlates… 1467

Table 2 T1-MRI cortical thickness correlates of Ekman 60 faces test

		EF anger	EF disgust	EF fear	EF happiness	EF sadness	EF surprise	EF total
Superior frontal gyrus	Left	**0.391* (0.011)**	**0.354 (0.021*)**	0.096 (0.547)	0.107 (0.499)	0.290 (0.062)	−0.087 (0.584)	**0.421* (0.006)**
	Right	**0.446* (0.003)**	0.275 (0.078)	−0.051 (0.749)	−0.014 (0.930)	0.241 (0.124)	0.037 (0.818)	**0.350 (0.020)**
Rostral middle frontal gyrus	Left	**0.382 (0.012)**	0.182 (0.248)	−0.090 (0.572)	0.071 (0.654)	0.249 (0.112)	0.212 (0.178)	**0.351 (0.023)**
	Right	0.247 (0.115)	**0.348 (0.024)**	0.023 (0.883)	0.063 (0.690)	0.263 (0.093)	0.213 (0.176)	**0.400 (0.009)**
Superior temporal gyrus	Left	0.211 (0.179)	0.107 (0.499)	−0.102 (0.519)	0.052 (0.741)	0.122 (0.443)	0.060 (0.705)	0.152 (0.338)
	Right	0.214 (0.174)	0.070 (0.658)	0.042 (0.790)	0.223 (0.156)	0.013 (0.936)	−0.027 (0.864)	0.162 (0.307)
Banks of the superior temporal sulcus	Left	**0.344 (0.026)**	0.207 (0.187)	−0.085 (0.590)	0.161 (0.307)	0.164 (0.301)	0.024 (0.878)	0.268 (0.086)
	Right	0.268 (0.086)	0.024 (0.879)	0.201 (0.202)	**0.386 (0.012)**	−0.014 (0.928)	−0.195 (0.217)	0.202 (0.200)
Middle temporal gyrus	Left	**0.382* (0.012)**	0.116 (0.466)	−0.072 (0.650)	0.159 (0.314)	0.066 (0.678)	−0.087 (0.586)	0.191 (0.224)
	Right	**0.352 (0.022)**	0.245 (0.118)	0.197 (0.212)	0.155 (0.328)	0.001 (0.993)	−0.056 (0.725)	**0.324 (0.036)**
Superior parietal cortex	Left	**0.368 (0.016)**	0.150 (0.342)	−0.038 (0.812)	−0.047 (0.770)	0.130 (0.414)	−0.147 (0.353)	0.188 (0.233)
	Right	**0.428* (0.005)**	0.209 (0.185)	−0.006 (0.969)	−0.076 (0.633)	0.234 (0.137)	−0.012 (0.938)	**0.322 (0.037)**
Inferior parietal cortex	Left	**0.326 (0.035)**	0.126 (0.425)	0.075 (0.638)	0.025 (0.875)	0.008 (0.961)	−0.169 (0.286)	0.170 (0.281)
	Right	**0.346 (0.025)**	0.273 (0.080)	0.008 (0.959)	0.150 (0.343)	0.197 (0.210)	−0.034 (0.830)	**0.325 (0.036)**
Insula	Left	0.181 (0.250)	0.303 (0.051)	−0.093 (0.557)	0.070 (0.660)	0.244 (0.120)	0.053 (0.741)	0.250 (0.110)
	Right	0.234 (0.135)	0.274 (0.079)	−0.024 (0.882)	0.133 (0.400)	0.163 (0.302)	0.148 (0.349)	0.303 (0.051)
Fusiform gyri	Left	0.225 (0.152)	0.296 (0.057)	−0.035 (0.824)	0.172 (0.276)	0.135 (0.394)	−0.017 (0.912)	0.245 (0.117)
	Right	0.242 (0.123)	**0.385* (0.012)**	0.013 (0.934)	0.217 (0.167)	0.034 (0.830)	−0.002 (0.992)	0.277 (0.075)
Parahipoccampal gyrus	Left	−0.125 (0.430)	0.254 (0.105)	−0.038 (0.809)	0.078 (0.622)	0.034 (0.830)	−0.220 (0.161)	−0.034 (0.830)
	Right	0.092 (0.563)	0.124 (0.435)	0.087 (0.584)	−0.047 (0.767)	0.058 (0.715)	−0.243 (0.121)	0.059 (0.713)
Entorhinal cortex	Left	−0.030 (0.849)	0.303 (0.051)	0.133 (0.403)	0.093 (0.558)	−0.088 (0.580)	−0.060 (0.704)	0.105 (0.506)
	Right	0.011 (0.943)	0.091 (0.567)	−0.072 (0.650)	0.000 (1.000)	−0.164 (0.298)	−0.211 (0.179)	−0.122 (0.440)
Cuneus	Left	−0.128 (0.420)	0.066 (0.676)	0.127 (0.423)	−0.006 (0.972)	−0.082 (0.604)	−0.229 (0.145)	−0.079 (0.619)
	Right	0.009 (0.954)	0.063 (0.693)	−0.082 (0.607)	0.077 (0.629)	−0.048 (0.761)	0.027 (0.867)	−0.011 (0.946)
Precuneus	Left	**0.381 (0.013)**	0.256 (0.102)	0.052 (0.746)	0.146 (0.356)	0.160 (0.312)	−0.078 (0.625)	**0.326 (0.035)**
	Right	**0.362* (0.019)**	0.285 (0.068)	0.010 (0.952)	0.078 (0.624)	0.179 (0.258)	−0.068 (0.670)	**0.309 (0.047)**

EF Ekman 60 Faces Test. Correlation coefficient (*p* value)

In bold those values with $p < 0.05$

*Significant correlations after FDR correction

Tags: emotion recognition, Imaging

Facial emotion recognition impairment in chronic temporal lobe epilepsy

Item Type Journal Article

Author Stefano Meletti
Author Francesca Benuzzi
Author Gaetano Cantalupo
Author Guido Rubboli
Author Carlo Alberto Tassinari
Author Paolo Nichelli

Abstract Purpose: To evaluate facial emotion recognition (FER) in a cohort of 176 patients with chronic temporal lobe epilepsy (TLE). Methods: FER was tested by matching facial expressions with the verbal labels for the following basic emotions: happiness, sadness, fear, disgust, and anger. Emotion recognition performances were analyzed in medial (n = 140) and lateral (n = 36) TLE groups. Fifty healthy subjects served as controls. The clinical and neuroradiologic variables potentially affecting the ability to

recognize facial expressions were taken into account. Results: The medial TLE (MTLE) group showed impaired FER (86% correct recognition) compared to both the lateral TLE patients (FER = 93.5%) and the controls (FER = 96.4%), with 42% of MTLE patients recording rates of FER that were lower [by at least 2 standard deviations (SDs)] than the control mean. The MTLE group was impaired compared to the healthy controls in the recognition of all basic facial expressions except happiness. The patients with bilateral MTLE were the most severely impaired, followed by the right and then the left MTLE patients. FER was not affected by type of lesion, number of antiepileptic drugs (AEDs), aura semiology, or gender. Conversely, the early onset of seizures/epilepsy was related to FER deficits. These deficits were already established in young adulthood, with no evidence of progression in older MTLE patients. Conclusion: These results on a large cohort of TLE patients demonstrate that emotion recognition deficits are common in MTLE patients and widespread across negative emotions. We confirm that early onset seizures with right or bilateral medial temporal dysfunction lead to severe deficits in recognizing facial expressions of emotions.

**Date**	2009-06
**Language**	English
**Extra**	Place: 111 RIVER ST, HOBOKEN 07030-5774, NJ USA Type: Article
**Volume**	50
**Publisher**	WILEY
**Pages**	1547-1559
**Publication**	EPILEPSIA
**DOI**	10.1111/j.1528-1167.2008.01978.x
**Issue**	6
**ISSN**	0013-9580
**Date Added**	14.7.2025, 14:50:42
**Modified**	5.9.2025, 14:46:15

Notes:

Not Included: not on PD

Tags: EXCLUDED

Facial emotion recognition impairment in patients with Parkinson's disease and isolated apathy.

**Item Type**	Journal Article
**Author**	Mercè Martínez-Corral
**Author**	Javier Pagonabarraga
**Author**	Gisela Llebaria
**Author**	Berta Pascual-Sedano
**Author**	Carmen García-Sánchez
**Author**	Alexandre Gironell
**Author**	Jaime Kulisevsky

Abstract Apathy is a frequent feature of Parkinson's disease (PD), usually related with executive dysfunction. However, in a subgroup of PD patients apathy may represent the only or predominant neuropsychiatric feature. To understand the mechanisms underlying apathy in PD, we investigated emotional processing in PD patients with and without apathy and in healthy controls (HC), assessed by a facial emotion recognition task (FERT). We excluded PD patients with cognitive impairment, depression, other affective disturbances and previous surgery for PD. PD patients with apathy scored significantly worse in the FERT, performing worse in fear, anger, and sadness recognition. No differences, however, were found between nonapathetic PD patients and HC. These findings suggest the existence of a disruption of emotional-affective processing in cognitive preserved PD patients with apathy. To identify specific dysfunction of limbic structures in PD, patients with isolated apathy may have therapeutic and prognostic implications.

Date 2010 Jul 28
Language eng
Extra Place: United States
Volume 2010
Pages 930627
Publication Parkinson's disease
DOI 10.4061/2010/930627
Journal Abbr Parkinsons Dis
ISSN 2042-0080 2090-8083
PMID 20976097
PMCID PMC2957329
Date Added 6.7.2025, 19:09:35
Modified 14.7.2025, 15:09:53

Notes:

Included

Sample characteristics

Size: PD-Apathy (PD-A) = 12, PD-nonApathy (PD-NA) = 19, HC = 16 (comparable in age, gender, and education)

PD-type: NA

PD-duration: PD-A: M=4.83 (SD=2.95); PD-NA: M=7.26 (SD=4.26)

Medication: On state

Hoehn-Yahr: PD-A: M=2.00 (SD=.00), PD-NA: M=2.03 (SD=0.35)

UPDRS-3: PD-A:M= 20.58 (SD=9.75); PD-NA: M=20.77 (SD=5.11)

Gender (male): PD-A: 10 (83.33%); PD-NA: 13 (68.42%)

Age: PD-A: M=65.67 (SD=4.96); PD-NA: M=60.37 (SD=9.38)

Other neurological disease (tumor, stroke, etc.): No visuoperceptive impairment

Other major psychopathology: no depression.

Origin country (or ethnicity): Spain

method (Review, meta-analysis or observational and/or self-reported):

instruments used in order to quantify the variables

Social cognition aspect: Facial emotion recognition

Name of the task: Based on "facial emotion recognition test (FERT)

Type of stimulus [face/voice etc., Ekman faces/other etc.]: 36 pictures. 6 per emotion. Caucasian and oriental actors.

Task condition: Pictures displayed for 3 seconds. No time-limit for response. Forced choice (happy, surprise, disgust, anger, sadness, fear)

Operationalization: The percentage of errors

Main findings related to the review's scope

We observed both a group effect and an emotion effect, with a significant interaction between group and emotion.

Post hoc analysis showed significant differences between apathetic PD patients and both nonapathetic patients and HC. No significant differences were observed between nonapathetic PD patients and HC.

Apathetic PD patients scored significantly worse only in the recognition of fear anger, and sadness.

Tags: Emotion Recognition, behavioral

Facial emotion recognition in individuals with mild cognitive impairment: An exploratory study.

Item Type Journal Article
Author Francesca Burgio
Author Arianna Menardi
Author Silvia Benavides-Varela
Author Laura Danesin
Author Andreina Giustiniani
Author Jan Van den Stock
Author Roberta De Mitri
Author Roberta Biundo
Author Francesca Meneghello
Author Angelo Antonini
Author Antonino Vallesi
Author Beatrice de Gelder
Author Carlo Semenza
Abstract Understanding facial emotions is fundamental to interact in social environments and modify behavior accordingly. Neurodegenerative processes can progressively transform affective responses and affect social competence. This exploratory study examined the neurocognitive correlates of face recognition, in individuals with two mild cognitive impairment (MCI) etiologies (prodromal to dementia - MCI, or consequent to Parkinson's disease - PD-MCI). Performance on the identification and memorization of neutral and emotional facial expressions was assessed in 31 individuals with MCI, 26 with PD-MCI, and 30 healthy controls (HC). Individuals with MCI exhibited selective impairment in recognizing faces expressing fear, along with difficulties in remembering both neutral and emotional faces. Conversely, individuals with PD-MCI showed no differences compared with the HC in either emotion recognition or memory. In MCI, no significant association emerged between the memory for facial expressions and cognitive difficulties. In PD-MCI, regression analyses showed significant associations with higher-level cognitive functions in the emotional memory task, suggesting the presence of compensatory mechanisms. In a subset of participants, voxel-based morphometry revealed that the performance on emotional tasks correlated with regional changes in gray matter volume. The performance in the matching of negative expressions was predicted by volumetric changes in brain areas engaged in face and emotional processing, in particular increased volume in thalamic nuclei and atrophy in the right parietal cortex. Future studies should leverage on neuroimaging data to determine whether differences in emotional recognition are mediated by pathology-specific atrophic patterns.
Date 2024 Jun
Language eng
License © 2024. The Psychonomic Society, Inc.
Extra Place: United States
Volume 24
Pages 599-614
Publication Cognitive, affective & behavioral neuroscience
DOI 10.3758/s13415-024-01160-5
Issue 3

**Journal Abbr**	Cogn Affect Behav Neurosci
**ISSN**	1531-135X 1530-7026
**PMID**	38316707
**Date Added**	6.7.2025, 19:09:42
**Modified**	5.9.2025, 14:29:44

Notes:

Included

sample characteristics

size: 26 PD-MCI, 31 MCI and 30 HC (age-matched)

Parkinson's Disease type and duration: idiopathic PD-MCI, Mduration=NA

Medication: on medication

Hoehn-Yahr: M=NA

UPDRS-3: M=NA

Gender (male): 20 males (77%)

averaged ages (SD, range): M= 70.35 (SD=11.13)

other neurological disease (tumor, stroke, etc.): None

other major psychopathology: None

origin country (or ethnicity): Italy

method observational and imaging

instruments used in order to quantify the variables

Social cognition aspect: emotion recognition

Name of the task: the Facial Expressive Action Stimulus Test (FEAST) battery

type of stimulus [face/voice etc., Ekman faces/other etc.]:

Facial Expression Matching Task - Human (FEM-H) - emotion recognition ability in human faces. On each trial, three pictures are shown: one picture on top (sample) and two pictures underneath. One of the two bottom pictures presents a face expressing the same emotion as the sample, the other is a distractor. The participant has to match the faces based on their emotional expression.

task condition: anger, disgust, fear, sadness, surprise, happiness

operationalization: accuracy and RT

Emotional Face Memory Task (FaMe-E) - includes an encoding and a recognition phase. Stimuli consist of Caucasian faces with a frontal emotional facial expression and frontal eye gaze. In the encoding phase, 50 stimuli are presented for 3000 ms, and participants are instructed to memorize each face as they will be asked to recognize them subsequently. In the recognition phase, two adjacent faces are presented simultaneously: the target face and a distractor, for a total of 50 trials.

task condition: fear, sadness, or happiness

operationalization: accuracy and RT

to evaluate the performance on face and emotional processing among groups, participants' proportion of errors and response times (RT's) at each FEAST task were calculated. Like in previous studies (Vandierendonck, 2017), both measures were combined into linear integrated speed-accuracy (LISAS) scores. Of note, higher LISAS scores correspond to worse performances in terms of slower RTs and/or higher rates of errors.

MRI and VBM (on sub-sample: n = 28 HC; n = 31 MCI; n = 10 PD-MCI)

1.5 T Achieva Philips scanner (Philips Medical Systems, Best, The Netherlands) with an 8-channel head coil at the San Camillo Hospital in Venice Lido, Italy.

A standard clinical T1-weighted anatomical scan was collected (TE = 3.5 ms, TR = 7.6 ms, 3D-acquisition, FOV: 240-mm × 240-mm × 280-mm, 1-mm x 1-mm x 0.59-mm voxel size). Images were then pre-processed using the Computational Anatomy Toolbox (CAT12) (Gaser et al., 2022) for SPM12 (Ashburner et al., 2014; www.fil.ac.uk/spm/) in Matlab R2017b (The Mathworks, Inc., Natick, MA 2017). Preprocessing steps included bias-field and noise removal, skull stripping, segmentation into the gray and white matter tissue components, smoothing, and normalization to MNI space. The CAT12 toolbox has the further advantage of providing ratings of image data quality, based on basic image properties, noise, and geometric distortions (e.g., due to motion).

for the purpose of the current study, volumetric data from the native space of the individual were extracted from regions of interest (ROIs) of the Desikan-Killiany (Desikan et al., 2006) and Cobra (https://github.com/CoBrALab/atlases) atlases, which respectively cover cortical, subcortical, and cerebellar areas of the human brain.

focused on: amygdala (amyg), the striatum (striat), the thalamus (thal), and the right parietal cortex (rPariet)

Main findings related to the review's scope

FEM-H: matching emotional faces: No sig dif between PD-MCI and HC/MCI

FaMe-E: MCI patients showed significantly higher LISAS scores compared with PD-MCI patients in the memory of happy ($t = 3.272$; $p = 0.005$), sad ($t = 2.953$; $p = 0.012$), and fearful faces.

No differences were observed between HC and PD-MCI patients

in the global LISAS score of memory of emotional faces, the MCI group performed worse than both HC and PD-MCI group, but no differences were found between PD-MCI and HC

for PD-MCI: the thalamic volume showed a positive association to LISAS scores.

MRI:

FAM-H: no sig results regarding the PD-MCI group.

Coefficients and significance levels of the predictors in regard to performance scores at the FaMe-E_Fear scale: increase in the volume of both the striatum and the right-parietal regions results in a better performance of the PD-MCI patients on this task (lower LISAS scores).

Tags: Emotion recognition, Imaging, behavioral

Facial emotion recognition in Parkinson's disease

**Item Type**	Journal Article
**Author**	Stefania Kalampokini
**Author**	Epameinondas Lyros
**Author**	Maxine Luley
**Author**	Jörg Spiegel
**Author**	Marcus Unger
**Date**	2016
**URL**	https://www.sciencedirect.com/science/article/pii/S1353802015005453
**Volume**	22
**Pages**	e55
**Publication**	Parkinsonism & Related Disorders
**DOI**	https://doi.org/10.1016/j.parkreldis.2015.10.109
**ISSN**	1353-8020
**Date Added**	6.7.2025, 19:12:36
**Modified**	6.7.2025, 19:12:36

Notes:

Not Included: Not an empirical research

Tags: EXCLUDED

Facial emotion recognition in Parkinson's disease: A review and new hypotheses.

**Item Type**	Journal Article
**Author**	Soizic Argaud
**Author**	Marc Vérin
**Author**	Paul Sauleau
**Author**	Didier Grandjean
**Abstract**	Parkinson's disease is a neurodegenerative disorder classically characterized by motor symptoms. Among them, hypomimia affects facial expressiveness and social communication and has a highly negative impact on patients' and relatives' quality of life. Patients also frequently experience nonmotor symptoms, including emotional-processing impairments, leading to difficulty in recognizing emotions from faces. Aside from its theoretical importance, understanding the disruption of facial emotion recognition in PD is crucial for improving quality of life for both patients and caregivers, as this impairment is associated with heightened interpersonal difficulties. However, studies assessing abilities in recognizing facial emotions in PD still report contradictory outcomes. The origins of this inconsistency are unclear, and several questions (regarding the role of dopamine replacement therapy or the possible consequences of hypomimia) remain unanswered. We therefore undertook a fresh review of relevant articles focusing on facial emotion recognition in PD to deepen current understanding of this nonmotor feature, exploring multiple significant

potential confounding factors, both clinical and methodological, and discussing probable pathophysiological mechanisms. This led us to examine recent proposals about the role of basal ganglia-based circuits in emotion and to consider the involvement of facial mimicry in this deficit from the perspective of embodied simulation theory. We believe our findings will inform clinical practice and increase fundamental knowledge, particularly in relation to potential embodied emotion impairment in PD. © 2018 The Authors. Movement Disorders published by Wiley Periodicals, Inc. on behalf of International Parkinson and Movement Disorder Society.

Date 2018 Apr
Language eng
License © 2018 The Authors. Movement Disorders published by Wiley Periodicals, Inc. on behalf of International Parkinson and Movement Disorder Society.
Extra Place: United States
Volume 33
Pages 554-567
Publication Movement disorders : official journal of the Movement Disorder Society
DOI 10.1002/mds.27305
Issue 4
Journal Abbr Mov Disord
ISSN 1531-8257 0885-3185
PMID 29473661
PMCID PMC5900878
Date Added 6.7.2025, 19:09:34
Modified 5.9.2025, 14:26:42

Notes:

__Not Included:__ not a systematic review

Tags: EXCLUDED

Facial Emotion Recognition in Parkinson's Disease: An fMRI Investigation.

Item Type Journal Article
Author Albert Wabnegger
Author Rottraut Ille
Author Petra Schwingenschuh
Author Petra Katschnig-Winter
Author Mariella Kögl-Wallner
Author Karoline Wenzel
Author Anne Schienle
Abstract BACKGROUND: Findings of behavioral studies on facial emotion recognition in Parkinson's disease (PD) are very heterogeneous. Therefore, the present investigation additionally used functional magnetic resonance imaging (fMRI) in order to compare brain activation during emotion perception between PD patients and healthy controls. METHODS AND FINDINGS: We included 17 nonmedicated, nondemented PD

patients suffering from mild to moderate symptoms and 22 healthy controls. The participants were shown pictures of facial expressions depicting disgust, fear, sadness, and anger and they answered scales for the assessment of affective traits. The patients did not report lowered intensities for the displayed target emotions, and showed a comparable rating accuracy as the control participants. The questionnaire scores did not differ between patients and controls. The fMRI data showed similar activation in both groups except for a generally stronger recruitment of somatosensory regions in the patients. CONCLUSIONS: Since somatosensory cortices are involved in the simulation of an observed emotion, which constitutes an important mechanism for emotion recognition, future studies should focus on activation changes within this region during the course of disease.

**Date**	2015
**Language**	eng
**Extra**	Place: United States
**Volume**	10
**Pages**	e0136110
**Publication**	PloS one
**DOI**	10.1371/journal.pone.0136110
**Issue**	8
**Journal Abbr**	PLoS One
**ISSN**	1932-6203
**PMID**	26285212
**PMCID**	PMC4540566
**Date Added**	6.7.2025, 19:09:35
**Modified**	5.9.2025, 15:00:10

Notes:

Included

Sample characteristics

Size: 17 PD, 22 HC

PD-type: Idiopathic PD

PD-duration: M = 6.28 (SD = 3.64)

Medication: OFF state

Hoehn-Yahr: M=2.09 SD=0.2 (2-2.5)

UPDRS-3: M = 36.1 (SD = 13.0), Range = 17-49

Gender (male): 9 males (53%)

Age: M=55.2 SD=9.4

Other neurological disease (tumor, stroke, etc.): none

Other major psychopathology: none

Origin country (or ethnicity): Austria

method (Review, meta-analysis or observational and/or self-reported):

instruments used in order to quantify the variables

Social cognition aspect: emotion recognition

Name of the task: NA

Type of stimulus [face/voice etc., Ekman faces/other etc.]: 50 pictures from the Karolinska Directed Emotional Faces

Task condition: disgust, sadness, anger, fear, or neutral

Operationalization: participants gave affective ratings for the pictures. For each image they indicated the intensity of expressed disgust, fear, sadness, and anger (Please indicate how intensely the depicted person experienced disgust/ fear/ anger/ sadness:1= 'very little',9= 'very intense')

calculated the classification accuracy, which was defined as the difference between the perceived intensity of a target emotion and the mean intensity of all non-target emotions for a specific facial expression (e.g., disgust classification accuracy for a disgust expression = disgust intensity minus mean intensity of non-target emotions [anger, fear, sadness]).

Method fMRI

3 Tesla Siemens TrioTim, 23-channel headcoil. For the functional runs a total of 164 volumes were acquired by using an echo-planar imaging protocol (35 descending slices; slice thickness: 3mm; TE = 30ms; TR = 2300ms; Voxel size: 3.0x3.0x3.0 mm; FoV: 192; flip angle: 90°; slice orientation -25° tilted from the AC-PC line). To account for saturation effects 3 slices from the beginning of the time series were discarded.

BOLD signal

Main findings related to the review's scope

no significant differences between PD and HC in intensity or accuracy.

Results fMRI

See Tab. 2 for MNI coordinates!

In comparison to PD, HC displayed greater activation in the putamen and inferior frontal gyrus when looking at sad faces.

PD patients were characterized by greater activation in parietal regions (SII, inferior parietal cortex) across all emotion conditions (anger, disgust, sadness, fear) than the control participants.

An exploratory whole-brain analysis had revealed no significant group differences.

SII activation was positively correlated with fear intensity ratings (MNI coordinates: 51, -9, 21, t = 3.45, p(FWE) = .038, cluster size = 34) and accuracy ratings for fearful expressions (MNI coordinates: 54, -9, 18, t = 3,61, p(FWE) = .045, cluster. size = 30). In addition, SII activation was positively correlated with disgust intensity ratings (MNI coordinates: -36, -27, 21, t = 3.15, p(FWE) = .032, cluster size = 18) and accuracy ratings for disgusted expressions (MNI coordinates: -36, -24, 15, t = 2.98, p(FWE) = .041, cluster size = 20). Anger intensity rating showed a positive association with inferior parietal activation (MNI coordinates: -48, -69, 33, t = 4.62, p(FWE) = .012; cluster size = 82).

Table 2. Comparison of brain activation in the emotion conditions between patients v

	H	X	Y
**PD > CG: Anger > Neutral**			
Secondary somatosensory cortex (BA 22)	R	63	-12
**PD > CG: Disgust > Neutral**			
Inferior parietal lobule (BA 48)	R	54	-33
Secondary somatosensory cortex (BA 48)	L	-48	-21
**PD > CG: Fear > Neutral**			
Inferior parietal lobule (BA 40)	L	-51	-45
OFC (BA 45)	R	57	30
**PD > CG: Sadness > Neutral**			
Secondary somatosensory cortex (BA 22)	R	63	-12
Secondary somatosensory cortex (BA 48)	L	-51	-12
**CG > PD: Sadness > Neutral**			
Putamen (BA 48)	R	30	18
Inferior frontal gyrus (BA 47)	R	30	21

BA = Brodmann Area; H = hemisphere, MNI coordinates (x,y,z), *p*(FWE) = corrected for f

doi:10.1371/journal.pone.0136110.t002

Tags: emotion recognition, Imaging, behavioral

Facial emotion recognition in Parkinson's disease: Association with age and olfaction.

**Item Type**	Journal Article
**Author**	S. Kalampokini
**Author**	E. Lyros
**Author**	M. Luley
**Author**	J. Schöpe
**Author**	J. Spiegel
**Author**	J. Bürmann
**Author**	U. Dillmann
**Author**	K. Fassbender
**Author**	M. M. Unger
**Abstract**	OBJECTIVE: The ability to recognize facial emotion expressions has been reported to be impaired in Parkinson's disease (PD), yet previous studies showed inconsistent findings. The aim of this study was to further investigate facial emotion recognition (FER) in PD patients and its association with demographic and clinical parameters (including motor and nonmotor symptoms). METHOD: Thirty-four nondemented PD patients and 24 age- and sex-matched healthy controls (HC) underwent clinical neurological and neuropsychological assessment, standardized olfactory testing with Sniffin' Sticks, and the Ekman 60 Faces Emotion Recognition Test. RESULTS: PD patients had a significantly lower score on the total FER task than HC (p = .006), even after controlling for the potential confounding factors depression and apathy. The PD group had a specific impairment in the recognition of surprise (p = .007). The recognition of anger approached statistical significance (p = .07). Increasing chronological age and age at disease onset were associated with worse performance on the FER task in PD patients. Olfactory function along with PD diagnosis predicted worse FER performance within all study participants. CONCLUSION: Facial emotion recognition and especially the recognition of surprise are significantly impaired in PD patients compared with age- and sex-matched HC. The association of FER with age and olfactory function is endorsed by common structures that undergo neurodegeneration in PD. The relevance of FER in social interaction stresses the clinical relevance and the need for further investigation in this field. Future studies should also determine whether impaired FER is already present in premotor stages of PD.
**Date**	2018 Apr
**Language**	eng
**Extra**	Place: England
**Volume**	40
**Pages**	274-284
**Publication**	Journal of clinical and experimental neuropsychology
**DOI**	10.1080/13803395.2017.1341470
**Issue**	3
**Journal Abbr**	J Clin Exp Neuropsychol
**ISSN**	1744-411X 1380-3395
**PMID**	28637374
**Date Added**	6.7.2025, 19:09:34
**Modified**	5.9.2025, 14:40:58

Notes:

Included

sample characteristics

size: 34 PD and 24 HC (match age and sex)

Parkinson's Disease type and duration: Idiopathic PD

Medication: On medication

Hoehn-Yahr: Md= 2.5 (1–4)

UPDRS-3: M= 20.7 SD= 6.7

Gender (male): 18 males (53%)

averaged ages (SD, range): M= 68.3 SD=8.2

other neurological disease (tumor, stroke, etc.): None

other major psychopathology: None

origin country (or ethnicity): Germany

method observational

instruments used in order to quantify the variables

Social cognition aspect: emotion recognition

Name of the task: NA

type of stimulus [face/voice etc., Ekman faces/other etc.]: Ekman

task condition: happiness, anger, sadness, fear, disgust, and surprise

operationalization: indicate which of the six emotions was depicted in each photograph by verbally referring to the emotion labels or pointing to one of the six emotion labels. >> SUM SCORE

Main findings related to the review's scope

PD patients had on average a statistically significant lower score on the total facial emotion recognition (FER) task than HC

PD group showed particularly an impairment in the recognition of surprise with a statistically significant lower score than HC

The emotion most accurately recognized by both groups was happiness, while fear was least accurately recognized by both groups.

PD group showed that FER was significantly associated with chronological age and age of onset of the disease. the effect of age on FER performance was more pronounced in the PD patients than in controls.

Tags: Emotion recognition, behavioral

Facial emotion recognition in Parkinson's disease: The role of executive and affective domains.

**Item Type**	Journal Article
**Author**	Antònia Siquier
**Author**	Pilar Andrés
**Abstract**	OBJECTIVE: The ability to recognize emotions from facial expression (FER) may be impaired in Parkinson's disease (PD). We aimed to explore FER in PD patients by using a dynamic presentation of emotions across different intensities and to examine the extent to which executive and affective alterations contributed to FER deficits. METHOD: Fifteen PD patients and 15 healthy controls were assessed on the emotion recognition task (ERT). We also explored how clinical and executive factors could have contributed to ERT accuracy. RESULTS: PD patients showed poorer performance on the ERT, specifically on angry expressions, but they benefited from increased intensity as much as controls did. Differences were also found for apathy, depression, and executive tests, especially in the inhibition domain. Importantly, differences between groups on the ERT disappeared when controlling for inhibition and the affective symptoms. A significant effect of inhibition dysfunction was also observed on the ERT performance. CONCLUSIONS: Our findings demonstrate the presence of emotion recognition deficits of morphed facial expressions in patients with PD. Moreover, they suggest that inhibition dysfunctions may act as an important factor negatively influencing FER. The present study highlights the complex nature of emotion processing and its relation with emotional-affective and cognitive aspects to provide a better understanding of FER deficits in PD. (PsycInfo Database Record (c) 2022 APA, all rights reserved).
**Date**	2022 Jul
**Language**	eng
**Extra**	Place: United States
**Volume**	36
**Pages**	384-393
**Publication**	Neuropsychology
**DOI**	10.1037/neu0000814
**Issue**	5
**Journal Abbr**	Neuropsychology
**ISSN**	1931-1559 0894-4105
**PMID**	35511563
**Date Added**	6.7.2025, 19:09:34
**Modified**	5.9.2025, 14:56:58

Notes:

Included

Sample characteristics

Size:15 PD, 15 HC

PD-type: NA

PD-duration: M = 5.56, SD = 4.51 (since first diagnosis

Medication: on state

Hoehn-Yahr: < 4, M = 1.77, SD = 0.37

UPDRS-3: M = 25.6, SD = 13.20

Gender (male): 14 (93%)

Age: M = 67.4, SD = 9.7

Other neurological disease (tumor, stroke, etc.): None

Other major psychopathology: None

Origin country (or ethnicity): Spain

method (Review, meta-analysis or observational and/or self-reported):

instruments used in order to quantify the variables

Social cognition aspect: emotion recognition

Name of the task: NA

Type of stimulus [face/voice etc., Ekman faces/other etc.]: dynamically morphed video clips of six facial expressions on which the degree of emotional expression increases in intensity by 20%, starting at neutral morphed into 40%, 60%, 80%, and 100%. The duration of the video clips ranges from 1 (40%) to 3 (100%) s.

Task condition: anger, disgust, fear, happiness, sadness, and surprise

Operationalization: Participants had to indicate after each morph which emotion was displayed by a six-alternative force choice response. 96 trials in total, with four trials for every four intensities and for every six emotions.

across the four intensities, the maximum score for each emotion was 16, with a maximum score of 96 for the entire test (number of correctly labeled expressions per emotion and intensity

Main findings related to the review's scope

PD patients were globally less accurate than healthy participants identifying the emotions.

Interactions between Emotion × Group was also found. PD participants seemed to suffer from difficulties in recognizing anger, happiness and surprise, but no group difference was observed for disgust, fear, and sadness.

Tags: emotion recognition, behavioral

Facial expression decoding in early Parkinson's disease

Item Type Journal Article
Author MD Pell
Author CL Leonard
Abstract The ability to derive emotional and non-emotional information from unfamiliar, static faces was evaluated in 21 adults with idiopathic Parkinson's disease (PD) and 21 healthy control subjects. Participants' sensitivity to emotional expressions was comprehensively assessed in tasks of discrimination, identification, and rating of five basic emotions: happiness, (pleasant) surprise, anger, disgust, and sadness. Subjects also discriminated and identified faces according to underlying phonemic ("facial speech") cues and completed a neuropsychological test battery. Results uncovered limited evidence that the processing of emotional faces differed between the two groups in our various conditions, adding to recent arguments that these skills are frequently intact in non-demented adults with PD [R. Adolphs, R. Schul, D. Tranel, Intact recognition of facial emotion in Parkinson's disease, Neuropsychology 12 (1998) 253-258]. Patients could also accurately interpret facial speech cues and discriminate the identity of unfamiliar faces in a normal manner. There were some indications that basal ganglia pathology in PD contributed to selective difficulties recognizing facial expressions of disgust, consistent with a growing literature on this topic. Collectively, findings argue that abnormalities for face processing are not a consistent or generalized feature of medicated adults with mild-moderate PD, prompting discussion of issues that may be contributing to heterogeneity within this literature. Our results imply a more limited role for the basal ganglia in the processing of emotion from static faces relative to speech prosody, for which the same PD patients exhibited pronounced deficits in a parallel set of tasks [M.D. Pell, C. Leonard, Processing emotional tone from speech in Parkinson's disease: a role for the basal ganglia, Cogn. Affect. Behav. Neurosci. 3 (2003) 275-288]. These diverging patterns allow for the possibility that basal ganglia mechanisms are more engaged by temporally-encoded social information derived from cue sequences over time. (c) 2004 Elsevier B.V. All rights reserved.
Date 2005-05
Language English
Extra Place: RADARWEG 29, 1043 NX AMSTERDAM, NETHERLANDS Type: Article
Volume 23
Publisher ELSEVIER
Pages 327-340
Publication COGNITIVE BRAIN RESEARCH
DOI 10.1016/j.cogbrainres.2004.11.004
Issue 2-3
ISSN 0926-6410
Date Added 14.7.2025, 14:50:43
Modified 5.9.2025, 14:49:48

Notes:

Included

Sample characteristics

Size: 21 PD, 21 HC (mateched for sex, age, education)

PD-type: NA

PD-duration: M = 3.9 SD = 1.9

Medication: All but one were on medication

Hoehn-Yahr: M = 2.0, SD = 0.5

UPDRS-3: M = 14.5, SD = 7.1

Gender (male): 11 (52%)

Age: M = 61.7, SD = 8.6, Range = 51-83

Other neurological disease (tumor, stroke, etc.): None

Other major psychopathology: None

Origin country (or ethnicity): Canada

method Behavioral

instruments used in order to quantify the variables

Social cognition aspect: emotion recognition

Name of the task: NA; Emotional Discrimination

Type of stimulus [face/voice etc., Ekman faces/other etc.]:

1. 30 faces from actors
2. 30 facial pairs with five combinations of speech sounds

Task condition: decide if same of different emotional pairing

Operationalization: correct answers

Social cognition aspect: emotion recognition

Name of the task: NA; Emotional Identification

Type of stimulus [face/voice etc., Ekman faces/other etc.]: Same stimuli

Task condition: multiple choice format with a closed set of verbal labels as response alternatives (happiness, pleasant surprise, anger, disgust, and sadness). 40 trials.

Same for facial speed identification (5 spoken sounds)

Operationalization: correct answers

Social cognition aspect: emotion recognition

Name of the task: NA; Emotional Expression rating

Type of stimulus [face/voice etc., Ekman faces/other etc.]: Same stimuli

Task condition: rate the degree to which facial expressions conveyed particular emotions using a continuous scale signifying increased presence of the target emotion. 52 faces on five separate occasions; each time, subjects were instructed to attend to only one of the five target emotions and to rate each stimulus for how much of the emotion was being expressed on a scale from 0 (not at all) to 5 (very much)

Same for facial speed identification (5 spoken sounds)

Operationalization: correct answers

Main findings related to the review's scope

Discrimination

The accuracy of the PD and HC groups did not differ significantly on this task

Identification

There was no main effect of Group membership on the recognition of static expressions of emotion overall

Emotional expression rating

No group effect.

Tags: Emotion recognition, behavioral

Facial Expression Processing Is Not Affected by Parkinson's Disease, but by Age-Related Factors

Item Type Journal Article

Author Dilara Derya
Author June Kang
Author Young Kwon
Author Christian Wallraven

Abstract The question whether facial expression processing may be impaired in Parkinson's disease (PD) patients so far has yielded equivocal results - existing studies, however, have focused on testing expression processing in recognition tasks with static images of six standard, emotional facial expressions. Given that non-verbal communication contains both emotional and non-emotional, conversational expressions and that input to the brain is usually dynamic, here we address the question of potential facial expression processing differences in a novel format: we test a range of conversational and emotional, dynamic facial expressions in three groups - PD patients (n = 20), age- and education-matched older healthy controls (n = 20), and younger adult healthy controls (n = 20). This setup allows us to address both effects of PD and age-related differences. We employed a rating task for all groups in which 12 rating dimensions were used to assess evaluative processing of 27 expression videos from six different actors. We found that ratings overall were consistent across groups with several rating

dimensions (such as arousal or outgoingness) having a strong correlation with the expressions' motion energy content as measured by optic flow analysis. Most importantly, we found that the PD group did not differ in any rating dimension from the older healthy control group (HCG), indicating highly similar evaluation processing. Both older groups, however, did show significant differences for several rating scales in comparison with the younger adults HCG. Looking more closely, older participants rated negative expressions compared to the younger participants as more positive, but also as less natural, persuasive, empathic, and sincere. We interpret these findings in the context of the positivity effect and in-group processing advantages. Overall, our findings do not support strong processing deficits due to PD, but rather point to age-related differences in facial expression processing.

Date 2019-11-14
Language English
Extra Place: AVENUE DU TRIBUNAL FEDERAL 34, LAUSANNE, CH-1015, SWITZERLAND Type: Article
Volume 10
Publisher FRONTIERS MEDIA SA
Publication FRONTIERS IN PSYCHOLOGY
DOI 10.3389/fpsyg.2019.02458
ISSN 1664-1078
Date Added 14.7.2025, 14:50:32
Modified 5.9.2025, 14:33:10

Notes:

Not Included: irrelevant analyses

Tags: EXCLUDED

Facial expression recognition and subthalamic nucleus stimulation.

Item Type Journal Article
Author U. Schroeder
Author A. Kuehler
Author A. Hennenlotter
Author B. Haslinger
Author V. M. Tronnier
Author M. Krause
Author R. Pfister
Author R. Sprengelmeyer
Author K. W. Lange
Author A. O. Ceballos-Baumann
Abstract BACKGROUND: Deep brain stimulation (DBS) of the subthalamic nucleus (STN) improves motor signs in Parkinson's disease. However, clinical studies suggest that DBS of the STN may also affect cognitive and emotional functions. OBJECTIVE: To

study the impact of STN stimulation in Parkinson's disease on perception of facial expressions. RESULTS: There was a selective reduction in recognition of angry faces, but not other expressions, during STN stimulation. CONCLUSIONS: The findings may have important implications for social adjustment in these patients.

Date 2004 Apr
Language eng
Extra Place: England
Volume 75
Pages 648-650
Publication Journal of neurology, neurosurgery, and psychiatry
DOI 10.1136/jnnp.2003.019794
Issue 4
Journal Abbr J Neurol Neurosurg Psychiatry
ISSN 0022-3050 1468-330X
PMID 15026519
PMCID PMC1739017
Date Added 6.7.2025, 19:09:38
Modified 5.9.2025, 14:56:08

Notes:

Not Included: Only a PD group: pre-post DBS

Interesint article though.

Tags: EXCLUDED

Facial expression recognition in people with medicated and unmedicated Parkinson’s disease

Item Type Journal Article

Author R. Sprengelmeyer
Author A. W. Young
Author K. Mahn
Author U. Schroeder
Author D. Woitalla
Author T. Büttner
Author W. Kuhn
Author H. Przuntek

Abstract Recognition of facial expressions of emotion was investigated in people with medicated and unmedicated Parkinson’s disease (PD) and matched controls (unmedicated PD, n=16; medicated PD, n=20; controls, n=40). Participants in the medicated group showed some visual impairment (impaired contrast sensitivity) and performed less well on perception of unfamiliar face identity, but did not show significant deficits in the perception of sex, gaze direction, or familiar identity from the face. For both Parkinson’s disease groups, there was evidence of impaired recognition of facial expressions in comparison to controls. These deficits were more

consistently noted in the unmedicated group, who were also found to perform worse than the medicated group at recognising disgust from prototypical facial expressions, and at recognising anger and disgust in computer-manipulated images. Although both Parkinson's disease groups showed impairments of facial expression recognition, the consistently worse recognition of disgust in the unmedicated group is consistent with the hypothesis from previous studies that brain regions modulated by dopaminergic neurons are involved in the recognition of disgust.

Date 2003
URL https://www.sciencedirect.com/science/article/pii/S0028393202002956
Volume 41
Pages 1047-1057
Publication Neuropsychologia
DOI https://doi.org/10.1016/S0028-3932(02)00295-6
Issue 8
ISSN 0028-3932
Date Added 6.7.2025, 19:12:35
Modified 5.9.2025, 14:57:22

Notes:

Included

Sample characteristics

Size: not-medicated PD=16; medicated PD=20; HC=40

PD-type: idiopathic Parkinson

PD-duration: NA

Medication: OFF and ON states

Hoehn-Yahr: not-medicated PD M=1.7 S.D.=0.5 (1-2.5); medicated PD M=2.6 S.D.=0.9 (1-4)

UPDRS-3: not-medicated PD M=16.4 (S.D. 9.5); medicated PD M=30.0 (S.D.=15.8)

Gender (male): not-medicated PD 8 male (50%); medicated PD 9 male (45%)

Age: not-medicated PD M=56.7 years (S.D. 10.6 years); medicated PD M=56.9 years (S.D. 9.9 years)

Other neurological disease (tumor, stroke, etc.): NA

Other major psychopathology: NA

Origin country (or ethnicity): NA

method observational

instruments used in order to quantify the variables

Social cognition aspect: Emotion Recognition

Name of the task: Ekman 60 Facest test

Type of stimulus [face/voice etc., Ekman faces/other etc.]: Photographs of the faces of 10 people used in this test were taken from the Ekman and Friesen series.

Task condition: six basic emotions (happiness, surprise, fear, sadness, disgust, and anger)

Operationalization: Correct answers

Social cognition aspect: Emotion Recognition

Name of the task: Emotion Hexagon

Type of stimulus [face/voice etc., Ekman faces/other etc.]: set of facial expressions taken from the Ekman and Friesen series

Task condition: Pictures were ordered by placing each adjacent to the one it was most likely to be confused with; this gave the sequence happiness–surprise–fear–sadness–disgust–anger. The end of this sequence (anger and happiness) were then joined to create a hexagon, and interpolated (‘morphed’) images were created for the six continua that lie around the perimeter of this hexagon.

Operationalization: The task was to decide whether the image presented was most like happiness, surprise, fear, sadness, disgust, or anger. >> Correct answers

Main findings related to the review's scope

1. Ekman 60 Faces test: not-medicated PD performed significantly worse than controls in recognizing fear, sadness, anger, and disgust. medicated PD performed significantly below controls in recognizing fear and anger. medicated PD performed significantly better than not-medicated people with Parkinson's disease in recognizing disgust. all other comparisons were not sig.
2. Emotion Hexagon: not-medicated PD performed significantly worse than controls in recognizing sadness, anger, and disgust. No sig. diff between medicated PD and HC. medicated PD performed significantly better than not-medicated participants with Parkinson's disease in recognizing anger and disgust.

Tags: emotion recognition, behavioral

Facial expressions and identities recognition in Parkinson disease.

Item Type Journal Article
Author Silvia Gobbo
Author Elisa Urso
Author Aurora Colombo
Author Matilde Menghini
Author Cecilia Perin
Author Ioannis Ugo Isaias
Author Roberta Daini
Abstract Parkinson's Disease (PD) is associated with motor and non-motor symptoms. Among the latter are deficits in matching, identification, and recognition of emotional facial expressions. On one hand, this deficit has been attributed to a dysfunction in emotion processing. Another explanation (which does not exclude the former) links this deficit with reduced facial expressiveness in these patients, which prevents them from properly understanding or embodying emotions. To disentangle the specific contribution of emotion comprehension and that of facial expression processing in PD's observed deficit with emotions we performed two experiments on non-emotional facial expressions. In Experiment 1, a group of PD patients and a group of Healthy Controls (HC) underwent a task of non-emotional expression recognition in faces of different identity and a task of identity recognition in faces with different expression. No differences were observed between the two groups in accuracies. In Experiment 2, PD patients and Healthy Controls underwent a task where they had to recognize the identity of faces encoded through a non-emotional facial expression, through a rigid head movement, or as neutral. Again, no group differences were observed. In none of the two experiments hypomimia scores had a specific effect on expression processing. We conclude that in PD patients the observed impairment with emotional expressions is likely due to a specific deficit for emotions to a greater extent than for facial expressivity processing.
Date 2024 Mar 15
Language eng
License © 2024 The Authors.
Extra Place: England
Volume 10
Pages e26860
Publication Heliyon
DOI 10.1016/j.heliyon.2024.e26860

Issue 5
Journal Abbr Heliyon
ISSN 2405-8440
PMID 38463872
PMCID PMC10923660
Date Added 6.7.2025, 19:09:35
Modified 5.9.2025, 14:36:46

Notes:

Not Included: not on SC
Tags: EXCLUDED

Facial expressions recognition and discrimination in Parkinson's disease.

Item Type Journal Article
Author Giulia Mattavelli
Author Edoardo Barvas
Author Chiara Longo
Author Francesca Zappini
Author Donatella Ottaviani
Author Maria Chiara Malaguti
Author Maria Pellegrini
Author Costanza Papagno
Abstract Emotion processing impairment is a common non-motor symptom in Parkinson's Disease (PD). Previous literature reported conflicting results concerning, in particular, the performance for different emotions, the relation with cognitive and neuropsychiatric symptoms and the affected stage of processing. This study aims at assessing emotion recognition and discrimination in PD. Recognition of six facial expressions was studied in order to clarify its relationship with motor, cognitive and neuropsychiatric symptoms. Sensitivity in discriminating happy and fearful faces was investigated to address controversial findings on impairment in early stages of emotion processing. To do so, seventy PD patients were tested with the Ekman 60 Faces test and compared with 46 neurologically unimpaired participants. Patients' performances were correlated with clinical scales and neuropsychological tests. A subsample of 25 PD patients and 25 control participants were also tested with a backward masking paradigm for sensitivity in happiness and fear discrimination. Results showed that PD patients were impaired in facial emotion recognition, especially for fearful expressions. The performance correlated with perceptual, executive and general cognitive abilities, but facial expression recognition deficits were present even in cognitively unimpaired patients. In contrast, patients' sensitivity in backward masking tasks was not reduced as compared to controls. Taken together our data demonstrate that facial emotion recognition, and fear expression in particular, is critically affected by neurodegeneration in PD and related to cognitive abilities; however, it appears before other cognitive impairments. Preserved performances in discriminating shortly presented facial expressions, suggest unimpaired early stages of emotion processing.

**Date**	2021 Mar
**Language**	eng
**License**	© 2020 The British Psychological Society.
**Extra**	Place: England
**Volume**	15
**Pages**	46-68
**Publication**	Journal of neuropsychology
**DOI**	10.1111/jnp.12209
**Issue**	1
**Journal Abbr**	J Neuropsychol
**ISSN**	1748-6653 1748-6645
**PMID**	32319735
**Date Added**	6.7.2025, 19:09:35
**Modified**	5.9.2025, 14:45:48

Notes:

Included

Sample characteristics

Size: 70 PD, 46 HC (no differences in age, education, gender)

PD-type: NA

PD-duration: M = 7.2, SD = 5.3

Medication: : on state

Hoehn-Yahr: M = 2.3, SD = 0.7, Range = 1-5

UPDRS-3: NA

Gender (male): 44 (63%)

Age: M = 67.97, SD = 8.2

Other neurological disease (tumor, stroke, etc.): MoCA had to be > 10

Other major psychopathology: NA

Origin country (or ethnicity): Italy

method observational

instruments used in order to quantify the variables

Social cognition aspect: Emotion recognition

Name of the task: Ekman 60 faces test

Type of stimulus [face/voice etc., Ekman faces/other etc.]: Ekman faces (6 emotions; surprise, happiness, fear, disgust, anger, sadness)

Task condition: Select correct emotion (6-way forced choice)

Operationalization: Accuracy (currect answers)

Main findings related to the review's scope

The ANOVA showed significant effects of Emotion, Group, and Emotion X Group interaction. PD patients were overall less accurate than healthy controls

Post hoc tests for the Emotion by Group interaction revealed that PD patients' accuracy was lower than control group in all emotions (all ps < .01) but happiness (p = .45).

Tags: emotion recognition, behavioral

Facial feedback and autonomic responsiveness reflect impaired emotional processing in Parkinson's Disease.

**Item Type**	Journal Article
**Author**	Michela Balconi
**Author**	Francesca Pala
**Author**	Rosa Manenti
**Author**	Michela Brambilla
**Author**	Chiara Cobelli
**Author**	Sandra Rosini
**Author**	Alberto Benussi
**Author**	Alessandro Padovani
**Author**	Barbara Borroni
**Author**	Maria Cotelli
**Abstract**	Emotional deficits are part of the non-motor features of Parkinson's disease but few attention has been paid to specific aspects such as subjective emotional experience and autonomic responses. This study aimed to investigate the mechanisms of emotional recognition in Parkinson's Disease (PD) using the following levels: explicit evaluation of emotions (Self-Assessment Manikin) and implicit reactivity (Skin Conductance Response; electromyographic measure of facial feedback of the zygomaticus and corrugator muscles). 20 PD Patients and 34 healthy controls were required to observe and evaluate affective pictures during physiological parameters recording. In PD, the appraisal process on both valence and arousal features of emotional cues were preserved, but we found significant impairment in autonomic responses. Specifically, in comparison to healthy controls, PD patients revealed lower Skin Conductance Response values to negative and high arousing emotional stimuli. In addition, the electromyographic measures showed defective responses exclusively limited to negative and high arousing emotional category: PD did not show increasing of corrugator activity in response to negative emotions as happened in heathy controls. PD subjects inadequately respond to the emotional categories which were considered more "salient": they had preserved appraisal process, but impaired automatic ability to distinguish between different emotional contexts.
**Date**	2016 Aug 11
**Language**	eng
**Extra**	Place: England
**Volume**	6
**Pages**	31453
**Publication**	Scientific reports
**DOI**	10.1038/srep31453
**Journal Abbr**	Sci Rep
**ISSN**	2045-2322
**PMID**	27509848
**PMCID**	PMC4980588
**Date Added**	6.7.2025, 19:09:38
**Modified**	5.9.2025, 14:27:29

Notes:

Not Included: not on SC

Tags: EXCLUDED

Failing as doorman and disc jockey at the same time: Amygdalar dysfunction in Parkinson's disease

**Item Type**	Journal Article
**Author**	Nico J. Diederich
**Author**	Jennifer G. Goldman
**Author**	Glenn T. Stebbins
**Author**	Christopher G. Goetz
**Abstract**	In Braak's model of ascending degeneration in Parkinson's disease (PD), involvement of the amygdala occurs simultaneously with substantia nigra degeneration. However, the clinical manifestations of amygdalar involvement in PD have not been fully delineated. Considered a multitask manager, the amygdala is a densely connected hub, coordinating and integrating tasks ranging from prompt, multisensorial emotion recognition to adequate emotional responses and emotional tuning of memories. Although phylogenetically predisposed to handle fear, the amygdala handles both aversive and positive emotional inputs. In PD, neuropathological and in vivo studies suggest primarily amygdalar hypofunction. However, as dopamine acts as an inverted U-shaped amygdalar modulator, medication-induced hyperactivity of the amygdala can occur. We propose that amygdalar (network) dysfunction contributes to reduced recognition of negative emotional face expressions, impaired theory of mind, reactive hypomimia, and impaired decision making. Similarly, impulse control disorders in predisposed individuals, hallucinations, anxiety, and panic attacks may be related to amygdalar dysfunction. When available, we discuss amygdala-independent trigger mechanisms of these symptoms. Although dopaminergic agents have mostly an activation effect on amygdalar function, adaptive and compensatory network changes may occur as well, but these have not been sufficiently explored. In conclusion, our model of amygdalar involvement brings together several elements of Parkinson's disease phenomenology heretofore left unexplained and provides a framework for testable hypotheses in patients during life and in autopsy analyses. (c) 2015 International Parkinson and Movement Disorder Society
**Date**	2016-01
**Language**	English
**Extra**	Place: 111 RIVER ST, HOBOKEN 07030-5774, NJ USA Type: Review
**Volume**	31
**Publisher**	WILEY
**Pages**	11-22
**Publication**	MOVEMENT DISORDERS
**DOI**	10.1002/mds.26460
**Issue**	1
**ISSN**	0885-3185
**Date Added**	14.7.2025, 14:50:36
**Modified**	5.9.2025, 14:33:33

Notes:

Not Included: not a systematic review

Tags: EXCLUDED

Fear recognition is impaired by subthalamic nucleus stimulation in Parkinson's disease.

**Item Type**	Journal Article
**Author**	Isabelle Biseul
**Author**	Paul Sauleau
**Author**	Claire Haegelen
**Author**	Pascale Trebon
**Author**	Dominique Drapier
**Author**	Sylvie Raoul
**Author**	Sophie Drapier
**Author**	François Lallement
**Author**	Isabelle Rivier
**Author**	Youenn Lajat
**Author**	Marc Verin
**Abstract**	Behavioural disturbances such as disorders of mood, apathy or indifference are often observed in Parkinson's disease (PD) patients with chronic high frequency deep brain stimulation of subthalamic nucleus (STN DBS). Neuropsychological modifications causing these adverse events induced by STN DBS remain unknown, even if limbic disturbances are hypothesised. The limbic system supports neural circuits processing emotional information. The aim of this work is to evaluate changes of emotional recognition in PD patients induced by STN DBS. Thirty PD patients were assessed using a computerised paradigm of recognition of emotional facial expressions [Ekman, P., & Friesen, W. V. (1976). Pictures of facial affect. Palo Alto, CA: Consulting Psychologists Press], 15 before STN DBS and 15 after. The two patients groups were compared to a group of 15 healthy control subjects. One series of 55 pictures of emotional facial expressions was presented to each patient. Patients had to classify the pictures according to seven basic emotions (happiness, sadness, fear, surprise, disgust, anger and no emotion). The intact ability to percept faces was firstly assured using the Benton Recognition Test. Recognition of fear expressions was significantly and selectively reduced in the post-operative group in comparison to both pre-operative and control groups. Our results demonstrate for the first time a selective reduction of recognition of facial expressions of fear by STN DBS. This impairment could be the first neuropsychological marker of a more general limbic dysfunction, thought to be responsible for the behavioural disorders reported after STN DBS.
**Date**	2005
**Language**	eng
**Extra**	Place: England
**Volume**	43
**Pages**	1054-1059
**Publication**	Neuropsychologia
**DOI**	10.1016/j.neuropsychologia.2004.10.006
**Issue**	7
**Journal Abbr**	Neuropsychologia
**ISSN**	0028-3932
**PMID**	15769491
**Date Added**	6.7.2025, 19:09:39

Modified 5.9.2025, 14:28:52

Notes:

Not Included: no HC, only pre-post DBS

Tags: EXCLUDED

Freezing of gait and affective theory of mind in Parkinson disease.

Item Type Journal Article

Author Alessia Raffo De Ferrari
Author Giovanna Lagravinese
Author Elisa Pelosin
Author Matteo Pardini
Author Carlo Serrati
Author Giovanni Abbruzzese
Author Laura Avanzino

Abstract BACKGROUND: Affective "Theory of Mind" (ToM) is the specific ability to represent own and others' emotional states and feelings. Several studies examined affective ToM ability in patients with Parkinson's disease (PD), using the "Reading the Mind in the Eyes test" (RMET). However, there has been no agreement as to whether or not affective ToM ability is impaired in PD and such discrepancy may be due to the heterogeneous clinical presentation of PD. Affective disturbance has been linked to the akinetic-rigid form of PD and to gait disturbances, like freezing of gait (FOG). Particularly, FOG has been associated with dysfunction in striatum ability of processing affective inputs. Here we hypothesized that the presence of FOG can be associated with impaired affective ToM ability in PD patients. METHODS: We evaluated ToM by means of RMET and executive functions using the Tower Of London (ToL) test in 29 PD patients (15 with FOG and 14 without FOG) and 19 healthy age-matched subjects. RESULTS: Our results showed that affective ToM is abnormal in PD patients, compared to healthy subjects and that it is more impaired in patients with FOG than in patients without FOG. Further, PD patients with FOG performed worse than PD patients without FOG on the ToL test. CONCLUSION: The affective aspects of ToM can be associated to FOG in patients with PD, thus supporting the idea that FOG is caused by a complex interplay between motor, cognitive and affective factors, rather than being a pure motor problem.

Date 2015 May
Language eng
License Copyright © 2015 Elsevier Ltd. All rights reserved.
Extra Place: England
Volume 21
Pages 509-513
Publication Parkinsonism & related disorders
DOI 10.1016/j.parkreldis.2015.02.023
Issue 5
Journal Abbr Parkinsonism Relat Disord
ISSN 1873-5126 1353-8020

PMID 25772323
Date Added 6.7.2025, 19:09:40
Modified 5.9.2025, 14:52:27

Notes:

Included

Sample characteristics

Size: 14 PD-FOG- (no freezing of gate), 15 PD-FOG+ (freezing of gait), 19 HC (age matched)

PD-type: Idiopathic PD

PD-duration: PD-FOG-: M = 7.9, SD = 3.5; PD-FOG+: M = 10.2, SD = 6.3

Medication: In ON during Assessment

Hoehn-Yahr: < 4; PD-FOG-: M = 2.4, Sd 0 0.5, PD-FOG+: M = 2.6, SD = 0.5

UPDRS-3: PD-FOG-: M = 30.8, SD = 9.5; PD-FOG+: M = 31.00, SD = 9.5

Gender (male): They recorded it, but I dont' see it reported.

Age: PD-FOG-: M = 71, SD = 4.3; PD-FOG+: M = 72, Sd = 4.5

Other neurological disease (tumor, stroke, etc.): None (at least for HC)

Other major psychopathology: None (at least for HC)

Origin country (or ethnicity): Italy

method behavioural

instruments used in order to quantify the variables

Social cognition aspect: affective ToM

Name of the task: Reading the Min in the Eyes Test (RMET)

Type of stimulus [face/voice etc., Ekman faces/other etc.]: f 36 photographs, randomly presented, of the eye region of a Caucasian actor (19 actors and 17 actresses).

Task condition: 4way forced choice

Operationalization: percentage of correct answers

Main findings related to the review's scope

For the Reading in the Mind Eyes Test, a significant main effect of group (c2 (2) = 17.98, p = 0.0001).

Further examination indicated that PD patients, both PD FOG+ and PD FOG-, performed significantly worse than healthy subjects (PD FOG+ vs HS: U = 21, p = 0.00002; PD FOG- vs HS: U= 80.50, p = 0.045), and that PD FOG+ performed significantly worse than PD FOG- (U = 58.50, p = 0.04)

PD FOG+ subjects performed below the proposed 2 SD cut off compared to healthy subjects

Tags: ToM, behavioral

Functional alterations in resting-state networks for Theory of Mind in Parkinson's disease.

**Item Type**	Journal Article
**Author**	Giuseppe Rabini
**Author**	Giulia Funghi
**Author**	Claudia Meli
**Author**	Enrica Pierotti
**Author**	Francesca Saviola
**Author**	Jorge Jovicich
**Author**	Alessandra Dodich
**Author**	Costanza Papagno
**Author**	Luca Turella
**Abstract**	In Parkinson's disease (PD), impairment of Theory of Mind (ToM) has recently attracted an increasing number of neuroscientific investigations. If and how functional connectivity of the ToM network is altered in PD is still an open question. First, we explored whether ToM network connectivity shows potential PD-specific functional alterations when compared to healthy controls (HC). Second, we tested the role of the duration of PD in the evolution of functional alterations in the ToM network. Between-group connectivity alterations were computed adopting resting-state functional magnetic resonance imaging (rs-fMRI) data of four groups: PD patients with short disease duration (PD-1, n = 72); PD patients with long disease duration (PD-2, n = 22); healthy controls for PD-1 (HC-1, n = 69); healthy controls for PD-2 (HC-2, n = 22). We explored connectivity differences in the ToM network within and between its three subnetworks: Affective, Cognitive and Core. PD-1 presented a global pattern of decreased functional connectivity within the ToM network, compared to HC-1. The alterations mainly involved the Cognitive and Affective ToM subnetworks and their reciprocal connections. PD-2-those with longer disease duration-showed an increased connectivity spanning the entire ToM network, albeit less consistently in the Core ToM network, compared to both the PD-1 and the HC-2 groups. Functional connectivity within the ToM network is altered in PD. The alterations follow a graded pattern, with decreased connectivity at short disease duration, which broadens to a generalized increase with longer disease duration. The alterations involve both the Cognitive and Affective subnetworks of ToM.
**Date**	2024 Mar
**Language**	eng
**License**	© 2023 The Authors. European Journal of Neuroscience published by Federation of European Neuroscience Societies and John Wiley & Sons Ltd.
**Extra**	Place: France
**Volume**	59
**Pages**	1213-1226
**Publication**	The European journal of neuroscience
**DOI**	10.1111/ejn.16145
**Issue**	6
**Journal Abbr**	Eur J Neurosci
**ISSN**	1460-9568 0953-816X
**PMID**	37670685
**Date Added**	6.7.2025, 19:09:41
**Modified**	5.9.2025, 14:52:21

Notes:

Included

Sample characteristics

Size: 72 PD1 (short disease duration), 22 PD2 (long disease duration), 69 HC1, 22 HC2

PD-type: NA

PD-duration: PD1: M = 2.11, SD = 1.46; PD2: M = 7.43, SD = 5.01

Medication: ON state

Hoehn-Yahr: PD1: M = 1.64, SD = 0.54; PD2: M = 1.70, SD = 0.56

UPDRS-3: NA

Gender (male): PD1: 52 (72%), PD2: 12 (60%)

Age: PD1: M = 67.10, SD = 5.71

Other neurological disease (tumor, stroke, etc.): Na

Other major psychopathology: NA

Origin country (or ethnicity): USA, Europe, Australia

method Imaging

paradigm = resting-state fMRI; no explicit task-based ToM measure during scanning.

ROI-to-ROI connectivity analysis was computed (Fisher's z-transformed pairwise Pearson's correlation coefficients, retaining both positive and negative values), obtaining a symmetric matrix of connectivity values for each participant."
"We directly compared the connectivity pattern of PD-1 versus HC-1, PD-1 versus PD-2 and PD-2 versus HC-2. We considered the ROI-to-ROI connections following two approaches: (1) by adopting 'network overall degree of connectivity' ... and (2) by directly comparing the ROI-to-ROI connectivity values within all regions of the three sub-networks.

ROI-selection

we considered a recent neuroanatomical model of ToM (Abu-Akel & Shamay-Tsoory, **2011**) and selected the following regions of interest for the three subnetworks from the Brainnetome Atlas (**https://atlas.brainnetome.org/publications.html**) (Fan et al., **2016**):

1. cToM (cognitive ToM): dmPFC, dlPFC, dACC, dATL, caudate, putamen.

2. aToM (Affective ToM): OFC, vmPFC, vACC, vATL, liFC, Amyg, accumbens.

3. coreToM (appears to support both affective and cognitive circuits): IPL (BA 39,40), pSTS and PreCun.

We directly compared the connectivity pattern of PD-1 versus HC-1, PD-1 versus PD-2 and PD-2 versus HC-2. We considered the ROI-to-ROI connections following two approaches: (1) by adopting 'network overall degree of connectivity' (a sum of connectivity values) as a summary measure of connectivity within and between the cToM, aToM and coreToM sub-networks and (2) by directly comparing the ROI-to-ROI connectivity values within all regions of the three sub-networks.

Main findings related to the review's scope

Differences in overall degree of connectivity within the ToM network

PD1 vs HC1

significant difference within the cToM (right: $p = 0.003$; left: $p = 0.005$), between the cToM and aToM (right: $p < 0.001$; left: $p < 0.001$) and between the coreToM and the cToM (right: $p = 0.007$; left: $p = 0.006$) in both hemispheres, and within the aToM in the left hemisphere ($p = 0.008$).

When considering cross-hemisphere connections, we saw a significant difference, mainly between networks, and in particular, between right cToM and left aToM ($p = 0.005$), between right aToM and left cToM ($p < 0.001$) and between right CoreToM and left cToM ($p = 0.01$). Significant differences were due to higher connectivity strength in HC-1

PD2 vs PD1

Overall, PD-1—with shorter disease duration— showed a lower level of connectivity within the ToM network, compared to PD-2, with longer disease duration.

In particular, we observed a significant difference within the cToM (right: $p < 0.001$; left: $p < 0.001$), within the aToM (right: $p < 0.001$; left: $p < 0.001$), between the cToM and aToM (right: $p < 0.001$; left: $p < 0.001$) and between the cToM and the CoreToM (right: $p = 0.038$; left: $p = 0.007$) in both hemispheres. In the left hemisphere, also connectivity differences between the aToM and the CoreToM were significant ($p = 0.009$). Looking at the betweenhemisphere connections, results showed a significant difference within the cToM ($p < 0.001$); within the aToM ($p < 0.001$); between the right cToM and left aToM ($p < 0.001$), between the right aToM and left cToM ($p = 0.009$) and between the right cToM and left CoreToM ($p = 0.009$).

Notably, a significant effect of interaction involving the sex grouping factor (Group Sex) was present within the cToM within the right and left hemisphere separately and between the nodes of the cToM across hemispheres

PD2 vs HC2

Overall, similarly to the comparison with the shortduration group (PD-1), the PD-2 group showed increased connectivity within the ToM network

Specifically, we saw a significant group difference within the aToM (right, $p < 0.001$; left, $p = 0.007$), within the cToM (right, $p = 0.008$; left, $p = 0.023$) and between the cToM and aToM (right, $p < 0.001$; left, $p < 0.001$) in both hemispheres and between the aToM and the CoreToM in the left hemisphere ($p = 0.023$). Significant group differences in the connections between hemispheres were present between right and left aToM ($p < 0.001$), between right and left cToM ($p = 0.033$), between right aToM and left cToM ($p = 0.0046$) and between left aToM and right cToM ($p < 0.001$)

ROI-to-ROI connectivity differences within the ToM network

Looking at the ROI-to-ROI connectivity values, there is a general trend for positive connectivity in the ToM network. However, several exceptions are present in the pairwise ROI-to-ROI connections, with distinct negative connections, particularly in the PD-1, the short-disease-duration group

PD1 vs HC1

Significant differences were particularly present within the cToM, between the cToM and aToM and between the coreToM regions and the cToM—in both hemispheres

Overall, significant differences between groups were due to lower connectivity values in PD-1.

In particular, the connections within the cToM regions and between the cToM and the aToM appeared to be almost unaltered within the two hemispheres.

Within each hemisphere, dmPFC, dlPFC and caudate were involved in several connectivity differences between groups. PD-1 showed significantly higher connectivity than HC-1 in between-hemisphere connections involving the dmPFC and dlPFC. The same regions presented higher connectivity within each hemisphere separately

PD1 vs PD2

There was a widespread difference between groups, both within and between the different subnetworks of the ToM. Interestingly, the connections involving the coreToM regions seem to be the least different between the two groups of PD. The significant differences predominantly highlight an increased connectivity in the long-disease-duration group compared to the shortdisease-duration group. Considering the connections within each hemisphere, caudate, dmPFC, dlPFC, iFC, vmPFC and v/dATL were particularly involved in significant differences. In the right hemisphere, Amyg, OFC and accumbens were associated with differential connectivity between groups.

PD2 vs HC2

Group differences were less widespread than in the preceding comparisons. Significant differences arose mainly in the right hemisphere, with significantly different connections involving OFc, vmPFC, dlPFC, dmPFC and putamen. In the left hemisphere, regions particularly involved in significant differences were OFC, in relation to PFC and IPL, and iFC. In between-hemisphere connections, ROI-to-ROI connectivity differences were heavily present within the aToM and in the betweennetwork connections, again engaging regions such as OFC, iFC, Amyg and putamen. We did not report any significant effect of interaction between group and sex at ROI-to-ROI level.

Tags: ToM, Imaging

Functional brain changes in Parkinson's disease: a whole brain ALE study

Item Type Journal Article
Author Lihua Gu
Author Hao Shu
Author Hui Xu
Author Yanjuan Wang
Abstract Background Resting-state functional magnetic resonance imaging (rs-fMRI) was widely used as an effective tool in the diagnosis of neurodegenerative diseases. However, prior rs-fMRI studies reported inconsistent results for comparison between Parkinson's disease (PD) and healthy controls (HC). Methods We searched studies published before December 2021 in databases (PubMed, Web of Science, and Google Scholar). An activation likelihood estimation (ALE) meta-analysis was made for functional changes in PD. Results The study finally included 25 studies (including 973 PD patients and 766 HC). PD patients showed reduced amplitude of low frequency fluctuations (ALFF) in the left superior temporal gyrus (STG), the left superior frontal gyrus (SFG), the left medial frontal gyrus (MFG), the left precuneus (PCUN), and the right lentiform nucleus, compared to HC. PD patients showed increased ALFF in the right SFG, the left superior parietal lobule (SPL), the left STG, the right fusiform gyrus, the left inferior temporal gyrus (ITG), and the right parahippocampal gyrus (PHG), compared to HC. PD patients showed reduced regional homogeneity (ReHo) in the right declive, the right MFG, the left culmen,

and the left thalamus, compared to HC. PD patients showed increased ReHo in the right SFG, compared to HC. Additionally, PD patients showed reduced functional connectivity (FC) in the right posterior cingulate (PCG), compared to HC. Conclusions The present ALE analysis has confirmed functional deficits in motor-, emotion-, and cognition-related regions in PD. Deficits in these regions in rs-fMRI studies could play a role in early diagnosis of PD.

Date 2022-10
Language English
Extra Place: VIA DECEMBRIO, 28, MILAN, 20137, ITALY Type: Article
Volume 43
Publisher SPRINGER-VERLAG ITALIA SRL
Pages 5909-5916
Publication NEUROLOGICAL SCIENCES
DOI 10.1007/s10072-022-06272-9
Issue 10
ISSN 1590-1874
Date Added 14.7.2025, 14:50:29
Modified 5.9.2025, 14:37:16

Notes:

Not Included: not on SC
Tags: EXCLUDED

Gender Differences in Parkinson's Disease: Clinical Characteristics and Cognition

Item Type Journal Article
Author Ivy N. Miller
Author Alice Cronin-Golomb
Abstract More men than women are diagnosed with Parkinson's disease (PD), and a number of gender differences have been documented in this disorder. Examples of clinical characteristics that appear in men more often than women include rigidity and rapid eye movement behavior disorder, whereas more women than men exhibit dyskinesias and depression. Differences between men and women in cognition have not been extensively examined, though there are reports of deficits in men in aspects of cognition that contribute to activities of daily living, in verbal fluency, and in the recognition of facial emotion, and deficits in women in visuospatial cognition. Side of disease onset may interact with gender to affect cognitive abilities. One possible source of male-female differences in the clinical and cognitive characteristics of PD is the effect of estrogen on dopaminergic neurons and pathways in the brain. This effect is not yet understood, as insight into how the fluctuation of estrogen over the lifetime affects the brain is currently limited. Further attention to this area of research will be important for accurate assessment and better management of PD. Attention should also be directed to multiple covariates that may affect clinical characteristics and cognition. Knowledge about differences in the presentation of PD symptoms in men and women and about the pathophysiology underlying those differences may

enhance the accuracy and effectiveness of clinical assessment and treatment of the disease. (C) 2010 Movement Disorder Society

Date 2010-12-15
Language English
Extra Place: 111 RIVER ST, HOBOKEN 07030-5774, NJ USA Type: Review
Volume 25
Publisher WILEY
Pages 2695-2703
Publication MOVEMENT DISORDERS
DOI 10.1002/mds.23388
Issue 16
ISSN 0885-3185
Date Added 14.7.2025, 14:50:41
Modified 14.7.2025, 14:50:41

Notes:

Not included: No Systematic Review-Article

Tags: EXCLUDED

Gray matter volume alterations in de novo Parkinson's disease: A mediational role in the interplay between sleep quality and anxiety.

Item Type Journal Article

Author Guixiang He
Author Xiaofang Huang
Author Haihua Sun
Author Yi Xing
Author Siyu Gu
Author Jingru Ren
Author Weiguo Liu
Author Ming Lu

Abstract OBJECTIVE: Parkinson's disease (PD) is increasingly recognized for its non-motor symptoms, among which emotional disturbances and sleep disorders frequently co-occur. The commonality of neuroanatomical underpinnings for these symptoms is not fully understood. This study is intended to investigate the differences in gray matter volume (GMV) between PD patients with anxiety (A-PD) and those without anxiety (NA-PD). Additionally, it seeks to uncover the interplay between GMV variations and the manifestations of anxiety and sleep quality. METHODS: A total of 37 A-PD patients, 43 NA-PD patients, and 36 healthy controls (HCs) were recruited, all of whom underwent voxel-based morphometry (VBM) analysis. Group differences in GMV were assessed using analysis of covariance (ANCOVA). Partial correlation between GMV, anxiety symptom, and sleep quality were analyzed. Mediation analysis explored the mediating role of the volume of GMV-distinct brain regions on the relationship between sleep quality and anxiety within the PD patient cohort. RESULTS: A-PD patients showed significantly lower GMV in the fusiform gyrus

(FG) and right inferior temporal gyrus (ITG) compared to HCs and NA-PD patients. GMV in these regions correlated negatively with Hamilton Anxiety Rating Scale (HAMA) scores (right ITG: r = -0.690, p < 0.001; left FG: r = -0.509, p < 0.001; right FG: r = -0.576, p < 0.001) and positively with sleep quality in PD patients (right ITG: r = 0.592, p < 0.001; left FG: r = 0.356, p = 0.001; right FG: r = 0.470, p < 0.001). Mediation analysis revealed that GMV in the FG and right ITG mediated the relationship between sleep quality and anxiety symptoms, with substantial effect sizes accounted for by the right ITG (25.74%) and FG (left: 11.90%, right: 15.59%). CONCLUSION: This study has shed further light on the relationship between sleep disturbances and anxiety symptoms in PD patients. Given the pivotal roles of the FG and the ITG in facial recognition and the recognition of emotion-related facial expressions, our findings indicate that compromised sleep quality, under the pathological conditions of PD, may exacerbate the reduction in GMV within these regions, impairing the recognition of emotional facial expressions and thereby intensifying anxiety symptoms.

**Date**	2024 Jul
**Language**	eng
**License**	© 2024 The Author(s). CNS Neuroscience & Therapeutics published by John Wiley & Sons Ltd.
**Extra**	Place: England
**Volume**	30
**Pages**	e14867
**Publication**	CNS neuroscience & therapeutics
**DOI**	10.1111/cns.14867
**Issue**	7
**Journal Abbr**	CNS Neurosci Ther
**ISSN**	1755-5949 1755-5930
**PMID**	39031989
**PMCID**	PMC11259571
**Date Added**	6.7.2025, 19:09:36
**Modified**	5.9.2025, 14:38:39

Notes:

Not Included: Not on SC

Tags: EXCLUDED

Grey matter abnormalities in Parkinson's disease: a voxel-wise meta-analysis.

**Item Type**	Journal Article
**Author**	X. Xu
**Author**	Q. Han
**Author**	J. Lin
**Author**	L. Wang
**Author**	F. Wu
**Author**	H. Shang

Abstract BACKGROUND AND PURPOSE: The pathophysiology of Parkinson's disease (PD) remains unclear. Voxel-based morphometry (VBM) detects local structural differences in brain tissue such as grey matter volume (GMV) between groups, which is helpful in understanding the pathophysiology of PD. Published VBM studies of GMV changes in PD have shown inconsistent results. Therefore, a voxel-wise meta-analysis of VBM studies was conducted to detect consistent GMV changes in PD. METHODS: The published literature was searched comparing whole-brain GMV between PD patients and healthy controls (HCs) using VBM. Coordinates were extracted for the clusters of significant GMV differences between PD patients and HCs. The meta-analysis was performed by seed-based d mapping software. RESULTS: A total of 63 studies with 2867 PD patients and 1990 HCs were included. Significant GMV reductions in some brain regions were detected in PD patients, which were involved in the basal ganglia, theory of mind, vocal and visual networks. These findings remained largely unchanged in the jackknife sensitivity analysis, and no significant heterogeneity or publication bias was detected. CONCLUSIONS: Parkinson's disease patients have GMV reductions in a number of brain regions involved in specific networks. These findings provide morphological evidence for the pathophysiology of PD.

Date 2020 Apr
Language eng
License © 2019 European Academy of Neurology.
Extra Place: England
Volume 27
Pages 653-659
Publication European journal of neurology
DOI 10.1111/ene.14132
Issue 4
Journal Abbr Eur J Neurol
ISSN 1468-1331 1351-5101
PMID 31770481
Date Added 6.7.2025, 19:09:41
Modified 5.9.2025, 15:01:19

Notes:

Not included: not on SC

Tags: EXCLUDED

Hearts and Minds: Emotion Recognition and Mentalizing in Parkinson's Disease and Progressive Supranuclear Palsy.

Item Type Journal Article
Author Marina I. Martins
Author Francisco E. C. Cardoso
Author Paulo Caramelli
Author Luciano I. Mariano

Author Natalia P. Rocha
Author Antônio Jaeger
Author Antônio L. Teixeira
Author Vítor Tumas
Author Sarah T. Camargos
Author Leonardo C. de Souza
Abstract OBJECTIVE: There are scarce data comparing Parkinson's disease (PD) and Progressive Supranuclear Palsy (PSP) in social cognition (SC). We aimed to compare patients with PSP and PD in SC. METHODS: We included three groups: PD (n = 18), PSP (n = 20) and controls (n = 23). Participants underwent neuropsychological exams, including the mini-version of the Social and Emotional Assessment, which is composed of the facial emotion recognition test (FERT) and the modified faux-pas (mFP) test, which assesses Theory of Mind (ToM). RESULTS: Patients with PD scored lower than controls in the FERT, but not in the mFP test. Patients with PSP performed worse than controls in both the mFP and FERT. PD and PSP groups did not differ in the FERT, but PSP performed worse than PD in the mFP test. The mFP test distinguished PSP from PD with 89% accuracy. CONCLUSION: The assessment of ToM may contribute to the differentiation between PD and PSP.
Date 2024 May 21
Language eng
License © The Author(s) 2023. Published by Oxford University Press. All rights reserved. For permissions, please e-mail: journals.permissions@oup.com.
Extra Place: United States
Volume 39
Pages 516-522
Publication Archives of clinical neuropsychology : the official journal of the National Academy of Neuropsychologists
DOI 10.1093/arclin/acad081
Issue 4
Journal Abbr Arch Clin Neuropsychol
ISSN 1873-5843 0887-6177
PMID 37856362
Date Added 6.7.2025, 19:09:34
Modified 5.9.2025, 14:45:30

Notes:

Included

Sample characteristics

Size: 18 PD, 20 PSP (progressive supranuclear palsy), 23 HC

PD-type: NA

PD-duration: M = 9.6, Sd = 6.2

Medication: NA

Hoehn-Yahr: Range = 1-3, M = 2.0, SD = 1.1

UPDRS-3: NA

Gender (male): 9 (50%)

Age: M = 66.2, SD = 9.8

Other neurological disease (tumor, stroke, etc.): none

Other major psychopathology: none

Origin country (or ethnicity): Brazil

method behavioural

instruments used in order to quantify the variables

Mini version of the Social cognition and emotional assessment (Mini-SEA)

Social cognition aspect: Facial Emotion Recognition

Name of the task: Facial Emotion Recognition Test (FERT)

Type of stimulus [face/voice etc., Ekman faces/other etc.]: 35 images of human faces.

Task condition: seven different emotions (fear, disgust, surprise, anger, happiness, sadness, neutral)

Operationalization: correct answers.

Social cognition aspect: ToM

Name of the task: Modified Faux-pas

Type of stimulus [face/voice etc., Ekman faces/other etc.]: 10 short stories describing a scene in which one character either commits (in five stories) or does not commit (in five others) a social faux pas (an inadequate action that violates social rules).

Task condition: standardized questions exploring how the participant interprets the social interactions depicted in the story

Operationalization: two components of the test were distinguished: the recognition of social norms violation (the sum of scores for questions 1, 2, and 3); and the mentalizing (the sum of scores for questions 4, 5, and 6). The scores on the mFP and on the FERT are converted to scores out of 15

The total Mini-SEA score (/30) is calculated by adding the FERT (/15) and mFP Test (/15) scores, with higher scores indicating better performance.

Main findings related to the review's scope

PD had impaired performance on the FERT, compared to controls. No sig dif between PD and PSP

Patients with PD did not differ from controls on ToM scores (mFP total score, recognition of social norms violation, and mentalizing)

Tags: ToM, Emotion Recognition, behavioral

Hemispheric asymmetries and prosodic emotion recognition deficits in Parkinson's disease.

Item Type Journal Article

Author Maria I. Ventura
Author Kathleen Baynes
Author Karen A. Sigvardt
Author April M. Unruh
Author Sarah S. Acklin
Author Heidi E. Kirsch
Author Elizabeth A. Disbrow

Abstract While Parkinson's disease (PD) has traditionally been described as a movement disorder, there is growing evidence of cognitive and social deficits associated with the disease. However, few studies have looked at multi-modal social cognitive deficits in patients with PD. We studied lateralization of both prosodic and facial emotion recognition (the ability to recognize emotional valence from either tone of voice or from facial expressions) in PD. The Comprehensive Affect Testing System (CATS) is a well-validated test of human emotion processing that has been used to study emotion recognition in several major clinical populations, but never before in PD. We administered an abbreviated version of CATS (CATS-A) to 24 medicated PD participants and 12 age-matched controls. PD participants were divided into two groups, based on side of symptom onset and unilateral motor symptom severity: left-affected (N = 12) or right-affected PD participants (N = 12). CATS-A is a computer-based button press task with eight subtests relevant to prosodic and facial emotion recognition. Left-affected PD participants with inferred predominant right-hemisphere pathology were expected to have difficulty with prosodic emotion recognition since there is evidence that the processing of prosodic information is right-hemisphere dominant. We found that facial emotion recognition was preserved in the PD group, however, left-affected PD participants had specific impairment in prosodic emotion recognition, especially for sadness. Selective deficits in prosodic emotion recognition suggests that (1) hemispheric effects in emotion recognition may contribute to the impairment of emotional communication in a subset of people with PD and (2) the coordination of neural networks needed to decipher temporally complex social cues may be specifically disrupted in PD.

Date 2012 Jul
Language eng

License Copyright © 2012 Elsevier Ltd. All rights reserved.

**Extra**	Place: England
**Volume**	50
**Pages**	1936-1945
**Publication**	Neuropsychologia
**DOI**	10.1016/j.neuropsychologia.2012.04.018
**Issue**	8
**Journal Abbr**	Neuropsychologia
**ISSN**	1873-3514 0028-3932
**PMID**	22564479
**Date Added**	6.7.2025, 19:09:35
**Modified**	5.9.2025, 14:59:49

Notes:

Included

Sample characteristics

Size: 24 PD -> 12 LPD (left affected), 12 RPD (right affected), 12 HC (age matched)

PD-type: Idiopathic PD

PD-duration: NA

Medication: ON state

Hoehn-Yahr: LPD: M = 2, RPD: M = 2

UPDRS-3: NA

Gender (male): LPD: 4 (33%), RPD: 6 (50%)

Age: LPD: M = 71, SD = 4.7; RPD: M = 66, SD = 4.2

Other neurological disease (tumor, stroke, etc.): none

Other major psychopathology: none

Origin country (or ethnicity): USA

method behavioral

instruments used in order to quantify the variables

Social cognition aspect: Emotion recognition

Name of the task: Abbreviated Comprehensive Affect Testing System (CATS-A) (Battery with multiple subtests) was used. Following, the subtests are described.

1. *Subtest 2* **Prosody discrimination**: participants heard two serially presented sentences spoken in emotional tones (happy, sad, angry, frightened or neutral) and determined if the sentences conveyed the same or different emotion. Participants selected one of two response buttons labeled "Same" or "Different."
2. *Subtest 3* **Prosody Identification**: participants heard one sentence and identified the emotion conveyed in the tone (happy, sad, angry, frightened or neutral). Participants selected one of five response buttons.
3. *Subtest 5* **Facial discrimination:** participants saw simultaneously presented black and white photos of two faces expressing emotions (happy, sad, angry, frightened or neutral) and determined if the faces they saw were expressing the same or different emotion. Participants selected one of two response buttons.
4. *Subtest 6* **Facial Identification:** participants saw one photo and were asked to identify the facial emotion expressed by the person in the picture (happy, sad, angry, frightened, surprised, disgusted or neutral). Participants selected one of seven response buttons.
5. *Subtest 7* **Face-Prosody matching:** participants heard one sentence spoken in an emotional tone (happy, sad, angry, frightened or neutral). They simultaneously viewed photos of 5 faces expressing these five emotions. Participants selected the face that expressed the emotion corresponding to the tone of voice. Participants used the mouse to select and click on the corresponding face.
6. *Subtest 8* **Stroop-like - Prosody-Sentence conflict:** participants heard a single sentence spoken in an emotional tone (happy, sad or neutral). The sentence contained conflicting prosody and meaning (e.g., "The puppies were rescued" — positive content spoken in a sad tone). Participants were instructed to ignore what was being said (i.e., the content) and instead identify emotional prosody. Participants selected one of three response buttons (in this example, sad).

averaged performance on Emotional Prosody Discrimination (**subtest 2**), Name Emotional Prosody (**subtest 3**), and Conflicting Prosody and Meaning (**subtest 8**), and referred to these combined subtests as the Composite Prosody Scale.

averaged performance on Affect Discrimination (**subtest 5**) and Name Affect (**subtest 6**), and referred to these combined subtests as the Composite Facial Scale.

Match Emotional Prosody and Face (**subtest 7**), were kept as separate subtest

Main findings related to the review's scope

accuracy:

the Composite Prosody Scale: left PD were less accurate then right PD or HC. no sig dif between right PD and HC.

the Composite Facial Scale: no sig dif

Face-Prosody matching: left PD were less accurate then right PD or HC. no sig dif between right PD and HC.

Tags: Emotion recognition, behavioral

Hemispheric specialization of the basal ganglia during vocal emotion decoding: Evidence from asymmetric Parkinson's disease and 18FDG PET

Item Type Journal Article

Author Nancy Stirnimann
Author Karim N'Diaye
Author Florence Le Jeune
Author Jean-François Houvenaghel
Author Gabriel Robert
Author Sophie Drapier
Author Dominique Drapier
Author Didier Grandjean
Author Marc Vérin
Author Julie Péron
Abstract The possible hemispheric specialization of the basal ganglia during emotional prosody (i.e., vocal emotion) processing has still to be elucidated. Coupled with affective measures and neuroimaging, Parkinson's disease offers a unique opportunity to study this question, on account of its characteristically asymmetric striatal dysfunction, which translates into predominantly contralateral motor symptoms. We investigated the cerebral metabolic bases of emotional prosody recognition in patients with Parkinson's disease with left- versus right-lateralized motor symptoms, postulating that patients with greater right hemispheric brain dysfunction have a specific impairment that correlates with the metabolic modification of a brain network known to be involved in emotional prosody. A total of 38 patients performed a validated emotional prosody recognition task and underwent a resting-state F-18 fluorodeoxyglucose PET scan, as well as clinical, motor, neuropsychological, and psychiatric assessments. Patients' performances were compared with those of 45 healthy controls. As expected, vocal emotion recognition was significantly poorer among patients with left-sided motor symptoms than among both right-sided patients and controls. There was no significant difference between right-sided patients and controls. This effect was observed for both the total score and the happiness subscore. Interestingly, regressions showed that the greater the emotional misattribution, the greater the patients' age and asymmetric motor symptom severity. Finally, at the metabolic level, positive correlations were found between the happiness recognition subscore and the metabolism of the right orbitofrontal cortex in patients with left-sided motor symptoms. A right orbitofrontal-basal ganglia coupling seems to be specifically involved in the vocal emotion recognition deficit observed in Parkinson's disease. The asymmetry of motor symptoms is thus an important clinical factor, in that it may influence the presence or severity of affective disorders in Parkinson's disease.
Date 2018
URL https://www.sciencedirect.com/science/article/pii/S0028393218303749
Volume 119
Pages 1-11
Publication Neuropsychologia
DOI https://doi.org/10.1016/j.neuropsychologia.2018.07.023
ISSN 0028-3932
Date Added 6.7.2025, 19:12:34
Modified 5.9.2025, 14:57:41

Notes:

Included

Sample characteristics

Size: 19 LDP (primarliy left-affected PD), 18 RPD (primarily right-affected PD), 45 HC (all machted for sex and age)

PD-type: NA

PD-duration: LPD M=12.85 SD=6.12; RPD M=11.79 SD-4.19

Medication: ON state

Hoehn-Yahr (on Dopa): LPD: M = 1.27, SD = 0.72; RPD: M = 1.03, SD = 0.97

UPDRS-3 (ON): LPD: M = 9, SD = 6.39; RPD: M = 8.43, SD = 5.97

Gender (male): 12 (63%) LPD and RPD each

Age: LPD: M = 56.95, SD = 8.97; RPD: M = 56.74, SD = 7.34

Other neurological disease (tumor, stroke, etc.): NA (had to be eligible for DBS)

Other major psychopathology: None

Origin country (or ethnicity): France

method behavioral

instruments used in order to quantify the variables

Social cognition aspect: emotion recognition

Name of the task: NA

Type of stimulus [face/voice etc., Ekman faces/other etc.]: 60 vocal pseudowords. The set of vocal stimuli was obtained by concatenating different syllables found in Indo-European languages so that they would be perceived of as natural utterances, with emotional intonations common to different cultures, but no semantic content. We selected utterances produced by 12 different actors (six women and six men) in a validated database

Task condition: anger, fear, happiness, sadness and neutral

Operationalization: judge the extent to which each of the different emotions was expressed, by moving a cursor along a visual analog scale ranging from Not at all to Very much. Participants rated each stimulus on six scales: one scale for each emotion featured (i.e. anger, fear, happiness and sadness) and one for neutral, plus a scale to rate the surprise emotion >> This procedure allowed us to compute two different indices:

1. a congruence index reflecting the proportion of correct responses (i.e., when the stimulus was most highly rated on the target emotion scale)
2. a discrimination index reflecting the difference between the rating on the *target* emotion scale and the averaged ratings on the five other *incorrect* emotion scales (i.e., target emotion recognition *over* the nontarget emotions)

PET imaging:

All participants underwent an FDG-PET scan in a resting state in the on-dopa state. in 2D mode, with an axial field of view of 15.2 cm and axial resolution of 4.8 mm. A 222–296 MBq injection of F-18 [redacted] was administered in a quiet, dimly lit room. A 20-min 2D scan was performed 30 min post-injection, with participants positioned at the center of the field of view.

The data were analyzed with SPM 12. All the participants' images were first spatially normalized to standard stereotactic space according to Talairach and Tournoux (Talairach and Tournoux, 1988)'s atlas. Affine transformation was performed to determine the 12 optimum parameters to register the brain to the [redacted], and subtle differences between the transformed image and the template were then removed using a nonlinear registration method. Finally, the spatially normalized images were smoothed using an isotropic 12-mm full width at half-maximum isotropic Gaussian kernel to compensate for interindividual anatomical variability and to render the imaging data more normally distributed.

Main findings related to the review's scope

For the congruence index, analyses revealed a main effect of emotion, but not of group, and no interaction effect.

For the discrimination index, analyses revealed main effects of emotion, and group, but no interaction effect.

total emotion discrimination index, LPD performed significantly more poorly than either HC or RPD, whereas there was no difference between RPD and HC

happiness discrimination index, LPD performed more poorly than either HC or RPD whereas there was no difference between RPD and HC

no significant group effect for sadness or anger

Relationship between vocal emotion recognition and PET

RPD: None of the models revealed significant clusters.

LPD: The models for the happiness congruence index and total emotion discrimination index revealed no significant clusters. For the happiness discrimination index, however, we observed positive correlations (i.e., decreased metabolic activity with decreased emotion performance) in the right orbitofrontal cortex (Brodmann area, BA 10) (see Fig. 3). No significant negative correlations (i.e., increased metabolic activity with increased emotion performance) were found.

Tags: emotion recognition, Imaging, behavioral

Hierarchical cluster analysis of multimodal imaging data identifies brain atrophy and cognitive patterns in Parkinson's disease

Item Type Journal Article
Author A. Inguanzo
Author R. Sala-Llonch
Author B. Segura
Author H. Erostarbe
Author A. Abos
Author A. Campabadal
Author C. Uribe

Author H. C. Baggio
Author Y. Compta
Author M. J. Marti
Author F. Valldeoriola
Author N. Bargallo
Author C. Junque
Abstract Background: Parkinson's disease (PD) is a heterogeneous condition. Cluster analysis based on cortical thickness has been used to define distinct patterns of brain atrophy in PD. However, the potential of other neuroimaging modalities, such as white matter (WM) fractional anisotropy (FA), which has also been demonstrated to be altered in PD, has not been investigated. Objective: We aim to characterize PD subtypes using a multimodal clustering approach based on cortical and subcortical gray matter (GM) volumes and FA measures. Methods: We included T1-weighted and diffusion-weighted MRI data from 62 PD patients and 33 healthy controls. We extracted mean GM volumes from 48 cortical and 17 subcortical regions using FSL-VBM, and the mean FA from 20 WM tracts using Tract-Based Spatial Statistics (TBSS). Hierarchical cluster analysis was performed with the PD sample using Ward's linkage method. Whole-brain voxel-wise intergroup comparisons of VBM and TBSS data were also performed using FSL. Neuropsychological and demographic statistical analyses were conducted using IBM SPSS Statistics 25.0. Results: We identified three PD subtypes, with prominent differences in GM patterns and little WM involvement. One group (n = 15) with widespread cortical and subcortical GM volume and WM FA reductions and pronounced cognitive deficits; a second group (n = 21) with only cortical atrophy limited to frontal and temporal regions and more specific neuropsychological impairment, and a third group (n = 26) without detectable atrophy or cognition impairment. Conclusion: Multimodal MRI data allows classifying PD patients into groups according to GM and WM patterns, which in turn are associated with the cognitive profile.
Date 2021-01
Language English
Extra Place: THE BOULEVARD, LANGFORD LANE, KIDLINGTON, OXFORD OX5 1GB, OXON, ENGLAND Type: Article
Volume 82
Publisher ELSEVIER SCI LTD
Pages 16-23
Publication PARKINSONISM & RELATED DISORDERS
DOI 10.1016/j.parkreldis.2020.11.010
ISSN 1353-8020
Date Added 14.7.2025, 14:50:31
Modified 5.9.2025, 14:40:10

Notes:

Not Included: not on SC

Tags: EXCLUDED

Hypomimia May Influence the Facial Emotion Recognition Ability in Patients with Parkinson's Disease.

Item Type Journal Article
Author Yu-Han Chuang
Author Chun-Hsiang Tan
Author Hui-Chen Su
Author Chung-Yao Chien
Author Pi-Shan Sung
Author Tsung-Lin Lee
Author Rwei-Ling Yu
Abstract BACKGROUND: Hypomimia is a clinical feature of Parkinson's disease (PD). Based on the embodied simulation theory, the impairment of facial mimicry may worsen facial emotion recognition; however, the empirical results are inconclusive. OBJECTIVE: We aimed to explore the worsening of emotion recognition by hypomimia. We further explored the relationship between the hypomimia, emotion recognition, and social functioning. METHODS: A total of 114 participants were recruited. The patients with PD and normal controls (NCs) were matched for demographic characteristics. All the participants completed the Mini-Mental State Examination and the Chinese Multi-modalities Emotion Recognition Test. In addition to the above tests, the patients were assessed with the Movement Disorder Society-Unified Parkinson's Disease Rating Scale and Parkinson's Disease Social Functioning Scale (PDSFS). RESULTS: Patients with PD with hypomimia had worse recognition of disgust than NCs ($p=0.018$). The severity of hypomimia was predictive of the recognition of disgust ($\beta=-0.275$, $p=0.028$). Facial emotion recognition was predictive of the PDSFS score of PD patients ($\beta=0.433$, $p=0.001$). We also found that recognizing disgust could mediate the relationship between hypomimia and the PDSFS score ($\beta=0.264$, $p=0.045$). CONCLUSION: Patients with hypomimia had the worst disgust facial recognition. Hypomimia may affect the social function of PD patients, which is related to recognizing the expression of disgust. Emotion recognition training may improve the social function of patients with PD.
Date 2022
Language eng
Extra Place: United States
Volume 12
Pages 185-197
Publication Journal of Parkinson's disease
DOI 10.3233/JPD-212830
Issue 1
Journal Abbr J Parkinsons Dis
ISSN 1877-718X 1877-7171
PMID 34569974
Date Added 6.7.2025, 19:09:35
Modified 5.9.2025, 14:30:57

Notes:

Included

sample characteristics

size: 57 PD (15 with hypomimia [PD-MF+] and 42 without [PD-MF-]) and 57 HC matched for gender, age, and education.

Parkinson's Disease type and duration: idiopathic PD, Mduration= 6.67 SD=4.99

Medication: NA

Hoehn-Yahr: M= 1.86 SD=0.64

UPDRS-3: M= 28.37 SD=10.32

Gender (male): 36 males (63%)

averaged ages (SD, range): M= 62.84 SD=7.95

other neurological disease (tumor, stroke, etc.): None

other major psychopathology: None

origin country (or ethnicity): China

method observational

instruments used in order to quantify the variables

Social cognition aspect: emotion recognition

Name of the task: The Chinese multi-modalities emotion recognition test (CMERT)

type of stimulus [face/voice etc., Ekman faces/other etc.]: subtests 3 and 4 to explore participants' facial emotion recognition ability—the two subtests were targeted at different task types. (i.e., emotion identification and emotion discrimination tasks).

task condition: neutral, happiness, sadness, anger, disgust, fear, and surprise

operationalization: Each item of subtest 3 had one photograph conveying different emotions (i.e., neutral, happiness, sadness, anger, disgust, fear, and surprise) and seven emotion labels on the tablet screen. The participants were asked to choose one of the seven emotion labels that best fit the emotions expressed by the people in the picture. Each item of subtest 4 had one emotion label and seven faces with various emotions on the tablet screen. The participants were asked to choose one of the seven photographs conveying different emotions that best fit the emotion label. >> accuracy.

Main findings related to the review's scope

significant difference was observed in the CMERT-subtest 4-disgust. All other comparisons (total and different emotion) were not significant.

No significant differences in subtest 3.

Consistent when exmained after dividing by MF

Tags: Emotion recognition, behavioral

I spy with my little eye: The detection of changes in emotional faces and the influence of facial feedback in Parkinson disease.

Item Type Journal Article
Author Maria Kuehne
Author Laura Polotzek
Author Aiden Haghikia
Author Tino Zaehle
Author Janek S. Lobmaier
Abstract BACKGROUND AND PURPOSE: Parkinson disease (PD) is a progressive neurodegenerative disorder that affects the motor system but also involves deficits in emotional processing such as facial emotion recognition. In healthy participants, it has been shown that facial mimicry, the automatic imitation of perceived facial expressions, facilitates the interpretation of the emotional states of our counterpart. In PD patients, recent studies revealed reduced facial mimicry and consequently reduced facial feedback, suggesting that this reduction might contribute to the prominent emotion recognition deficits found in PD. METHODS: We investigated the influence of facial mimicry on facial emotion recognition. Twenty PD patients and 20 healthy controls (HCs) underwent a classical facial mimicry manipulation (holding a pen with the lips, teeth, or nondominant hand) while performing an emotional change detection task with faces. RESULTS: As expected, emotion recognition was significantly influenced by facial mimicry manipulation in HCs, further supporting the hypothesis of facial feedback and the related theory of embodied simulation. Importantly, patients with PD, generally and independent from the facial mimicry manipulation, were impaired in their ability to detected emotion changes. Our data further show that PD patients' facial emotional recognition abilities are completely unaffected by mimicry manipulation, suggesting that PD patients cannot profit from an artificial modulation of the already impaired facial feedback. CONCLUSIONS: These findings suggest that it is not the hypomimia and the absence of facial feedback per se, but a disruption of the facial feedback loop, that leads to the prominent emotion recognition deficit in PD patients.
Date 2023 Mar
Language eng
License © 2022 The Authors. European Journal of Neurology published by John Wiley & Sons Ltd on behalf of European Academy of Neurology.
Extra Place: England
Volume 30
Pages 622-630
Publication European journal of neurology
DOI 10.1111/ene.15647
Issue 3
Journal Abbr Eur J Neurol
ISSN 1468-1331 1351-5101
PMID 36435983
Date Added 6.7.2025, 19:09:37
Modified 5.9.2025, 14:42:30

Notes:

Included

sample characteristics

size: 20 PD and 20 HC

Parkinson's Disease type and duration: idiopathic PD, Mduration = 13.3 SD= 16.83

Medication: on medication

Hoehn-Yahr: NA

UPDRS-3: NA

Gender (male): 10 males (50%)

averaged ages (SD, range): M= 70.85 SD= 6.7

other neurological disease (tumor, stroke, etc.): None

other major psychopathology: None

origin country (or ethnicity): Germany

method observational

instruments used in order to quantify the variables

Social cognition aspect: emotion recognition

Name of the task:

type of stimulus [face/voice etc., Ekman faces/other etc.]: colored visual stimuli consisted of 24 different characters (12 female, 12 male) from the Karolinska face database. the hair region was cut off and the background of all images was gray- scaled. For each character, six different emotional change sequences were created.

task condition: neutral, happy, sad: morphs from neutral to happy, happy to neutral, neutral to sad, and sad to neutral facial expressions for quantitative changes and changes from happy to sad and sad to happy facial expressions for the qualitative changes. facial mimicry manipulation (holding a pen with the lips, teeth, or nondominant hand).

operationalization:

The emotional change detection task - The playback of these sequences was self- paced; by pressing the space bar, participants navigated forward through the morph sequences. Each morph sequence comprised 40 frames, and with every button press, the initial emotion changed stepwise into another one. As soon as a change of the initial emotional expression was detected, participants pressed the enter button. >> frame number of the detection of change

Main findings related to the review's scope

Parkinson disease (PD) patients need significantly more frames to detect emotional change.

Tags: Emotion recognition, behavioral

Identification and intensity of disgust: Distinguishing visual, linguistic and facial expressions processing in Parkinson disease.

**Item Type**	Journal Article
**Author**	Anna Sedda
**Author**	Sara Petito
**Author**	Maria Guarino
**Author**	Andrea Stracciari
**Abstract**	OBJECTIVES & METHODS: Most of the studies since now show an impairment for facial displays of disgust recognition in Parkinson disease. A general impairment in disgust processing in patients with Parkinson disease might adversely affect their social interactions, given the relevance of this emotion for human relations. However, despite the importance of faces, disgust is also expressed through other format of visual stimuli such as sentences and visual images. The aim of our study was to explore disgust processing in a sample of patients affected by Parkinson disease, by means of various tests tackling not only facial recognition but also other format of visual stimuli through which disgust can be recognized. RESULTS: Our results confirm that patients are impaired in recognizing facial displays of disgust. Further analyses show that patients are also impaired and slower for other facial expressions, with the only exception of happiness. Notably however, patients with Parkinson disease processed visual images and sentences as controls. CONCLUSIONS: Our findings show a dissociation within different formats of visual stimuli of disgust, suggesting that Parkinson disease is not characterized by a general compromising of disgust processing, as often suggested. The involvement of the basal ganglia-frontal cortex system might spare some cognitive components of emotional processing, related to memory and culture, at least for disgust.
**Date**	2017 Jul 14
**Language**	eng
**License**	Copyright © 2017 Elsevier B.V. All rights reserved.
**Extra**	Place: Netherlands
**Volume**	330
**Pages**	30-36
**Publication**	Behavioural brain research
**DOI**	10.1016/j.bbr.2017.05.003
**Journal Abbr**	Behav Brain Res
**ISSN**	1872-7549 0166-4328
**PMID**	28476571
**Date Added**	6.7.2025, 19:09:38
**Modified**	5.9.2025, 14:56:25

Notes:

Included

Sample characteristics

Size: 19 PD, 20 HC

PD-type: Idiopathic PD

PD-duration: M=9 (SD=6.7)

Medication: Na

Hoehn-Yahr: 9 patients less then 2; 10 patients with 3 or 4

UPDRS-3: NA

Gender (male): 10 males (53%)

Age: M=71.8 (SD=7.8)

Other neurological disease (tumor, stroke, etc.): None

Other major psychopathology: None

Origin country (or ethnicity): Italy

method (Review, meta-analysis or observational and/or self-reported):

instruments used in order to quantify the variables

Social cognition aspect: Facial emotion recognition

Name of the task: Facial emotion recognition task (FER task)

Type of stimulus [face/voice etc., Ekman faces/other etc.]: Ekman faces. 12 displays for each of the 5 emotions, portrayed by 4 individuals (2 males and 2 females)

Task condition: fear, sadness, disgust, happiness and anger

Operationalization: Correct answers

Main findings related to the review's scope

FER analysis

main effect of Group and a significant interaction between Emotion x Group.

On average, controls recognized emotions with greater accuracy than patients did.

controls outperformed patients in anger, fear, sadness, and disgust recognition, but not happiness.

Tags: Emotion recognition, behavioral

Imaging changes associated with cognitive abnormalities in Parkinson's disease

**Item Type**	Journal Article
**Author**	Yuko Koshimori
**Author**	Barbara Segura
**Author**	Leigh Christopher
**Author**	Nancy Lobaugh
**Author**	Sarah Duff-Canning
**Author**	Romina Mizrahi
**Author**	Clement Hamani
**Author**	Anthony E. Lang
**Author**	Kelly Aminian
**Author**	Sylvain Houle
**Author**	Antonio P. Strafella
**Abstract**	The current study investigates both gray and white matter changes in non-demented Parkinson's disease (PD) patients with varying degrees of mild cognitive deficits and elucidates the relationships between the structural changes and clinical sequelae of PD. Twenty-six PD patients and 15 healthy controls (HCs) were enrolled in the study. Participants underwent T1-weighted and diffusion tensor imaging (DTI) scans. Their cognition was assessed using a neuropsychological battery. Compared with HCs, PD patients showed significant cortical thinning in sensorimotor (left pre- and postcentral gyri) and cognitive (left dorsolateral superior frontal gyrus [DLSFG]) regions. The DLSFG cortical thinning correlated with executive and global cognitive impairment in PD patients. PD patients showed white matter abnormalities as well, primarily in bilateral frontal and temporal regions, which also correlated with executive and global cognitive impairment. These results seem to suggest that both gray and white matter changes in the frontal regions may constitute an early pathological substrate of cognitive impairment of PD providing a sensitive biomarker for brain changes in PD.
**Date**	2015-07
**Language**	English
**Extra**	Place: TIERGARTENSTRASSE 17, D-69121 HEIDELBERG, GERMANY Type: Article
**Volume**	220
**Publisher**	SPRINGER HEIDELBERG
**Pages**	2249-2261
**Publication**	BRAIN STRUCTURE & FUNCTION
**DOI**	10.1007/s00429-014-0785-x
**Issue**	4
**ISSN**	1863-2653
**Date Added**	14.7.2025, 14:50:37
**Modified**	5.9.2025, 14:42:19

Notes:

Not Included: Not assessing SC

Tags: EXCLUDED

Impact of dopamine and cognitive impairment on neural reactivity to facial emotion in Parkinson's disease.

**Item Type**	Journal Article
**Author**	Rotem Dan
**Author**	Filip Růžička
**Author**	Ondrej Bezdicek
**Author**	Jan Roth
**Author**	Evžen Růžička
**Author**	Josef Vymazal
**Author**	Gadi Goelman
**Author**	Robert Jech
**Abstract**	Emotional and cognitive impairments in Parkinson's disease (PD) are prevalent, hamper interpersonal relations and reduce quality of life. It is however unclear to what extent these domains interplay in PD-related deficits and how they are influenced by dopaminergic availability. This study examined the effect of cognitive impairment and dopaminergic medication on neural and behavioral mechanisms of facial emotion recognition in PD patients. PD patients on and off dopaminergic medication and matched healthy controls underwent an emotional face matching task during functional MRI. In addition, a comprehensive neuropsychological evaluation of cognitive function was conducted. Increased BOLD response to emotional faces was found in the visual cortex of PD patients relative to controls irrespective of cognitive function and medication status. Administration of dopaminergic medication in PD patients resulted in restored behavioral accuracy for emotional faces relative to controls and decreased retrosplenial cortex BOLD response to emotion relative to off-medication state. Furthermore, cognitive impairment in PD patients was associated with reduced behavioral accuracy for non-emotional stimuli and predicted BOLD response to emotion in the anterior and posterior cingulate cortices, depending on medication status. Findings of aberrant visual and retrosplenial BOLD response to emotion are suggested to stem from altered attentional and/or emotion-driven modulation from subcortical and higher cortical regions. Our results indicate neural disruptions and behavioral deficits in emotion processing in PD patients that are dependent on dopaminergic availability and independent of cognitive function. Our findings highlight the importance of dopaminergic treatment not only for the motor symptoms but also the emotional disturbances in PD.
**Date**	2019 Nov
**Language**	eng
**License**	Copyright © 2019 Elsevier B.V. and ECNP. All rights reserved.
**Extra**	Place: Netherlands
**Volume**	29
**Pages**	1258-1272
**Publication**	European neuropsychopharmacology : the journal of the European College of Neuropsychopharmacology
**DOI**	10.1016/j.euroneuro.2019.09.003
**Issue**	11
**Journal Abbr**	Eur Neuropsychopharmacol
**ISSN**	1873-7862 0924-977X
**PMID**	31607424
**Date Added**	6.7.2025, 19:09:37

Modified 5.9.2025, 14:32:20

Notes:

Included

sample characteristics

size: 25 PD and 32 HC (matched for age, gender and education.)

Parkinson's Disease type and duration: idiopathic PD Mduration = 11.9 SD=4.7 (4–26)

Medication: on and off medication

Hoehn-Yahr: PD-on M=2 SD= 0.5 (1–3), PD-off NA

UPDRS-3: PD-on M= 15.2 SD= 7.5 (4–31), PD-off M=30.4 SD= 11.1 (8–64)

Gender (male): 15 males (60%)

averaged ages (SD, range): M= 64.7 SD= 8.3

other neurological disease (tumor, stroke, etc.): None

other major psychopathology: None

origin country (or ethnicity): Czech Republic

method observational and imaging

instruments used in order to quantify the variables

Social cognition aspect: Emotion recognition

Name of the task: the emotional face matching task

type of stimulus [face/voice etc., Ekman faces/other etc.]: Facial stimuli were derived from Ekman's Pictures of Facial Affect (POFA).

a target face was presented at the top of the screen and subjects were instructed to select one of two faces presented at the bottom which showed the same emotional expression as the target face (fearful or angry).

task condition: anger and fear

operationalization: accuracy and reaction time

fMRI: 3T MR scanner (Magnetom Skyra, Siemens, Germany). Wakefulness was monitored during the whole scan using an MRI compatible camera. Functional images were acquired using T_2*-weighted gradient-echo echo-planar imaging (GE-EPI) sequence with TR=2 s, TE=30 ms, image matrix=64 × 64, field of view=192 × 192 mm, flip angle=90°, resolution=3 × 3 × 3 mm, interslice gap=0.45 mm. Anatomical images were acquired using a sagittal T1-weighted MP-RAGE sequence with TR=2.2 s, TE=2.43 ms, resolution=1 × 1 × 1 mm; and a T2-weighted 2D sequence with TR=3.2 s, TE=9 ms, resolution=0.9 × 0.9 × 3 mm. T1-weighted images were acquired for coregistration and normalization of the functional images and T2-weighted images were acquired for diagnostic purpose to rule out significant atrophy or any other pathological brain changes.

BOLD response

<u>Main findings related to the review's scope</u>

PD-off had the worse perfomance in accuracy, in comparison to PD-on or HC. No significant difference between PD-on and HC.

No significant differences in RT between groups.

blood oxygenation level dependent (BOLD) contrast signals.

PD patients OFF medication showed increased BOLD response to negative emotional faces compared to healthy controls in the bilateral calcarine sulci and lingual gyri. greater extent in the right hemisphere.

PD patients ON medication showed increased BOLD response to negative emotional faces compared to healthy controls in the bilateral calcarine sulci and lingual gyri. greater extent in the right hemisphere.

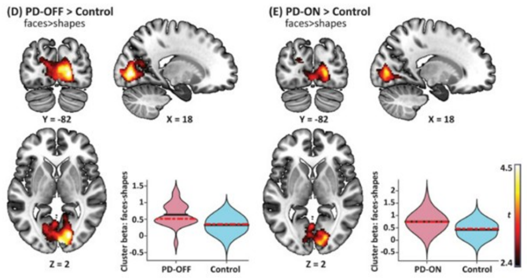

PD patients OFF medication showed increased BOLD response to negative emotional faces compared to PD patients ON medication in the bilateral posterior cingulate/retrosplenial cortex

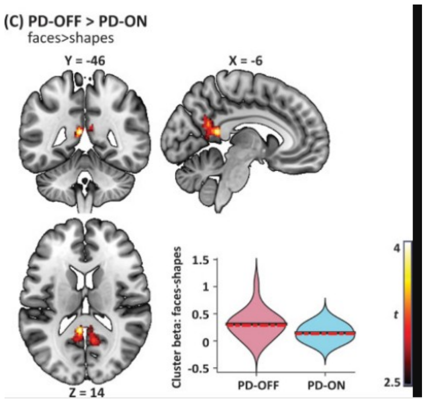

Tags: Emotion recognition, Imaging, behavioral

Impact of gender and genetics on emotion processing in Parkinson's disease - A multimodal study.

**Item Type**	Journal Article
**Author**	Julia Heller
**Author**	Shahram Mirzazade
**Author**	Sandro Romanzetti
**Author**	Ute Habel
**Author**	Birgit Derntl
**Author**	Nils M. Freitag
**Author**	Jörg B. Schulz
**Author**	Imis Dogan
**Author**	Kathrin Reetz
**Abstract**	•Understanding of the phenotypic heterogeneity of Parkinson's disease is needed.•Gender and genetics determine manifestation and progression of Parkinson's disease.•Altered emotion processing in Parkinson's disease is specific to male patients.•This is influenced by endocrinal and genetic factors in both genders.•This finding may impact the diagnosis and treatment of emerging clinical features.
**Date**	2018
**Language**	eng
**Extra**	Place: Netherlands
**Volume**	18

Pages 305-314
Publication NeuroImage. Clinical
DOI 10.1016/j.nicl.2018.01.034
Journal Abbr Neuroimage Clin
ISSN 2213-1582
PMID 29876251
PMCID PMC5987844
Date Added 6.7.2025, 19:09:36
Modified 5.9.2025, 14:38:56

Notes:

Included

sample characteristics

size: 51 PD and 44 HC (matched on sex and age)

Parkinson's Disease type and duration: NA. Mduration = 6.86 SD=5.69. divided by sex.

Medication: Mixed

Hoehn-Yahr: M=1.75 (1-3)

UPDRS-3: M=23.47 SD=10.45

Gender (male): 26 males (51%)

averaged ages (SD, range): M= 64.0 SD= 9.2

other neurological disease (tumor, stroke, etc.): None

other major psychopathology: None

origin country (or ethnicity): Germany

method observational

instruments used in order to quantify the variables

Social cognition aspect: emotion recognition

Name of the task: the computer-based Ekman 60 faces test

type of stimulus [face/voice etc., Ekman faces/other etc.]: Ekman faces

task condition: happiness, disgust, anger, fear, sadness and surprise

operationalization: accuracy

Main findings related to the review's scope

females outperforming males in total emotion recognition performance

this gender effect also emerged for the recognition of disgust

Anger - Post-hoc t-tests showed no significant differences in females (PD versus HC) and between female and male HC, while male PD patients performed significantly worse than female PD patients and male HC

Tags: Emotion recognition, behavioral

Impact of motor symptom asymmetry on non-motor outcomes in Parkinson's disease: a systematic review

**Item Type**	Journal Article
**Author**	P. Voruz
**Author**	D. Guerin
**Author**	J. A. Peron
**Date**	2025-07-01
**Volume**	11
**Publication**	NPJ PARKINSONS DISEASE
**DOI**	10.1038/s41531-025-01046-4
**Issue**	1
**Date Added**	14.7.2025, 14:48:38
**Modified**	14.7.2025, 15:07:56

Notes:

Included: Systematic Review (includes emotion recognition

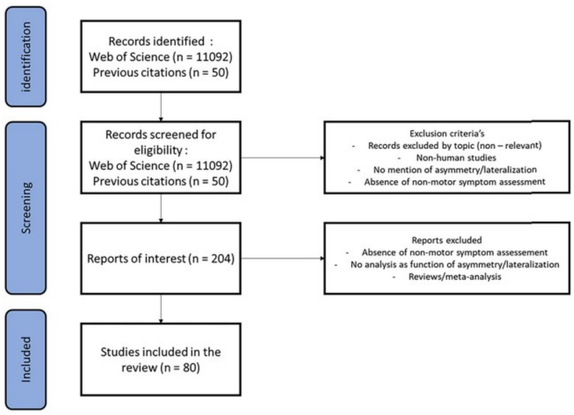

Impact of motor asymmetry on emotional abilities in PD

In terms of emotion abilities, the majority of studies have focused on emotion recognition abilities, in particular on the ability to recognize facial or vocal emotions. These studies highlighted reduced performance for emotion recognition abilities in LPD, regardless of the valence of the emotions, with deficits for negative emotions (disgust; anger; sadness), positive emotions (happiness) but also for the recognition of neutral stimuli. Some studies showed no effects and others showed reduced performances at the same level in both groups as compared to healthy controls, while only one study showed reduced abilities in RPD, but on measures of emotional valence of artworks, not on stimuli with human characteristics

Effects of therapies on emotional processing as a function of motor symptom asymmetry

Regarding emotion recognition abilities, only one study assessed changes following STN-DBS, finding that patients with LPD showed normalization in emotional prosody recognition three months postoperatively. This normalization was evident in ratings for neutral, happy, and angry stimuli. In contrast, RPD patients exhibited a postoperative decline in recognizing emotional prosody, despite comparable preoperative performance to the control group. Notably, significant differences were observed postoperatively for the Fear recognition

Limitations

The systematic literature review highlights five main groups of limitations across the included studies: (i) heterogeneity in experimental designs and assessment tools, (ii) variability in patient sociodemographic and clinical characteristics, (iii) inconsistency in methods used to calculate motor symptom asymmetry, (iv) insufficient control of confounding variables such as gender, medication

status, and motor symptom subtypes, and (v) lack of standardization in the reporting and interpretation of concomitant treatments. These limitations often overlap and compound one another, making it difficult to compare results across studies and draw robust, generalizable conclusions regarding non-motor symptom expression in PD. Addressing these issues will require more harmonized research protocols and more systematic consideration of clinically relevant variables.

Tags: emotion recognition, behavioral

Impaired decision-making in Parkinson's disease

**Item Type**	Journal Article
**Author**	M Mimura
**Author**	R Oeda
**Author**	M Kawamura
**Abstract**	The present study demonstrated that individuals with Parkinson's disease display impairments in making profitable decisions, as evidenced by poorer performance on the Iowa Gambling Task relative to matched controls. The ability to make beneficial judgments solely correlates with ability to appropriately attribute internal mental-state to other people, as shown in a mind-reading task. However, decision-making ability for individuals with Parkinson's disease is not associated with effective execution, intellectual function or depressed mood. These findings offer a new perspective on the cognitive impairments that affect social living and adaptive decisions for individuals with Parkinson's disease. (c) 2006 Elsevier Ltd. All rights reserved.
**Date**	2006
**Language**	English
**Extra**	Place: THE BOULEVARD, LANGFORD LANE, KIDLINGTON, OXFORD OX5 1GB, OXON, ENGLAND Type: Article
**Volume**	12
**Publisher**	ELSEVIER SCI LTD
**Pages**	169-175
**Publication**	PARKINSONISM & RELATED DISORDERS
**DOI**	10.1016/j.parkreldis.2005.12.003
**Issue**	3
**ISSN**	1353-8020
**Date Added**	14.7.2025, 14:50:43
**Modified**	5.9.2025, 14:46:57

Notes:

Included

Sample characteristics

Size: 18 PD, 40 HC (matched for age, sex, intellectual level)

PD-type: Idiopathic PD

PD-duration: NA

Medication: All patients took l-dopa

Hoehn-Yahr: 2 or 3

UPDRS-3: NA

Gender (male): 5 (38%)

Age: M = 68.9, SD = 7.0, Range = 49-76

Other neurological disease (tumor, stroke, etc.): None

Other major psychopathology: None

Origin country (or ethnicity): Japan

method (Review, meta-analysis or observational and/or self-reported):

instruments used in order to quantify the variables

Social cognition aspect: ToM

Name of the task: Reading the Mind in the Eyes Test (RMET)

Type of stimulus [face/voice etc., Ekman faces/other etc.]: 25 black-and-white photographs (8.5 cm! 17 cm) presenting the eye region of 25 faces of adult Japanese people (13 women, 12 men)

Task condition: the subject was presented with a photo and a choice of antonyms indicating mental-states, and asked to choose the word best describing what the person in the picture was thinking or feeling.

Operationalization: Correct answers, Max. score = 25

Main findings related to the review's scope

RMET

1. 1. For the mental-state condition, the PD group successfully identified a smaller number of eye expressions than the HC2 group.
   2. In contrast, the PD group performed at the same level as the HC2 group on the gender condition.

Tags: ToM, behavioral

Impaired emotion recognition in music in Parkinson’s disease

Item Type Journal Article
Author Mirjam J. van Tricht
Author Harriet M. M. Smeding
Author Johannes D. Speelman
Author Ben A. Schmand
Abstract Music has the potential to evoke strong emotions and plays a significant role in the lives of many people. Music might therefore be an ideal medium to assess emotion recognition. We investigated emotion recognition in music in 20 patients with idiopathic Parkinson's disease (PD) and 20 matched healthy volunteers. The role of cognitive dysfunction and other disease characteristics in emotion recognition was also evaluated. We used 32 musical excerpts that expressed happiness, sadness, fear or anger. PD patients were impaired in recognizing fear and anger in music. Fear recognition was associated with executive functions in PD patients and in healthy controls, but the emotion recognition impairments of PD patients persisted after adjusting for executive functioning. We found no differences in the recognition of happy or sad music. Emotion recognition was not related to depressive symptoms, disease duration or severity of motor symptoms. We conclude that PD patients are impaired in recognizing complex emotions in music. Although this impairment is related to executive dysfunction, our findings most likely reflect an additional primary deficit in emotional processing.
Date 2010
URL https://www.sciencedirect.com/science/article/pii/S027826261000076X
Volume 74
Pages 58-65
Publication Brain and Cognition
DOI https://doi.org/10.1016/j.bandc.2010.06.005
Issue 1
ISSN 0278-2626
Date Added 6.7.2025, 19:12:36
Modified 5.9.2025, 14:58:41

Notes:

Not Included: not on SC

Tags: EXCLUDED

Impaired emotion recognition in music in Parkinson's disease.

Item Type Journal Article
Author Mirjam J. van Tricht
Author Harriet M. M. Smeding
Author Johannes D. Speelman
Author Ben A. Schmand
Abstract Music has the potential to evoke strong emotions and plays a significant role in the lives of many people. Music might therefore be an ideal medium to assess emotion recognition. We investigated emotion recognition in music in 20 patients with idiopathic Parkinson's disease (PD) and 20 matched healthy volunteers. The role of

cognitive dysfunction and other disease characteristics in emotion recognition was also evaluated. We used 32 musical excerpts that expressed happiness, sadness, fear or anger. PD patients were impaired in recognizing fear and anger in music. Fear recognition was associated with executive functions in PD patients and in healthy controls, but the emotion recognition impairments of PD patients persisted after adjusting for executive functioning. We found no differences in the recognition of happy or sad music. Emotion recognition was not related to depressive symptoms, disease duration or severity of motor symptoms. We conclude that PD patients are impaired in recognizing complex emotions in music. Although this impairment is related to executive dysfunction, our findings most likely reflect an additional primary deficit in emotional processing.

Date 2010 Oct
Language eng
License 2010 Elsevier Inc. All rights reserved.
Extra Place: United States
Volume 74
Pages 58-65
Publication Brain and cognition
DOI 10.1016/j.bandc.2010.06.005
Issue 1
Journal Abbr Brain Cogn
ISSN 1090-2147 0278-2626
PMID 20633975
Date Added 6.7.2025, 19:09:37
Modified 5.9.2025, 14:59:31

Notes:

Not Included: not on SC

Impaired Emotional Mirroring in Parkinson's Disease-A Study on Brain Activation during Processing of Facial Expressions

Item Type Journal Article
Author Anna Pohl
Author Silke Anders
Author Hong Chen
Author Harshal Jayeshkumar Patel
Author Julia Heller
Author Kathrin Reetz
Author Klaus Mathiak
Author Ferdinand Binkofski
Abstract Background: Affective dysfunctions are common in patients with Parkinson's disease, but the underlying neurobiological deviations have rarely been examined. Parkinson's disease is characterized by a loss of dopamine neurons in the substantia nigra resulting in impairment of motor and non-motor basal ganglia-cortical loops.

Concerning emotional deficits, some studies provide evidence for altered brain processing in limbic-and lateral-orbitofrontal gating loops. In a second line of evidence, human premotor and inferior parietal homologs of mirror neuron areas were involved in processing and understanding of emotional facial expressions. We examined deviations in brain activation during processing of facial expressions in patients and related these to emotion recognition accuracy. Methods: 13 patients and 13 healthy controls underwent an emotion recognition task and a functional magnetic resonance imaging (fMRI) measurement. In the Emotion Hexagon test, participants were presented with blends of two emotions and had to indicate which emotion best described the presented picture. Blended pictures with three levels of difficulty were included. During fMRI scanning, participants observed video clips depicting emotional, non-emotional, and neutral facial expressions or were asked to produce these facial expressions themselves. Results: Patients performed slightly worse in the emotion recognition task, but only when judging the most ambiguous facial expressions. Both groups activated inferior frontal and anterior inferior parietal homologs of mirror neuron areas during observation and execution of the emotional facial expressions. During observation, responses in the pars opercularis of the right inferior frontal gyrus, in the bilateral inferior parietal lobule and in the bilateral supplementary motor cortex were decreased in patients. Furthermore, in patients, activation of the right anterior inferior parietal lobule was positively related to accuracy in the emotion recognition task. Conclusion: Our data provide evidence for a contribution of human homologs of monkey mirror areas to the emotion recognition deficit in Parkinson's disease.

Date 2017-12-18
Language English
Extra Place: AVENUE DU TRIBUNAL FEDERAL 34, LAUSANNE, CH-1015, SWITZERLAND Type: Article
Volume 8
Publisher FRONTIERS MEDIA SA
Publication FRONTIERS IN NEUROLOGY
DOI 10.3389/fneur.2017.00682
ISSN 1664-2295
Date Added 14.7.2025, 14:50:35
Modified 5.9.2025, 14:51:29

Notes:

Not Included: not on SC

Tags: EXCLUDED

Impaired empathy: A non-motor symptom associated with advanced stages of Parkinson's disease

Item Type Journal Article
Author Nele Schmidt
Author Laura Paschen
Author Günther Deuschl

Author Karsten Witt
Date 2017
URL https://www.sciencedirect.com/science/article/pii/S2210533617300205
Volume 8
Pages 4
Publication Basal Ganglia
DOI https://doi.org/10.1016/j.baga.2017.02.012
ISSN 2210-5336
Date Added 6.7.2025, 19:12:34
Modified 6.7.2025, 19:12:34

Notes:

Not Included: a poster

Tags: EXCLUDED

Impaired facial emotion recognition in relation to social behaviours in de novo Parkinson's disease.

Item Type Journal Article
Author Anne Carien Slomp
Author Sygrid van der Zee
Author Jeffrey M. Boertien
Author Marleen J. J. Gerritsen
Author Teus van Laar
Author Jacoba M. Spikman
Abstract Facial emotion recognition (FER) is a crucial component of social cognition and is essential in social-interpersonal behaviour regulation. Although FER impairment is well-established in advanced PD, data about FER at the time of diagnosis and its relationship with social behavioural problems in daily life are lacking. The aim was to examine FER at the time of PD diagnosis compared to a matched healthy control (HC) group and to associate FER with indices of social behavioural problems. In total, 142 de novo, treatment-naïve PD patients and 142 HC were included. FER was assessed by the Ekman 60 faces test (EFT). Behavioural problems in PD patients were assessed using the Dysexecutive Questionnaire (DEX-self and DEX-proxy) and the Apathy Evaluation Scale (AES-self). PD patients had significantly lower EFT-total scores (p = .001) compared to HC, with worse recognition of Disgust (p = .001) and Sadness (p = .016). Correlational analyses yielded significant correlations between AES-self and both EFT-total (r(s) = .28) and Fear (r(s) = .22). Significant negative correlations were found between DEX-proxy and both EFT-total (r(s) = -.28) and Anger (r(s) = -.26). Analyses of DEX-subscales showed that proxy ratings were significantly higher than patient-ratings for the Social Conventions subscale (p = .047). This DEX-proxy subscale had the strongest correlation with EFT-total (r(s) = -.29). Results show that de novo PD patients already show impaired FER compared to HC. In addition, lower FER is linked to self-reported apathy and proxy-reported social-behavioural problems, especially concerning social conventions. These findings validate the importance of the inclusion of social cognition measures in the

	neuropsychological assessment even in early PD.
**Date**	2024 Jun
**Language**	eng
**License**	© 2023 The Authors. Journal of Neuropsychology published by John Wiley & Sons Ltd on behalf of The British Psychological Society.
**Extra**	Place: England
**Volume**	18
**Pages**	205-216
**Publication**	Journal of neuropsychology
**DOI**	10.1111/jnp.12341
**Issue**	2
**Journal Abbr**	J Neuropsychol
**ISSN**	1748-6653 1748-6645
**PMID**	37488778
**Date Added**	6.7.2025, 19:09:36
**Modified**	5.9.2025, 14:57:10

Notes:

Included

Sample characteristics

Size: 142 PD, 142 HC (matched for sex, age, education

PD-type: NA

PD-duration: de novo

Medication: NA

Hoehn-Yahr: NA

UPDRS-3: M = 30.68, SD = 11.47

Gender (male): 101 (71%)

Age: M = 65.03, SD = 9.34

Other neurological disease (tumor, stroke, etc.): None

Other major psychopathology: None

Origin country (or ethnicity): Dutch

method behavioral

instruments used in order to quantify the variables

Social cognition aspect: emotion recognition

Name of the task: Ekman's 60 Faces Test (EFT)

Type of stimulus [face/voice etc., Ekman faces/other etc.]:60 black-and-white pictures of men and woman.

Task condition: anger, disgust, fear, happiness, sadness, surprise

Operationalization: Each picture was shown for 5 s, after which the participant had to determine which emotion was expressed. Labels of the six emotions were shown at the bottom of the computer screen at all time >> accuracy

EFT = total score

Main findings related to the review's scope

PD patients had significantly lower EFT-total scores than HC

PD patients had significantly lower scores on the emotions Disgust and Sadness compared to HC. no other sig dif.

Tags: Emotion Recognition, behavioral

Impaired neural processing of dynamic faces in left-onset Parkinson's disease

Item Type Journal Article
Author Patricia Garrido-Vasquez
Author Marc D. Pell
Author Silke Paulmann
Author Bernhard Sehm
Author Sonja A. Kotz
Abstract Parkinson's disease (PD) affects patients beyond the motor domain. According to previous evidence, one mechanism that may be impaired in the disease is face processing. However, few studies have investigated this process at the neural level in PD. Moreover, research using dynamic facial displays rather than static pictures is scarce, but highly warranted due to the higher ecological validity of dynamic stimuli. In the present study we aimed to investigate how PD patients process emotional and non emotional dynamic face stimuli at the neural level using event-related potentials. Since the literature has revealed a predominantly right-lateralized network for dynamic face processing, we divided the group into patients with left (LPD) and right (RPD) motor symptom onset (right versus left cerebral hemisphere predominantly affected, respectively). Participants watched short video clips of happy, angry, and neutral expressions and engaged in a shallow gender decision task in order to avoid confounds of task difficulty in the data. In line with our expectations, the LPD group showed significant face processing deficits compared to controls. While there were no group differences in early, sensory-driven processing (fronto-central N1 and posterior P1), the vertex positive potential, which is considered the fronto-central counterpart of the face-specific posterior N170 component, had a reduced amplitude and delayed latency in the LPD group. This may indicate disturbances of structural face processing in LPD. Furthermore, the effect was independent of the emotional content of the videos. In contrast, static facial identity recognition performance in LPD was not significantly different from controls, and comprehensive testing of cognitive functions did not reveal any deficits in this group. We therefore conclude that PD, and more specifically the predominant right-hemispheric affection in left-onset PD, is associated with impaired processing of dynamic facial expressions, which could be one of the mechanisms behind the often reported problems of PD patients in their social lives. (C) 2016 Elsevier Ltd. All rights reserved.
Date 2016-02
Language English
Extra Place: THE BOULEVARD, LANGFORD LANE, KIDLINGTON, OXFORD OX5 1GB, ENGLAND Type: Article
Volume 82
Publisher PERGAMON-ELSEVIER SCIENCE LTD
Pages 123-133
Publication NEUROPSYCHOLOGIA
DOI 10.1016/j.neuropsychologia.2016.01.017
ISSN 0028-3932
Date Added 14.7.2025, 14:50:36
Modified 5.9.2025, 14:36:22

Notes:

Not Included: EEG study
Tags: EXCLUDED

Impaired perception of vocal emotions in Parkinson's disease: influence of speech time processing and executive functioning.

**Item Type**	Journal Article
**Author**	C. Breitenstein
**Author**	D. Van Lancker
**Author**	I. Daum
**Author**	C. H. Waters
**Abstract**	Little is known about the underlying dimensions of impaired recognition of emotional prosody that is frequently observed in patients with Parkinson's disease (PD). Because patients with PD also suffer from working memory deficits and impaired time perception, the present study examined the contribution of (a) working memory (frontal executive functioning) and (b) processing of the acoustic parameter speech rate to the perception of emotional prosody in PD. Two acoustic parameters known to be important for emotional classifications (speech duration and pitch variability) were systematically varied in prosodic utterances. Twenty patients with PD and 16 healthy controls (matched for age, sex, and IQ) participated in the study. The findings imply that (1) working memory dysfunctions and perception of emotional prosody are not independent in PD, (2) PD and healthy control subjects perceived vocal emotions categorically along two acoustic manipulation continua, and (3) patients with PD show impairments in processing of speech rate information.
**Date**	2001 Mar
**Language**	eng
**Extra**	Place: United States
**Volume**	45
**Pages**	277-314
**Publication**	Brain and cognition
**DOI**	10.1006/brcg.2000.1246
**Issue**	2
**Journal Abbr**	Brain Cogn
**ISSN**	0278-2626
**PMID**	11237372
**Date Added**	6.7.2025, 19:09:39
**Modified**	5.9.2025, 14:29:35

Notes:

Included

sample characteristics

size: 20 PD and 16 HC (matched in age and education)

Parkinson's Disease type and duration: idiopathic PD, EARLY-PD M= 1.35 SD= 0.65 MODERATE-PD M= 4.93 SD= 4.86

Medication: 14 on medication (MODERATE-PD), 6 placebo-controlled (EARLY-PD)

Hoehn-Yahr: M=NA

UPDRS-3: MODERATE-PD M= 27.5 SD=13.5 4–56 EARLY-PD M= 17.5 SD=5.1 11.5–26

Gender (male): MODERATE-PD 9 males (64%) EARLY-PD 4 males (67%)

averaged ages (SD, range): MODERATE-PD M=72.6 SD=6 6.9 EARLY-PD M=68.3 SD=5.1

other neurological disease (tumor, stroke, etc.): None

other major psychopathology: NA

origin country (or ethnicity): Germany

method observational

instruments used in order to quantify the variables

Social cognition aspect: emotion recognition

Name of the task: the Florida Affect Battery

type of stimulus [face/voice etc., Ekman faces/other etc.]: ‘‘Conflicting emotional prosody’’ (prosody in the English language). 32 emotional prosodic stimuli, spoken by a female voice. Half of the sentences convey the same emotional meaning in both semantic content and emotional prosody (e.g., ‘‘The couple beamed at their brand new grandson,’’ spoken in a happy tone of voice). These are the so-called congruent stimuli. On the remaining 16 trials, the emotional prosody is inconsistent with the emotional content of the sentence. These incongruent trials are further divided according to the degree of discrepant information expressed by the prosodic and semantic message. The first type is referred to as ‘‘conflicting’’ because in these 8 stimuli, the emotional prosody is directly in conflict with the emotional semantic meaning of the sentence (e.g., ‘‘The couple beamed at their brand new grandson’’ said in an angry tone of voice). The second type of incongruent stimuli comprised 8 stimuli in which a neutral semantic meaning was paired with an emotional prosodic message or vice versa (e.g., ‘‘The chairs are made of wood,’’ spoken in a sad tone of voice, or ‘‘The man held his dying son,’’ spoken in a neutral tone of voice). These were the so-called ‘‘conflicting-neutral’’ stimuli.

operationalization: Subjects were given the instruction to listen to the emotional tone of voice while disregarding what the speaker said. A total prosody score is calculated as well as separate scores for the congruent and incongruent (conflicting and conflicting-neutral) conditions.

Name of the task: NA

type of stimulus [face/voice etc., Ekman faces/other etc.]: Systematic variation of duration and pitch cues (prosody tape in the German language). from a standardized set of emotional prosodic utterances

(Breitenstein, Daum, Ackermann, Luetgehetmann, & Mueller, 1996) were selected. The sentences were all in German (to minimize distraction effects by semantic meaning of the sentences) and neutral in propositional content, but differed in their meaning (four different meanings) and their emotional tone of voice

Conditions: happy, sad, angry, frightened, and neutral

operationalization: Besides judging which of five possible emotions they had perceived, subjects were also asked to rate each item on a 5-point scale: (a) ‘‘How certain are you about the choice of that emotion?’’ and (b) ‘‘How active or passive did the utterance sound?’’

Main findings related to the review's scope

“Conflicting Emotional Prosody” (Prosody in the English Language):

The data provide evidence that the MODERATE-PD group based responses more often on sentence content, especially when the prosodic and the semantic meanings of the stimulus differed only slightly such that the contrast between the prosodic and semantic channels of meaning was less stark (e.g., ‘‘They slit all the tires on my car,’’ spoken with a neutral tone of voice). This occurred despite the instruction to disregard the semantic channel.

On specific emotions: pronounced impairment of the MODERATE-PD group compared to the HC and the EARLY-PD groups in classifying happy intonations. For all groups, correct identification rates were lowest for angry and happy and highest for sad utter ances.

Systematic Variation of Duration and Pitch Cues (Prosody Tape in the German Language):

MODERATE-PD and EARLY-PD patients did not significantly differ in their performance in any of the below reported analyses and our previous experience demonstrated that large sample sizes are required to achieve smooth trends across manipulation factors, data were pooled across both PD groups.

Differences apeared only in Happy condition: PD patients were benefiting less by the gradual increase in speech duration than the HC subjects.

Tags: Emotion recognition, behavioral

Impaired processing of conspecifics in Parkinson's disease

Item Type Journal Article

Author Luca Piretti
Author Sonia Di Tella
Author Maria Rita Lo Monaco
Author Valentina Delle Donne
Author Raffaella Ida Rumiati
Author Maria Caterina Silveri

Abstract Experimental evidence indicates that the inferior frontal gyrus (IFG) processes emotional/affective features crucial to elaborate knowledge about social groups and that knowledge of social concepts is stored in the anterior temporal lobe (ATL). We investigated whether knowledge about social groups is impaired in Parkinson's disease (PD), in which dysfunctional connectivity between IFG and ATL has been

demonstrated. PD patients (N = 20) and healthy controls (HC, N = 16) were given a lexical decision task in a semantic priming paradigm: the prime-targets included 144 words and 144 pseudowords, each preceded by three types of prime ("animals," "things," "persons"). Out of these 288 prime-targets, forty-eight were congruent (same category) and 96 incongruent (different category). Out of 48 congruent prime-targets, 24 denoted social items and 24 nonsocial items. Thus, four types of trials were obtained: congruent social; congruent nonsocial; incongruent social; incongruent nonsocial. Congruent target-words were recognized better than incongruent target-words by all groups. The semantic priming effect was preserved in PD; however, accuracy was significantly lower in PD than in HC in social items. No difference emerged between the two groups in nonsocial items. Impaired processing of words denoting social groups in PD may be due to impairment in accessing the affective/emotional features that characterize conceptual knowledge of social groups, for the functional disconnection between the IFG and the ATL.

**Date**	2024-09-02
**Language**	English
**Extra**	Place: 2-4 PARK SQUARE, MILTON PARK, ABINGDON OX14 4RN, OXON, ENGLAND Type: Article
**Volume**	31
**Publisher**	ROUTLEDGE JOURNALS, TAYLOR & FRANCIS LTD
**Pages**	787-795
**Publication**	APPLIED NEUROPSYCHOLOGY-ADULT
**DOI**	10.1080/23279095.2022.2074299
**Issue**	5
**ISSN**	2327-9095
**Date Added**	14.7.2025, 14:50:29
**Modified**	5.9.2025, 14:51:14

Notes:

Not Included: not on SC

Tags: EXCLUDED

Impaired Recognition of Facial Emotion in Patients With Parkinson Disease Under Dopamine Therapy.

**Item Type**	Journal Article
**Author**	Rosanna Palmeri
**Author**	Viviana Lo Buono
**Author**	Lilla Bonanno
**Author**	Cettina Allone
**Author**	Nancy Drago
**Author**	Chiara Sorbera
**Author**	Vincenzo Cimino
**Author**	Giuseppe di Lorenzo

Author Alessia Bramanti
Author Silvia Marino

Abstract INTRODUCTION: Parkinson disease (PD) is a neurodegenerative disorder characterized by motor and nonmotor symptoms. The impaired ability to recognize facial emotion expressions represents an important nonmotor symptom. The aim of this study is to investigate the ability in recognizing facial emotion expressions in patients with PD under dopamine replacement therapy. METHODS: Thirty medicated patients with PD and 15 healthy controls (HC) were enrolled. All participants performed the Ekman 60-Faces test for emotional recognition. All patients underwent a neuropsychological evaluation for global cognitive functioning, depression, and anxiety. RESULTS: Patients with PD were impaired in recognizing emotions. Significant differences between PD and HC were found in Ekman 60-Faces test scores (P < .001), and in Ekman 60-Faces test subscales, in particular, sadness, fear, disgust, anger, and surprise (P < .001). CONCLUSIONS: The nigrostriatal dopaminergic depletion seems to determine emotional information processing dysfunction. This relevant nonmotor symptom could have consequences in daily living reducing interactions and social behavioral competence.

Date 2020 Sep
Language eng
Extra Place: United States
Volume 33
Pages 265-271
Publication Journal of geriatric psychiatry and neurology
DOI 10.1177/0891988719882094
Issue 5
Journal Abbr J Geriatr Psychiatry Neurol
ISSN 0891-9887
PMID 31635513
Date Added 6.7.2025, 19:09:36
Modified 5.9.2025, 14:48:58

Notes:

Included

Sample characteristics

Size: 30 PD, 15 HC (sex, age, education matched)

PD-type: Idiopathic

PD-duration: M = 4.63, SD = 2.66

Medication: on state

Hoehn-Yahr: max= 3; M = 2.4 [SD=0.77]

UPDRS-3: NA

Gender (male): 15 (50%)

Age: M = 64.20, Sd = 3.79

Other neurological disease (tumor, stroke, etc.): None

Other major psychopathology: None

Origin country (or ethnicity): NA

method (Review, meta-analysis or observational and/or self-reported):

instruments used in order to quantify the variables

Social cognition aspect: Facial Emotion recognition

Name of the task: The Ekman 60-Faces test

Type of stimulus [face/voice etc., Ekman faces/other etc.]: The test consists of 10 photographs showing people's faces (6 woman) taken from the Ekman and Friesen series of Pictures of Facial Affect.

Task condition: each photograph displayed 1 of 6 basic facial expressions (anger, disgust, fear, sadness, happiness, and surprise).

Operationalization: Patients were asked to select 1 of 6 emotion labels, under the picture, that best describes the facial expression shown. There is not a time for the response and no feedback was given >> accuracy

Main findings related to the review's scope

significant lower scores for PD in comparison to HC in Ekman 60-Faces test total score (P < .001), and in particular, sadness, fear, disgust, anger, and surprise (P < .001), but no significant difference for happiness (P = .94)

Tags: Emotion Recognition, behavioral

Impaired recognition of facial expressions of anger in Parkinson's disease patients acutely withdrawn from dopamine replacement therapy

**Item Type**	Journal Article
**Author**	Andrew D. Lawrence
**Author**	Ines K. Goerendt
**Author**	David J. Brooks
**Abstract**	We have previously reported that acute dopaminergic blockade in healthy volunteers results in a transient disruption of the recognition of facial expressions of anger, whilst leaving intact the recognition of other facial expressions (including fear and disgust) and facial identity processing. Parkinson's disease (PD) is characterised by cell loss in dopaminergic neuronal populations, and hence we predicted that PD would be associated with impaired anger recognition. We reasoned that treatment with dopamine replacement therapy (DRT) could mask any deficit present in PD, and therefore studied facial expression recognition in a group of PD patients transiently withdrawn from DRT. Seventeen PD patients were compared to 21 age- and IQ-matched controls on the Ekman 60 task, which required the forced-choice labelling of 10 exemplars of each of six facial expressions (anger, disgust, fear, sadness, happiness, surprise). In line with our predictions, PD patients showed a selective impairment in the recognition of facial expressions of anger. This deficit was not related to the PD patients' performance on the Benton unfamiliar-face matching task, which was normal, nor was the deficit related to overall disease severity, or to depression symptoms. However, as predicted by simulation theories, impaired anger recognition in PD was related to reduced levels of the anger-linked temperament trait, exploratory excitability. The results extend our previous findings of a role for dopamine in the processing of facial expressions of anger, and demonstrate the power of adopting a phylogenetic, comparative perspective on emotions.
**Date**	2007
**URL**	https://www.sciencedirect.com/science/article/pii/S0028393206001618
**Volume**	45
**Pages**	65-74
**Publication**	Neuropsychologia
**DOI**	https://doi.org/10.1016/j.neuropsychologia.2006.04.016
**Issue**	1
**ISSN**	0028-3932
**Date Added**	6.7.2025, 19:12:35
**Modified**	5.9.2025, 14:43:01

Notes:

Included

sample characteristics

size: 17 PD and 21 HC (matches in age and reading-estimated IQ)

Parkinson's Disease type and duration: NA

Medication: off medication (Patients were asked to abstain from taking their medication the night before the assessment was scheduled to take place)

Hoehn-Yahr: NA

UPDRS-3: M= 22.7 SD=6.0

Gender (male): 10 males (59%)

averaged ages (SD, range): M= 56.5 SD=8.3

other neurological disease (tumor, stroke, etc.): NA

other major psychopathology: NA

origin country (or ethnicity): United Kingdom

method observational

instruments used in order to quantify the variables

Social cognition aspect: emotion recognition

Name of the task: Forced-choice labelling of facial expressions (‘Ekman 60’; Calder et al., 1996)

type of stimulus [face/voice etc., Ekman faces/other etc.]: 10 models (6 females) were selected from the Ekman and Friesen (1976) Pictures of Facial Affect series

task condition: anger, disgust, fear, sadness, happiness and surprise

operationalization: participants were asked to select one of six emotion labels (anger, disgust, fear, sadness, happiness, surprise) that best described the facial expression shown. Sum score of correct responses.

Main findings related to the review's scope

significant interaction between emotion and group: within-subject - the PD patients showed impaired recognition of anger but not of any other expression; between subject - it was better than the controls'.

Tags: Emotion recognition, behavioral

Impaired recognition of prosody and subtle emotional facial expressions in Parkinson's disease.

**Item Type**	Journal Article
**Author**	Sharon L. Buxton
**Author**	Lorraine MacDonald
**Author**	Lynette J. Tippett
**Abstract**	Accurately recognizing the emotional states of others is crucial for successful social interactions and social relationships. Individuals with Parkinson's disease (PD) have shown deficits in emotional recognition abilities although findings have been inconsistent. This study examined recognition of emotions from prosody and from facial emotional expressions with three levels of subtlety, in 30 individuals with PD (without dementia) and 30 control participants. The PD group were impaired on the prosody task, with no differential impairments in specific emotions. PD participants were also impaired at recognizing facial expressions of emotion, with a significant association between how well they could recognize emotions in the two modalities, even after controlling for disease severity. When recognizing facial expressions, the PD group had no difficulty identifying prototypical Ekman and Friesen (1976) emotional faces, but were poorer than controls at recognizing the moderate and difficult levels of subtle expressions. They were differentially impaired at recognizing moderately subtle expressions of disgust and sad expressions at the difficult level. Notably, however, they were impaired at recognizing happy expressions at both levels of subtlety. Furthermore how well PD participants identified happy expressions conveyed by either face or voice was strongly related to accuracy in the other modality. This suggests dysfunction of overlapping components of the circuitry processing happy expressions in PD. This study demonstrates the usefulness of including subtle expressions of emotion, likely to be encountered in everyday life, when assessing recognition of facial expressions.
**Date**	2013 Apr
**Language**	eng
**Extra**	Place: United States
**Volume**	127
**Pages**	193-203
**Publication**	Behavioral neuroscience
**DOI**	10.1037/a0032013
**Issue**	2
**Journal Abbr**	Behav Neurosci
**ISSN**	1939-0084 0735-7044
**PMID**	23565934
**Date Added**	6.7.2025, 19:09:38
**Modified**	5.9.2025, 14:29:55

Notes:

Included

sample characteristics

size: 30 PD and 30 HC (matched in age, years of education, gender, and estimated Full-Scale IQ)

Parkinson's Disease type and duration: idiopathic PD, Mduration=6.68 SD=5.46

Medication: on medication

Hoehn-Yahr: Md=3 (2-4)

UPDRS-3: M=28.90 SD=11.69 (12-67)

Gender (male): 20 males (67%)

averaged ages (SD, range): M=66.97 SD=7.54

other neurological disease (tumor, stroke, etc.): None

other major psychopathology: None

origin country (or ethnicity): NA

method observational

instruments used in order to quantify the variables

Social cognition aspect: emotion recognition

Name of the task: Affective Prosody Task: two tasks from the Aprosodia Battery

type of stimulus [face/voice etc., Ekman faces/other etc.]: The first set of stimuli comprised 20 semantically neutral sentences ("I am going to the other movies") spoken by a man using five different affective voice tones. The second set consisted of a monosyllabic phrase ("Bah bah bah bah bah bah bah bah") produced with the same five affective voice tones by the same man. Thus overall there were eight items per affective tone.

task condition: angry, afraid, happy, sad, and neutral

operationalization: accuracy

Name of the task: Facial Expressions of Emotion Task

type of stimulus [face/voice etc., Ekman faces/other etc.]: based on Ekman faces. included subtle as well as prototypical expressions of emotion. each morphed between the emotional expression and the neutral face (using Morpheus 1.85). Morphing involved the production of 14 increasingly more subtle emotional expressions, each level differing in intensity by 7%. The easy level has 100% intensity of expression as these are the standard Ekman and Friesen (1976) faces.

task condition: neutral, angry, disgusted, afraid, happy, sad, and surprised

operationalization: accuracy

Main findings related to the review's scope

Prosody: the PD Group was less accurate at identifying the correct emotional expression portrayed by prosody than the Control group

Overall participants were more accurate at identifying sad, angry; and surprised affective voice expression than happy and neutral expressions. Participants were also significantly more accurate at recognizing sad voice expressions than surprised. No group or interaction effects with group.

Facial emotion recognition: The PD group was significantly poorer than the Control group at identifying facial emotional expressions overall.

no difference in accuracy between the groups when identifying emotions at the easy level of the task (prototypical Ekman faces), p = .31. The PD group was, however, significantly less accurate than the Control group on the moderate (p < .001) and difficult (p < .001) levels of the task, when the emotional expressions were more subtle.

There were no significant differences between the performances of the two groups at identifying emotional expressions at the easy level of difficulty. In contrast the PD group was significantly poorer than the Control group at identifying happy expressions at the moderate and difficult levels of emotion (p = .002 and p < .001, respectively) and poorer at recognizing sad expressions at the most difficult level only (p = .03). Additionally, the PD group was significantly poorer than the Control group at identifying disgusted expressions only at the moderate level of difficulty (p < .001)

Tags: Emotion recognition, behavioral

Impairment in Theory of Mind in Parkinson's Disease Is Explained by Deficits in Inhibition.

Item Type Journal Article
Author Jennifer A. Foley
Author Claire Lancaster
Author Elena Poznyak
Author Olga Borejko
Author Elaine Niven
Author Thomas Foltynie
Author Sharon Abrahams
Author Lisa Cipolotti
Abstract OBJECTIVE: Several studies have reported that people with Parkinson's disease (PD) perform poorly on tests of 'Theory of Mind' (ToM), suggesting impairment in the ability to understand and infer other people's thoughts and feelings. However, few studies have sought to separate the processes involved in social reasoning from those involved in managing the inhibitory demands on these tests. In this study, we investigated the contribution of inhibition to ToM performance in PD. METHODS: 18 PD patients and 22 age-matched healthy controls performed a ToM test that separates the ability to infer someone else's perspective from the ability to inhibit one's own. Participants also completed a battery of standard measures of social and executive functioning, including measures of inhibition. RESULTS: The PD patients performed worse on the ToM test only when the inhibitory demands were high. When the level of inhibition required was reduced, there were no significant group differences. Furthermore, executive impairments in PD patients were limited to

measures of inhibition, with disadvantages associated with poorer ToM performance in this group. CONCLUSIONS: This study provides convincing evidence that the apparent impairment observed on ToM tests in PD is explained by deficits in inhibition.

**Date**	2019
**Language**	eng
**Extra**	Place: United States
**Volume**	2019
**Pages**	5480913
**Publication**	Parkinson's disease
**DOI**	10.1155/2019/5480913
**Journal Abbr**	Parkinsons Dis
**ISSN**	2090-8083 2042-0080
**PMID**	31275544
**PMCID**	PMC6558602
**Date Added**	6.7.2025, 19:09:40
**Modified**	14.7.2025, 15:08:43

Notes:

Included

sample characteristics

size: 18 PD and 22 HC

Parkinson's Disease type and duration: idiopathic PD, Mduration = 6.11 SD= 3.07

Medication: on medication

Hoehn-Yahr: NA

UPDRS-3: NA

Gender (male): 10 males (56%)

averaged ages (SD, range): M= 63.83 SD= 10.73

other neurological disease (tumor, stroke, etc.): None

other major psychopathology: None

origin country (or ethnicity): United Kingdom

method observational

instruments used in order to quantify the variables

Social cognition aspect: Theory of mind and emotion recognition

Name of the task: the Reading the Mind in the Eyes Test, Revised Version (RMET)

type of stimulus [face/voice etc., Ekman faces/other etc.]: identify their mental state from one of the four possible responses.

operationalization: accuracy

Name of the task: the Ekman 60 Faces

type of stimulus [face/voice etc., Ekman faces/other etc.]: the faces of 10 actors and asked to identify the emotion expressed from one of six possible responses

task condition: happiness, sadness, disgust, fear, surprise, and anger

operationalization: accuracy

Main findings related to the review's scope

PD perfomed worse than HC in RMET, but not on the Ekman test

Tags: Emotion recognition, ToM, behavioral

Impairment of Emotional Memory and Ability to Identify Emotional States in Patients with Parkinson's Disease

**Item Type**	Journal Article
**Author**	Zh. M. Glozman
**Author**	O. S. Levin
**Author**	N. Yu. Lycheva
**Date**	11/2003
**Language**	en
**Library Catalog**	DOI.org (Crossref)
**URL**	https://link.springer.com/10.1023/B:HUMP.0000008842.71661.20
**Accessed**	11.8.2025, 19:24:01
**License**	https://www.springernature.com/gp/researchers/text-and-data-mining
**Volume**	29
**Pages**	707-711
**Publication**	Human Physiology
**DOI**	10.1023/B:HUMP.0000008842.71661.20
**Issue**	6
**Journal Abbr**	Human Physiology
**ISSN**	0362-1197, 1608-3164
**Date Added**	11.8.2025, 19:24:01
**Modified**	11.8.2025, 19:24:01

Notes:

Included

sample characteristics

size: 60 PD and 10 HC Matched in education

Parkinson's Disease type and duration: NA

Medication: NA

Hoehn-Yahr: M= 2.2 SD= 0.4 1.5-3

UPDRS-3: NA

Gender (male): 32 males (53%)

averaged ages (SD, range): M= 62.0 SD= 6.7 48-77

other neurological disease (tumor, stroke, etc.): NA

other major psychopathology: NA

origin country (or ethnicity): NA

method observational

instruments used in order to quantify the variables

Social cognition aspect: emotion recognition

Name of the task: NA

type of stimulus [face/voice etc., Ekman faces/other etc.]: Twelve pictures with faces of humans in different emotional states

task conditions: happiness, joy, pleasure, delight, astonishment, tranquility, indifference, anger, anxiety, fear, and suffering

operationalization: classify the pictures according to three emotional states (negative, positive, and neutral >> accuracy

Main findings related to the review's scope

PD significantly more often incorrectly interpreted emotional states shown in pictures, particularly negative emotions.

Tags: Emotion recognition, behavioral

Impairment of odor recognition in Parkinson's disease caused by weak activations of the orbitofrontal cortex

Item Type Journal Article

Author Yuri Masaoka
Author Nahoko Yoshimura
Author Manabu Inoue
Author Mitsuru Kawamura
Author Ikuo Homma

Abstract Olfactory dysfunction and abnormalities of olfactory brain structures are found in patients with Parkinson's disease (PD), and a number of studies have reported that olfactory dysfunction is caused by abnormalities of the central olfactory systems. We previously analyzed electroencephalograms (EEGs) and respiration simultaneously in normal subjects while testing for detection and recognition of odors. We identified changes in respiration pattern in response to odor stimuli and found inspiratory phase-locked alpha oscillations (I-a). The genesis of I-a were identified in olfactoryrelated areas including the entorhinal cortex, hippocampus, amygdale and orbitofrontal cortex with an EEG dipole tracing method. In the present study, we used the same protocol in PD patients and compared results of PD with those of age-matched controls. All PD patients detected odor, but 5 out of 10 showed impaired odor recognition. Changes in breathing pattern associated with emotional changes during exposure to odor stimuli were not observed in PD patients. I-a waveforms were not observed; however, positive waves followed by negative waves were identified approximately 100 ms after inspiration onset. Dipoles of this component were localized in the entorhinal cortex for odor detection in all patients and in the entorhinal cortex and middle temporal gyrus for PD patients who Could discriminate odors. Odor recognition in PD could be subserved by a different neural circuit from that of normal Subjects, done through the temporal association cortex as a subsystem for recognizing the odor; however, the system may not be associated with the odor-induced emotions. (c) 2006 Elsevier Ireland Ltd. All rights reserved.

Date 2007-01-22
Language English
Extra Place: ELSEVIER HOUSE, BROOKVALE PLAZA, EAST PARK SHANNON, CO, CLARE, 00000, IRELAND Type: Article
Volume 412
Publisher ELSEVIER IRELAND LTD
Pages 45-50
Publication NEUROSCIENCE LETTERS
DOI 10.1016/j.neulet.2006.10.055
Issue 1
ISSN 0304-3940
Date Added 14.7.2025, 14:50:43
Modified 5.9.2025, 14:45:34

Notes:

Not Included: Does not study PD

Tags: EXCLUDED

[Impairment of social cognition in Parkinson's disease].

Item Type Journal Article
Author Nahoko Yoshimura
Author Mitsuru Kawamura
Abstract The characteristic four symptoms of motor deficits, bradykinesia, rigidity, resting tremor and impairment of postural reflex with Parkinson's disease (PD) are accompanied by specific cognitive deficits. Deficits of executive functions, skill learning, and visuospatial cognition are well known. The results of our recent studies show the deficit of social cognition which include facial expression recognition, sensation of gaze direction, "theory of mind" in comprehension, and decision making. These symptoms can be explained by the damage of the amygdala and/or its connection, which are known to play a crucial role in social cognition, it is attributed to the involvement of amygdala and it's connection to the frontal lobe in PD. The impairment of social cognition is an important mental change associated with PD.
Date 2005 Feb
Language jpn
Extra Place: Japan
Volume 57
Pages 107-113
Publication No to shinkei = Brain and nerve
Issue 2
Journal Abbr No To Shinkei
ISSN 0006-8969
PMID 15856756
Date Added 6.7.2025, 19:09:42
Modified 5.9.2025, 15:01:48

Notes:

Not Included: not in English
Tags: EXCLUDED

Impairments in face discrimination and emotion recognition are related to aging and cognitive dysfunctions in Parkinson's disease with dementia.

Item Type Journal Article
Author Mary Wen-Reng Ho
Author Sarina Hui-Lin Chien
Author Ming-Kuei Lu
Author Jui-Cheng Chen
Author Yu Aoh
Author Chun-Ming Chen
Author Hsien-Yuan Lane
Author Chon-Haw Tsai

Abstract Patients with Parkinson's disease (PD) suffer from motor and non-motor symptoms; 40% would develop dementia (PD-D). Impaired face and emotion processing in PD has been reported; however, the deficits of face processing in PD-D remain unclear. We investigated three essential aspects of face processing capacity in PD-D, and the associations between cognitive, neuropsychiatric assessments and task performances. Twenty-four PD-D patients (mean age: 74.0 ± 5.55) and eighteen age-matched healthy controls (HC) (mean age: 71.0 ± 6.20) received three computerized tasks, morphing-face discrimination, dynamic facial emotion recognition, and expression imitation. Compared to HC, PD-D patients had lower sensitivity (d') and greater neural internal noises in discriminating faces; responded slower and had difficulties with negative emotions; imitated some expressions but with lower strength. Correlation analyses revealed that patients with advancing age, slow mentation, and poor cognition (but not motor symptoms) showed stronger deterioration in face perception. Importantly, these correlations were absent in the age-matched HC. The present study is among the first few examined face processing in patients with PD-D, and found consistent deficits correlated with advancing age and slow mentation. We propose that face discrimination task could be included as a potential test for the early detection of dementia in PD.

Date 2020 Mar 9
Language eng
Extra Place: England
Volume 10
Pages 4367
Publication Scientific reports
DOI 10.1038/s41598-020-61310-w
Issue 1
Journal Abbr Sci Rep
ISSN 2045-2322
PMID 32152359
PMCID PMC7062803
Date Added 6.7.2025, 19:09:35
Modified 5.9.2025, 14:39:26

Notes:

Included

sample characteristics

size: 24 PD with dementia (PDD) and 18 HC age-matched

Parkinson's Disease type and duration: NA

Medication: OFF medication

Hoehn-Yahr: NA

UPDRS-3: M=32.22 SD= 16.93

Gender (male): 13 males (54%)

averaged ages (SD, range): M=74.0 SD= 5.55 (62-81)

other neurological disease (tumor, stroke, etc.): None

other major psychopathology: NA

origin country (or ethnicity): China

method (Review, meta-analysis or observational and/or self-reported):

instruments used in order to quantify the variables

Social cognition aspect: emotion recognition

Name of the task: Dynamic facial emotion recognition.

type of stimulus [face/voice etc., Ekman faces/other etc.]: female and male stimuli. A total of 12 color dynamic facial emotion GIF videos were created by morphing the neutral face (0% intensity) with the six basic emotions

task condition: neutral, Anger, Disgust, Fear, Happy, Sad, and Surprise

operationalization: Participants were told to answer orally at any time when they recognized the emotion. >> accuracy and RT

Main findings related to the review's scope

Accuracy

the HC group had a higher overall accuracy than the PD-D group

Beyond group, From high to low, Happy, Sad, Anger, Suprise, Disgust, and Fear

the HC group performed significantly better in perceiving Anger, Disgust, Sad, and Surprise, than the PD-D group.

RT

The PD-D group responded markedly slower than the HC group

Beyond group, from fast to slow, the mean RT for Happy, Sad, Anger, Suprise, Fear and Disgust

the HC group were significantly faster in recognizing Happy, Anger, Suprise, Fear, and Disgust.
Tags: Emotion recognition, behavioral

Impairments in social cognition in early medicated and unmedicated Parkinson disease.

**Item Type**	Journal Article
**Author**	María Roca
**Author**	Teresa Torralva
**Author**	Ezequiel Gleichgerrcht
**Author**	Anabel Chade
**Author**	Gonzalo Gómez Arévalo
**Author**	Oscar Gershanik
**Author**	Facundo Manes
**Abstract**	BACKGROUND: Theory of mind (ToM) refers to the ability to infer others' mental states, including intentions and feelings, and is considered to be a critical part of social cognition. Earlier studies in individuals with Parkinson disease (PD) have shown ToM deficits in the more advanced stages of the disease. There is currently no evidence of social cognition deficits in patients in the early stages of PD. METHODS: In this study, we compared patients with early PD (n=36) and a control group of healthy subjects (n=36). Patients were assessed with 2 ToM tasks designed to differentially detect subtle deficits in the affective and cognitive aspects of ToM. Patients were also assessed with a complete neuropsychologic battery which included classic executive tests aimed at investigating the relationship between ToM and executive functions. Performance of medicated (n=16) and unmedicated (n=20) patients was also compared. RESULTS: Our results are the first to indicate that ToM is affected in the early stages of PD. As has already been reported in more advanced stages of PD, such deficits seem to be related to the cognitive aspects of this domain. In our study, these deficits were not related with performance on executive functioning, depression, or medication usage. CONCLUSIONS: These results provide evidence for ToM impairments early in the course of PD. Recognition of ToM impairments in early PD is important, as these deficits may impact patients' social interactions and quality of life.
**Date**	2010 Sep
**Language**	eng
**Extra**	Place: United States
**Volume**	23
**Pages**	152-158
**Publication**	Cognitive and behavioral neurology : official journal of the Society for Behavioral and Cognitive Neurology
**DOI**	10.1097/WNN.0b013e3181e078de
**Issue**	3
**Journal Abbr**	Cogn Behav Neurol
**ISSN**	1543-3641 1543-3633
**PMID**	20829664

Date Added 6.7.2025, 19:09:34
Modified 5.9.2025, 14:53:24

Notes:

Included

Sample characteristics

Size: 36 PD (early) -> 16 PD-med (medicated), 20 PD-nomed (unmedicated), 35 HC

PD-type: NA

PD-duration: PD-med: M = 1.69, SD = 1.55; PD-nomed: M 0 1.23, MSDd = 1.56

Medication: LEDD mentioned

Hoehn-Yahr: Range = 1-2; PD-med: M = 1.42, SD = 0.57; PD-nomed: M = 1.33, SD = 0.54

UPDRS-3: NA

Gender (male): NA

Age: PD-med: M = 63.4, SD = 8.47; PD-nomed: M = 63.5, SD = 11.8

Other neurological disease (tumor, stroke, etc.): none

Other major psychopathology: none

Origin country (or ethnicity): Argentina

method behavioural

instruments used in order to quantify the variables

Social cognition aspect: affective ToM

Name of the task: Reading the mind in the eyes test

Type of stimulus [face/voice etc., Ekman faces/other etc.]: 15 affective stimuli of the original task

Task condition: choose between 2 adjectives for the one that best described what the individual in the picture was thinking or feeling

Operationalization: Total score for this task was calculated as the number of items correctly identified.

Social cognition aspect: ToM

Name of the task: Faux pas test

Type of stimulus [face/voice etc., Ekman faces/other etc.]: participants read a story that may or may not contain a social faux pas. 10 stories

Task condition: priate had been said, and if so, why it was inappropriate. Performance was scored regarding the adequate identification of the faux pas (hits) and the adequate rejection of those stories which did not contain a faux pas (rejects).

When a faux pas was correctly identified, subjects were also asked 2 additional questions to measure intentionality—

Operationalization: The score was 1 point for each faux pas correctly identified (maximum: 10), or non-faux pas correctly rejected (maximum: 10).

From 2 question in faux-pas:

Recognizing that the person committing the faux pas was unaware that they had said something inappropriate (maximum 10)—and emotional attribution, in which participants should recognize that the person hearing the faux pas might have felt hurt or insulted (maximum 10).

Main findings related to the review's scope

RMET

When comparing total performance between PD patients and controls, no significant differences were found on the Mind in the Eyes task (U= 285.5, P= 0.12).

When performance was compared between medicated and unmedicated PD patients, as well as control subjects, no significant difference were found across the groups (w2= 2.45, df = 2, P= 0.29) on the total score of the Mind in the Eyes.

Faux-Pas

When comparing performance between PD patients and control subjects, a significant effect was found on the Faux Pas Total Score (U = 242.5.0, P = 0.017)

Significant differences also emerged on the hits (U = 437.00, P = 0.04) and on the intentionality scores (U = 183.0, P<0.01), whereas no significant differences were observed either on the reject score (U = 490.0, P= 0.13) or on the emotional attribution score (U = 386.5, P = 0.13).

when performance was analyzed between medicated and unmedicated PD patients, as well as control subjects, no significant differences were found across the groups on the hits (w2= 2.20, df = 2, P= 0.33) or rejects (w2= 2.81, df = 2, P = 0.25) scores on the Faux Pas. However, when analyzing the total score on this task, a significant difference was found across the groups (w2= 6.98, df = 2, P= 0.03), with controls significantly outperforming medicated (U = 89.0, P<0.01) but not unmedicated (U = 153.5, P = 0.14) PD patients.

When analyzing the affective and cognitive subcomponents of the Faux Pas separately, again, no significant

differences were found on the affective aspects as measured by the emotion attribution score (w2= 0.86, df = 2, P = 0.65). In contrast, a significant difference was indeed found for the intention attribution scores across the 3 groups (w2= 49.0, df = 2, P<0.01), with controls significantly outperforming both medicated (U = 75.0, P = 0.044) and unmedicated (U = 49.0, P<0.001) groups of PD patients. No significant differences were found between the PD patient groups (U = 122.0, P = 0.63).

Tags: ToM, behavioral

Implicit and explicit emotional processing in Parkinson's disease.

**Item Type**	Journal Article
**Author**	Céline Borg
**Author**	Nathalie Bedoin
**Author**	Soline Bogey
**Author**	George A. Michael
**Author**	Aurélia Poujois

Author Bernard Laurent
Author Catherine Thomas-Antérion
Abstract INTRODUCTION: Our study investigated the ability of nondemented Parkinson's disease (PD) patients to explicitly identify emotional words and to show implicit sensitivity to these emotions in a task that did not require emotional processing. METHODS: Twelve PD patients and 12 healthy controls, matched for age and education, performed lexical decision (LD) and emotional categorisation tasks (fear, disgust, and happiness) on the same words. RESULTS: PD patients were specifically impaired in the explicit identification of disgust with a decreased accuracy in LD. However, a slowdown in LD latency in both PD patients and the control group suggested the persistence of emotional sensitivity to disgust. CONCLUSION: Despite the persistence of an automatic capture by the emotional content of disgust, PD patients may suffer from emotional deficits in recognising both the emotional and semantic components of words, resulting in blunted emotional responses.
Date 2012
Language eng
Extra Place: England
Volume 34
Pages 289-296
Publication Journal of clinical and experimental neuropsychology
DOI 10.1080/13803395.2011.639296
Issue 3
Journal Abbr J Clin Exp Neuropsychol
ISSN 1744-411X 1380-3395
PMID 22229340
Date Added 6.7.2025, 19:09:39
Modified 5.9.2025, 14:29:26

Notes:

Not Included: not on SC

Tags: EXCLUDED

Implicit and explicit processing of emotional facial expressions in Parkinson's disease.

Item Type Journal Article
Author Caroline Wagenbreth
Author Lena Wattenberg
Author Hans-Jochen Heinze
Author Tino Zaehle
Abstract OBJECTIVE: Besides motor problems, Parkinson's disease (PD) is associated with detrimental emotional and cognitive functioning. Deficient explicit emotional processing has been observed, whilst patients also show impaired Theory of Mind (ToM) abilities. However, it is unclear whether this PD patients' ToM deficit is based on an inability to infer otherś emotional states or whether it is due to explicit

emotional processing deficits. We investigated implicit and explicit emotional processing in PD with an affective priming paradigm in which we used pictures of human eyes for emotional primes and a lexical decision task (LDT) with emotional connoted words for target stimuli. METHOD: Sixteen PD patients and sixteen matched healthy controls performed a LTD combined with an emotional priming paradigm providing emotional information through the facial eye region to assess implicit emotional processing. Second, participants explicitly evaluated the emotional status of eyes and words used in the implicit task. RESULTS: Compared to controls implicit emotional processing abilities were generally preserved in PD with, however, considerable alterations for happiness and disgust processing. Furthermore, we observed a general impairment of patients for explicit evaluation of emotional stimuli, which was augmented for the rating of facial expressions. CONCLUSIONS: This is the first study reporting results for affective priming with facial eye expressions in PD patients. Our findings indicate largely preserved implicit emotional processing, with a specific altered processing of disgust and happiness. Explicit emotional processing was considerably impaired for semantic and especially for facial stimulus material. Poor ToM abilities in PD patients might be based on deficient explicit emotional processing, with preserved ability to implicitly infer other people's feelings.

Date 2016 Apr 15
Language eng
License Copyright © 2016 Elsevier B.V. All rights reserved.
Extra Place: Netherlands
Volume 303
Pages 182-190
Publication Behavioural brain research
DOI 10.1016/j.bbr.2016.01.059
Journal Abbr Behav Brain Res
ISSN 1872-7549 0166-4328
PMID 26850933
Date Added 6.7.2025, 19:09:39
Modified 5.9.2025, 15:00:29

Notes:

Not Included: Operationalization does not assess SC

Tags: EXCLUDED

Improving functional disability and cognition in Parkinson disease: randomized controlled trial.

Item Type Journal Article
Author Javier Peña
Author Naroa Ibarretxe-Bilbao
Author Inés García-Gorostiaga
Author Maria Angeles Gomez-Beldarrain
Author María Díez-Cirarda

Author Natalia Ojeda

Abstract OBJECTIVES: To examine the efficacy of an integrative cognitive training program (REHACOP) to improve cognition, clinical symptoms, and functional disability of patients with Parkinson disease (PD). METHODS: Forty-two patients diagnosed with PD in Hoehn & Yahr stages 1 to 3 were randomly assigned to either the cognitive training group (REHACOP) or the control group (occupational activities) for 3 months (3 sessions, 60 min/wk). Primary outcomes were change on processing speed, verbal memory, visual memory, executive functioning, and theory of mind. Secondary outcomes included changes on neuropsychiatric symptoms, depression, apathy, and functional disability. The trial was registered with clinicaltrials.gov (NCT02118480). RESULTS: No baseline group differences were found. Bootstrapped analysis of variance results showed significant differences in the mean change scores between the REHACOP group and control group in processing speed (0.13 [SE = 0.07] vs -0.15 [SE = 0.09], p = 0.025), visual memory (0.10 [SE = 0.10] vs -0.24 [SE = 0.09], p = 0.011), theory of mind (1.00 [SE = 0.37] vs -0.27 [SE = 0.29], p = 0.013), and functional disability (-5.15 [SE = 1.35] vs 0.53 [SE = 1.49], p = 0.012). CONCLUSIONS: Patients with PD receiving cognitive training with REHACOP demonstrated statistically significant and clinically meaningful changes in processing speed, visual memory, theory of mind, and functional disability. Future studies should consider the long-term effect of this type of intervention. These findings support the integration of cognitive training into the standard of care for patients with PD. CLASSIFICATION OF EVIDENCE: This study provides Class II evidence that for patients with PD, an integrative cognitive training program improves processing speed, visual memory, theory of mind, and functional disability.

Date 2014 Dec 2
Language eng
License © 2014 American Academy of Neurology.
Extra Place: United States
Volume 83
Pages 2167-2174
Publication Neurology
DOI 10.1212/WNL.0000000000001043
Issue 23
Journal Abbr Neurology
ISSN 1526-632X 0028-3878
PMID 25361785
PMCID PMC4276404
Date Added 6.7.2025, 19:09:41
Modified 5.9.2025, 14:49:54

Notes:

Not Included: Evaluation of a training program for PD. No HC

Tags: EXCLUDED

In vivo cholinergic basal forebrain degeneration and cognition in Parkinson's disease: Imaging results from the COPPADIS study

**Item Type**	Journal Article
**Author**	Michel J. Grothe
**Author**	Miguel A. Labrador-Espinosa
**Author**	Silvia Jesus
**Author**	Daniel Macias-Garcia
**Author**	Astrid Adarmes-Gomez
**Author**	Fatima Carrillo
**Author**	Elena Iglesias Camacho
**Author**	Pablo Franco-Rosado
**Author**	Florinda Roldan Lora
**Author**	Juan Francisco Martin-Rodriguez
**Author**	Miquel Aguilar Barbera
**Author**	Pau Pastor
**Author**	Sonia Escalante Arroyo
**Author**	Berta Solano Vila
**Author**	Anna Cots Foraster
**Author**	Javier Ruiz Martinez
**Author**	Francisco Carrillo Padilla
**Author**	Mercedes Pueyo Morlans
**Author**	Isabel Gonzalez Aramburu
**Author**	Jon Infante Ceberio
**Author**	Jorge Hernandez Vara
**Author**	Oriol de Fabregues-Boixar
**Author**	Teresa de Deus Fonticoba
**Author**	Berta Pascual-Sedano
**Author**	Jaime Kulisevsky
**Author**	Pablo Martinez-Martin
**Author**	Diego Santos-Garcia
**Author**	Pablo Mir
**Author**	COPPADIS Study Grp
**Abstract**	Introduction: We aimed to assess associations between multimodal neuroimaging measures of cholinergic basal forebrain (CBF) integrity and cognition in Parkinson's disease (PD) without dementia. Methods: The study included a total of 180 non-demented PD patients and 45 healthy controls, who underwent structural MRI acquisitions and standardized neurocognitive assessment through the PD-Cognitive Rating Scale (PD-CRS) within the multicentric COPPADIS-2015 study. A subset of 73 patients also had Diffusion Tensor Imaging (DTI) acquisitions. Volumetric and microstructural (mean diffusivity, MD) indices of CBF degeneration were automatically extracted using a stereotactic CBF atlas. For comparison, we also assessed multimodal indices of hippocampal degeneration. Associations between imaging measures and cognitive performance were assessed using linear models. Results: Compared to controls, CBF volume was not significantly reduced in PD patients as a group. However, across PD patients lower CBF volume was significantly associated with lower global cognition (PD-CRStotal: r = 0.37, p < 0.001), and this association remained significant after controlling for several potential confounding variables (p = 0.004). Analysis of individual item scores showed that this association spanned executive and memory domains. No analogue cognition associations were observed for CBF MD. In covariate-controlled models, hippocampal volume was not

associated with cognition in PD, but there was a significant association for hippocampal MD (p = 0.02). Conclusions: Early cognitive deficits in PD without dementia are more closely related to structural MRI measures of CBF degeneration than hippocampal degeneration. In our multicentric imaging acquisitions, DTI-based diffusion measures in the CBF were inferior to standard volumetric assessments for capturing cognition-relevant changes in non-demented PD.

Date 2021-07
Language English
Extra Place: THE BOULEVARD, LANGFORD LANE, KIDLINGTON, OXFORD OX5 1GB, OXON, ENGLAND Type: Article
Volume 88
Publisher ELSEVIER SCI LTD
Pages 68-75
Publication PARKINSONISM & RELATED DISORDERS
DOI 10.1016/j.parkreldis.2021.05.027
ISSN 1353-8020
Date Added 14.7.2025, 14:50:30
Modified 5.9.2025, 14:37:12

Notes:

Not Included: not on SC

Tags: EXCLUDED

Influence of continuous subcutaneous apomorphine infusion on cognition and behavior in Parkinson's disease: A systematic review

Item Type Journal Article
Author J. F. Houvenaghel
Author M. Meyer
Author E. Schmitt
Author A. Arifi
Author E. Benchetrit
Author A. Bichon
Author C. Cau
Author L. Lavigne
Author E. Le Mercier
Author V. Czernecki
Author K. Dujardin
Date 2025-03
Volume 181
Pages 156-171
Publication REVUE NEUROLOGIQUE
DOI 10.1016/j.neurol.2024.06.008

Issue 3
ISSN 0035-3787
Date Added 14.7.2025, 14:48:38
Modified 5.9.2025, 14:39:34

Notes:

<u>**Not Included:**</u> although being systematic review, included only one paper on SC.

Tags: EXCLUDED

Intact emotion recognition and experience but dysfunctional emotion regulation in idiopathic Parkinson's disease.

Item Type Journal Article
Author Rottraut Ille
Author Albert Wabnegger
Author Petra Schwingenschuh
Author Petra Katschnig-Winter
Author Mariella Kögl-Wallner
Author Karoline Wenzel
Author Anne Schienle
Abstract BACKGROUND: A specific non-motor impairment in Parkinson's disease (PD) concerns difficulties to accurately identify facial emotions. Findings are numerous but very inconsistent, ranging from general discrimination deficits to problems for specific emotions up to no impairment at all. By contrast, only a few studies exist about emotion experience, altered affective traits and states in PD. OBJECTIVE: To investigate the decoding capacity for affective facial expressions, affective experience of emotion-eliciting images and affective personality traits in PD. METHODS: The study sample included 25 patients with mild to moderate symptom intensity and 25 healthy controls (HC) of both sexes. The participants were shown pictures of facial expressions depicting disgust, fear, and anger as well as disgusting and fear-relevant scenes. Additionally, they answered self-report scales for the assessment of affective traits. RESULTS: PD patients had more problems in controlling anger and disgust feelings than HC. Higher disgust sensitivity in PD was associated with lower functioning in everyday life and lower capacity to recognize angry faces. Furthermore, patients reported less disgust towards poor hygiene and spoiled food and they stated elevated anxiety. However, the clinical group displayed intact facial emotion decoding and emotion experience. Everyday life functionality was lowered in PD and decreased with stronger motor impairment. Furthermore, disease duration was negatively associated to correct classification of angry faces. CONCLUSIONS: Our data indicate that problems with emotion regulation may appear already in earlier disease stages of PD. By contrast, PD patients showed appropriate emotion recognition and experience. However, data also point to a deterioration of emotion recognition capacity with the course of the disease. Compensatory mechanisms in PD patients with less advanced disease are discussed.
Date 2016 Feb 15
Language eng

License Copyright © 2015 The Authors. Published by Elsevier B.V. All rights reserved.
Extra Place: Netherlands
Volume 361
Pages 72-78
Publication Journal of the neurological sciences
DOI 10.1016/j.jns.2015.12.007
Journal Abbr J Neurol Sci
ISSN 1878-5883 0022-510X
PMID 26810520
Date Added 6.7.2025, 19:09:34
Modified 5.9.2025, 14:40:07

Notes:

Included

sample characteristics

size: 25 PD and 25 HC matched in sex age and education years

Parkinson's Disease type and duration: Idiopathic PD Mduration = M=6.0 SD=3.0

Medication: On medication

Hoehn-Yahr: M=2.10 SD=0.28 (2-3)

UPDRS-3: M= 34.2 SD =8.5

Gender (male): 15 males (60%)

averaged ages (SD, range): M =56.1 SD=8.5

other neurological disease (tumor, stroke, etc.): Dementia excluded

other major psychopathology: No depression

origin country (or ethnicity): Austria

method observational

instruments used in order to quantify the variables

Social cognition aspect: emotion recognition

Name of the task: NA; from the Karolinska set

type of stimulus [face/voice etc., Ekman faces/other etc.]: 30 pictures with emotional facial expressions (Equal sex)

task condition: fear, anger, and disgust

operationalization: For each facial expression (fear, disgust, anger), subjects rated how intense the depicted person had experienced the five basic emotions (happiness, fear, sadness, anger, and disgust; e.g., 'Please indicate how intense the depicted person experienced disgust': 1 = very little; 9 = very intense). intensity and recognition accuracy for fearful, disgusted, and angry faces.

Main findings related to the review's scope

Intensity Ratings

PD patients rated higher fear intensity for fearful faces than HC (d = 0.52).

However, groups did not differ in intensities of target emotions for disgusted and angry faces.

Recognition Accuracy

PD patients and HC did not differ in classification accuracy for facial disgust, fear, and anger.

Tags: Emotion recognition, behavioral

Intact recognition of facial emotion in Parkinson's disease.

Item Type Journal Article
Author R. Adolphs
Author R. Schul
Author D. Tranel
Abstract Although the basal ganglia have been shown to be critical for the expression of emotion in prosody and facial expressions, it is unclear whether they are also critical for recognition of emotions. Selective pathology of parts of the basal ganglia is a hallmark of individuals with Parkinson's disease, and such patients have been examined in several studies of emotion. We examined 18 patients with Parkinson's disease (11 men, 7 women) and 13 age-, education-, gender ratio-, and IQ-matched normal controls on their ability to recognize emotions signaled by facial expressions. Parkinson's patients performed entirely normally on a quantitative task of recognizing emotional facial expressions. The findings do not support the notion that the sectors of basal ganglia that are dysfunctional in Parkinson's disease are essential for recognizing emotion in facial expressions.
Date 1998 Apr
Language eng
Extra Place: United States
Volume 12
Pages 253-258
Publication Neuropsychology
DOI 10.1037//0894-4105.12.2.253
Issue 2
Journal Abbr Neuropsychology
ISSN 0894-4105
PMID 9556771
Date Added 6.7.2025, 19:09:37
Modified 5.9.2025, 14:25:02

Notes:

Included

sample characteristics

size: 18 Idiopathic PD and 13 HC

PD-type: IPS

PD-duration: M = 7.8 ± 4 years (at least 2 years)

Medication: ON state

Hoehn-Yahr: at least 2 (range, 2-5)

UPDRS-3: NA

Gender: 11 male (61%)

Age: M=63.67, SD=11.28, Range: 39-83

Other neurological disease (tumor, stroke, etc.): NA

Other major psychopathology: None of the patients had dementia

origin country (or ethnicity): NA

method observational

instruments used in order to quantify the variables

Social cognition aspect: emotion recognition

Name of the task: NA

type of stimulus [face/voice etc., Ekman faces/other etc.]: black-and-white slides of emotional expressions - Ekman faces

task condition: anger, fear, happiness, surprise, sadness, disgust and neutral

operationalization: each stimulus was ranked on a scale of 0-5 (0 = not at all, 5 = very much) with respect to the following six labels in random order: happy, sad, disgusted, angry, afraid, and surprised. The correlation with normal mean rating (high correlation = the ratings were similar to those given by normal controls)

Main findings related to the review's scope

happy faces were recognized better than faces depicting any other emotion. Controls and patients with PD thus performed very similarly in rating the emotions signalled by facial expressions.

PD patients performed normally with respect to recognition of all of the six basic emotions.

Tags: Emotion recognition, behavioral

Integrating IoMT and AI for Proactive Healthcare: Predictive Models and Emotion Detection in Neurodegenerative Diseases

Item Type Journal Article
Author Virginia Sandulescu
Author Marilena Ianculescu
Author Liudmila Valeanu
Author Adriana Alexandru
Date 2024-09
Volume 17
Publication ALGORITHMS
DOI 10.3390/a17090376
Issue 9
Date Added 14.7.2025, 14:48:38
Modified 5.9.2025, 14:55:03

Notes:

Not Included: not on SC
Tags: EXCLUDED

Intensity-dependent facial emotion recognition and cognitive functions in Parkinson's disease.

Item Type Journal Article
Author Francesca Assogna
Author Francesco E. Pontieri
Author Luca Cravello
Author Antonella Peppe
Author Mariangela Pierantozzi
Author Alessandro Stefani
Author Paolo Stanzione
Author Clelia Pellicano
Author Carlo Caltagirone
Author Gianfranco Spalletta
Abstract Patients with Parkinson's disease (PD) frequently display non-motor symptoms. In this study, we investigated intensity-dependent facial emotion recognition in patients with PD and healthy controls (HC), matched for age, gender, and education, and its relationship to individual cognitive domains. Seventy patients with PD and 70 HC were submitted to a clinical, neuropsychological, and psychopathological evaluation. Facial emotion recognition performance was assessed using the Penn Emotion Recognition Test (PERT). The patients with PD recognized fewer low- and high-intensity facial expressions of disgust than HC. This effect was selective, because their global ability to recognize emotions was intact. Both patients with PD and HC recognized high-intensity better than low-intensity emotions, except for disgust,

which was recognized better at low intensity. In the patients with PD, overall facial emotion recognition and selective disgust recognition performances were related to deficits in many neuropsychological domains (verbal and visuo-spatial memory, attention, praxis, and verbal fluency). The ability to recognize emotions is a complex cognitive process requiring the integrity of several functions. Therefore, it is likely that structural or functional derangement of the discrete neural pathways involved in these cognitive functions in patients with PD makes it difficult for them to recognize emotions expressed by others.

Date 2010 Sep
Language eng
Extra Place: England
Volume 16
Pages 867-876
Publication Journal of the International Neuropsychological Society : JINS
DOI 10.1017/S1355617710000755
Issue 5
Journal Abbr J Int Neuropsychol Soc
ISSN 1469-7661 1355-6177
PMID 20663240
Date Added 6.7.2025, 19:09:35
Modified 5.9.2025, 14:27:10

Notes:

Included

sample characteristics

size: 70 PD and 70 HC matched for age, gender, and education

Parkinson's Disease type and duration: idiopathic PD, Mduration=4.9 SD= 4.2

Medication: on medication

Hoehn-Yahr: NA

UPDRS-3: M=20.1 SD=9.7

Gender (male): 48 males (69%)

averaged ages (SD, range): M= 62.2 SD= 11.7

other neurological disease (tumor, stroke, etc.): None

other major psychopathology: None

origin country (or ethnicity): Italy

method observational

instruments used in order to quantify the variables

Social cognition aspect: Emotion Recognition

Name of the task: The Penn Emotion Recognition Test (PERT)

type of stimulus [face/voice etc., Ekman faces/other etc.]: digitized high-quality color pictures of 3-dimensional (3D) facial expressions of evoked or felt emotions and non-emotional or neutral expressions. FINAL PRODUCT: static, bi-dimensional picture that accurately re constructs the geometry and reproduces the facets of the human face more realistically than a simple 2D image. There are eight low-intensity and eight high intensity expressions.

task condition: happiness, sadness, anger, fear, disgust

operationalization: Accuracy

Main findings related to the review's scope

worse recognition for PD in comparison to HC in the disgust facial emotion and for neutral faces. All other the same, including global.

**neutral does not survive correction.

Tags: Emotion recognition, behavioral

Inter-hemispheric EEG coherence analysis in Parkinson's disease: assessing brain activity during emotion processing.

**Item Type**	Journal Article
**Author**	R. Yuvaraj
**Author**	M. Murugappan
**Author**	Norlinah Mohamed Ibrahim
**Author**	Kenneth Sundaraj
**Author**	Mohd Iqbal Omar
**Author**	Khairiyah Mohamad
**Author**	R. Palaniappan
**Author**	M. Satiyan
**Abstract**	Parkinson's disease (PD) is not only characterized by its prominent motor symptoms but also associated with disturbances in cognitive and emotional functioning. The objective of the present study was to investigate the influence of emotion processing on inter-hemispheric electroencephalography (EEG) coherence in PD. Multimodal emotional stimuli (happiness, sadness, fear, anger, surprise, and disgust) were presented to 20 PD patients and 30 age-, education level-, and gender-matched healthy controls (HC) while EEG was recorded. Inter-hemispheric coherence was computed from seven homologous EEG electrode pairs (AF3-AF4, F7-F8, F3-F4, FC5-FC6, T7-T8, P7-P8, and O1-O2) for delta, theta, alpha, beta, and gamma frequency bands. In addition, subjective ratings were obtained for a representative of emotional stimuli. Interhemispherically, PD patients showed significantly lower coherence in theta, alpha, beta, and gamma frequency bands than HC during emotion processing. No significant changes were found in the delta frequency band coherence. We also found that PD patients were more impaired in recognizing negative emotions (sadness, fear, anger, and disgust) than relatively positive emotions (happiness and surprise). Behaviorally, PD patients did not show impairment in emotion recognition as measured by subjective ratings. These findings suggest that PD patients may have an impairment of inter-hemispheric functional connectivity (i.e., a decline in cortical connectivity) during emotion processing. This study may increase the awareness of EEG emotional response studies in clinical practice to uncover potential neurophysiologic abnormalities.
**Date**	2015 Feb
**Language**	eng
**Extra**	Place: Austria
**Volume**	122
**Pages**	237-252
**Publication**	Journal of neural transmission (Vienna, Austria : 1996)
**DOI**	10.1007/s00702-014-1249-4
**Issue**	2
**Journal Abbr**	J Neural Transm (Vienna)
**ISSN**	1435-1463 0300-9564
**PMID**	24894699
**Date Added**	6.7.2025, 19:09:39
**Modified**	5.9.2025, 15:02:28

Notes:

Not Included: Same behavioural results as previous study
(On the analysis of EEG power, frequency and asymmetry in Parkinson's disease during emotion processing.)

Tags: EXCLUDED

Intuition: A social cognitive neuroscience approach.

**Item Type**	Journal Article
**Author**	Matthew D. Lieberman
**Date**	2000
**Language**	en
**Short Title**	Intuition
**Library Catalog**	Crossref
**URL**	https://doi.apa.org/doi/10.1037/0033-2909.126.1.109
**Accessed**	13.7.2025, 20:20:49
**Volume**	126
**Publisher**	American Psychological Association (APA)
**Pages**	109-137
**Publication**	Psychological Bulletin
**DOI**	10.1037/0033-2909.126.1.109
**Issue**	1
**ISSN**	1939-1455, 0033-2909
**Date Added**	13.7.2025, 20:20:49
**Modified**	13.7.2025, 20:20:49

Notes:

Not Included: not a systematic review. Note: shows conceptual ideas for the neural origin on social cognition (to some extant)

Tags: EXCLUDED

Investigating the contribution of white matter hyperintensities and cortical thickness to empathy in neurodegenerative and cerebrovascular diseases.

**Item Type**	Journal Article
**Author**	Miracle Ozzoude
**Author**	Brenda Varriano
**Author**	Derek Beaton
**Author**	Joel Ramirez
**Author**	Melissa F. Holmes
**Author**	Christopher J. M. Scott

Author Fuqiang Gao
Author Kelly M. Sunderland
Author Paula McLaughlin
Author Jennifer Rabin
Author Maged Goubran
Author Donna Kwan
Author Angela Roberts
Author Robert Bartha
Author Sean Symons
Author Brian Tan
Author Richard H. Swartz
Author Agessandro Abrahao
Author Gustavo Saposnik
Author Mario Masellis
Author Anthony E. Lang
Author Connie Marras
Author Lorne Zinman
Author Christen Shoesmith
Author Michael Borrie
Author Corinne E. Fischer
Author Andrew Frank
Author Morris Freedman
Author Manuel Montero-Odasso
Author Sanjeev Kumar
Author Stephen Pasternak
Author Stephen C. Strother
Author Bruce G. Pollock
Author Tarek K. Rajji
Author Dallas Seitz
Author David F. Tang-Wai
Author John Turnbull
Author Dar Dowlatshahi
Author Ayman Hassan
Author Leanne Casaubon
Author Jennifer Mandzia
Author Demetrios Sahlas
Author David P. Breen
Author David Grimes
Author Mandar Jog
Author Thomas D. L. Steeves
Author Stephen R. Arnott
Author Sandra E. Black
Author Elizabeth Finger
Author Maria Carmela Tartaglia

Abstract Change in empathy is an increasingly recognised symptom of neurodegenerative diseases and contributes to caregiver burden and patient distress. Empathy impairment has been associated with brain atrophy but its relationship to white matter hyperintensities (WMH) is unknown. We aimed to investigate the relationships amongst WMH, brain atrophy, and empathy deficits in neurodegenerative and cerebrovascular diseases. Five hundred thirteen participants with Alzheimer's disease/ mild cognitive impairment, amyotrophic lateral sclerosis, frontotemporal dementia (FTD), Parkinson's disease, or cerebrovascular disease (CVD) were included. Empathy was assessed using the Interpersonal Reactivity Index. WMH were measured using a semi-automatic segmentation and FreeSurfer was used to measure cortical thickness. A heterogeneous pattern of cortical thinning was found between groups, with FTD showing thinning in frontotemporal regions and CVD in left superior parietal, left insula, and left postcentral. Results from both univariate and multivariate analyses revealed that several variables were associated with empathy, particularly cortical thickness in the fronto-insulo-temporal and cingulate regions, sex (female), global cognition, and right parietal and occipital WMH. Our results suggest that cortical atrophy and WMH may be associated with empathy deficits in neurodegenerative and cerebrovascular diseases. Future work should consider investigating the longitudinal effects of WMH and atrophy on empathy deficits in neurodegenerative and cerebrovascular diseases.

Date 2022 Jun
Language eng
License © 2022. The Author(s), under exclusive licence to American Aging Association.
Extra Place: Switzerland
Volume 44
Pages 1575-1598
Publication GeroScience
DOI 10.1007/s11357-022-00539-x
Issue 3
Journal Abbr Geroscience
ISSN 2509-2723 2509-2715
PMID 35294697
PMCID PMC9213606
Date Added 6.7.2025, 19:09:39
Modified 5.9.2025, 14:48:27

Notes:

Included

Sample characteristics

Size: 140 PD, 126 AD/MCI, 40 ALS, 52 FTD, 155 CVD (cerebrovascular disease

PD-type: idiopathic PD

PD-duration: NA

Medication: Na

Hoehn-Yahr: NA

UPDRS-3: NA

Gender (male): 109 (78%)

Age: M = 67.94 (6.34)

Other neurological disease (tumor, stroke, etc.): NA

Other major psychopathology: NA

Origin country (or ethnicity): Canada

method bevahioural/quesitonnair and imaging

instruments used in order to quantify the variables

Social cognition aspect: Empathy

Name of the task: interpersonal reactivity index (IRI)

Type of stimulus [face/voice etc., Ekman faces/other etc.]: Questionnaire

Operationalization: two subscales - EC, emotional concern and PT, perspective taking

Main findings related to the review's scope

Comparing participant and study partner ratings of EC did not reveal any difference within PD

There were significant differences between participant and study partner ratings of PT within PD; ratings showing higher participant and lower study partner PT scores.

Tags: Empathy, Questionnaire

Irony comprehension and theory of mind deficits in patients with Parkinson's disease.

Item Type Journal Article
Author Laura Monetta
Author Christopher M. Grindrod
Author Marc D. Pell

Abstract Many individuals with Parkinson's disease (PD) are known to have difficulties in understanding pragmatic aspects of language. In the present study, a group of eleven non-demented PD patients and eleven healthy control (HC) participants were tested on their ability to interpret communicative intentions underlying verbal irony and lies, as well as on their ability to infer first- and second-order mental states (i.e., theory of mind). Following Winner et al. (1998), participants answered different types of questions about the events which unfolded in stories which ended in either an ironic statement or a lie. Results showed that PD patients were significantly less accurate than HC participants in assigning second-order beliefs during the story comprehension task, suggesting that the ability to make a second-order mental state attribution declines in PD. The PD patients were also less able to distinguish whether the final statement of a story should be interpreted as a joke or a lie, suggesting a failure in pragmatic interpretation abilities. The implications of frontal lobe dysfunction in PD as a source of difficulties with working memory, mental state attributions, and pragmatic language deficits are discussed in the context of these findings.

Date 2009 Sep
Language eng
Extra Place: Italy
Volume 45
Pages 972-981
Publication Cortex; a journal devoted to the study of the nervous system and behavior
DOI 10.1016/j.cortex.2009.02.021
Issue 8
Journal Abbr Cortex
ISSN 0010-9452
PMID 19371867
Date Added 6.7.2025, 19:09:41
Modified 5.9.2025, 14:47:22

Notes:

Included

Sample characteristics

Size: 11 PD, 11 HC (matched for age, sex, education)

PD-type: Idiopathic PD

PD-duration: M = 9.1, SD = 3.2

Medication: Optimally medicated during testing

Hoehn-Yahr: Mild to moderate; M = 2.5, SD = 0.9

UPDRS-3:

Gender (male):

Age: mean age: 67.1, SD = 10.9, range: 55–86

Other neurological disease (tumor, stroke, etc.): none

Other major psychopathology: Sig. differences in depressive symptomatology between PD and HC (mainly due to 2 PD patients exhibiting mild depressive symptomatology)

Origin country (or ethnicity): Canada/native inglish speakers)

method (Review, meta-analysis or observational and/or self-reported):

instruments used in order to quantify the variables

Social cognition aspect: Facial emotion recogition

Name of the task: Benton face recognition and benton phoneme discrimination

---

Social cognition aspect: Irony comprehension / ToM

Name of the task: Pragmatic interpretation short stories

Type of stimulus [face/voice etc., Ekman faces/other etc.]: Each story (approximately 250 words in length) described a situation where one person (the witness) observes another person (the protagonist of the story) doing something sneaky (e.g., eating a muffin while the person is on a strict diet). Half of the items were lie stories and half were irony stories; the main difference between the two story types was that for lie stories, the protagonist did not realize that s/he had been caught and uttered a lie to the witness to avoid getting caught. In the irony stories, the protagonist did realize that s/he had been caught and uttered an ironic comment/joke to hide the embarrassment of being caught. Thus, the only structural difference between the stories in each condition was whether the protagonist knew or did not know that the other person knew the truth. There were six irony and six lie stories in total

Task condition: As prosody is often instrumental for conveying irony (Cheang and Pell, 2008), the final statement of each story was read in a neutral tone on the tape to avoid providing prosodic information that could help identify whether the story ended in an ironic joke or a lie. The participants' answers were recorded by the examiner on a scoresheet before continuing with the story; these responses were later graded for accuracy. Six questions were asked

1. Fact question
2. First-order belief question
3. second-order true or false belief question
4. second-order belief follow-up question
5. second-order expectation question
6. interpretation question

Operationalization: Correct answers

Main findings related to the review's scope

This analysis revealed a significant main effect of Group, reflecting that the HC participants were more accurate overall than the PD patients.

Significant two-way interactions of Group Question Type and Story Type Question Type were also revealed. Finally, the three-way interaction of Group Story Type Question Type was also significant. No other main effects or interactions were found to be significant.

1. Fact question: No sig. group diff.
2. First-order belief questions: The ANOVA revealed a main effect of Group, which was explained by the fact that PD participants were significantly less accurate than the HC participants on these questions.
3. Second-order true or false belief questions and follow-up questions: significant main effect of Group, which confirmed that the PD patients were significantly less accurate overall in answering questions based on second-order beliefs.
4. Second-order expectation questions: The ANOVA yielded no significant main or interaction effects for this question (all p's > .05
5. Interpretation questions: significant main effect of Group, confirming that the PD patients were less accurate when responding to the interpretation question. Post-hoc Tukey (HSD) comparisons on the interaction indicated that whereas the HC participants were comparable in how well they could interpret lies versus jokes, PD patients were significantly less able to recognize the intent of the irony stories

Tags: ToM, behavioral

Is "reading mind in the eyes" impaired in Parkinson's disease?

Item Type Journal Article

Author Natsuko Tsuruya
Author Mutsutaka Kobayakawa
Author Mitsuru Kawamura

Abstract Theory of mind (ToM) is the specific cognitive ability to understand other people's mental states. Several studies have examined ToM ability in patients with Parkinson's disease (PD), using the "reading mind in the eyes" test (RMET). However, there has been no agreement as to whether or not ToM ability in this task is affected by PD. In order to obtain conclusive evidence regarding ToM ability in PD, we examined RMET-related performance in nondemented PD patients; we also used the semantic discrimination task to exclude the possibility that PD patients had difficulty in distinguishing emotional adjectives. Results indicated that ToM ability in the RMET is impaired in PD patients, and that this finding was not attributable to the visual processing of faces or the verbal comprehension of emotional adjectives. Thus, the affective aspects of ToM can be impaired in PD patients.

Date 2011

URL https://www.sciencedirect.com/science/article/pii/S1353802010002245
Volume 17
Pages 246-248
Publication Parkinsonism & Related Disorders
DOI https://doi.org/10.1016/j.parkreldis.2010.09.001
Issue 4
ISSN 1353-8020
Date Added 6.7.2025, 19:12:36
Modified 5.9.2025, 14:59:07

Notes:

Included

Sample characteristics

Size: 20 PD, 210 HC, matched for age, gender, education)

PD-type: NA

PD-duration: M = 5.1, SD = 0.7

Medication: Assessment in ON

Hoehn-Yahr: M = 1.5, SD = 0.7

UPDRS-3: NA

Gender (male): 11 (55%)

Age: M = 70.5, SD = 8.6

Other neurological disease (tumor, stroke, etc.): none

Other major psychopathology: None

Origin country (or ethnicity): Japan

method behavioural

instruments used in order to quantify the variables

Social cognition aspect: ToM

Name of the task: Reading the mind in the eyes test (RMET)

Type of stimulus [face/voice etc., Ekman faces/other etc.]: 20 photographs of the eye region of the faces of Japanese actors

Task condition: 4-way forced choice

Operationalization: correct answers

Main findings related to the review's scope

PD patients performed significantly worse than the HC subjects.

In order to investigate if early PD influences performance on the RMET test, we analyzed the data limited to patients with Hoehn & Yahr stage I (N = 13). The result showed the same pattern; PD patients performed worse than NC subjects in the RMET test.

Tags: ToM, behavioral

Is theory-of-mind impaired in Parkinson's disease?

Item Type Journal Article
Author Alexia Mengelberg

Author Richard J. Siegert

Abstract INTRODUCTION: Theory-of-mind (ToM) refers to the ability to understand and predict the behaviour of others based on their mental states. Research from brain imaging and lesion studies indicate that the frontal lobes are both involved and necessary to understand mental states. METHODS: A total of 13 people with Parkinson's disease were compared with 11 age-matched controls on tasks involving ToM, based on the hypothesis that the frontal lobes are affected by Parkinson's disease. The four tasks included both ToM and non-ToM components, such as memory, to investigate the specific nature of the deficit. RESULTS: The group with Parkinson's disease scored significantly lower on three out of four of the ToM components of the tasks, relative to controls, but were not impaired on any of the non-ToM components of the four tasks. CONCLUSION: The results suggest the impairment was specific to ToM and thus have implications for a dedicated specific ToM module.

Date 2003 Aug
Language eng
Extra Place: England
Volume 8
Pages 191-209
Publication Cognitive neuropsychiatry
DOI 10.1080/13546800244000292
Issue 3
Journal Abbr Cogn Neuropsychiatry
ISSN 1354-6805
PMID 16571560
Date Added 6.7.2025, 19:09:41
Modified 6.7.2025, 19:09:41

Notes:

Included

Sample characteristics

Size: 13 PD, 11 HC (elderly)

PD-type: NA

PD-duration: NA

Medication: All patients except one were ON

Hoehn-Yahr: 4 stage 2, 6 stage 3, 2 stage 4

UPDRS-3: Na

Gender (male): 4 (31%)

Age: M = 72.92, SD = 8.98, Range = 50-84

Other neurological disease (tumor, stroke, etc.): NA

Other major psychopathology: NA

Origin country (or ethnicity): New Zealand

method behavioural

instruments used in order to quantify the variables

Social cognition aspect: ToM

Name of the task: Card-sequencing task

Type of stimulus [face/voice etc., Ekman faces/other etc.]: 18 story sequences each depicted on four cards using black-and-white cartoon type drawings.

Task condition: Participants were asked to arrange the cars in a straight line so that they showed a logical sequence of events. could be on false beliefs, social situation, mechanical, or complex reasoning.

Operationalization: 2 points if first card was placed in correct position, 2 if the last card was correct, 1 each for correctly positioning the middle two cards. : Max = 6. Min = 0

Social cognition aspect: ToM

Name of the task: Short-passage task

Type of stimulus [face/voice etc., Ekman faces/other etc.]: 167 short stories and questions

Task condition: Participants had to answer a test question. Could be on ToM or physical (similar topic areas, but did not require the understand of the mental states)

Operationalization: 2 points for a full and explicit answer, 1 for a partial answer, 0 for incorrect answer.

Social cognition aspect: ToM

Name of the task: First order story and questions

Type of stimulus [face/voice etc., Ekman faces/other etc.]: 1 story three paragraphs long

Task condition: Answer questions verbally. False-belief-question; Inference question, fact question, memory question.

Operationalization: 2 points for full answer which correctly made an inference about ehe main character's fals belief, 1 for a partial answer, 0 for an incorrect answer. for the inference, facna dna memory questions participants scored either 1 or 0.

Social cognition aspect: ToM

Name of the task: second order story

Same as first order story but it required understand of "one character thinks another character thinksX", in order to answer the fals belief question.

Main findings related to the review's scope

1. Card sequences: PD sig. worse on false-belief set of cards. No sig. difference for total score and other subscores (social situation, mechanical, complex reasoning)
2. Passages: PD significantly worse in ToM task. Worse but not significant in physical task
3. First order false-belief: PD sig. worse in false-belief question. No difference in total score and other subscores (inference question, fact question, memory question).
4. Second order false-belief story: Overall, PD scored lower, but not significantly

Tags: ToM, behavioral

Ketamine reversed short-term memory impairment and depressive-like behavior in animal model of Parkinson's disease.

Item Type Journal Article

Author Débora Dalla Vecchia
Author Luiz Kae Sales Kanazawa
Author Etiéli Wendler
Author Palloma de Almeida Soares Hocayen
Author Maria Aparecida Barbato Frazão Vital
Author Reinaldo Naoto Takahashi
Author Claudio Da Cunha
Author Edmar Miyoshi
Author Roberto Andreatini

Abstract The most common features of Parkinson's disease (PD) are motor impairments, but many patients also present depression and memory impairment. Ketamine, an N-methyl-d-aspartate (NMDA) receptor antagonist, has been shown to be effective in patients with treatment-resistant major depression. Thus, the present study evaluated the action of ketamine on memory impairment and depressive-like behavior in an animal model of PD. Male Wistar rats received a bilateral infusion of 6 μg/side 6-hydroxydopamine (6-OHDA) into the substantia nigra pars compacta (SNc). Short-term memory was evaluated by the social recognition test, and depressive-like

behaviors were evaluated by the sucrose preference and forced swimming tests (FST). Drug treatments included vehicle (i.p., once a week); ketamine (5, 10 and 15 mg/kg, i.p., once a week); and imipramine (20 mg/kg, i.p., daily). The treatments were administered 21 days after the SNc lesion and lasted for 28 days. The SNc lesion impaired short-term social memory, and all ketamine doses reversed the memory impairment and anhedonia (reduction of sucrose preference) induced by 6-OHDA. In the FST, 6-OHDA increased immobility, and all doses of ketamine and imipramine reversed this effect. The anti-immobility effect of ketamine was associated with an increase in swimming but not in climbing, suggesting a serotonergic effect. Ketamine and imipramine did not reverse the 6-OHDA-induced reduction in tyrosine hydroxylase immunohistochemistry in the SNc. In conclusion, ketamine reversed depressive-like behaviors and short-term memory impairment in rats with SNc bilateral lesions, indicating a promising profile for its use in PD patients.

Date 2021 Mar
Language eng
License Copyright © 2020 Elsevier Inc. All rights reserved.
Extra Place: United States
Volume 168
Pages 63-73
Publication Brain research bulletin
DOI 10.1016/j.brainresbull.2020.12.011
Journal Abbr Brain Res Bull
ISSN 1873-2747 0361-9230
PMID 33359641
Date Added 6.7.2025, 19:09:38
Modified 5.9.2025, 14:59:37

Lifespan reference curves for harmonizing multi-site regional brain white matter metrics from diffusion MRI

Item Type Journal Article
Author Alyssa H. Zhu
Author Talia M. Nir
Author Shayan Javid
Author Julio E. Villalon-Reina
Author Amanda L. Rodrigue
Author Lachlan T. Strike
Author Greig I. de Zubicaray
Author Katie L. McMahon
Author Margaret J. Wright
Author Sarah E. Medland
Author John Blangero
Author David C. Glahn
Author Peter Kochunov
Author Douglas E. Williamson

Author Asta K. Haberg
Author Paul M. Thompson
Author Neda Jahanshad
Date 2025-05-06
Volume 12
Publication SCIENTIFIC DATA
DOI 10.1038/s41597-025-05028-2
Issue 1
Date Added 14.7.2025, 14:48:38
Modified 14.7.2025, 15:08:28

Notes:

Not Included: No PD-group

Tags: EXCLUDED

Locus Coeruleus Integrity from 7 T MRI Relates to Apathy and Cognition in Parkinsonian Disorders

Item Type Journal Article
Author Rong Ye
Author Claire O'Callaghan
Author Catarina Rua
Author Frank H. Hezemans
Author Negin Holland
Author Maura Malpetti
Author P. Simon Jones
Author Roger A. Barker
Author Caroline H. Williams-Gray
Author Trevor W. Robbins
Author Luca Passamonti
Author James Rowe

Abstract Background Neurodegeneration in the locus coeruleus (LC) contributes to neuropsychiatric symptoms in both Parkinson's disease (PD) and progressive supranuclear palsy (PSP). Spatial precision of LC imaging is improved with ultrahigh field 7 T magnetic resonance imaging. Objectives This study aimed to characterize the spatial patterns of LC pathological change in PD and PSP and the transdiagnostic relationship between LC signals and neuropsychiatric symptoms. Methods Twenty-five people with idiopathic PD, 14 people with probable PSP-Richardson's syndrome, and 24 age-matched healthy controls were recruited. Participants underwent clinical assessments and high-resolution (0.08 mm(3)) 7 T-magnetization-transfer imaging to measure LC integrity in vivo. Spatial patterns of LC change were obtained using subregional mean contrast ratios and significant LC clusters; we further correlated the LC contrast with measures of apathy and cognition, using both mixed-effect models and voxelwise analyses. Results PSP and PD groups showed significant LC degeneration in the caudal subregion relative to controls. Mixed-effect models

revealed a significant interaction between disease-group and apathy-related correlations with LC degeneration (beta = 0.46, SE [standard error] = 0.17, F(1, 35) = 7.46, P = 0.01), driven by a strong correlation in PSP (beta = -0.58, SE = 0.21, t(35) = -2.76, P = 0.009). Across both disease groups, voxelwise analyses indicated that lower LC integrity was associated with worse cognition and higher apathy scores. Conclusions The relationship between LC and nonmotor symptoms highlights a role for noradrenergic dysfunction across both PD and PSP, confirming the potential for noradrenergic therapeutic strategies to address transdiagnostic cognitive and behavioral features in neurodegenerative disease. (c) 2022 The Authors. Movement Disorders published by Wiley Periodicals LLC on behalf of International Parkinson and Movement Disorder Society

Date 2022-08
Language English
Extra Place: 111 RIVER ST, HOBOKEN 07030-5774, NJ USA Type: Article
Volume 37
Publisher WILEY
Pages 1663-1672
Publication MOVEMENT DISORDERS
DOI 10.1002/mds.29072
Issue 8
ISSN 0885-3185
Date Added 14.7.2025, 14:50:29
Modified 5.9.2025, 15:01:29

Notes:

Not Included: No SC studied

Tags: EXCLUDED

Longitudinal assessment of social cognition in de novo Parkinson's disease patients and its relationship with dopaminergic innervation

Item Type Journal Article
Author Clara Trompeta
Author Carmen Gasca-Salas
Author José A. Pineda-Pardo
Author Pasqualina Guida
Author Melanie Cohn
Author David Mata-Marín
Author Mariana HG Monje
Author Miguel López-Aguirre
Author Ignacio Obeso
Author Álvaro Sánchez Ferro
Abstract Background Social Cognition (SC) has been scarcely studied in Parkinson's disease (PD), and findings in early disease are controversial. SC encompasses different capacities such as facial emotion recognition (FER); Theory of Mind (ToM), the

ability to understand other people's intentions (cognitive-ToM) and emotions (affective-ToM); and self-monitoring, the ability to regulate one's own behavior in social contexts. A relationship between dopaminergic deficit and SC in PD has been suggested. Objectives To prospectively assess, over a two-year period, SC in newly diagnosed drug-naïve, cognitively normal and non-depressed PD patients. Furthermore, we aimed to evaluate the relationship between SC and Fluorodopa (Positron Emission Tomography) Ki uptake, which is a marker of dopaminergic depletion. Methods We compared SC performance between 25 de novo PD patients and 20 healthy controls (HC), and within-patients at baseline and two-year follow-up. The SC assessment included FER, ToM, as well as self-monitoring measures. The relationship between SC and dopaminergic innervation was also assessed in patients. Results SC scores did not differ between PD and HC groups at baseline, nor between baseline and follow-up evaluation in PD. A significant positive correlation between self-monitoring and Fluorodopa Ki uptake in the left pallidum in PD patients was found at baseline. At follow-up, ToM (stories) positively correlated with Fluorodopa Ki uptake in the right thalamus and the left putamen. Conclusion SC appears to be preserved in de novo PD and remains stable in the short-term. Although more evidence is needed, our results support a relationship between dopamine innervation in subcortical regions and SC.

Date 2023
URL https://www.sciencedirect.com/science/article/pii/S0166432823003728
Volume 454
Pages 114654
Publication Behavioural Brain Research
DOI https://doi.org/10.1016/j.bbr.2023.114654
ISSN 0166-4328
Date Added 6.7.2025, 19:12:35
Modified 5.9.2025, 14:58:58

Notes:

Included

Sample characteristics

Size: 25 PD (de novo), 20 HC (age, educatin, gender matched)

within patients at baseline and 2-year follow-up

PD-type: NA

PD-duration: M = 1, Sd = 0.55

Medication: OFF-state

Hoehn-Yahr: NA

UPDRS-3: M = 19.52, SD = 10.58

Gender (male): 15 (60%)

Age: M = 56.04, SD = 9.76

Other neurological disease (tumor, stroke, etc.): NA

Other major psychopathology: none

Origin country (or ethnicity): Spain

method (Review, meta-analysis or observational and/or self-reported):

instruments used in order to quantify the variables

Social cognition aspect: emotion recognition

Name of the task: NA

Type of stimulus [face/voice etc., Ekman faces/other etc.]: sixty-four photographs from the Karolinska Directed Emotional Faces (KDEF)

Task condition: included eight for each emotion (anger, disgust, fear, happiness, sadness, surprise) and 16 for neutral faces (no expression)

Operationalization: Participants selected one of the six emotions or a neutral face for each photograph with no time limit>>Correct answers

---

Social cognition aspect: Cognitive ToM

Name of the task: Theory of Mind Picture Stories Task (ToM stories)

Type of stimulus [face/voice etc., Ekman faces/other etc.]: Six picture stories were presented, each containing four cards depicting a scene

Task condition: The participant was required to order the cards in the correct chronological sequence. They were then asked to answer a total 23 questions addressing their ability to detect cheating, cooperation, and deception, and to understand false and true beliefs attributed to the characters (1 point each)

Operationalization: Up to six points were assigned for each correct sequence (maximum sum score of 36. The maximum score was 59 points (ToM sequencing 36 and ToM questionnaire 23)

Social cognition aspect: AffectiveToM

Name of the task: Spanish adaptation of the Reading the Mind in The Eyes Test (RMET)

Type of stimulus [face/voice etc., Ekman faces/other etc.]: 36 photographs of eye-region

Task condition: Participants were asked to select one of four words that they consider best describes the mental state of 36 based on photographs of eye regions

Operationalization: One point was given for each correct answer, and the maximum possible score was 36.

Social cognition aspect: Self-Monitoring (how well the patients is able to change behavior in order to adapt social situations

Name of the task: Self-Monitoring Scale (RSMS)

Type of stimulus [face/voice etc., Ekman faces/other etc.]: a 13-item questionnaire completed by the informant.

Task condition: The answers are on a 6-point Likert-scale and are also distributed from "very bad"(0) to "very good" (5)

Operationalization: The total score is 65 points, and a higher the score reflects better ability to adapt to a social context.

PET:

Scans were acquired at rest, with at least 6 h of fasting, on a hybrid 3 T mMRBiograph system (Siemens Healthcare, Erlangen, Germany). he acquisition started immediately following an intravenous injection of 5 mCi of 6-[18 F]-fluoro-L-dopa (Fluorodopa). The night before and one hour before the PET exam, [redacted] (200 mg) was administered orally. PET data were acquired in list-mode for 90 min. Scans were corrected for attenuation using a 4-compartment MR-based map derived from a dual-echo Dixon-based sequence (TR: 3.6 ms; TE: [1.23,2.46] ms) [66]. Twenty-two timeframes of PET activity were reconstructed: 10 frames of 90 s, 9 frames of 300 s, and 3 frames of 600 s. The effective resolution of these images was 2.09 × 2.09 × 2.03 mm^3.The Patlak graphical method was used in order to estimate voxel-based Fluorodopa [redacted] (Ki), taking the average time activity curve from an [redacted] mask as reference. The Ki was then averaged in the [redacted] innervated regions of interest (ROIs) according to the most sensitive subcortical dopaminergic innervated areas (caudate, putamen, globus pallidus, thalamus) on both sides separately. ROIs were obtained using the FMRIB's integrated registration and segmentation tool (FIRST-FSL) for subcortical segmentation. Of note, cortical areas known to be involved in SC in other populations, such as the [redacted] and the orbitofrontal cortex, were not included in our analyses given the low sensitivity of Fluorodopa imaging across these areas.

<u>Main findings related to the review's scope</u>

Assessment at baseline

The scores on FER, ToM tests and the RSMS (informant) scale did not differ significantly between the two groups.

Correlation between social cognition and pet imaging - only on PD

At baseline a significant positive correlation between RSMS and Fluorodopa Ki uptake in left pallidum (rs = 0.509; P = 0.026) was observed in PD patients. No other sig. correclations.

At follow-up we found a significant positive correlation between the 1) total score from the ToM stories and Fluorodopa Ki uptake in the right thalamus (rs =0.647; P = 0.017) and the left putamen (rs =0.563; p = 0.045), and 2) the sequencing score from the ToM stories and Fluorodopa Ki uptake in the right thalamus (rs =0.554; P = 0.050) in PD patients

Tags: Emotion recognition, ToM, Imaging, social decision-making, behavioral, Questionnaire

---

Management of Motor and Non-Motor Symptoms in Parkinson's Disease

**Item Type**	Journal Article
**Author**	Fabienne Sprenger
**Author**	Werner Poewe
**Date**	4/2013
**Language**	en
**Library Catalog**	DOI.org (Crossref)
**URL**	http://link.springer.com/10.1007/s40263-013-0053-2
**Accessed**	18.1.2026, 22:54:31
**License**	http://www.springer.com/tdm
**Volume**	27
**Pages**	259-272
**Publication**	CNS Drugs
**DOI**	10.1007/s40263-013-0053-2
**Issue**	4
**Journal Abbr**	CNS Drugs
**ISSN**	1172-7047, 1179-1934
**Date Added**	18.1.2026, 22:54:31
**Modified**	18.1.2026, 22:54:31

Measuring emotion recognition by people with Parkinson's disease using eye-tracking with dynamic facial expressions

**Item Type**	Journal Article
**Author**	Judith Bek
**Author**	Ellen Poliakoff
**Author**	Karen Lander
**Abstract**	Background Motion is an important cue to emotion recognition, and it has been suggested that we recognize emotions via internal simulation of others' expressions. There is a reduction of facial expression in Parkinson's disease (PD), which may influence the ability to use motion to recognise emotions in others. However, the majority of previous work in PD has used only static expressions. Moreover, few studies have used eye-tracking to explore emotion processing in PD. New method We measured accuracy and eye movements in people with PD and healthy controls when

identifying emotions from both static and dynamic facial expressions. Results The groups did not differ overall in emotion recognition accuracy, but motion significantly increased recognition only in the control group. Participants made fewer and longer fixations when viewing dynamic expressions, and interest area analysis revealed increased gaze to the mouth region and decreased gaze to the eyes for dynamic stimuli, although the latter was specific to the control group. Comparison with existing methods Ours is the first study to directly compare recognition of static and dynamic emotional expressions in PD using eye-tracking, revealing subtle differences between groups that may otherwise be undetected. Conclusions It is feasible and informative to use eye-tracking with dynamic expressions to investigate emotion recognition in PD. Our findings suggest that people with PD may differ from healthy older adults in how they utilise motion during facial emotion recognition. Nonetheless, gaze patterns indicate some effects of motion on emotional processing, highlighting the need for further investigation in this area.

Date 2020
URL https://www.sciencedirect.com/science/article/pii/S0165027019303814
Volume 331
Pages 108524
Publication Journal of Neuroscience Methods
DOI https://doi.org/10.1016/j.jneumeth.2019.108524
ISSN 0165-0270
Date Added 6.7.2025, 19:12:36
Modified 5.9.2025, 14:28:11

Notes:

Included

sample characteristics

size: 18 PD and 10 HC (matched in sex)

Parkinson's Disease type and duration: idiopathic PD, Mduration= 5.9 years (SD=5.3)

Medication: on medication

Hoehn-Yahr: range=1-3

UPDRS-3: M= 40.1 (SD=13.2)

Gender (male): 9 males (50%)

averaged ages (SD, range): M= 63.2 years (SD=5.5)

other neurological disease (tumor, stroke, etc.): NA

other major psychopathology: NA

origin country (or ethnicity): United Kingdom

method observational

instruments used in order to quantify the variables

Social cognition aspect: emotion recognition

Name of the task: the Amsterdam Dynamic Facial Expression Set

type of stimulus [face/voice etc., Ekman faces/other etc.]: videos of male and female models of different ethnicities (Mediterranean and Northern European). Dynamic expressions are depicted in videos in which the face begins in a neutral expression and culminates in the peak intensity of the emotion.

Static versions of each stimulus consist of a still image of the face at the apex of the expression.

task condition: joy, sadness, anger, fear, surprise and disgust), as well as neutral

operationalization: Accuracy and eye-movement

Eye movements were recorded using an Eyelink 1000+ eye tracker (SR Research Ltd.) with remote monocular pupil capture at a sampling rate of 500 Hz. Participants were seated at a distance of 700 mm from a 530 mm x 300 mm monitor in a dimly-lit room.

Main findings related to the review's scope

The groups did not differ overall in emotion recognition accuracy, but motion significantly increased recognition only in the control group.

PD mainly focused on the nose-bridge area, both in static and dynamic pictures. While HC scanned the

faces from the nose-bridge to the mouth region equally, both in static and dynamic pictures.
Tags: Emotion recognition, behavioral

Mental State Recognition Deficits Linked to Brain Changes in Parkinson's Disease Without Dementia.

**Item Type**	Journal Article
**Author**	Giulia Funghi
**Author**	Giuseppe Rabini
**Author**	Claudia Meli
**Author**	Chiara Speranza
**Author**	Enrica Pierotti
**Author**	Francesca Saviola
**Author**	Stefano Tambalo
**Author**	Francesca Zappini
**Author**	Giorgio Fumagalli
**Author**	Luca Turella
**Author**	Jorge Jovicich
**Author**	Costanza Papagno
**Author**	Alessandra Dodich
**Abstract**	Recent studies have reported social cognitive deficits, particularly in emotional processing, in Parkinson's disease (PD). However, a comprehensive characterization of these deficits and their underlying neural correlates remains elusive. Therefore, this study aims to investigate the association between deficits in the recognition of complex mental states and structural/functional brain changes in non-demented PD individuals. To reach this aim, 24 PD participants underwent clinical assessment, neuropsychological testing and the FAcial Complex Expressions (FACE) test, a novel test of complex mental state recognition from faces. Patients were classified as clinically impaired (n = 8) or unimpaired (n = 16) based on performance on this test. Magnetic resonance imaging data were acquired to investigate the association between FACE test performance and both resting-state functional connectivity and grey matter volume, within the emotion understanding network and at the whole-brain level. Statistical analyses also included the comparison of imaging metrics between the impaired and unimpaired groups. Results showed that complex mental state recognition in PD was significantly associated with both defective and compensatory mechanisms at the functional and anatomical level within the emotion understanding network, particularly involving the amygdala, dorsomedial prefrontal cortex, primary/secondary somatosensory cortices, and right anterior temporal cortex. Whole-brain results extended the network to temporal and medial frontal areas. In conclusion, reduced recognition of complex mental states in non-demented PD patients is associated with alterations in the emotion understanding network A comprehensive characterization of early emotional deficits in these patients may have significant implications in the characterization of the cognitive phenotype, with potential benefit for tailored non-pharmacological intervention.
**Date**	2025 Feb
**Language**	eng

License © 2025 The Author(s). European Journal of Neuroscience published by Federation of European Neuroscience Societies and John Wiley & Sons Ltd.
Extra Place: France
Volume 61
Pages e70014
Publication The European journal of neuroscience
DOI 10.1111/ejn.70014
Issue 4
Journal Abbr Eur J Neurosci
ISSN 1460-9568 0953-816X
PMID 39957381
PMCID PMC11831241
Date Added 6.7.2025, 19:09:38
Modified 5.9.2025, 14:36:03

Notes:

Included

sample characteristics

size: 24 PD

Parkinson's Disease type and duration: idiopathic PD

Medication: on medication

Hoehn-Yahr: M=1.77 SD=0.67 max=3

UPDRS-3: M=19 SD=8.6

Gender (male): 13 males (54%)

averaged ages (SD, range): M= 67.1 SD= 7.0 min=50

other neurological disease (tumor, stroke, etc.): No dementia

other major psychopathology: None

origin country (or ethnicity): Canada

method observational and imaging

instruments used in order to quantify the variables

Social cognition aspect:

Name of the task: the Facial Complex Expressions (FACE) test

type of stimulus [face/voice etc., Ekman faces/other etc.]: Coloured facial expressions representing different mental states and interpreted by professional actors.

operationalization: required to provide a verbal response by selecting from four adjectives the one that most accurately described the actor's facial expression.

MRI: 3.0 T MRI scanner (Prisma, Siemens) with a 64-channel head receive coil.
MPRAGE_GRAPPA: 176 volumes, isotropic voxel resolution of 1 mm, sagittal plane orientation, flip angle of 7°, matrix = 256 × 256, repetition time (TR) of 2.53 s, echo time (TE) of 1.35, 3.07, 4.79, 6.51 ms, TI of 1100 ms, slice thickness of 1 mm. Additionally, resting-state functional magnetic resonance images (rs-fMRI) were acquired using echo planar imaging (EPI) T2*-weighted scans. The following acquisition parameters were used: TE of 28 ms, TR of 1.0 s, flip angle of 59°, axial slice thickness of 2 mm. A total of 400 whole-brain volumes were acquired in a resting-state run of 6 min 40 s, isotropic voxel size 2 mm, AC/PC aligned.

Functional connectivity: rs-fMRI analyses were performed using CONN functional connectivity toolbox and SPM12. using a flexible pre-processing pipeline, including (a) realignment with correction of susceptibility- distortion interactions, (b) slice timing correction, (c) outlier detection, (d) direct segmentation and normalization to the Montreal Neurological Institute(MNI) space and (e) smoothing (only for voxel-level analyses, not for ROI-level analyses). Functional and anatomical data were normalized into standard MNI space ,segmented into grey matter (GM), white matter (WM) and

cerebrospinal fluid (CSF) tissue classes, and resampled to 2-mmisotropic voxels following a direct normalization procedure using SPM unified segmentation and normalization algorithm with the default IXI-549 tissue probability map template. Last, functional data (only for voxel-level analyses) were smoothed using spatial convolution with a Gaussian kernel of 8-mm full width half maximum (FWHM).

Gray-matter: Anatomical images were analysed using the Computational Anatomy Toolbox (CAT12.8.2-r2170) and SPM12. The T1-weighted anatomical images were pre-processed according to the standard VBM pre-processing pipeline of CAT12, which includes: (a) tissue segmentation into GM, WM, and CSF, (b) spatial normalization to the MNI space, (c) modulation, and (d) spatial smoothing with a Gaussian kernel of 8-mm full width at half maximum (FWHM) (Gaser et al., 2022). In addition, to assess data quality, we visually inspected each segmented and modulated image and considered the Image Quality Ratings (IQR) index provided in the CAT12 reports. The IQR index is calculated based on measures of noise, bias, and image resolution, so we checked it to further ensure image quality. In this study, all images were above the satisfactory level of IQR (IQR index > 70%), which is consistent with the description of quality ratings in the CAT12 guideline.

OFFERED MODEL:

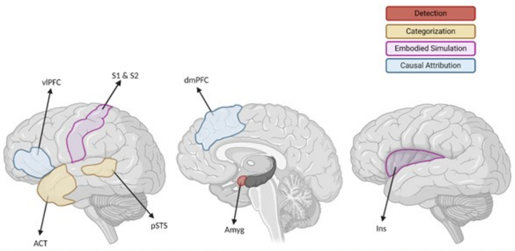

FIGURE 2 | Brain regions associated with the different functional aspects of emotion understanding (adapted from Spunt and Adolphs 2019). Image created with BioRender.com (https://www.biorender.com, agreement number: QC26XF9BAG).

Main findings related to the review's scope

PD patients were classified as impaired (PD-IMP) or unim-paired (PD-UN) based on their performance on the FACE test according to Italian normative data.

Individuals with an adjusted FACE score <24.612 (Terruzzi et al. 2023) were included in the PD-IMP (N=8) group, while the others were in the PD-UN group (N=16).

functional connectivity (FC): PD patients impaired on the FACE test had lower FC than unimpaired PD patients between left and right amygdala, left amygdala and left anterior temporal cortex, and right amygdala and left anterior temporal cortex.

No significant regions were identified with higher functional connectivity (FC).

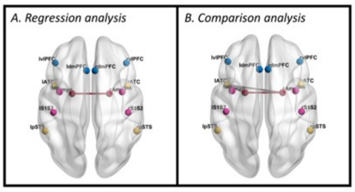

FIGURE 3 | ROI-based FC results—(A) Significant positive association between ROI-to-ROI FC metrics and FACE test score in PD patients. (B) Difference in average connectivity between PD-UN and PD-IMP groups. Images created with BrainNet Viewer (http://www.nitrc.org/projects/bnv/) (Xia, Wang, and He 2013).

At the structural level, ROI-based multiple regression analysis across the entire PD sample on 14 ROIs involved in emotion understanding showed a significant effect of FACE test score in bilateral dorsomedial prefrontal cortex, bilateral primary and secondary somatosensory cortex, bilateral amygdala, and right anterior temporal cortex (see Figure 4 and Table 2).

Exploratory: using the left and right amygdala separately as seeds revealed significant positive correlations between the FACE test performance and FC between the left amygdala and both the right temporal pole/right amygdala (x = 30, y = 10, z = −30;p-FDR = 0.0258) and the left insular cortex (x = −40, y = 14,z = −12; p-FDR = 0.0258), as well as between the right–left amygdala (x = −18; y = −06; z = −18; p-FDR = 0.0151) across the entire PD sample

On the other hand, results of comparative SBC analyses between PD-UN and PD-IMP showed a difference in mean FC between bilateral amygdala and bilateral temporal cluster (including inferior and middle temporal gyrus,temporal pole), as well as between right amygdala and bilateralpre/post-central gyrus, with PD-IMP group showing reducedFC (Figure 5B and Table 3). No increased FC was found in thePD-IMP compared to the PD-UN subgroup.

VBM: the entire PD sample on 14 ROIs involved in emotion understanding showed a significant effect of FACE test score in bilateral dorsomedial prefrontal cortex, bilateral primary and secondary somatosensory cortex, bilateral amygdala, and rightanterior temporal cortex

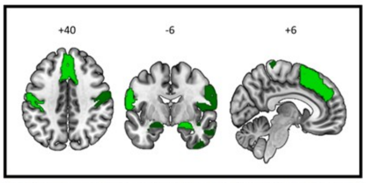

FIGURE 4 | ROI-based VBM results—Significant positive association between GM volume and FACE test score in PD patients. Colour scale indicates t statistic: light green, t value >3; dark green, t value >2. Significance: $p < 0.05$ FDR corrected. Images displayed in neurological convention.

A significant difference also emerged between the two groups (PD-IMP vs PD-UN) only in the right amygdala (p value uncorrected = 0.0057, tvalue = 2.82), where the PD-UN group had higher GM volumethan the PD-IMP

Exploratory: sig cluster spanning the dorsomedial and the ventromedial prefrontal cor-tex (peak MNI coordinates: x = 0; y = 45; z = 15; k = 4109; $p < 0.05$FDR corrected at cluster level; t value = 6.72) across the entirePD sample. When comparing PD-IMP and PD-UN, no resultssurvived FDR correction. Using an uncorrected threshold atvoxel-level ($p < 0.001$), the differences between impaired and un-impaired PD patients were located in the dorsomedial prefrontalcortex (paracingulate and cingulate gyrus, x = 0, y = 45, z = 14; p-unc < 0.001, t value = 5.83; middle frontal gyrus, x = −36, y = 11,z = 36, p-unc = 0.001, t value = 3.65), left lateral occipital cortex(x = −32, y = −86, z = 44; p-unc < 0.001, t value = 4.38) and righttemporal pole/amygdala (x = 27, y = 5, z = −29; p-unc = 0.001, tvalue = 3.70)

TABLE 3 | Whole-brain FC results—SBC analysis of left/right amygdala comparing PD-UN and PD-IMP.

Probabilistic anatomical label	Cluster (*x,y,z*)	Uncorrected *p*	FDR-corrected *p*	*t* value
Seed—Left Amygdala				
L Temporal Pole	−40,+14,−12	0.000002	0.000077	9.81
R Hippocampus/Right Amygdala	+24,−26,−12	0.000088	0.001409	7.61
R Inferior Temporal Gyrus, posterior division	+50,−8,−28	0.000278	0.002433	6.22
L Middle Temporal Gyrus, posterior division	−64,−28,−8	0.000359	0.002433	5.89
R Temporal Pole	+28,−6,−30	0.000380	0.002433	5.78
L Inferior Temporal Gyrus, anterior division	−46,−8,−30	0.001479	0.007888	5.77
L Supramarginal Gyrus, posterior division	−42,−48,+10	0.004734	0.021641	5.54
L Temporal Pole	−40,+2,−44	0.005647	0.022589	5.12
Seed—Right Amygdala				
L Amygdala	−18,−6,−20	0.000001	0.000036	9.04
L/R Precentral Gyrus	+6,−22,+ 54	0.000004	0.000104	6.78
L Precentral and Postcentral Gyrus	−40,−18,+42	0.000007	0.000135	6.37
R Postcentral Gyrus	+66,−10,+14	0.001917	0.027314	5.80
R Inferior Temporal Gyrus, posterior division	+52,−8,−28	0.002688	0.030648	5.04

Note: Coordinates for each cluster that exceeds the thresholds specified in the text, showing a higher FC in the PD-UN versus PD-IMP group.

TABLE 2 | ROI-based VBM results—Significant positive association with GM volume in PD patients.

Regions of interest	Uncorrected *p*	FDR-corrected *p*	*t* value
L dorsomedial prefrontal cortex	0.0025	0.0146	3.16
L ventrolateral prefrontal cortex	0.0326	—	1.95
L primary & secondary somatosensory cortex	0.0042	0.0146	2.93
L amygdala	0.0189	0.0440	2.23
L anterior temporal cortex	0.0336	—	1.94
R dorsomedial prefrontal cortex	0.0034	0.0146	3.01
R primary and secondary somatosensory cortex	0.0138	0.0386	2.38
R amygdala	0.0017	0.0146	3.33
R anterior temporal cortex	0.0242	0.0484	2.10

Note: Both FDR-corrected and uncorrected results are displayed.

Tags: Emotion recognition, Imaging

MENTALIZING DEFICIT IN NEUROLOGICAL DISORDERS: A REVIEW ARTICLE

Item Type Journal Article
Author Herold Robert
Author Varga Eszter
Author Mike Andrea
Author Tenyi Tamas
Author Simon Maria
Author Hajnal Andras
Author Fekete Sandor
Author Illes Zsolt
Abstract Introduction - Mentalization is the ability to attribute mental states (intentions, desires, thoughts, emotions) to others, and hence to predict their behaviour. This ability fundamentally determines our participation in social relationships and

adaptation to society. A significant proportion of the disorders of the central nervous system (CNS) affects those brain structures and neurotransmitter systems that play a role in the mentalizing processes. Accordingly, a number of CNS disorders may be associated with mentalizing deficits, which may affect the outcome of these diseases. Here, we review recent research on mentalizing abilities in neurological diseases. Methods - An internet database search was performed to identify publications on the subject. Results - Sixty-two publications in English corresponded to the search criteria. These publications reported impaired mentalization in several neurological disorders (e.g. epilepsy, Parkinson's disease, multiple sclerosis, dementias, traumatic brain injury). Discussion - The results indicate that a number of neurological disorders associate with mentalizing deficit. This deficit is often present in the early stages of the diseases and has a prognostic value, which in turn emphasizes the importance of the early detection and adequate rehabilitation.

Date 2015-11-30
Language Hungarian
Extra Place: MARGIT KRT 31-33, BUDAPEST, 1024, HUNGARY Type: Review
Volume 68
Publisher LITERATURA MEDICA
Pages 364-373
Publication IDEGGYOGYASZATI SZEMLE-CLINICAL NEUROSCIENCE
DOI 10.18071/isz.isz.68.364
Issue 11-12
ISSN 0019-1442
Date Added 14.7.2025, 14:50:36
Modified 5.9.2025, 14:53:10

Notes:

Not Included: not is English

Tags: EXCLUDED

Metacognition of emotion recognition across neurodegenerative diseases

Item Type Journal Article
Author Indira Garcia-Cordero
Author Joaquín Migeot
Author Sol Fittipaldi
Author Alexia Aquino
Author Cecilia Gonzalez Campo
Author Adolfo García
Author Agustín Ibáñez
Abstract Metacognition (monitoring) of emotion recognition is fundamental for social interactions. Correct recognition of and confidence in the emotional meaning inferred from others' faces are fundamental for guiding and adjusting interpersonal behavior. Yet, although emotion recognition impairments are well documented across neurodegenerative diseases, the role of metacognition in this domain remains poorly understood. Here, we evaluate multimodal neurocognitive markers of metacognition

in 83 subjects, encompassing patients with behavioral variant frontotemporal dementia [bvFTD, n = 18], Alzheimer's disease [AD, n = 27], and demographically-matched controls (n = 38). Participants performed a classical facial emotion recognition task and, after each trial, they rated their confidence in their performance. We examined two measures of metacognition: (i) calibration: how well confidence tracks accuracy; and (ii) a metacognitive index (MI) capturing the magnitude of the difference between confidence and accuracy. Then, whole-brain grey matter volume and fMRI-derived resting-state functional connectivity were analyzed to track associations with metacognition. Results showed that metacognition deficits were linked to basic emotion recognition. Metacognition of negative emotions was compromised in patients, especially disgust in bvFTD as well as sadness in AD. Metacognition impairments were associated with reduced volume of fronto-temporo-insular and subcortical areas in bvFTD and fronto-parietal regions in AD. Metacognition deficits were associated with disconnection of large-scale fronto-posterior networks for both groups. This study reveals a link between emotion recognition and metacognition in neurodegenerative diseases. The characterization of metacognitive impairments in bvFTD and AD would be relevant for understanding patients' daily life changes in social behavior.

Date 2021
URL https://www.sciencedirect.com/science/article/pii/S0010945221000241
Volume 137
Pages 93-107
Publication Cortex
DOI https://doi.org/10.1016/j.cortex.2020.12.023
ISSN 0010-9452
Date Added 6.7.2025, 19:12:35
Modified 5.9.2025, 14:36:08

Notes:

Not Included: not on PD

Tags: EXCLUDED

Mirror neurons and their relationship with neurodegenerative disorders.

Item Type Journal Article

Author Elisabetta Farina
Author Francesca Borgnis
Author Thierry Pozzo

Abstract The finding of mirror neurons (MNs) has provided a biological substrate to a new concept of cognition, relating data on actions and perceptions not only to integrate perception in action planning and execution but also as a neural mechanism supporting a wide range of cognitive functions. Here we first summarize data on MN localization and role in primates, then we report findings in normal human subjects: functional magnetic resonance imaging and neurophysiological studies sustain that MNs have a role in motor learning and recognizing actions and intentions of others, and they also support an embodied view of language, empathy, and memory. Then, we detail the results of literature searching on MNs and embodied cognition in

Parkinson's disease (PD), frontotemporal dementia (FTD)/amyotrophic lateral sclerosis (ALS), and in mild cognitive impairment (MCI)/Alzheimer's disease (AD). In PD the network of MN could be altered, but its hyperactivation might support motor and cognitive performances at least in early stages. In the ALS/FTD continuum, preliminary evidence points out to an involvement of the MN network, which could explain language and inter-subjectivity deficits shown in patients affected by these clinical entities. In the MCI/AD spectrum, a few recent studies suggest a possible progressive involvement from posterior to anterior areas of the MN network, with the brain putting in place compensatory mechanisms in early stages. Reinterpreting neurodegenerative diseases at the light of the new views about brain organization stemming from the discovery of MN could help to better comprehend clinical manifestations and open new pathways to rehabilitation.

Date 2020 Jun
Language eng
License © 2020 Wiley Periodicals, Inc.
Extra Place: United States
Volume 98
Pages 1070-1094
Publication Journal of neuroscience research
DOI 10.1002/jnr.24579
Issue 6
Journal Abbr J Neurosci Res
ISSN 1097-4547 0360-4012
PMID 31975553
Date Added 6.7.2025, 19:09:42
Modified 5.9.2025, 14:35:32

Notes:

Not Included: not an empirical paper

Tags: EXCLUDED

Misperceptions and Parkinson's disease

Item Type Journal Article

Author Joseph H. Friedman

Abstract Most of the neurobehavioral aspects of Parkinson's disease have been well established and studied, but many are not well known, and hardly studied. This article focuses on several behavioral abnormalities that are common, and frequently cause difficulty for the patient and family due to lack of recognition as part of the disease. While it is well known that L-Dopa dyskinesias are frequently not recognized or under appreciated by patients, a similar lack of recognition may affect the patient's own speech volume, where their center of gravity is located, whether they are tilted to one side, and their under-recognition of others' emotional displays. In addition, PD patients are often misperceived by others incorrect impression of their emotional and cognitive state based purely on facial expression. These changes and others are briefly reviewed. (C) 2017 Elsevier B.V. All tights reserved.

Date 2017-03-15
Language English
Extra Place: RADARWEG 29, 1043 NX AMSTERDAM, NETHERLANDS Type: Review
Volume 374
Publisher ELSEVIER
Pages 42-46
Publication JOURNAL OF THE NEUROLOGICAL SCIENCES
DOI 10.1016/j.jns.2016.12.059
Issue SI
ISSN 0022-510X
Date Added 14.7.2025, 14:50:35
Modified 5.9.2025, 14:35:56

Notes:

Not Included: not a systematic review

however, might be relevant to read
Tags: EXCLUDED

Mixed emotions: the contribution of alexithymia to the emotional symptoms of autism

Item Type Journal Article
Author G. Bird
Author R. Cook
Abstract It is widely accepted that autism is associated with disordered emotion processing and, in particular, with deficits of emotional reciprocity such as impaired emotion recognition and reduced empathy. However, a close examination of the literature reveals wide heterogeneity within the autistic population with respect to emotional competence. Here we argue that, where observed, emotional impairments are due to alexithymia-a condition that frequently co-occurs with autism-rather than a feature of autism per se. Alexithmia is a condition characterized by a reduced ability to identify and describe one's own emotion, but which results in reduced empathy and an impaired ability to recognize the emotions of others. We briefly review studies of emotion processing in alexithymia, and in autism, before describing a recent series of studies directly testing this `alexithymia hypothesis'. If found to be correct, the alexithymia hypothesis has wide-reaching implications for the study of autism, and how we might best support subgroups of autistic individuals with, and without, accompanying alexithymia. Finally, we note the presence of elevated rates of alexithymia, and inconsistent reports of emotional impairments, in eating disorders, schizophrenia, substance abuse, Parkinson's Disease, multiple sclerosis and anxiety disorders. We speculate that examining the contribution of alexithymia to the emotional symptoms of these disorders may bear fruit in the same way that it is starting to do in autism.
Date 2013-07
Language English

Extra Place: MACMILLAN BUILDING, 4 CRINAN ST, LONDON N1 9XW, ENGLAND
Type: Review
Volume 3
Publisher NATURE PUBLISHING GROUP
Publication TRANSLATIONAL PSYCHIATRY
DOI 10.1038/tp.2013.61
ISSN 2158-3188
Date Added 14.7.2025, 14:50:38
Modified 14.7.2025, 14:50:38

Notes:

Not Included: not on PD
Tags: EXCLUDED

Moral decision-making and theory of mind in patients with idiopathic Parkinson's disease.

Item Type Journal Article
Author Jan B. Rosen
Author Matthias Brand
Author Christin Polzer
Author Georg Ebersbach
Author Elke Kalbe
Abstract OBJECTIVE: Cognitive impairments in theory of mind (ToM), executive processing, and decision-making are frequent and highly relevant symptoms in patients with Parkinson's disease (PD). These functions have been related to moral decision-making. Their association to moral decision-making in PD, however, has not been studied yet. It was hypothesized that moral decisions in patients with PD differ from those in healthy control participants, and that more egoistic decisions are related to ToM as well as executive dysfunctions in patients with PD. METHOD: Nineteen patients with PD and 20 healthy control participants were examined with an everyday moral decision-making task, comprised of 10 low and 10 high emotional forced-choice moral dilemma short stories with egoistic and altruistic options. All participants received an elaborate neuropsychological test battery. Electrodermal skin conductance responses were recorded to examine possible unconscious emotional reactions during moral decision-making. RESULTS: The groups performed comparably in total scores of moral decision-making. Although ToM did not differ between groups, it was inversely related to altruistic moral decisions in the healthy control group, but not in patients with PD. Executive functions were not related to moral decision-making. No differences were found for skin conductance responses, yet they differed from zero in both groups. CONCLUSION: Our findings indicate that moral decisions do not differ between patients with PD and healthy control participants. However, different underlying processes in both groups can be presumed. While healthy control participants seem to apply ToM to permit egoistic moral decisions in low emotional dilemmas, patients with PD seem to decide independently from ToM. These mechanisms as well as neuropsychological and neurophysiological correlates are discussed.

**Date**	2013 Sep
**Language**	eng
**Extra**	Place: United States
**Volume**	27
**Pages**	562-572
**Publication**	Neuropsychology
**DOI**	10.1037/a0033595
**Issue**	5
**Journal Abbr**	Neuropsychology
**ISSN**	1931-1559 0894-4105
**PMID**	24040930
**Date Added**	6.7.2025, 19:09:40
**Modified**	5.9.2025, 14:53:51

Notes:

Included

Sample characteristics

Size: 19 PD, 20 HC (compatible with respect to age, sex, education)

PD-type: Idiopathic PD

PD-duration: M = 5.79, Sd = 2.20

Medication: Tested in ON

Hoehn-Yahr: Median = 2.5, Range = 1-4

UPDRS-3: NA

Gender (male): 7 (37%)

Age: M = 65.16, Sd = 7.75

Other neurological disease (tumor, stroke, etc.): none

Other major psychopathology: none

Origin country (or ethnicity): Germany

method behavioural

instruments used in order to quantify the variables

Social cognition aspect: Moral Decision-Making

Name of the task: everyday moral decision-making task (EMDM)

Type of stimulus [face/voice etc., Ekman faces/other etc.]: 20 short stories describing everyday moral dilemma situations

Task condition: Every short story was presented on one screen, with a subsequent forcedchoice "yes" or "no" question on whether the subject would choose a proposed behavior, if he or she were confronted with the described situation.

Subdivided into high- and low emotional stories.

On the proposed behaviors in forced-choice questions following the stories, half (n 10) were designed to be "egoistic" while the other half (n 10) were to be "altruistic"

Operationalization: For high emotional, low emotional, and the whole set of stories, the percentage of altruistic decisions was calculated as the moral decision-making scoreAltered moral decision-making of patients with PD in terms of the outcome variable altruistic decisions was a priori assumed, but not found in the current study. Although the HC group made altruistic decisions more often than patients with PD on a descriptive level and effect sizes indicated medium effects for overall moral decision-making and decisions in high emotional moral dilemmas, differences were not significant

---

SC aspect: Affective ToM

Name of Task: REMT

(not further described, so i assume the normal 36 pics and 4-way forced choice)

Main findings related to the review's scope

No sig. diff in REMT (p = 0.253, d = 0.37); (HC slightly better)

Altered moral decision-making of patients with PD in terms of the outcome variable altruistic decisions was a priori assumed, but not found in the current study. Although the HC group made altruistic decisions more often than patients with PD on a descriptive level and effect sizes indicated medium effects for overall moral decision-making and decisions in high emotional moral dilemmas, differences were not significant.

Additionally, decision-making in high emotional moral dilemmas did not differ from decision-making in low emotional moral dilemmas in the PD patient group, t(18) 0.31, p .762, d 0.10, or in the HC group, t(19) 0.99, p .336, d 0.27.

Tags: ToM, social decision-making, behavioral

Motor symptom asymmetry in Parkinson's disease predicts emotional outcome following subthalamic nucleus deep brain stimulation.

**Item Type**	Journal Article
**Author**	Philippe Voruz
**Author**	Florence Le Jeune
**Author**	Claire Haegelen
**Author**	Karim N'Diaye
**Author**	Jean-François Houvenaghel
**Author**	Paul Sauleau
**Author**	Sophie Drapier
**Author**	Dominique Drapier
**Author**	Didier Grandjean
**Author**	Marc Vérin
**Author**	Julie Péron
**Abstract**	The objective of this study was to explore the brain modifications associated with vocal emotion (i.e., emotional prosody) processing deficits in patients with Parkinson's disease after deep brain stimulation of the subthalamic nucleus, and the impact of motor asymmetry on these deficits. We therefore conducted 18-fluorodeoxyglucose positron emission tomography scans of 29 patients with left- or right-sided motor symptoms of Parkinson's disease before and after surgery, and correlated changes in their glucose metabolism with modified performances on the recognition of emotional prosody. Results were also compared with those of a matched healthy control group. Patients with more left-sided motor symptoms exhibited a deficit in vocal emotion recognition for neutral, anger, happiness and sadness in the preoperative condition that was normalized postoperatively. Patients with more right-sided motor symptoms performed comparably to controls in the preoperative condition, but differed significantly on fear postoperatively. At the metabolic level, the improvement observed among patients with left-sided motor symptoms was correlated with metabolic modifications in a right-lateralized network known to be involved in emotional prosody, while the behavioral worsening observed

among patients with right-sided motor symptoms was correlated with metabolic modifications in the left parahippocampal gyrus and right cerebellum. We suggest that surgery has a differential impact on emotional processing according to motor symptom lateralization, and interpret these results as reflecting the (de)synchronization of the limbic loop in the postoperative condition.

Date 2020 Jul
Language eng
License Copyright © 2020. Published by Elsevier Ltd.
Extra Place: England
Volume 144
Pages 107494
Publication Neuropsychologia
DOI 10.1016/j.neuropsychologia.2020.107494
Journal Abbr Neuropsychologia
ISSN 1873-3514 0028-3932
PMID 32413433
Date Added 6.7.2025, 19:09:39
Modified 5.9.2025, 15:00:05

Notes:

Included

Study pre-post DBS. Reported results here only pre DBS.

PET scans are only post-DBS, hence not presented here.

Sample characteristics

Size: PD (primarily left-affected system onset; LPD) = 13; RPD = 16; HC = 29

PD-type: NA

PD-duration: RPD: M=11.19 SD=3.58; LPD: M=11.15 SD=4.69

Medication: ON state

Hoehn-Yahr-pre (ON): RPD: M=1.1 SD= 0.99, LPD: M=1.03 SD= 0.83

UPDRS-3-pre (ON): RPD: M=8.16 SD= 5.32, LPD: M=8.27 SD=6.39

Gender (male): LPD: 7 (54%); RPD: 8 (50%)

Age (M+-SD): RPD: M=56.13 SD= 6.69; LPD: M= 57.00 SD=9.60

Other neurological disease (tumor, stroke, etc.): none

Other major psychopathology: NA

Origin country (or ethnicity): France

method observational

instruments used in order to quantify the variables

Social cognition aspect: emotion recognition

Name of the task: NA

Type of stimulus [face/voice etc., Ekman faces/other etc.]: Set of vocal stimuli from validated database, short segments of meaningless speach. obtained by concatenating different syllables found in Indo-European languages so that they would be perceived of as natural utterances, with emotional intonation (across different cultures) but no semantic content. utterances produced by 12 different actors (6 women and 6 men)

Task condition: Four categories of emotional prosody (anger, fear, happiness, and sadness), together with a neutral condition

Operationalization: Percentage of correct responses. Participants were required to listen to each stimulus, after which they were asked to rate its emotional content on a set of visual analogue scales ranging from Not at all to Very much, which were simultaneously displayed on the computer screen.

Main findings related to the review's scope

LPD had lower accuracy in happy and neutral rate then RPD or HC. no sig dif between RPD and HC.

LPD had lower accuracy in sadness then HC. no sig dif between RPD and HC or between LPD and RPD.

anger, fear - no sig dif between the three groups.

Tags: emotion recognition, behavioral

Moving beyond basic emotions in Parkinson's disease.

**Item Type**	Journal Article
**Author**	Sarah P. Coundouris
**Author**	Julie D. Henry
**Author**	Alexander C. Lehn
**Abstract**	OBJECTIVE: Emotion recognition is a fundamental neurocognitive capacity that is a critical predictor of interpersonal function and, in turn, mental health. Although people with Parkinson's disease (PD) often exhibit difficulties recognizing emotions, almost all studies to date have focused on basic emotions (happiness, sadness, anger, surprise, fear, and disgust), with little consideration of how more cognitively complex self-conscious emotions such as contempt, embarrassment, and pride might also be affected. Further, the few studies that have considered self-conscious emotions have relied on high intensity, static stimuli. The aim of the present study was to therefore provide the first examination of how self-conscious emotion recognition is affected by PD using a dynamic, dual-intensity measure that more closely captures how emotion recognition judgements are made in daily life. METHOD: People with PD (n = 42) and neurotypical controls (n = 42) completed a validated measure of self-conscious facial emotion recognition. For comparative purposes, in addition to a broader clinical test battery, both groups also completed a traditional static emotion recognition measure and a measure of self-conscious emotional experience. RESULTS: Relative to controls, the PD group did not differ in their capacity to recognize basic emotions but were impaired in their recognition of self-conscious emotions. These difficulties were associated with elevated negative affect and poorer subjective well-being. CONCLUSIONS: Difficulties recognizing self-conscious emotions may be more problematic for people with PD than difficulties recognizing basic ones, with implications for interventions focused on helping people with this disorder develop and maintain strong social networks. PRACTITIONER POINTS: This is the first direct investigation into how the recognition of self-conscious emotion is affected in Parkinson's disease using dynamic, dual-intensity stimuli, thus providing an important extension to prior literature that has focused solely on basic emotion recognition and/or relied on static, high-intensity stimuli. Results revealed preserved basic facial emotional recognition coexisting with impairment in all three self-conscious emotions assessed, therefore suggesting that the latter stimuli type may function as a more sensitive indicator of Parkinson's disease-related social cognitive impairment. Problems with self-conscious emotion recognition in people with Parkinson's disease were associated with poorer broader subjective well-being and increased negative affect. This aligns with the broader literature linking interpersonal difficulties with poorer clinical outcomes in this cohort.
**Date**	2022 Sep
**Language**	eng
**License**	© 2022 The British Psychological Society.
**Extra**	Place: England
**Volume**	61
**Pages**	647-665

Publication The British journal of clinical psychology
DOI 10.1111/bjc.12354
Issue 3
Journal Abbr Br J Clin Psychol
ISSN 0144-6657
PMID 35048398
Date Added 6.7.2025, 19:09:38
Modified 5.9.2025, 14:31:52

Notes:

Included

sample characteristics

size: 42 PD and 42 HC

Parkinson's Disease type and duration: NA , Mduration= 6.26 (SD=3.71)

Medication: on medication

Hoehn-Yahr: *M* = 2.36, *SD* = 0.88 (1-4)

UPDRS-3: NA

Gender (male): 21 males (50%)

averaged ages (SD, range): M= 64.81 (SD=10.20)

other neurological disease (tumor, stroke, etc.): None

other major psychopathology: None

origin country (or ethnicity): Australia

method observational

instruments used in order to quantify the variables

Social cognition aspect: emotion recognition

Name of the task: ADFES-BIV

type of stimulus [face/voice etc., Ekman faces/other etc.]: presented videos of three males and females. Each one second video begins with a neutral blank stare (Figure **1a**) before developing into one of the emotional expressions (Figure **1b** low intensity; Figure **1c** high intensity) or remaining neutral.

task condition: neutral, basic (anger, disgust, fear, sadness, surprise, and happiness), and self-conscious (contempt, embarrassment, and pride) emotional expressions at two standardised intensities (low, high)

Name of the task: FACES

type of stimulus [face/voice etc., Ekman faces/other etc.]: conventional validated static emotion recognition. posed, high-intensity facial expressions of five basic emotions.

task condition: (anger, sadness, disgust, fear, and happiness) and a neutral expression

operationalization: accuracy

Main findings related to the review's scope

the PD group did not differ from HC in their capacity to recognize basic emotions

compared to the control group, the PD group was significantly impaired in their recognition of all three self-conscious emotions (contempt, embarrassment, and pride)

contempt was most frequently misconstrued as neutral in both groups. However, the PD participants

were over 10 times as likely to incorrectly recognize contempt as anger, and three times more likely to identify contempt as pride.

Tags: Emotion recognition, behavioral

MRI biomarkers of motor and non-motor symptoms in Parkinson's disease

Item Type Journal Article

Author Sephira G. Ryman

Author Kathleen L. Poston

Abstract Parkinson's disease is a heterogeneous disorder with both motor and non-motor symptoms that contribute to functional impairment. To develop effective, disease modifying treatments for these symptoms, biomarkers are necessary to detect neuropathological changes early in the disease course and monitor changes over time. Advances in MRI scan sequences and analytical techniques present numerous promising metrics to detect changes within the nigrostriatal system, implicated in the cardinal motor symptoms of the disease, and detect broader dysfunction involved in the non-motor symptoms, such as cognitive impairment. There is emerging evidence that iron sensitive, neuromelanin sensitive, diffusion sensitive, and resting state functional magnetic imaging measures can capture changes within the nigrostriatal system. Iron, neuromelanin, and diffusion sensitive measures demonstrate high specificity and sensitivity in distinguishing Parkinson's disease relative to controls, with inconsistent results differentiating Parkinson's disease relative to atypical parkinsonian disorders. They may also serve as useful monitoring biomarkers, with each possibly detecting different aspects of the disease course (early nigrosome changes versus broader substantia nigra changes). Investigations of non-motor symptoms, such as cognitive impairment, require careful consideration of the nature of cognitive deficits to characterize regional and network specific impairment. While the early, executive dysfunction observed is consistent with nigrostriatal degeneration, the memory and visuospatial impairments, the harbingers of a dementia process reflect dopaminergic independent dysfunction involving broader regions of the brain.

Date 2020

URL https://www.sciencedirect.com/science/article/pii/S1353802019304298

Volume 73

Pages 85-93

Publication Parkinsonism & Related Disorders

DOI https://doi.org/10.1016/j.parkreldis.2019.10.002

ISSN 1353-8020

Date Added 6.7.2025, 19:12:35

Modified 5.9.2025, 14:54:34

Notes:

Not Included: does not Study SC

Tags: EXCLUDED

Multidimensional Emotion Recognition Based on Semantic Analysis of Biomedical EEG Signal for Knowledge Discovery in Psychological Healthcare

**Item Type**	Journal Article
**Author**	Ling Wang
**Author**	Hangyu Liu
**Author**	Tiehua Zhou
**Author**	Wenlong Liang
**Author**	Minglei Shan
**Abstract**	Electroencephalogram (EEG) as biomedical signal is widely applied in the medical field such as the detection of Alzheimer's disease, Parkinson's disease, etc. Moreover, by analyzing the EEG-based emotions, the mental status of individual can be revealed for further analysis on the psychological causes of some diseases such as cancer, which is considered as a vital factor on the induction of certain diseases. Therefore, once the emotional status can be correctly analyzed based on EEG signal, more healthcare-oriented applications can be furtherly carried out. Currently, in order to achieve efficiency and accuracy, diverse amounts of EEG-based emotions recognition methods generally extract features by analyzing the overall characteristics of signal, along with optimization strategy of channel selection to minimize the information redundancy. Those methods have been proved their effectiveness, however, there still remains a big challenge when applied with single channel information for emotion recognition task. Therefore, in order to recognize multidimensional emotions based on single channel information, an emotion quantification analysis (EQA) method is proposed to objectively analyze the semantically similarity between emotions in valence-arousal domains, and a multidimensional emotion recognition (EMER) model is proposed on recognizing multidimensional emotions according to the partial fluctuation pattern (PFP) features based on single channel information, and result shows that even though semantically similar emotions are proved to have similar change patterns in EEG signals, each single channel of 4 frequency bands can efficiently recognize 20 different emotions with an average accuracy above 93% separately.
**Date**	2021-02
**Language**	English
**Extra**	Place: ST ALBAN-ANLAGE 66, CH-4052 BASEL, SWITZERLAND Type: Article
**Volume**	11
**Publisher**	MDPI
**Publication**	APPLIED SCIENCES-BASEL
**DOI**	10.3390/app11031338
**Issue**	3
**Date Added**	14.7.2025, 14:50:31
**Modified**	5.9.2025, 15:00:37

Notes:

Not Included: Not SC-study

Tags: EXCLUDED

Multimodal mechanisms of human socially reinforced learning across neurodegenerative diseases

**Item Type**	Journal Article
**Author**	Agustina Legaz
**Author**	Sofía Abrevaya
**Author**	Martín Dottori
**Author**	Cecilia González Campo
**Author**	Agustina Birba
**Author**	Miguel Martorell Caro
**Author**	Julieta Aguirre
**Author**	Andrea Slachevsky
**Author**	Rafael Aranguiz
**Author**	Cecilia Serrano
**Author**	Claire M Gillan
**Author**	Iracema Leroi
**Author**	Adolfo M García
**Author**	Sol Fittipaldi
**Author**	Agustín Ibañez
**Abstract**	AbstractSocial feedback can selectively enhance learning in diverse domains. Relevant neurocognitive mechanisms have been studied mainly in healthy persons, yielding correlational findings. Neurodegenerative lesion models, coupled with multimodal brain measures, can complement standard approaches by revealing direct multidimensional correlates of the phenomenon.To this end, we assessed socially reinforced and non-socially reinforced learning in 40 healthy participants as well as persons with behavioural variant frontotemporal dementia (n = 21), Parkinson's disease (n = 31) and Alzheimer's disease (n = 20). These conditions are typified by predominant deficits in social cognition, feedback-based learning and associative learning, respectively, although all three domains may be partly compromised in the other conditions. We combined a validated behavioural task with ongoing EEG signatures of implicit learning (medial frontal negativity) and offline MRI measures (voxel-based morphometry).In healthy participants, learning was facilitated by social feedback relative to non-social feedback. In comparison with controls, this effect was specifically impaired in behavioural variant frontotemporal dementia and Parkinson's disease, while unspecific learning deficits (across social and non-social conditions) were observed in Alzheimer's disease. EEG results showed increased medial frontal negativity in healthy controls during social feedback and learning. Such a modulation was selectively disrupted in behavioural variant frontotemporal dementia. Neuroanatomical results revealed extended temporo-parietal and fronto-limbic correlates of socially reinforced learning, with specific temporo-parietal associations in behavioural variant frontotemporal dementia and predominantly fronto-limbic regions in Alzheimer's disease. In contrast, non-socially reinforced learning was consistently linked to medial temporal/hippocampal regions. No associations with cortical volume were found in Parkinson's disease. Results are consistent with core social deficits in behavioural variant frontotemporal dementia, subtle disruptions in ongoing feedback-mechanisms and social processes in Parkinson's disease and generalized learning alterations in Alzheimer's disease. This multimodal approach highlights the impact of different neurodegenerative profiles on learning and social

feedback.Our findings inform a promising theoretical and clinical agenda in the fields of social learning, socially reinforced learning and neurodegeneration.

**Date**	2022-04-29
**Language**	en
**Library Catalog**	Crossref
**URL**	https://academic.oup.com/brain/article/145/3/1052/6371182
**Accessed**	13.7.2025, 20:22:42
**License**	https://creativecommons.org/licenses/by-nc/4.0/
**Volume**	145
**Publisher**	Oxford University Press (OUP)
**Pages**	1052-1068
**Publication**	Brain
**DOI**	10.1093/brain/awab345
**Issue**	3
**ISSN**	0006-8950, 1460-2156
**Date Added**	13.7.2025, 20:22:42
**Modified**	5.9.2025, 14:43:14

Notes:

Not Included: Not assessing SC

Tags: EXCLUDED

Multimodal Neurocognitive Markers of Naturalistic Discourse Typify Diverse Neurodegenerative Diseases

**Item Type**	Journal Article
**Author**	Agustina Birba
**Author**	Sol Fittipaldi
**Author**	Judith C. Cediel Escobar
**Author**	Cecilia Gonzalez Campo
**Author**	Agustina Legaz
**Author**	Agostina Galiani
**Author**	Mariano N. Diaz Rivera
**Author**	Miquel Martorell Caro
**Author**	Florencia Alifano
**Author**	Stefanie D. Pina-Escudero
**Author**	Juan Felipe Cardona
**Author**	Alejandra Neely
**Author**	Gonzalo Forno
**Author**	Mari Carpinella
**Author**	Andrea Slachevsky
**Author**	Cecilia Serrano
**Author**	Lucas Sedeno

Author Agustin Ibanez
Author Adolfo M. Garcia
Abstract Neurodegeneration has multiscalar impacts, including behavioral, neuroanatomical, and neurofunctional disruptions. Can disease-differential alterations be captured across such dimensions using naturalistic stimuli? To address this question, we assessed comprehension of four naturalistic stories, highlighting action, nonaction, social, and nonsocial events, in Parkinson's disease (PD) and behavioral variant frontotemporal dementia (bvFTD) relative to Alzheimer's disease patients and healthy controls. Text-specific correlates were evaluated via voxel-based morphometry, spatial (fMRI), and temporal (hd-EEG) functional connectivity. PD patients presented action-text deficits related to the volume of action-observation regions, connectivity across motor-related and multimodal-semantic hubs, and frontal hd-EEG hypoconnectivity. BvFTD patients exhibited social-text deficits, associated with atrophy and spatial connectivity patterns along social-network hubs, alongside right frontotemporal hd-EEG hypoconnectivity. Alzheimer's disease patients showed impairments in all stories, widespread atrophy and spatial connectivity patterns, and heightened occipitotemporal hd-EEG connectivity. Our framework revealed disease-specific signatures across behavioral, neuroanatomical, and neurofunctional dimensions, highlighting the sensitivity and specificity of a single naturalistic task. This investigation opens a translational agenda combining ecological approaches and multimodal cognitive neuroscience for the study of neurodegeneration.
Date 2022-08-03
Language English
Extra Place: JOURNALS DEPT, 2001 EVANS RD, CARY, NC 27513 USA Type: Article
Volume 32
Publisher OXFORD UNIV PRESS INC
Pages 3377-3391
Publication CEREBRAL CORTEX
DOI 10.1093/cercor/bhab421
Issue 16
ISSN 1047-3211
Date Added 14.7.2025, 14:50:30
Modified 5.9.2025, 14:28:42

Notes:

Not Included: not on SC

Tags: EXCLUDED

Neural signatures underlying the effect of social structure on empathy and altruistic behaviors

Item Type Journal Article
Author Xia Tian
Author Zixin Zheng
Author Renhui Li
Author Yue-Jia Luo

Author Chunliang Feng

Abstract Humans inhabit complex social networks, monitoring social structures that encompass both direct and indirect relationships. However, previous research primarily focused on direct relationships, leaving the neural basis of how social structure influences socioemotional processes understudied. This study addressed this gap by investigating the neural pathways underlying the influence of social structure on empathy and altruistic behaviors. During fMRI scanning, participants viewed painful or non-painful stimulation to innocent strangers who shared preferences with targets who had either treated participants fairly or unfairly. Afterwards, participants rated the pain experienced by these innocents and shared money with other innocents. Participants showed reduced empathic and altruistic responses toward innocents resembling unfair (vs. fair) targets, accompanied by heightened activation in regions crucial for emotion regulation and mentalizing, such as the lateral and medial prefrontal cortex. Furthermore, whole-brain and local neural patterns in the anterior insula and premotor cortex robustly discriminated painful (but not non-painful) stimulation of different innocents, suggesting that social structure altered emotional and sensorimotor aspects of empathy. These alterations might be driven by top-down regulation, as indicated by heightened functional connectivity between the lateral prefrontal cortex and sensorimotor areas, as well as between the anterior insula and subgenual anterior cingulate cortex when witnessing the pain of innocents resembling fair (vs. unfair) targets. Together, our work is the first to uncover the neural underpinnings through which human empathy and altruistic behaviors are shaped by social structure beyond direct self-other relationships.

Date 2025
URL https://www.sciencedirect.com/science/article/pii/S1053811925002708
Volume 315
Pages 121267
Publication NeuroImage
DOI https://doi.org/10.1016/j.neuroimage.2025.121267
ISSN 1053-8119
Date Added 6.7.2025, 19:12:34
Modified 5.9.2025, 14:58:33

Notes:

Not Included: No PD group

Tags: EXCLUDED

Neuroanatomical and neurochemical bases of theory of mind

Item Type Journal Article

Author Ahmad Abu-Akel
Author Simone Shamay-Tsoory

Abstract This paper presents a novel neurobiological model of theory of mind (ToM) that incorporates both neuroanatomical and neurochemical levels of specificity. Within this model, cortical and subcortical regions are functionally organized into networks that subserve the ability to represent cognitive and affective mental states to both self and other. The model maintains that (1) cognitive and affective aspects of ToM are

subserved by dissociable, yet interacting, prefrontal networks. The cognitive ToM network primarily engages the dorsomedial prefrontal cortex, the dorsal anterior cingulate cortex and the dorsal striatum; and the affective ToM network primarily engages the ventromedial and orbitofrontal cortices, the ventral anterior cingulate cortex, the amygdala and the ventral striatum; (2) self and other mental-state representation is processed by distinct brain regions within the mentalizing network, and that the ability to distinguish between self and other mental states is modulated by a functionally interactive dorsal and ventral attention/selection systems at the temporoparietal junction and the anterior cingulate cortex; and (3) ToM functioning is dependent on the integrity of the dopaminergic and serotonergic systems which are primarily engaged in the maintenance and application processes of represented mental states. In addition to discussing the mechanisms involved in mentalizing in terms of its component processes, we discuss the model's implications to pathologies that variably impact one's ability to represent, attribute and apply mental states. (C) 2011 Elsevier Ltd. All rights reserved.

Date 2011-09
Language English
Extra Place: THE BOULEVARD, LANGFORD LANE, KIDLINGTON, OXFORD OX5 1GB, ENGLAND Type: Review
Volume 49
Publisher PERGAMON-ELSEVIER SCIENCE LTD
Pages 2971-2984
Publication NEUROPSYCHOLOGIA
DOI 10.1016/j.neuropsychologia.2011.07.012
Issue 11
ISSN 0028-3932
Date Added 14.7.2025, 14:50:40
Modified 5.9.2025, 14:24:15

Notes:

Not Included: not a systematic review

Tags: EXCLUDED

Neuroanatomical correlates of impaired decision-making and facial emotion recognition in early Parkinson's disease.

Item Type Journal Article
Author Naroa Ibarretxe-Bilbao
Author Carme Junque
Author Eduardo Tolosa
Author Maria-Jose Marti
Author Francesc Valldeoriola
Author Nuria Bargallo
Author Mojtaba Zarei

Abstract Decision-making and recognition of emotions are often impaired in patients with Parkinson's disease (PD). The orbitofrontal cortex (OFC) and the amygdala are critical structures subserving these functions. This study was designed to test whether there are any structural changes in these areas that might explain the impairment of decision-making and recognition of facial emotions in early PD. We used the Iowa Gambling Task (IGT) and the Ekman 60 faces test which are sensitive to the integrity of OFC and amygdala dysfunctions in 24 early PD patients and 24 controls. High-resolution structural magnetic resonance images (MRI) were also obtained. Group analysis using voxel-based morphometry (VBM) showed significant and corrected ($P < 0.05$ FEW-small volume correction) gray matter (GM) loss in the right amygdala and bilaterally in the OFC in PD patients. Volumetric analyses were also performed but did not yield significant differences between groups. Left lateral GM volume in OFC showed a slight correlation with the IGT, and bilateral OFC GM was strongly correlated with Ekman test performance in PD patients. We conclude that: (i) impairment in decision-making and recognition of facial emotions occurs at the early stages of PD, (ii) these neuropsychological deficits are accompanied by degeneration of OFC and amygdala, and (iii) bilateral OFC reductions are associated with impaired recognition of emotions, and GM volume loss in left lateral OFC is related to decision-making impairment in PD.

Date 2009 Sep
Language eng
Extra Place: France
Volume 30
Pages 1162-1171
Publication The European journal of neuroscience
DOI 10.1111/j.1460-9568.2009.06892.x
Issue 6
Journal Abbr Eur J Neurosci
ISSN 1460-9568 0953-816X
PMID 19735293
Date Added 6.7.2025, 19:09:36
Modified 5.9.2025, 14:40:00

Notes:

Included

sample characteristics

size: 24 PD and 24 HC (matched by age, gender and years of education)

Parkinson's Disease type and duration: Mduration = 3.06 SD=1.6

Medication: Most on medication (3 not)

Hoehn-Yahr: M= 1.73 SD=0.4

UPDRS-3: M= 14.67 SD=3.5

Gender (male): 16 males (67%)

averaged ages (SD, range): M= 56.13 SD=8.5

other neurological disease (tumor, stroke, etc.): None

inclusion criteria for patients were: (i) age 40–65 years; (ii) Hoehn and Yahr stage < II; (iii) disease duration < 5 years; and (iv) absence of motor fluctuations.

other major psychopathology: None

origin country (or ethnicity): Spain

method observational and imaging

instruments used in order to quantify the variables

Social cognition aspect: emotion recognition

Name of the task: NA

type of stimulus [face/voice etc., Ekman faces/other etc.]: Ekman (six female, four male) from the Ekman and Friesen series of Pictures of Facial Affect

task condition: anger, disgust, fear, happiness, sadness or surprise

operationalization: subjects must decide whether the expression corresponds to anger, disgust, fear, happiness, sadness or surprise. >> accuracy.

MRI: Images were acquired using a TIM TRIO 3T scanner (Siemens, Germany). A set of high-resolution 3-dimensional T1-weighted images was acquired with a MPRAGE sequence in sagittal orientation (TR/TE = 2300/2.98 ms; TI = 900 ms; 256 × 256 matrix, 1 mm isotropic voxel).

VBM - carried out with FSL software. averaged to create a study-specific template, to which the native GM images were then non-linearly re-registered. The modulated segmented images were then smoothed with an isotropic Gaussian kernel with a sigma of 3.5 mm (8 mm FWHM).

In addition to VBM analysis, volumetric analysis of the orbitofrontal cortex (OFC), amygdala and

primary olfactory cortex were performed.

Main findings related to the review's scope

PD performed worse on the Ekman total score than HC.

PD patients obtained lower scores than HC in all emotions except happiness.

Performance on recognition of happiness was very similar between PD and HC and a ceiling-effect was observed for this emotion

The Ekman total score correlated positively with OFC volume after adjusting for GM total volume. This correlation was corrected for multiple comparisons and as a result we obtained a big cluster embracing left and right OFC. OFC GM volume did not correlate with Ekman performance in HC group.

Tags: Emotion recognition, Imaging, behavioral

Neuroanatomical Correlates of Theory of Mind Deficit in Parkinson's Disease: A Multimodal Imaging Study.

Item Type Journal Article

Author María Díez-Cirarda
Author Natalia Ojeda
Author Javier Peña
Author Alberto Cabrera-Zubizarreta
Author María Ángeles Gómez-Beldarrain
Author Juan Carlos Gómez-Esteban
Author Naroa Ibarretxe-Bilbao

Abstract BACKGROUND: Parkinson's disease (PD) patients show theory of mind (ToM) deficit since the early stages of the disease, and this deficit has been associated with working memory, executive functions and quality of life impairment. To date, neuroanatomical correlates of ToM have not been assessed with magnetic resonance imaging in PD. The main objective of this study was to assess cerebral correlates of ToM deficit in PD. The second objective was to explore the relationships between ToM, working memory and executive functions, and to analyse the neural correlates of ToM, controlling for both working memory and executive functions. METHODS: Thirty-seven PD patients (Hoehn and Yahr median = 2.0) and 15 healthy controls underwent a neuropsychological assessment and magnetic resonance images in a 3T-scanner were acquired. T1-weighted images were analysed with voxel-based morphometry, and white matter integrity and diffusivity measures were obtained from diffusion weighted images and analysed using tract-based spatial statistics. RESULTS: PD patients showed impairments in ToM, working memory and executive functions; grey matter loss and white matter reduction compared to healthy controls. Grey matter volume decrease in the precentral and postcentral gyrus, middle and inferior frontal gyrus correlated with ToM deficit in PD. White matter in the superior longitudinal fasciculus (adjacent to the parietal lobe) and white matter adjacent to the frontal lobe correlated with ToM impairment in PD. After controlling for executive functions, the relationship between ToM deficit and white matter remained significant

for white matter areas adjacent to the precuneus and the parietal lobe. CONCLUSIONS: Findings reinforce the existence of ToM impairment from the early Hoehn and Yahr stages in PD, and the findings suggest associations with white matter and grey matter volume decrease. This study contributes to better understand ToM deficit and its neural correlates in PD, which is a basic skill for development of healthy social relationships.

Date 2015
Language eng
Extra Place: United States
Volume 10
Pages e0142234
Publication PloS one
DOI 10.1371/journal.pone.0142234
Issue 11
Journal Abbr PLoS One
ISSN 1932-6203
PMID 26559669
PMCID PMC4641650
Date Added 6.7.2025, 19:09:40
Modified 5.9.2025, 14:33:48

Notes:

Included

sample characteristics

size: 37 PD and 15 HC (matched in age, gender and years of education)

Parkinson's Disease type and duration: NA

Medication: on medication

Hoehn-Yahr: M=1.89 SD=0.45 (1-3)

UPDRS-3: M=36.61 SD=17.27

Gender (male): 22 males (60%)

averaged ages (SD, range): M=67.97 SD=6.17

other neurological disease (tumor, stroke, etc.): None

other major psychopathology: None

origin country (or ethnicity): Spain

method observational and imaging

instruments used in order to quantify the variables

Social cognition aspect: ToM

Name of the task: the Happe Test "Strange Stories Task"

type of stimulus [face/voice etc., Ekman faces/other etc.]: 8 stories concerning double bluff, mistakes, persuasion and white lies, 4 used now. Participants had to read aloud each story and then, answer a question requiring an inference about the character's thoughts, which required an inference about the speaker's/actor's intentions. The participant was asked to answer the required questions, explaining his/her point of view, after demonstrating that he/she understood the task with an example story.

operationalization: the global score was selected. Responses were scored between 0 and 2, strictly following the instructions of the manual, where explicit answers were scored with 2 points and implicit answers with 1 point and no response or non-related responses with 0 points

MRI: Diffusion-weighted images were obtained on a Phillips 3T Achieva, in an axial orientation in an anterior-posterior phase direction using a single-shot EPI sequence (TR = 7540 and TE = 76, matrix size = 120mm x 117mm; flip angle = 90°, FOV = 240x240x132, slice thickness = 2 mm, no gap, 66 slices, acquisition time = 9'31", voxel size = 1.67x1.67x2.0) with diffusion weighting in 32 uniformly distributed directions (b = 1,000 s/mm^2) and 1 b = 0 s/mm^2. A T1-weighted scan was also acquired in sagittal orientation (TR = 7.4 and TE = 3.4, matrix size = 228mm x 218mm; flip angle = 9°, FOV = 250x250x180, slice thickness = 1.1 mm, 300 slices, acquisition time = 4'55", voxel size = 0.98x0.98x0.6).

VBM - VBM analyses were carried out using the FMRIB Software Library (FSL). a study-specific template was created so that all images could be registered in the same stereotactic space (spatial normalisation). affine registered to the GM ICBM-152 template and averaged to create an affine GM template. After the normalisation, the resulting GM images were modulated by multiplying with Jacobian determinants to correct for the volume change induced by the nonlinear spatial normalisation. Finally, the images were smoothed with sigma of 3.5 mm (8 mm FWHM).

TBSS - Diffusion data were preprocessed and analysed using tools from FSL. each subject's images were concatenated and radiologically oriented. Then, the data were corrected for motion and eddy currents, performed brain-extraction BET, and the diffusion gradients (bvecs) were rotated to be corrected accordingly, providing a more accurate estimate of tensor orientations [40]. Then, all FA, MD, RD and AD images were obtained by fitting a tensor model to the raw diffusion data using FDT (DTIFIT). After, TBSS [41] was used for group comparisons and correlations analyses. Using TBSS, the data were prepared to apply a nonlinear registration of all FA images into standard space, the mean FA image was created using a threshold of 0.2 and thinned to create a "mean FA skeleton" which represents the centres of all tracts common to the group. MD data were analysed using "tbss non FA" script from TBSS, which applies the original non lineal registration to the MD data, merges all subjects warped MD data into a 4D file, then project this onto the original mean FA skeleton, and creates the 4D projected data. The same process was repeated for RD and AD.

Main findings related to the review's scope

behavioral:

HC had better score when compared to PD in the ToM test

imaging:

GM >>

PD patients showed reduced GM volume in the left temporal, parietal and occipital lobes.

GM regions that showed potential reductions in PD patients compared to healthy controls were mostly located in the left inferior temporal gyrus (anterior and posterior division) and the temporal fusiform cortex

	Cluster size (voxels)	MNI coordinate			t value	*p* value	Effect size (Cohen´s *d* / *r*) *df* = 36
		x	y	z			
**Group Comparison**							
L Inferior temporal gyrus	1973	-48	-10	-48	3.77	.001*	1.15
	267	52	-18	-40	3.05	.003*	.84
L Lateral Occipital Cortex, L Superior Parietal Lobe	745	-18	-76	56	3.33	.001*	.93
L Inferior Parietal Lobe	582	-44	-44	22	2.99	.001*	.91
R Temporal Lobe	158	44	26	-26	3.73	.003*	1.14
**Correlation with ToM in PD**							
L Precentral gyrus, L Postcentral gyrus	830	-32	-24	64	3.59	< .001*	.51
L Anterior Cingulate gyrus	147	-4	-12	28	2.24	< .001*	.34
	109	0	32	2	2.91	< .001*	.43
L Middle frontal gyrus, L Inferior frontal gyrus	84	-30	18	32	2.17	< .001*	.34

Cluster size denotes the extent of the cluster of significant voxels. MNI coordinates refer to the location of the most statistically significant voxel in the cluster.
*Differences are significant at $p < .001$ uncorrected.
PD = Parkinson's disease; ToM = Theory of Mind; L = Left; R = Right; MNI = Montreal Neurological Institute. *df* = Degrees of Freedom.

No significant correlations were observed at $p < .05$ (FWE-corrected) statistical threshold, but the exploratory analysis showed possible associations between ToM and GM volume, in the left precentral and postcentral gyrus, anterior cingulate gyrus, middle frontal gyrus and the inferior frontal gyrus in PD (Table 2) ($p < .001$ uncorrected). Healthy controls' performance in ToM test showed no significant correlation with GM volume.

Because executive functions showed a positive association with ToM, we also included this variable as a covariate in the regression analysis. No significant clusters were obtained in the regression analysis between GM volume decrease and ToM after controlling for executive functions in PD.

WM >>

PD patients showed FA reduction in the right uncinate fasciculus adjacent to the insular cortex and slight WM FA reduction was observed in the frontal lobe compared to healthy control group ($p < .001$ uncorrected). Results showed no significant differences between PD and healthy control groups in MD, RD or AD indexes.

ToM deficit in PD patients correlated positively with FA reduction and negatively with MD and RD indexes of WM tracts. Most significant correlations were found between WM and ToM deficit in the bilateral superior longitudinal fasciculus in PD

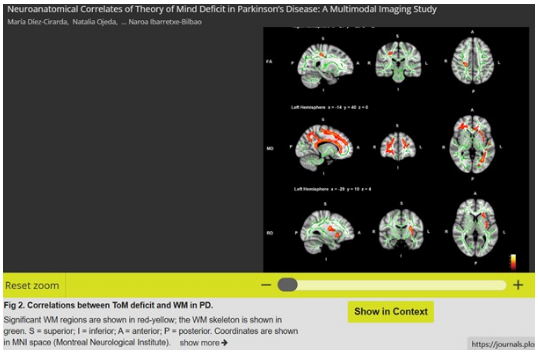

	(voxels)	coordinate			value	value	*df* = 36
		x	y	z			
**Group Comparison**							
**FA**							
R Uncinate Fasciculus	42	34	15	-13	2.95	.001*	.81
**Correlation with ToM in PD**							
**FA**							
R Superior Longitudinal Fasciculus	97	23	-29	42	5.18	.032**	.65
**MD**							
L Superior longitudinal Fasciculus	13046	-40	-55	36	5.86	.031**	.69
	259	-31	14	28	5.20	.040**	.65
L External Capsule	483	-33	3	4	4.14	.039**	.56
**RD**							
L Superior Longitudinal Fasciculus	650	-30	-10	14	3.8	.040**	.53
**Correlation with ToM in PD controlling for Executive Functions**							
**FA**							
R Superior Longitudinal Fasciculus	227	28	-26	43	2.61	.002*	.39
**MD**							
L Superior Longitudinal Fasciculus	1322	-40	-55	35	5.33	.049**	.66
	878	-19	-45	43	3.93	.043**	.54
L Inferior Longitudinal Fasciculus	165	-31	-69	-1	4.04	.049**	.55
**RD**							
R Corticoespinal Tract	296	14	-13	66	1.24	.002*	.20
	124	-27	-20	63	0.86	.001*	.14
L Inferior Longitudinal Fasciculus	169	-27	-63	47	1.69	.001*	.27
	115	-28	-8	-16	1.23	.001*	.20
L Superior Longitudinal Fasciculus	109	-58	-26	6	1.31	.002*	.21

Cluster size denotes the extent of the cluster of significant voxels. MNI coordinates refer to the location of the most statistically significant voxel in the cluster.

*Differences are significant at $p < .001$ uncorrected.

**Differences are significant at $p < .05$ corrected for family-wise error (FWE).

PD = Parkinson's disease; ToM = Theory of Mind; R = Right; L = Left; FA = Fractional Anisotropy; MD = Mean Diffusivity; RD = Radial Diffusivity; MNI = Montreal Neurological Institute; *df* = Degrees of Freedom.

WM FA reduction in the right superior longitudinal fasciculus and corticospinal tract adjacent to the primary somatosensory cortex correlated with ToM deficit in PD (Brodmann Area 3a) ($p < .05$ FWE-corrected)

In addition, MD index correlated negatively with ToM deficit in PD, and significant clusters were found in the left superior longitudinal fasciculus located longitudinally in the superior frontal gyrus and premotor cortex (Brodmann Area 6), continuing through the primary somatosensory cortex in the parietal lobe, the precuneus and finishing in the occipital cortex. Furthermore, MD in the left inferior longitudinal fasciculus, the right inferior fronto-occipital fasciculus and the left uncinate fasciculus, adjacent to middle temporal gyrus, to the orbitofrontal cortex, and frontal lobe respectively also correlated with ToM deficit in PD. Finally, MD in the callosal body also correlated with ToM impairment in PD patients

Moreover, RD in the left superior longitudinal fasciculus and corticospinal tract adjacent to the secondary somatosensory cortex, in the external capsule, and in the left anterior thalamic radiation and inferior fronto-occipital fasciculus in the frontal lobe, correlated negatively with ToM deficit in PD (Fig 2; Table 3).

AD index showed no significant relationship with ToM deficit in PD. No significant correlations were found between ToM performance in healthy controls and FA, RD, MD or AD

After controlling for executive functions, MD in the left superior longitudinal fasciculus adjacent to anterior intra-parietal sulcus, superior parietal lobe and precuneus showed significant associations with ToM deficit in PD (Fig 3; Table 3) ($p < .05$ FWE-corrected). Exploratory analyses showed potential associations between ToM impairment in PD and FA and RD in the right superior longitudinal fasciculus adjacent to somatosensory cortex (Table 3) ($p < .001$ uncorrected).

Tags: ToM, Imaging, behavioral

Neurobiological correlates of emotional processing in Parkinson's disease: A systematic review of experimental studies

**Item Type**	Journal Article
**Author**	Anja J. H. Moonen
**Author**	Anke Wijers
**Author**	Kathy Dujardin
**Author**	Albert F. G. Leentjens
**Abstract**	Deficits in emotional processing in patients with Parkinson's disease (PD) have received increasing interest over the past decades. In this systematic review, we present the results of 18 behavioral studies that have examined the neurobiological base of emotional processing in PD. Multiple aspects of emotional processing have been studied, using a variety of research methods. Deficits in PD are mainly related to autonomic and perceptive processing of intense emotional stimuli, which is accompanied by structural and functional neurobiological abnormalities in predominantly ventral regions of affective neurocircuitry. These structures are more strongly dependent on dopaminergic neurotransmission than the dorsal structures of affective neurocircuitry, which are more related to the cognitive and regulatory aspects of emotion and appear to remain largely intact in PD patients. Considering the importance of active dopaminergic neurotransmission, PD can serve as a prolific model for studying the neurobiological correlates of normal human emotional behavior as well as psychiatric disorders such as anxiety, depression, and apathy. Moreover, the fact that PD patients are able to cognitively regulate or modulate their emotional responses despite reduced dopamine supplies, can have important implications for the treatment of affective disorders not only in PD patients but in the general population likewise.
**Date**	2017
**URL**	https://www.sciencedirect.com/science/article/pii/S0022399917303768
**Volume**	100
**Pages**	65-76
**Publication**	Journal of Psychosomatic Research
**DOI**	https://doi.org/10.1016/j.jpsychores.2017.07.009
**ISSN**	0022-3999
**Date Added**	6.7.2025, 19:12:36
**Modified**	5.9.2025, 14:47:26

Notes:

Included: Systematic Review

Search Strategy

A systematic literature search was conducted in PubMed and PsycINFO, which was extended with searches of references listed in the reviewed papers

Box 1
Search strategy.

Search terms	
PubMed	Parkinson*(title/abstract) OR Parkinson's disease (MeSH) AND (emotion* O OR arousal OR prosody OR subjective*) AND Humans (MeSH) NOT "reviev
PsycINFO	Parkinson*[title/abstract] AND (emotion* OR facial* OR arousal OR prosody subjective*)

Papers were selected according to the following inclusion criteria: i) patients were diagnosed with idiopathic Parkinson's disease ii) emotional processing, measured in a behavioral task was the main outcome iii) the study included a neurobiological measure of emotional processing, iv) data analyses incorporated both the behavioral and neurobiological measurements of emotional processing

Quality assessment

Combined items from 2 different checklists

We selected the items that specifically rated descriptive, statistical and internal validity, leaving out those items that were not relevant for our study (e.g., items on specific pharmaceutical issues). We then expanded our selection with four additional items from the STROBE statement, concerning study design, statistical interpretation of data, and power calculation.

Items were scored as either good (2), moderate (1), or inadequate/undefined (0), which enabled us to compare the quality of included studies mutually, despite the fact that we combined items from different checklists

PRISMA

From the 4022 papers, 3974 could be excluded after reading the title and abstract alone. Further screening for eligibility resulted in 17 studies that were included for extensive review. One additional study was included after checking the reference lists of the included papers

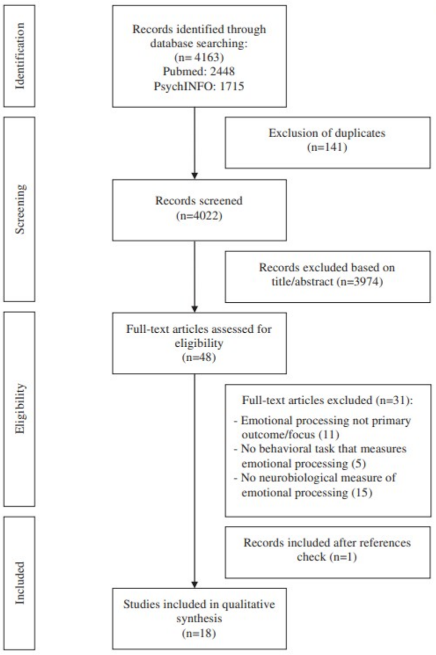

Fig.

Results

Study characteristics

The literature search resulted in 18 articles that were included for further review.

All studies were cross-sectional.

A total of 11 studies investigated facial emotion recognition, 3 studies measured physiological arousal, 2 studies looked at emotional prosody recognition, and 2 studies used a multimodal audio-visual approach. Seven studies measured neurobiological correlates of emotional processing by using electro-encephalogram (EEG) and measuring the event-related potentials (ERP) or spectral modifications (1 study), 6 used blood-oxygen-level dependent functional magnetic resonance imaging (BOLD-fMRI), 2

used structural magnetic resonance imaging (MRI), 2 used positron emission tomography (PET), and 1 used single photon emission CT (SPECT).

Methodological quality

Quality varied among included studies and stayed fairly stable over time.

The mean quality score was 26,1 (range 11–32) out of 36, with the majority of scores lying above 20.

Facial emotion recognition (FER)

fMRI

Tessitore et al: 9 early PD in ON and 9 matched HC had to match angry and fearful facial expressions:

Despite equal task performances, the robust bilateral amygdala response found in healthy subjects was absent in PD patients who were temporarily depleted from DRT. After dopamine repletion, the amygdala response was partially restored, yet remained diminished compared to the healthy subjects, which indicates that reduced dopamine availability in the amygdala is associated with functional processing deficits for emotional facial expressions

Lotze et al: 9 early to adv PD in OFF, 9 9HC had to do emotion recognition task (i.e. emotional vs. neutral gestures/faces)

PD showed more errors than HC.

PD patients showed decreased functional activity for observing emotional gestures in the left ventrolateral prefrontal cortex (VLPFC) and right superior temporal sulcus (STS). Additional PET scanning quantified less striatal dopamine transporter availability (DAT) in PD patients compared to healthy controls, with a specific association between reduced DAT in the left putamen and more severe motor impairment as well as more recognition errors in PD patients. Moreover, left putaminal DAT appeared to be positively correlated with left VLPFC activity, which indicates how disturbed dopaminergic neurotransmission can affect functional activity in this area during emotional gesture recognition.

However, these patients were not re-assessed during their on state, hence the influence of levodopa treatment remains unknown here.

Delaveau et al: 14 non-depressedPD in ON and 13 HC (non-matched). Investigated dopaminergic modulation in FER:

In non-depressed PD patients, the dopaminergic mesolimbic pathway appears to be relatively intact. Hence, DRT as treatment for motor symptoms may overdose these intact limbic regions and consequently disrupt normal amygdala functioning, as shown previously in healthy subjects

Delaveau et al: reported that PD patients showed similar right amygdala activation as the controls during a FER task when administered under placebo. However, amygdala activation was indeed abnormal in both groups after administration of levodopa.

Effect of L-Dopa on default mode network(DMN) deactivation during the FER task: Whereas the control group showed the classical deactivation of cortical areas (e.g., medial prefrontal cortex and posterior cingulate cortex), PD patients under placebo did not. However, after levodopa administration, DMN deactivation improved significantly, which again indicates that dopamine levels can modulate brain activation in regions that are essential for perceptual emotional processing.

Wabnegger et al: 17 mild to moderate PD in OFF and 22 HC (matched):

Showd comparable affective ratings when presented with negateive (i.e., disgust, anger, fear, sadness) and neutral facial expressions. Region-of-interest analyses revealed increased reactivity in somatosensory regions (all emotions) versus decreased reactivity in the putamen and inferior frontal gyrus (sadness) in PD patients versus controls. The authors speculate about a possible compensatory mechanism that enables intact FER despite striatal deficits. The specific role of dopamine was not further investigated in this study as patients were only assessed in their off-state.

Event-related potentials (ERP)

Yoshimura et al: studied FER by measuring ERPs in a sample of 9 mild to moderate PD patients in ON and 10 non-matched healthy controls

PD patients performed the task equally well as healthy controls,

ERP results showed that PD patients appeared to use different neural substrates for recognizing emotions in fearful facial expressions. More specifically, 7 out of 10 healthy subjects showed an increase in initial negative response (N1) of which the equivalent current dipoles were concentrated in the amygdala. In PD patients, on the other hand, N1 was centered bilaterally in the angular gyrus and supramarginal gyrus, and notably there was no neuronal activity in the amygdala.

(One could however question whether localization of generators with a dipole fit is suitable to identify activity in subcortical structures.)

Wieser et al: 18 mild to moderate PD in ON and 17 HC (matched) tested regarding emotional recognition accuracy and affectiave ratings of facial expressions.

Accuracy and affective ratings not impaired in PD.

However, the authours did find indirect evidence for diminished early visual discrimination of emotional facial expressions in PD, as reflected by the lack of increased early posterior activity in occipital regions that was present in HC.

Late cortical evaluative processing seemed to be intact, as PD patients showed no deficits in late components of the ERPs (i.e., late positive potentials) localized in parietal regions.

MRI

Ibarretxe-Bilbao et al: 24 PD in ON and 24 matched HC tested in facial expressions recognition task for all basic emotions excpent happiness (fear, anger, sadness, surprise, disgust).

PD performed worse for all emotions.

Voxel-based morphometry (VBM) region of interest analyses of grey matter (GM) volume revealed that PD patients showed significant GM volume loss in the right amygdala and bilateral OFC compared to healthy subjects. Interestingly, after correcting for total GM volume, a strong positive correlation between total FER performance in patients and OFC volume became apparent.

Baggio et al: 39 early PD in ON and 23 age-matched HC recognied negative facial expressions (fear, anger, disgust, sadness)

PD performed sig. worse than HC.

VBM analyses further showed positive correlations between recognition accuracy and GM volume in the right OFC, amygdala, and dorsal postcentral gyrus for sadness; in the right fusiform gyrus, ventral striatum, and subgenual cortex for anger; and in the dorsal anterior cingulate cortex (ACC) for disgust identification. In addition, diffusion tensor imaging (DTI) data revealed a positive correlation between sadness identification and white matter density in the right frontal lobe, of which the latter was

significantly reduced in PD patients.

Emotional prosody recognition

ERP

Schroder et al: 14 mild PD in ON and 14 machted HC studied for recognising emotion from prosody (recognising emotional content of spoken language).

PD patients made more errors than healthy controls in recognizing sadly spoken words in a vocal emotional processing experiment. In addition, early ERP components showed disturbed preattentive processing of sad words, whereas late ERP components showed reduced reactivity in response to happy words. The authors point to striatal dopamine deficiency as a possible explanation for the impaired early preattentive processing of emotional prosody.

Garrido-Vásquez et al: 10 LPD, 12 RPD, 22 matched HC. All PD mild to moderate and in ON.

PD patients performed equally well compared to healthy controls in categorizing different vocal emotions, although sad sounds were not included here. Data from early ERP components showed deviated ERP patterns (i.e., no P200 reduction) in LPD patients for almost all emotional categories. RPD patients and healthy controls showed no such deficit, which suggests that especially right striatal neurodegeneration might affect emotional prosody recognition

Neurobiological model for emotional processing deficits in PD (see figure)

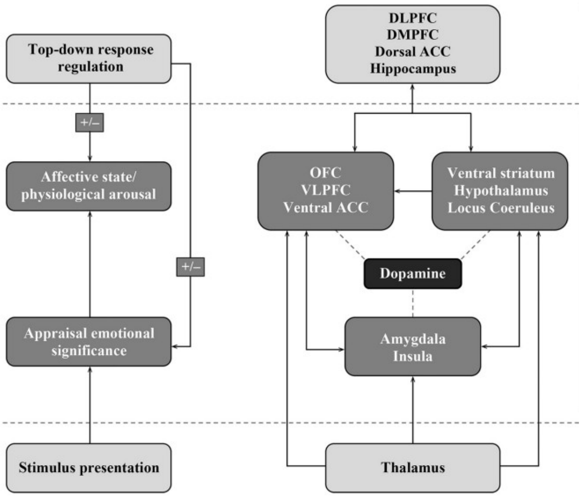

Data on physiological arousal reported but not included in this summary!

Tags: Emotion recognition, Imaging

Neurocognitive Impairment and Social Cognition in Parkinson's Disease Patients.

Item Type Journal Article
Author Triantafyllos Doskas
Author Konstantinos Vadikolias
Author Konstantinos Ntoskas
Author George D. Vavougios
Author Dimitrios Tsiptsios
Author Polyxeni Stamati
Author Ioannis Liampas
Author Vasileios Siokas
Author Lambros Messinis
Author Grigorios Nasios
Author Efthimios Dardiotis
Abstract In addition to motor symptoms, neurocognitive impairment (NCI) affects patients with prodromal Parkinson's disease (PD). NCI in PD ranges from subjective cognitive complaints to dementia. The purpose of this review is to present the available evidence of NCI in PD and highlight the heterogeneity of NCI phenotypes as well as the range of factors that contribute to NCI onset and progression. A review of publications related to NCI in PD up to March 2023 was performed using PubMed/ Medline. There is an interconnection between the neurocognitive and motor symptoms of the disease, suggesting a common underlying pathophysiology as well as an interconnection between NCI and non-motor symptoms, such as mood disorders, which may contribute to confounding NCI. Motor and non-motor symptom evaluation could be used prognostically for NCI onset and progression in combination with imaging, laboratory, and genetic data. Additionally, the implications of NCI on the social cognition of afflicted patients warrant its prompt management. The etiology of NCI onset and its progression in PD is multifactorial and its effects are equally grave as the motor effects. This review highlights the importance of the prompt identification of subjective cognitive complaints in PD patients and NCI management.
Date 2024 Apr 16
Language eng
Extra Place: Switzerland
Volume 16
Pages 432-449
Publication Neurology international
DOI 10.3390/neurolint16020032
Issue 2
Journal Abbr Neurol Int
ISSN 2035-8385 2035-8377

PMID 38668129
PMCID PMC11054167
Date Added 6.7.2025, 19:09:33
Modified 5.9.2025, 14:34:11

Notes:

Included - systematic review

sample characteristics

A thorough search of the literature on PubMed/Medline for articles published up to March 2023 using the following keywords: Parkinson, Parkinson's, Parkinson's disease, cognitive impairment, cognition, cognitive decline, mild cognitive impairment, subjective cognitive complaints, social cognition, alexithymia, and theory of mind was conducted. The retrieved publications were then searched for further pertinent references, if applicable. The sources that fulfilled the inclusion criteria were case series and cohort studies as well as systemic reviews, and meta-analyses. The results of systematic reviews and meta-analyses contributed greater validity compared to individual studies. Small sample size studies or case series studies were excluded due to the inherent limitations of a limited population sample and increased heterogeneity among patients as well as the lack of uniformity in assessments used.

**Although they state they have followed PRISMA, it is not available and it is unclear how many papers they have reviewed.

Main findings related to the review's scope

6. Social Cognition and PD

Social cognition (SC) refers to the distinct cognitive and emotional functions that are based on social interactions and not only influence but also determine social behavior [142,143]. It entails the ability to perceive, interpret, and generate responses to the intentions, moods, emotions, and behaviors of other people. SC includes (1) the theory of mind (ToM), which is the ability to understand other people's intentions (cognitive theory of mind) or their emotional state (affective theory of mind) that collectively explain and predict their behavior [144]; (2) empathy, which is the emotional (affective empathy) or cognitive (cognitive empathy) response to the perceived states of others; (3) social perception, which is the ability to understand social and emotional cues, such as body language and facial emotion recognition, which refers to the ability to recognize and distinguish the emotional state of others based on their facial expressions; and (4) social behavior [145,146]. Facial emotion recognition, as well as SC overall, is intricately related to neural cognition and involves neurocognitive functions, including attention and memory.

Aging complicates SC functions [34,147,148]. Despite the general belief that complex rather than basic emotions (joy, sadness, anger, surprise, anxiety, and disgust/disgust) are affected by aging [146], a recent meta-analysis of studies in healthy elderly patients demonstrated a reduced and differential ability to recognize both positive and negative basic emotions, with fewer deficits in emotion recognition for happiness and more deficits in fear recognition (in ascending order happiness, disgust, anger, sadness, surprise, and fear) [149]. This finding is important because the recognition of basic emotions is dictated by different neurocognitive functions [150]. Other factors, such as gender and comorbidities, most notably depression, affect SC and the recognition of facial expressions [147,151]. Regardless of comorbidities, NCI can aggravate affective disorders and SC deficits regardless of comorbid conditions [34,99].

Most of the available studies confirm that PD adversely affects facial emotion recognition, ToM, and empathy [34,147,153,154,155,156]. Compared to healthy controls, PD patients have significant deficits in ToM (both affective and cognitive) and cognitive but not affective empathy, with an emphasis on the recognition of negative versus positive emotions [148,157]. Deficits in the executive function of inhibition are implicated in ToM deficits in PD patients [155]. Clinical parameters implicated in social

perceptual difficulties in PD include dopaminergic treatment and the prevalence of motor symptoms, predominantly on the left side [148]. One of the characteristic motor symptoms of PD, freezing, is related to performance in emotional ToM assessments and anxiety, thus confirming the link between the motor, affective, and neurocognitive symptoms of PD [158,159]. Additionally, transcranial brain stimulation has a beneficial effect on ToM assessments in MCI-PD patients [33].

Similarly, PD patients have reduced, but not absent, or disturbed ability to process facial emotions and experience problems in emotion decoding but not emotion regulation compared to healthy controls [151,154,160]. Since emotion recognition may involve the tendency to reproduce other people's facial expressions, the hypomimic and reduced movement of facial muscles in PD can further complicate facial emotion assessments [154,161]. MCI-PD patients have a statistically significantly reduced ability to recognize facial emotions compared to PD-CN, who in turn were found to have reduced ability compared to healthy volunteers [162]. Additionally, facial emotion recognition was reduced more progressively in patients with SCD, non-amnestic MCI, and amnestic MCI, compared to health volunteers [162]. The contribution of memory deficits to emotion recognition deficits, both facial and vocal, has been consistently reported in PD patients versus healthy volunteers, regardless of depression and visuospatial deficits [153]. Facial emotion recognition is directly linked to visuospatial functions in PD patients, thus highlighting the importance of working, verbal, and visuospatial memory, attention, and verbal fluency, with varying strengths depending on the emotions assessed [154,162,163]. Despite this potential heterogeneity, negative emotion recognition is consistently affected more than positive emotion recognition [153,154,164]. A subsequent study on emotion recognition in non-depressed MCI-PD, PD-CN, and healthy volunteers demonstrated that PD patients had a reduced ability to recognize facial emotions, especially anger, compared to healthy volunteers, and although healthy individuals and PD-CN focused on the mouth and eyes when visually exploring other people's faces, MCI-PD patients tended to focus on the center of the face and spent significantly less time scanning the mouth [164]. PN-CN patients also had a reduced ability to focus compared to healthy volunteers. Therefore, inefficient visual exploration may contribute to the impaired ability to recognize facial emotions in PD, while visual scanning of facial emotions is altered even in the absence of NCI.

Tags: emotion recognition, ToM, behavioral

Neurocognitive Predictors of Understanding of Intentions in Parkinson Disease.

Item Type Journal Article

Author Zuzana Kosutzka
Author Maria Kralova
Author Alice Kusnirova
Author Mariana Papayova
Author Peter Valkovic
Author Zsolt Csefalvay
Author Michal Hajduk

Abstract OBJECTIVES: Theory of Mind (ToM), the ability to understand other people's mental states, is essential in everyday social interactions. The relationship between cognitive domains and ToM impairment in Parkinson disease (PD) has been receiving growing attention with ambiguous findings. The objective of the current study was to ascertain which cognitive domain predicts understanding of intentions and the impact of PD-specific clinical measures on ToM performance. A secondary aim was to evaluate whether cognitive impairment mediates the relationship between severity of illness and ToM impairment. METHODS: Fifty-one nondemented patients with idiopathic PD, ranging from early to advanced stages, were enrolled. A comprehensive neurocognitive battery and 2 ToM tasks (Hinting Task and Comic

Strip Task) were administered during the patients' best "on" medication state. RESULTS: Only the task of measuring working memory capacity was significantly associated with both ToM tasks (Hinting Task Spearman rank correlation [rs] = 0.309, P ≤ .05; Comic Strip Task rs = 0.595, P ≤ .01). Patients with more progressed disease and higher doses of dopaminergic medication performed significantly worse in the Comic Strip Task. Based on the mediation analysis, relationship between the severity of the illness and understanding of intentions was mediated by cognitive flexibility. CONCLUSION: In PD, understanding of intentions is related to neurocognition, with working memory and cognitive flexibility playing a crucial role. The severity of PD predicts ToM performance.

Date 2019 Jul
Language eng
Extra Place: United States
Volume 32
Pages 178-185
Publication Journal of geriatric psychiatry and neurology
DOI 10.1177/0891988719841727
Issue 4
Journal Abbr J Geriatr Psychiatry Neurol
ISSN 0891-9887
PMID 30961413
Date Added 6.7.2025, 19:09:41
Modified 5.9.2025, 14:42:24

Notes:

Not Included: no control group

Tags: EXCLUDED

Neuroeconomic measures of social decision-making across the lifespan

Item Type Journal Article

Author Lusha Zhu
Author Daniel Walsh
Author Ming Hsu

Abstract Social and decision-making deficits are often the first symptoms of a striking number of neurodegenerative disorders associated with aging. These includes not only disorders that directly impact dopamine and basal ganglia, such as Parkinson's disorder, but also degeneration in which multiple neural pathways are affected over the course of normal aging. The impact of such deficits can be dramatic, as in cases of financial fraud, which disproportionately affect the elderly. Unlike memory and motor impairments, however, which are readily recognized as symptoms of more serious underlying neurological conditions, social and decision-making deficits often do not elicit comparable concern in the elderly. Furthermore, few behavioral measures exist to quantify these deficits, due in part to our limited knowledge of the core cognitive components or their neurobiological substrates. Here we probe age-related differences in decision-making using a game theory paradigm previously

shown to dissociate contributions of basal ganglia and prefrontal regions to behavior. Combined with computational modeling, we provide evidence that age-related changes in elderly participants are driven primarily by an over-reliance in trial-and-error reinforcement learning that does not take into account the strategic context, which may underlie cognitive deficits that contribute to social vulnerability in elderly individuals.

Date 2012
Language English
Extra Place: AVENUE DU TRIBUNAL FEDERAL 34, LAUSANNE, CH-1015, SWITZERLAND Type: Article
Volume 6
Publisher FRONTIERS MEDIA SA
Publication FRONTIERS IN NEUROSCIENCE
DOI 10.3389/fnins.2012.00128
Date Added 14.7.2025, 14:50:40
Modified 5.9.2025, 15:03:07

Notes:

Not Included: No PD-group (young vs. elderly)
Tags: EXCLUDED

Neuroimaging Meta-Analyses Reveal Convergence of Interoception, Emotion, and Social Cognition Across Neurodegenerative Diseases.

Item Type Journal Article
Author Jessica L. Hazelton
Author Fábio Carneiro
Author Marcelo Maito
Author Fabian Richter
Author Agustina Legaz
Author Florencia Altschuler
Author Leidy Cubillos-Pinilla
Author Yu Chen
Author Colin P. Doherty
Author Sandra Baez
Author Agustín Ibáñez
Abstract BACKGROUND: Simultaneous interoceptive, emotional, and social cognition deficits are observed across neurodegenerative diseases. Indirect evidence suggests shared neurobiological bases underlying these impairments, termed the allostatic-interoceptive network (AIN). However, no study has yet explored the convergence of these deficits in neurodegenerative diseases or examined how structural and functional changes contribute to cross-domain impairments. METHODS: A Preferred Reporting Items for Systematic Reviews and Meta-Analyses (PRISMA) activated likelihood estimate meta-analysis encompassed studies that met the following inclusion criteria: interoception, emotion, or social cognition tasks; neurodegenerative

diseases (behavioral variant frontotemporal dementia, primary progressive aphasias, Alzheimer's disease, Parkinson's disease, multiple sclerosis); and neuroimaging (structural: magnetic resonance imaging voxel-based morphometry; functional: magnetic resonance imaging and fluorodeoxyglucose-positron emission tomography). RESULTS: Of 20,593 studies, 170 met inclusion criteria (58 interoception, 65 emotion, and 47 social cognition) involving 7032 participants (4963 patients and 2069 healthy control participants). In all participants combined, conjunction analyses revealed AIN involvement of the insula, amygdala, orbitofrontal cortex, anterior cingulate, striatum, thalamus, and hippocampus across domains. In behavioral variant frontotemporal dementia, this conjunction was replicated across domains, with further involvement of the temporal pole, temporal fusiform cortex, and angular gyrus. A convergence of interoception and emotion in the striatum, thalamus, and hippocampus in Parkinson's disease and the posterior insula in primary progressive aphasias was also observed. In Alzheimer's disease and multiple sclerosis, disruptions in the AIN were observed during interoception, but no convergence with emotion was identified. CONCLUSIONS: Neurodegeneration induces dysfunctional AIN across atrophy, connectivity, and metabolism, more accentuated in behavioral variant frontotemporal dementia. Findings bolster the predictive coding theories of large-scale AIN, calling for more synergistic approaches to understanding interoception, emotion, and social cognition impairments in neurodegeneration.

**Date**	2025 Jun 1
**Language**	eng
**License**	Copyright © 2024 Society of Biological Psychiatry. Published by Elsevier Inc. All rights reserved.
**Extra**	Place: United States
**Volume**	97
**Pages**	1079-1090
**Publication**	Biological psychiatry
**DOI**	10.1016/j.biopsych.2024.10.013
**Issue**	11
**Journal Abbr**	Biol Psychiatry
**ISSN**	1873-2402 0006-3223
**PMID**	39442786
**PMCID**	PMC12010404
**Date Added**	6.7.2025, 19:09:34
**Modified**	5.9.2025, 14:37:40

Notes:

Included – Meta-Analysis

A systematic literature review of all neuroimaging studies of interoception, emotion, or social cognition in neurodegenerative diseases was conducted up until August 30, 2023

Search terms resulted in 20,593 publications using PubMed and PsycINFO via Ovid databases.

Inclusion criteria

Emotion. Studies were included in emotion if they measured recognition of emotion in other people or experience of own emotions (e.g., behavioral tasks of emotion recognition, carer rated questionnaires regarding emotionality). Multimodal emotion recognition was considered across modalities (e.g., face, voices, bodies). Emotional experiences included alexithymia (e.g., difficulty describing own emotions) and emotional apathy (e.g., inability to use emotional cues to guide behavior) (see supplementary tables 3-4 for detailed measures).
Social cognition. Studies were included in social cognition if they measured empathy or theory of mind, and general social functioning (e.g., behavioral measures or carer-rated questionnaires) (see supplementary tables 5-6 for detailed measures).

…

+ Emotion (Only): “emotion∗” OR “emotion recognition∗” OR “emotion perception∗” OR “affective∗” OR “affect∗”

+ Social Cognition (Only): “social cognition∗” OR “theory of mind∗” OR “mentalizing∗” OR “mentali∗” OR “empathy∗” OR “empath∗”

Main findings related to the review's scope

2 VBM studies and 8 fMRI on "emotion"

Area	Side	Cluster	Size (mm³)	X	Y	Z	ALE	p	Z
Emotion in PD									
Deactivations/reduced integrity									
Post central gyrus	L	1	400	-44	-26	40	0.01361	0.00001	4.40
Amygdala	R	2	368	16	-6	-14	0.01182	0.00002	4.13
Putamen	L	3	216	-24	-6	10	0.00995	0.00007	3.80
Pallidum	L	3	-	-22	-6	2	0.00892	0.00019	3.55
Anterior Insula	R	4	152	30	18	-8	0.00925	0.00016	3.61
Orbitofrontal cortex	R	4	-	30	22	-12	0.00925	0.00016	3.61
Activations									
Central opercular cortex, post central gyrus	R	1	640	63	-12	15	0.01777	0.00000002	5.47
Frontal pole	R	2	432	6	62	22	0.01187	0.00000535	4.40
Central opercular cortex, post central gyrus	L	3	304	-48	-20	22	0.00961	0.00003552	3.97
Central opercular cortex.	L	3	-	-50	-12	20	0.00938	0.00004625	3.91
Intracalcarine cortex	R	4	152	18	-82	2	0.01003	0.00001980	4.11
Central opercular cortex, pre central gyrus	L	5	152	-54	0	6	0.00998	0.00003104	4.00
Planum temporale, Parietal operculum	L	6	152	-48	-40	18	0.00998	0.00003104	4.00
Post central gyrus	L	7	152	-42	-26	40	0.00998	0.00003104	4.00
Frontal pole	L	8	104	-16	58	22	0.00912	0.00007662	3.79

Note. Clusters are reported at whole-brain voxel-wise uncorrected p <.001, with a cluster extent of 100mm³. Results shown include whole-brain and ROI-l
imaging techniques. AD = Alzheimer's disease, PPA = Primary progressive aphasias, PD = Parkinson's disease, L = Left, R = Right.

Supplementary Table 27. Whole-brain functional and structural coordinates of social cognition in neurodegenerative diseases.

Area	Side	Cluster	Size (mm^3)	X	Y	Z	ALE	*p*	Z
Subcallosal cortex [a]	R	1	1912	10	18	-18	0.03212	0.0000002	5.06
Subcallosal cortex [a]	R	1	-	4	12	-14	0.02759	0.0000028	4.54
Accumbens [a]	R	1	-	10	10	-6	0.02531	0.0000098	4.27
Orbitofrontal cortex [a]	R	1	-	18	20	-20	0.02410	0.0000189	4.12
Orbitofrontal cortex [a]	R	1	-	22	28	-18	0.01992	0.0001626	3.59
Orbitofrontal cortex [a]	L	2	1640	-16	22	-16	0.02991	0.0000007	4.81
Orbitofrontal cortex [a]	L	2	-	-12	22	-22	0.02724	0.0000033	4.50
Amygdala [b]	R	3	1136	26	4	-16	0.02926	0.0000011	4.74
Amygdala, hippocampus [b]	R	3	-	28	-8	-18	0.02125	0.0000828	3.77
Caudate [b]	L	4	832	-8	12	8	0.03448	0.0000001	5.32
Orbitofrontal cortex, Frontal pole [b]	R	5	688	32	28	-20	0.02328	0.0000291	4.02
Orbitofrontal cortex, Anterior Insula [b]	R	5	-	34	22	-12	0.02298	0.0000341	3.98
Posterior Insula [b]	L	6	640	-40	-8	-10	0.02641	0.0000053	4.40
Posterior Insula [b]	L	6	-	-42	-14	-2	0.02142	0.0000760	3.79
Anterior Insula [b]	L	7	584	-36	16	4	0.02681	0.0000043	4.45
Anterior Insula [b]	L	7	-	-38	8	2	0.01693	0.0007469	3.18
Temporal pole [b]	R	8	504	50	14	-30	0.02529	0.0000099	4.27
Temporal pole [b]	R	8	-	58	10	-32	0.02115	0.0000871	3.75
Temporal fusiform cortex [b]	L	9	240	-28	-8	-38	0.02231	0.0000482	3.90
Temporal fusiform cortex [b]	R	10	200	26	-2	-40	0.02334	0.0000282	4.03

Note. Results shown are [a] Cluster-level FWE $p < .05$, 5000 permutations, threshold $p < .001$, and [b] voxel-wise whole-brain uncorrected $p < .001$. R = Right; L = Left

Supplementary Table 26. Whole-brain structural correlates of emotion in neurodegenerative diseases

Area	Side	Cluster	Size (mm^3)	X	Y	Z	ALE	*p*	Z
Inferior temporal gyrus [a]	L	1	1584	-40	-6	-50	0.03062	0.0000001	5.25
Temporal fusiform cortex [a]	L	1	-	-40	-14	-44	0.02118	0.0000219	4.09
Temporal fusiform cortex [a]	L	1	-	-30	-8	-44	0.01905	0.0000715	3.80
Temporal fusiform cortex [a]	R	2	976	38	-10	-46	0.02245	0.0000107	4.25
Inferior temporal gyrus [a]	R	2	-	40	4	-48	0.01933	0.0000611	3.84
Inferior temporal gyrus [a]	R	2	-	44	-4	-48	0.01718	0.0002072	3.53
Amygdala, Hippocampus [a]	L	3	832	-30	-10	-18	0.02529	0.0000020	4.61
Amygdala [a]	L	3	-	-26	-4	-12	0.01839	0.0001036	3.71
Caudate [b]	R	4	720	10	12	8	0.03501	0.0000000	5.74
Temporal pole [b]	L	5	704	-36	6	-40	0.02705	0.0000007	4.83
Caudate [b]	L	6	656	-10	4	10	0.02224	0.0000121	4.22
Thalamus [b]	R	7	552	4	-10	4	0.02844	0.0000003	5.00
Frontal pole [b]	R	8	504	16	56	-12	0.02156	0.0000177	4.14
Frontal pole [b]	R	8	-	22	64	-8	0.01943	0.0000578	3.86
Hippocampus, thalamus [b]	L	9	424	-20	-34	-6	0.02386	0.0000047	4.43
Temporal pole, Anterior Insula [b]	R	10	416	34	8	-20	0.02359	0.0000055	4.40
Posterior Insula [b]	L	11	344	-34	-20	4	0.02208	0.0000132	4.20
Thalamus [b]	R	12	320	14	-10	-2	0.02494	0.0000025	4.57
Middle temporal gyrus [b]	L	13	280	-50	-44	-4	0.01996	0.0000431	3.93
Postcentral gyrus, precentral gyrus [b]	L	14	224	-48	-6	26	0.02042	0.0000334	3.99

Note. Results shown are [a] Cluster-level FWE $p < .05$, 5000 permutations, threshold $p < .001$, and [b] voxel-wise whole-brain uncorrected $p < .001$. R = Right; L = Left.

Regarding our first aim, we observed consistent dysfunction of the allostatic-interoceptive network (AIN) contributing to interoceptive, emotional, and social cognition impairment in cortical predictive regions, such as the orbitofrontal cortex, anterior insula, anterior cingulate cortex, and amygdala (5,13), and subcortical or relay regions, such as the posterior insula, striatum, hippocampus, and thalamus, in neurodegeneration (5,13).

Tags: Emotion recognition, Imaging, G-SC

[Neurological disease and facial recognition].

Item Type Journal Article
Author Mitsuru Kawamura
Author Azusa Sugimoto
Author Mutsutaka Kobayakawa
Author Natsuko Tsuruya

Abstract To discuss the neurological basis of facial recognition, we present our case reports of impaired recognition and a review of previous literature. First, we present a case of infarction and discuss prosopagnosia, which has had a large impact on face recognition research. From a study of patient symptoms, we assume that prosopagnosia may be caused by unilateral right occipitotemporal lesion and right cerebral dominance of facial recognition. Further, circumscribed lesion and degenerative disease may also cause progressive prosopagnosia. Apperceptive prosopagnosia is observed in patients with posterior cortical atrophy (PCA), pathologically considered as Alzheimer's disease, and associative prosopagnosia in frontotemporal lobar degeneration (FTLD). Second, we discuss face recognition as part of communication. Patients with Parkinson disease show social cognitive impairments, such as difficulty in facial expression recognition and deficits in theory of mind as detected by the reading the mind in the eyes test. Pathological and functional imaging studies indicate that social cognitive impairment in Parkinson disease is possibly related to damages in the amygdalae and surrounding limbic system. The social cognitive deficits can be observed in the early stages of Parkinson disease, and even in the prodromal stage, for example, patients with rapid eye movement (REM) sleep behavior disorder (RBD) show impairment in facial expression recognition. Further, patients with myotonic dystrophy type 1 (DM 1), which is a multisystem disease that mainly affects the muscles, show social cognitive impairment similar to that of Parkinson disease. Our previous study showed that facial expression recognition impairment of DM 1 patients is associated with lesion in the amygdalae and insulae. Our study results indicate that behaviors and personality traits in DM 1 patients, which are revealed by social cognitive impairment, are attributable to dysfunction of the limbic system.

Date 2012 Jul
Language jpn
Extra Place: Japan
Volume 64
Pages 799-813
Publication Brain and nerve = Shinkei kenkyu no shinpo
Issue 7
Journal Abbr Brain Nerve
ISSN 1881-6096
PMID 22764352
Date Added 6.7.2025, 19:09:42
Modified 5.9.2025, 14:41:33

Notes:

Not Included: Not in English
Tags: EXCLUDED

Neuropsychological correlates of theory of mind in patients with early Parkinson's disease.

Item Type Journal Article
Author Gabriella Santangelo

Author Carmine Vitale
Author Luigi Trojano
Author Domenico Errico
Author Marianna Amboni
Author Anna Maria Barbarulo
Author Dario Grossi
Author Paolo Barone

Abstract The theory of mind is the ability to attribute mental states to oneself and others and to understand that others have beliefs, desires and intentions different from one's own. The aim of the study was to explore the neuropsychological correlates of theory of mind in patients affected by early Parkinson's disease (PD). Thirty-three PD patients and 33 age-, sex-, and education-matched control subjects underwent the Frontal Assessment Battery, as well as tasks assessing "cognitive" and "affective" theory of mind, and memory abilities; questionnaires evaluating behavioral disorders and quality of life were also administrated. Although the 2 groups did not differ on neuropsychological tasks, PD patients' performance on tasks assessing cognitive and affective theory of mind was significantly worse than controls. Moreover, PD patients had more behavioral disorders and worse quality of life than controls. After covarying for behavioral and quality of life scores, the differences between patients and controls on theory of mind tasks remained significant. "Cognitive" theory of mind was associated with Frontal Assessment Battery score and 2 domains of quality of life scale, whereas "affective" theory of mind scores correlated only with behavioral scales such as the Frontal Behavioral Inventory and Apathy Evaluation Scale. The results demonstrate that both affective and cognitive aspects of theory of mind are simultaneously impaired in early PD and suggest that deficits in the 2 subcomponents of theory of mind may be linked to dysfunction of different frontosubcortical circuitries in early PD.

Date 2012 Jan
Language eng
License Copyright © 2011 Movement Disorder Society.
Extra Place: United States
Volume 27
Pages 98-105
Publication Movement disorders : official journal of the Movement Disorder Society
DOI 10.1002/mds.23949
Issue 1
Journal Abbr Mov Disord
ISSN 1531-8257 0885-3185
PMID 21915910
Date Added 6.7.2025, 19:09:41
Modified 5.9.2025, 14:55:23

Notes:

Included

Sample characteristics

Size: 33 PD, 33 HC (matched for age, education, sex)

PD-type: Idiopathic PD

PD-duration: M = 6.8, SD = 4.7

Medication: LEDD calculated; Testing in ON

Hoehn-Yahr: Range = 1-2, M = 1.7, SD = 0.6

UPDRS-3: M = 19, SD = 36

Gender (male): 22 (66%)

Age: M = 62.7, SD = 10.5

Other neurological disease (tumor, stroke, etc.): NA

Other major psychopathology: None

Origin country (or ethnicity): Italy

method behavioural

instruments used in order to quantify the variables

Social cognition aspect: ToM

Name of the task: Advanced Test of ToM (AT)

Type of stimulus [face/voice etc., Ekman faces/other etc.]: 13 stories describing naturalistic situations in which 2 or more characters interacted with each other as in familiar or social contexts (eg, children arguing about a toy's property, a mother chiding a son for not appreciating food, children pretending to behave as adults)

Task condition: The subject was asked why the characters behaved as they did;

Operationalization: the total score ranges from 0 (worst performance) to 13 (best performance).

Social cognition aspect: ToM

Name of the task: Emotion Attribution Task (EAT)

Type of stimulus [face/voice etc., Ekman faces/other etc.]: 35 short stories describing emotional situations (eg, an employee apprehending to receive an extra salary, a man attacked by a big black spider, a woman finding a worm in her food)

Task condition: the subject was asked what the main protagonists might feel in that situation. Five stories were designed to elicit attribution of sadness, 5 of fear, 5 of embarrassment, 5 of disgust, 5 of happiness, 5 of anger, and 5 of envy

Operationalization: The total score ranges from 0 (worst performance) to 35 (best performance); partial scores for each emotion range from 0 (worst performance) to 5 (best performance).

Main findings related to the review's scope

PD patients performed worse than control subjects on both tasks designed to evaluate ToM abilities

On the EAT, PD patients had more difficulty than controls in attributing all emotions but embarrassment (ie, sadness, fear, disgust, happiness, anger, and envy),

After MANCOVA with covariates:

PD patients and control subjects on tasks assessing the ability of attributing sadness, fear, disgust, anger, and envy to others; the lack of differences between the 2 groups on the ability of attributing embarrassment to others was also confirmed.

Tags: ToM, behavioral

Neuropsychological, neuropsychiatric, and clinical correlates of affective and cognitive theory of mind in Parkinson's disease: A meta-analysis.

Item Type Journal Article
Author Gianpaolo Maggi
Author Amable Manuel Cima Muñoz
Author Ignacio Obeso
Author Gabriella Santangelo
Abstract OBJECTIVE: Theory of mind (ToM) is the ability to infer others' mental (Cognitive) and emotional (Affective) states, both being impaired in Parkinson's disease (PD). However, the clinical, neuropsychological, and neuropsychiatric features underlying Affective and Cognitive ToM deficits in PD are unclear. Therefore, we performed a meta-analytic study to test whether PD demographical, clinical, neuropsychological, or neuropsychiatric changes related differently to both ToM processes. METHOD: A systematic literature search was performed up to January 2022, including a total of 31 studies following our search terms. Data from each study were obtained from demographic (age, education), clinical (disease duration, Hoehn & Yahr staging system, Unified Parkinson's Disease Rating Scale-III, levodopa equivalent daily dose), neuropsychological (global cognitive functioning, memory subdomains, executive functions subdomains, processing speed/complex attention/working memory, visuospatial and constructional abilities, and language), and neuropsychiatric (depression, apathy, anxiety) variables. RESULTS: Affective ToM impairment in PD was related to lower educational level and global cognition, deficits of generativity, decision making, attention/working memory, and language. Conversely, Cognitive ToM deficits were associated with advanced age, poorer global cognition, executive dysfunctions, and language impairments. Medication moderated the relationship between attention/working memory and Cognitive ToM, whereas age moderated the association of Affective ToM with language. No significant associations were found between ToM deficits and patients' neuropsychiatric or clinical states. CONCLUSIONS: These findings clarify the neuropsychological and clinical features that explain ToM deficits in PD. Possibly, our results suggest the need to explore the complex neural networks involving frontostriatal and temporoparietal circuits behind changes in social cognition in PD. (PsycInfo Database Record (c) 2022 APA, all rights reserved).
Date 2022 Sep

Language eng
Extra Place: United States
Volume 36
Pages 483-504
Publication Neuropsychology
DOI 10.1037/neu0000807
Issue 6
Journal Abbr Neuropsychology
ISSN 1931-1559 0894-4105
PMID 35389722
Date Added 6.7.2025, 19:09:40
Modified 5.9.2025, 14:44:25

Notes:

Not Included: no comp. group.

correlates with EF
Tags: EXCLUDED

Neurostructural correlates of harm action/outcome aversion: The role of empathy

Item Type Journal Article

Author Shu Su
Author Ling-Xiang Xia

Abstract Harm aversion is essential for normal human functioning; however, the neuroanatomical mechanisms underlying harm aversion remain unclear. To explore this issue, we examined the brain structures associated with the two distinct dimensions of harm aversion (harm action/outcome aversion) and the potential mediating role of the four aspects of empathy: fantasy, perspective-taking, empathic concern, and personal distress. A sample of 214 healthy young adults underwent structural magnetic resonance imaging. Voxel-based morphometry was used to assess regional gray matter volume (rGMV) and regional gray matter density (rGMD). Whole-brain multiple regression analysis revealed significant correlations between harm action aversion and rGMV/rGMD in various brain regions, including the inferior frontal gyrus (IFG) and precuneus for both rGMV and rGMD, the cerebellum for rGMV, and the superior frontal gyrus for rGMD. The rGMV/rGMD in the IFG and the rGMD in the primary somatosensory cortex (S1) were correlated with harm outcome aversion. Utilizing 10-fold balanced cross-validation analysis, we confirmed the robustness of these significant associations between rGMV/rGMD in these brain regions and harm action/outcome aversion. Importantly, mediation analysis revealed that empathic concern mediated the relationship between rGMV/rGMD in the precuneus and harm action aversion. Additionally, empathic concern, personal distress, and total empathy mediated the relationship between rGMD in the S1 and harm outcome aversion. These findings enhance our understanding of the neural mechanism of harm aversion by integrating insights from the brain structure, harm aversion, and the personality hierarchy models while also extending the frontal

asymmetry model of Emotion

Date 2025
URL https://www.sciencedirect.com/science/article/pii/S1053811924004695
Volume 305
Pages 120972
Publication NeuroImage
DOI https://doi.org/10.1016/j.neuroimage.2024.120972
ISSN 1053-8119
Date Added 6.7.2025, 19:12:36
Modified 5.9.2025, 14:57:45

Notes:

Not Included: No PD group

Tags: EXCLUDED

New insights into facial emotion recognition in Parkinson's disease with and without mild cognitive impairment from visual scanning patterns.

Item Type Journal Article
Author Josefine Waldthaler
Author Charlotte Krüger-Zechlin
Author Lena Stock
Author Zain Deeb
Author Lars Timmermann
Abstract BACKGROUND: Recognizing emotional facial expressions is crucial for social interactions. Cognitive impairment and oculomotor abnormalities are common features of Parkinson's disease (PD) which may contribute to the performance in facial emotion recognition (FER) in PD. OBJECTIVE: The aim of this study was to analyze eye movement behavior during a facial emotion recognition (FER) task with respect to cognitive state in PD patients and healthy controls. METHODS: Eye movements of 24 non-demented, non-depressed PD patients (12 with intact cognitive functions and 12 with Mild Cognitive Impairment (MCI) according to MDS task force criteria level 2), and 12 age-, sex and education-matched healthy controls were recorded during visual exploration of 28 emotional (happiness, surprise, disgust, anger, fear and sadness) and neutral faces. Participants were asked to identify the displayed emotion out of a sevenfold multiple choice question. RESULTS: PD-MCI patients showed reduced FER with specific impairment of anger recognition. Although the scanned area of PD patients with intact cognition was significantly restricted, they did not differ in FER from healthy subjects. While healthy subjects and cognitively intact PD patients scanned faces with preference for mouth and eyes, patients with PD-MCI tended to look at the center of the face and spent significantly less time fixating the mouth. CONCLUSIONS: Ineffective visual exploration may contribute to impaired emotion recognition in PD. Visual scanning of emotional faces is altered in PD even in the absence of cognitive impairment. The progression to PD-MCI may result in further deterioration of scanning behavior and FER impairment.
Date 2019

**Language**	eng
**License**	© 2019 The Authors.
**Extra**	Place: England
**Volume**	1
**Pages**	102-108
**Publication**	Clinical parkinsonism & related disorders
**DOI**	10.1016/j.prdoa.2019.11.003
**Journal Abbr**	Clin Park Relat Disord
**ISSN**	2590-1125
**PMID**	34316611
**PMCID**	PMC8288515
**Date Added**	6.7.2025, 19:09:35
**Modified**	5.9.2025, 15:00:33

Notes:

Included

Sample characteristics

Size: 24 PD (12 with normal cognition, PD-CN; 12 with PD-MCI), 12 HC

PD-type: NA

PD-duration: PD-CN: M = 8.8 SD = 4.4; PD-MCI: M = 7.8, SD = 3.5

Medication: ON state

Hoehn-Yahr: NA

UPDRS-3: PD-CN: M = 25.5, SD = 10.7; PD-MCI: M = 33.6, SD = 13.5

Gender (male): NA

Age: PD-CN: M = 63.4, SD = 9.5; PD-MCI: M = 63.8, SD = 10.2

Other neurological disease (tumor, stroke, etc.): None

Other major psychopathology: None

Origin country (or ethnicity): Germany

method observational

instruments used in order to quantify the variables

Social cognition aspect: Emotion Recognition

Name of the task: NA

Type of stimulus [face/voice etc., Ekman faces/other etc.]: 28 static photographs of emotional faces (half male, half female) from the validated Radboud database.

Task condition: six basic emotions anger, fear, happiness, surprise, sadness and disgust and four neutral faces were presented for 5000 ms.

Operationalization: the participant was asked to choose one emotion out of a sevenfold multiple choice question >> Error rates

Main findings related to the review's scope

ANOVA revealed a significant group effect for the total error rate

multiple comparison resulted in a higher error rate in PD-MCI patients than in healthy controls.

By emotion, PD-MCI patients performed significantly lower than HC and PD-CN patients exclusively in the recognition of anger.

Tags: emotion recognition, behavioral

No Higher Risk-Seeking Tendencies or Altered Self-Estimation in a Social Decision-Making Task in Patients with Parkinson's Disease

Item Type Journal Article
Author Alexandra C. Zapf
Author Ann-Kristin Folkerts
Author Larissa Kahler
Author Alfons Schnitzler
Author Paul Reker
Author Michael T. Barbe
Author Esther Florin
Author Elke Kalbe
Abstract Background: Parkinson's disease (PD) has been associated with a tendency towards more risky decisions. However, the commonly used paradigms typically neglect the social context. Objective: Here, we investigated social decision-making and self-estimation in a competitive experimental task. Methods: A computerized experimental setting was used in which 86 PD patients (age = 66.5 [50-79], 62.8% male, H&Y= 2 [1.5-3]) and 44 healthy controls (HC; age = 67 [54-79], 54.4% male) in groups of four performed mathematical addition tasks in which they were asked to calculate as many sums as possible in five minutes. Participants had to choose their preferred compensation scheme (”piece rate” versus “tournament”) and retrospectively rank their performance in comparison to the suspected performance of the others. A comprehensive neuropsychological test battery was also conducted. Results: No significant difference was found in overall social decision-making and self-estimation between PD patients and HC. However, for those individuals who made inadequate decisions, PD patients engaged in significantly more risk-averse and HC in more risky decisions. Concerning those inadequate decisions, the PD patients made more extreme decisions (severity of social decision-making) in both directions (risk-averse, risk-seeking). Conclusion: Our data indicate that social decision-making behavior and self-estimation are largely intact in PD patients with mild to moderate disease stages and intact global cognition, executive functions, and social cognition. Future studies with more heterogeneous PD samples regarding their neuropsychological profile will have to examine at which state social decision-making may be affected and by which factors this behavior might be influenced.
Date 2022
Language English
Extra Place: NIEUWE HEMWEG 6B, 1013 BG AMSTERDAM, NETHERLANDS Type: Article
Volume 12
Publisher IOS PRESS
Pages 1045-1057
Publication JOURNAL OF PARKINSONS DISEASE
DOI 10.3233/JPD-212960
Issue 3
ISSN 1877-7171
Date Added 14.7.2025, 14:50:30
Modified 5.9.2025, 15:02:53

Notes:

Not Included: not on SC
Tags: EXCLUDED

Non-motor Symptoms and Treatments in Parkinson’s Disease

**Item Type**	Journal Article
**Author**	Jennifer G. Goldman
**Date**	2025
**URL**	https://www.sciencedirect.com/science/article/pii/S0733861924001038
**Volume**	43
**Pages**	291-317
**Publication**	Neurologic Clinics
**DOI**	https://doi.org/10.1016/j.ncl.2024.12.008
**Issue**	2
**ISSN**	0733-8619
**Date Added**	6.7.2025, 19:12:34
**Modified**	5.9.2025, 14:36:50

Notes:

Not Included: not on SC
Tags: EXCLUDED

Non-motor symptoms in Parkinson's disease

**Item Type**	Journal Article
**Author**	Ronald F. Pfeiffer
**Date**	01/2016
**Language**	en
**Library Catalog**	DOI.org (Crossref)
**URL**	https://linkinghub.elsevier.com/retrieve/pii/S135380201500379X
**Accessed**	18.1.2026, 22:55:19
**Volume**	22
**Pages**	S119-S122
**Publication**	Parkinsonism & Related Disorders
**DOI**	10.1016/j.parkreldis.2015.09.004
**Journal Abbr**	Parkinsonism & Related Disorders
**ISSN**	13538020
**Date Added**	18.1.2026, 22:55:19
**Modified**	18.1.2026, 22:55:19

Nonmotor Symptoms in Parkinson Disease: A Descriptive Review on Social Cognition Ability.

**Item Type**	Journal Article
**Author**	Rosanna Palmeri
**Author**	Viviana Lo Buono
**Author**	Francesco Corallo
**Author**	Maria Foti
**Author**	Giuseppe Di Lorenzo
**Author**	Placido Bramanti
**Author**	Silvia Marino
**Abstract**	Parkinson disease (PD) is a neurodegenerative disorder characterized by motor and nonmotor symptoms. Nonmotor symptoms include cognitive deficits and impairment in emotions recognition ability associated with loss of dopaminergic neurons in the substantia nigra and with alteration in frontostriatal circuits. In this review, we analyzed the studies on social cognition ability in patients with PD. We searched on PubMed and Web of Science databases and screening references of included studied and review articles for additional citations. From initial 260 articles, only 18 met search criteria. A total of 496 patients were compared with 514 health controls, through 16 different tests that assessed some subcomponents of social cognition, such as theory of mind, decision-making, and emotional face recognition. Studies on cognitive function in patients with PD have focused on executive function. Patients with PD showed impairment in social cognition from the earliest stages of disease. This ability seems to not be significantly associated with other cognitive functions.
**Date**	2017 Mar
**Language**	eng
**Extra**	Place: United States
**Volume**	30
**Pages**	109-121
**Publication**	Journal of geriatric psychiatry and neurology
**DOI**	10.1177/0891988716687872
**Issue**	2
**Journal Abbr**	J Geriatr Psychiatry Neurol
**ISSN**	0891-9887
**PMID**	28073327
**Date Added**	6.7.2025, 19:09:34
**Modified**	5.9.2025, 14:49:05

Notes:

Not Included: Not a systematic Review

Tags: EXCLUDED

Not all sounds sound the same: Parkinson's disease affects differently emotion processing in music and in speech prosody.

**Item Type**	Journal Article
**Author**	César F. Lima
**Author**	Carolina Garrett
**Author**	São Luís Castro
**Abstract**	Does emotion processing in music and speech prosody recruit common neurocognitive mechanisms? To examine this question, we implemented a cross-domain comparative design in Parkinson's disease (PD). Twenty-four patients and 25 controls performed emotion recognition tasks for music and spoken sentences. In music, patients had impaired recognition of happiness and peacefulness, and intact recognition of sadness and fear; this pattern was independent of general cognitive and perceptual abilities. In speech, patients had a small global impairment, which was significantly mediated by executive dysfunction. Hence, PD affected differently musical and prosodic emotions. This dissociation indicates that the mechanisms underlying the two domains are partly independent.
**Date**	2013
**Language**	eng
**Extra**	Place: England
**Volume**	35
**Pages**	373-392
**Publication**	Journal of clinical and experimental neuropsychology
**DOI**	10.1080/13803395.2013.776518
**Issue**	4
**Journal Abbr**	J Clin Exp Neuropsychol
**ISSN**	1744-411X 1380-3395
**PMID**	23477505
**Date Added**	6.7.2025, 19:09:38
**Modified**	5.9.2025, 14:43:44

Notes:

Included

sample characteristics

size: 24 Idiopathic PD and 25 HC (similar age, education, and musical training)

Parkinson's Disease type and duration: Mduration= 8.3 SD=4.9

Medication: on medication

Hoehn-Yahr: M=1.9 SD=0.5 range=1-3

UPDRS-3: M=17.9 SD=9.1

Gender (male): 17 males (71%)

averaged ages (SD, range): M= 61.8 SD=11.8

other neurological disease (tumor, stroke, etc.): None

other major psychopathology: None

origin country (or ethnicity): Portugal

method observational

instruments used in order to quantify the variables

Social cognition aspect: emotion recognition

Name of the task: NA

Speech stimuli taken from validated database on emotional prosody (Castro & Lima, 2010).

type of stimulus [face/voice etc., Ekman faces/other etc.]: The **speech** stimuli, spoken sentences expressing four emotions

task condition: sadness, fear, happiness, and surprise

Faces from the Karolinska Directed Emotional Faces database (Lundqvist, Flykt, & Öhman, 1998)

type of stimulus [face/voice etc., Ekman faces/other etc.]: **Facial expressions**, Faces were color frontal views of male and female amateur actors with no beards, mous taches, earrings, eyeglasses, or visible make-up.

Task conditions: sadness, fear, happiness, or surprise

operationalization: On each presentation, participants rated on a 7-point scale, from 0 (not at all) to 6 (very much), how much the stimulus expressed one emotion only: sadness, fear, happiness, or peacefulness/surprise.

Main findings related to the review's scope

Speech: correlation with norms: beyond groups, they were higher for happiness (r = .82), surprise (.77),

and sadness (.79, ps > .4), and lower for fear. No other effects.

Categorizations were higher for the intended than for the nonintended emotions of the sentences, with the exception of fear, which was confused with surprise. The patients' categorizations were generally of similar magnitude to that of controls.

The analyses on the distribution of non-intended responses revealed that patients and controls were similar for sadness and fear. For happiness, though, patients responded with surprise less often than controls and for surprise they responded more often with happiness.

Face: Norms. Beyond groups, they were highest for happiness (r = .82, ps < .01), intermediate for sadness (.79) and surprise (.77, p = .07), and lowest for fear. No other effects.

Tags: Emotion recognition, behavioral

Olfaction and apathy in early idiopathic Parkinson's disease

**Item Type**	Journal Article
**Author**	Alfonso E. Martinez-Nunez
**Author**	Kaitie Latack
**Author**	Miguel Situ-Kcomt
**Author**	Abhimanyu Mahajan
**Abstract**	Background Apathy remains a disabling symptom in Parkinson's disease (PD) with limited therapeutic success. Processing of emotions and smell share neuroanatomical and evolutionary pathways. Objectives To explore the association of apathy with smell dysfunction (SD) in early PD. Methods We analyzed patients with de-novo PD, with follow-up of at least 5 years from the Parkinson's Progression Markers Initiative. SD and apathy were defined using University of Pennsylvania Smell Identification Test and MDS-UPDRS part 1A. Odds ratios were calculated between apathy and olfaction groups. Kaplan-Meier survival analysis was grouped by presence/ absence of smell dysfunction. The Log Rank test was used to compare time to apathy. Results We found no association between presence of apathy in patients with and without SD (OR 1.01 [0.49–2.08]). There was no significant difference between PD patients with and without SD in time to apathy (p = 0.72). Conclusions SD does not portend greater risk of apathy in PD.
**Date**	2022
**URL**	https://www.sciencedirect.com/science/article/pii/S0022510X22001769
**Volume**	439
**Pages**	120314
**Publication**	Journal of the Neurological Sciences
**DOI**	https://doi.org/10.1016/j.jns.2022.120314
**ISSN**	0022-510X
**Date Added**	6.7.2025, 19:12:36
**Modified**	5.9.2025, 14:45:19

Notes:

Not included: Doesn’t study SC
Tags: EXCLUDED

On the analysis of EEG power, frequency and asymmetry in Parkinson's disease during emotion processing.

**Item Type**	Journal Article
**Author**	Rajamanickam Yuvaraj
**Author**	Murugappan Murugappan
**Author**	Norlinah Mohamed Ibrahim
**Author**	Mohd Iqbal
**Author**	Kenneth Sundaraj
**Author**	Khairiyah Mohamad
**Author**	Ramaswamy Palaniappan
**Author**	Edgar Mesquita
**Author**	Marimuthu Satiyan
**Abstract**	OBJECTIVE: While Parkinson's disease (PD) has traditionally been described as a movement disorder, there is growing evidence of disruption in emotion information processing associated with the disease. The aim of this study was to investigate whether there are specific electroencephalographic (EEG) characteristics that discriminate PD patients and normal controls during emotion information processing. METHOD: EEG recordings from 14 scalp sites were collected from 20 PD patients and 30 age-matched normal controls. Multimodal (audio-visual) stimuli were presented to evoke specific targeted emotional states such as happiness, sadness, fear, anger, surprise and disgust. Absolute and relative power, frequency and asymmetry measures derived from spectrally analyzed EEGs were subjected to repeated ANOVA measures for group comparisons as well as to discriminate function analysis to examine their utility as classification indices. In addition, subjective ratings were obtained for the used emotional stimuli. RESULTS: Behaviorally, PD patients showed no impairments in emotion recognition as measured by subjective ratings. Compared with normal controls, PD patients evidenced smaller overall relative delta, theta, alpha and beta power, and at bilateral anterior regions smaller absolute theta, alpha, and beta power and higher mean total spectrum frequency across different emotional states. Inter-hemispheric theta, alpha, and beta power asymmetry index differences were noted, with controls exhibiting greater right than left hemisphere activation. Whereas intra-hemispheric alpha power asymmetry reduction was exhibited in patients bilaterally at all regions. Discriminant analysis correctly classified 95.0% of the patients and controls during emotional stimuli. CONCLUSION: These distributed spectral powers in different frequency bands might provide meaningful information about emotional processing in PD patients.
**Date**	2014 Apr 9
**Language**	eng
**Extra**	Place: England
**Volume**	10
**Pages**	12
**Publication**	Behavioral and brain functions : BBF
**DOI**	10.1186/1744-9081-10-12

Journal Abbr Behav Brain Funct
ISSN 1744-9081
PMID 24716619
PMCID PMC4234023
Date Added 6.7.2025, 19:09:37
Modified 5.9.2025, 15:02:49

Notes:

Not Included: not on SC
Tags: EXCLUDED

On the integrity of functional brain networks in schizophrenia, Parkinson's disease, and advanced age: Evidence from connectivity-based single-subject classification.

Item Type Journal Article
Author Rachel N. Pläschke
Author Edna C. Cieslik
Author Veronika I. Müller
Author Felix Hoffstaedter
Author Anna Plachti
Author Deepthi P. Varikuti
Author Mareike Goosses
Author Anne Latz
Author Svenja Caspers
Author Christiane Jockwitz
Author Susanne Moebus
Author Oliver Gruber
Author Claudia R. Eickhoff
Author Kathrin Reetz
Author Julia Heller
Author Martin Südmeyer
Author Christian Mathys
Author Julian Caspers
Author Christian Grefkes
Author Tobias Kalenscher
Author Robert Langner
Author Simon B. Eickhoff
Abstract Previous whole-brain functional connectivity studies achieved successful classifications of patients and healthy controls but only offered limited specificity as to affected brain systems. Here, we examined whether the connectivity patterns of functional systems affected in schizophrenia (SCZ), Parkinson's disease (PD), or normal aging equally translate into high classification accuracies for these conditions.

We compared classification performance between pre-defined networks for each group and, for any given network, between groups. Separate support vector machine classifications of 86 SCZ patients, 80 PD patients, and 95 older adults relative to their matched healthy/young controls, respectively, were performed on functional connectivity in 12 task-based, meta-analytically defined networks using 25 replications of a nested 10-fold cross-validation scheme. Classification performance of the various networks clearly differed between conditions, as those networks that best classified one disease were usually non-informative for the other. For SCZ, but not PD, emotion-processing, empathy, and cognitive action control networks distinguished patients most accurately from controls. For PD, but not SCZ, networks subserving autobiographical or semantic memory, motor execution, and theory-of-mind cognition yielded the best classifications. In contrast, young-old classification was excellent based on all networks and outperformed both clinical classifications. Our pattern-classification approach captured associations between clinical and developmental conditions and functional network integrity with a higher level of specificity than did previous whole-brain analyses. Taken together, our results support resting-state connectivity as a marker of functional dysregulation in specific networks known to be affected by SCZ and PD, while suggesting that aging affects network integrity in a more global way. Hum Brain Mapp 38:5845-5858, 2017. © 2017 Wiley Periodicals, Inc.

Date 2017 Dec
Language eng
License © 2017 Wiley Periodicals, Inc.
Extra Place: United States
Volume 38
Pages 5845-5858
Publication Human brain mapping
DOI 10.1002/hbm.23763
Issue 12
Journal Abbr Hum Brain Mapp
ISSN 1097-0193 1065-9471
PMID 28876500
PMCID PMC5931403
Date Added 6.7.2025, 19:09:42
Modified 5.9.2025, 14:51:24

Notes:

Included

Sample characteristics

Size: 80 PD, 84 HC (matched for gender, whinin-scanner movement and age)

PD-type: NA

PD-duration: M = 7.2 SD = 5.3

Medication: ON state

Hoehn-Yahr: NA

UPDRS-3: NA

Gender (male): NA

Age: M = 59 SD = 9

Other neurological disease (tumor, stroke, etc.): NA nor PD; none for HC

Other major psychopathology: PD patients hav on average a MCI but not depression symptoms

Origin country (or ethnicity): Germany

method behavioural

instruments used in order to quantify the variables

No SC-task.

Only fMRI:

They aimed to investigate whether **resting-state functional connectivity (RSFC) patterns** of specific brain networks can reliably predict a participant's group membership: Parkinson's disease (PD) versus healthy controls, schizophrenia (SCZ) versus healthy controls, or older versus younger adults.

To achieve this, they:

1. **RS fMRI data acquisition and preprocessing** – Participants lay still with eyes closed, letting their mind wander. Data were processed using SPM8 (motion correction, normalization to the MNI-152 template, smoothing).
2. **Network selection** – They included 12 functional networks defined from prior meta-analyses, each containing at least 10 nodes.
3. **RSFC computation** – Time series were extracted for each node, nuisance signals (motion parameters, WM, CSF) were regressed out, high-pass filtering was applied, and Pearson correlations between all pairs of nodes in each network were computed and Fisher Z-transformed.
4. **SVM classification** – Each network was analyzed separately using a linear, non-sparse two-class SVM to test whether RSFC patterns could predict group membership:
   - Input features: edge-wise RSFC between all nodes in a network.
   - Training/testing: nested 10-fold cross-validation.
   - Performance metrics: accuracy, balanced accuracy, sensitivity, specificity, AUC, d'.
   - Procedure repeated 25 times to ensure stability of results.
5. **Comparison across networks and groups** –
   - Pairwise t-tests between networks to determine which network classified best within each group.
   - Comparisons between SCZ and PD after standardizing accuracies to z-scores.
   - Log-likelihood ratios to identify networks better at distinguishing one patient group from another.

Main findings related to the review's scope

For PD, the networks subserving autobiographical memory (AM; Acc. = 75%; AUC = 0.76), motor execution (Motor; Acc. = 70%; AUC = 0.77), semantic memory (SM; Acc. = 69%; AUC = 0.75), and **theory-of-mind cognition (ToM; Acc. = 67%; AUC = 0.71**) yielded the highest classification accuracies, that is, contained the most informative PD-related differences in RSFC. The AM network was significantly better in the PD classification compared to all other networks ($p < 0.001$).

Both EmoSF and Empathy networks showed the best performance at distinguishing SCZ patients from HCs (EmoSF: z = 5.9; Empathy: z = 5.5) but were **notably worse at discriminating PD patients from their HCs (EmoSF: z = 3.2; Empathy: z = 3.2).**

AM and SM networks achieved high accuracies in classifying PD patients and controls (AM: z = 6.3; SM: z = 4.5)

Tags: ToM, Imaging

Orbital and ventromedial prefrontal cortex functioning in Parkinson's disease: neuropsychological evidence.

Item Type Journal Article

Author Michele Poletti

Author Ubaldo Bonuccelli

Abstract A recent paper (Zald & Andreotti, 2010) reviewed neuropsychological tasks that assess the function of the orbital and ventromedial portions of the prefrontal cortex (OMPFC). Neuropathological studies have shown that the function of the OMPFC should be preserved in the early stages of Parkinson's disease (PD) but becomes affected in the advanced stages of PD. This pattern has also been suggested by studies that have shown that dopaminergic drugs impair the performance of early PD patients

in OMPFC tasks that involve reinforcement learning but enhance the performance of advanced PD patients. Based on these empirical findings, we reviewed the neuropsychological evidence of OMPFC functions in PD patients to test two hypotheses regarding the following: (1) OMPFC functions at different stages of PD; (2) different effects of dopaminergic drugs on OMPFC functions based on PD stage and task demand. We focused our review only on the neuropsychological tasks that were specific and sensitive to the functions of the OMPFC and that were adopted at different stages of PD, such as reversal learning tasks, the Iowa Gambling Task and the affective Theory of Mind task. We found robust empirical evidence that in early PD, OMPFC functions are preserved and dopaminergic drugs result in a detrimental effect when the task involves reinforcement learning. Further studies are needed to verify the status of OMPFC functions in non-demented, advanced PD and to describe the longitudinal course of OMPFC functions in this clinical population.

**Date**	2012 Jun
**Language**	eng
**License**	© 2012 Elsevier Inc. All rights reserved.
**Extra**	Place: United States
**Volume**	79
**Pages**	23-33
**Publication**	Brain and cognition
**DOI**	10.1016/j.bandc.2012.02.002
**Issue**	1
**Journal Abbr**	Brain Cogn
**ISSN**	1090-2147 0278-2626
**PMID**	22387277
**Date Added**	6.7.2025, 19:09:41
**Modified**	5.9.2025, 14:51:44

Notes:

Not Included: Not a Systematic Review (Literature Review)

Tags: Excluded

Parameters of emotional processing in neuropsychiatric disorders: Conceptual issues and a battery of tests

**Item Type**	Journal Article
**Author**	Joan C. Borod
**Author**	Joan Welkowitz
**Author**	Murray Alpert
**Author**	Alizah Z. Brozgold
**Author**	Candace Martin
**Author**	Eric Peselow
**Author**	Leonard Diller
**Date**	8/1990
**Language**	en

**Short Title**	Parameters of emotional processing in neuropsychiatric disorders
**Library Catalog**	DOI.org (Crossref)
**URL**	https://linkinghub.elsevier.com/retrieve/pii/002199249090003H
**Accessed**	11.8.2025, 17:49:58
**License**	https://www.elsevier.com/tdm/userlicense/1.0/
**Volume**	23
**Pages**	247-271
**Publication**	Journal of Communication Disorders
**DOI**	10.1016/0021-9924(90)90003-H
**Issue**	4-5
**Journal Abbr**	Journal of Communication Disorders
**ISSN**	00219924
**Date Added**	11.8.2025, 17:49:58
**Modified**	11.8.2025, 17:49:58

Notes:

Included

sample characteristics

size: 20 PD, 20 schizophrenics (SZs), 12 unipoler depressives (UDs), 19 right-brain-damaged (RBDs), and 21 HC

Parkinson's Disease type and duration: idiopathic, NA

Medication: NA

Hoehn-Yahr: NA

UPDRS-3: NA

Gender (male): 12 males (60%)

averaged ages (SD, range): M= 65.7 SD= 8.0

other neurological disease (tumor, stroke, etc.): None

other major psychopathology: None

origin country (or ethnicity): NA

method observational

instruments used in order to quantify the variables

Social cognition aspect: emotion recognition

Name of the task: NA

type of stimulus [face/voice etc., Ekman faces/other etc.]: Ekman, six males, six females).

task conditions: happiness, surprise, sadness, fear, anger, disgust, neutral

operationalization: discrimination, identification. >> accuracy

Name of the task: NA

type of stimulus [face/voice etc., Ekman faces/other etc.]: These tasks were developed by Tucker et al. (1977); minor modifications have been made in the response format. Stimuli consisted of four sentences of neutral content, e.g., "Fish can jump out of the water," spoken by a male poser in one of three emotional tones and in an indifferen tone.

task conditions: happiness, sadness, anger, neutral

operationalization: discrimination, identification. >> accuracy

Main findings related to the review's scope

No differences between PD and HC in face-discrimination, face-identification, or voice-discrimination.

PD were less accurate then HC in voice-identification.

SZ were less accurate then PD in face-discrimination.

PD were similar in all tasks to UD and RBD.
Tags: Emotion recognition, behavioral

Parkinson disease

**Item Type**	Journal Article
**Author**	Werner Poewe
**Author**	Klaus Seppi
**Author**	Caroline M. Tanner
**Author**	Glenda M. Halliday
**Author**	Patrik Brundin
**Author**	Jens Volkmann
**Author**	Anette-Eleonore Schrag
**Author**	Anthony E. Lang
**Date**	2017-03-23
**Language**	en
**Library Catalog**	DOI.org (Crossref)
**URL**	https://www.nature.com/articles/nrdp201713
**Accessed**	18.1.2026, 22:52:46
**Volume**	3
**Pages**	17013
**Publication**	Nature Reviews Disease Primers
**DOI**	10.1038/nrdp.2017.13
**Issue**	1
**Journal Abbr**	Nat Rev Dis Primers
**ISSN**	2056-676X
**Date Added**	18.1.2026, 22:52:46
**Modified**	18.1.2026, 22:52:46

Parkinson disease patients' performance in Theory of Mind (ToM) and decision-making tasks with and without Deep Brain Stimulation (DBS)

**Item Type**	Journal Article
**Author**	Laura Orduz-Bastidas
**Author**	Adriana Martinez-Martinez
**Author**	Camilo Hurtado-Parrado
**Author**	Wilson Lopez-Lopez
**Author**	Cesar Acevedo-Triana

Abstract Background: Patients with Parkinson's Disease (PD) show non-motor symptoms, such as cognitive impairment, disrupting executive functions, and mood alterations. Two processes currently researched in these areas are Theory of Mind (ToM) and decision-making in PD patients. ToM is the ability to identify mental states (affective or cognitive) in others, and it is a necessary skill for successful communication in social situations. Decision-making is researched in PD patients due to alterations in dopaminergic pathways involved in cortico-striatal circuits. These pathways have been linked to cognitive functions. Both processes (ToM and decision making) could be altered in PD patients after deep brain stimulation (DBS) therapy. Objective: To compare the performance of PD patients (with and without DBS) and healthy controls (HC) in Theory of Mind and decision-making tasks. Methods: We implemented in three groups of patients (PD, n = 4; PD-DBS, n = 5 and HC, n = 5) the Yoni task to identify affective and cognitive features in ToM, and Iowa Gambling Task (IGT) to assess decision-making. Results: There were no differences across the PD groups in ToM, both in the affective and cognitive features. Regarding decision-making (IGT scores), we obtained results consistent with previous findings, with PD patients showing impairments in this process. Conclusions: Some results suggest that DBS therapy affected PD patients' decision-making performance when compared to healthy controls. Our results describe some non-motor changes related to DBS often seen in PD patients.

Date 2020-08
Language English
Extra Place: RICARDO MATTE PEREZ 492 PROVIDENCIA, SANTIAGO, 00000, CHILE Type: Article
Volume 38
Publisher SOCIEDAD CHILENA PSICOLOGIA CLINICA
Pages 259-282
Publication TERAPIA PSICOLOGICA
DOI 10.4067/S0718-48082020000200259
Issue 2
ISSN 0718-4808
Date Added 14.7.2025, 14:50:31
Modified 5.9.2025, 14:48:11

Notes:

Included

Sample characteristics

Size: 4 PD, 5 PD-DBS, 5 HC (matched for age and education)

PD-type: NA

PD-duration: PD: M = 6.5, SD 0 2.38, PD-DBS: 13.75, SD = 7.68

Medication: Received dopaminergic precursor medication

Hoehn-Yahr: NA

UPDRS-3: NA

Gender (male): PD: 4 (100%), PD-DBS: 4 (80%)

Age: PD: M = 55.7, SD = 14.5), PD-DBS: M = 62.2, SD = 4.8

Other neurological disease (tumor, stroke, etc.): None

Other major psychopathology: None

Origin country (or ethnicity): Colombian

method behavioural

instruments used in order to quantify the variables

Social cognition aspect: ToM

Name of the task: Yoni task

Type of stimulus [face/voice etc., Ekman faces/other etc.]: A cartoon outline of a face (named "Yoni") is presented briefly in the center of the screen, accompanied by four options in order to respond to a verbal instruction located at the top of the screen

Task condition: 98 trials divided into three blocks

Operationalization: Correct answers

Main findings related to the review's scope

ToM

There were no statistically significant differences comparing the three groups.

However, visual inspection of Figure 3 shows that the HC group has higher scores in the majority ToM components, i.e., affective (first and second level), cognitive (first and second level), combined affective and cognitive, social emotions recognition (envy and schadenfreude), and control trials.

To test if the tendency for differences in the Yoni Task was consistent, we split the group of subjects using the Median score – "below the median" and "above the median" - and compared performance in the Identification variable. Phi and Cramer's V test showed that the PD group was significantly lower than PD-DBS and HC (Phi = .86; p = .008).

Tags: ToM, behavioral

Parkinson disease-associated cognitive impairment

Item Type Journal Article
Author Dag Aarsland
Author Lucia Batzu
Author Glenda M. Halliday
Author Gert J. Geurtsen
Author Clive Ballard
Author K. Ray Chaudhuri
Author Daniel Weintraub
Date 2021-07-01
Language en
Library Catalog DOI.org (Crossref)
URL https://www.nature.com/articles/s41572-021-00280-3
Accessed 18.1.2026, 22:55:53
Volume 7
Pages 47
Publication Nature Reviews Disease Primers
DOI 10.1038/s41572-021-00280-3
Issue 1
Journal Abbr Nat Rev Dis Primers
ISSN 2056-676X
Date Added 18.1.2026, 22:55:53
Modified 18.1.2026, 22:55:53

Parkinson's disease

Item Type Journal Article
Author Bastiaan R Bloem
Author Michael S Okun
Author Christine Klein
Date 06/2021
Language en
Library Catalog DOI.org (Crossref)
URL https://linkinghub.elsevier.com/retrieve/pii/S014067362100218X
Accessed 18.1.2026, 22:54:01
Volume 397
Pages 2284-2303
Publication The Lancet
DOI 10.1016/S0140-6736(21)00218-X
Issue 10291
Journal Abbr The Lancet
ISSN 01406736

Date Added 18.1.2026, 22:54:01
Modified 18.1.2026, 22:54:01

Parkinson's Disease Without Dementia

Item Type Book Section
Author Thomas Holtgraves
Author Magda Giordano
Editor L Cummings
Abstract Although viewed primarily as a motor disorder, Parkinson's disease (PD) is also associated with a variety of communication and cognitive deficits. In this chapter, we review research on pragmatic deficits in PD, as well as related cognitive processes that can contribute to those deficits. A variety of comprehension deficits have been demonstrated in PD, including deficits in the speed and accuracy with which non-literal meanings and speech acts are recognized, as well as an impaired ability to recognize emotions. These deficits overlap somewhat with various executive functions (e.g. working memory) and theory of mind abilities. Individuals with PD are also impaired in terms of language production, possibly in part because of their comprehension deficits. Major production deficits include reduced informational content, longer and more frequent pauses and associated turn-taking disruption, inappropriate levels of politeness, and deficits in various nonverbal accompaniments. The extent to which these production deficits are associated with general cognitive decline remains somewhat unclear. There is evidence that the severity of pragmatic deficits in PD is associated with greater disease severity, and that dopaminergic medication can reduce some of these deficits.
Date 2017
Language English
Extra Type: Article; Book Chapter
Volume 11
Place GEWERBESTRASSE 11, CHAM, CH-6330, SWITZERLAND
Publisher SPRINGER INTERNATIONAL PUBLISHING AG
ISBN 978-3-319-47489-2 978-3-319-47487-8
Pages 379-407
Series Perspectives in Pragmatics Philosophy and Psychology
Book Title RESEARCH IN CLINICAL PRAGMATICS
DOI 10.1007/978-3-319-47489-2_15
ISSN 2214-3807
Date Added 14.7.2025, 14:50:35
Modified 5.9.2025, 14:39:31

Notes:

Not Included: a book chapter.

Tags: EXCLUDED

Parkinson's disease: The psychological aspects of a chronic illness.

Item Type Journal Article
Author Gayle A. Dakof
Author Gerald A. Mendelsohn
Date 1986
Language en
Short Title Parkinson's disease
Library Catalog Crossref
URL https://doi.apa.org/doi/10.1037/0033-2909.99.3.375
Accessed 13.7.2025, 20:21:10
Volume 99
Publisher American Psychological Association (APA)
Pages 375-387
Publication Psychological Bulletin
DOI 10.1037/0033-2909.99.3.375
Issue 3
ISSN 1939-1455, 0033-2909
Date Added 13.7.2025, 20:21:10
Modified 13.7.2025, 20:21:10

Notes:

Not Included: not on SC
Tags: EXCLUDED

Parkinsonian patients with deficits in the dysexecutive spectrum are impaired on theory of mind tasks.

Item Type Journal Article
Author Alberto Costa
Author Antonella Peppe
Author Matteo Martini
Author Katia Coletta
Author Massimiliano Oliveri
Author Carlo Caltagirone
Author Giovanni A. Carlesimo
Abstract Understanding the mental states of others entails a number of cognitive processes known as Theory of Mind (ToM). A relationship between ToM deficits and executive disorders has been hypothesized in individuals with Parkinson's disease (PD). The present study was aimed at investigating the effect of dysexecutive deficits on ToM abilities in PD patients without dementia. Participants included 30 PD patients and 30 healthy subjects (HC). PD patients were divided into two groups according to their executive test performance: patients with poor (dysexecutive group; n=15) and

normal (executively unimpaired group; n=15) performance. All participants were administered faux pas recognition written stories. The dysexecutive PD patients performed less accurately than both HC and executively unimpaired PD individuals on all faux pas story questions ($p < 0.05$); the executively unimpaired PD group performed as accurately as the HC group on the ToM tasks. Results of the study clearly demonstrate that PD is not tout court associated with ToM impairments and that these may occur in PD patients as a function of the degree of their executive impairment. Our findings also indirectly confirm previous data on the role of the prefrontal regions in mediating ToM capacities.

Date 2013 Jan 1
Language eng
Extra Place: Netherlands
Volume 27
Pages 523-533
Publication Behavioural neurology
DOI 10.3233/BEN-129018
Issue 4
Journal Abbr Behav Neurol
ISSN 1875-8584 0953-4180
PMID 23242360
PMCID PMC5214465
Date Added 6.7.2025, 19:09:41
Modified 5.9.2025, 14:31:35

Notes:

Included

sample characteristics

size: 30 PD (devided into two groups) and 30 HC

Parkinson's Disease type and duration: idiopathic PD, Mduration=NA

Medication: on medication

Hoehn-Yahr: NA

UPDRS-3: M=28.7 SD=12.85

Gender (male): 21 males (70%)

averaged ages (SD, range): M= 62.45 SD=6.01

other neurological disease (tumor, stroke, etc.): None

other major psychopathology: None

origin country (or ethnicity): NA

method observational

instruments used in order to quantify the variables

Social cognition aspect: ToM

Name of the task: Faux Pas task

type of stimulus [face/voice etc., Ekman faces/other etc.]: this procedure was constructed basing on previous ToM paradigms. We composed 12 written short stories followed by a series of questions. The stories were composed of six Recognition of Faux Pas tasks and six control stories. In faux pas stories a prior event that occurred between two people is described. Subsequently, the two characters meet again and one, forgetting the prior encounter, says something awkward, which could offend the other person. The questions require the subject to detect the faux pas (question 1: "Did someone say something they shouldn't have said?"), to understand the mental state of the listener (question 2: "Why shouldn't they have said it?") and to understand the mental state of the speaker (question 3: "Why did they say it?"),choosing between different alternatives by pressing the corresponding button on the keyboard.

operationalization: RT and Accuracy

PD the patients were split into two subgroups according to the following parameters: 1) a pathological score corresponding to a performance < 95% of the lower tolerance limit of the normal population distribution on the Modified Card Sorting test (MCST; see below for a detailed description of the this test [33]); ii) 2) a score below the median of the whole PD group on at least two of the other tests individually examining the following executive subcomponents

Main findings related to the review's scope

The dysexecutive PD patients performed less accurately than both HC and executively unimpaired PD

individuals on all faux pas story questions ($p < 0.05$); the executively unimpaired PD group performed as accurately as the HC group on the ToM tasks.

the dy-sexecutive PD individuals were significantly less accurate than both HC and executively unimpaired PD subjects. Moreover, allgroups obtained lower average scores on the third ques-tion than on the first and second questions, respectively.

Faux paus - the composite score was significantlylower in the dysexecutive PD group compared with both HC and the executivelyunimpaired PD group. No difference between unimpaired PD group and HC.

No differences in RT between groups, in general.

Tags: ToM, behavioral

Patients with Parkinson's disease display a dopamine therapy related negative bias and an enlarged range in emotional responses to facial emotional stimuli.

**Item Type**	Journal Article
**Author**	Daniel Lundqvist
**Author**	Joakim Svärd
**Author**	Åsa Michelgård Palmquist
**Author**	Håkan Fischer
**Author**	Per Svenningsson
**Abstract**	OBJECTIVE: The literature on emotional processing in Parkinson's disease (PD) patients shows mixed results. This may be because of various methodological and/or patient-related differences, such as failing to adjust for cognitive functioning, depression, and/or mood. METHOD: In the current study, we tested PD patients and healthy controls (HCs) using emotional stimuli across a variety of tasks, including visual search, short-term memory (STM), categorical perception, and emotional stimulus rating. The PD and HC groups were matched on cognitive ability, depression, and mood. We also explored possible relationships between task results and antiparkinsonian treatment effects, as measured by levodopa equivalent dosages (LED), in the PD group. RESULTS: The results show that PD patients use a larger emotional range compared with HCs when reporting their impression of emotional faces on rated emotional valence, arousal, and potency. The results also show that dopaminergic therapy was correlated with stimulus rating results such that PD patients with higher LED scores rated negative faces as less arousing, less negative, and less powerful. Finally, results also show that PD patients display a general slowing effect in the visual search tasks compared with HCs, indicating overall slowed responses. There were no group differences observed in the STM or categorical perception tasks. CONCLUSIONS: Our results indicate a relationship between emotional responses, PD, and dopaminergic therapy, in which PD per se is associated with stronger emotional responses, whereas LED levels are negatively correlated with the strength of emotional responses. (PsycINFO Database Record
**Date**	2017 Sep
**Language**	eng

License (c) 2017 APA, all rights reserved).
Extra Place: United States
Volume 31
Pages 605-612
Publication Neuropsychology
DOI 10.1037/neu0000371
Issue 6
Journal Abbr Neuropsychology
ISSN 1931-1559 0894-4105
PMID 28581310
Date Added 6.7.2025, 19:09:39
Modified 5.9.2025, 14:44:04

Notes:

Included

sample characteristics

size: 23 PD and 23 matched HC (by sex and age)

Parkinson's Disease type and duration: NA

Medication: on state

Hoehn-Yahr: M= 2.1 SD=.7

UPDRS-3: M= 37.0 SD=16.1

Gender (male): 14 males (61%)

averaged ages (SD, range): M= 70.6 SD=6.2

other neurological disease (tumor, stroke, etc.): None

other major psychopathology: None

origin country (or ethnicity): NA

method observational

instruments used in order to quantify the variables

Social cognition aspect: emotion recognition

Name of the task: NA

type of stimulus [face/voice etc., Ekman faces/other etc.]:

Three tasks: visual search, categorical perception, Visual Short-Term Memory, and rating task.

Averaged Karolinska Directed Emotional Faces (AKDEF) set - both an averaged male and averaged female face. Modified versions of the angry and happy faces were used. The modification (using SqirlsMorph 2.1; http://www.xiberpix.net/) consisted of morphing emotional expressions at intensities between 0 (neutral) and 100% (happy or angry, respectively) in steps of 10%.

In the emotional rating and visual search tasks, the previously described averaged faces displaying angry, happy, and neutral expressions, as well as faces displaying fear, disgust, sadness, and surprise, were used as stimuli. In these tasks, the emotions were fully expressed (at 100% intensity).

Visual search task. The visual search task contained a circular display of six faces. All faces showed identical expressions in half of the 288 trials (so-called target-absent trials). In the other 144 trials, one facial expression deviated from the other faces in the array (target-present trials). During target-absent trials, all faces were angry, afraid, sad, neutral, happy, disgusted, or surprised. During the target-present trials, there was either an emotional face among neutral faces, or a neutral target face among emotional distractors. The target face appeared once at each position in the array. Participants were instructed to indicate whether one face was different from the other with button presses using their right index finger, or whether the faces were all the same with button presses using their left index finger.

Operationalization: RT and accuracy for the target-present trials with emotional (angry and happy) targets.

Categorical perception task. This task contained pairs of faces, presented side by side. The faces in each pair differed 20% in intensity, ranging from 0% to 20% pairs up to 80% to 100% pairs (see Figure 1, bottom panel). Thus, nine pairs were presented for each of the expressions of anger and happiness, separately for the female and male faces.

Operationalization: A peak intensity interval (e.g., 40%–60%) was identified for each participant for the angry and happy expressions separately by counting the number of times the participant reported that the expression differed in each interval.

Visual STM task. In this task, a morphed face that expressed an emotion with an intensity between 10 % and 90 % (in 10 % intervals) was presented for 3 s. After an interval of 1 s, the same face reappeared with a new randomly assigned emotional intensity. Participants were instructed to memorize the first face, and then upon the display of the second face, to visually adjust the face's intensity so it matched the memory of the first displayed face. As for the other tasks, this task also contained four separate blocks (female, neutral to angry; female, neutral to happy; male, neutral to angry; male, neutral to happy).

Operationalization: Squere difference between the actual intensity and the intensity they have rated.

Rating task. A total of 14 faces (all seven expressions in the two genders) with full emotional expression (100% intensity) were presented to the participants. The faces were presented one at a time.

Operationalization: rated (ranged from -1 to 1 in steps of .001) on the emotional dimensions of Arousal (active passive), Valence (pleasant–unpleasant), and Potency (weak strong).

Conditions: emotions.

Main findings related to the review's scope

Visual Search Task: no group differences in accuracy. PD patients generated longer RTs compared with HCs. Beyond groups, angry and sad faces were less accurately detected and with slower RTs than all other expression.

Categorical Perception Task: Beyond groups, a shift in categorical perception from neutral to an emotional expression started earlier for happy than for angry faces.

Visual STM Task: both were less accurate in memory performance for angry than for happy faces. Also, both performed better at mid-intensity levels (30%-60%) than at low (10%–20%) and high (70%–90%) intensity levels.

Rating Task: Emotion X Group interaction > (1) PD rated fearful, disgusted, and sad faces as more negative compared with HCs. (2) PD gave higher Arousal ratings for fearful faces, and lower ratings for neutral faces, than HCs.

Range analysis of ratings showed that PD patients use an average 29% larger emotional range than HCs across dimensions (Valence, 40%; Arousal, 26%; Potency, 22%).

Tags: Emotion recognition, behavioral

Perception of emotional speech in Parkinson's Disease

Item Type Journal Article
Author Christine Schröder
Author Janine Möbes
Author Martin Schütze
Author Friedemann Szymanowski
Author Wido Nager
Author Marc Bangert
Author Thomas Frank Münte
Author Reinhard Dengler
Abstract Abstract Nonmotor symptoms in Parkinson's disease (PD) involving cognition and emotionality have progressively received attention. The objective of the present study was to investigate recognition of emotional prosody in patients with PD (n = 14) in comparison to healthy control subjects (HC, n = 14). Event-related brain potentials (ERP) were recorded in a modified oddball paradigm under passive listening and active target detection instructions. Results showed a poorer performance of PD patients in classifying emotional prosody. ERP generated by emotional deviants (happy/sad) during passive listening revealed diminished amplitudes of the mismatch-related negativity for sad deviants, indicating an impairment of early preattentive processing of emotional prosody in PD. © 2006 Movement Disorder Society
Date 10/2006
Language en
Library Catalog DOI.org (Crossref)
URL https://movementdisorders.onlinelibrary.wiley.com/doi/10.1002/mds.21038
Accessed 11.8.2025, 19:06:03
License http://onlinelibrary.wiley.com/termsAndConditions#vor
Volume 21
Pages 1774-1778
Publication Movement Disorders
DOI 10.1002/mds.21038
Issue 10
Journal Abbr Movement Disorders
ISSN 0885-3185, 1531-8257
Date Added 11.8.2025, 19:06:03
Modified 11.8.2025, 19:06:03

Notes:

Included

sample characteristics

size: 14 PD and 14 HC matched for age, sex, and education

Parkinson's Disease type and duration: idiopathic, Mduration= 4.8 (SD=5.5)

Medication: on medication

Hoehn-Yahr: M= 1.2 (SD=0.4)

UPDRS-3: M= 16.2 (SD=6.4)

Gender (male): 7 males (50%)

averaged ages (SD, range): M= 62.0 (SD=5.9)

other neurological disease (tumor, stroke, etc.): No dementia

other major psychopathology: No depression

origin country (or ethnicity): NA

method observational

instruments used in order to quantify the variables

Social cognition aspect: emotion recognition

Name of the task: NA

type of stimulus [face/voice etc., Ekman faces/other etc.]: The female first name "Anna" was prerecorded in three different emotional tones spoken by an experienced professional speaker (AD conversion rate, 44,000 Hz). A short two-syllable word "Anna" was chosen because of the requirements of the oddball paradigm.

task conditions: positive, negative, neutral

operationalization: RT and accuracy

Main findings related to the review's scope

RT: no significant differences between groups.

Accuracy: PD patients made more errors especially for sadly spoken words
Tags: Emotion recognition, behavioral

Perception of emotionally incongruent cues: evidence for overreliance on body vs. face expressions in Parkinson's disease

**Item Type**	Journal Article
**Author**	Yasmin Abo Foul
**Author**	David Arkadir
**Author**	Anastasia Demikhovskaya
**Author**	Yehuda Noyman
**Author**	Eduard Linetsky
**Author**	Muneer Abu Snineh
**Author**	Hillel Aviezer
**Author**	Renana Eitan
**Date**	2024-05-06
**Volume**	15
**Publication**	FRONTIERS IN PSYCHOLOGY
**DOI**	10.3389/fpsyg.2024.1287952
**ISSN**	1664-1078
**Date Added**	14.7.2025, 14:48:39
**Modified**	5.9.2025, 14:35:49

Notes:

Included

sample characteristics

size: Individuals with PD (N = 37), individuals with SCHIZOFRENIA (N = 30), and matched healthy controls (HC, N = 50)

Parkinson's Disease type and duration: idiopathic PD, Mduration=11.67 SD=8.76

Medication: on medication

Hoehn-Yahr: NA

UPDRS-3: M=19.09 SD=8.67

Gender (male): 26 males (70%)

averaged ages (SD, range): M=61.41 SD=8.8 (28-71)

other neurological disease (tumor, stroke, etc.): NA

other major psychopathology: None

origin country (or ethnicity): Israel

method (Review, meta-analysis or observational and/or self-reported):

instruments used in order to quantify the variables

Social cognition aspect: emotion recognition

Name of the task: NA

type of stimulus [face/voice etc., Ekman faces/other etc.]: Face-body composites were constructed with stimuli obtained from standardized sets. Stereotypical images of facial expressions of sadness, anger, fear, and happiness were taken from the Amsterdam Dynamic Facial Expression Set (ADFES) (van der Schalk et al., 2011). Stereotypical body expressions of sadness, anger, fear, and happiness were taken from the Bochum Emotional Stimulus Set (BESST) (Thoma et al., 2013). Using Adobe Photoshop, we created realistically proportioned face-body composites by crossing all emotional categories of faces with bodies. These stimuli have been recently validated in a normal population (Lecker et al., 2017) and in healthy older adults (Abo Foul et al., 2018).

task condition: congruent (e.g., an angry face on an angry body) and incongruent (for example, an angry face on a fearful body).

The experiment comprised three blocks, namely, (1) *body only*, (2) *face only*, and (3) *face with body*.

operationalization: Participants were instructed to select the emotion that best reflected the target's feelings from a list of four labels, namely, fear, anger, sadness, and happiness. In the case of face-body composites, no instructions were given prioritizing the face or body; rather, responses were to be made based on the overall impression of the target's emotion. >>> accuracy

Main findings related to the review's scope

Incongruent trials - to quantify the recognition of emotion from incongruent composites, we analyzed

the tendency to categorize the composites as conveying the emotion of the face, the body, or neither.

individuals with PD were more likely to be affected by body cues than the HC (p = 0.026) and SZ groups

HC had a greater tendency to categorize the composites according to facial expression than the PD group (p = 0.006) but no difference was found between the HC and SZ groups (p = 0.64).

Happy: Compared to individuals with PD, SZ and HC tended to categorize incongruent composites as the face (p<.001). Individuals with PD were more likely to categorize them as the body emotion or other emotion

Sad: Individuals with SZ were more likely to categorize composites as the face compared to PD group (p=.01), while they were less likely to categorize them as bodily expressions compared to PD (p=.001) and HC (p=.02).

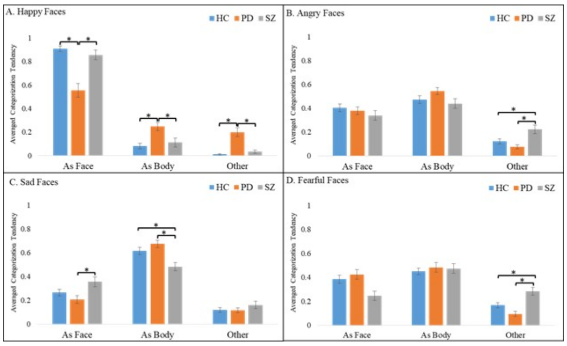

The averaged categorization tendency of: (A) happy faces, (B) angry faces, (C) sad faces and (D) fearful faces appearing with incongruent bodies for the HC, PD and SZ groups. The categorizations: as face emotion, as body emotion and other (i.e., the chosen emotion did not correspond to the face or the body) are placed along the x-axis. Error bars represent standard errors.

* $p < 0.01$. HC = healthy controls, PD = individuals with Parkinson's disease, SZ = individuals with schizophrenia.

Congruent trials

bodily emotions gained the lowest mean affective perception while facial expressions and congruent faces with bodies showed comparable means

happiness showed the highest affective perception average while fear gained the lowest mean

SZ group showed deteriorated affective perception abilities compared to HC and PD, that showed

comparable performance

Tags: Emotion recognition, behavioral

Perceptual Bias for Affective and Nonaffective Information in Asymmetric Parkinson's Disease

Item Type Journal Article
Author Jared G. Smith
Author John P. Harris
Author Salem Khan
Author Elizabeth A. Atkinson
Author M. Susan Fowler
Author Ralph P. Gregory
Abstract Objective: To relate lateralized impairments of visual perception in Parkinson's disease to asymmetries in the severity of motor symptoms. Method: Ten patients with worse left-sided motor symptoms (LPD), 15 with worse right-sided (RPD), and 13 healthy age-matched controls (all right-handed) viewed mirror-imaged pairs of emotional chimeric faces, (left side smiling, right neutral, and vice versa), of ereyscales (strips whose luminance varied smoothly from black on the left to white on the right, and vice versa) and of gender chimeric faces (left side male, right female, and vice versa). Participants signaled which stimulus appeared happier, brighter, or more feminine, respectively, so showing which side received more attention. Results: For emotional chimeras, controls and LPD showed little bias, whereas RPD showed a strong bias to left hemispace (p = .018, r = .45). Across all patients, this bias was associated with severity of right-sided motor impairment (p = .018, r = .49). The bias was much weaker and insignificant for greyscales (p = .72, r = .14). For gender chimeras, RPD again showed a significantly greater left hemispace bias than did LPD (p = .037, r = .47), although neither patient group differed significantly from controls. Across all patients, this bias correlated with ratio of right-to-left symptom severity (p = .044, r = .48). Conclusions: The left hemispace bias in RPD is greater for facial than for luminance judgments, and is amplified for emotional judgments. Asymmetrical degeneration of the striatum. particularly involving the left side, appears to underlie this deficit in visual processing.
Date 2010-07
Language English
Extra Place: 750 FIRST ST NE, WASHINGTON, DC 20002-4242 USA Type: Article
Volume 24
Publisher AMER PSYCHOLOGICAL ASSOC
Pages 443-456
Publication NEUROPSYCHOLOGY
DOI 10.1037/a0019279
Issue 4
ISSN 0894-4105
Date Added 14.7.2025, 14:50:41
Modified 5.9.2025, 14:57:14

Notes:

Included

Experiment 1:

Sample characteristics

Size: 25 PD -> 10 LPD (left side worse); 15 RPD (right side worse), 13 HC (age matched)

PD-type: Idiopathic PD

PD-duration: LPD: M = 7.10, SD = 4.60; RPD: M = 5.70, SD = 0.54

Medication: ON state

Hoehn-Yahr: in ON: LPD: M = 2.30, SD = 0.67; RPD: M = 2.60, SD = 0.54

UPDRS-3: in ON LPD: M = 18.56, SD = 3.28; RPD: M = 20.27, SD = 6.42

Gender (male): LPD: 5 (50%); RPD: 12 (80%)

Age: LPD: M = 65.62, SD = 6.63; RPD: M = 70.39, SD = 6.93

Other neurological disease (tumor, stroke, etc.): none

Other major psychopathology: none

Origin country (or ethnicity): England

method behavioral

instruments used in order to quantify the variables

Social cognition aspect: Facial emotion recognition

Name of the task: Emotional Chimeric Faces Test

Type of stimulus [face/voice etc., Ekman faces/other etc.]: 36 gif-files. photographing twice each of nine posers, once with a smiling and once with a neutral expression and joining the vertical halves of each together to form a chimeric face (i.e., a left-side smiling face was paired with a right-side neutral face or a right-side smiling face was paired with a left-side neutral face). Each chimera was then paired with its mirror image

Task condition: The 36 trials were arranged in a pseudorandom order whereby trials were divided into four randomly ordered blocks of nine trials.

Operationalization: Subjects were asked to indicate which of the two faces looked happier overall. >> Correct answers

---

Main findings related to the review's scope

Biases

Whereas the emotional chimeric faces asymmetry scores of both LPD patients (M 0.06, SD 0.53) and control subjects (M 0.07, SD 0.48) were not significantly different from zero (p .5), RPD patients exhibited a highly significant left hemispace bias for chimeras, (M 0.53, SD 0.43, t(14) 4.84, p .001, r .79).

Greater left hemispheric bias in RPD group
Tags: Emotion recognition, behavioral

Performance of Patients with Early Parkinson Disease on an Executive and Social Cognition Battery.

Item Type Journal Article
Author Sol Esteves
Author Ezequiel Gleichgerrcht
Author Teresa Torralva
Author Anabel Chade
Author Gonzalo Gómez Arévalo
Author Oscar Gershanik
Author Facundo Manes
Author María Roca
Abstract OBJECTIVE: To demonstrate the usefulness of incorporating the Executive and Social Cognition Battery (ESCB) to detect executive and social cognition deficits, which are otherwise not captured by more "classical" executive tests in early Parkinson disease (PD). BACKGROUND: PD is a neurodegenerative disorder that includes executive and social cognition deficits. While cognitive assessment in PD still relies on classical executive tasks to detect frontal deficits, these traditional tests often fail to uncover subtle, yet relevant, frontal impairment. METHODS: We evaluated 39 PD patients and 47 controls with a battery of classical executive tests and the ESCB. The ESCB includes a series of tasks that more closely resemble real-life activities and have been previously shown to be useful in detecting executive deficits in other neuropsychiatric disorders with frontal involvement. RESULTS: We observed that both batteries used in a complementary way yielded better results, as 15 of the 39 patients presented deficits only on some of the ESCB tests, but not on the classical battery, while 5 patients presented deficits only on some tests of the classical battery, but not on the ESCB. Fourteen patients presented deficits on some tests of either battery, and 5 patients did not present deficits on any of the tests. CONCLUSIONS: We found that, used along with traditional neuropsychological tasks, the ESCB may be useful in providing a more comprehensive evaluation of frontal dysfunction among patients with PD, thus contributing to the early diagnosis of cognitive disorders in this patient population.
Date 2018 Sep
Language eng
Extra Place: United States
Volume 31
Pages 142-150
Publication Cognitive and behavioral neurology : official journal of the Society for Behavioral and Cognitive Neurology
DOI 10.1097/WNN.0000000000000159
Issue 3
Journal Abbr Cogn Behav Neurol
ISSN 1543-3641 1543-3633
PMID 30239464

Date Added 6.7.2025, 19:09:34
Modified 5.9.2025, 14:35:14

Notes:

Included

sample characteristics

size: 39 PD and 47 HC same geographic area as patients.

Parkinson's Disease type and duration: NA

Medication: 17 on medication

Hoehn-Yahr: NA, max=2

UPDRS-3: NA

Gender (male): NA

averaged ages (SD, range): M=62.97 SD=10.04

other neurological disease (tumor, stroke, etc.): None

other major psychopathology: NA

origin country (or ethnicity): Argentina

method observational

instruments used in order to quantify the variables

Social cognition aspect: ToM

Name of the task: Mind in the Eyes

type of stimulus [face/voice etc., Ekman faces/other etc.]: 17 photographs of the eye region of different human faces.

operationalization: accuracy

Name of the task: Faux Pas (Stone et al, 1998)

type of stimulus [face/voice etc., Ekman faces/other etc.]: On each trial of the test, the participant was read a short, paragraph-long story. To reduce working memory load, a written version of the story was also placed in front of them. In 10 of the stories, a faux pas occurred, in which one character unintentionally said something hurtful or insulting to another. In the remaining 10 stories, there was no faux pas.

After each story, the participant was asked whether something inappropriate had been said and, if so, why it was inappropriate. If the answer was incorrect, a follow-up memory question was asked to ensure that the basic facts of the story had been retained. If they had not, the story was reexamined, and all questions were repeated.

operationalization: accuracy

Main findings related to the review's scope

PD performed worse than HC in both tasks

Tags: ToM, behavioral

Personality and social cognition in neurodegenerative disease.

**Item Type**	Journal Article
**Author**	Tal Shany-Ur
**Author**	Katherine P. Rankin
**Abstract**	PURPOSE OF REVIEW: Neurodegenerative diseases often cause focal damage to brain structures mediating social cognition and personality, resulting in altered interpersonal communication and behavior. We review recent research describing this phenomenon in various aspects of social cognition. RECENT FINDINGS: Corresponding to their pervasive socioemotional deficits, patients with frontotemporal dementia perform poorly on laboratory-based tasks including recognizing emotions, attending to salient information that guides social behavior, representing social knowledge, comprehending others' mental states, and maintaining insight to their own difficulties. Together with poor executive and regulation mechanisms, these social cognition deficits ultimately impact behavior. Patients with logopenic and nonfluent primary progressive aphasia have some deficits recognizing emotional prosody, whereas those with the semantic variant show more widespread deficits in social comprehension. Although Alzheimer's disease patients perform poorly on some social cognition tasks, this typically reflects general cognitive impairment, and their real-life social functioning is less affected than in diseases targeting frontotemporal structures. Studies in motor diseases such as Parkinson's suggest some degradation of emotion recognition and social comprehension, which should be investigated further. SUMMARY: We summarize recent findings concerning perception and evaluation of socioemotional information, social knowledge storage and access, advanced information processing mechanisms, and behavioral response selection and regulation across various neurodegenerative diseases.
**Date**	2011 Dec
**Language**	eng
**Extra**	Place: England
**Volume**	24
**Pages**	550-555
**Publication**	Current opinion in neurology
**DOI**	10.1097/WCO.0b013e32834cd42a
**Issue**	6
**Journal Abbr**	Curr Opin Neurol
**ISSN**	1473-6551 1350-7540
**PMID**	22002077
**PMCID**	PMC3808271
**Date Added**	6.7.2025, 19:09:34
**Modified**	5.9.2025, 14:56:35

Notes:

Not Included: Not a systematic Review
Tags: EXCLUDED

Predicting and Characterizing Neurodegenerative Subtypes with Multimodal Neurocognitive Signatures of Social and Cognitive Processes.

Item Type Journal Article
Author Agustín Ibañez
Author Sol Fittipaldi
Author Catalina Trujillo
Author Tania Jaramillo
Author Alejandra Torres
Author Juan F. Cardona
Author Rodrigo Rivera
Author Andrea Slachevsky
Author Adolfo García
Author Maxime Bertoux
Author Sandra Baez
Abstract BACKGROUND: Social cognition is critically compromised across neurodegenerative diseases, including the behavioral variant frontotemporal dementia (bvFTD), Alzheimer's disease (AD), and Parkinson's disease (PD). However, no previous study has used social cognition and other cognitive tasks to predict diagnoses of these conditions, let alone reporting the brain correlates of prediction outcomes. OBJECTIVE: We performed a diagnostic classification analysis using social cognition, cognitive screening (CS), and executive function (EF) measures, and explored which anatomical and functional networks were associated with main predictors. METHODS: Multiple group discriminant function analyses (MDAs) and ROC analyses of social cognition (facial emotional recognition, theory of mind), CS, and EF were implemented in 223 participants (bvFTD, AD, PD, controls). Gray matter volume and functional connectivity correlates of top discriminant scores were investigated. RESULTS: Although all patient groups revealed deficits in social cognition, CS, and EF, our classification approach provided robust discriminatory characterizations. Regarding controls, probabilistic social cognition outcomes provided the best characterization for bvFTD (together with CS) and PD, but not AD (for which CS alone was the best predictor). Within patient groups, the best MDA probabilities scores yielded high classification rates for bvFTD versus PD (98.3%, social cognition), AD versus PD (98.6%, social cognition+CS), and bvFTD versus AD (71.7%, social cognition+CS). Top MDA scores were associated with specific patterns of atrophy and functional networks across neurodegenerative conditions. CONCLUSION: Standardized validated measures of social cognition, in combination with CS, can provide a dimensional classification with specific pathophysiological markers of neurodegeneration diagnoses.
Date 2021
Language eng
Extra Place: United States
Volume 83
Pages 227-248

Publication Journal of Alzheimer's disease : JAD
DOI 10.3233/JAD-210163
Issue 1
Journal Abbr J Alzheimers Dis
ISSN 1875-8908 1387-2877
PMID 34275897
PMCID PMC8461708
Date Added 6.7.2025, 19:09:42
Modified 5.9.2025, 14:39:45

Notes:

Included

sample characteristics

size: 223 participants

Parkinson's Disease type and duration: 20 variant frontotemporal dementia (bvFTD), 33 typical AD, 51 PD. The performance of bvFTD, AD, and PD patients was compared with that of 29, 35, and 55 healthy controls, respectively. Matching criteria (age, sex, and years of education).

Medication: on medication

Hoehn-Yahr: NA

UPDRS-3: NA

Gender (male): 31 males (61%)

averaged ages (SD, range): M= 68.9 SD=7.9

other neurological disease (tumor, stroke, etc.): None

other major psychopathology: None

origin country (or ethnicity): Argentina, Chile and Colombia

method (Review, meta-analysis or observational and/or self-reported):

instruments used in order to quantify the variables

Social cognition aspect: emotion recognition and ToM

Name of the task: the Mini-Social Cognition and Emotional Assessment (MiniSEA)

type of stimulus [face/voice etc., Ekman faces/other etc.]: a FER test and a shortened version of the Faux Pas test, tapping ToM.

task condition: NA

operationalization: The total MiniSEA score is the sum of both subscores, yielding a maximum score of 30.

MRI: A subsample (n=176) of 16 bvFTD, 30 AD, 43 PD patients, and their matched controls (totaling 17, 30, and 40 subjects, respectively) underwent structural T1 scans. Resting-state fMRI recordings (n =168) were obtained from 14 bvFTD, 27 AD, 41 PD patients and their matched controls (17, 30, and 39, subjects respectively)—see matching criteria in Supplementary Table 4. Procceing NA.

VBM

MRI acquisition and preprocessing steps followed guidelines from the Organization for Human Brain Mapping [37]. Images were preprocessed using the DARTEL Toolbox following reported procedures [38]. Then, modulated 10-mm full-width half-maximum kernel-smoothed images [39] were normalized to the MNI space and analyzed through general linear models for 2nd level analyses on SPM-12

software. Based on previous literature

Functional connectivity analysis

The first five volumes of each subject's resting-state sequence were discarded. Then, as in previous FC reports [44], images were preprocessed with the Data Processing Assistant for Resting-State fMRI (DPARSF V4.3; http://rfmri.org/DPARSF). Following previous studies [44, 45], pre-processing steps included 1) slice-timing correction (using middle slice of each volume as the reference scan), 2) realignment to the first scan of the session to correct head movement, 3) normalization to the MNI space using the echo-planar imaging (EPI) template from SPM, 4) smoothing using a 8-mm full-width-at-half-maximum isotropic Gaussian kernel, and 5) bandpass filtering (0.01–0.08 Hz). Six motion parameters, CFS, and WM signals were regressed to reduce the effect of motion and physiological artifacts such as cardiac and respiration effects (REST V1.7 toolbox).

Main findings related to the review's scope

PD performed worse on the miniSEA than HC.

The MiniSEA discriminated most accurately between PD patients and controls. correctly classified 64.7% of PD.

In comparison to bvFTD or AD, the MiniSEA correctly classified 100% of PD patients.

For PD patients versus controls, the MiniSEA total score reached a sensitivity of 85% and a specificity of 65% at a cut-off of 23 points (AUC=0.78, CI: 0.68–0.88; $p<0.001$)

For bvFTD versus PD patients, the MiniSEA total score reached a sensitivity of 100% and a specificity of 93% at a cut-off of 15 points (AUC=0.99, CI: 0.99–1.00; $p<0.001$)

Higher values in the MiniSEA discriminant scores (greater probability of belonging to the PD group) were associated with lower GM volumes in parietal, frontal, and temporal regions

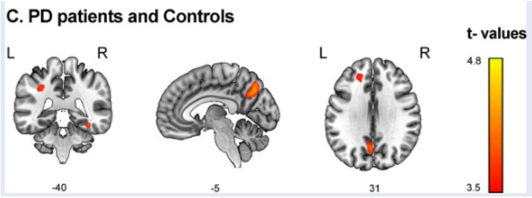

Higher discriminant scores from MiniSEA scores were associated with lower FC between (a) the right parietal superior lobule and the bilateral hippocampi and the cerebellum, (b) the right superior frontal gyrus and the angular gryus and posterior cingulate, and (c) the right inferior temporal gyrus and right superior occipital gyrus

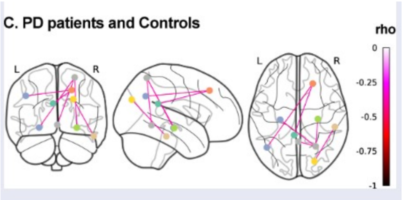

Supplementary Table 9. Brain regions related to discriminant scores in Parkinson's disease patients and controls.

Cluster size	Regions	Coordinates x	Coordinates y	Coordinates z	Peak T	Peak Z
	**MiniSEA**					
852	Precuneus L	-2	-72	50	4.81	4.50
	Precuneus L	-3	-72	40	4.56	4.29
593	Inferior parietal R	28	-54	54	4.24	4.02
223	Inferior parietal L	-32	-38	36	4.12	3.91
198	Inferior occipital gyrus R	36	-87	-9	4.07	3.87
151	Fusiform gyrus R	38	-42	-15	3.90	3.72
95	Superior temporal gyrus L	-40	-45	20	3.74	3.58
125	Middle frontal gyrus L	-20	32	22	3.52	3.39
132	Fusiform gyrus R	42	-15	-28	3.35	3.23
	Fusiform gyrus R	38	-27	-22	3.34	3.23

L: left; MiniSEA: Mini Social Cognition and Emotional Assessment; R: right.
$p < 0.001$, uncorrected; extent threshold = 30 voxels.

Supplementary Table 11. Associations between functional connectivity and discriminant scores in Parkinson's disease patients and controls.

Brain regions	Spearman's rho	*p*-value
**MiniSEA and IFS**		
Precentral gyrus L - Middle frontal gyrus R	-0,371	0,000697
Superior frontal gyrus L - Superior parietal lobule R	-0,398	0,00026
Superior frontal gyrus R - Superior parietal lobule R	-0,364	0,000892
Superior frontal gyrus, orbital R - Inferior frontal gyrus, orbital R	-0,409	0,000166
Middle frontal gyrus L - Superior parietal lobule R	-0,426	8,16E-05
Middle frontal gyrus R - Superior parietal lobule R	-0,39	0,000343
Inferior frontal gyrus, orbital L - Rectus gyrus L	-0,378	0,000547
Supplementary motor area L - Paracentral lobule L	-0,37	0,000717
Supplementary motor area L - Cerebelum R	-0,367	0,000815
Olfactory cortex R - Thalamus L	-0,399	0,000248
Olfactory cortex R - Thalamus R	-0,362	0,000981
Paracentral lobule L - Cerebelum L	-0,371	0,000705
Paracentral lobule L - Cerebelum R	-0,394	0,000296
Paracentral lobule L - Cerebelum R	-0,405	0,000193
Paracentral lobule L - Vermis	-0,386	0,000405

IFS: INECO Frontal Screening battery; L: left; MiniSEA: Mini Social Cognition and Emotional Assessment; R: right.
$p < 0.001$, uncorrected.

Tags: Imaging, G-SC

Preserved cognition after deep brain stimulation (DBS) in the subthalamic area for Parkinson's disease: a case report

**Item Type**	Journal Article
**Author**	Donatus Cyron
**Author**	Marion Funk
**Author**	Marie-An Deletter
**Author**	Kai Scheufler
**Abstract**	At present, subthalamic nucleus (STN) stimulation is the preferred procedure for the amelioration of motor symptoms in medication refractory Parkinson's disease. Results are however impaired by negative impacts on mood, cognition, incentive, and social judgment. Alternative targets are therefore explored. We describe a case with stimulation of subthalamic fibre tracts that showed clear improvement of cognitive and social abilities. Avoiding the STN may be advantageous in progressive Parkinson's disease to avoid non-motor complications and enhance quality of life.
**Date**	2010-12
**Language**	English
**Extra**	Place: SACHSENPLATZ 4-6, PO BOX 89, A-1201 WIEN, AUSTRIA Type: Article
**Volume**	152
**Publisher**	SPRINGER WIEN
**Pages**	2097-2100
**Publication**	ACTA NEUROCHIRURGICA
**DOI**	10.1007/s00701-010-0755-x
**Issue**	12
**ISSN**	0001-6268
**Date Added**	14.7.2025, 14:50:41
**Modified**	5.9.2025, 14:32:05

Notes:

Not Included: a case report

Tags: EXCLUDED

Primary vision and facial emotion recognition in early Parkinson's disease.

**Item Type**	Journal Article
**Author**	Géraldine Hipp
**Author**	Nico J. Diederich
**Author**	Vannina Pieria
**Author**	Michel Vaillant

Abstract BACKGROUND: In early stages of idiopathic Parkinson's disease (IPD), lower order vision (LOV) deficits including reduced colour and contrast discrimination have been consistently reported. Data are less conclusive concerning higher order vision (HOV) deficits, especially for facial emotion recognition (FER). However, a link between both visual levels has been hypothesized. OBJECTIVE: To screen for both levels of visual impairment in early IPD. METHODS: We prospectively recruited 28 IPD patients with disease duration of 1.4+/-0.8 years and 25 healthy controls. LOV was evaluated by Farnsworth-Munsell 100 Hue Test, Vis-Tech and Pelli-Robson test. HOV was examined by the Ekman 60 Faces Test and part A of the Visual Object and Space recognition test. RESULTS: IPD patients performed worse than controls on almost all LOV tests. The most prominent difference was seen for contrast perception at the lowest spatial frequency (p=0.0002). Concerning FER IPD patients showed reduced recognition of "sadness" (p=0.01). "Fear" perception was correlated with perception of low contrast sensitivity in IPD patients within the lowest performance quartile. Controls showed a much stronger link between "fear" perception" and low contrast detection. CONCLUSION: At the early IPD stage there are marked deficits of LOV performances, while HOV performances are still intact, with the exception of reduced recognition of "sadness". At this stage, IPD patients seem still to compensate the deficient input of low contrast sensitivity, known to be pivotal for appreciation of negative facial emotions and confirmed as such for healthy controls in this study.

Date 2014 Mar 15

Language eng

License Copyright © 2014 The Authors. Published by Elsevier B.V. All rights reserved.

Extra Place: Netherlands

Volume 338

Pages 178-182

Publication Journal of the neurological sciences

DOI 10.1016/j.jns.2013.12.047

Issue 1-2

Journal Abbr J Neurol Sci

ISSN 1878-5883 0022-510X

PMID 24484973

Date Added 6.7.2025, 19:09:34

Modified 5.9.2025, 14:39:15

Notes:

Included

sample characteristics

size: 28 early-stage idiopathic PD and 25 HC

Parkinson's Disease type and duration: Mduration = 1.36 SD= 0.8, Max duration = 3 years

Medication: on medication

Hoehn-Yahr: NA

UPDRS-3: M= 8.39 SD=3.52 (0-25)

Gender (male): 13 males (46%)

averaged ages (SD, range): M= 62.49 SD= 11.9

other neurological disease (tumor, stroke, etc.): NA

other major psychopathology: NA

origin country (or ethnicity): Luxembourg

method observational

instruments used in order to quantify the variables

Social cognition aspect: emotion recognition

Name of the task: the Ekman 60 Faces Test

type of stimulus [face/voice etc., Ekman faces/other etc.]: Ekman greyscale photographs of faces expressing one of the six emotions

task condition: anger, fear, disgust, happiness, sadness, and surprise

operationalization: correct sum

Main findings related to the review's scope

IPD patients performed worse than control subjects for the emotion “sadness”. There were no further significant differences between both groups.

IPD patients performed better on recognition of positive emotions, in comparison to negative ones.

Tags: Emotion recognition, behavioral

Problems with Facial Mimicry Might Contribute to Emotion Recognition Impairment in Parkinson's Disease.

Item Type Journal Article

Author Margaret T. M. Prenger
Author Penny A. MacDonald
Abstract Difficulty with emotion recognition is increasingly being recognized as a symptom of Parkinson's disease. Most research into this area contends that progressive cognitive decline accompanying the disease is to be blamed. However, facial mimicry (i.e., the involuntary congruent activation of facial expression muscles upon viewing a particular facial expression) might also play a role and has been relatively understudied in this clinical population. In healthy participants, facial mimicry has been shown to improve recognition of observed emotions, a phenomenon described by embodied simulation theory. Due to motor disturbances, Parkinson's disease patients frequently show reduced emotional expressiveness, which translates into reduced mimicry. Therefore, it is likely that facial mimicry problems in Parkinson's disease contribute at least partly to the emotional recognition deficits that these patients experience and might greatly influence their social cognition abilities and quality of life. The present review aims to highlight the need for further inquiry into the motor mechanisms behind emotional recognition in Parkinson's disease by synthesizing behavioural, physiological, and neuroanatomical evidence.
Date 2018
Language eng
Extra Place: United States
Volume 2018
Pages 5741941
Publication Parkinson's disease
DOI 10.1155/2018/5741941
Journal Abbr Parkinsons Dis
ISSN 2090-8083 2042-0080
PMID 30534356
PMCID PMC6252194
Date Added 6.7.2025, 19:09:36
Modified 14.7.2025, 15:11:06

Notes:

Not Included: Not a systematic Review
Tags: EXCLUDED

Processing emotional tone from speech in Parkinson’s disease: A role for the basal ganglia

Item Type Journal Article
Author Marc D. Pell
Author Carol L. Leonard
Date 12/2003
Language en
Short Title Processing emotional tone from speech in Parkinson’s disease
Library Catalog DOI.org (Crossref)

**URL**	http://link.springer.com/10.3758/CABN.3.4.275
**Accessed**	11.8.2025, 17:20:36
**License**	http://www.springer.com/tdm
**Volume**	3
**Pages**	275-288
**Publication**	Cognitive, Affective, & Behavioral Neuroscience
**DOI**	10.3758/CABN.3.4.275
**Issue**	4
**Journal Abbr**	Cognitive, Affective, & Behavioral Neuroscience
**ISSN**	1530-7026, 1531-135X
**Date Added**	11.8.2025, 17:20:36
**Modified**	11.8.2025, 17:20:36

Notes:

Included

sample characteristics

size: 21 PD and 21 HC matched sex, age, and educational status

Parkinson's Disease type and duration: idiopathic PD, Mduration=3.9 SD=1.9

Medication: on medication

Hoehn-Yahr: M=2 SD=0.5

UPDRS-3: M=14.5 SD=7.1

Gender (male): 11 males (53%)

averaged ages (SD, range): M=61.7 SD=8.6 51-83

other neurological disease (tumor, stroke, etc.): None

other major psychopathology: None

origin country (or ethnicity): Canada

method observational

instruments used in order to quantify the variables

Social cognition aspect: emotion recognition

Name of the task: NA

type of stimulus [face/voice etc., Ekman faces/other etc.]: four male and four female actors. Digital recordings of short nonsense utterances (e.g., Someone migged the pazing) and semantically biasing, well-formed utterances of comparable length (e.g., I didn't make the team) were elicited from each actor in five distinct emotional tones

task conditions: happiness, pleas ant surprise, anger, disgust, and sadness

operationalization: Discrimination of emotional prosody, Identification of emotional prosody. >> accuracy

Main findings related to the review's scope

Discrimination: non significant effect

Identification: Emotional prosody recognition was significantly inferior in the PD than in the HC group in the PP-3 and PP-5 tasks, each of which required subjects to derive their interpretations strictly from prosodic components of the signal

Tags: Emotion recognition, behavioral

Processing facial identity and emotional expression in normal aging and neurodegenerative diseases

Item Type Journal Article
Author Laurence Chaby
Author Pauline Narme
Abstract The ability to recognize facial identity and emotional facial expression is central to social relationships. This paper reviews studies concerning face recognition and emotional facial expression during normal aging as well as in neurodegenerative diseases occurring in the elderly. It focuses on Alzheimer's disease, frontotemporal and semantic dementia, and also Parkinson's disease. The results of studies on healthy elderly individuals show subtle alterations in the recognition of facial identity and emotional facial expression from the age of 50 years, and increasing after 70. Studies in neurodegenerative diseases show that - during their initial stages - face recognition and facial expression can be specifically affected. Little has been done to assess these difficulties in clinical practice. They could constitute a useful marker for differential diagnosis, especially for the clinical differentiation of Alzheimer's disease (AD) from frontotemporal dementia (FTD). Social difficulties and some behavioural problems observed in these patients may, at least partly, result from these deficits in face processing. Thus, it is important to specify the possible underlying anatomofunctional substrates of these deficits as well as to plan suitable remediation programs.
Date 2009-03
Language French
Extra Place: 127 AVE DE LA REPUBLIQUE, 92120 MONTROUGE, FRANCE Type: Article
Volume 7
Publisher JOHN LIBBEY EUROTEXT LTD
Pages 31-42
Publication PSYCHOLOGIE & NEUROPSYCHIATRIE DU VIEILLISSEMENT
DOI 10.1684/pnv.2008.0154
Issue 1
ISSN 1760-1703
Date Added 14.7.2025, 14:50:42
Modified 5.9.2025, 14:30:07

Notes:

Not Included: not in English
Tags: EXCLUDED

Real-life consequences of cognitive dysfunction in Parkinson's disease

Item Type Book Section
Author Kevin R. Kay
Author Ergun Y. Uc
Editor NS Narayanan

Editor RL Albin

Abstract While motor symptoms are the most recognized features of Parkinson's disease (PD), cognitive dysfunction is a key determinant of consequences of PD in real-life. In this chapter we review important domains where cognitive dysfunction negatively impacts the lives of people with PD (PwPD), such as difficulties in occupational and social life, and instrumental ADLs such as driving. Early loss of employment has important effects for PwPD, their families, and society. PwPD experience higher rates of family and social discord as well as important changes in their social roles. These processes are largely mediated through cognitive dysfunction, particularly difficulties processing and understanding emotions, decreased attention, and executive dysfunction. Cognitive dysfunction is also an important mediator of driving impairments, which contributes to decreased independence in PwPD. Finally, we briefly review the costs associated with cognitive impairment in PD. Both indirect and direct costs for PwPD with cognitive impairment are substantially higher than for PwPD with normal cognition.

Date 2022
Language English
Extra Type: Article; Book Chapter
Volume 269
Place 525 B STREET, SUITE 1900, SAN DIEGO, CA 92101-4495 USA
Publisher ELSEVIER ACADEMIC PRESS INC
ISBN 978-0-323-90164-2
Pages 113-136
Series Progress in Brain Research
Book Title COGNITION IN PARKINSON'S DISEASE
DOI 10.1016/bs.pbr.2022.01.005
ISSN 0079-6123
Date Added 14.7.2025, 14:50:30
Modified 5.9.2025, 14:41:37

Notes:

Not Included: A book chapter

Tags: EXCLUDED

Recognition of emotion from facial, prosodic and written verbal stimuli in Parkinson's disease.

Item Type Journal Article

Author Yayoi Kan
Author Mitsuru Kawamura
Author Yukihiro Hasegawa
Author Satoshi Mochizuki
Author Katsuki Nakamura

Abstract Although the basal ganglia are thought to be important in recognizing emotion, there is contradictory evidence as to whether patients with Parkinson's disease (PD) have deficits in recognizing facial expressions. In addition, few studies have examined their ability to recognize emotion from non-visual stimuli, such as voices. We examined the ability of PD patients and age-matched controls to recognize emotion in three different modalities: facial, prosodic, and written verbal stimuli. Compared to controls, PD patients showed deficits in recognizing fear and disgust in facial expressions. These impairments were not seen in their recognition of prosodic or written verbal stimuli. This modality-specific deficit suggests that the neural substrates for recognizing emotion from different modalities are not fully identical.

Date 2002 Sep
Language eng
Extra Place: Italy
Volume 38
Pages 623-630
Publication Cortex; a journal devoted to the study of the nervous system and behavior
DOI 10.1016/s0010-9452(08)70026-1
Issue 4
Journal Abbr Cortex
ISSN 0010-9452
PMID 12465672
Date Added 6.7.2025, 19:09:37
Modified 5.9.2025, 14:41:10

Notes:

Included

sample characteristics

size: 16 PD and 24 HC

Parkinson's Disease type and duration: NA

Medication: on medication

Hoehn-Yahr: 2 or 3

UPDRS-3: NA

Gender (male): 5 males (31%)

averaged ages (SD, range): M= 68.8 SD= 7.30

other neurological disease (tumor, stroke, etc.): None demented

other major psychopathology: 7 were considered depressed

origin country (or ethnicity): Japan

method observational

instruments used in order to quantify the variables

Social cognition aspect: emotion recognition

Name of the task: NA

type of stimulus [face/voice etc., Ekman faces/other etc.]: videotaped facial expressions expressed by professional male and female actors. The actors were filmed twice, first head-on and then from a 45-degree angle.

task condition: happiness, sadness, anger, fear, surprise, and disgust

type of stimulus: static facial expressions, we used the facial stimuli sets from Nakamura et al. (1999)

task condition: happiness, sadness, anger, disgust, and emotionally neutral.

type of stimulus: Prosodic Stimuli - The same actors were requested to read four semantically neutral sentences (such as "good morning") and six short, nonsense sentences, and to use tone to convey the six basic emotions, while being recorded on an audiocassette tape.

task condition: happiness, sadness, anger, fear, surprise, disgust

type of stimulus: Written Verbal Stimuli - written verbal stimuli from Adolphs et al. (1999), in translation, which consist of 30 sentences

task condition: happiness, sadness, anger, fear, surprise, disgust

operationalization: select from the cards the one basic emotion that best described the emotional state

represented in the video recording, photograph, tape recording, or sentence. >> percentage of correct responses

Main findings related to the review's scope

Emotion Recognition from Moving Facial Stimuli: PD patients' recognition of fear and disgust was impaired compared to that of control

PD patients recognized fear and disgust less well than the other four emotions

fear was the emotion least recognized by both PD patients and controls

Emotion Recognition from Prosodic Stimuli: beyond groups, fear and disgust were recognized less than the other four emotions

Emotion Recognition from Written Verbal Stimuli: beyond groups, surprise was less well recognized than happiness or anger, and recognition of disgust was worse than recognition of happiness

Correlations: better static facial recognition was correlated positivly with moving face recognition and prosodic stimuli; all three were significantly correlated to each other. None were related to Written verbal stimuli.

Tags: Emotion recognition, behavioral

Recognition of emotional prosody is altered after subthalamic nucleus deep brain stimulation in Parkinson's disease

Item Type Journal Article

Author Julie Peron
Author Didier Grandjean
Author Florence Le Jeune
Author Paul Sauleau
Author Claire Haegelen
Author Dominique Drapier
Author Tiphaine Rouaud
Author Sophie Drapier
Author Marc Verin

Abstract The recognition of facial emotions is impaired following subthalamic nucleus (STN) deep brain stimulation (DBS) in Parkinson's disease (PD). These changes have been linked to a disturbance in the STN's limbic territory, which is thought to be involved in emotional processing. This was confirmed by a recent PET study where these emotional modifications were correlated with changes in glucose metabolism in different brain regions, including the amygdala and the orbitofrontal regions that are well known for their involvement in emotional processing. Nevertheless, the question as to whether these emotional changes induced by STN DBS in PD are modality-specific has yet to be answered. The objective of this study was therefore to examine the effects of STN DBS in PD on the recognition of emotional prosody. An original emotional prosody paradigm was administered to twenty-one post-operative PD patients, twenty-one pre-operative PD patients and twenty-one matched controls. Results showed that both the pre- and post-operative groups differed from the healthy controls. There was also a significant difference between the pre and post groups.

More specifically, an analysis of their continuous judgments revealed that the performance of the post-operative group compared with that of the other two groups was characterized by a systematic emotional bias whereby they perceived emotions more strongly. These results suggest that the impaired recognition of emotions may not be specific to the visual modality but may also be present when emotions are expressed through the human voice, implying the involvement of the STN in the brain network underlying the recognition of emotional prosody. (C) 2009 Elsevier Ltd. All rights reserved.

**Date**	2010-03
**Language**	English
**Extra**	Place: THE BOULEVARD, LANGFORD LANE, KIDLINGTON, OXFORD OX5 1GB, ENGLAND Type: Article
**Volume**	48
**Publisher**	PERGAMON-ELSEVIER SCIENCE LTD
**Pages**	1053-1062
**Publication**	NEUROPSYCHOLOGIA
**DOI**	10.1016/j.neuropsychologia.2009.12.003
**Issue**	4
**ISSN**	0028-3932
**Date Added**	14.7.2025, 14:50:41
**Modified**	5.9.2025, 14:50:38

Notes:

Included

Sample characteristics

Size: 21 PD, 21 PD-DBS, 21 HC (matched for gender, age, education

PD-type: Idiopathic PD

PD-duration: PD: M = 11, SD = 3.6; PD-DBS: M = 11.3, Sd = 4.1

Medication: on state

Hoehn-Yahr (ON): PD: M = 1.3, SD = 0.6; PD-DBS: M = 1.3, SD = 1.0

UPDRS-3 (ON): PD: M = 9.5, SD = 6.9; PD-DBS: M = 13.7, SD = 8.8

Gender (male): PD: 10 (45%); PD-DBS: 10 (4%)

Age: PD: M = 59.5, SD = 7.9; PD-DBS: M = 58.8, SD = 7.4

Other neurological disease (tumor, stroke, etc.): none

Other major psychopathology: none

Origin country (or ethnicity): France

method behavioural

instruments used in order to quantify the variables

Social cognition aspect: Vocal emotion recognition

Name of the task: Na

Type of stimulus [face/voice etc., Ekman faces/other etc.]: The set of vocal stimuli (pseudowords) comprised 60 stimuli: 12 actors × 5 emotion conditions. short segments of meaningless speech (pseudowords).

Task condition: Four different categories of emotional prosody (anger, fear, happiness and sadness), together with a neutral condition

Operationalization: judge the emotional content of each stimulus using a set of visual analogue scales displayed simultaneously on the computer screen. More specifically, participants were instructed to judge the extent to which the different emotions were expressed on a visual analogue scale ranging from “not at all” to “very much”. Participants rated six scales: one scale for each emotion presented (anger, fear, happiness and sadness) and one for the neutral utterance, plus a scale to rate the “surprise” emotion

A response was deemed to be correct when the subject rated the “Target” scale (e.g. the “Anger” scale when the stimulus was “Anger”) higher than all the other scales

Main findings related to the review's scope

Categorical judgements

pre-operative PD patient group performed more poorly than the HC group, 2(1) = 6.21, p = .01). There was no difference between the pre and post groups, 2(1) = 0.85, p = .1, or between the post and HC

groups, 2(1) = 2.47, p = .12.

No sig. diff. for any emotions: Anger: 2(2) = 3.95, p = .14; Fear: 2(2) = 2.62, p = .27; Happiness: 2(2) = 2.05, p = .36; Neutral: 2(2) = .48, p = .79; Sadness: 2(2) = 5.60, p = .06

Tags: Emotion recognition, behavioral

Recognition of emotions expressed on the face impairments in Parkinson's disease

Item Type Journal Article
Author Kaveh Shafiei
Author Mohammad Ali Shafa
Author Forugh Mohammadi
Author Ali Arabpour
Abstract Background: Facial emotion recognition (FER) is a complex process, involving many brain circuits, including the basal ganglia that its motor involvement causes Parkinson's disease (PD). The previous studies used different tools for assessment of FER in PD. There is a discrepancy between the results of these studies due to different tools. In this study, we used a modified version of the Multimodal Emotion Recognition Test (MERT) to compare patients with PD to healthy controls (HCs). Methods: It was a cross-sectional study with primary objective of the mean percentage of the correct answers in MERT. Subjects had to name the emotions presented with different modalities. Results: 30 subjects were recruited and assessed in each group. The mean total MERT score was significantly lower in subjects with PD compared to HCs (35.0% vs. 44.5%). FER was significantly better when emotions were presented by video and worse when presented by still pictures. Both subjects with PD and HCs had lower MERT scores in recognizing negative emotions. There was no significant correlation between the duration and severity of PD and MERT score. Conclusion: Our study provided more pieces of evidence for impairment of FER in PD for recognizing emotions like sadness, disgust, and fear compared to happy expressions.
Date 2020
Language English
Extra Place: CTR ELECTR RES PROVISION & J IMPROVEMENT, #7, POURSINA AVE, QODS AVE, PO 1417653911, TEHRAN, 11498, IRAN Type: Article
Volume 19
Publisher TEHRAN UNIV MEDICAL SCIENCES
Pages 32-35
Publication CURRENT JOURNAL OF NEUROLOGY
Issue 1
ISSN 2717-011X
Date Added 14.7.2025, 14:50:32
Modified 5.9.2025, 14:56:28

Notes:

Included

Sample characteristics

Size: 30 PD, 30 HC

PD-type: Idiopathic PD

PD-duration: NA

Medication: ON state

Hoehn-Yahr: M = 2.3, SD = 0.4

UPDRS-3: NA

Gender (male): 17 (56%)

Age: M = 58.2, SD = 7.0

Other neurological disease (tumor, stroke, etc.): None

Other major psychopathology: None

Origin country (or ethnicity): Iran

method behavioral

instruments used in order to quantify the variables

Social cognition aspect: Emotion recognition

Name of the task: Multimodal Emotion Recognition Test (MERT)

Type of stimulus [face/voice etc., Ekman faces/other etc.]: 30 video clips of actors for each of 10 emotions. The emotions presented to the subjects in four modalities: Still picture, Videos, Videos with audio and only audio, and in total of 120 items. The auditory content of the test was a standard pseudo-linguistic sentence (i.e., a sentence without meaning).

Task condition: despair, cold and hot anger, anxiety, panic fear, happiness, elated joy, disgust, contempt, and sadness

Operationalization: identify the presented emotions. >> accuracy mean score

Main findings related to the review's scope

Mean total MERT score of patients with PD was significantly lower than HCs, regardless of modality in which emotion was presented.

patients with PD had significantly lower score in recognizing contempt, anxiety, disgust, cold anger, and hot anger. no other sig dif.

Tags: Emotion recognition, behavioral

Recognition of emotions from visual and prosodic cues in Parkinson's disease.

**Item Type**	Journal Article
**Author**	Alessandra Ariatti
**Author**	Francesca Benuzzi
**Author**	Paolo Nichelli
**Abstract**	OBJECTIVE: To assess whether Parkinson Disease (PD) patients are impaired at perceiving emotions from facial and prosodic cues and whether any putative defective performance concerns recognition of a particular emotion. BACKGROUND: Braak et al. [1] demonstrated that in different stages PD pathology involves the nigrostriatal system, the amygdala, and the insular cortex. Discrete brain lesions to these structures can cause selective deficits in recognising facial and prosodic stimuli expressing particular emotions. However, the investigation of facial and prosodic emotional processing in PD patients has lead to conflicting results. MATERIALS AND METHODS: We compared 27 cognitively unimpaired PD patients with control subjects by means of the Facial Emotion Recognition Battery and the Emotional Prosody Recognition Battery. RESULTS: PD patients were impaired in recognising, selecting, and matching facial affects. In particular, the Facial Emotion Recognition Battery demonstrated a severe impairment in recognising sad and fearful faces. In the Emotional Prosody Recognition Battery PD patients demonstrated a diffuse impairment, including the recognition of emotional and propositional prosody. CONCLUSIONS: Face emotion processing is impaired in PD patients, with a disproportionate deficit involving fear and sadness. The pattern of face expression processing impairment in PD patients might depend on the regional distribution of the pathology. The widespread involvement of both emotional and propositional prosodic processing parallels the aprosodic characteristics of Parkinsonian speech production.
**Date**	2008 Sep
**Language**	eng
**Extra**	Place: Italy
**Volume**	29
**Pages**	219-227
**Publication**	Neurological sciences : official journal of the Italian Neurological Society and of the Italian Society of Clinical Neurophysiology
**DOI**	10.1007/s10072-008-0971-9
**Issue**	4
**Journal Abbr**	Neurol Sci
**ISSN**	1590-1874
**PMID**	18810595
**Date Added**	6.7.2025, 19:09:37
**Modified**	5.9.2025, 14:26:50

Notes:

Included

sample characteristics

size: 27 PD and 68 HC

Parkinson's Disease type and duration: idiopathic, Mduration=5.96 SD=3.44 (1-15)

Medication: NA

Hoehn-Yahr: M=1.92 SD=0.64 (1-3)

UPDRS-3: M=28.85 SD=10.73 (6-52)

Gender (male): 15 males (56%)

averaged ages (SD, range): M=66.93 SD=6.67 (51-76)

other neurological disease (tumor, stroke, etc.): None

other major psychopathology: None

origin country (or ethnicity): Italy

method observational

instruments used in order to quantify the variables

Social cognition aspect: Emotion Recognition

Name of the task: The Facial Emotion Recognition Battery

type of stimulus [face/voice etc., Ekman faces/other etc.]: Ekman faces

task condition: neutral, happiness, sadness, disgust, fear and anger.

operationalization: Facial Affect Naming (FAN), Facial Affect Selection (FAS), Facial Affect Matching (FAM)

Name of the task: The Emotional Prosody Recognition Battery

type of stimulus [face/voice etc., Ekman faces/other etc.]: visual and auditory presentation of Italian sentences arranged in four different tasks

task condition: neutral, happiness, sadness, disgust, fear and anger.

operationalization: Prosodic Affect Naming (PrAN), Prosodic Affect Discrimination (PrAD)

accuracy in all

Main findings related to the review's scope

PD impaired at the Facial Affect Name Recognition and at the Facial Affect Matching tasks in comparison to HC

Facial Affect Name: Specifically, PD turned out to be impaired on fear and sadness

Compared with controls, PD patients were significantly impaired at the Prosodic Affect Naming task. Specifically, PD patients were impaired in recognising happy intonation.

Tags: Emotion recognition, behavioral

Recognition of facial and musical emotions in Parkinson's disease.

**Item Type**	Journal Article
**Author**	A. Saenz
**Author**	A. Doé de Maindreville
**Author**	A. Henry
**Author**	S. de Labbey
**Author**	S. Bakchine
**Author**	N. Ehrlé
**Abstract**	BACKGROUND AND PURPOSE: Patients with amygdala lesions were found to be impaired in recognizing the fear emotion both from face and from music. In patients with Parkinson's disease (PD), impairment in recognition of emotions from facial expressions was reported for disgust, fear, sadness and anger, but no studies had yet investigated this population for the recognition of emotions from both face and music. METHODS: The ability to recognize basic universal emotions (fear, happiness and sadness) from both face and music was investigated in 24 medicated patients with PD and 24 healthy controls. The patient group was tested for language (verbal fluency tasks), memory (digit and spatial span), executive functions (Similarities and Picture Completion subtests of the WAIS III, Brixton and Stroop tests), visual attention (Bells test), and fulfilled self-assessment tests for anxiety and depression. RESULTS: Results showed that the PD group was significantly impaired for recognition of both fear and sadness emotions from facial expressions, whereas their performance in recognition of emotions from musical excerpts was not different from that of the control group. The scores of fear and sadness recognition from faces were neither correlated to scores in tests for executive and cognitive functions, nor to scores in self-assessment scales. CONCLUSION: We attributed the observed dissociation to the modality (visual vs. auditory) of presentation and to the ecological value of the musical stimuli that we used. We discuss the relevance of our findings for the care of patients with PD.
**Date**	2013 Mar
**Language**	eng
**License**	© 2012 The Author(s) European Journal of Neurology © 2012 EFNS.
**Extra**	Place: England
**Volume**	20
**Pages**	571-577
**Publication**	European journal of neurology
**DOI**	10.1111/ene.12040
**Issue**	3
**Journal Abbr**	Eur J Neurol

ISSN 1468-1331 1351-5101
PMID 23279689
Date Added 6.7.2025, 19:09:36
Modified 5.9.2025, 14:54:40

Notes:

Included

Sample characteristics

Size: 24 PD, 24 HC (matched for age, education, gender)

PD-type: NA

PD-duration: at least 4 years

Medication: on state

Hoehn-Yahr: max = 4; M = 2, SD = 0.37

UPDRS-3: M = 18.62, SD = 11.13

Gender (male): 12 (50%)

Age: M = 63.83, SD = 7.15

Other neurological disease (tumor, stroke, etc.):None

Other major psychopathology: Nine patients were receiving an antidepressant

Origin country (or ethnicity): France

method behavioral

instruments used in order to quantify the variables

Social cognition aspect: emotion recognition

Name of the task: NA

Type of stimulus [face/voice etc., Ekman faces/other etc.]: Adapted from the Pictures of Facial Affects. For each emotion (fear, happiness and sadness), hybrid faces were created by using a morphing software allowing variation of the level of emotional intensity, from full-blown expression (100% intensity) to neutral expression (0% intensity). For each emotion, we generated a continuum comprising 10 stimuli by increasing the level of intensity by 10%. The final stimuli set was composed of 30 black and white photographs

Task condition: fear, happiness and sadness

Operationalization: The participants were asked to produce orally the label that best described the facial expression shown on the screen. >> Correct answers

Main findings related to the review's scope

Facial emotion recognition

Scores of the PD group for emotional recognition from face were significantly lower than those obtained by the control group.

PD performed worse compared to HC in identifying both fear and sadness, whereas the difference between the HC and the patient group did not reach the significance level for happiness.

Tags: emotion recognition, behavioral

[Recognition of facial expression of emotions in Parkinson's disease: a theoretical review].

**Item Type**	Journal Article
**Author**	L. Alonso-Recio
**Author**	J. M. Serrano-Rodriguez
**Author**	F. Carvajal-Molina
**Author**	A. Loeches-Alonso
**Author**	P. Martin-Plasencia
**Abstract**	INTRODUCTION: Emotional facial expression is a basic guide during social interaction and, therefore, alterations in their expression or recognition are important limitations for communication. AIM: To examine facial expression recognition abilities and their possible impairment in Parkinson's disease. DEVELOPMENT: First, we review the studies on this topic which have not found entirely similar results. Second, we analyze the factors that may explain these discrepancies and, in particular, as third objective, we consider the relationship between emotional recognition problems and cognitive impairment associated with the disease. Finally, we propose alternatives strategies for the development of studies that could clarify the state of these abilities in Parkinson's disease. CONCLUSIONS: Most studies suggest deficits in facial expression recognition, especially in those with negative emotional content. However, it is possible that these alterations are related to those that also appear in the course of the disease in other perceptual and executive processes. To advance in this issue, we consider necessary to design emotional recognition studies implicating differentially the executive or visuospatial processes, and/or contrasting cognitive abilities with facial expressions and non emotional stimuli. The precision of the status of these abilities, as well as increase our knowledge of the functional consequences of the characteristic brain damage in the disease, may indicate if we should pay special attention in their rehabilitation inside the programs implemented.
**Date**	2012 Apr 16
**Language**	spa
**Extra**	Place: Singapore
**Volume**	54
**Pages**	479-489
**Publication**	Revista de neurologia
**Issue**	8
**Journal Abbr**	Rev Neurol
**ISSN**	1576-6578 0210-0010
**PMID**	22492101
**Date Added**	6.7.2025, 19:09:38
**Modified**	5.9.2025, 14:25:35

Notes:

Not Included: not a systematic revirew

Tags: EXCLUDED

Reduced early visual emotion discrimination as an index of diminished emotion processing in Parkinson's disease? - Evidence from event-related brain potentials.

**Item Type**	Journal Article
**Author**	Matthias J. Wieser
**Author**	Elisabeth Klupp
**Author**	Peter Weyers
**Author**	Paul Pauli
**Author**	David Weise
**Author**	Daniel Zeller
**Author**	Joseph Classen
**Author**	Andreas Mühlberger
**Abstract**	Although Parkinson's disease (PD) is defined by its motor symptoms, it is now well recognized that cognitive and affective domains, such as recognition of emotion from facial expressions, may also be impaired. To examine brain mechanisms involved in processing of emotion recognition from facial expressions, we obtained affective ratings and visual event-related potentials (ERPs) in response to facial expressions from 18 PD patients under dopamine-replacement therapy, and 17 healthy age- and sex-matched controls. In control subjects, the early posterior negativity (EPN) of the ERP, which is thought to reflect early perceptual emotion discrimination, was larger in response to emotional compared to neutral facial expressions. In contrast, this emotional modulation of the EPN was absent in PD patients indicating impaired early emotion discrimination. Behaviorally, PD patients showed no impairments in emotion recognition as measured by affective ratings. These findings suggest that facial emotion processing may be disrupted at an early stage of visual neural processing in PD. Absence of behavioral impairment may point to compensatory strategies of emotion recognition in medicated PD patients. Further research should clarify these dissociations between behavioral and neurophysiological levels of emotion processing in PD.
**Date**	2012 Oct
**Language**	eng
**License**	Copyright © 2011 Elsevier Srl. All rights reserved.
**Extra**	Place: Italy
**Volume**	48
**Pages**	1207-1217
**Publication**	Cortex; a journal devoted to the study of the nervous system and behavior
**DOI**	10.1016/j.cortex.2011.06.006
**Issue**	9
**Journal Abbr**	Cortex
**ISSN**	1973-8102 0010-9452
**PMID**	21764048
**Date Added**	6.7.2025, 19:09:39
**Modified**	5.9.2025, 15:00:58

Notes:

Included

Sample characteristics

Size: 18 PD, 17 HC (matched for age, education and sex)

PD-type: Idiopathic PD

PD-duration: M = 4.9, SD = 4.0, Range = 0.5-13

Medication: ON state

Hoehn-Yahr: 1-3, Median = 2

UPDRS-3: M = 16.2, SD = 6.2

Gender (male): 13 (72%)

Age: *M* = 62.9 years, *SD* = 9.9

Other neurological disease (tumor, stroke, etc.): None

Other major psychopathology: None

Origin country (or ethnicity): Germany

method (Review, meta-analysis or observational and/or self-reported):

instruments used in order to quantify the variables

Social cognition aspect: Emotion Recognition

Name of the task: NA

Type of stimulus [face/voice etc., Ekman faces/other etc.]: Photographs of faces of 36 different individuals (18 males and 18 females) were taken from the Karolinska Directed Emotional Faces (KDEF) database

Task condition: For this experiment, angry, happy, fearful, disgusted, sad, and neutral expressions in frontal orientation were used.

Operationalization: participants were asked to make a forced-choice emotion recognition task >> Correct answers.

and participants were asked to rate the subset used in the emotion recognition task earlier with respect to emotional arousal and valence on nine-point visual analog scales by pressing the appropriate key on the keyboard >> average score for arousal

Main findings related to the review's scope

No differences between groups and no Group Emotion interaction emerged

With regard to arousal, no group differences or interaction of group and emotion were observed

Tags: emotion recognition, behavioral

Reduced Empathy Scores in Patients with Parkinson's Disease: A Non-Motor Symptom Associated with Advanced Disease Stages.

**Item Type**	Journal Article
**Author**	Nele Schmidt
**Author**	Laura Paschen
**Author**	Günther Deuschl
**Author**	Karsten Witt
**Abstract**	BACKGROUND: Empathy describes the ability to infer and share emotional experiences of other people and is a central component of normal social functioning. Impaired empathy might be a non-motor symptom in Parkinson's disease (PD). OBJECTIVE: To examine empathic abilities and their relationship to clinical and cognitive functioning in PD patients. METHODS: Empathy was measured in 75 non-demented PD patients and 34 age-matched healthy controls using a German version of the Interpersonal Reactivity Index. Moreover, we collected demographic and clinical data and conducted a comprehensive neuropsychological test battery. RESULTS: PD patients had a significant lower global empathy score than healthy controls. Furthermore, we found significant group differences for the cognitive empathy scales but not for the scales which are sensitive for affective empathy components. The empathy decrease was significantly higher in advanced Hoehn & Yahr stages. There were only sporadic significant correlations between empathy scores and cognitive variables. CONCLUSIONS: PD patients show a stage dependent empathy score decrease which is driven mainly by cognitive aspects of empathy. However, emotional empathy aspects are not reduced.
**Date**	2017
**Language**	eng
**Extra**	Place: United States
**Volume**	7
**Pages**	713-718
**Publication**	Journal of Parkinson's disease
**DOI**	10.3233/JPD-171083
**Issue**	4
**Journal Abbr**	J Parkinsons Dis
**ISSN**	1877-718X 1877-7171
**PMID**	28759973
**Date Added**	6.7.2025, 19:09:40
**Modified**	5.9.2025, 14:55:55

Notes:

Not Included: Same sample as the previous article (“Impaired empathy: A non-motor symptom associated with advanced stages of Parkinson's disease”)

Tags: EXCLUDED

Reduced facial expressiveness in Parkinson's disease: A pure motor disorder?

**Item Type**	Journal Article
**Author**	Lucia Ricciardi
**Author**	Matteo Bologna
**Author**	Francesca Morgante
**Author**	Diego Ricciardi
**Author**	Bruno Morabito
**Author**	Daniele Volpe
**Author**	Davide Martino
**Author**	Alessandro Tessitore
**Author**	Massimiliano Pomponi
**Author**	Anna Rita Bentivoglio
**Author**	Roberto Bernabei
**Author**	Alfonso Fasano
**Abstract**	Background and aims: Impaired emotional facial expressiveness is an important feature in Parkinson's disease (PD). Although there is evidence of a possible relationship between reduced facial expressiveness and altered emotion recognition or imagery in PD, it is unknown whether other aspects of the emotional processing, such as subjective emotional experience (alexithymia), might influence hypomimia in this condition. In this study wee aimed to investigate possible relationship between reduced facial expressiveness and altered emotion processing (including facial recognition and alexithymia) in patients with PD. Methods: Forty PD patients and seventeen healthy controls were evaluated. Facial expressiveness was rated on video recordings, according to the UPDRS-III item 19 and using an ad hoc scale assessing static and dynamic facial expression and posed emotions. Six blind raters evaluated the patients' videos. Emotion facial recognition was tested using the Ekman Test; alexithymia was assessed using Toronto Alexithymia Scale (TAS-20). Results: PD patients had a significantly reduced static and dynamic facial expressiveness and a deficit in posing happiness and surprise. They performed significantly worse than healthy controls in recognizing surprise (p = 0.03). The Ekman total score positively correlated with the global expressiveness (R boolean AND 2 = 0.39, p = 0.01) and with the expressiveness of disgust (R boolean AND 2 = 032, p = 0.01). The occurrence of alexithymia was not different between PD patients and HC; however, a significant negative correlation between the expressiveness of disgust was found for a subscore of TAS (R boolean AND 2 = -.447, p = 0.007). Conclusions: Reduced facial expressiveness in PD may be in part related to difficulties with emotional recognition in a context of an unimpaired subjective emotional experience. (C) 2015 Elsevier B.V. All rights reserved.
**Date**	2015-11-15
**Language**	English
**Extra**	Place: PO BOX 211, 1000 AE AMSTERDAM, NETHERLANDS Type: Article
**Volume**	358
**Publisher**	ELSEVIER SCIENCE BV
**Pages**	125-130
**Publication**	JOURNAL OF THE NEUROLOGICAL SCIENCES
**DOI**	10.1016/j.jns.2015.08.1516
**Issue**	1-2
**ISSN**	0022-510X
**Date Added**	14.7.2025, 14:50:36
**Modified**	5.9.2025, 14:52:36

Notes:

Included

Sample characteristics

Size: 40 PD, 17 HC (no difference in age, education, MMSE and BDI)

PD-type: NA

PD-duration: M = 7.6, SD = 4.7

Medication: Assessment in ON

Hoehn-Yahr: Collected, but not reported

UPDRS-3: M = 23.2, SD = 8.4

Gender (male): 22 (55%)

Age: M = 68.85, SD 0 7.8

Other neurological disease (tumor, stroke, etc.): none

Other major psychopathology: none

Origin country (or ethnicity): Italy

method behavioural

instruments used in order to quantify the variables

Social cognition aspect: emotion recognition

Name of the task: Ekman 60 Faces TEst

Type of stimulus [face/voice etc., Ekman faces/other etc.]: 60 Ekman pictures (6 basic emotions: happiness, sadness, disgust, fear, anger, surpirse)

Task condition: 6 way forced choice

Operationalization: The maximum total score for all six emotions is 60 (10 for each basic emotion).

Main findings related to the review's scope

PD patients performed significantly worse at the Ekman test compared to HC in recognizing surprise ($p \leq 0.01$). all other non-sig (happiness, sadness, disgust, fear, anger).

Tags: Emotion recognition, behavioral

Reduced Modulation of Theta and Beta Oscillations Mediates Empathy Impairment in Parkinson's Disease.

Item Type Journal Article

Author Jinying Han
Author Liuzhenxiong Yu
Author Mengqi Wang
Author Xin Chen
Author Ziye Zhao
Author Pingping Liu
Author Lili Hu
Author Lingling Lv
Author Fengbo Xing
Author Ruihua Cao
Author Rong Ye
Author Kai Wang
Author Panpan Hu
Abstract BACKGROUND: Empathy is an inaccessible part of advanced social cognitive functions in humans. Impairment of empathy greatly affects the quality of life of patients with Parkinson's disease (PD) but the underlying neurophysiologic mechanisms have not been established. OBJECTIVES: The dynamic process of brain oscillations in PD pain empathy was explored and the mechanism of empathy damage was studied. METHODS: A total of 27 patients with PD and 13 healthy controls were recruited to undergo a pain judgment task, and the event-related potentials were recorded. This study compared the changes in theta and beta oscillations among two groups after the presentation of painful and neutral stimuli. RESULTS: Time-frequency analysis results revealed that patients with PD exhibited event-related theta oscillation synchronization and beta oscillation desynchronization during pain empathy. Compared to healthy controls, patients with PD exhibited a reduced magnitude of beta oscillation desynchronization in response to painful stimuli and attenuated synchronization of theta oscillations induced by neutral stimuli. There are abnormal beta power differences between painful and neutral stimuli, while no differences were found in theta power in PD. Moreover, a positive correlation existed between the degree of beta oscillation desynchronization associated with painful stimuli and the accuracy of pain judgments. CONCLUSION: Pain empathy deficits in PD were associated with reduced dynamic modulation of brain theta and beta oscillations.
Date 2025 Feb
Language eng
License © 2025 The Author(s). Brain and Behavior published by Wiley Periodicals LLC.
Extra Place: United States
Volume 15
Pages e70294
Publication Brain and behavior
DOI 10.1002/brb3.70294
Issue 2
Journal Abbr Brain Behav
ISSN 2162-3279
PMID 39957064
PMCID PMC11830629
Date Added 6.7.2025, 19:09:39
Modified 5.9.2025, 14:37:35

Notes:

Included

sample characteristics

size: 27 PD and 13 HC

Parkinson's Disease type and duration: idiopathic PD, Mduration = 2.61 SD= 2.50

Medication: NA

Hoehn-Yahr: Md=1.5 (1-2)

UPDRS-3: M= 21.55 SD= 9.21

Gender (male): 18 males (67%)

averaged ages (SD, range): M= 59.18 SD= 8.93

other neurological disease (tumor, stroke, etc.): None

other major psychopathology: None

origin country (or ethnicity): China

method observational

instruments used in order to quantify the variables

Social cognition aspect: empathy

Name of the task: The pain empathy task

type of stimulus [face/voice etc., Ekman faces/other etc.]: participants were asked to determine whether the person in the picture was experiencing pain. The visual stimulation in the task involves first-person vision, exclusively showcasing the limbs in either a painful or neutral state while omitting facial features to focus the participants' attention on the limb interactions. The stimuli consisted of photographs depicting scenarios involving left or right limbs subjected to different forms of potential harm. Specifically, these included images of limbs being pricked by needles, glass either piercing or not piercing the skin, and doors pinching or not pinching hands. For example, one of the stimulus images featured a knife either piercing or not piercing a hand

task condition: pain/neutral, right/left

operationalization: make a judgment regarding the nature of the stimulus (painful or neutral) by pressing designated left or right buttons on a response device. Accuracy and RT

Main findings related to the review's scope

Accuracy: accuracy of painful stimuli in PD patients was significantly lower than that of neutral stimuli. However, there was no significant difference in judgment accuracy in HCs

RT: no sig effects

Tags: empathy, behavioral

Relating anatomical and social connectivity: white matter microstructure predicts emotional empathy.

Item Type Journal Article
Author Carolyn Parkinson
Author Thalia Wheatley
Abstract Understanding cues to the internal states of others involves a widely distributed network of brain regions. Although white matter (WM) connections are likely crucial for communication between these regions, the role of anatomical connectivity in empathic processing remains unexplored. The present study tested for a relationship between anatomical connectivity and empathy by assessing the WM microstructural correlates of affective empathy, which promotes interpersonal understanding through emotional reactions, and cognitive empathy, which does so via perspective taking. Associations between fractional anisotropy (FA) and the emotional (empathic concern, EC) and cognitive (perspective taking, PT) dimensions of empathy as assessed by the Interpersonal Reactivity Index were examined. EC was positively associated with FA in tracts providing communicative pathways within the limbic system, between perception and action-related regions, and between perception and affect-related regions, independently of individual differences in age, gender, and other dimensions of interpersonal reactivity. These findings provide a neuroanatomical basis for the rapid, privileged processing of emotional sensory information and the automatic elicitation of responses to the affective displays of others.
Date 2014 Mar
Language eng
Extra Place: United States
Volume 24
Pages 614-625
Publication Cerebral cortex (New York, N.Y. : 1991)
DOI 10.1093/cercor/bhs347
Issue 3
Journal Abbr Cereb Cortex
ISSN 1460-2199 1047-3211
PMID 23162046
Date Added 6.7.2025, 19:09:42
Modified 5.9.2025, 14:49:13

Notes:

Not Included: No PD group

Interestion study though!

Tags: EXCLUDED

[Relationship between severity of hypomimia and basic emotion recognition in Parkinson's disease].

**Item Type**	Journal Article
**Author**	F. Cossini
**Author**	C. Cuesta
**Author**	K. Román
**Author**	S. Zambrano
**Author**	W. Rubinstein
**Author**	D. Politis
**Abstract**	INTRODUCTION: Parkinson's disease is characterised by the presence of motor symptoms including hypomimia, and by non-motor symptoms including alterations in facial recognition of basic emotions. Few studies have investigated this alteration and its relationship to the severity of hypomimia. OBJECTIVE: The objective is to study the relationship between hypomimia and the facial recognition of basic emotions in subjects with Parkinson's disease. SUBJECTS AND METHODS: Twenty-three patients and 29 controls were evaluated with the test battery for basic emotion facial recognition. The patients were divided into two subgroups according to the intensity of their hypomimia. RESULTS: The comparison in battery test performance between the minimal/mild hypomimia and moderate/severe hypomimia groups was statistically significant in favour of the former group. CONCLUSIONS: This finding shows a close relationship between expression and facial recognition of emotions, which could be explained through the mechanism of motor simulation.
**Date**	2024 Aug 1
**Language**	spa
**Extra**	Place: Singapore
**Volume**	79
**Pages**	71-76
**Publication**	Revista de neurologia
**DOI**	10.33588/rn.7903.2024169
**Issue**	3
**Journal Abbr**	Rev Neurol
**ISSN**	1576-6578 0210-0010
**PMID**	39007858
**PMCID**	PMC11469093
**Date Added**	6.7.2025, 19:09:35
**Modified**	5.9.2025, 14:31:29

Notes:

Not Included: not in English

Tags: EXCLUDED

Reliability and validity of a videotape method to describe expressive behavior in persons with Parkinson's disease

Item Type Journal Article
Author KD Lyons
Author L Tickle-Degnen
Abstract The ability to effectively communicate thoughts, feelings, and identity to others is an important aspect of occupational performance. The symptoms of Parkinson's disease can impair a person's ability to verbally and non-verbally communicate with others. In order to better understand issues of communication functioning for this population, research tools to describe expressive and communicative behavior during occupation and social interaction are needed. In this study, six persons with Parkinson's disease participated in individual, videotaped interviews focused on problem solving during daily activities. Three trained graduate students viewed edited clips from the videotapes and completed a rating scale of expressive behavior designed by the authors. Data support the reliability and construct validity of the behavioral rating scale, suggesting that measures of expressive behavior of persons with Parkinson's disease can be effectively derived using short segments of videotaped activity.
Date 2005-02
Language English
Extra Place: 4720 MONTGOMERY LANE, BETHESDA, MD 20814-3425 USA Type: Article
Volume 59
Publisher AMER OCCUPATIONAL THERAPY ASSOC, INC
Pages 41-49
Publication AMERICAN JOURNAL OF OCCUPATIONAL THERAPY
DOI 10.5014/ajot.59.1.41
Issue 1
ISSN 0272-9490
Date Added 14.7.2025, 14:50:43
Modified 14.7.2025, 14:50:43

Notes:

Not Included: No SC aspect was examined
Tags: EXCLUDED

Research on Emotion Recognition and Dementias: Foundations and Prospects

Item Type Journal Article
Author Gregorio Gonzalez-Alcaide
Author Mercedes Fernandez-Rios
Author Rosa Redolat
Author Emilia Serra
Abstract Background: The study of emotion recognition could be crucial for detecting alterations in certain cognitive areas or as an early sign of neurological disorders. Objective: The main objective of the study is to characterize research development on emotion recognition, identifying the intellectual structure that supports this area of knowledge, and the main lines of research attracting investigators' interest. Methods: We identified publications on emotion recognition and dementia included in the Web

of Science Core Collection, analyzing the scientific output and main disciplines involved in generating knowledge in the area. A co-citation analysis and an analysis of the bibliographic coupling between the retrieved documents elucidated the thematic orientations of the research and the reference works that constitute the foundation for development in the field. Results: A total of 345 documents, with 24,282 bibliographic references between them, were included. This is an emerging research area, attracting the interest of investigators in Neurosciences, Psychology, Clinical Neurology, and Psychiatry, among other disciplines. Four prominent topic areas were identified, linked to frontotemporal dementia, autism spectrum disorders, Alzheimer's disease, and Parkinson's and Huntington disease. Many recent papers focus on the detection of mild cognitive impairment. Conclusion: Impaired emotion recognition may be a key sign facilitating the diagnosis and early treatment of different neurodegenerative diseases as well as for triggering the necessary provision of social and family support, explaining the growing research interest in this area.

Date 2021
Language English
Extra Place: NIEUWE HEMWEG 6B, 1013 BG AMSTERDAM, NETHERLANDS Type: Article
Volume 82
Publisher IOS PRESS
Pages 939-950
Publication JOURNAL OF ALZHEIMERS DISEASE
DOI 10.3233/JAD-210096
Issue 3
ISSN 1387-2877
Date Added 14.7.2025, 14:50:31
Modified 5.9.2025, 14:36:55

Notes:

<u>**Not Included:**</u> not on PD

Tags: EXCLUDED

Review of automated emotion-based quantification of facial expression in Parkinson's patients

Item Type Journal Article

Author Bhakti Sonawane
Author Priyanka Sharma

Abstract Among various means of communication, the human face is utmost powerful. Persons suffering from Parkinson's disease (PD) experience hypomimia which often leads to reduction in facial expression. Hypomimia affects in social interaction and has a highly undesirable impact on patient's as well as his relative's quality of life. To track the longitudinal progression of PD, usually Movement Disorder Society's Unified Parkinson's Disease Rating Scale (MDS-UPDRS) is used in clinical studies and item 3.2 (i.e., facial expression) of MDS-UPDRS defines hypomimia levels. Assessment of facial expressions has traditionally relied on an observer-based scale

which can be time-consuming. Computational analysis techniques for facial expressions can assist the clinician in decision making. Intention of such techniques is to predict objective and accurate score for facial expression. The aim of this paper is to present up-to-date review on computational analysis techniques for measurement of emotional facial expression of people with PD (PWP) along with an overview on clinical applications of automated facial expression analysis. This led us to examine a pilot experimental work for masked face detection in PD. For the same, a deep learning-based model was trained on NVIDIA GeForce 920M GPU. It was observed that deep learning-based model yields 85% accuracy on the testing images.

Date 2021-05
Language English
Extra Place: ONE NEW YORK PLAZA, SUITE 4600, NEW YORK, NY, UNITED STATES Type: Review
Volume 37
Publisher SPRINGER
Pages 1151-1167
Publication VISUAL COMPUTER
DOI 10.1007/s00371-020-01859-9
Issue 5
ISSN 0178-2789
Date Added 14.7.2025, 14:50:32
Modified 5.9.2025, 14:57:18

Notes:

Not Included: not a systematic Review

Tags: EXCLUDED

Role of clinical neuropsychology in deep brain stimulation: Review of the literature and considerations for clinicians

Item Type Journal Article
Author Joseph A. Mole
Author Simon J. Prangnell
Date 2019-05-04
Language en
Short Title Role of clinical neuropsychology in deep brain stimulation
Library Catalog DOI.org (Crossref)
URL https://www.tandfonline.com/doi/full/10.1080/23279095.2017.1407765
Accessed 18.1.2026, 22:56:15
Volume 26
Pages 283-296
Publication Applied Neuropsychology: Adult
DOI 10.1080/23279095.2017.1407765
Issue 3

Journal Abbr Applied Neuropsychology: Adult
ISSN 2327-9095, 2327-9109
Date Added 18.1.2026, 22:56:15
Modified 18.1.2026, 22:56:15

Sad and happy facial emotion recognition impairment in progressive supranuclear palsy in comparison with Parkinson's disease.

Item Type Journal Article
Author Francesco E. Pontieri
Author Francesca Assogna
Author Alessandro Stefani
Author Mariangela Pierantozzi
Author Giuseppe Meco
Author Dario Benincasa
Author Carlo Colosimo
Author Carlo Caltagirone
Author Gianfranco Spalletta
Abstract The severity of motor and non-motor symptoms of progressive supranuclear palsy (PSP) has a profound impact on social interactions of affected individuals and may, consequently, contribute to alter emotion recognition. Here we investigated facial emotion recognition impairment in PSP with respect to Parkinson's disease (PD), with the primary aim of outlining the differences between the two disorders. Moreover, we applied an intensity-dependent paradigm to examine the different threshold of encoding emotional faces in PSP and PD. The Penn emotion recognition test (PERT) was used to assess facial emotion recognition ability in PSP and PD patients. The 2 groups were matched for age, disease duration, global cognition, depression, anxiety, and daily L-Dopa intake. PSP patients displayed significantly lower recognition of sad and happy emotional faces with respect to PD ones. This applied to global recognition, as well as to low-intensity and high-intensity facial emotion recognition. These results indicate specific impairment of recognition of sad and happy facial emotions in PSP with respect to PD patients. The differences may depend upon diverse involvement of cortical-subcortical loops integrating emotional states and cognition between the two conditions, and might represent a neuropsychological correlate of the apathetic syndrome frequently encountered in PSP.
Date 2012 Aug
Language eng
License Copyright © 2012 Elsevier Ltd. All rights reserved.
Extra Place: England
Volume 18
Pages 871-875
Publication Parkinsonism & related disorders
DOI 10.1016/j.parkreldis.2012.04.023
Issue 7
Journal Abbr Parkinsonism Relat Disord
ISSN 1873-5126 1353-8020

PMID 22595619
Date Added 6.7.2025, 19:09:35
Modified 5.9.2025, 14:52:09

Notes:

Included

Sample characteristics

Size: 176 PD, 19 PSP

PD-type: NA

PD-duration: M = 3.9, SD = 2.7

Medication: ON state

Hoehn-Yahr: NA

UPDRS-3: NA

Gender (male): 107 (60%)

Age: M = 68.2, SD = 7.0

Other neurological disease (tumor, stroke, etc.): none

Other major psychopathology: none

Origin country (or ethnicity): Italy

method (Review, meta-analysis or observational and/or self-reported):

instruments used in order to quantify the variables

Social cognition aspect: Facial Emotion Recognition

Name of the task: Penn Emotion Recognition Test (PERT)

Type of stimulus [face/voice etc., Ekman faces/other etc.]: digitized high-quality pictures of 3-dimensional (3D) facial expressions of evoked or felt emotions and non-emotional or neutral expressions. 96 color photographs of facial expressions of five emotions (happiness, sadness, anger, fear, disgust) and neutral faces. There are 8 low-intensity and 8 high-intensity expressions for each emotion and neutral expressions. Across emotional categories, stimuli are balanced for poser's gender and ethnicity. There are 48 male and 48 female faces, 59 of Caucasian people, and 37 of non-Caucasian people

Task condition: label the basic emotions (6 way forced choice (anger, happiness, sadness, fear, disgust, neutral)

Operationalization: Correct answers

Total score for each emotion, "low-intensity score", "high-intensity score",
Three "global emotions" scores:

1. sum of the five emotion total socre
2. sum of the five emotion total scores when emotions were of low-level intensity
3. sum of the five emotion total scores for high-level intensity emotions

Main findings related to the review's scope

A MANOVA using total scores of each facial emotion as dependent variables indicated that the two diagnostic groups differed significantly in the recognition of facial emotion expressions. A series of

follow-up ANOVAs revealed that PSP patients were significantly impaired in the recognition of facial emotions expressing happiness and sadness.

The two diagnostic groups differed in the recognition of facial emotion expressions of high intensity. A series of follow-up ANOVAs revealed that PSP patients were significantly impaired in the recognition of facial emotions expressing happiness and sadness of high intensity

Tags: Emotion Recognition, behavioral

Selective attention and facial expression recognition in patients with Parkinson's disease.

**Item Type**	Journal Article
**Author**	Laura Alonso-Recio
**Author**	Juan M. Serrano
**Author**	Pilar Martín
**Abstract**	Parkinson's disease (PD) has been associated with facial expression recognition difficulties. However, this impairment could be secondary to the one produced in other cognitive processes involved in recognition, such as selective attention. This study investigates the influence of two selective attention components (inhibition and visual search) on facial expression recognition in PD. We compared facial expression and non-emotional stimuli recognition abilities of 51 patients and 51 healthy controls, by means of an adapted Stroop task, and by "The Face in the Crowd" paradigm, which assess Inhibition and Visual Search abilities, respectively. Patients scored worse than controls in both tasks with facial expressions, but not with the other nonemotional stimuli, indicating specific emotional recognition impairment, not dependent on selective attention abilities. This should be taken into account in patients' neuropsychological assessment given the relevance of emotional facial expression for social communication in everyday settings.
**Date**	2014 Jun
**Language**	eng
**License**	© The Author 2014. Published by Oxford University Press. All rights reserved. For permissions, please e-mail: journals.permissions@oup.com.
**Extra**	Place: United States
**Volume**	29
**Pages**	374-384
**Publication**	Archives of clinical neuropsychology : the official journal of the National Academy of Neuropsychologists
**DOI**	10.1093/arclin/acu018
**Issue**	4
**Journal Abbr**	Arch Clin Neuropsychol
**ISSN**	1873-5843 0887-6177
**PMID**	24760956
**Date Added**	6.7.2025, 19:09:36
**Modified**	5.9.2025, 14:26:04

Notes:

Included

sample characteristics

size: 51 PD and 51 HC did not significantly differ for sex, age, educational level, and general cognitive abilities

Parkinson's Disease type and duration: idiopathic PD, Mduration= 6.32 years (*SD* = 3.87)

Medication: on medication

Hoehn-Yahr: Md=2 1-4

UPDRS-3: NA

Gender (male): 20 males (39%)

averaged ages (SD, range): M= 64.72 SD=3.98

other neurological disease (tumor, stroke, etc.): None

other major psychopathology: None

origin country (or ethnicity): Spain

method observational

instruments used in order to quantify the variables

Social cognition aspect: emotion recognition

Name of the task: NA, Inhibition task

type of stimulus [face/voice etc., Ekman faces/other etc.]: "Stroop effect" paradigm, one with emotional facial expressions (EFE)-word and one with Color-word. For the EFE-word set, 40 pictures of males and females faces (20 men) showing happy, anger, fear, disgust, or sadness (eight faces for each emotion) were selected from the FACES Database. The hair and background was removed from all pictures, in order to eliminate insignificant or distracting information for EFE recognition.

task condition: four congruent stimuli (the emotion category name corresponded to the EFE shown) and four with an incongruent superimposed name (not corresponding to the EFE shown).

operationalization: identify the EFE and to ignore the superimposed emotion category name >> RT and accuracy

Name of the task: NA, Visual search task

type of stimulus [face/voice etc., Ekman faces/other etc.]: the "a face-in-the-crowd effect" paradigm. 30 pictures of 15 different male and 15 female faces displaying happiness, anger, fear, disgust, and sadness (six examples of each EFE) were selected from the FACES Database.

task condition: Half of the trials comprised 24 copies of the same example (target-absent trials), and the remaining were composed of 23 copies of the same EFE plus another one showing a different EFE (target trials).

operationalization: Participants were instructed to decide whether all the 24 faces showed the same EFE or whether any displayed a different one. >> RT and accuracy

Main findings related to the review's scope

Inhibition Task

Accuracy-significant difference when comparing PD and HC scores for EFE-word; but not for Color-word. PD patients were less accurate in their responses than the HC group in EFE-word

Reaction times-Neither a significant Group × Stimuli interaction nor a significant main effect of were observed.

Visual Search Task

Accuracy- significant difference when comparing PD and HC scores for EFE crowd but not for Nonemotional crowd. In EFE crowd, PD patients' mean accuracy score was below that of the HC group.

Reaction times- Neither a significant Group × Stimuli interaction nor a main effect of Group

Tags: Emotion recognition, behavioral

Selective impairment of emotion recognition through music in Parkinson's disease: does it suggest the existence of different networks for music and speech prosody processing?

**Item Type**	Journal Article
**Author**	Tobias A. Mattei
**Author**	Abraham H. Rodriguez
**Author**	Juri Bassuner
**Date**	2013
**Language**	eng
**Extra**	Place: Switzerland
**Volume**	7
**Pages**	161
**Publication**	Frontiers in neuroscience
**DOI**	10.3389/fnins.2013.00161
**Journal Abbr**	Front Neurosci
**ISSN**	1662-4548 1662-453X
**PMID**	24062634
**PMCID**	PMC3771238
**Date Added**	6.7.2025, 19:09:38
**Modified**	5.9.2025, 14:45:53

Notes:

Not Included: No study, but a comment
Tags: EXCLUDED

Self-reported and experimentally induced self-disgust is heightened in Parkinson's disease: Contribution of behavioural symptoms.

**Item Type**	Journal Article
**Author**	Marianna Tsatali
**Author**	Paul G. Overton
**Author**	Ana B. Vivas
**Abstract**	Parkinson's disease (PD) is associated with deficits in the recognition and expression of basic emotions, although self-reported levels of the self-conscious emotions shame and embarrassment are higher. However, one self-conscious emotion-self-disgust-which has been shown to have a negative impact on psychological wellbeing, has not been investigated in PD before. Here we employed self-report measures of self-conscious emotions, and an emotion induction paradigm involving images of the self, and narrated personal vignettes of instances when patients with PD (and controls) found themselves disgusting. We found that self-reported and induced levels of self-disgust were higher in PD patients than in matched controls, and that trait self-disgust was specifically related to disorders of impulse control in PD patients. Given the link between self-disgust and impaired psychological wellbeing, and the prevalence of anxiety and depression in PD, self-disgust might make a useful therapeutic target for psychological interventions in the condition.
**Date**	2019
**Language**	eng
**Extra**	Place: United States
**Volume**	14
**Pages**	e0223663
**Publication**	PloS one
**DOI**	10.1371/journal.pone.0223663
**Issue**	10
**Journal Abbr**	PLoS One
**ISSN**	1932-6203
**PMID**	31618239
**PMCID**	PMC6799866
**Date Added**	6.7.2025, 19:09:38
**Modified**	5.9.2025, 14:59:04

Notes:

Not Included: not on SC

Tags: EXCLUDED

Sex Differences in Brain and Cognition in de novo Parkinson's Disease

**Item Type**	Journal Article
**Author**	Javier Oltra
**Author**	Carme Uribe
**Author**	Anna Campabadal
**Author**	Anna Inguanzo
**Author**	Gemma C. Monté-Rubio
**Author**	Maria J. Martí
**Author**	Yaroslau Compta
**Author**	Francesc Valldeoriola
**Author**	Carme Junque
**Author**	Barbara Segura
**Abstract**	Background and Objective: Brain atrophy and cognitive impairment in neurodegenerative diseases are influenced by sex. We aimed to investigate sex differences in brain atrophy and cognition in de novo Parkinson's disease (PD) patients.Methods: Clinical, neuropsychological and T1-weighted MRI data from 205 PD patients (127 males: 78 females) and 69 healthy controls (40 males: 29 females) were obtained from the PPMI dataset.Results: PD males had a greater motor and rapid eye movement sleep behavior disorder symptomatology than PD females. They also showed cortical thinning in postcentral and precentral regions, greater global cortical and subcortical atrophy and smaller volumes in thalamus, caudate, putamen, pallidum, hippocampus, and brainstem, compared with PD females. Healthy controls only showed reduced hippocampal volume in males compared to females. PD males performed worse than PD females in global cognition, immediate verbal recall, and mental processing speed. In both groups males performed worse than females in semantic verbal fluency and delayed verbal recall; as well as females performed worse than males in visuospatial function.Conclusions: Sex effect in brain and cognition is already evident in de novo PD not explained by age per se, being a relevant factor to consider in clinical and translational research in PD.
**Date**	2022-01-06
**Library Catalog**	Crossref
**URL**	https://www.frontiersin.org/articles/10.3389/fnagi.2021.791532/full
**Accessed**	13.7.2025, 20:22:34
**License**	https://creativecommons.org/licenses/by/4.0/
**Volume**	13
**Publisher**	Frontiers Media SA
**Publication**	Frontiers in Aging Neuroscience
**DOI**	10.3389/fnagi.2021.791532
**Journal Abbr**	Front. Aging Neurosci.
**ISSN**	1663-4365
**Date Added**	13.7.2025, 20:22:34
**Modified**	13.7.2025, 20:22:34

Notes:

Not Included: Study doesn’t measure SC
Tags: EXCLUDED

Sex, Age, and Emotional Valence: Revealing Possible Biases in the `Reading the Mind in the Eyes' Task

Item Type Journal Article
Author Jana Kynast
Author Matthias L. Schroeter
Abstract The `Reading the Mind in the Eyes' test (RMET) assesses a specific socio-cognitive ability, i.e., the ability to identify mental states from gaze. The development of this ability in a lifespan perspective is of special interest. Whereas former investigations were limited mainly to childhood and adolescence, the focus has been shifted towards aging, and psychiatric and neurodegenerative diseases recently. Although the RMET is frequently applied in developmental psychology and clinical settings, stimulus characteristics have never been investigated with respect to potential effects on test performance. Here, we analyzed the RMET stimulus set with a special focus on interrelations between sex, age and emotional valence. Forty-three persons rated age and emotional valence of the RMET picture set. Differences in emotional valence and age ratings between male and female items were analyzed. The linear relation between age and emotional valence was tested over all items, and separately for male and female items. Male items were rated older and more negative than female stimuli. Regarding male RMET items, age predicted emotional valence: older age was associated with negative emotions. Contrary, age and valence were not linearly related in female pictures. All ratings were independent of rater characteristics. Our results demonstrate a strong confound between sex, age, and emotional valence in the RMET. Male items presented a greater variability in age ratings compared to female items. Age and emotional valence were negatively associated among male items, but no significant association was found among female stimuli. As personal attributes impact social information processing, our results may add a new perspective on the interpretation of previous findings on interindividual differences in RMET accuracy, particularly in the field of developmental psychology, and age-associated neuropsychiatric diseases. A revision of the RMET might be afforded to overcome confounds identified here.
Date 2018-04-24
Language English
Extra Place: AVENUE DU TRIBUNAL FEDERAL 34, LAUSANNE, CH-1015, SWITZERLAND Type: Article
Volume 9
Publisher FRONTIERS MEDIA SA
Publication FRONTIERS IN PSYCHOLOGY
DOI 10.3389/fpsyg.2018.00570
ISSN 1664-1078
Date Added 14.7.2025, 14:50:34
Modified 5.9.2025, 14:42:40

Notes:

Not Included: Not on Parkinson
Tags: EXCLUDED

Social and non-social working memory in neurodegeneration.

**Item Type**	Journal Article
**Author**	Agustina Legaz
**Author**	Pavel Prado
**Author**	Sebastián Moguilner
**Author**	Sandra Báez
**Author**	Hernando Santamaría-García
**Author**	Agustina Birba
**Author**	Pablo Barttfeld
**Author**	Adolfo M. García
**Author**	Sol Fittipaldi
**Author**	Agustín Ibañez
**Abstract**	Although social functioning relies on working memory, whether a social-specific mechanism exists remains unclear. This undermines the characterization of neurodegenerative conditions with both working memory and social deficits. We assessed working memory domain-specificity across behavioral, electrophysiological, and neuroimaging dimensions in 245 participants. A novel working memory task involving social and non-social stimuli with three load levels was assessed across controls and different neurodegenerative conditions with recognized impairments in: working memory and social cognition (behavioral-variant frontotemporal dementia); general cognition (Alzheimer's disease); and unspecific patterns (Parkinson's disease). We also examined resting-state theta oscillations and functional connectivity correlates of working memory domain-specificity. Results in controls and all groups together evidenced increased working memory demands for social stimuli associated with frontocinguloparietal theta oscillations and salience network connectivity. Canonical frontal theta oscillations and executive-default mode network anticorrelation indexed non-social stimuli. Behavioral-variant frontotemporal dementia presented generalized working memory deficits related to posterior theta oscillations, with social stimuli linked to salience network connectivity. In Alzheimer's disease, generalized working memory impairments were related to temporoparietal theta oscillations, with non-social stimuli linked to the executive network. Parkinson's disease showed spared working memory performance and canonical brain correlates. Findings support a social-specific working memory and related disease-selective pathophysiological mechanisms.
**Date**	2023 Jul
**Language**	eng
**License**	Copyright © 2023. Published by Elsevier Inc.
**Extra**	Place: United States
**Volume**	183
**Pages**	106171
**Publication**	Neurobiology of disease
**DOI**	10.1016/j.nbd.2023.106171

Journal Abbr Neurobiol Dis
ISSN 1095-953X 0969-9961
PMID 37257663
PMCID PMC11177282
Date Added 6.7.2025, 19:09:33
Modified 5.9.2025, 14:43:20

Notes:

Not Included: Not assessing SC
Tags: EXCLUDED

Social brain dysfunctions in patients with Parkinson's disease: a review of theory of mind studies.

Item Type Journal Article
Author Rwei-Ling Yu
Author Ruey-Meei Wu
Abstract Human social interaction is essential in daily life and crucial for a promising life, especially in people who suffer from disease. Theory of Mind (ToM) is fundamental in social interaction and is described as the ability to impute the mental states of others in social situations. Studies have proposed that a complex neuroanatomical network that includes the frontal cortex mediates ToM. The primary neuropathology of Parkinson's disease (PD) involves the frontal-striatal system; therefore, patients with PD are expected to exhibit deficits in ToM. In this review, we summarize the current research with a particular focus on the patterns of impaired ToM, potential mediators of ToM, and the impact of ToM deficits on clinical disability in PD. Further studies to investigate the progression of ToM and its relationship with dementia in subjects in PD are needed.
Date 2013 Mar 28
Language eng
Extra Place: England
Volume 2
Pages 7
Publication Translational neurodegeneration
DOI 10.1186/2047-9158-2-7
Issue 1
Journal Abbr Transl Neurodegener
ISSN 2047-9158
PMID 23537376
PMCID PMC3621839
Date Added 6.7.2025, 19:09:41
Modified 5.9.2025, 15:02:02

Notes:

Not Included: not a systematic Review
Tags: EXCLUDED

Social Cognition and Cognitive Decline in Patients with Parkinson's Disease.

**Item Type**	Journal Article
**Author**	Laura Alonso-Recio
**Author**	Fernando Carvajal
**Author**	Carlos Merino
**Author**	Juan Manuel Serrano
**Abstract**	Social cognition (SC) comprises an array of cognitive and affective abilities such as social perception, theory of mind, empathy, and social behavior. Previous studies have suggested the existence of deficits in several SC abilities in Parkinson disease (PD), although not unanimously. OBJECTIVE: The aim of this study is to assess the SC construct and to explore its relationship with cognitive state in PD patients. METHOD: We compare 19 PD patients with cognitive decline, 27 cognitively preserved PD patients, and 29 healthy control (HC) individuals in social perception (static and dynamic emotional facial recognition), theory of mind, empathy, and social behavior tasks. We also assess processing speed, executive functions, memory, language, and visuospatial ability. RESULTS: PD patients with cognitive decline perform worse than the other groups in both facial expression recognition tasks and theory of mind. Cognitively preserved PD patients only score worse than HCs in the static facial expression recognition task. We find several significant correlations between each of the SC deficits and diverse cognitive processes. CONCLUSIONS: The results indicate that some components of SC are impaired in PD patients. These problems seem to be related to a global cognitive decline rather than to specific deficits. Considering the importance of these abilities for social interaction, we suggest that SC be included in the assessment protocols in PD.
**Date**	2021 Aug
**Language**	eng
**Extra**	Place: England
**Volume**	27
**Pages**	744-755
**Publication**	Journal of the International Neuropsychological Society : JINS
**DOI**	10.1017/S1355617720001204
**Issue**	7
**Journal Abbr**	J Int Neuropsychol Soc
**ISSN**	1469-7661 1355-6177
**PMID**	33243315
**Date Added**	6.7.2025, 19:09:33
**Modified**	5.9.2025, 14:25:40

Notes:

Included

sample characteristics

size: 28 cognitively intact PD patients (PD_CogInt), 19 PD patients with cognitive decline (PD_CogDec), and 27 HC

Parkinson's Disease type and duration: idiopathic PD, PD_CogInt Mduration= 5.36 SD= 5.26 PD_CogDec Mduration= 4.14 SD= 4.90

Medication: NA

Hoehn-Yahr: Md=2 both groups

UPDRS-3: NA

Gender (male): PD_CogInt 13 males (46%) PD_CogDec 9 males (47%)

averaged ages (SD, range): PD_CogInt M=68.68 SD=6.55 PD_CogDec M=72.84 SD=7.01

other neurological disease (tumor, stroke, etc.): None

other major psychopathology: None

origin country (or ethnicity): Spain

method observational

instruments used in order to quantify the variables

Social cognition aspect: emotion recognition

Name of the task: NA

type of stimulus [face/voice etc., Ekman faces/other etc.]: 50 static (photograph) and another with 50 dynamic (videos) stimuli. The photographs were selected from the FACES Database.

task condition: happiness, sadness, fear, anger, and neutral face

operationalization: Accuracy

Social cognition aspect: ToM

Name of the task: RMET

type of stimulus [face/voice etc., Ekman faces/other etc.]: 36 photographs of the eye region of the faces of male and female actors presented in different papers.

operationalization: Accuracy

Social cognition aspect: empathy

Name of the task: the Empathy Quotient

type of stimulus [face/voice etc., Ekman faces/other etc.]: self-report measure of empathy. 40 questions tapping empathy and 20 filler items to distract the participant from a relentless focus on empathy.

Responses are given on a 4-point scale ranging from “strongly agree” to “strongly disagree”, and approximately half of the items are reversed.

operationalization: Participants received 0 for a “nonempathic” response, whatever the magnitude, and 1 or 2 for an “empathic response” depending on the strength of the reply. The total score is out of 80.

Main findings related to the review's scope

Emotion recognition: PD_CogDec group scored lower than the HC and PD_CogInt.

no differences were found between HC and PD_CogInt

In the static task, both PD groups performed worse than HC. In the dynamic task, PD_CogDec performed worse than HC and PD_CogInt

Theory of mind: while PD_CogDec performed worse than HC, there were no differences between PD_CogInt and HC, or between PD_CogInt and PD_CogDec

Empathy: no differences among the groups

Tags: empathy, ToM, behavioral, Questionnaire

Social Cognition and Covid-19: a rapid scoping review

**Item Type**	Journal Article
**Author**	Tommaso Barlattani
**Author**	Simonetta Mantenuto
**Author**	Chiara D'amelio
**Author**	Arianna DI Berardo
**Author**	Francesco Capelli
**Author**	Valentina Leonardi
**Author**	Valentina Socci
**Author**	Rodolfo Rossi
**Author**	Alessandro Rossi
**Author**	Francesca Pacitti
**Date**	2024-12
**Volume**	59
**Pages**	279-289
**Publication**	RIVISTA DI PSICHIATRIA
**Issue**	6
**ISSN**	0035-6484
**Date Added**	14.7.2025, 14:48:38
**Modified**	5.9.2025, 14:27:41

Notes:

Not Included: not a systematic review
Tags: EXCLUDED

Social Cognition and Mild Cognitive Impairment in Mid-Stage Parkinson's Disease.

**Item Type**	Journal Article
**Author**	Roberto Fernández-Fernández
**Author**	Guillermo Lahera
**Author**	Beatriz Fernández-Rodríguez
**Author**	Pasqualina Guida
**Author**	Clara Trompeta
**Author**	David Mata-Marín
**Author**	Carmen Gasca-Salas
**Abstract**	Mild cognitive impairment (MCI) is a relevant non-motor feature in Parkinson's disease (PD). Social cognition (SC) is a cognitive domain that refers to the ability to decode others' intentions and to guide behavior in social contexts. We aimed to compare SC performance in mid-stage PD patients compared to a healthy population and according to their cognitive state. Fifty-two PD patients were classified as being cognitively normal (PD-CN) or having mild cognitive impairment (PD-MCI) following the Movement Disorder Society (MDS) Level II criteria. SC assessment included facial emotion recognition (FER), affective and cognitive theory of mind (ToM), and self-monitoring (RSMS test). Twenty-seven age-matched healthy controls (HC) were enrolled. PD-MCI patients scored worse than HC on affective and cognitive ToM task scores. Only cognitive ToM scores were significantly lower when compared with the PD-MCI and PD-CN groups. We found no differences in FER or self-monitoring performance. There were significant correlations between cognitive ToM and executive functions, memory, language, and attention, whereas FER and affective ToM correlated with memory. Our findings indicates that SC is normal in cognitively unimpaired and non-depressed mid-stage PD patients, whereas a decline in affective and cognitive ToM is linked to the presence of MCI.
**Date**	2024 Jan 29
**Language**	eng
**Extra**	Place: Switzerland
**Volume**	14
**Publication**	Behavioral sciences (Basel, Switzerland)
**DOI**	10.3390/bs14020101
**Issue**	2
**Journal Abbr**	Behav Sci (Basel)
**ISSN**	2076-328X
**PMID**	38392454
**PMCID**	PMC10885927
**Date Added**	6.7.2025, 19:09:33
**Modified**	5.9.2025, 14:35:41

Notes:

Included

sample characteristics

size: 52 PD ans 27 HC (age- and gender-matched)

Parkinson's Disease type and duration: NA, Mduration = 5.64 SD=2.09, max=10

Medication: on medication

Hoehn-Yahr: NA

UPDRS-3: M=20.35 sd=8.22

Gender (male): 37 males (71%)

averaged ages (SD, range): M=67.78 SD=5.29, min=50

other neurological disease (tumor, stroke, etc.): None

other major psychopathology: None

origin country (or ethnicity): Spain

method (Review, meta-analysis or observational and/or self-reported):

instruments used in order to quantify the variables

Social cognition aspect: emotion recognition

Name of the task: the Karolinska Directed Emotional Faces (KDEF) test

type of stimulus [face/voice etc., Ekman faces/other etc.]: 64 chosen photographs, 8 for each emotion

task condition: anger, disgust, fear, happiness, sadness, surprise and neutral

operationalization: accuracy

Social cognition aspect: Affective ToM

Name of the task: the Reading the Mind in The Eyes Test (RMET)

type of stimulus [face/voice etc., Ekman faces/other etc.]: eye region of male and female subjects.

operationalization: accuracy

Social cognition aspect: cognitive ToM

Name of the task: the Theory of Mind Picture Stories Task

type of stimulus [face/voice etc., Ekman faces/other etc.]: cartoon picture story consisting of four pictures that comprise a first-order false belief, a second-order false belief, and a tactical deception. The participants had to order the four pictures in the correct chronological sequence. If they failed, a correction was made, and the story was correctly presented before ToM testing.

operationalization: Participants were then asked about the story with questions about the ability to infer

the mental states of the characters in the story. Scores were classified into a maximum global rate of 59 points, a correct sequencing rate (maximum score of 36), and a correct questionnaire score (maximum score of 23); two points were given to the first and last cards and 1 point to the rest and for each question.

Social cognition aspect: regulated social behavior

Name of the task: the Revised Self-Monitoring Scale (RSMS)

type of stimulus [face/voice etc., Ekman faces/other etc.]: 13 items to be answered by the informant, covering the capacity to regulate one's (patient) behavior in a social context.

operationalization: 6-point Likert-type scale, and the patients are more likely to adapt to a social context when the score is higher.

Main findings related to the review's scope

thirty-three patients were classified as PD-CN, while nineteen were classified as PD-MCI.

In case of cognitive ToM, we found significant differences in ToM stories total scores ($F = 6.85$, $p < 0.05$) between PD-MCI groups (M = 44.5, SD = 10.76) compared to PD-CN (M = 50.5, SD = 9.07) and HC (M = 51.73, SD = 6.12). When we analyzed the ToM stories sequencing and questionnaire scores separately, only questionnaire scores remained statistically significant ($F = 11.14$, $p < 0.05$) for the same groups.

in the case of affective ToM, we observed significant differences in RMET test (F = 8.43, $p < 0.05$), but only between PD-MCI (M = 18.4, SD = 3.24) and HC (M = 22.4, SD = 4.59).

The analysis of the total KDEF score and analysis of each emotion individually showed no significant results ($F = 2.23$, $p = 0.32$).

RSMS - no sig dif.

Tags: Emotion recognition, ToM, social decision-making, behavioral, Questionnaire

---

Social cognition and the brain: A meta-analysis

Item Type Journal Article

Author Frank Van Overwalle

Abstract Abstract This meta-analysis explores the location and function of brain areas involved in social cognition, or the capacity to understand people's behavioral intentions, social beliefs, and personality traits. On the basis of over 200 fMRI studies, it tests alternative theoretical proposals that attempt to explain how several brain areas process information relevant for social cognition. The results suggest that inferring temporary states such as goals, intentions, and desires of other people —even when they are false and unjust from our own perspective—strongly engages the temporo-parietal junction (TPJ). Inferring more enduring dispositions of others and the self, or interpersonal norms and scripts, engages the medial prefrontal cortex (mPFC), although temporal states can also activate the mPFC. Other candidate tasks reflecting general-purpose brain processes that may

potentially subserve social cognition are briefly reviewed, such as sequence learning, causality detection, emotion processing, and executive functioning (action monitoring, attention, dual task monitoring, episodic memory retrieval), but none of them overlaps uniquely with the regions activated during social cognition. Hence, it appears that social cognition particularly engages the TPJ and mPFC regions. The available evidence is consistent with the role of a TPJ-related mirror system for inferring temporary goals and intentions at a relatively perceptual level of representation, and the mPFC as a module that integrates social information across time and allows reflection and representation of traits and norms, and presumably also of intentionality, at a more abstract cognitive level. Hum Brain Mapp, 2009. © 2008 Wiley-Liss, Inc.

Date 03/2009
Language en
Short Title Social cognition and the brain
Library Catalog DOI.org (Crossref)
URL https://onlinelibrary.wiley.com/doi/10.1002/hbm.20547
Accessed 19.1.2026, 7:26:28
License http://onlinelibrary.wiley.com/termsAndConditions#vor
Volume 30
Pages 829-858
Publication Human Brain Mapping
DOI 10.1002/hbm.20547
Issue 3
Journal Abbr Human Brain Mapping
ISSN 1065-9471, 1097-0193
Date Added 19.1.2026, 7:26:28
Modified 19.1.2026, 7:26:28

Social cognition and the human brain

Item Type Journal Article
Author Ralph Adolphs
Date 12/1999
Language en
Library Catalog DOI.org (Crossref)
URL https://linkinghub.elsevier.com/retrieve/pii/S1364661399013996
Accessed 19.1.2026, 7:18:57
License https://www.elsevier.com/tdm/userlicense/1.0/
Volume 3
Pages 469-479
Publication Trends in Cognitive Sciences
DOI 10.1016/S1364-6613(99)01399-6
Issue 12
Journal Abbr Trends in Cognitive Sciences
ISSN 13646613
Date Added 19.1.2026, 7:18:57

Modified 19.1.2026, 7:18:57

Social Cognition Dysfunctions in Neurodegenerative Diseases: Neuroanatomical Correlates and Clinical Implications.

Item Type Journal Article
Author Foteini Christidi
Author Raffaella Migliaccio
Author Hernando Santamaría-García
Author Gabriella Santangelo
Author Francesca Trojsi
Abstract Social cognitive function, involved in the perception, processing, and interpretation of social information, has been shown to be crucial for successful communication and interpersonal relationships, thereby significantly impacting mental health, well-being, and quality of life. In this regard, assessment of social cognition, mainly focusing on four key domains, such as theory of mind (ToM), emotional empathy, and social perception and behavior, has been increasingly evaluated in clinical settings, given the potential implications of impairments of these skills for therapeutic decision-making. With regard to neurodegenerative diseases (NDs), most disorders, characterized by variable disease phenotypes and progression, although similar for the unfavorable prognosis, are associated to impairments of social cognitive function, with consequent negative effects on patients' management. Specifically, in some NDs these deficits may represent core diagnostic criteria, such as for behavioral variant frontotemporal dementia (bvFTD), or may emerge during the disease course as critical aspects, such as for Parkinson's and Alzheimer's diseases. On this background, we aimed to revise the most updated evidence on the neurobiological hypotheses derived from network-based approaches, clinical manifestations, and assessment tools of social cognitive dysfunctions in NDs, also prospecting potential benefits on patients' well-being, quality of life, and outcome derived from potential therapeutic perspectives of these deficits.
Date 2018
Language eng
Extra Place: Netherlands
Volume 2018
Pages 1849794
Publication Behavioural neurology
DOI 10.1155/2018/1849794
Journal Abbr Behav Neurol
ISSN 1875-8584 0953-4180
PMID 29854017
PMCID PMC5944290
Date Added 6.7.2025, 19:09:33
Modified 5.9.2025, 14:30:50

Notes:

Not Included: not a systematic review
Tags: EXCLUDED

Social Cognition Impairments in Mice Overexpressing Alpha-Synuclein Under the Thy1 Promoter, a Model of Pre-manifest Parkinson's Disease.

**Item Type**	Journal Article
**Author**	Iddo Magen
**Author**	Eileen Ruth Torres
**Author**	Diana Dinh
**Author**	Andrew Chung
**Author**	Eliezer Masliah
**Author**	Marie-Françoise Chesselet
**Abstract**	BACKGROUND: Patients with Parkinson's disease (PD) may exhibit deficits in "Theory of Mind", the ability to read others' mental states and react appropriately, a prerequisite for successful social interaction. Alpha-synuclein overexpression is widely distributed in the brain of patients with sporadic PD, suggesting that it may contribute to the non-motor deficits observed in PD patients. Mice over-expressing human wild-type alpha-synuclein under the Thy1 promoter (Thy1-aSyn mice) have synaptic deficits in the frontostriatal pathway, low cortical acetylcholine, and high level of expression of mGluR5 receptors, which have all been implicated in social recognition deficits. OBJECTIVE: To determine whether Thy1-aSyn mice present alterations in their response to social stimuli. METHODS: We have submitted Thy1-aSyn mice to tests adapted from autism models. RESULTS: At 7-8 month of age Thy1-aSyn mice explored their conspecifics significantly less than did wild-type littermates, without differences in exploration of inanimate objects, and pairs of Thy1-aSyn mice were involved in reciprocal interactions for a shorter duration than wild-type mice at this age. These deficits persisted when the test animal was enclosed in a beaker and were not present at 3-4 months of age despite the presence of olfactory deficits at that age, indicating that they were not solely caused by impairment in olfaction. CONCLUSION: Thy1-aSyn mice present progressive deficits in social recognition, supporting an association between alpha-synuclein overexpression and Theory of Mind deficits in PD and providing a useful model for identifying mechanisms and testing novel treatments for these deficits which impact patients and caretakers quality of life.
**Date**	2015
**Language**	eng
**Extra**	Place: United States
**Volume**	5
**Pages**	669-680
**Publication**	Journal of Parkinson's disease
**DOI**	10.3233/JPD-140503
**Issue**	3
**Journal Abbr**	J Parkinsons Dis
**ISSN**	1877-718X 1877-7171
**PMID**	25588356

PMCID PMC5757648
Date Added 6.7.2025, 19:09:42
Modified 5.9.2025, 14:44:19

Notes:

Not Included: Animal model

Tags: EXCLUDED

[Social cognition impairments in Parkinson's disease].

Item Type Journal Article
Author E. A. Lyashenko
Author O. V. Iakovleva
Abstract Parkinson's disease is the second most common neurodegenerative disease of the elderly caused by the neurodegenerative process in different parts of the brain, which resulted in motor and non-motor symptoms. Investigation of non-motor symptoms of Parkinson's disease is increasingly rising for the last years. Social cognition is a special type of cognitive process, which provides people interaction in the society and their impairment also can be observed in Parkinson's disease. Social cognitive functions include many aspects: the theory of the mind, morality, personality changes and behavioral disorders. Each of these aspects is based on different neurophysiological, neurochemical and neuroanatomical substrates. This article is an effort to get closer to understanding of the changes, which occur in the brain of a patient with Parkinson's disease.
Date 2019
Language rus
Extra Place: Russia (Federation)
Volume 119
Pages 37-43
Publication Zhurnal nevrologii i psikhiatrii imeni S.S. Korsakova
DOI 10.17116/jnevro201911909237
Issue 9. Vyp. 2
Journal Abbr Zh Nevrol Psikhiatr Im S S Korsakova
ISSN 1997-7298
PMID 31825388
Date Added 6.7.2025, 19:09:34
Modified 5.9.2025, 14:44:10

Notes:

Not Included: Not in English

Tags: EXCLUDED

Social cognition in basal ganglia pathologies: Theory of Mind in Huntington's and Parkinson's diseases.

**Item Type**	Journal Article
**Author**	Sonia Di Tella
**Author**	Paola Zinzi
**Author**	Isabella Anzuino
**Author**	Maria Rita Lo Monaco
**Author**	Alice Tondinelli
**Author**	Marianna Magistri
**Author**	Martina Petracca
**Author**	Marcella Solito
**Author**	Paolo Calabresi
**Author**	Anna Rita Bentivoglio
**Author**	Maria Caterina Silveri
**Abstract**	Theory of Mind (ToM) is the ability to predict the behaviour of others by inferring their cognitive and affective states. The literature suggests that different neural substrates within the basal ganglia are involved in the affective (ventral striatum) and cognitive (dorsal striatum) components of ToM. We investigated ToM dysfunction in two different basal ganglia pathologies, Huntington's disease (HD) and Parkinson's disease (PD), in their early stages. Indeed, a different progression of neurodegeneration from the dorsal striatum to the ventral striatum is described in the two diseases. We also investigated whether there is a correlation between ToM and executive function. Twenty-one patients with HD, 21 with PD, and 22 healthy subjects (HS) were recruited. All participants completed a ToM assessment using the Yoni task, which assesses both cognitive and affective components at two levels of meta-representational difficulty (i.e. first-order items only require inferring the mental state of a person, while second-order items also require inferring the mental states of a person about others). The clinical groups also underwent a full neuropsychological assessment. In HD patients, both cognitive and affective ToM were equally impaired, whereas in PD patients, impairment of the cognitive component predominated. Specifically, compared to HS, HD patients scored lower on both inferential levels and on both cognitive and affective components, whereas PD patients scored lower than HS only on second-order and cognitive items. In the clinical groups, there was an imbalance between the cognitive and affective components, with higher accuracy on affective items. Performance on the Yoni task did not correlate with tests assessing executive functions. We suggest that the different pattern of ToM alteration in HD and PD may be a result of differential involvement of the ventral and dorsal striatum and that ToM abilities in these clinical populations are not directly supported by executive functioning.
**Date**	2025 Feb 20
**Language**	eng
**License**	© The Author(s) 2025. Published by Oxford University Press.
**Extra**	Place: England
**Volume**	20
**Publication**	Social cognitive and affective neuroscience
**DOI**	10.1093/scan/nsaf007
**Issue**	1
**Journal Abbr**	Soc Cogn Affect Neurosci

ISSN 1749-5024 1749-5016
PMID 39948742
PMCID PMC11840954
Date Added 6.7.2025, 19:09:34
Modified 5.9.2025, 14:33:29

Notes:

Included

sample characteristics

size: 21 PD, 21 Huntington's Disease (HD), and 22 HC (matched for gender)

Parkinson's Disease type and duration: Mduration = 8.05 SD=3.73

Medication: on medication

Hoehn-Yahr: NA

UPDRS-3: M= 25.48 SD=11.29

Gender (male): 12 (57.1%)

averaged ages (SD, range): M= 71.86 SD= 6.00

other neurological disease (tumor, stroke, etc.): None

other major psychopathology: None

origin country (or ethnicity): Italy

method observational

instruments used in order to quantify the variables

Social cognition aspect: ToM

Name of the task: the Yoni task

type of stimulus [face/voice etc., Ekman faces/other etc.]: 98 items, 84 mental items, and 14 physical items (control items), in which the subject is shown a stylized face in the centre of the screen (named 'Yoni [Gianni]') and four coloured images in the corners of the screen. These pictures can refer to faces or to different semantic categories, such as fruits, animals, vehicles, and so on. Based on the sentence at the top of the screen and cues such as Yoni's eye gaze or facial expression, the participant must select the correct image to which Yoni is referring

task condition: the items differ in terms of the component of ToM involved (affective ToM—'Yoni loves…'; cognitive ToM—'Yoni is thinking…'; physical control condition—'Yoni is close to…') and the difficulty of the meta-representations, which can be first-order ('Yoni is thinking of/loves…') or second-order ('Yoni is thinking of/loves … that … wants/loves') (Rossetto et al. 2018). There is only one correct answer.

operationalization: accuracy and response times (RTs).

Accuracy scores: first-order ToM (range 0–24), second-order ToM (range 0–60), affective ToM (range 0–48), and cognitive ToM (range 0–36), and total (summing first- and second-order items and dividing the sum by the total number of mental items >> total ToM = first-order ToM + second-order ToM/84). CA balance score = (affective ToM/48 − cognitive ToM/36)/(affective ToM/48 + cognitive ToM/36), where a value close to zero indicates a balance between affective and cognitive ToM, a positive index indicates a higher level of affective than cognitive ToM, and a negative index indicates a higher level of cognitive than affective ToM.

Main findings related to the review's scope

Accuracy:

Total score >> both HD and PD scoring lower than HC, and HD scoring lower than PD

PD performed significantly worse than HC only on second-order tasks, but not first-order

No differences emerged between the two clinical groups on control condition items.

cognitive ToM >> both clinical groups performing worse than HC

affective ToM >> HD showing lower accuracy than HC and PD

cognitive/affective accuracy (CA) balance score >> both clinical groups showing higher accuracy on affective than cognitive items compared to HC

Tags: ToM, behavioral

Social Cognition in Humans

**Item Type**	Journal Article
**Author**	Chris D. Frith
**Author**	Uta Frith
**Date**	08/2007
**Language**	en
**Library Catalog**	DOI.org (Crossref)
**URL**	https://linkinghub.elsevier.com/retrieve/pii/S0960982207014923
**Accessed**	19.1.2026, 7:25:25
**License**	https://www.elsevier.com/tdm/userlicense/1.0/
**Volume**	17
**Pages**	R724-R732
**Publication**	Current Biology
**DOI**	10.1016/j.cub.2007.05.068
**Issue**	16
**Journal Abbr**	Current Biology
**ISSN**	09609822
**Date Added**	19.1.2026, 7:25:25
**Modified**	19.1.2026, 7:25:25

Social Cognition in Parkinson's Disease after Focused Ultrasound Subthalamotomy: A Controlled Study.

**Item Type**	Journal Article
**Author**	Pasqualina Guida

Author Raúl Martínez-Fernández
Author Jorge U. Máñez-Miró
Author Marta Del Álamo
Author Guglielmo Foffani
Author Beatriz Fernández-Rodríguez
Author Mariana H. G. Monje
Author Ignacio Obeso
Author José A. Obeso
Author Carmen Gasca-Salas
Abstract Social cognition (SC) encompasses a set of cognitive functions that enable individuals to understand and respond appropriately to social interactions. Although focused ultrasound subthalamotomy (FUS-STN) effectively treats Parkinson's disease (PD) clinical motor features, its impact and safety on cognitive-behavioral interactions/interpersonal awareness are unknown. This study investigated the effects of unilateral FUS-STN on facial emotion recognition (FER) and affective and cognitive theory of mind (ToM) in PD patients from a randomized sham-controlled trial (NCT03454425). Subjects performed SC evaluation before and 4 months after the procedure while still under blind assessment conditions. The SC assessment included the Karolinska Directed Emotional Faces task for FER, the Reading the Mind in the Eyes (RME) test for affective ToM, and The Theory of Mind Picture Stories Task (ToM PST) (order, questions, and total score) for cognitive ToM. The active treatment group showed anecdotal-to-moderate evidence of no worsening in SC after FUS-STN. Anecdotal evidence for an improvement was recognized in the SC score changes, from baseline to post-treatment, for the active treatment group compared with sham for the RME, ToM PST order, ToM PST total, FER total, and recognition of fear, disgust, and anger. This study provides the first evidence that unilateral FUS-STN does not impair social cognitive abilities, indicating that it can be considered a safe treatment approach for this domain in PD patients. Furthermore, the results suggest FUS-STN may even lead to some improvement in social cognitive outcomes, which should be considered as a preliminary finding requiring further investigation with larger samples sizes. © 2024 International Parkinson and Movement Disorder Society.
Date 2024 Oct
Language eng
License © 2024 International Parkinson and Movement Disorder Society.
Extra Place: United States
Volume 39
Pages 1763-1772
Publication Movement disorders : official journal of the Movement Disorder Society
DOI 10.1002/mds.29945
Issue 10
Journal Abbr Mov Disord
ISSN 1531-8257 0885-3185
PMID 39140267
Date Added 6.7.2025, 19:09:33
Modified 5.9.2025, 14:37:22

Notes:

Not Included: only compare intervention vs non, with no HC
Tags: EXCLUDED

Social cognition in Parkinson's disease and functional movement disorders.

**Item Type**	Journal Article
**Author**	Maria Caterina Silveri
**Author**	Maria Rita Lo Monaco
**Author**	Alice Tondinelli
**Author**	Martina Petracca
**Author**	Paola Zinzi
**Author**	Serena Fragapane
**Author**	Gino Pozzi
**Author**	Francesco Pagnini
**Author**	Anna Rita Bentivoglio
**Author**	Sonia Di Tella
**Abstract**	INTRODUCTION: Functional movement disorders (FMD) can overlap with Parkinson's disease (PD), and distinguishing between the two clinical conditions can be complex. Framing social cognition (theory of mind) (TOM) disorder, attention deficit, and psychodynamic features of FMD and PD may improve diagnosis. METHODS: Subjects with FMD and PD and healthy controls (HC) were administered tasks assessing TOM abilities and attention. The psychodynamic hypothesis of conversion disorder was explored by a questionnaire assessing dissociative symptoms. A comprehensive battery of neuropsychological tasks was also administered to FMD and PD. RESULTS: Although both FMD and PD scored lower than HC on all TOM tests, significant correlations between TOM and neuropsychological tasks were found only in PD but not in FMD. Only PD showed a reduction in attentional control. Dissociative symptoms occurred only in FMD. DISCUSSION: Cognitive-affective disturbances are real in FMD, whereas they are largely dependent on cognitive impairment in PD. Attentional control is preserved in FMD compared to PD, consistent with the hypothesis that overload of voluntary attentional orientation may be at the basis of the onset of functional motor symptoms. On a psychodynamic level, the confirmation of dissociative symptoms in FMD supports the conversion disorder hypothesis. CONCLUSION: FMD and PD can be distinguished on an affective and cognitive level. At the same time, however, the objective difficulty often encountered in distinguishing between the two pathologies draws attention to how blurred the boundary between 'organic' and 'functional' can be.
**Date**	2024 Aug
**Language**	eng
**License**	© 2024. Fondazione Società Italiana di Neurologia.
**Extra**	Place: Italy
**Volume**	45
**Pages**	3775-3784
**Publication**	Neurological sciences : official journal of the Italian Neurological Society and of the Italian Society of Clinical Neurophysiology
**DOI**	10.1007/s10072-024-07452-5

Issue 8
Journal Abbr Neurol Sci
ISSN 1590-3478 1590-1874
PMID 38521891
Date Added 6.7.2025, 19:09:33
Modified 5.9.2025, 14:56:51

Notes:

Included

Sample characteristics

Size: 23 FMD (functional movement disorder), 23 PD, 20 HC)

PD-type: NA

PD-duration:

Medication: On state

Hoehn-Yahr: NA

UPDRS-3: M = 12, SD = 15.5

Gender (male): 15 (65.2%)

Age: M 0 72.04, SD = 6.91, Range = 56-84

Other neurological disease (tumor, stroke, etc.): NA

Other major psychopathology: NA

Origin country (or ethnicity): Italy

method (Review, meta-analysis or observational and/or self-reported):

instruments used in order to quantify the variables

Social cognition aspect: Affective ToM

Name of the task: Rading the min with the eye test (RMET)

Type of stimulus [face/voice etc., Ekman faces/other etc.]: 37 photographs (36 visual stimuli and one practical stimulus) of the eye region of the face of an actor or actress, each presented on a slide

Task condition: The participant is asked to indicate the word that describes the mental state expressed by the eyes, choosing from four adjectives

Operationalization: Correct answers

Social cognition aspect: cognitive ToM

Name of the task: Faux pas stories

Type of stimulus [face/voice etc., Ekman faces/other etc.]: 10 stories, five with faux pas and five without

Task condition: answer 4 questions (faux pas recognition question, reality question, inference question, gaffe motivation question

Operationalization: A point is awarded only if the subject answers questions 1 to 4 correctly

Main findings related to the review's scope

subjects with PD performed significantly worse than HC on the RMET

a between-group difference was also found for faux pas, with both PD and FMD performing significantly worse than HC

Tags: ToM, behavioral

[Social cognition in Parkinson's disease dementia and behavioral variant of frontotemporal dementia].

**Item Type**	Journal Article
**Author**	M. E. Tabernero
**Author**	F. Musich
**Author**	F. C. Cossini
**Author**	D. G. Politis
**Abstract**	AIM: To study the presence of alterations in social cognition in Parkinson's disease dementia (PDD) and behavioral variant frontotemporal dementia (bvFTD), to compare the levels of involvement, and to analyze their relevance as a tool to distinguish between both demential profile. SUBJECTS AND METHODS: For this purpose, 34 patients diagnosed with PDD, 21 men and 13 women, with an average age of 70 years and 8 years of education, and 26 patients diagnosed with bvFTD, 9 men and 17 women, with an average age of 68 years and 6 years of education, were assessed. The control group consisted of 30 healthy subjects, with a mean of 66 years of age and 8 of schooling. Reading the Mind in the Eyes was use as an emotional Theory of Mind (ToM) test; First-Order False Belief as a cognitive ToM test; Faux Pas as mixed test, considering their emotional (FPec) and cognitive (FPcc) components; and Iowa Gambling Task for social decision-making. RESULTS: Both groups of patients showed alterations in all tests compared to the control group. A significant difference between PDD and bvFTD was also observed for Reading the Mind in the Eyes test, Faux Pas total score, FPec and FPcc, with lower means and medians for PDD patients on all scores except for FPcc. CONCLUSIONS: This findings suggests that emotional ToM would be most affected PDD patients, while the use of cognitive ToM and social decision-making tests would be restrain tools to differentiate between a type of dementia or another.
**Date**	2017 Dec 16
**Language**	spa
**Extra**	Place: Singapore
**Volume**	65
**Pages**	539-545
**Publication**	Revista de neurologia
**Issue**	12
**Journal Abbr**	Rev Neurol
**ISSN**	1576-6578 0210-0010
**PMID**	29235616
**Date Added**	6.7.2025, 19:09:33
**Modified**	5.9.2025, 14:58:04

Notes:

Not Included: Article written in Spanish.
Tags: EXCLUDED

Social Cognition in Parkinson's Disease: A Case-Control Study.

Item Type Journal Article
Author Govinda Siripurapu
Author Bhawna Verma
Author Deblina Biswas
Author Anandapadmanabhan Reghu
Author Aayushi Vishnoi
Author Divya Madathiparambil Radhakrishnan
Author Arunmozhimaran Elavarasi
Author Anu Gupta
Author Venugopalan Yamuna Vishnu
Author Mamta Bhushan Singh
Author Rohit Bhatia
Author Manjari Tripathi
Author Achal Srivastava
Author Madakasira Vasantha Padma Srivastava
Author Roopa Rajan
Abstract BACKGROUND: Social cognition is the study of how people make sense of themselves and others. Impairment in several domains of social cognition is increasingly being recognized in Parkinson's disease (PD). OBJECTIVES: We aimed to study multiple domains of social cognition in Indian PD patients using a culturally appropriate, validated instrument. METHODS: We recruited 52 individuals with PD and 31 healthy volunteers (HV) and used the Social Cognition Rating Tools in Indian Setting (SOCRATIS) tool to assess theory of mind (ToM), attributional biases and social cue perception. Quality of life (QoL) was assessed using the PDQOL scale. RESULTS: Baseline characteristics were comparable between PD and HV. The mean (SD) FOT index (first order ToM index) was 0.86(0.18) in PD and 0.99(0.07) in HV [P < 0.001]. The PD group showed higher Externalizing Bias [EB, 4.42(3.91)], compared to HV [1.58(3.22), P = 0.001]. The mean (SD) Faux Pas Composite Index (FPCI ALT) was 0.69(0.09) in PD and 0.78(0.13) in HV [P < 0.001]. Social cognition indices were not associated with QoL in PD. Clinical parameters-age, gender, HAM-D, MOCA, education, levodopa equivalent daily dose of medication, number of PD drugs and trihexyphenidyl use did not predict social cognition. CONCLUSION: PD patients were less successful than age, gender matched controls in understanding social situations and other's thought processes and had higher tendency to attribute undesirable events to external causes. Deficits in social cognition did not impair the quality of life.
Date 2023 Mar
Language eng
License © 2022 International Parkinson and Movement Disorder Society.
Extra Place: United States
Volume 10

Pages 399-405
Publication Movement disorders clinical practice
DOI 10.1002/mdc3.13653
Issue 3
Journal Abbr Mov Disord Clin Pract
ISSN 2330-1619
PMID 36949784
PMCID PMC10026278
Date Added 6.7.2025, 19:09:33
Modified 5.9.2025, 14:57:02

Notes:

Not Included: Case study
Tags: EXCLUDED

Social cognition in Parkinson's disease.

Item Type Journal Article
Author S. J. G. Lewis
Author L. Ricciardi
Date 2021 Apr
Language eng
Extra Place: England
Volume 85
Pages 122-123
Publication Parkinsonism & related disorders
DOI 10.1016/j.parkreldis.2021.02.024
Journal Abbr Parkinsonism Relat Disord
ISSN 1873-5126 1353-8020
PMID 33640252
Date Added 6.7.2025, 19:09:33
Modified 5.9.2025, 14:43:25

Notes:

Not Included: Editorial letter
Tags: EXCLUDED

Social Cognition in Patients with Early-Onset Parkinson's Disease.

Item Type Journal Article

Author Ana Natalia Seubert-Ravelo
Author Ma Guillermina Yáñez-Téllez
Author María Lizbeth Lazo-Barriga
Author Alejandra Calderón Vallejo
Author Carlos Eduardo Martínez-Cortés
Author Adela Hernández-Galván

Abstract Social cognition (SC) deficits have been linked to Parkinson's disease (PD) but have been less well researched than general cognitive processes, especially in early-onset PD (EOPD), despite this population often having greater social and family demands. Most studies focus on recognition of facial emotion, theory of mind (ToM), and decision-making domains, with limited research reporting on social reasoning. The main objective of this work was to compare SC ability across four domains: emotional processing, social reasoning, ToM, and decision-making between patients with EOPD and healthy controls. Twenty-five nondemented patients with EOPD and 25 controls matched for sex, age, and educational level were enrolled. A battery that included six SC tests was administered to all study participants; a decision-making scale was completed by participants' partners. Statistically significant differences were found between patients with EOPD and controls in all subtests across the four SC domains studied. The EOPD group demonstrated worse performance on all tasks, with large effect sizes. Differences remained significant after adjusting for Montreal Cognitive Assessment (MoCA) test scores for all SC subtests except the decision-making scale and the Iowa gambling task. No significant correlations between SC and other clinical PD variables were found. Our study shows that patients with EOPD perform significantly below controls in multiple SC domains affecting recognition of facial emotion, social reasoning, ToM, and decision-making. Only decision-making seems to be mediated by overall cognitive ability. The confounding or contributing effect of other clinical PD variables should be studied further.

Date 2021
Language eng
License Copyright © 2021 Ana Natalia Seubert-Ravelo et al.
Extra Place: United States
Volume 2021
Pages 8852087
Publication Parkinson's disease
DOI 10.1155/2021/8852087
Journal Abbr Parkinsons Dis
ISSN 2090-8083 2042-0080
PMID 33505651
PMCID PMC7810525
Date Added 6.7.2025, 19:09:33
Modified 14.7.2025, 15:19:43

Notes:

Included

Sample characteristics

Size: 25 PD (early-onset PD; EOPD), 25 HC matched for age and education

PD-type: early-onset PD (before the age of 50)

PD-duration: M = 10.6, SD = 4.2

Medication: ON state

Hoehn-Yahr: M = 2.6, SD = 0.7

UPDRS-3: NA

Gender (male): 18 (72%)

Age: M = 56.2, SD = 5.36

Other neurological disease (tumor, stroke, etc.): None

Other major psychopathology: No MDD

Origin country (or ethnicity): Mexico

method behavioural

instruments used in order to quantify the variables

Name of the task: Social Cognition Battery (COGSOC)

seven subtests, of which only the following were relevant:

1. **emotion recognition (POFA)**

Type of stimulus [face/voice etc., Ekman faces/other etc.]: 6 Ekman faces printed in black and white in a half-letter size sheet

Task condition: anger, fear, happiness, sadness, and surprise plus a neutral expression

Operationalization: accuracy

2. **Social reasoning: Causal relationships comprehension A—causes and B—consequences**

Type of stimulus [face/voice etc., Ekman faces/other etc.]: Each part, A and B, consists of eight and six illustrations, respectively, depicting scenes representing simple actions involving a maximum of two characters, printed in color in a half-letter-sized sheet.

Operationalization: The participant is asked to verbally provide the most probable, logical, and immediate action that took place before (causes—part A) or after (consequences—part B) the scene.

scored according to a 3-point scale: 0 points when the answer has no causal connection with the scene; 1 point when the causal relation is not immediate or is unlikely; and 2 points when the answer reflects a logical, immediate, and probable relation to the scene.

3. **Social reasoning - Absurdity identification**

Type of stimulus [face/voice etc., Ekman faces/other etc.]: six illustrations, each printed in color in a letter-sized sheet. Each illustration contains a scenario with three to five absurdities that sum a total of 23 items.

Operationalization: without a time limit, each scenario and point out what is absurd, illogical, or incongruent. The total score is the total number of absurdities correctly identified and thus ranges 0–23. It should be noted that participants must search without any verbal or physical cues from the evaluator scene, in which some of the absurdities are not centrally positioned; therefore, this subtest has a higher visual scanning demand in comparison to other subtests in the battery.

4. **Social reasoning - Social judgment ability**

Type of stimulus [face/voice etc., Ekman faces/other etc.]: 11 different social problems are represented visually, each using an illustration printed in color in a letter-sized sheet. Each illustration is accompanied by a verbal statement given by the evaluator, which specifies the problem and states a question. Given that, in some illustrations, more than one character can be involved in the scene, and the complementary question is necessary to inquire about the actions of a specific character.

Operationalization: The textual answer is registered for each of the 11 items and then scored according to a 3-point scale: 0 points when the proposed action is inconvenient and illogical or does not solve or further complicates the problem, 1 point when the action partially solves the problem or implies certain risk, and 2 points when the action offers a viable, correct, and safe solution to the problem. The test includes a guide with common answers for each answer level (0–2) to facilitate scoring. The total subtest score ranges from 0 to 22.

5. **ToM - RME test revised**

Type of stimulus [face/voice etc., Ekman faces/other etc.]: 36 pictures of the eye region of human faces printed in black and white, 19 corresponding to men and 17 to women.

Operationalization: accuracy

Main findings related to the review's scope

The patients with EOPD demonstrated worse performance in all subtests measuring SC domains, compared to HC.

Compared to HC, The EOPD group had a significantly higher misidentification rate when presented with a neutral face, a face expressing happiness, and a face expressing surprise. no significant differences were found regarding anger, fear, or sadness.

Tags: emotion recognition, ToM, social decision-making, behavioral

---

Social cognition of indirect speech: Evidence from Parkinson's disease

Item Type Journal Article
Author Patrick McNamara
Author Thomas Holtgraves
Author Raymon Durso
Author Erica Harris
Abstract We examined potential neurocognitive mechanisms of indirect speech in support of face management in 28 patients with Parkinson's disease (PD) and 32 elderly controls with chronic disease. In Experiment 1, we demonstrated automatic activation of

indirect meanings of particularized implicatures in controls but not in PD patients. Failure to automatically engage comprehension of indirect meanings of indirect speech acts in PD patients was correlated with a measure of prefrontal dysfunction. In Experiment 2, we showed that while PD patients and controls offered similar interpretations of indirect speech acts, PD participants were overly confident in their interpretations and unaware of errors of interpretation. Efficient reputational adjustment mechanisms apparently require intact striatal–prefrontal networks.

Date 2010
URL https://www.sciencedirect.com/science/article/pii/S0911604409000864
Volume 23
Pages 162-171
Publication Journal of Neurolinguistics
DOI https://doi.org/10.1016/j.jneuroling.2009.12.003
Issue 2
ISSN 0911-6044
Date Added 6.7.2025, 19:12:34
Modified 5.9.2025, 14:46:06

Notes:

Included

Sample characteristics

Size: 28 PC, 32 HC

PD-type: Idiopathic PD

PD-duration: NA

Medication: LDE mentioned

Hoehn-Yahr: The majority (>90%) were either level two or three on the Hoehn and Yahr scale (M = 2.74)

UPDRS-3: NA

Gender (male):

Age: M = 56.3

Other neurological disease (tumor, stroke, etc.): None

Other major psychopathology: none

Origin country (or ethnicity): USA

method (Review, meta-analysis or observational and/or self-reported):

Experiment 2 (same sample)

instruments used in order to quantify the variables

Social cognition aspect: Social decision making

Name of the task: Sentence verification task

Type of stimulus [face/voice etc., Ekman faces/other etc.]: Twelve scenarios (six opinion and six disclosure), similar to those used in Experiment 1 (but with different content).

Task condition: There were three versions of each scenario. In one version the requested information was described as clearly negative (negative context), in another version the requested information was described as clearly positive (positive context), and in another version no information was provided (neutral context). In addition, there were eight filler scenarios. The fillers included scenarios in which the reply was not a relevance violation, but instead directly conveyed a negative or positive opinion or disclosure

Operationalization: Participants were asked to read a scenario and corresponding remarks, and to then write down how they would interpret the reply. Participants were asked to place their interpretations in one of three categories to indicate their degree of confidence in the reply (speaker most certainly meant = 1; speaker probably meant = 2; speaker might have meant = 3)

Participants' responses were coded as either literal or indirect, and if the latter, whether the interpretation was negative, positive or neutral.

The percentage of negative interpretation and degree of confidence were the primary dependent measures.

Main findings related to the review's scope

There was a significant effect for valence

Follow-up tests indicated that there was no difference in the number of negative interpretations for the no context and negative context scenarios ($p > .4$). The positive scenario resulted in significantly fewer negative interpretations than either the negative or no context versions (both ps < .001). Most importantly, this pattern of data was very similar for both PD and control participants (see Fig. 2), and the Participant Classification Valence interaction was not significant

For confidence, there was a significant valence effect, and participants were more confident of their interpretations in the negative and no context versions than in the positive scenarios (both ps < .01). Again, this pattern of data was very similar for both PD and control participants. However, there was a significant difference between the PD and control participants in terms of overall confidence, PD participants ($M = 1.53$) were more confident of their interpretations than the control participants ($M = 1.72$)

Tags: social decision-making, behavioral

Social cognition training improves recognition of distinct facial emotions and decreases misattribution errors in healthy individuals.

Item Type Journal Article

Author Samantha Evy Schoeneman Patel
Author Kristen M. Haut
Author Erin Guty
Author David Dodell-Feder
Author Abhishek Saxena
Author Mor Nahum
Author Christine I. Hooker

Abstract Facial emotion recognition is a key component of social cognition. Impaired facial emotion recognition is tied to poor psychological wellbeing and deficient social functioning. While previous research has demonstrated the potential for social cognition training to improve overall facial emotion recognition, questions remain regarding what aspects of emotion recognition improve. We report results from a randomized controlled trial that evaluates whether computerized social cognition training can improve recognition of distinct facial emotions in healthy participants. This investigation was designed to better understand the therapeutic potential of social cognition training for individuals with neuropsychiatric disorders. Fifty-five healthy adult participants were randomly assigned to an internet-based intervention during which they either completed social cognition training (SCT) or played control computer games (CON) for 10.5 h over 2-3 weeks. Facial emotion recognition was measured with the Penn ER-40, which was conducted before and after training. The following variables were collected and analyzed: facial emotion recognition accuracy for each emotion (i.e., anger, fear, happy, neutral (no emotional expression), and sad), reaction times for each emotion, and response error types (i.e., frequency of an emotion being chosen incorrectly, frequency of an emotion being missed, and frequency of an emotion being confused for another particular emotion). ANOVAs and t-tests were used to elucidate intervention effects both within and between groups. Results showed that the SCT group improved their accuracy for angry and neutral faces. They also improved their reaction times for neutral, fearful, and sad

faces. Compared to the CON group, the SCT group had significantly faster reaction times to neutral faces after training. Lastly, the SCT group decreased their tendency to confuse angry faces for no emotional expression and to confuse no emotional expression for sad faces. In contrast, the CON group did not significantly improve their accuracy or reaction times on any emotional expression, and they did not improve their response error types. We conclude that social cognition training can improve recognition of distinct emotions in healthy participants and decrease response error patterns, suggesting it has the potential to improve impaired emotion recognition and social functioning in individuals with facial emotion recognition deficits.

Date 2022
Language eng
License Copyright © 2022 Schoeneman Patel, Haut, Guty, Dodell-Feder, Saxena, Nahum and Hooker.
Extra Place: Switzerland
Volume 13
Pages 1026418
Publication Frontiers in psychiatry
DOI 10.3389/fpsyt.2022.1026418
Journal Abbr Front Psychiatry
ISSN 1664-0640
PMID 36424990
PMCID PMC9680726
Date Added 6.7.2025, 19:09:37
Modified 5.9.2025, 14:55:59

Notes:

Not Included: Training study with healthy individuals

Tags: EXCLUDED

Social cognitive deficits and their neural correlates in progressive supranuclear palsy

Item Type Journal Article
Author Boyd C. P. Ghosh
Author Andrew J. Calder
Author Polly V. Peers
Author Andrew D. Lawrence
Author Julio Acosta-Cabronero
Author Joao M. Pereira
Author John R. Hodges
Author James B. Rowe
Abstract Although progressive supranuclear palsy is defined by its akinetic rigidity, vertical supranuclear gaze palsy and falls, cognitive impairments are an important determinant of patients' and carers' quality of life. Here, we investigate whether there

is a broad deficit of modality-independent social cognition in progressive supranuclear palsy and explore the neural correlates for these. We recruited 23 patients with progressive supranuclear palsy (using clinical diagnostic criteria, nine with subsequent pathological confirmation) and 22 age- and education-matched controls. Participants performed an auditory (voice) emotion recognition test, and a visual and auditory theory of mind test. Twenty-two patients and 20 controls underwent structural magnetic resonance imaging to analyse neural correlates of social cognition deficits using voxel-based morphometry. Patients were impaired on the voice emotion recognition and theory of mind tests but not auditory and visual control conditions. Grey matter atrophy in patients correlated with both voice emotion recognition and theory of mind deficits in the right inferior frontal gyrus, a region associated with prosodic auditory emotion recognition. Theory of mind deficits also correlated with atrophy of the anterior rostral medial frontal cortex, a region associated with theory of mind in health. We conclude that patients with progressive supranuclear palsy have a multimodal deficit in social cognition. This deficit is due, in part, to progressive atrophy in a network of frontal cortical regions linked to the integration of socially relevant stimuli and interpretation of their social meaning. This impairment of social cognition is important to consider for those managing and caring for patients with progressive supranuclear palsy.

Date 2012-07
Language English
Extra Place: GREAT CLARENDON ST, OXFORD OX2 6DP, ENGLAND Type: Article
Volume 135
Publisher OXFORD UNIV PRESS
Pages 2089-2102
Publication BRAIN
DOI 10.1093/brain/aws128
Issue 7
ISSN 0006-8950
Date Added 14.7.2025, 14:50:39
Modified 5.9.2025, 14:36:36

Notes:

Not Included: not on PD
Tags: EXCLUDED

[Social cognitive function in neurodegenerative diseases].

Item Type Journal Article
Author A. Sh Chimagomedova
Author E. A. Lyashenko
Author O. V. Babkina
Author O. V. Iakovleva
Author E. E. Vasenina
Author O. S. Levin

Abstract The ability to perceive, analyze people's mental states, intentions, thoughts and feelings is an important cognitive function for normal social behavior and interaction. Over the past decade, more attention has been paid to studying how behavioral disorders in patients with neurodegenerative diseases may be explained by theory of mind deficit and whether it can be useful for differential diagnosis. The authors consider the issues of neuroanatomy, neurophysiology of a special kind of cognitive functions provided normal social interaction and interpersonal relationship, problems of its determining in neurodegenerative diseases.

Date 2017
Language rus
Extra Place: Russia (Federation)
Volume 117
Pages 168-173
Publication Zhurnal nevrologii i psikhiatrii imeni S.S. Korsakova
DOI 10.17116/jnevro2017117111168-173
Issue 11
Journal Abbr Zh Nevrol Psikhiatr Im S S Korsakova
ISSN 1997-7298
PMID 29265104
Date Added 6.7.2025, 19:09:41
Modified 5.9.2025, 14:30:46

Notes:

Not Included: not in English
Tags: EXCLUDED

Social cognitive impairment in early Parkinson's disease: A novel "mild impairment"?

Item Type Journal Article
Author Virginie Czernecki
Author Eve Benchetrit
Author Marion Houot
Author Fanny Pineau
Author Graziella Mangone
Author Jean-Christophe Corvol
Author Marie Vidailhet
Author Richard Levy
Abstract INTRODUCTION: Social cognition (SC) deficit has recently been described in the early stages of Parkinson's disease (PD), but findings remain unclear. Our objective was to determine the frequency of SC impairment in newly-diagnosed PD patients and whether it is independent of Mild Cognitive Impairment (MCI). METHODS: We enrolled 109 patients with idiopathic PD diagnosed within the previous four years (ICEBERG cohort) and 39 healthy participants. SC was evaluated using the Mini-Social Cognition and Emotional Assessment (Mini-SEA) that allows a multi-domain

assessment of SC. Relationships between SC and clinical characteristics, global cognitive efficiency, mood, anxiety, apathy and impulse control disorders, were also evaluated. RESULTS: 30% of patients had significant socio-emotional impairment. Moreover, SC deficit in isolation was 3.5 times more frequent than MCI in isolation (20.2% vs 5.5% respectively). Both emotion identification and Theory of Mind were impaired compared to healthy participants. No effect of age, level of education, disease severity, dopamine replacement therapy, or global cognitive efficiency were found. Only scores on the Frontal Assessment Battery were correlated with SC abilities. CONCLUSION: SC impairment is frequent in early PD and should be given more consideration. It often occurs in the absence of any other cognitive disorder and may represent the most common neuropsychological deficit in early-stage PD. In line with the definition of PD-MCI criteria, we consider the addition of a sixth MCI sub-type termed "Mild Social Cognition Impairment (MSCI)". Further studies are required to validate the addition of this new MCI domain.

Date 2021 Apr
Language eng
License Copyright © 2021 Elsevier Ltd. All rights reserved.
Extra Place: England
Volume 85
Pages 117-121
Publication Parkinsonism & related disorders
DOI 10.1016/j.parkreldis.2021.02.023
Journal Abbr Parkinsonism Relat Disord
ISSN 1873-5126 1353-8020
PMID 33812772
Date Added 6.7.2025, 19:09:34
Modified 5.9.2025, 14:32:10

Notes:

Included

sample characteristics

size: 109 PD and 39 HC

Parkinson's Disease type and duration: idiopathic PD, Mduration= 17.32 SD= 11.84

Medication: on medication

Hoehn-Yahr: M= 2.06 SD= 0.25

UPDRS-3: M= 30.23 SD= 7.93 (off medication)

Gender (male): 73 males (67%)

averaged ages (SD, range): M= 61.90 SD= 9.31

other neurological disease (tumor, stroke, etc.): None

other major psychopathology: None

origin country (or ethnicity): France

method observational

instruments used in order to quantify the variables

Social cognition aspect: emotion recognition and ToM

Name of the task: The Mini-social cognition and Emotional Assessment (Mini-SEA)

type of stimulus [face/voice etc., Ekman faces/other etc.]: a multidomain assessment of [redacted]. It includes two subtests: one to measure emotional identification, through a facial emotion recognition test based on the Ekman pictures sample; and the other for [redacted] abilities using a shortened version of the "Faux-Pas" Recognition test.

Emotion identification test

Participants had to identify which emotion was expressed on a picture of a face out of six primary emotions.

task condition: (fear, sadness, disgust, surprise, anger, happiness) and a neutral expression

operationalization: total positive (happiness) emotion score (out of 5) and a negative (fear, sadness, disgust, anger) emotion score (out of 20).

ToM test

This subtest included five stories described as a "faux-pas" (i.e., an embarrassing or tactless act or remark made unintentionally by someone in a social situation), and five control stories. To reduce memory load, participants were read the stories with a written version placed in front of them. After each story, participants were asked if something inappropriate was said ("faux pas detection"). If they answered positively, further clarifying questions were asked to test their understanding of the "faux pas" situation: four questions about cognitive understanding (attribution, intention, inference,

understanding) and one about the feelings of the protagonist. Finally, patients were asked two control questions to check their comprehension

Operationalization: The total score was the sum of correct responses for “faux pas” stories (out of 30). Two complementary sub-scores were calculated: a “cognitive ToM” score (out of 25) that results from the sum of the first five questions and an “affective ToM” score (out of 5) from the last question.

Main findings related to the review's scope

The PD patients obtained significantly lower scores than the HC both for the Mini-SEA total score, and for the two sub-scores, the emotional identification task (p = 0.022) and the “faux pas” (ToM) task

Only negative emotions, mainly fear and sadness, were not well identified by the patients in the emotional identification task.

Tags: Emotion recognition, ToM, behavioral

Social cognitive impairment in Parkinson's disease

Item Type Journal Article

Author Mitsuru Kawamura
Author Shinichi Koyama

Abstract Parkinson's disease (PD) is thought to be primarily a disorder of the motor system due to dysfunction of the nigrostriatal dopaminergic system. However, recent studies have revealed that social cognition tasks, such as mind-reading, facial expression recognition, and decision-making, are also impaired in PD. The studies also demonstrated that these impairments can occur due to dysfunctions of the mesocorticolimbic dopaminergic system, particularly in the amygdala. The social cognitive impairments may develop in the early stage of PD.

Date 2007-08
Language English
Extra Place: TIERGARTENSTRASSE 17, D-69121 HEIDELBERG, GERMANY Type: Article; Proceedings Paper
Volume 254
Publisher SPRINGER HEIDELBERG
Pages 49-53
Publication JOURNAL OF NEUROLOGY
DOI 10.1007/s00415-007-4008-8
Issue 4
ISSN 0340-5354
Date Added 14.7.2025, 14:50:43
Modified 5.9.2025, 14:41:26

Notes:

Not Included not an empirical study

Tags: EXCLUDED

Social decision-making in Parkinson's disease.

Item Type Journal Article
Author Jonathan A. Caballero
Author Noémie Auclair Ouellet
Author Natalie A. Phillips
Author Marc D. Pell
Abstract INTRODUCTION: Parkinson's Disease (PD) commonly affects cognition and communicative functions, including the ability to perceive socially meaningful cues from nonverbal behavior and spoken language (e.g., a speaker's tone of voice). However, we know little about how people with PD use social information to make decisions in daily interactions (e.g., decisions to trust another person) and whether this ability rests on intact cognitive functions and executive/decision-making abilities in nonsocial domains. METHOD: Non-demented adults with and without PD were presented utterances that conveyed differences in speaker confidence or politeness based on the way that speakers formulated their statement and their tone of voice. Participants had to use these speech-related cues to make trust-related decisions about interaction partners while playing the Trust Game. Explicit measures of social perception, nonsocial decision-making, and related cognitive abilities were collected. RESULTS: Individuals with PD displayed significant differences from control participants in social decision-making; for example, they showed greater trust in game partners whose voice sounded confident and who explicitly stated that they would cooperate with the participant. The PD patients displayed relative intact social perception (speaker confidence or politeness ratings) and were unimpaired on a nonsocial decision-making task (the Dice game). No obvious relationship emerged between measures of social perception, social decision-making, or cognitive functioning in the PD sample. CONCLUSIONS: Results provide evidence of alterations in decision-making restricted to social contexts in PD individuals with relatively preserved cognition with minimal changes in social perception. Researchers and practitioners interested in how PD affects social perception and cognition should include assessments that emulate social interactions, as non-interactive tasks may fail to detect the full impact of the disease on those affected.
Date 2022 May
Language eng
Extra Place: England
Volume 44
Pages 302-315
Publication Journal of clinical and experimental neuropsychology
DOI 10.1080/13803395.2022.2112554
Issue 4
Journal Abbr J Clin Exp Neuropsychol
ISSN 1744-411X 1380-3395
PMID 35997248
Date Added 6.7.2025, 19:09:34
Modified 5.9.2025, 14:30:01

Notes:

Included

sample characteristics

size: 15 PD and 14 HC (Matched in age and education)

Parkinson's Disease type and duration: NA, Mduration=NA

Medication: on medication

Hoehn-Yahr: M=NA 1-3

UPDRS-3: M=NA

Gender (male): 8 males (53%)

averaged ages (SD, range): M = 70.33 years old; SD = 5.39

other neurological disease (tumor, stroke, etc.): None

other major psychopathology: NA

origin country (or ethnicity): Canada

method observational

instruments used in order to quantify the variables

Social cognition aspect: ToM

Name of the task: The Faux-pas test

type of stimulus [face/voice etc., Ekman faces/other etc.]: 20 short stories describing a social context ending with a comment, which would be considered a "faux-pas" (i.e., socially inappropriate) in half of the scenarios. Five ratio scores ranging from zero to one were obtained from the test: "Detection score" (FP-Det), which measures the correct identification of inappropriate comments; "Understanding inappropriateness" (FP-Ina) which measures the capacity to understand why the comment is inappropriate; "Intention" (FP-Int), which measures the capacity of attributing intentions to the characters; "Belief" (FP-Bel), which measures the capacity to understand the characters' knowledge within the story; and "Empathy" (FP-Emp) which measures the capacity to infer the characters' affective experience.

operationalization: sum correct score

Social cognition aspect: social decision making

Name of the task: the Trust Game

type of stimulus [face/voice etc., Ekman faces/other etc.]: 64 audio recordings were used to represent the "trustee" in different trials. Utterances varied in terms of explicit (lexical-semantic content) and implicit (tone of voice) cues revealing the speakers' level of politeness or confidence (32 recordings for each). For the confidence recordings, the statement "I will be a fair player" was created to be appropriate and natural for the social context depicted in the Trust Game. This base statement was then manipulated to include lexical cues conveying different degrees of confidence: "For sure, I will be a fair player," "I'm pretty sure, I will be a fair player" and "Maybe, I will be a fair player." Each

statement was said in a confident and doubtful tone of voice by two male and two female speakers (4 statements x 4 speakers x 2 tones of voice (confident, doubtful) = 32 confidence recordings total). For the politeness recordings, the contextually-relevant statement "Transfer me some money" was constructed and then lexically modified to vary the perceived (im)politeness of the statement, as follows: "Could you transfer me some money?," "Transfer me some money please," "Transfer me some money fool." Each statement was said in a polite and rude tone of voice by the same four speakers (4 statements x 4 speakers x 2 tones of voice (polite, rude) = 32 politeness recordings total).

operationalization: After listening to the recording, participants decided how many tokens they would give to the person they just heard (from 0 to 10) by clicking an on-screen control, the response was recorded, and the next trial began. After a short break following the Trust Game, participants completed two short perceptual tasks in which they listened again to all 64 recordings presented in the Trust Game. For each trial, participants evaluated the speaker in terms of how confident or polite they sounded (according to the condition) on a 5-point Likert scale (from 1-Not at all to 5-Very much). The order of block presentation (confidence, politeness) and stimuli within each block were randomized. To highlight the lack of social interaction in this task, participants were told that the speakers would not be informed about their responses in this task.

Main findings related to the review's scope

Explicit confidence ratings: Partners who spoke in a confident vs. doubtful voice always increased perceptual ratings of confidence. The differential effects of tone of voice on confidence ratings (confident > doubtful) were significantly greater in the PD than the HC.

Explicit politeness ratings: PD participants assigned higher politeness ratings than HC participants when partners said *Please* and *Could you* but not when partners produced the bare request *Transfer* or said *Fool*

Implicit effects of confidence distinctions on interpersonal trust: PD participants gave significantly more tokens/displayed greater trust than HC participants in partners when stimuli encoded confidence distinctions. this tendency was restricted to three statements: *For sure* and *I will*. No group differences were observed when the speaker said *Maybe*

Implicit effects of politeness distinctions on interpersonal trust: All participants gave more tokens to partners who spoke in a polite vs. rude tone of voice, but this effect was larger for PD than HC. PD participants gave polite (vs. rude) sounding partners more tokens when they also said *Please, Could you* or *Transfer.* When speakers made impolite statements (*Fool*), PD participants offered *less* tokens to polite vs. rude sounding partners

Tags: social decision-making, behavioral

Social inappropriateness in neurodegenerative disorders.

**Item Type**	Journal Article
**Author**	Philippe Desmarais
**Author**	Krista L. Lanctôt
**Author**	Mario Masellis
**Author**	Sandra E. Black
**Author**	Nathan Herrmann

Abstract BACKGROUND: New onset of mood and behavioral changes in middle-aged patients are frequently the first manifestations of an unrecognized neurocognitive disorder. Impairment of social cognition, the cognitive ability to process social information coming from others, such as emotions, to attribute mental states to others, and to respond appropriately to them, is often at the origin of behavioral manifestations in neurodegenerative disorders. METHODS: This paper reviews the current literature on social cognition impairment in neurocognitive disorders, particularly in prodromal stages of behavioral-variant frontotemporal dementia (bvFTD), Alzheimer's disease (AD), idiopathic Parkinson's disease (IPD), and Lewy body dementia (LBD). The concepts of social cognition will be reviewed, including its impairment and neural basis, its clinical assessment, and the different therapeutic interventions available clinically. RESULTS: Socially inappropriate behaviors, such as loss of empathy, inappropriateness of affect, and disinhibition are frequently reported in prodromal bvFTD and in prodromal AD. Lack of self-control, reduced perception of social cues, such as recognition of facial emotions and sarcastic speech, and impaired Theory of Mind all contribute to the neuropsychiatric symptoms and are secondary to neurodegeneration in specific brain regions. In contrasts to bvFTD and AD, deficits in social cognition in IPD occur later in the course of the disease and are often multifactorial in origin. CONCLUSIONS: Through various manifestations, social inappropriateness is frequently the first clinical sign of a neurodegenerative process, especially in AD and bvFTD, years before noticeable impairment on classical neuropsychological assessment and brain atrophy on imaging.

Date 2018 Feb
Language eng
Extra Place: United States
Volume 30
Pages 197-207
Publication International psychogeriatrics
DOI 10.1017/S1041610217001260
Issue 2
Journal Abbr Int Psychogeriatr
ISSN 1741-203X 1041-6102
PMID 28689508
Date Added 6.7.2025, 19:09:41
Modified 5.9.2025, 14:33:16

Notes:

Included – narrative/systematic review

sample characteristics

We performed a comprehensive review of the literature using electronic databases (Ovid MEDLINE, Embase, PsycINFO, and Cochrane Central Register of Controlled Trials) for papers published in English or French using keywords and synonyms associated with the epidemiology, pathogenesis, diagnosis, and treatment for “social inappropriateness,” “theory of mind (ToM),” “empathy,” and “neurodegenerative disorders” such as AD, bvFTD, idiopathic Parkinson's disease (IPD), and LBD. We manually searched the reference lists of identified reports for additional papers to supplement our electronic search. We conducted our searches up to November 2016, without restrictions for time. **We largely selected publications in the past 10 years, but did not exclude important older publications. Selection criteria also included a judgment on the novelty of studies and their relevance for the well-informed general physician.**

Main findings related to the review's scope

Studies have reported that IPD patients have difficulties with recognition of faces, as well as recognition of emotions from visual and auditory cues (Ariatti ***et al***., 2008; Bodden *et al.*, 2010). Impairment in ToM has been reported in IPD patients on the False Belief, Faux-Pas, and RMET tasks when compared to healthy adults (Saltzman *et al*., 2000). Striatal dopamine depletion in the dorsolateral and orbital frontostriatal loops has been implicated in their impaired ToM performances. However, evidence must be interpreted carefully as those studies included advanced IPD patients where executive dysfunction and psychiatric comorbidities are prevalent and could explain impairment on assessments. In contrast to AD and bvFTD, social cognitive deficits in IPD appear later in the course of the disease, as suggested from studies demonstrating early IPD patients perform comparably to healthy controls (Péron *et al.*, 2009; Bodden *et al*., 2010). Unlike AD and bvFTD, it is not known if these deficits translate to significant interpersonal difficulties. Although IPD patients are known to be at an increased risk of impulsive control disorders (ICDs), such as compulsive gambling, buying, sexual behaviors, and eating, these behavioral manifestations are thought to be primarily related to the use of PD medications, especially dopamine agonists (Weintraub *et al.*, 2015).

Tags: ToM, behavioral

Social perception in adults with Parkinson's disease.

Item Type Journal Article
Author Marc D. Pell
Author Laura Monetta
Author Kathrin Rothermich
Author Sonja A. Kotz
Author Henry S. Cheang
Author Skye McDonald
Abstract OBJECTIVE: Our study assessed how nondemented patients with Parkinson's disease (PD) interpret the affective and mental states of others from spoken language (adopt a "theory of mind") in ecologically valid social contexts. A secondary goal was to examine the relationship between emotion processing, mentalizing, and executive

functions in PD during interpersonal communication. METHOD: Fifteen adults with PD and 16 healthy adults completed The Awareness of Social Inference Test, a standardized tool comprised of videotaped vignettes of everyday social interactions (McDonald, Flanagan, Rollins, & Kinch, 2003). Individual subtests assessed participants' ability to recognize basic emotions and to infer speaker intentions (sincerity, lies, sarcasm) from verbal and nonverbal cues, and to judge speaker knowledge, beliefs, and feelings. A comprehensive neuropsychological evaluation was also conducted. RESULTS: Patients with mild-moderate PD were impaired in the ability to infer "enriched" social intentions, such as sarcasm or lies, from nonliteral remarks; in contrast, adults with and without PD showed a similar capacity to recognize emotions and social intentions meant to be literal. In the PD group, difficulties using theory of mind to draw complex social inferences were significantly correlated with limitations in working memory and executive functioning. CONCLUSIONS: In early PD, functional compromise of the frontal-striatal-dorsal system yields impairments in social perception and understanding nonliteral speaker intentions that draw upon cognitive theory of mind. Deficits in social perception in PD are exacerbated by a decline in executive resources, which could hamper the strategic deployment of attention to multiple information sources necessary to infer social intentions.

Date 2014 Nov
Language eng
License PsycINFO Database Record (c) 2014 APA, all rights reserved.
Extra Place: United States
Volume 28
Pages 905-916
Publication Neuropsychology
DOI 10.1037/neu0000090
Issue 6
Journal Abbr Neuropsychology
ISSN 1931-1559 0894-4105
PMID 24885448
Date Added 6.7.2025, 19:09:42
Modified 5.9.2025, 14:49:42

Notes:

Included

Sample characteristics

Size: 15 PD, 16 HC (age, education machted)

PD-type: Idiopathic PD

PD-duration: M = 10.6 years (range 4 to 15 years)

Medication: ON state

Hoehn-Yahr: Range = 2-3.5

UPDRS-3: Range = 16-43; M = 29.9, Sd = 9.1

Gender (male): 8 (53%)

Age: M=70.1 SD=10.8

Other neurological disease (tumor, stroke, etc.): None

Other major psychopathology: None

Origin country (or ethnicity): Canada

method behavioural

instruments used in order to quantify the variables

Social cognition aspect: Empathy AND Emotion recognition

Name of the task: The Awareness of Social Inference Test (TASIT)

this tool was designed to assess the ability to perceive social cues presented in a realistic setting

Type of stimulus [face/voice etc., Ekman faces/other etc.]: short video clips performed by trained professional (Australian) actors in English

Task condition:

1. Emotion evaluation: This test examines the ability to categorize six basic emotions (happy, sad, fear, disgust, surprise, and anger) from verbal and nonverbal cues. Participants viewed 28 video vignettes, each 15 to 60 s in duration, of professional actors portraying one of the six emotions or "neutral" scenes that conveyed no emotion. 7-way forced choice
2. Test of social inference (minimal): This test evaluates the ability to perceive social inferences and to make judgments about the thoughts and feelings of speakers from their verbal and paralinguistic cues (i.e., facial expressions, prosody). This test consists of 15 short (20 to 60 s) video vignettes, which model a range of everyday conversational exchanges between two or more persons, performed by the same actors from Part 1. Participants must interpret the behavior of a target actor in the vignette. There are three types of exchanges in Part 2: sincere exchanges, simple sarcastic exchanges, and paradoxical sarcastic exchanges
3. Test of social inference (enriched): This test evaluates the ability to make social inferences inherent in telling lies or in expressing sarcasm. Similar to Part 2, this test consists of a series of 16 unique video vignettes (each lasting between 15 and 60 s) in which actors are engaged in routine conversations. However, in the "enriched" test, there are no depictions of literal conversations between characters; rather, participants must always uncover the intended, nonliteral meanings of the target actor. Half of the vignettes depict situations in which target characters are being sarcastic, and the remaining vignettes depict situations in which target characters are telling white (or "sympathetic") lies to spare a second character's feelings. In the sarcastic vignettes, all involved characters are aware of the target character's state of mind, whereas in the lie vignettes, the nontarget characters are not. Participants are provided enriched verbal or visual contextual cues to determine the communicative intention of the target actor

Operationalization: Correct answers

Main findings related to the review's scope

1. Emotion evaluation: There was no significant effect of Group or interaction of Group Emotion
2. Test of social inference (minimal): There was no evidence that the groups differed on this task, as no main or interactive effects reached. statistical significance (Group, F[1, 29] 1.72, p .20, p 2 0.056; Intention, F[2, 58] 1.45, p .24, p 2 0.048; Group Intention, F(2, 58) 0.46, p .62, p 2 0.015; all ns).
3. Test of social inference (enriched): The analysis revealed significant main effects for Intention, F(1, 29) 10.89, p .01, r .52, p 2 0.273, and for Group, F(1, 22) 8.63, p .01, r .48, p 2 0.23, but no significant interaction of these factors, F(1, 29) 0.09, p 2 0.003, ns. The Group main effect was explained by the inferior capacity of PD patients to recognize nonliteral intentions when compared with the HC group overall

Tags: Emotion recognition, Empathy, behavioral

Social perceptual function in parkinson's disease: A meta-analysis.

Item Type Journal Article
Author Sarah P. Coundouris
Author Alexandra G. Adams
Author Sarah A. Grainger
Author Julie D. Henry

Abstract Social perceptual impairment is a common presenting feature of Parkinson's disease (PD) that has the potential to contribute considerably to disease burden. The current study reports a meta-analytic integration of 79 studies which shows that, relative to controls, PD is associated with a moderate emotion recognition deficit (g = -0.57, K = 73), and that this deficit is robust and almost identical across facial and prosodic modalities. However, the magnitude of this impairment does appear to vary as a

function of task and emotion type, with deficits generally greatest for identification tasks (g = -0.65, K = 54), and for negative relative to other basic emotions. With respect to clinical variables, dopaminergic medication, deep brain stimulation, and a predominant left side onset of motor symptoms are each associated with greater social perceptual difficulties. However, the magnitude of social perceptual impairment seen for the four atypical parkinsonian conditions is broadly comparable to that associated with PD. The theoretical and practical implications of these findings are discussed.

Date 2019 Sep
Language eng
License Copyright © 2019 Elsevier Ltd. All rights reserved.
Extra Place: United States
Volume 104
Pages 255-267
Publication Neuroscience and biobehavioral reviews
DOI 10.1016/j.neubiorev.2019.07.011
Journal Abbr Neurosci Biobehav Rev
ISSN 1873-7528 0149-7634
PMID 31336113
Date Added 6.7.2025, 19:09:38
Modified 5.9.2025, 14:31:41

Notes:

Included – meta-analysis

sample characteristics

The meta-analysis was conducted in line with PRISMA guidelines (Moher et al., 2009). A systematic literature search of four electronic databases (PsycINFO, MEDLINE, PubMed, Web of Science) was completed in December 2018. The search terms corresponded with those used by Adams et al.'s (2019; for [redacted] terms) and Coundouris et al.'s (2019; for PD terms) meta-analytic reviews. Specifically the key terms searched were, [redacted], social perception, emotion perception, emotion recognition, facial expression*, prosody, [redacted], mentalising, pragmatic impairment, non-literal language, sarcas*, lie*, joke*, empath*, perspective taking, Frontal Systems [redacted] Scale, Frontal Behavioral Inventory, Socioemotional Dysfunction Scale, Peer-Report Social Functioning Scale, Social Impairment Rating Scale, Iowa Scales of Personality Change, Frontal Lobe Personality Scale; in combination with, hypokinetic [redacted], Parkinson's disease, [redacted], Parkinson'

N = 79

To ensure that the studies had comparable outcomes, the following additional inclusion criteria to the social perception tasks were applied:

1)Measures were included if they assessed the ability to interpret perceptual cues such as recognising facial emotions, interpreting tone of voice, and identifying bodily expressions;

2)Tasks, such as those measuring lexical perception (e.g. Paulmann and Pell, 2010), musical perception (e.g. Lima et al., 2013; Saenz et al., 2013), or affective images/videos/sounds (i.e. from the International Affective Picture System and International Affective Digitised Sounds database; e.g. Yuvaraj et al., 2014, 2017) were considered ineligible, as they lack the core social component associated with viewing faces and communicative gestures or listening to voices;

3)For faces and prosody, only tasks requiring judgement of the cross-culturally identifiable six basic emotions (happiness, sadness, anger, fear, surprise, and disgust), and neutral expressions/tones were included. More complex social emotion judgements were ineligible (e.g. Laskowska, 2015; Martins et al., 2008);

4)For bodily gestures, tasks were included if they measured one's capacity to recognise at least one of the six basic emotions, or recognise socially communicative gestures.

5)To qualify as a pure social perception task, the measure could not have examined emotional experience over perception, involve imitation or production, or have introduced additional cognitive demands (e.g. Alonso-Recio et al., 2014a, 2014c); and

6)Studies had to report task accuracy; studies that only reported intensity or valence were ineligible (e.g. Lin et al., 2016; Péron et al., 2015). However, studies that converted intensity data into decoding accuracy scores (i.e. correct if the highest intensity rating corresponded to the target emotion) could contribute.

Seventy-nine studies published between 1984 and 2018 met the inclusion criteria, and in total, data from 1987 PwPD, 94 people with one of the PPS, and 2067 controls contributed to the analyses. The three groups of participants were broadly equivalent in terms of age

(M_{PD} = 63.21, SD_{PD} = 6.24, M_{PPS} = 67.26, SD_{PPS} = 0.70, and $M_{Control}$ = 62.88, $SD_{Control}$ = 5.04). For PwPD, the mean disease duration was 7.47 years (*SD* = 3.05), and for the PPS, 4.28 (*SD* = 0.45). The mean PD disease severity was 2.11 (*SD* = 1.08), using Hoehn and Yahr's (1967) scale, and with the UPDRS was 22.00 (*SD* = 8.30). Finally, PD participants' average MMSE score was 27.63 (*SD* = 4.33).

Main findings related to the review's scope

Highlights

•Parkinson's disease is associated with significant social perceptual impairment.

•The magnitude of this impairment is broadly equivalent across facial and prosodic modalities.

•Impairment is evident for all six basic emotions, but is generally greater for negatively valanced emotions.

•Both medication and deep brain stimulation are associated with poorer social perceptual function.

•People with predominant left side onset of motor symptoms have greater social perceptual difficulties.

social perceptual difficulties as a common feature of PD

Task features. The first key finding to emerge was that PwPD are moderately and equivalently impaired in their ability to recognise emotion from facial and prosodic stimuli.

Specific emotions. Another key finding to emerge in the current review was the identification of significant PD-related difficulties for all six of the basic emotions, as well as for neutral expressions.

Clinical features. The final goal of the current meta-analysis was to clarify whether specific clinical features are related to social perceptual difficulties. Focusing firstly on medication, the results revealed that medicated, dopamine-replete PwPD presented with significant social perceptual deficits that were moderate in magnitude, and substantially greater than those in a non-medicated, hypodopaminergic state.

The current meta-analysis also identified a negative effect of DBS on PwPD's social perceptual ability.

The results of the current meta-analysis also provide novel evidence that motor symptom onset lateralisation is related to the magnitude of the social perceptual difficulties associated with PD.
Tags: emotion recognition, behavioral

Social problem solving, social cognition, and mild cognitive impairment in Parkinson's disease.

**Item Type**	Journal Article
**Author**	Rachel J. Anderson
**Author**	Anna C. Simpson
**Author**	Shelley Channon

Author Michael Samuel
Author Richard G. Brown
Abstract Cognitive impairment is a recognized feature of Parkinson's disease (PD), which, even if mild, can impact some aspects of a patient's ability to deal with everyday life. The current study examined the ability to solve social problems in three groups of participants: PD patients with mild cognitive impairment (PD-MCI); PD patients with no evidence of cognitive impairment (PD-N); and non-PD age-matched controls. All participants completed measures examining their ability to understand the actions and sarcastic remarks of others; provide a range of, and select, optimal solutions to social problems; and their self-perception of problem-solving abilities. Deficits emerged in the PD-MCI, but not the PD-N, group, suggesting that difficulties related to pathophysiological changes are associated with cognitive impairment and not PD per se. The findings are discussed with reference to the substrate of executive function and social cognition, and their implications for social interaction and everyday problem solving for people with PD.
Date 2013 Apr
Language eng
Extra Place: United States
Volume 127
Pages 184-192
Publication Behavioral neuroscience
DOI 10.1037/a0030250
Issue 2
Journal Abbr Behav Neurosci
ISSN 1939-0084 0735-7044
PMID 23067384
Date Added 6.7.2025, 19:09:33
Modified 5.9.2025, 14:26:15

Notes:

Included

sample characteristics

size: 36 PD and 16 HC age-matched

Parkinson's Disease type and duration: , Mduration= 7.3 SD=5.4

Medication: on medication

Hoehn-Yahr: M=2.81 SD=0.62 2-4

UPDRS-3: M= 25.3 SD=10.4

Gender (male): 27 males (55%)

averaged ages (SD, range): M= 65.9 SD=8.5

other neurological disease (tumor, stroke, etc.): none

other major psychopathology: NA

origin country (or ethnicity): United Kingdom

method observational

instruments used in order to quantify the variables

Social cognition aspect: Social problem solving

Name of the task: Social Problem Fluency.

type of stimulus [face/voice etc., Ekman faces/other etc.]: 10 different scenarios to assess their ability to generate a range of appropriate and effective solutions to awkward problem situations

operationalization: Participants were first asked to explain why the situation might be awkward for the character involved (scored 1 or 0) and to rate the awkwardness. Participants were then asked what the character could do in this situation and allowed 1 min to suggest as many solutions as they could. Responses were recorded and solutions subsequently rated using operationalized criteria as either socially sensitive and practical (SP; e.g., make an excuse that he needs to work), socially sensitive only (S; e.g., concentrate on his book), practical only (P; e.g., tell her to mind her own business), or neither socially sensitive nor practical (N). For each scenario, two broad, alternative satisfactory solutions were defined for each of the SP, S, and P response types. A particular type of solution was counted once only for each scenario to avoid counting simple variations in the solutions offered. Across the 10 scenarios tested, participants could score a maximum of 20 SP, 20 S, 20 P, and 10 N responses. The scores for each of these were converted to percentages. In addition, the total number of solutions offered across all scenarios was calculated, excluding only clear repetitions. After the questions, four alternative solutions are presented for each scenario and the participants are asked to choose the 'best' solution for the character in the situation. Only one of the solutions for each represented an SP response. To score the alternatives, the number of times where an SP solution was chosen as the 'best' were summed (maximum 10) and converted to a percentage.

Social cognition aspect: Social problem solving

Name of the task: Social Problem Resolution.

type of stimulus [face/voice etc., Ekman faces/other etc.]: This consisted of 10 different scenarios with the same type of structure as the Social Problem Fluency Task items, each presenting an awkward social situation.

operationalization: Participants were asked to generate one “best” solution. The scoring categories were the same as in the Social Problem Fluency Task: SP (e.g., discuss it again with the neighbors and negotiate), S (e.g., discuss it with other residents), P (e.g., just keep complaining to the neighbors), and N. The percentage of responses in each category was calculated. A score of 2 was given for a SP solution, 1 for an S or P solution, and 0 for an N solution. A total score (maximum 20, converted to a percentage) indicated the overall quality of the solutions offered.

Social cognition aspect: Social problem solving

Name of the task: the Social Problem-Solving Inventory–Revised (Short Form; SPSI-R:S)

type of stimulus [face/voice etc., Ekman faces/other etc.]: self-report 25-item scale assessing five dimensions of problem-solving styles and solution generation: two “adaptive” problem-solving dimensions (positive problem orientation and rational problem solving), and three “dysfunctional” dimensions (negative problem orientation, impulsivity/carelessness style, and avoidance style).

operationalization: Total scores are compared with normative data provided by D’Zurilla et al. (2002).

Social cognition aspect: Social cognition

Name of the task: The Mentalistic Interpretation Task

type of stimulus [face/voice etc., Ekman faces/other etc.]: 15 brief, written scenarios, which remained on view during the response to remove the need to retain the item material in memory. assessed the ability to interpret sarcastic remarks or human actions (both requiring mentalizing ability) and scenarios involving simple physical events that served as a control.

operationalization: Participants were asked

(a) to offer an interpretation of the scenario (scored 1 or 0 using operationalized criteria);

(b) to choose the most likely explanation from four possible alternatives provided — one correct, one incorrect or irrelevant, and two that were either general or irrelevant; and

(c) to answer a factual question to check their understanding of the scenario.

Responses were recorded and later scored, with 2 points given to a clear and accurate explanation of the scenario, 1 point for a partially correct response, and 0 for an incorrect or irrelevant response, giving a quality of interpretation score for each scenario type (maximum 10, converted to a percentage). For the choice question, only correct selections were scored (maximum 5 per scenario type converted to a percentage), with all other selections scored 0. Scores for sarcastic remarks and actions were collapsed to provide a single index of mentalizing ability.

-

Main findings related to the review's scope

Divide sample into PD-MCI P-N AND HC

PD-MCI Versus PD-N

The Social Problem Fluency Task: the PD-MCI group was significantly less able to identify the reasons for awkwardness in the scenarios but did not differ in the subjective judgment of degree of

awkwardness.

In generating possible solutions, the PD-MCI produced only a mean of 39.8% of the maximum possible number of SP responses across the scenarios compared with 55.8% in the PD-N group. The two groups did not differ with respect to the number of solutions in the other categories or in their ability to select the "best" solution when given a range of alternatives.

The overall mean quality of the best solution offered on the Social Problem Resolution Task was not significantly worse in the PD-MCI group. The PD-MCI group tended to offer more irrelevant or ineffective solutions but did not offer fewer SP, S, or P solutions.

No differences emerged between the two groups on any of the SPSI-R:S subscales.

PD-N Versus Controls

The only significant difference to emerge between the PD-N and control groups was on the Social Problem Resolution Task, with PD-N producing fewer ineffective or irrelevant solutions.

The PD-N group, when compared with controls, did not exhibit any deficits with respect to the measures of social cognition or social problem solving.

Tags: social decision-making, behavioral

Social Problem Solving: Theory and Assessment.

**Item Type**	Book Section
**Editor**	Edward C. Chang
**Editor**	Thomas J. D'Zurilla
**Editor**	Lawrence J. Sanna
**Author**	Thomas J. D'Zurilla
**Author**	Arthur M. Nezu
**Author**	Albert Maydeu-Olivares
**Date**	2004
**Language**	en
**Short Title**	Social Problem Solving
**Library Catalog**	DOI.org (Crossref)
**URL**	https://content.apa.org/books/10805-001
**Accessed**	19.1.2026, 7:35:52
**Place**	Washington
**Publisher**	American Psychological Association
**ISBN**	978-1-59147-147-9
**Pages**	11-27
**Book Title**	Social problem solving: Theory, research, and training.
**DOI**	10.1037/10805-001
**Date Added**	19.1.2026, 7:35:52
**Modified**	19.1.2026, 7:35:52

Social Symptoms of Parkinson's Disease

Item Type Journal Article
Author Margaret T. M. Prenger
Author Racheal Madray
Author Kathryne Van Hedger
Author Mimma Anello
Author Penny A. MacDonald
Abstract Parkinson's disease (PD) is typically well recognized by its characteristic motor symptoms (e.g., bradykinesia, rigidity, and tremor). The cognitive symptoms of PD are increasingly being acknowledged by clinicians and researchers alike. However, PD also involves a host of emotional and communicative changes which can cause major disruptions to social functioning. These incude problems producing emotional facial expressions (i.e., facial masking) and emotional speech (i.e., dysarthria), as well as difficulties recognizing the verbal and nonverbal emotional cues of others. These social symptoms of PD can result in severe negative social consequences, including stigma, dehumanization, and loneliness, which might affect quality of life to an even greater extent than more well-recognized motor or cognitive symptoms. It is, therefore, imperative that researchers and clinicans become aware of these potential social symptoms and their negative effects, in order to properly investigate and manage the socioemotional aspects of PD. This narrative review provides an examination of the current research surrounding some of the most common social symptoms of PD and their related social consequences and argues that proactively and adequately addressing these issues might improve disease outcomes.
Date 2020-12-31
Language English
Extra Place: 111 RIVER ST, HOBOKEN 07030-5774, NJ USA Type: Review
Volume 2020
Publisher WILEY
Publication PARKINSONS DISEASE
DOI 10.1155/2020/8846544
ISSN 2090-8083
Date Added 14.7.2025, 14:50:31
Modified 14.7.2025, 14:50:31

Notes:

Not Included: Not a systematic Review
Tags: EXCLUDED

Source activity during emotion processing and its relationship to cognitive impairment in Parkinson's disease.

Item Type Journal Article
Author Kartik K. Iyer

Author Tiffany R. Au
Author Anthony J. Angwin
Author David A. Copland
Author Nadeeka N. W. Dissanayaka
Abstract BACKGROUND: Neural mechanisms contributing to an underlying cognitive impairment in Parkinson's disease (PD) are poorly understood. An effective method to probe cognitive processing deficits in PD is the examination of brain activity during emotional processes, particularly in explicit language emotion recognition contexts. METHODS: The present study utilised cortical source imaging of event related potentials (ERP) from electroencephalography (EEG) to evaluate valence judgements on negative and neutral target words in an automatic affective priming paradigm. Fifty non-demented PD patients, unmedicated for depression or anxiety, completed affective priming tasks during EEG monitoring. Cognitive impairment was measured using the validated Parkinson's Disease-Cognitive Rating Scale (PD-CRS). RESULTS: Results reveal that compared to healthy age-matched controls, PD patients demonstrate a reduced N400 activation during affective priming tasks in bilateral regions of the middle frontal gyrus (MFG), inferior parietal lobule (IPL) and, notably, have a late wave ERP component (LPP) in left MFG, present between 600 and 800 ms, following family-wise error correction (p(FWE) < 0.05). LPP in PD patients were significantly associated with PD-CRS scores. LIMITATIONS: Although affective priming paradigms are an effective means for various domains of cognition, it is not a focused cognitive behavioural test for cognitive dysfunction. Our study is thus limited to a surrogate measure of cognitive dysfunction via examination of emotional word processing cues. CONCLUSIONS: These findings suggest that source imaging methods with ERP paradigms in PD are effective in identifying delayed cognitive processes in PD.
Date 2019 Jun 15
Language eng
License Copyright © 2019 Elsevier B.V. All rights reserved.
Extra Place: Netherlands
Volume 253
Pages 327-335
Publication Journal of affective disorders
DOI 10.1016/j.jad.2019.05.012
Journal Abbr J Affect Disord
ISSN 1573-2517 0165-0327
PMID 31078832
Date Added 6.7.2025, 19:09:38
Modified 5.9.2025, 14:40:25

Notes:

<u>**Not Included**</u>: Not on SC.

Tags: EXCLUDED

Specific impairments in the recognition of emotional facial expressions in Parkinson's disease.

**Item Type**	Journal Article
**Author**	Uraina S. Clark
**Author**	Sandy Neargarder
**Author**	Alice Cronin-Golomb
**Abstract**	Studies investigating the ability to recognize emotional facial expressions in non-demented individuals with Parkinson's disease (PD) have yielded equivocal findings. A possible reason for this variability may lie in the confounding of emotion recognition with cognitive task requirements, a confound arising from the lack of a control condition using non-emotional stimuli. The present study examined emotional facial expression recognition abilities in 20 non-demented patients with PD and 23 control participants relative to their performance on a non-emotional landscape categorization test with comparable task requirements. We found that PD participants were normal on the control task but exhibited selective impairments in the recognition of facial emotion, specifically for anger (driven by those with right hemisphere pathology) and surprise (driven by those with left hemisphere pathology), even when controlling for depression level. Male but not female PD participants further displayed specific deficits in the recognition of fearful expressions. We suggest that the neural substrates that may subserve these impairments include the ventral striatum, amygdala, and prefrontal cortices. Finally, we observed that in PD participants, deficiencies in facial emotion recognition correlated with higher levels of interpersonal distress, which calls attention to the significant psychosocial impact that facial emotion recognition impairments may have on individuals with PD.
**Date**	2008
**Language**	eng
**Extra**	Place: England
**Volume**	46
**Pages**	2300-2309
**Publication**	Neuropsychologia
**DOI**	10.1016/j.neuropsychologia.2008.03.014
**Issue**	9
**Journal Abbr**	Neuropsychologia
**ISSN**	0028-3932
**PMID**	18485422
**PMCID**	PMC2491661
**Date Added**	6.7.2025, 19:09:39
**Modified**	5.9.2025, 14:31:11

Notes:

Included

sample characteristics

size: 20 PD and 23 HC (match age or education)

Parkinson's Disease type and duration: idiopathic PD, Mduration= 7.3 SD=4.2

Medication: on medication

Hoehn-Yahr: Md=2 (2-3)

UPDRS-3: NA

Gender (male): 10 males (50%)

averaged ages (SD, range): M= 60.2 SD=8.2

other neurological disease (tumor, stroke, etc.): None

other major psychopathology: None

origin country (or ethnicity): NA

method observational

instruments used in order to quantify the variables

Social cognition aspect: Emotion recognition

Name of the task: prosodic portion of the New York Emotion Battery

type of stimulus [face/voice etc., Ekman faces/other etc.]: The content of each sentence was neutral, whereas the prosody of the sentence varied based on emotion. Each sentence was presented twice in succession, after which the participant identified the emotion expressed from a list of emotions

task condition: anger, disgust, fear, happy, sad, and positive surprise

operationalization: accuracy

Main findings related to the review's scope

No significant effects

Tags: emotion recognition, behavioral

Speech dysfunction, cognition, and Parkinson's disease.

**Item Type**	Book Section
**Author**	Andrea Rohl
**Author**	Stephanie Gutierrez
**Author**	Karim Johari

Author Jeremy Greenlee
Author Kris Tjaden
Author Angela Roberts
Abstract Communication difficulties are a ubiquitous symptom of Parkinson's disease and include changes to both motor speech and language systems. Communication challenges are a significant driver of lower quality of life. They are associated with decreased communication participation, social withdrawal, and increased risks for social isolation and stigmatization in persons with Parkinson's disease. Recent theoretical advances and experimental evidence underscore the intersection of cognition and motor processes in speech production and their impact on spoken language. This chapter overviews a growing evidence base demonstrating that cognitive impairments interact with motor changes in Parkinson's disease to negatively affect communication abilities in myriad ways, at all stages of the disease, both in the absence and presence of dementia. The chapter highlights common PD interventions (pharmacological, surgical, and non-pharmacological) and how cognitive influences on speech production outcomes are considered in each.
Date 2022
Language eng
License Copyright © 2022 Elsevier B.V. All rights reserved.
Extra Place: Netherlands PMID: 35248193 PMCID: PMC11321444
Volume 269
Pages 153-173
Book Title Progress in brain research
Date Added 6.7.2025, 19:09:33
Modified 5.9.2025, 14:53:37

Notes:

Not Included: Not a systematic Review

Tags: EXCLUDED

Startle reflex hyporeactivity in Parkinson's disease: An emotion-specific or arousal-modulated deficit?

Item Type Journal Article
Author K. M. Miller
Author M. S. Okun
Author M. Marsiske
Author E. B. Fennell
Author D. Bowers
Abstract We previously reported that patients with Parkinson's disease (PD) demonstrate reduced psychophysiologic reactivity to unpleasant pictures as indexed by diminished startle eyeblink magnitude [Bowers, D., Miller, K., Bosch, W., Gokcay, D., Pedraza, O., Springer, U., et al. (2006). Faces of emotion in Parkinsons disease: Micro-expressivity and bradykinesia during voluntary facial expressions. Journal of the International Neuropsychological Society, 12(6), 765-773; Bowers, D., Miller, K., Mikos, A., Kirsch-Darrow, L, Springer, U., Fernandez, H., et al. (2006). Startling

facts about emotion in Parkinson's disease: Blunted reactivity to aversive stimuli. Brain, 129(Pt 12), 3356-3365]. In the present study, we tested the hypothesis that this hyporeactivity was primarily driven by diminished reactivity to fear-eliciting stimuli as opposed to other types of aversive pictures. This hypothesis was based on previous evidence suggesting amygdalar abnormalities in PD patients, coupled with the known role of the amygdala in fear processing. To test this hypothesis, 24 patients with Parkinson's disease and 24 controls viewed standardized sets of emotional pictures that depicted fear, disgust (mutilations, contaminations), pleasant, and neutral contents. Startle eyeblinks were elicited while subjects viewed these emotional pictures. Results did not support the hypothesis of a specific emotional reactivity deficit to fear pictures. Instead, the PD patients showed reduced reactivity to mutilation pictures relative to other types of negative pictures in the context of normal subjective ratings. Further analyses revealed that controls displayed a pattern of increased startle eyeblink magnitude for “high arousal” versus “low arousal” negative pictures, regardless of picture category, whereas startle eyeblink magnitude in the PD group did not vary by arousal level. These results suggest that previous findings of decreased aversion-modulated startle is driven by reduced reactivity to highly arousing negative stimuli rather than to a specific category (i.e., fear or disgust) of emotion stimuli. Published by Elsevier Ltd.

Date 2009-07
Language English
Extra Place: THE BOULEVARD, LANGFORD LANE, KIDLINGTON, OXFORD OX5 1GB, ENGLAND Type: Article
Volume 47
Publisher PERGAMON-ELSEVIER SCIENCE LTD
Pages 1917-1927
Publication NEUROPSYCHOLOGIA
DOI 10.1016/j.neuropsychologia.2009.03.002
Issue 8-9
ISSN 0028-3932
Date Added 14.7.2025, 14:50:42
Modified 5.9.2025, 14:46:53

Notes:

not included: not on SC

Tags: EXCLUDED

Stimulation of subthalamic nucleus inhibits emotional activation of fusiform gyrus

Item Type Journal Article
Author Jacob Geday
Author Karen Ostergaard
Author Albert Gjedde

Abstract In patients with Parkinson's disease, deep brain stimulation of the subthalamic nucleus is known to impair their ability to correctly identify facial expressions of negative emotions. This difficulty exists only when the stimulator is active. The reason for the impairment is unknown. To test the hypothesis that the stimulation itself is responsible, we used positron emission tomography to compare functional activations of brain regions in nine patients with Parkinson's disease treated with surgically implanted electrodes into both subthalamic nuclei, and 22 healthy volunteers. Both groups viewed images with neutral or emotional content from Aarhus University's standard Empathy Picture System (www.geday.net/eps) with 360 images of people in pleasant, unpleasant or neutral real-life situations, presenting either the situations or close-ups of the facial expressions of the people involved. Both groups, the patients with stimulation OFF and the healthy volunteers, had raised regional blood flow rates (rCBF) in the right fusiform gyrus when they viewed emotionally expressive faces compared to neutral faces. With stimulation turned on, this response was significantly inhibited in the patients because of a raised rCBF at baseline during the neutral faces. Stimulation of the STN did not alter fusiform reaction to emotionally pregnant scenes; nor did healthy volunteers and patients react differently to these stimuli regardless of stimulation status. Also, STN stimulation raised the emotional activation of the anterior cingulate and lowered the activity of the putamen. The findings suggest that the stimulation of the subthalamic nucleus interferes with the integration of specific neocortical networks involved in the recognition of facial expressions. (c) 2006 Elsevier Inc. All rights reserved.

Date 2006-11-01
Language English
Extra Place: 525 B ST, STE 1900, SAN DIEGO, CA 92101-4495 USA Type: Article
Volume 33
Publisher ACADEMIC PRESS INC ELSEVIER SCIENCE
Pages 706-714
Publication NEUROIMAGE
DOI 10.1016/j.neuroimage.2006.06.056
Issue 2
ISSN 1053-8119
Date Added 14.7.2025, 14:50:43
Modified 5.9.2025, 14:36:26

Notes:

Not Included: only compare intervention vs non, with no HC

Tags: EXCLUDED

STN-DBS does not change emotion recognition in advanced Parkinson's disease.

Item Type Journal Article

Author Luisa Albuquerque
Author Miguel Coelho
Author Maurício Martins

Author Leonor Correia Guedes
Author Mário M. Rosa
Author Joaquim J. Ferreira
Author Maria Begoña Cattoni
Author Herculano Carvalho
Author A. Gonçalves Ferreira
Author Isabel Pavão Martins

Abstract Deep brain stimulation of the subthalamic nuclei (STN-DBS) for the treatment of levodopa-induced motor complications in advanced Parkinson's disease (APD) has been associated with neuropsychiatric disorders. It has been suggested that a postoperative decline in visual emotion recognition is responsible for those adverse events, although there is also evidence that emotional processing deficits can be present before surgery. The aim of the present study is to compare the ability to recognize emotions before and one year after surgery in APD. METHODS: Consecutively operated APD patients were tested pre-operatively and one year after STN-DBS by the Comprehensive Affect Testing System (CATS), which evaluates visual recognition of 7 basic emotions (happiness, sadness, anger, fear, surprise, disgust and neutral) on facial expressions and 4 emotions on prosody (happiness, sadness, anger and fear). RESULTS: In a sample of 30 patients 6 had depression or apathy at baseline that significantly increased to 14 post-surgery. There were no significant changes in the tests of identity discrimination, discrimination of emotional faces, naming of emotional faces, recognition of emotional prosody, and naming of emotional prosody after STN-DBS. The results of emotion tests could not predict the development of the neuropsychiatric symptoms. DISCUSSION: This study does not support the hypothesis of an acquired change in emotion recognition, either in faces or in prosody, after STN-DBS in APD patients. Neuropsychiatric symptoms appearing after STN-DBS should not be attributed to new deficits in emotional recognition.

Date 2014 Feb
Language eng
License Copyright © 2013 Elsevier Ltd. All rights reserved.
Extra Place: England
Volume 20
Pages 166-169
Publication Parkinsonism & related disorders
DOI 10.1016/j.parkreldis.2013.10.010
Issue 2
Journal Abbr Parkinsonism Relat Disord
ISSN 1873-5126 1353-8020
PMID 24182523
Date Added 6.7.2025, 19:09:36
Modified 5.9.2025, 14:25:20

Notes:

<u>**Not Included:**</u> not including comparison group (only pre-post DBS)

Tags: EXCLUDED

Structural and functional abnormalities in Parkinson's disease based on voxel-based morphometry and resting-state functional magnetic resonance imaging

**Item Type**	Journal Article
**Author**	Jin Hua Zheng
**Author**	Wen Hua Sun
**Author**	Jian Jun Ma
**Author**	Zhi Dong Wang
**Author**	Qing Qing Chang
**Author**	Lin Rui Dong
**Author**	Xiao Xue Shi
**Author**	Ming Jian Li
**Author**	Qi Gu
**Author**	Si Yuan Chen
**Abstract**	Objective To explore differences in gray matter volume (GMV) and white matter volume (WMV) between patients with Parkinson's disease (PD) and healthy controls, and to examine whether the structural abnormalities correlate with functional abnormalities. Methods T1-weighted magnetic resonance imaging and resting-state functional magnetic resonance imaging (fMRI) were performed on 180 patients with PD and 58 age- and sex-matched healthy controls. We used voxel-based morphometry (VBM) to compare GMV and WMV between groups, and resting-state fMRI to compare amplitudes of low-frequency fluctuations (ALFF) in the structurally abnormal brain regions. Results Structural neuroimaging showed smaller whole-brain GMV, but not WMV, in patients. Furthermore, VBM revealed smaller GMV in the right superior temporal gyrus (STG) and left frontotemporal space in patients, after correction for multiple comparisons. Patients also showed significantly higher ALFF in the right STG. GMV in the right STG and left frontotemporal space in patients correlated negatively with age and scores on Part III of the Movement Disorder Society Unified Parkinson's Disease Rating Scale, but not with PD duration. Conclusions Structural atrophy in the frontotemporal lobe may be a useful imaging biomarker in PD, such as for detecting disease progression. Furthermore, this structural atrophy appears to correlate with enhanced spontaneous brain activity. This study associates particular structural and functional abnormalities with PD neuropathology.
**Date**	2022
**URL**	https://www.sciencedirect.com/science/article/pii/S0304394022003962
**Volume**	788
**Pages**	136835
**Publication**	Neuroscience Letters
**DOI**	https://doi.org/10.1016/j.neulet.2022.136835
**ISSN**	0304-3940
**Date Added**	6.7.2025, 19:12:35
**Modified**	5.9.2025, 15:03:01

Notes:

Not Included: Not about SC
Tags: EXCLUDED

Structural correlates of facial emotion recognition deficits in Parkinson's disease patients.

**Item Type**	Journal Article
**Author**	H. C. Baggio
**Author**	B. Segura
**Author**	N. Ibarretxe-Bilbao
**Author**	F. Valldeoriola
**Author**	M. J. Marti
**Author**	Y. Compta
**Author**	E. Tolosa
**Author**	C. Junqué
**Abstract**	The ability to recognize facial emotion expressions, especially negative ones, is described to be impaired in Parkinson's disease (PD) patients. Previous neuroimaging work evaluating the neural substrate of facial emotion recognition (FER) in healthy and pathological subjects has mostly focused on functional changes. This study was designed to evaluate gray matter (GM) and white matter (WM) correlates of FER in a large sample of PD. Thirty-nine PD patients and 23 healthy controls (HC) were tested with the Ekman 60 test for FER and with magnetic resonance imaging. Effects of associated depressive symptoms were taken into account. In accordance with previous studies, PD patients performed significantly worse in recognizing sadness, anger and disgust. In PD patients, voxel-based morphometry analysis revealed areas of positive correlation between individual emotion recognition and GM volume: in the right orbitofrontal cortex, amygdala and postcentral gyrus and sadness identification; in the right occipital fusiform gyrus, ventral striatum and subgenual cortex and anger identification, and in the anterior cingulate cortex (ACC) and disgust identification. WM analysis through diffusion tensor imaging revealed significant positive correlations between fractional anisotropy levels in the frontal portion of the right inferior fronto-occipital fasciculus and the performance in the identification of sadness. These findings shed light on the structural neural bases of the deficits presented by PD patients in this skill.
**Date**	2012 Jul
**Language**	eng
**License**	Copyright © 2012 Elsevier Ltd. All rights reserved.
**Extra**	Place: England
**Volume**	50
**Pages**	2121-2128
**Publication**	Neuropsychologia
**DOI**	10.1016/j.neuropsychologia.2012.05.020
**Issue**	8
**Journal Abbr**	Neuropsychologia
**ISSN**	1873-3514 0028-3932
**PMID**	22640663

Date Added 6.7.2025, 19:09:36
Modified 5.9.2025, 14:27:23

Notes:

Included

sample characteristics

size: 39 PD and 23 HC age-matched

Parkinson's Disease type and duration: , Mduration= 5.6 SD=3.8

Medication: on medication

Hoehn-Yahr: M= 1.8 SD=0.5

UPDRS-3: M= 16.5 SD=8.3

Gender (male): 27 males (69%)

averaged ages (SD, range): M= 63.5 SD=11.4

other neurological disease (tumor, stroke, etc.): None

other major psychopathology: None

origin country (or ethnicity): Spain

method observational and imaging

instruments used in order to quantify the variables

Social cognition aspect: emotion recognition

Name of the task: the Ekman 60 test

type of stimulus [face/voice etc., Ekman faces/other etc.]: 60 pictures of faces from the Ekman and Friesen series of Pictures of Facial Affect

task condition: anger, fear, sadness, disgust, happiness and surprise

operationalization: accuracy

MRI: 3T scanner (TIM Trio, Siemens, Germany). High-resolution 3-dimensional T1-weighted images were acquired in the sagittal plane (TR 2300 ms, TE 2.98 ms, TI 900 ms; 256×256 matrix, 1 mm isotropic voxel). Sagittal diffusion tensor images were obtained using a single-shot EPI sequence (TR 5533 ms, TE 88 ms), with diffusion encoding in 30 directions at b=0 and 1000 s/mm2.

VBM: FSL tools. The resulting images were averaged to create a study-specific template, to which the native GM images were then non-linearly re-registered. The modulated segmented images were then smoothed with an isotropic Gaussian kernel with a sigma of 3 mm.

WM: TBSS (Tract-Based Spatial Statistics), part of FSL. FA images were created by fitting a tensor

model to the raw diffusion data using FDT, and then brain-extracted using BET. Mean FA image was created and thinned to create a mean FA skeleton which represents the centers of all tracts common to the group.

Main findings related to the review's scope

PD patients' total Ekman test scores and subscores in the identification of fear, sadness, anger and disgust were significantly lower than controls'.

Whole-brain voxelwise analysis: this analysis showed no significant correlations between Ekman test scores and GM volume.

ROI analysis, *sadness* scores: positive correlations were found between patients' *sadness* scores and GM volume in the right OFC – a small cluster in its lateral portion, corresponding to Brodmann areas 11/47, and a medial cluster in the transition of Brodmann areas 11 and 14, – in the medial part of the right amygdala, and in the dorsal part of the right postcentral gyrus

ROI analysis, *anger* scores: *anger* scores correlated positively with GM volume in the ventral striatum bilaterally (nuclei accumbens), in the right occipital fusiform gyrus and in the subgenual cortex (Brodmann areas 25 and 32)

ROI analysis, *disgust* scores: a positive correlation was found between *disgust* scores and GM volume in the dorsal ACC (Brodmann area 24)

ROI analysis, total Ekman scores: total emotion recognition scores correlated positively with GM volume in the dorsal ACC, in an area that partially overlaps the cluster of significant correlation between GM volume and *disgust* scores (p<0.05, FWE-corrected)

PD patients had significantly reduced GM volumes compared with controls in all of the clusters described above

	Structure	MNI coordinates of maxima (x,y,z)	Volume (mm^3)	r/p	Group comparison (t/p values)
**Sadness**	Right OFC (lateral)	40, 30, −20	280	.45/.025	2.182/.033*
	Right OFC (medial)	4, 42, −28	1008	.53/.013	2.246/.028*
	Right PCG	34, −32, 50	264	.49/.030	3.656/.001*
	Right amygdala	16, −6, −12	184	.49/.036	4.380/ <.001*
**Anger**	Right occipital fusiform gyrus	24, −64, −10	1480	.54/.017	3.664/.001*
	Subgenual cortex	6, 10, −10	1720	.64/.016	2.419/.019*
	Right NAcc	6, 4, −8	1192	.49/.001	.703/.485
	Left NAcc	−6, 4, −6	1328	.45/.010	3.094/.003*
**Disgust**	ACC	2, 16, 22	296	.51/.035	2.895/.005*
**Total Ekman**	ACC	2, 18, 24	160	.46/.038	2.594/.012*

WM:

Whole-FA skeleton voxelwise analysis revealed a strong positive correlation between *sadness* scores and FA levels in a cluster in the right frontal lobe WM, with its maximum located in the topography of the inferior fronto-occipital fasciculus but also involving the forceps minor and body of the corpus callosum (Fig. 2A–C). Intergroup analysis revealed that PD patients had significantly lower FA levels in this cluster than HC

Table 6. Clusters of significant positive correlation between FA and *sadness* scores ($p<0.05$, FWE−corrected).

Topography	MNI coordinates of maxima (x, y, z)	Cluster volumen (mm^3)	r/p	Group comparison (t/ p values)
**Right IFOF, right forceps minor**[a]	34, 37, 4	1312	.53/.033	1.856/.032*
**Body of corpus callosum, left centrum semiovale**	−9, 5, 17	228	.47/.046	2.195/.069
**Left ILF**[b]	−43, −24, −11	342	.59/.004	.029/.977
**Left IFOF**[b]	−40, −25, −5	454	.56/.004	.057/.955

IFOF: inferior fronto-occipital fasciculus. SLF: superior longitudinal fasciculus. ILF: inferior longitudinal fasciculus. "r": Pearson's correlation coefficients. "*" indicates significant ($p<0.05$) group differences in FA levels.

ROI analysis revealed additional areas of correlation between FA levels and *sadness* scores in the left temporal lobe white matter, comprising the topographies of the inferior fronto-occipital fasciculus and the inferior longitudinal fasciculus

No correlations were found between FA and other Ekman subscores or total Ekman scores either in whole-FA skeleton or in ROI analyses.

Tags: Emotion recognition, Imaging, behavioral

Subthalamic Deep Brain Stimulation: Mapping Non-Motor Outcomes to Structural Connections.

Item Type Journal Article
Author Garance M. Meyer
Author Ilkem Aysu Sahin
Author Barbara Hollunder
Author Konstantin Butenko
Author Nanditha Rajamani
Author Clemens Neudorfer
Author Lauren A. Hart
Author Jan Niklas Petry-Schmelzer
Author Haidar S. Dafsari
Author Michael T. Barbe
Author Veerle Visser-Vandewalle
Author Philip E. Mosley

Author Andreas Horn

Abstract In Parkinson's Disease (PD), deep brain stimulation of the subthalamic nucleus (STN-DBS) reliably improves motor symptoms, and the circuits mediating these effects have largely been identified. However, non-motor outcomes are more variable, and it remains unclear which specific brain circuits need to be modulated or avoided to improve them. Since numerous non-motor symptoms potentially respond to DBS, it is challenging to independently identify the circuits mediating each one of them. Data compression algorithms such as principal component analysis (PCA) may provide a powerful alternative. This study aimed at providing a proof of concept for this approach by mapping changes along extensive score batteries to a few anatomical fiber bundles and, in turn, estimating changes in individual scores based on stimulation of these tracts. Retrospective data from 56 patients with PD and bilateral STN-DBS was included. The patients had undergone comprehensive clinical assessments covering changes in appetitive behaviors, mood, anxiety, impulsivity, cognition, and empathy. PCA was implemented to identify the main dimensions of neuropsychiatric and neuropsychological outcomes. Using DBS fiber filtering, we identified the structural connections whose stimulation was associated with change along these dimensions. Then, estimates of individual symptom outcomes were derived based on the stimulation of these connections by inverting the PCA. Finally, changes along a specific non-motor score were estimated in an independent validation dataset (N = 68) using the tract model. Four principal components were retained, which could be interpreted to reflect (i) general non-motor improvement; (ii) improvement of mood and cognition and worsening of trait impulsivity; (iii) improvement of cognition; and (iv) improvement of empathy and worsening of impulsive-compulsive behaviors. Each component was associated with the stimulation of spatially segregated fiber bundles connecting regions of the frontal cortex with the subthalamic nucleus. The extent of stimulation of these tracts was able to explain significant amounts of variance in outcomes for individual symptoms in the original cohort (circular analysis), as well as in the rank of depression outcomes in the independent validation cohort. Our approach represents an innovative concept for mapping changes along extensive score batteries to a few anatomical fiber bundles and could pave the way toward personalized deep brain stimulation.

Date 2025 Apr 1
Language eng
License © 2025 The Author(s). Human Brain Mapping published by Wiley Periodicals LLC.
Extra Place: United States
Volume 46
Pages e70207
Publication Human brain mapping
DOI 10.1002/hbm.70207
Issue 5
Journal Abbr Hum Brain Mapp
ISSN 1097-0193 1065-9471
PMID 40193128
PMCID PMC11974458
Date Added 6.7.2025, 19:09:42
Modified 5.9.2025, 14:46:35

Notes:

Not Included: No SC aspect
Tags: EXCLUDED

Subthalamic Nucleus Deep Brain Stimulation Alters Prefrontal Correlates of Emotion Induction

Item Type Journal Article
Author Sarah K. B. Bick
Author Bradley S. Folley
Author Jutta S. Mayer
Author Sohee Park
Author P. David Charles
Author Corrie R. Camalier
Author Srivatsan Pallavaram
Author Peter E. Konrad
Author Joseph S. Neimat
Abstract ObjectivesDeep brain stimulation (DBS) of the subthalamic nucleus (STN) improves motor symptoms in advanced Parkinson's disease. STN DBS may also affect emotion, possibly by impacting a parallel limbic cortico-striatal circuit. The objective of this study was to investigate changes in prefrontal cortical activity related to DBS during an emotion induction task. Materials and MethodsWe used near infrared spectroscopy to monitor prefrontal cortex hemodynamic changes during an emotion induction task. Seven DBS patients were tested sequentially in the stimulation-on and stimulation-off states while on dopaminergic medication. Patients watched a series of positive, negative, and neutral videos. The general linear model was used to compare prefrontal oxygenated hemoglobin concentration between DBS states. ResultsDeep brain stimulation was correlated with prefrontal oxygenated hemoglobin changes relative to the stimulation off state in response to both positive and negative videos. These changes were specific to emotional stimuli and were not seen during neutral stimuli. ConclusionsThese results suggest that STN stimulation influences the prefrontal cortical representation of positive and negative emotion induction.
Date 2017-04
Language English
Extra Place: 111 RIVER ST, HOBOKEN 07030-5774, NJ USA Type: Article
Volume 20
Publisher WILEY
Pages 233-237
Publication NEUROMODULATION
DOI 10.1111/ner.12537
Issue 3
ISSN 1094-7159
Date Added 14.7.2025, 14:50:35
Modified 5.9.2025, 14:28:37

Notes:

Not Included: no HC, only ON-OFF DBS
Tags: EXCLUDED

Subthalamic nucleus oscillations during facial emotion processing and apathy in Parkinson's disease.

**Item Type**	Journal Article
**Author**	Jun Li
**Author**	Linbin Wang
**Author**	Yixin Pan
**Author**	Peng Huang
**Author**	Lu Xu
**Author**	Yuyao Zhang
**Author**	Dirk De Ridder
**Author**	Valerie Voon
**Author**	Dianyou Li
**Abstract**	BACKGROUND: Parkinson's disease (PD) is primarily characterized by motor symptoms, but patients also experience a relatively high prevalence of non-motor symptoms, including emotional and cognitive impairments. While the subthalamic nucleus (STN) is a common target for deep brain stimulation to treat motor symptoms in PD, its role in emotion processing is still under investigation. This study examines the subthalamic neural oscillatory activities during facial emotion processing and its association with affective characteristics. METHODS: Twenty PD patients who underwent subthalamic deep brain stimulation surgery performed a facial-expression-recognition task while STN local field potential (LFP) and frontal electroencephalography (EEG) were recorded. The facial-emotion-induced time-frequency decomposition of the STN-LFP and the frontal EEG, as well as the LFP-EEG coherence, were analyzed. Furthermore, the correlation between STN activities and affective characteristics was examined. RESULTS: Facial expressions elicited increased delta-theta-band and decreased alpha-beta-band activities in STN-LFP. Reduced alpha-beta-band LFP desynchronization was correlated with the severity of apathy. Increased theta-band and decreased alpha-beta-band EEG activities responded to facial emotion. Notably, lower coherence between STN-LFP and frontal EEG in delta-theta-band activity and alpha-band activity correlated with the degree of anhedonia. CONCLUSION: These results indicate that subthalamic activities during facial emotion processing are associated with apathy and anhedonia, emphasizing the cognitive-limbic function of STN and its role as a physiological target for apathy neuromodulation in PD.
**Date**	2025 Mar 15
**Language**	eng
**License**	Copyright © 2025 Elsevier B.V. All rights reserved.
**Extra**	Place: Netherlands
**Volume**	373
**Pages**	314-324
**Publication**	Journal of affective disorders
**DOI**	10.1016/j.jad.2025.01.005

Journal Abbr J Affect Disord
ISSN 1573-2517 0165-0327
PMID 39761756
Date Added 6.7.2025, 19:09:37
Modified 5.9.2025, 14:43:38

Notes:

Not Included: no comparison group

Tags: EXCLUDED

Subthalamic nucleus oscillations during vocal emotion processing are dependent of the motor asymmetry of Parkinson's disease.

Item Type Journal Article
Author Damien Benis
Author Claire Haegelen
Author Philippe Voruz
Author Jordan Pierce
Author Valérie Milesi
Author Jean-François Houvenaghel
Author Marc Vérin
Author Paul Sauleau
Author Didier Grandjean
Author Julie Péron
Abstract The subthalamic nucleus (STN) is involved in different aspects of emotional processes and more specifically in emotional prosody recognition. Recent studies on the behavioral effects of deep brain stimulation (DBS) in patients with Parkinson's disease (PD) have uncovered an asymmetry in vocal emotion decoding in PD, with left-onset PD patients showing deficits for the processing of happy voices. Whether and how PD asymmetry affects STN electrophysiological responses to emotional prosody, however, remains unknown. In the current study, local field potential activity was recorded from eight left- and six right-lateralized motor-onset PD patients (LOPD/ROPD) undergoing DBS electrodes implantation, while they listened to angry, happy and neutral voices. Time-frequency decomposition revealed that theta (2-6 Hz), alpha (6-12 Hz) and gamma (60-150 Hz) band responses to emotion were mostly bilateral with a differential pattern of response according to patient's sides-of onset. Conversely, beta-band (12-20 Hz and 20-30 Hz) emotional responses were mostly lateralized in the left STN for both patient groups. Furthermore, STN theta, alpha and gamma band responses to happiness were either absent (theta band) or reduced (alpha and gamma band) in the most affected STN hemisphere (contralateral to the side-of onset), while a late low-beta band left STN happiness-specific response was present in ROPD patients and did not occur in LOPD patients. Altogether, in this study, we demonstrate a complex pattern of oscillatory activity in the human STN in response to emotional voices and reveal a crucial influence of disease laterality on STN low-frequency oscillatory activity.
Date 2020 Nov 15

Language eng
License Copyright © 2020. Published by Elsevier Inc.
Extra Place: United States
Volume 222
Pages 117215
Publication NeuroImage
DOI 10.1016/j.neuroimage.2020.117215
Journal Abbr Neuroimage
ISSN 1095-9572 1053-8119
PMID 32745674
Date Added 6.7.2025, 19:09:38
Modified 5.9.2025, 14:28:23

Notes:

Not Included: no HC (only after DBS)

Tags: EXCLUDED

Subthalamic nucleus stimulation affects fear and sadness recognition in Parkinson's disease.

Item Type Journal Article
Author Julie Péron
Author Isabelle Biseul
Author Emmanuelle Leray
Author Siobhan Vicente
Author Florence Le Jeune
Author Sophie Drapier
Author Dominique Drapier
Author Paul Sauleau
Author Claire Haegelen
Author Marc Vérin
Abstract Bilateral subthalamic nucleus (STN) deep brain stimulation (DBS) in Parkinson's disease (PD) can produce emotional disorders that have been linked to disturbance of the STN's limbic territory. The aim of this study was to confirm the impairment of the recognition of facial emotions (RFE) induced by STN DBS, not only ruling out the effect of the disease's natural progression in relation to the effect of DBS, but also assessing the influence of modifications in dopamine replacement therapy (DRT) following STN DBS. RFE was investigated in 24 PD patients who underwent STN DBS and 20 PD patients treated with apomorphine. They were assessed 3 months before and after treatment. The 2 patient groups were compared with a group of 30 healthy matched controls. The results showed that RFE for negative emotions (fear and sadness) was impaired in only the STN DBS group in the posttreatment condition and was unrelated to DRT. Results confirm the selective reduction of RFE induced by STN DBS, due neither to the disease's natural progression nor to modifications in DRT.

**Date**	2010 Jan
**Language**	eng
**License**	Copyright 2009 APA, all rights reserved.
**Extra**	Place: United States
**Volume**	24
**Pages**	1-8
**Publication**	Neuropsychology
**DOI**	10.1037/a0017433
**Issue**	1
**Journal Abbr**	Neuropsychology
**ISSN**	1931-1559 0894-4105
**PMID**	20063943
**Date Added**	6.7.2025, 19:09:38
**Modified**	5.9.2025, 14:50:16

Notes:

Included

Sample characteristics

Size: 24 PD-DBS (pre-surgery), 20 PD, 30 HC

PD-type: NA

PD-duration: PD M = 11.3, SD = 5.2; PD-DBS M=11.9 SD=2.5

Medication: ON-state

Hoehn-Yahr: PD M = 1.7, Sd = 0.7; PD-DBS M=1 SD=0.6

UPDRS-3: PD M = 14.7, Sd = 9.5; PD-DBS M=8.2 SD=4.5

Gender (male): PD 8 males (40%); PD-DBS 17 males (71%)

Age: PD M = 64.3 SD = 10.8; PD-DBS M=59 SD=10.4

Other neurological disease (tumor, stroke, etc.): None

Other major psychopathology: None

Origin country (or ethnicity): France

method (Review, meta-analysis or observational and/or self-reported):

instruments used in order to quantify the variables

Social cognition aspect: Recognition of Facial Emotions (RFE)

Name of the task: NA

Type of stimulus [face/voice etc., Ekman faces/other etc.]: 55 computerized photographic slides of seven facial expressions . From Ekman

Task condition: (happiness, sadness, fear, surprise, disgust, anger, and neutral)

After observing the picture for 3 s, participants were prompted to give an answer (verbally) by choosing the most suitable response from the list of the emotions

Operationalization: Correct answers

Main findings related to the review's scope

In the pretreatment condition, no significant difference was found in RFE between the STN group, the APO group, and the HC group for any of the seven individual expressions or for the total score

Tags: Emotion Recognition, behavioral

Subthalamic nucleus stimulation affects orbitofrontal cortex in facial emotion recognition: a PET study.

**Item Type**	Journal Article
**Author**	F. Le Jeune
**Author**	J. Péron
**Author**	I. Biseul
**Author**	S. Fournier
**Author**	P. Sauleau
**Author**	S. Drapier
**Author**	C. Haegelen
**Author**	D. Drapier
**Author**	B. Millet
**Author**	E. Garin
**Author**	J.-Y. Herry
**Author**	C.-H. Malbert
**Author**	M. Vérin
**Abstract**	Deep brain stimulation (DBS) of the bilateral subthalamic nucleus (STN) in Parkinson's disease is thought to produce adverse events such as emotional disorders, and in a recent study, we found fear recognition to be impaired as a result. These changes have been attributed to disturbance of the STN's limbic territory and would appear to confirm that the negative emotion recognition network passes through the STN. In addition, it is now widely acknowledged that damage to the orbitofrontal cortex (OFC), especially the right side, can result in impaired recognition of facial emotions (RFE). In this context, we hypothesized that this reduced recognition of fear is correlated with modifications in the cerebral glucose metabolism of the right OFC. The objective of the present study was first, to reinforce our previous results by demonstrating reduced fear recognition in our Parkinson's disease patient group following STN DBS and, second, to correlate these emotional performances with glucose metabolism using (18)FDG-PET. The (18)FDG-PET and RFE tasks were both performed by a cohort of 13 Parkinson's disease patients 3 months before and 3 months after surgery for STN DBS. As predicted, we observed a significant reduction in fear recognition following surgery and obtained a positive correlation between these neuropsychological results and changes in glucose metabolism, especially in the right OFC. These results confirm the role of the STN as a key basal ganglia structure in limbic circuits.
**Date**	2008 Jun
**Language**	eng
**Extra**	Place: England
**Volume**	131
**Pages**	1599-1608
**Publication**	Brain : a journal of neurology
**DOI**	10.1093/brain/awn084
**Issue**	Pt 6
**Journal Abbr**	Brain
**ISSN**	1460-2156 0006-8950
**PMID**	18490359
**PMCID**	PMC2408938
**Date Added**	6.7.2025, 19:09:38
**Modified**	5.9.2025, 14:43:09

Notes:

Included

sample characteristics

size: 13 PD and 30 HC (matched for age, sex ratio, handedness and education level)

Parkinson's Disease type and duration: NA, Mduration = 10.9 SD=2.2

Medication: on mediaction

Hoehn-Yahr: M=1 SD=0.6

UPDRS-3: M=7.1 SD=3.8

Gender (male): 9 males (69%)

averaged ages (SD, range): M= 57 SD=7.8

other neurological disease (tumor, stroke, etc.): Standard selection and exclusion criteria for surgery were applied to all patients (Welter et al., 2002). brain atrophy was excluded on the basis of the preoperative MRI. excluded from the study, if face recognition as measured by the Benton Recognition Test was impaired.

other major psychopathology: Standard selection and exclusion criteria for surgery were applied to all patients (Welter et al., 2002).

origin country (or ethnicity): France

method observational and imaging

instruments used in order to quantify the variables

Social cognition aspect: emotion recognition

Name of the task: RFE (recognition of facial emotions)

type of stimulus [face/voice etc., Ekman faces/other etc.]: Ekman.

task condition: happiness, sadness, fear, surprise,disgust, anger and no emotion

operationalization: The percentage of correct responses

PET scan: F-FDG PET in a resting state with eyes open. PET measurements were performed using a dedicated Discovery ST PET scanner (GEMS, Milwaukee, USA) in 2D mode with an axial field of view of 15.2 cm. A 222–296M Bq injection. The data were analysed using SPM2 software. Normalized into standard stereotactic space according to Talairach and Tournoux's atlas (Talairach and Tournoux, 1988). statistical parametric mapping (SPM) software package, in line with Gispert et al. (2003), we created our own 18F-FDG template, using the images of 15 control subjects acquired in the same injection, acquisition and reconstruction conditions.

Main findings related to the review's scope

Behavioral: No significant difference was found in the pre-operative and HC groups for RFE, either for each of the six emotions or for the overall score.

**although imaging PET data are available, they were not collected as only post-DBS correlation were presented.

Tags: Emotion recognition, behavioral

Subthalamic nucleus stimulation affects theory of mind network: a PET study in Parkinson's disease.

Item Type Journal Article

Author Julie Péron
Author Florence Le Jeune
Author Claire Haegelen
Author Thibaut Dondaine
Author Dominique Drapier
Author Paul Sauleau
Author Jean-Michel Reymann
Author Sophie Drapier
Author Tiphaine Rouaud
Author Bruno Millet
Author Marc Vérin

Abstract BACKGROUND: There appears to be an overlap between the limbic system, which is modulated by subthalamic nucleus (STN) deep brain stimulation (DBS) in Parkinson's disease (PD), and the brain network that mediates theory of mind (ToM). Accordingly, the aim of the present study was to investigate the effects of STN DBS on ToM of PD patients and to correlate ToM modifications with changes in glucose metabolism. METHODOLOGY/PRINCIPAL FINDINGS: To this end, we conducted (18)FDG-PET scans in 13 PD patients in pre- and post-STN DBS conditions and correlated changes in their glucose metabolism with modified performances on the Eyes test, a visual ToM task requiring them to describe thoughts or feelings conveyed by photographs of the eye region. Postoperative PD performances on this emotion recognition task were significantly worse than either preoperative PD performances or those of healthy controls (HC), whereas there was no significant difference between preoperative PD and HC. Conversely, PD patients in the postoperative condition performed within the normal range on the gender attribution task included in the Eyes test. As far as the metabolic results are concerned, there were correlations between decreased cerebral glucose metabolism and impaired ToM in several cortical areas: the bilateral cingulate gyrus (BA 31), right middle frontal gyrus (BA 8, 9 and 10), left middle frontal gyrus (BA 6), temporal lobe (fusiform gyrus, BA 20), bilateral parietal lobe (right BA 3 and right and left BA 7) and bilateral occipital lobe (BA 19). There were also correlations between increased cerebral glucose metabolism and impaired ToM in the left superior temporal gyrus (BA 22), left inferior frontal gyrus (BA 13 and BA 47) and right inferior frontal gyrus (BA 47). All these structures overlap with the brain network that mediates ToM. CONCLUSION/ SIGNIFICANCE: These results seem to confirm that STN DBS hinders the ability to infer the mental states of others and modulates a distributed network known to

subtend ToM.

**Date**	2010 Mar 29
**Language**	eng
**Extra**	Place: United States
**Volume**	5
**Pages**	e9919
**Publication**	PloS one
**DOI**	10.1371/journal.pone.0009919
**Issue**	3
**Journal Abbr**	PLoS One
**ISSN**	1932-6203
**PMID**	20360963
**PMCID**	PMC2847915
**Date Added**	6.7.2025, 19:09:41
**Modified**	5.9.2025, 14:50:44

Notes:

Included

Sample characteristics

Size: 13 PD (same group pre- and post DBS OP), HC (comparable for handedness and gender ratio, age, education)

PD-type: NA

PD-duration: M = 10.5, SD = 3.6

Medication: Assessment in ON

Hoehn-Yahr (ON): M = 2.3, SD = 0.8

UPDRS-3 (ON): M = 31.4, SD = 12.2

Gender (male): 8 (62%)

Age: M = 53.3, SD = 8.5

Other neurological disease (tumor, stroke, etc.): None

Other major psychopathology: none

Origin country (or ethnicity): France

method behavioral

instruments used in order to quantify the variables

Social cognition aspect: ToM

Name of the task: Adapted Reading the mind in the eyes test

Type of stimulus [face/voice etc., Ekman faces/other etc.]: 17 photographs of the eye region of the faces of male and female actors

Task condition: Four adjectives corresponding to complex mental state descriptors (e.g. hateful, panicked) were printed on each slide, with one adjective in each corner and the photograph in the middle. One of these words (the target word) correctly described the mental state of the person in the photograph, while the others were included as foils

Operationalization: The maximum ‘‘Emotion score’’ on the test was therefore 17, which was converted into a percentage of correct responses

PET:

All subjects were studied using FDG PET in a resting state with eyes open. The data were analysed by means of statistical parametric mapping (SPM2 using software from the Wellcome Dept of Cognitive Neurology, London, UK) implemented in Matlab, Version 7 (Mathworks Inc., Sherborn, MA). Statistical parametric maps are spatially extended statistical processes that are used to characterize regionally specific effects in imaging data. They combine the general linear model (used to create the statistical map) and the theory of Gaussian fields to make statistical inferences about regional effects. All subject images were first realigned and spatially normalized into standard stereotactic space in accordance with the Talairach and Tournoux atlas [42]. Affine transformation was performed to determine the 12 optimum parameters for registering the brain image to the template, and the subtle

differences between the transformed image and the template were then removed using a nonlinear registration method. Finally, spatially normalized images were smoothed using a 12-mm full width at half-maximum isotropic Gaussian kernel to compensate for interindividual anatomical variability and to render the imaging data more normally distributed.

The PET data were used exclusively to compare pre- versus post-surgery measurements and to examine the relationship between changes in ToM scores and changes in brain metabolism, and were not compared to healthy controls or any other groups outside of the PD patients themselves.

Main findings related to the review's scope

In the preoperative condition, no significant difference was found between the PD and HC groups for any of the variables of the Reading the Mind in the Eyes Test (all measures p..5).

In the postoperative condition, a significant difference was found between the PD and HC groups for the Emotion score of the Reading the Mind in the Eyes Test (U = 42.50, p = .03).

Within the PD patient group, analyses revealed a significant difference between the preand postoperative conditions for the Emotion score of the Eyes test (z = 22.14, p = .03)

PET scan

When we studied postoperative increases in metabolism, two clusters were found to be significant at p,0.001, with multiplecomparison correction. Hypermetabolism was observed in the bilateral cerebellum and right inferior parietal lobule (BA 40).

Table 3. Summary of the analysis of correlations between decreased cerebral glucose metabolism and the emotion recognition task of the Reading the Mind in the Eyes Test ($p<0.005$, k>30).

Region	Talairach coordinates x	Talairach coordinates y	Talairach coordinates z
Right parietal lobe, postcentral gyrus, BA 3	14	−35	71
Right parietal lobe, precuneus, BA 7	12	−55	60
Limbic lobe, right posterior cingulate gyrus, BA 31	0	−31	35
Right frontal lobe, middle frontal gyrus, BA 9	52	21	30
Right frontal lobe, middle frontal gyrus, BA 8	34	25	43
Left occipital lobe, cuneus, BA 19	−14	−97	0
Right occipital lobe, cuneus, BA 19	26	−88	23
Right frontal lobe, middle frontal gyrus, BA 10	36	39	20
Left frontal lobe, left middle frontal gyrus, BA6	−10	−59	60
Left parietal lobe, precuneus, BA 7	−6	−61	60
Limbic lobe, left posterior cingulate gyrus, BA 31	−2	−37	42

doi:10.1371/journal.pone.0009919.t003

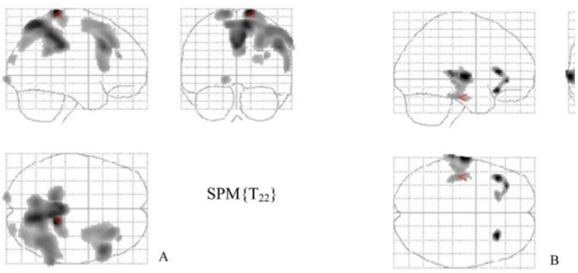

Figure 1. Statistical parametric maps displaying correlations between cerebral glucose metabolism and patients. (**A**) correlations between decreased cerebral glucose metabolism and impaired ToM, and (**B**) correlatio glucose metabolism and impaired ToM. Significant differences (two-tailed $p<0.005$, $k>30$) are shown in three ortho
doi:10.1371/journal.pone.0009919.g001

Tags: ToM, Imaging, behavioral

Subthalamic nucleus stimulation impairs emotional conflict adaptation in Parkinson's disease.

**Item Type**	Journal Article
**Author**	Friederike Irmen
**Author**	Julius Huebl
**Author**	Henning Schroll
**Author**	Christof Brücke
**Author**	Gerd-Helge Schneider
**Author**	Fred H. Hamker
**Author**	Andrea A. Kühn
**Abstract**	The subthalamic nucleus (STN) occupies a strategic position in the motor network, slowing down responses in situations with conflicting perceptual input. Recent evidence suggests a role of the STN in emotion processing through strong connections with emotion recognition structures. As deep brain stimulation (DBS) of the STN in patients with Parkinson's disease (PD) inhibits monitoring of perceptual and value-based conflict, STN DBS may also interfere with emotional conflict processing. To assess a possible interference of STN DBS with emotional conflict processing, we used an emotional Stroop paradigm. Subjects categorized face stimuli according to their emotional expression while ignoring emotionally congruent or incongruent superimposed word labels. Eleven PD patients ON and OFF STN DBS and eleven age-matched healthy subjects conducted the task. We found conflict-induced response slowing in healthy controls and PD patients OFF DBS, but not ON DBS, suggesting STN DBS to decrease adaptation to within-trial conflict. OFF DBS, patients showed more conflict-induced slowing for negative conflict stimuli, which was diminished by STN DBS. Computational modelling of STN influence on conflict adaptation disclosed DBS to interfere via increased baseline activity.
**Date**	2017 Oct 1
**Language**	eng
**License**	© The Author (2017). Published by Oxford University Press.
**Extra**	Place: England
**Volume**	12
**Pages**	1594-1604
**Publication**	Social cognitive and affective neuroscience
**DOI**	10.1093/scan/nsx090
**Issue**	10
**Journal Abbr**	Soc Cogn Affect Neurosci
**ISSN**	1749-5024 1749-5016
**PMID**	28985419
**PMCID**	PMC5647801
**Date Added**	6.7.2025, 19:09:39
**Modified**	5.9.2025, 14:40:19

Notes:

Included

sample characteristics

size: 11 PD and 11 HC

Parkinson's Disease type and duration: Idiopathic PD who undergo DBS, Mduration=11.5 SD=4.2

Medication: on medication

Hoehn-Yahr: NA

UPDRS-3: OFF DBS: M= 36.4 SD=9.1

Gender (male): 9 males (82%)

averaged ages (SD, range): M= 62 SD=6.4

other neurological disease (tumor, stroke, etc.): None

other major psychopathology: None

origin country (or ethnicity): Germany

method observational:

instruments used in order to quantify the variables

Social cognition aspect: emotion recognition

Name of the task: to the **emotional Stroop task** used by **Etkin et al. (2006)**

type of stimulus [face/voice etc., Ekman faces/other etc.]: black and white photographs of happy and sad faces taken from the 2D Facial Emotional Stimuli dataset. The faces were superimposed with the German words for 'joy' [Freude] or 'grief' [Trauer] in prominent red letters.

task condition: congruent and incongruent

operationalization: Stroop effect = RTincong - RTcong >> To compare the patient group ON and OFF DBS with the control group, reaction times of each patient (RTx) were standardized by subtracting the mean reaction times of the control group (RTcontrols) and dividing by the control group's standard deviation. The standardized mean reaction times (RTx.std) for each subject of the patient group thus described how far the subject's mean lay from the mean of the control group.

Main findings related to the review's scope

PD patients OFF DBS show a strong Stroop effect only for conflicting negative stimuli, while conflicting positive stimuli had no Stroop effect.

HC showed both positive and negative stroop effect, however no sig-dif between the effects size.

Tags: Emotion recognition, behavioral

Subthalamic nucleus stimulation in Parkinson disease induces apathy A PET study

Item Type Journal Article
Author F. Le Jeune
Author D. Drapier
Author A. Bourguignon
Author J. Peron
Author H. Mesbah
Author S. Drapier
Author P. Sauleau
Author C. Haegelen
Author D. Travers
Author E. Garin
Author C. H. Malbert
Author B. Millet
Author M. Verin
Abstract Objective: Apathy may be induced by subthalamic nucleus deep brain stimulation (STN-DBS) in Parkinson disease (PD). We therefore wished to test the hypothesis that apathy induced by STN-DBS correlates with changes in glucose metabolism, using (18)FDG-PET. Methods: Twelve patients with PD were assessed 3 months before (M - 3) and 3 months after (M = 3) STN-DBS with (18)FDG-PET and the Apathy Evaluation Scale. Results: Apathy had significantly worsened at M = 3 after STN-DBS. Positive correlations were observed between this variation in apathy scores and changes in glucose metabolism, especially in the right frontal middle gyrus (Brodmann area [BA] 10) and right inferior frontal gyrus (BA 46 and BA 47). Negative correlations between the two were observed in the right posterior cingulate gyrus (BA 31) and left medial frontal lobe (BA 9). Conclusion: These preliminary results confirm the role of the subthalamic nucleus in associative and limbic circuitry in humans and suggest that it is a key basal ganglia structure in motivation circuitry. Neurology (R) 2009; 73: 1746-1751
Date 2009-11-24
Language English
Extra Place: TWO COMMERCE SQ, 2001 MARKET ST, PHILADELPHIA, PA 19103 USA Type: Article
Volume 73
Publisher LIPPINCOTT WILLIAMS & WILKINS
Pages 1746-1751
Publication NEUROLOGY
DOI 10.1212/WNL.0b013e3181c34b34
Issue 21
ISSN 0028-3878
Date Added 14.7.2025, 14:50:42
Modified 14.7.2025, 14:50:42

Notes:

Not Included: Not assessing SC
Tags: EXCLUDED

Subthalamic Nucleus Stimulation Modulates Cognitive Theory of Mind in Parkinson's Disease.

**Item Type**	Journal Article
**Author**	Haoyun Xiao
**Author**	Liqin Lang
**Author**	Zheng Ye
**Author**	Jianjun Wu
**Abstract**	BACKGROUND: Theory of mind (ToM), the ability to infer others' mental state, is essential for social interaction among human beings. It has been widely reported that both cognitive (inference of knowledge) and affective (inference of emotion) components of ToM are disrupted in Parkinson's disease (PD). Previous studies usually focused on the involvement of the prefrontal cortex. OBJECTIVE: This study investigated the causal role of the subthalamic nucleus (STN), a key hub of the fronto-basal ganglia loops, in ToM. METHODS: Thirty-four patients with idiopathic PD (15 women, aged 62.2 ± 8.3 years) completed a Yoni task with deep brain stimulation (DBS) ON and OFF. The Yoni task was designed to separate the cognitive and affective components of ToM. Volumes of tissue activated (VTA) were computed for three subregions of the STN. RESULTS: DBS showed insignificant effects on ToM inference costs at the group level, which may be due to the large interindividual variability. The associative VTA correlated with the cognitive inference cost change but not the affective inference cost change. Patients with greater associative STN stimulation infer more slowly on cognitive ToM. Stimulating associative STN can adversely affect cognitive ToM in PD patients, especially in patients with a wide range of stimulation (≥0.157) or cognitive decline (Montreal Cognitive Assessment < 26). CONCLUSIONS: The associative STN plays a causal role in cognitive ToM in patients with PD. However, stimulating the associative STN likely impairs cognitive ToM and potentially leads to social interaction deficits in PD. © 2024 International Parkinson and Movement Disorder Society.
**Date**	2024 Jul
**Language**	eng
**License**	© 2024 International Parkinson and Movement Disorder Society.
**Extra**	Place: United States
**Volume**	39
**Pages**	1154-1165
**Publication**	Movement disorders : official journal of the Movement Disorder Society
**DOI**	10.1002/mds.29830
**Issue**	7
**Journal Abbr**	Mov Disord
**ISSN**	1531-8257 0885-3185
**PMID**	38696281
**Date Added**	6.7.2025, 19:09:41
**Modified**	5.9.2025, 15:01:14

Notes:

Not Included: No control group. Only PD pre-post STN

Tags: EXCLUDED

Subthalamic nucleus: A key structure for emotional component synchronization in humans

**Item Type**	Journal Article
**Author**	Julie Peron
**Author**	Sascha Fruehholz
**Author**	Marc Verin
**Author**	Didier Grandjean
**Abstract**	Affective neuroscience is concerned with identifying the neural bases of emotion. For historical and methodological reasons, models describing the brain architecture that supports emotional processes in humans have tended to neglect the basal ganglia, focusing instead on cortical and amygdalar mechanisms. Now, however, deep brain stimulation (DBS) of the subthalamic nucleus (STN), a neurosurgical treatment for Parkinson's disease and obsessive-compulsive disorder, is helping researchers explore the possible functional role of this particular basal ganglion in emotional processes. After reviewing studies that have used DBS in this way, we propose a model in which the STN plays a crucial role in producing temporally organized neural co-activation patterns at the cortical and subcortical levels that are essential for generating emotions and related feelings. (C) 2013 Elsevier Ltd. All rights reserved.
**Date**	2013-03
**Language**	English
**Extra**	Place: THE BOULEVARD, LANGFORD LANE, KIDLINGTON, OXFORD OX5 1GB, ENGLAND Type: Review
**Volume**	37
**Publisher**	PERGAMON-ELSEVIER SCIENCE LTD
**Pages**	358-373
**Publication**	NEUROSCIENCE AND BIOBEHAVIORAL REVIEWS
**DOI**	10.1016/j.neubiorev.2013.01.001
**Issue**	3
**ISSN**	0149-7634
**Date Added**	14.7.2025, 14:50:39
**Modified**	5.9.2025, 14:50:29

Notes:

Not Included: No empirical study

Tags: EXCLUDED

Subtypes of mild cognitive impairment in patients with Parkinson's disease: evidence from the LANDSCAPE study

**Item Type**	Journal Article
**Author**	Elke Kalbe
**Author**	Sarah Petra Rehberg
**Author**	Ines Heber
**Author**	Martin Kronenbuerger
**Author**	Jörg B Schulz
**Author**	Alexander Storch
**Author**	Katharina Linse
**Author**	Christine Schneider
**Author**	Susanne Gräber
**Author**	Inga Liepelt-Scarfone
**Author**	Daniela Berg
**Author**	Judith Dams
**Author**	Monika Balzer-Geldsetzer
**Author**	Rüdiger Hilker
**Author**	Carola Oberschmidt
**Author**	Karsten Witt
**Author**	Nele Schmidt
**Author**	Brit Mollenhauer
**Author**	Claudia Trenkwalder
**Author**	Annika Spottke
**Author**	Sandra Roeske
**Author**	Hans-Ulrich Wittchen
**Author**	Oliver Riedel
**Author**	Richard Dodel
**Date**	10/2016
**Language**	en
**Short Title**	Subtypes of mild cognitive impairment in patients with Parkinson's disease
**Library Catalog**	DOI.org (Crossref)
**URL**	https://jnnp.bmj.com/lookup/doi/10.1136/jnnp-2016-313838
**Accessed**	18.1.2026, 22:59:59
**Volume**	87
**Pages**	1099-1105
**Publication**	Journal of Neurology, Neurosurgery & Psychiatry
**DOI**	10.1136/jnnp-2016-313838
**Issue**	10
**Journal Abbr**	J Neurol Neurosurg Psychiatry
**ISSN**	0022-3050, 1468-330X
**Date Added**	18.1.2026, 22:59:59
**Modified**	18.1.2026, 22:59:59

Supervised learning for automatic emotion recognition in Parkinson’s disease through smartwatch signals

**Item Type**	Journal Article
**Author**	Lucia Pepa
**Author**	Luca Spalazzi
**Author**	Maria Gabriella Ceravolo
**Author**	Marianna Capecci
**Abstract**	People with Parkinson’s Disease (PwPD) usually experience several neuropsychiatric signs such as anxiety, depression, and negative emotions that contribute to disability and worsening of quality of life. Notwithstanding, the assessment of these symptoms are largely underrated, subjective and difficult due to a large overlapping with other PD symptoms, like hypomimia and bradikinesia. The aim and novelty of the current work is to study and validate a method for automatic emotion recognition in PwPD during daily living through autonomic signals acquired by acceptable and low-cost consumer technology. The best shallow learning algorithm and the best minimal feature set are individuated. 11 PwPD and 8 subjects with no history of neurological injury or illness were enrolled in the study. Participants were asked to watch video clips purposely selected to arouse emotions, and annotate arousal and valence of emotions triggered by video clips, while their heart rate, skin conductance, and temperature were recorded by a smartwatch. Smartwatch data was used for features extraction, while participants’ reported arousal and valence were used as gold-standard to train machine learning algorithms for emotion classification (low/high arousal, positive/negative valence). Different feature sets and different algorithms (i.e. decision tree (DT), random forest (RF), support vector machine (SVM), and multilayer perceptron (MLP)) were evaluated to find the best solution for each group of participant. In each group of participants, it was possible to find a combination of feature set and algorithm to reach a classification accuracy greater than 90%. The Random Forest reached the best performance in both groups and for both valence and arousal. For each classification task (valence or arousal, PwPD or controls), the best model was selected and the minimal feature set was found by performing a recursive feature elimination based on the Shapley value. A lower accuracy of appraisal emerged for arousal compared to valence. Obtained results showed the feasibility of automatic emotion recognition in PwPDs through autonomic signals. Autonomic dysfunction in PwPDs may explain the lower arousal accuracy. The findings warrant confirmation from trials on larger samples and there are open issues to be deepened in future work.
**Date**	2024
**URL**	https://www.sciencedirect.com/science/article/pii/S0957417424003397
**Volume**	249
**Pages**	123474
**Publication**	Expert Systems with Applications
**DOI**	https://doi.org/10.1016/j.eswa.2024.123474
**ISSN**	0957-4174
**Date Added**	6.7.2025, 19:12:36
**Modified**	5.9.2025, 14:49:58

Notes:

Not Included: no comp. group.

Study tries to identify emotions in PD patients
Tags: EXCLUDED

Test Retest Reliability of Measures Commonly Used to Measure Striatal Dysfunction across Multiple Testing Sessions: A Longitudinal Study

Item Type Journal Article
Author Clare E. Palmer
Author Douglas Langbehn
Author Sarah J. Tabrizi
Author Marina Papoutsi
Abstract Cognitive impairment is common amongst many neurodegenerative movement disorders such as Huntington's disease (HD) and Parkinson's disease (PD) across multiple domains. There are many tasks available to assess different aspects of this dysfunction, however, it is imperative that these show high test-retest reliability if they are to be used to track disease progression or response to treatment in patient populations. Moreover, in order to ensure effects of practice across testing sessions are not misconstrued as clinical improvement in clinical trials, tasks which are particularly vulnerable to practice effects need to be highlighted. In this study we evaluated test-retest reliability in mean performance across three testing sessions of four tasks that are commonly used to measure cognitive dysfunction associated with striatal impairment: a combined Simon Stop-Signal Task; a modified emotion recognition task; a circle tracing task; and the trail making task. Practice effects were seen between sessions 1 and 2 across all tasks for the majority of dependent variables, particularly reaction time variables; some, but not all, diminished in the third session. Good test-retest reliability across all sessions was seen for the emotion recognition, circle tracing, and trail making test. The Simon interference effect and stop-signal reaction time (SSRT) from the combined-Simon-Stop-Signal task showed moderate test-retest reliability, however, the combined SSRT interference effect showed poor test-retest reliability. Our results emphasize the need to use control groups when tracking clinical progression or use pre-baseline training on tasks susceptible to practice effects.
Date 2018-01-12
Language English
Extra Place: AVENUE DU TRIBUNAL FEDERAL 34, LAUSANNE, CH-1015, SWITZERLAND Type: Article
Volume 8
Publisher FRONTIERS MEDIA SA
Publication FRONTIERS IN PSYCHOLOGY
DOI 10.3389/fpsyg.2017.02363
ISSN 1664-1078
Date Added 14.7.2025, 14:50:34
Modified 5.9.2025, 14:48:51

Notes:

Not Included: No group comparison with HC
Tags: EXCLUDED

The amygdala of patients with Parkinson's disease is silent in response to fearful facial expressions

Item Type Journal Article
Author N Yoshimura
Author M Kawamura
Author Y Masaoka
Author I Homma
Abstract We previously found that patients with Parkinson's disease (PD) were impaired with respect to recognition of fear and disgust in facial expressions. To investigate the neural mechanisms that underlie this impairment, we recorded visual event-related potentials (ERPs) in response to the viewing of fearful facial expressions. Ten normal elderly volunteers and nine patients with PD were studied. Fearful, surprised, and neutral facial expressions were presented randomly for 500 ms each, with a probability of 0.1, 0.1, and 0.8, respectively. The locations of the components of the ERPs were analyzed using a scalp-skull-brain/dipole tracing method. The ERPs elicited in response to the facial stimuli consisted of a negative peak (N1), two positive peaks, and a subsequent slow negative shift. For N1, the equivalent current dipoles were concentrated in the fusiform gyrus, right superior temporal gyrus, parahippocampal gyrus, cingulate, cortex, and cerebellum, in normal subjects. In response to the fearful stimulus, dipoles were also generated from the amygdala in seven out of 10 normal subjects. In contrast, in patients with PD, N1 was centered bilaterally in the angular gyrus and supramarginal gyrus, and there was no neuronal activity in the amygdala. After N1, dipoles moved toward the frontal region in normal subjects, whereas they remained in the parietal lobes in patients with PD. These results suggest that neither the amygdala nor the temporal visual-associated cortices are involved in responding to fearful expressions in patients with PD. Corticostriatal connections may be variably affected by a lack of dopamine or by pathological changes in the amygdala. Thus, somatosensory recruitment may overcome the mild cognitive emotional deficits that are present in patients with PD owing to a dysfunction of the amygdala. (C) 2005 IBRO. Published by Elsevier Ltd. All rights reserved.
Date 2005
Language English
Extra Place: THE BOULEVARD, LANGFORD LANE, KIDLINGTON, OXFORD OX5 1GB, ENGLAND Type: Article
Volume 131
Publisher PERGAMON-ELSEVIER SCIENCE LTD
Pages 523-534
Publication NEUROSCIENCE
DOI 10.1016/j.neuroscience.2004.09.054
Issue 2
ISSN 0306-4522
Date Added 14.7.2025, 14:50:43

Modified 5.9.2025, 15:01:45

Notes:

Not Included: experimental paradigm doesn't assess SC

Tags: EXCLUDED

The combined effect of subthalamic nuclei deep brain stimulation and l-dopa increases emotion recognition in Parkinson's disease

Item Type Journal Article
Author Laurie Mondillon
Author Martial Mermillod
Author Serban C. Musca
Author Isabelle Rieu
Author Tiphaine Vidal
Author Patrick Chambres
Author Catherine Auxiette
Author Hélène Dalens
Author Louise Marie Coulangeon
Author Isabelle Jalenques
Author Jean-Jacques Lemaire
Author Miguel Ulla
Author Philippe Derost
Author Ana Marques
Author Franck Durif
Abstract Deep brain stimulation of the subthalamic nucleus (DBS) is a widely used surgical technique to suppress motor symptoms in Parkinson's disease (PD), and as such improves patients' quality of life. However, DBS may produce emotional disorders such as a reduced ability to recognize emotional facial expressions (EFE). Previous studies have not considered the fact that DBS and l-dopa medication can have differential, common, or complementary consequences on EFE processing. A thorough way of investigating the effect of DBS and l-dopa medication in greater detail is to compare patients' performances after surgery, with the two therapies either being administered ('on') or not administered ('off'). We therefore used a four-condition (l-dopa 'on'/DBS 'on', l-dopa 'on'/DBS 'off', l-dopa 'off'/DBS 'on', and l-dopa 'off'/DBS 'off') EFE recognition paradigm and compared implanted PD patients to healthy controls. The results confirmed those of previous studies, yielding a significant impairment in the detection of some facial expressions relative to controls. Disgust recognition was impaired when patients were 'off' l-dopa and 'on' DBS, and fear recognition impaired when 'off' of both therapies. More interestingly, the combined effect of both DBS and l-dopa administration seems much more beneficial for EFE recognition than the separate administration of each individual therapy. We discuss the implications of these findings in the light of the inverted U curve function that describes the differential effects of dopamine level on the right orbitofrontal cortex (OFC). We propose that, while l-dopa could "overdose" in

dopamine the ventral stream of the OFC, DBS would compensate for this over-activation by decreasing OFC activity, thereby restoring the necessary OFC–amygdala interaction. Another finding is that, when collapsing over all treatment conditions, PD patients recognized more neutral faces than the matched controls, a result that concurs with embodiment theories.

Date 2012
URL https://www.sciencedirect.com/science/article/pii/S0028393212003636
Volume 50
Pages 2869-2879
Publication Neuropsychologia
DOI https://doi.org/10.1016/j.neuropsychologia.2012.08.016
Issue 12
ISSN 0028-3932
Date Added 6.7.2025, 19:12:36
Modified 5.9.2025, 14:47:14

Notes:

Included

Sample characteristics

Size: 14 DBS-PD, 14 HC (matched on age, gender, education)

PD-type: NA

PD-duration: M = 12.36, SE = 0.71

Medication: OFF state

Hoehn-Yahr: NA

UPDRS-3: OFF-DBS M=32.64 SE=3.16

ON-DBS M=14.75 SE=1.72

Gender (male): 9 (64%)

Age: M = 62.2, SD = 6.3

Other neurological disease (tumor, stroke, etc.): None

Other major psychopathology: None

Origin country (or ethnicity): NA

method (Review, meta-analysis or observational and/or self-reported):

instruments used in order to quantify the variables

Social cognition aspect: Facial emotion recognition

Name of the task: NA

Type of stimulus [face/voice etc., Ekman faces/other etc.]: Emotional facial expressions extracted from The Karolinska directed emotional faces database. We selected color pictures of 16 different individuals (8 women and 8 men), each displaying 7 different emotional expressions (EFE), namely the 6 basic emotions; 100% emotional intensity) and a neutral expression (0% emotional intensity).

Task condition: anger, disgust, fear, happiness, sadness, and surprise

Operationalization: Correct answers

Main findings related to the review's scope

DBS-ON

PD were less accurate then HC in disgust. all the rest not sig

DBS-OFF

PD were less accurate then HC in fear. all the rest not sig

Tags: Emotion recognition, behavioral

The dopamine D1 receptor positive allosteric modulator, DETQ, improves cognition and social interaction in aged mice and enhances cortical and hippocampal acetylcholine efflux

**Item Type**	Journal Article
**Author**	Lakshmi Rajagopal
**Author**	Mei Huang
**Author**	Sanaz Mahjour
**Author**	Chelsea Ryan
**Author**	Ahmad Elzokaky
**Author**	Kjell A. Svensson
**Author**	H. Y. Meltzer
**Date**	2024-02-29
**Volume**	459
**Publication**	BEHAVIOURAL BRAIN RESEARCH
**DOI**	10.1016/j.bbr.2023.114766
**ISSN**	0166-4328
**Date Added**	14.7.2025, 14:48:39
**Modified**	5.9.2025, 14:52:32

Notes:

Not Included: Study in mice

Tags: EXCLUDED

The effect of cognitive status and visuospatial performance on affective theory of mind in Parkinson's disease.

**Item Type**	Journal Article
**Author**	Audrey McKinlay
**Author**	Michelle Albicini
**Author**	Phillip S. Kavanagh
**Abstract**	It is now well accepted that theory of mind (ToM) functioning is impaired in Parkinson's disease (PD) patients. However, what remain unknown are the functions that underlie this impairment. It has been suggested that cognitive skills may be key in this area of functioning; however, many of the cognitive tests used to assess this have relied on intact visuospatial abilities. This study aimed to examine whether deficits in ToM were generated by cognitive or visuospatial dysfunction and the mediating effect of visuospatial function on ToM performance. Fifty PD patients (31 male, 19 female; mean age = 66.34 years) and 49 healthy controls (16 male, 33 female; mean age = 67.29 years) completed a ToM task (reading the mind in the eyes) and visuospatial task (line orientation). The results revealed that current cognitive status was a significant predictor for performance on the ToM task, and that 54% of the total effect of cognitive status on ToM was mediated by visuospatial abilities. It was concluded that visuospatial functioning plays an important mediating role for the

relationship between executive dysfunction and affective ToM deficits in PD patients, and that visuospatial deficits may directly contribute to the presence of affective ToM difficulties seen in individuals with PD.

**Date**	2013
**Language**	eng
**Extra**	Place: New Zealand
**Volume**	9
**Pages**	1071-1076
**Publication**	Neuropsychiatric disease and treatment
**DOI**	10.2147/NDT.S49104
**Journal Abbr**	Neuropsychiatr Dis Treat
**ISSN**	1176-6328 1178-2021
**PMID**	24019747
**PMCID**	PMC3760454
**Date Added**	6.7.2025, 19:09:41
**Modified**	5.9.2025, 14:46:02

Notes:

Included

Sample characteristics

Size: 50 PD, 49 HC

PD-type: Idiopathic PD

PD-duration: NA

Medication: on state

Hoehn-Yahr: Range: 1-4

UPDRS-3: NA

Gender (male): 31 (61%)

Age: M = 66.34

Other neurological disease (tumor, stroke, etc.): None

Other major psychopathology: None (PD greater depressive symptomatology and lower level of cognitive functioning)

Origin country (or ethnicity): England

method (Review, meta-analysis or observational and/or self-reported):

instruments used in order to quantify the variables

Social cognition aspect: ToM

Name of the task: Reading the in in the eyes task (RMET)

Type of stimulus [face/voice etc., Ekman faces/other etc.]: 36 faces showing the eye-region

Task condition: Judge what the person in the picture might be feeling (4-way forced choice)

Operationalization: Correct answers

Main findings related to the review's scope

PD patients had significantly lower levels of ToM than controls.

Total effect of disease on ToM became nonsignificant with the introduction of cognitive functioning and visuospatial abilities into the model

Tags: ToM, behavioral

The Effect of Lateralization of Motor Onset and Emotional Recognition in PD Patients Using EEG.

Item Type Journal Article

Author R. Yuvaraj
Author M. Murugappan
Author R. Palaniappan
Abstract The objective of this research was to investigate the relationship between emotion recognition and lateralization of motor onset in Parkinson's disease (PD) patients using electroencephalogram (EEG) signals. The subject pool consisted of twenty PD patients [ten with predominantly left-sided (LPD) and ten with predominantly right-sided (RPD) motor symptoms] and 20 healthy controls (HC) that were matched for age and gender. Multimodal stimuli were used to evoke simple emotions, such as happiness, sadness, fear, anger, surprise, and disgust. Artifact-free emotion EEG signals were processed using the auto regressive spectral method and then subjected to repeated ANOVA measures. No group differences were observed across behavioral measures; however, a significant reduction in EEG spectral power was observed at alpha, beta and gamma frequency oscillations in LPD, compared to RPD and HC participants, suggesting that LPD patients (inferred right-hemisphere pathology) are impaired compared to RPD patients in emotional processing. We also found that PD-related emotional processing deficits may be selective to the perception of negative emotions. Previous findings have suggested a hemispheric effect on emotion processing that could be related to emotional response impairment in a subgroup of PD patients. This study may help in clinical practice to uncover potential neurophysiologic abnormalities of emotional changes with respect to PD patient's motor onset.
Date 2017 May
Language eng
Extra Place: United States
Volume 30
Pages 333-342
Publication Brain topography
DOI 10.1007/s10548-016-0524-0
Issue 3
Journal Abbr Brain Topogr
ISSN 1573-6792 0896-0267
PMID 27663236
Date Added 6.7.2025, 19:09:38
Modified 5.9.2025, 15:02:44

Notes:

Not Included: not on SC

Tags: EXCLUDED

The effects of medical comorbidity, cognition, and age on patient-reported outcomes in Parkinson's disease.

Item Type Journal Article
Author Haesung Kim
Author Lisa M. Shulman

Author Sunita Shakya
Author Ann Gruber-Baldini
Abstract OBJECTIVE: The purpose of this cross-sectional study was to compare the independent contributions of medical comorbidity, cognition, and age on patient-reported outcomes in Parkinson's disease (PD). METHODS: 572 PD patients completed the Patient-Reported Outcome Measurement Information System (PROMIS®)-29 v2.0 Profile (physical function, anxiety, depression, fatigue, sleep disturbance, satisfaction with participation in social roles, pain interference) and PROMIS Global Health (mental health and physical health) scales. Comorbidity was measured with the Cumulative Illness Rating Scale-Geriatric (CIRS-G) and cognition with the Montreal Cognitive Assessment (MoCA). Multiple regression models examined the 9 PROMIS measures as predicted by comorbidity, cognition, and age, adjusting for demographic and clinical characteristics (UPDRS and disease duration). RESULTS: Comorbidity was associated with poorer outcomes in all nine PROMIS domains. Cognition was associated with two of nine domains: physical function and anxiety. Age was associated with five domains: anxiety, depression, sleep disturbance, satisfaction with participation in social roles, and global mental health. Comorbidity showed greater effects on all nine domains than cognition or age (higher standardized beta coefficients). CONCLUSION: Medical comorbidity, cognition, and age have different impacts on patient-reported outcomes in PD. Medical comorbidity has a greater impact than either cognition or age on a range of patient-reported physical and mental health domains. Medical comorbidity is an important contributor to the patient's perspective of their physical and mental health.
Date 2023 Nov
Language eng
License Copyright © 2023 Elsevier Ltd. All rights reserved.
Extra Place: England
Volume 116
Pages 105892
Publication Parkinsonism & related disorders
DOI 10.1016/j.parkreldis.2023.105892
Journal Abbr Parkinsonism Relat Disord
ISSN 1873-5126 1353-8020
PMID 37837675
PMCID PMC10841750
Date Added 6.7.2025, 19:09:34
Modified 5.9.2025, 14:41:49

Notes:

Not Included: Not assessing SC

Assessing satisfaction from social role

Tags: EXCLUDED

The effects of right and left hemiparkinsonism on prosody

Item Type Journal Article

**Author**	Lee Xenakis Blonder
**Author**	Raquel E. Gur
**Author**	Ruben C. Gur
**Date**	2/1989
**Language**	en
**Library Catalog**	DOI.org (Crossref)
**URL**	https://linkinghub.elsevier.com/retrieve/pii/0093934X89900618
**Accessed**	11.8.2025, 18:41:39
**License**	https://www.elsevier.com/tdm/userlicense/1.0/
**Volume**	36
**Pages**	193-207
**Publication**	Brain and Language
**DOI**	10.1016/0093-934X(89)90061-8
**Issue**	2
**Journal Abbr**	Brain and Language
**ISSN**	0093934X
**Date Added**	11.8.2025, 18:41:39
**Modified**	11.8.2025, 18:41:39

Notes:

Included

sample characteristics

size: 21 PD (divided into LPD RPD) and 17 HC matched for age and education

Parkinson's Disease type and duration: idiopathic, Mduration=4.23 SD=3.16 1-15

Medication: on medication

Hoehn-Yahr: M=1.23 SD=0.53 1-3

UPDRS-3: NA

Gender (male): 11 males (52%)

averaged ages (SD, range): NA

other neurological disease (tumor, stroke, etc.): None

other major psychopathology: None

origin country (or ethnicity): USA

method observational

instruments used in order to quantify the variables

Social cognition aspect: emotion recognition

Name of the task: NA, Receptive Emotional Prosody

type of stimulus [face/voice etc., Ekman faces/other etc.]: prerecorded stimulus audiotape of 10 semantically neutral sentences and identify emotions conveyed prosodically (Weintraub et al., 1981)

task conditions: happy, sad, neutral, puzzled, angry

operationalization: accuracy

Name of the task: NA, Emotional and non emotional faces identification.

type of stimulus [face/voice etc., Ekman faces/other etc.]: Ekman faces. Judged photographs of 12 male and 12 female

task conditions: happy, sad, angry, frightened, disgusted, neutral

operationalization: accuracy >> identification and recognition (matching)

Main findings related to the review's scope

no significant differences.

Tags: Emotion recognition, behavioral

The FACE test: a new neuropsychological task to assess the recognition of complex mental states from faces.

Item Type Journal Article
Author Stefano Terruzzi
Author Giulia Funghi
Author Claudia Meli
Author Nicole Barozzi
Author Francesca Zappini
Author Costanza Papagno
Author Alessandra Dodich
Abstract BACKGROUND: Social cognition deficits are reported in several neurodegenerative diseases, including Parkinson's disease (PD). However, the availability of tasks for the clinical assessment is still limited, preventing the full characterization of socio-cognitive dysfunctions in neurological patients. This study aims to present a new task to assess the recognition of complex mental states from faces (FACE test), reporting normative data for the Italian population and an example of its clinical application to 40 PD patients. METHODS: Two-hundred twenty-nine Italian participants with at least 5 years of education were enrolled. Data were analyzed according to the method of equivalent scores; test-retest reliability and convergent validity were assessed. Two short versions of the FACE test were defined for clinical and research purposes. The prevalence of deficits in the FACE test was computed in the PD sample, as well as correlations with cognitive performance and diagnostic accuracy. RESULTS: Regression analyses revealed significant effects of demographic variables on FACE performance, with younger and more educated individuals showing higher scores. Twenty-eight percent of PD patients showed borderline/pathological performance, which was correlated with emotion recognition/attribution abilities, and attentive-executive functions. The FACE test was accurate (80%) in distinguishing PD patients with socio-cognitive dysfunctions from both controls and PD patients without emotion recognition/attribution difficulties. CONCLUSION: The FACE test represents a new tool assessing the ability to recognize complex mental states from facial expressions. Overall, these results support its use in both clinical and research settings, as well as the presence of affective processing deficits in a subsample of PD patients.
Date 2023 Jul
Language eng
License © 2023. The Author(s).
Extra Place: Italy
Volume 44
Pages 2339-2347
Publication Neurological sciences : official journal of the Italian Neurological Society and of the Italian Society of Clinical Neurophysiology
DOI 10.1007/s10072-023-06697-w
Issue 7
Journal Abbr Neurol Sci
ISSN 1590-3478 1590-1874
PMID 36849696
PMCID PMC10257594

Date Added 6.7.2025, 19:09:39
Modified 5.9.2025, 14:58:22

Notes:

Not Included: Study generated normative data. No group comparisons between HC and PD
Tags: EXCLUDED

The facial emotion recognition deficit in Parkinson's disease: Implications of a visual scanning strategy.

Item Type Journal Article
Author Nicoletta Ciccarelli
Author Isabella Anzuino
Author Fulvio Pepe
Author Eugenio Magni
Author Daniela Traficante
Author Maria Caterina Silveri
Abstract OBJECTIVE: We explored the relationship between a visual scanning strategy and a facial emotion recognition deficit in Parkinson's disease (PD). METHOD: Thirty nondemented PD patients (balanced for symptom side at onset) and 20 age, education and gender-matched healthy controls (HC) were enrolled. The PD group underwent a comprehensive neuropsychological battery also exploring the executive functions. In both groups, eye movements were recorded while subjects categorized facial emotion from Ekman's 60-faces test. We were particularly interested in the location of fixations on facial pictures (top vs. bottom) and in emotional valence (positive vs. negative). We also compared performance of the two groups on a verbal emotion attribution task. RESULTS: Compared to HC, PD patients performed worse on visual recognition of negative emotions such as anger, fear, and sadness (where the upper part of the face is more informative than the lower part); the two groups did not differ on the verbal emotion attribution task. HC modified their visual scanning strategy (both number and overall time duration of fixations) according to the valence of the emotion; by contrast, PD showed the same pattern regardless of the valence. In the PD group, accuracy in the visual recognition of negative emotions and fixation pattern correlated with performance on tasks exploring executive functions; however, no associations were observed with severity of motor state. CONCLUSIONS: Our results suggest that visual scanning strategy contributes significantly to the facial emotion recognition deficit of PD patients, especially at a "high level" related to cognitive control of eye movements. (PsycInfo Database Record (c) 2022 APA, all rights reserved).
Date 2022 May
Language eng
Extra Place: United States
Volume 36
Pages 279-287
Publication Neuropsychology
DOI 10.1037/neu0000802

Issue 4
Journal Abbr Neuropsychology
ISSN 1931-1559 0894-4105
PMID 35286104
Date Added 6.7.2025, 19:09:35
Modified 5.9.2025, 14:31:02

Notes:

Included

sample characteristics

size: 30 PD and 20 HC (matched for age, years of education, gender, and MOCA)

Parkinson's Disease type and duration: NA, Mduration= 5.50 SD= 3.48

Medication: on medication

Hoehn-Yahr: NA

UPDRS-3: M= 23.63 SD=8.42

Gender (male): 19 males (63%)

averaged ages (SD, range): M= 68.50 (*SD* = 7.38)

other neurological disease (tumor, stroke, etc.): None

other major psychopathology: None

origin country (or ethnicity): Italy

method observational

instruments used in order to quantify the variables

Social cognition aspect: Emotion recognition

Name of the task: Ekman 60 Faces Test

type of stimulus [face/voice etc., Ekman faces/other etc.]: Ekman faces

task condition: happiness, sadness, anger, disgust, fear, and surprise

Operationalization: Accuracy

Name of the task: from the Sartori Social Intelligence Battery, B subtest—Emotions Attribution Test

type of stimulus [face/voice etc., Ekman faces/other etc.]: the examiner reads short scenes (e.g., "Mario has been called in to his boss. His boss says "You're fired") and the subject has to indicate the emotion (i.e., happiness, sadness, anger, disgust, fear, envy or embarrassment) the main character is feeling.

operationalization: accuracy and eye-movement

eye-movement: eye-tracking device. Each picture was displayed on a 22-inch monitor, connected to a Dell Notebook PC W76CU interfaced with an SMI (Senso-Motoric Instruments) RED500 device that has high spatial (<0.4° of visual angle) and temporal (500 Hz) resolution. Viewing was binocular and the experiment was implemented and run through the Experiment Center 3.0 software (2010). Participants were seated approximately 60 cm from the monitor and the eye tracker was calibrated by asking the subject to track a black dot moving across the screen to five different locations before the experiment began.

Main findings related to the review's scope

Compared to HC, PD patients performed worse on visual recognition of negative emotions such as anger, fear, and sadness (where the upper part of the face is more informative than the lower part); the two groups did not differ on the verbal emotion attribution task. HC modified their visual scanning strategy (both number and overall time duration of fixations) according to the valence of the emotion; by contrast, PD showed the same pattern regardless of the valence.

Ekamn: the total score of the PD group (*p* = .013) was significantly worse than that of the HC as were the anger (*p* = .012) and fear (*p* = .017) subscores. No difference was found for the sadness, disgust, happiness, and surprise subscores.

Verbal Emotion Attribution Test: no significant effects, besides emotion.

eye-movement: PD patients, the number of fixations was smaller for positive than for negative emotions, but there was no significant difference in the number of fixations at the top and the bottom part of the face, irrespective of the emotional valence.

Gaze duration provides a similar pattern of results

Tags: Emotion recognition, ToM, behavioral

The functional correlates of face perception and recognition of emotional facial expressions as evidenced by fMRI

Item Type Journal Article

Author M. Jehna
Author C. Neuper
Author A. Ischebeck
Author M. Loitfelder
Author S. Ropele
Author C. Langkammer
Author F. Ebner
Author S. Fuchs
Author R. Schmidt
Author F. Fazekas
Author C. Enzinger

Abstract Recognition and processing of emotional facial expression are crucial for social behavior and employ higher-order cognitive and visual working processes. In neuropsychiatric disorders, impaired emotion recognition most frequently concerned three specific emotions, i.e., anger, fear, and disgust. As incorrect processing of (neutral) facial stimuli per se might also underlie deficits in the recognition of emotional facial expressions, we aimed to assess all these aspects in one experiment. We therefore report here a functional magnetic resonance imaging (fMRI) paradigm for parallel assessment of the neural correlates of both the recognition of neutral faces and the three clinically most relevant emotions for future use in patients with neuropsychiatric disorders. FMRI analyses were expanded through comparisons of the emotional conditions with each other. The differential insights resulting from

these two analyses strategies are compared and discussed. 30 healthy participants (21 F/9 M; age 36.3±14.3, 17–66years) underwent fMRI and behavioral testing for non-emotional and emotional face recognition. Recognition of neutral faces elicited activation in the fusiform gyri. Processing angry faces led to activation in left middle and superior frontal gyri and the anterior cingulate cortex. There was considerable heterogeneity regarding the fear versus neutral contrast, resulting in null effects for this contrast. Upon recognition of disgust, activation was noted in bilateral occipital, in the fronto-orbital cortex and in the insula. Analyzing contrasts between emotional conditions showed similar results (to those of contrasting with reference conditions) for separated emotional network patterns. We demonstrate here that our paradigm reproduces single aspects of separate previous studies across a cohort of healthy subjects, irrespective of age. Our approach might prove useful in future studies of patients with neurologic disorders with potential effect on emotion recognition.

Date 2011
URL https://www.sciencedirect.com/science/article/pii/S0006899311006743
Volume 1393
Pages 73-83
Publication Brain Research
DOI https://doi.org/10.1016/j.brainres.2011.04.007
ISSN 0006-8993
Date Added 6.7.2025, 19:12:34
Modified 5.9.2025, 14:40:37

Notes:

Not Included: Not on PD.

Maybe generally important?

Tags: EXCLUDED

The Impact of Clinical and Cognitive Variables on Social Functioning in Parkinson's Disease: Patient versus Examiner Estimates

Item Type Journal Article
Author Patrick McNamara
Author Karina Stavitsky
Author Raymon Durso
Author Erica Harris
Abstract Purpose. To assess the impact of clinical variables on social skills and behaviors in Parkinson's disease (PD) patients and patient versus examiner estimates of social functioning. Methods. Twenty-eight patients with PD and 32 controls with chronic disease were assessed with a battery of neuropsychologic, personality, mood, and social function tests. Results. Patients' estimates of their own social functioning were not significantly different from examiners' estimates. The impact of clinical variables on social functioning in PD revealed depression to be the strongest association of social functioning in PD on both the patient and the examiner version of the Social Adaptation Self-Evaluation Scale. Conclusions. PD patients appear to be well aware of their social strengths and weaknesses. Depression and motor symptom severity are

significant predictors of both self- and examiner reported social functioning in patients with PD. Assessment and treatment of depression in patients with PD may improve social functioning and overall quality of life.

Date 2010
Language English
Extra Place: ADAM HOUSE, 3RD FLR, 1 FITZROY SQ, LONDON, W1T 5HF, ENGLAND Type: Article
Volume 2010
Publisher HINDAWI LTD
Publication PARKINSONS DISEASE
DOI 10.4061/2010/263083
ISSN 2090-8083
Date Added 14.7.2025, 14:50:42
Modified 14.7.2025, 14:50:42

Notes:

Not Included: No group comparisons reported

Tags: EXCLUDED

The impact of deep brain stimulation of the subthalamic nucleus on facial emotion recognition in patients with Parkinson's disease.

Item Type Journal Article
Author Annelien A. Duits
Author Eva M. de Ronde
Author R. Saman Vinke
Author Sandra H. Vos
Author Rianne A. J. Esselink
Author Roy P. C. Kessels
Abstract Deep brain stimulation (DBS) of the subthalamic nucleus (STN) is successful in patients with advanced Parkinson's disease (PD) but may worsen cognitive outcome, including facial emotion recognition (FER). Data-analyses on 59 consecutive PD patients with complete pre- and postoperative assessments, using a sensitive FER test, showed no changes in FER 1 year after STN-DBS surgery, both after group and individual analyses. These findings do however not exclude the impact of FER in and on itself on the outcome after STN-DBS.
Date 2024 Mar
Language eng
License © 2023 The Authors. Journal of Neuropsychology published by John Wiley & Sons Ltd on behalf of The British Psychological Society.
Extra Place: England
Volume 18 Suppl 1
Pages 134-141
Publication Journal of neuropsychology

DOI 10.1111/jnp.12336
Journal Abbr J Neuropsychol
ISSN 1748-6653 1748-6645
PMID 37353988
Date Added 6.7.2025, 19:09:38
Modified 5.9.2025, 14:34:26

Notes:

Not Included: not comparing HC to PD, only longitudinal effect of DBS
Tags: EXCLUDED

The impact of executive dysfunctions on Theory of Mind abilities in Parkinson's disease.

Item Type Journal Article
Author Gianpaolo Maggi
Author Diana Di Meglio
Author Carmine Vitale
Author Marianna Amboni
Author Ignacio Obeso
Author Gabriella Santangelo
Abstract Theory of Mind (ToM) is the ability to infer and reason about others' mental states, a process impaired by Parkinson's disease (PD). ToM performance in PD seems to be strongly related to executive functioning but the exact nature of this relationship is still unclear. We aim to investigate the direct impact of several executive dysfunctions on ToM deficits (Affective and Cognitive ToM) in PD patients. Sixty-eight PD patients underwent neuropsychological tests evaluating executive control such as inhibition, cognitive flexibility, processing speed or working memory and Cognitive and Affective ToM. We divided participants into two groups based on their performance on executive tests: PD patients with poor executive functioning (PD-EF-) and those with preserved executive functioning (PD-EF+). To explore the direct impact of executive subdomains on ToM abilities, two mediation models were executed in the whole sample. We found that PD patients with poor executive functioning reported poorer scores on Affective and Cognitive ToM tasks than PD patients with preserved executive functions, controlling for age and education. Moreover, parallel mediation models, conducted in the whole sample, indicated that performance on phonological fluency mediated the relationships between educational level and both Affective and Cognitive ToM, controlling the effect of other executive tests. These findings further support the idea that executive functions are crucial in ToM processes. Particularly, phonological fluency, whose execution requires both verbal abilities and cognitive flexibility, mediated ToM performance controlling the effect of other executive functions. The identification of neuropsychological processes underpinning ToM abilities might represent a plausible target for cognitive training to strengthen ToM abilities in PD.
Date 2022 Nov 5
Language eng

License Copyright © 2022 Elsevier Ltd. All rights reserved.
Extra Place: England
Volume 176
Pages 108389
Publication Neuropsychologia
DOI 10.1016/j.neuropsychologia.2022.108389
Journal Abbr Neuropsychologia
ISSN 1873-3514 0028-3932
PMID 36206823
Date Added 6.7.2025, 19:09:40
Modified 5.9.2025, 14:44:32

Notes:

Not Included: no comp. group.

correlate with EF

Tags: EXCLUDED

The influence of facial masking and sex on older adults' impressions of individuals with Parkinson's disease.

Item Type Journal Article
Author Amanda R. Hemmesch
Author Linda Tickle-Degnen
Author Leslie A. Zebrowitz
Date 2009
Language en
Library Catalog Crossref
URL https://doi.apa.org/doi/10.1037/a0016105
Accessed 13.7.2025, 20:22:06
Volume 24
Publisher American Psychological Association (APA)
Pages 542-549
Publication Psychology and Aging
DOI 10.1037/a0016105
Issue 3
ISSN 1939-1498, 0882-7974
Date Added 13.7.2025, 20:22:06
Modified 13.7.2025, 20:22:06

Notes:

Not Included: not on SC
Tags: EXCLUDED

The mirror system, theory of mind and Parkinson's disease.

**Item Type**	Journal Article
**Author**	Manuel Alegre
**Author**	Jorge Guridi
**Author**	Julio Artieda
**Abstract**	The mirror system includes a group of neurons in the monkey cortex that discharge both when a movement is executed and when that same movement is observed. An equivalent system in humans has been proposed to mediate action and emotion understanding, being therefore related to theory of mind. Theory of mind (TOM) is the ability to infer our own or, more frequently, other person's mental states. It is severely impaired in disorders of the autistic spectrum, but it is also affected in other neurological diseases including Parkinson's disease (PD). Two recent studies have shown that the basal ganglia may be involved in action observation, as the subthalamic nucleus shows changes in activity during movement observation similar to those observed during movement execution. These findings suggest that the basal ganglia may be involved in mirror circuit activity, which might be affected in PD in a similar way to normal movement execution. Given the relationship between the mirror system and theory of mind, we hypothesize that TOM deficits in PD might be at least partially mediated by mirror system dysfunction.
**Date**	2011 Nov 15
**Language**	eng
**License**	Copyright © 2011 Elsevier B.V. All rights reserved.
**Extra**	Place: Netherlands
**Volume**	310
**Pages**	194-196
**Publication**	Journal of the neurological sciences
**DOI**	10.1016/j.jns.2011.07.030
**Issue**	1-2
**Journal Abbr**	J Neurol Sci
**ISSN**	1878-5883 0022-510X
**PMID**	21839480
**Date Added**	6.7.2025, 19:09:40
**Modified**	5.9.2025, 14:25:29

Notes:

Not Included: not an empirical paper
Tags: EXCLUDED

The moral brain and moral behaviour in patients with Parkinson's disease: a review of the literature

Item Type Journal Article
Author Patrick Santens
Author Giel Vanschoenbeek
Author Marijke Miatton
Author Miet De Letter
Abstract Morality is a complex and versatile concept that necessitates the integrated activity of multiple interacting networks in the brain. Numerous cortical and subcortical areas, many of which are implicated in either emotional and cognitive control or Theory of Mind, are involved in the processing of moral behaviour. Different methods have been used to investigate various aspects of morality, which has lead to confusing and sometimes opposing results. Emotional, cognitive and personality changes have long been recognized in Parkinson's disease (PD) patients, suggesting a potential impact on moral aspects of behaviour in daily living situations. Alterations in social cognition have been described in all stages of PD but these are rather directly related to PD pathology and not to dopaminergic or DBS treatment. There are no convincing data supporting the hypothesis that dopaminergic treatment or deep brain stimulation of the STN per se interfere with morality in PD patients, although subgroups of patients may display socially unacceptable behaviour. Research in social cognition in PD patients is a fascinating topic that needs further attention in view of the impact on quality of life for PD patients and their caregivers.
Date 2018-09
Language English
Extra Place: TIERGARTENSTRASSE 17, D-69121 HEIDELBERG, GERMANY Type: Review
Volume 118
Publisher SPRINGER HEIDELBERG
Pages 387-393
Publication ACTA NEUROLOGICA BELGICA
DOI 10.1007/s13760-018-0986-9
Issue 3
ISSN 0300-9009
Date Added 14.7.2025, 14:50:34
Modified 5.9.2025, 14:55:27

Notes:

Not Included: Not a systematic Review
Tags: EXCLUDED

The neurobiology of social cognition

Item Type Journal Article
Author Ralph Adolphs

**Date**	4/2001
**Language**	en
**Library Catalog**	DOI.org (Crossref)
**URL**	https://linkinghub.elsevier.com/retrieve/pii/S0959438800002026
**Accessed**	19.1.2026, 7:19:20
**License**	https://www.elsevier.com/tdm/userlicense/1.0/
**Volume**	11
**Pages**	231-239
**Publication**	Current Opinion in Neurobiology
**DOI**	10.1016/S0959-4388(00)00202-6
**Issue**	2
**Journal Abbr**	Current Opinion in Neurobiology
**ISSN**	09594388
**Date Added**	19.1.2026, 7:19:20
**Modified**	19.1.2026, 7:19:20

The place of dopamine in the cortico-basal ganglia circuit

**Item Type**	Journal Article
**Author**	S.N. Haber
**Date**	12/2014
**Language**	en
**Library Catalog**	DOI.org (Crossref)
**URL**	https://linkinghub.elsevier.com/retrieve/pii/S0306452214008707
**Accessed**	19.1.2026, 16:51:38
**License**	https://www.elsevier.com/tdm/userlicense/1.0/
**Volume**	282
**Pages**	248-257
**Publication**	Neuroscience
**DOI**	10.1016/j.neuroscience.2014.10.008
**Journal Abbr**	Neuroscience
**ISSN**	03064522
**Date Added**	19.1.2026, 16:51:38
**Modified**	19.1.2026, 16:51:38

The PRISMA 2020 statement: an updated guideline for reporting systematic reviews

**Item Type**	Journal Article
**Author**	Matthew J Page
**Author**	Joanne E McKenzie
**Author**	Patrick M Bossuyt
**Author**	Isabelle Boutron

**Author**	Tammy C Hoffmann
**Author**	Cynthia D Mulrow
**Author**	Larissa Shamseer
**Author**	Jennifer M Tetzlaff
**Author**	Elie A Akl
**Author**	Sue E Brennan
**Author**	Roger Chou
**Author**	Julie Glanville
**Author**	Jeremy M Grimshaw
**Author**	Asbjørn Hróbjartsson
**Author**	Manoj M Lalu
**Author**	Tianjing Li
**Author**	Elizabeth W Loder
**Author**	Evan Mayo-Wilson
**Author**	Steve McDonald
**Author**	Luke A McGuinness
**Author**	Lesley A Stewart
**Author**	James Thomas
**Author**	Andrea C Tricco
**Author**	Vivian A Welch
**Author**	Penny Whiting
**Author**	David Moher
**Date**	2021-03-29
**Language**	en
**Short Title**	The PRISMA 2020 statement
**Library Catalog**	DOI.org (Crossref)
**URL**	https://www.bmj.com/lookup/doi/10.1136/bmj.n71
**Accessed**	19.1.2026, 7:41:02
**Pages**	n71
**Publication**	BMJ
**DOI**	10.1136/bmj.n71
**Journal Abbr**	BMJ
**ISSN**	1756-1833
**Date Added**	19.1.2026, 7:41:02
**Modified**	19.1.2026, 7:41:02

The recognition of facial emotion expressions in Parkinson's disease.

**Item Type**	Journal Article
**Author**	Francesca Assogna
**Author**	Francesco E. Pontieri
**Author**	Carlo Caltagirone
**Author**	Gianfranco Spalletta

Abstract A limited number of studies in Parkinson's Disease (PD) suggest a disturbance of recognition of facial emotion expressions. In particular, disgust recognition impairment has been reported in unmedicated and medicated PD patients. However, the results are rather inconclusive in the definition of the degree and the selectivity of emotion recognition impairment, and an associated impairment of almost all basic facial emotions in PD is also described. Few studies have investigated the relationship with neuropsychiatric and neuropsychological symptoms with mainly negative results. This inconsistency may be due to many different problems, such as emotion assessment, perception deficit, cognitive impairment, behavioral symptoms, illness severity and antiparkinsonian therapy. Here we review the clinical characteristics and neural structures involved in the recognition of specific facial emotion expressions, and the plausible role of dopamine transmission and dopamine replacement therapy in these processes. It is clear that future studies should be directed to clarify all these issues.

Date 2008 Nov
Language eng
Extra Place: Netherlands
Volume 18
Pages 835-848
Publication European neuropsychopharmacology : the journal of the European College of Neuropsychopharmacology
DOI 10.1016/j.euroneuro.2008.07.004
Issue 11
Journal Abbr Eur Neuropsychopharmacol
ISSN 0924-977X
PMID 18707851
Date Added 6.7.2025, 19:09:35
Modified 5.9.2025, 14:27:02

Notes:

Not Included: not a systematic review
Tags: EXCLUDED

The relationship between executive functions and fluid intelligence in Parkinson's disease.

Item Type Journal Article
Author M. Roca
Author F. Manes
Author A. Chade
Author E. Gleichgerrcht
Author O. Gershanik
Author G. G. Arévalo
Author T. Torralva
Author J. Duncan

Abstract BACKGROUND: We recently demonstrated that decline in fluid intelligence is a substantial contributor to frontal deficits. For some classical 'executive' tasks, such as the Wisconsin Card Sorting Test (WCST) and Verbal Fluency, frontal deficits were entirely explained by fluid intelligence. However, on a second set of frontal tasks, deficits remained even after statistically controlling for this factor. These tasks included tests of theory of mind and multitasking. As frontal dysfunction is the most frequent cognitive deficit observed in early Parkinson's disease (PD), the present study aimed to determine the role of fluid intelligence in such deficits. METHOD: We assessed patients with PD (n=32) and control subjects (n=22) with the aforementioned frontal tests and with a test of fluid intelligence. Group performance was compared and fluid intelligence was introduced as a covariate to determine its role in frontal deficits shown by PD patients. RESULTS: In line with our previous results, scores on the WCST and Verbal Fluency were closely linked to fluid intelligence. Significant patient-control differences were eliminated or at least substantially reduced once fluid intelligence was introduced as a covariate. However, for tasks of theory of mind and multitasking, deficits remained even after fluid intelligence was statistically controlled. CONCLUSIONS: The present results suggest that clinical assessment of neuropsychological deficits in PD should include tests of fluid intelligence, together with one or more specific tasks that allow for the assessment of residual frontal deficits associated with theory of mind and multitasking.

Date 2012 Nov
Language eng
Extra Place: England
Volume 42
Pages 2445-2452
Publication Psychological medicine
DOI 10.1017/S0033291712000451
Issue 11
Journal Abbr Psychol Med
ISSN 1469-8978 0033-2917
PMID 22440401
PMCID PMC3466050
Date Added 6.7.2025, 19:09:42
Modified 5.9.2025, 14:53:15

Notes:

Not Included: Did not study SC
Tags: EXCLUDED

The Relationship between Motor Symptoms, Signs, and Parkinsonism with Facial Emotion Recognition Deficits in Individuals with 22q11.2 Deletion Syndrome at High Genetic Risk for Psychosis

Item Type Journal Article
Author Tommaso Accinni

Author Martina Fanella
Author Marianna Frascarelli
Author Antonino Buzzanca
Author Georgios D. Kotzalidis
Author Carolina Putotto
Author Bruno Marino
Author Alessia Panzera
Author Antonella Moschillo
Author Massimo Pasquini
Author Massimo Biondi
Author Carlo Di Bonaventura
Author Fabio Di Fabio

Abstract Background. The 22q11.2 Deletion Syndrome (22q11.2DS) is a genetic condition at high risk of developing both psychosis and motor disorders. Social Cognition (SC) deficits have been associated not only with schizophrenia but also with Parkinson's disease (PD). The present study assessed SC deficits in 22q11.2DS and investigated the interaction between motor symptoms and deficits in Facial Emotion Expressions (FEE) recognition and in Theory of Mind (ToM) tasks in people with 22q11.2DS. Methods. We recruited 38 individuals with 22q11.2DS without psychosis (N=38, DEL) and 18 with 22q11.2DS and psychosis (N=18, DEL_SCZ). The Positive And Negative Syndrome Scale (PANSS), Ekman's 60 Faces Test (EK-60F), the Awareness of Social Inference Test (TASIT EmRec), and the Movement Disorder Society-Unified Parkinson's Disease Rating Scale part III (UPDRS III) were administered. Correlations were sought between UPDRS III and both TASIT EmRec and EK-60F scores. Analyses were conducted separately for each psychopathological subgroup. Results. Higher UPDRS III (p=0.04) and lower EK-60F (p=0.025) scores were observed in the DEL_SCZ group. We found inverse correlations between UPDRS III and both TASIT EmRec (r=-0.289, p=0.031) and EK-60F (r=-0.387, p=0.006) scores in the whole sample. Correlations were no longer significant in the DEL_SCZ group (UPDRS III-TASIT EmRec p=0.629; UPDRS III-EK60F p=0.933) whilst being stronger in the DEL group (TASIT EmRec, r=-0.560, p<0.001; EK60F, r=-0.542, p<0.001). Analyses were adjusted for CPZ Eq and IQ. Conclusions. A modulation between FEE recognition deficits and motor symptoms and signs was observed in the 22q11.2DS group, likely affecting patients' quality of life.

Date 2023-03-28
Language English
Extra Place: 111 RIVER ST, HOBOKEN 07030-5774, NJ USA Type: Article
Volume 2023
Publisher WILEY
Publication ACTA NEUROLOGICA SCANDINAVICA
DOI 10.1155/2023/8546610
ISSN 0001-6314
Date Added 14.7.2025, 14:50:28
Modified 14.7.2025, 14:50:28

Notes:

Not Included: Not a Parkinson sample

Tags: EXCLUDED

[The Role of Brodmann Area 12: Taste, Social Cognition, and Mental Time].

**Item Type**	Journal Article
**Author**	Mitsuru Kawamura
**Abstract**	Broadman area 12, together with area 11, is located in the orbitofrontal area. A voxel-based morphometric (VBM) study revealed the association between bilateral brodmann areas 12/47 and taste disturbance in individuals with frontotemporal lobar degeneration (FTLD). In our VBM study in patients with Parkinson's disease, decision-making impairments were associated with atrophy of the bilateral Brodmann area 12, indicating that this area may play an important role in social cognitive function. Our recent study also demonstrated that this area may serve as time order judgement or mental time travel.
**Date**	2017 Apr
**Language**	jpn
**Extra**	Place: Japan
**Volume**	69
**Pages**	375-381
**Publication**	Brain and nerve = Shinkei kenkyu no shinpo
**DOI**	10.11477/mf.1416200754
**Issue**	4
**Journal Abbr**	Brain Nerve
**ISSN**	1881-6096
**PMID**	28424392
**Date Added**	6.7.2025, 19:09:42
**Modified**	5.9.2025, 14:41:14

Notes:

Not Included: Not in English

Tags: EXCLUDED

The Role of Social Cognition Abilities in Parkinson's Disease in the Era of COVID-19 Emergency.

**Item Type**	Journal Article
**Author**	Alessandra Dodich
**Author**	Costanza Papagno
**Author**	Luca Turella
**Author**	Claudia Meli

Author Francesca Zappini
Author Pamela Narduzzi
Author Alessandro Gober
Author Enrica Pierotti
Author Marika Falla
Abstract Introduction: Parkinson's Disease (PD) is characterized by motor and non-motor symptoms, among which deficits in social cognition might affect ~20% of patients. This study aims to evaluate the role of social cognitive abilities in the perceived impact of COVID-19 emergency, and the effects of lockdown measures on patients' social network and caregivers' burden. Methods: Fourteen PD patients performed a neuropsychological battery including sociocognitive tasks before the introduction of COVID-19 restrictive measures (i.e., social distancing and isolation). A structured interview through an online platform was performed in the last 2 weeks of the first lockdown phase to assess patients' health status, perception of COVID-19 emergency, changes in caregivers' burden, and patients' social isolation. Non-parametric analyses were performed to evaluate the association between social skills and patients' COVID-19 perception, as well as the effects of restrictive measures. Results: At baseline evaluation, half of the PD patients showed sociocognitive dysfunctions, mainly on mentalizing abilities. Patients with impaired social cognition skills showed a significantly lower concern on the possible effects of COVID-19 on their health. Caregiver burden and patients' social network remained stable during the lockdown. Conclusion: These preliminary results underline that PD sociocognitive dysfunctions might affect patients' abilities to estimate the effects of COVID-19 infection. However, the lack of a significant increase in caregivers' burden and social isolation suggests, in our sample, a good coping to COVID-19 emergency. Since COVID-19 pandemic can have direct and indirect severe consequences in patients with PD, the development of educational and preventive programs is recommended.
Date 2021
Language eng
License Copyright © 2021 Dodich, Papagno, Turella, Meli, Zappini, Narduzzi, Gober, Pierotti and Falla.
Extra Place: Switzerland
Volume 12
Pages 571991
Publication Frontiers in psychology
DOI 10.3389/fpsyg.2021.571991
Journal Abbr Front Psychol
ISSN 1664-1078
PMID 33859588
PMCID PMC8042207
Date Added 6.7.2025, 19:09:34
Modified 5.9.2025, 14:34:03

Notes:

Not Included: not on SC as need for this review

Tags: EXCLUDED

The role of the dorsal striatum in the recognition of emotions expressed by voice in Parkinson's disease.

**Item Type**	Journal Article
**Author**	Sonia Di Tella
**Author**	Isabella Anzuino
**Author**	Federica Biassoni
**Author**	Maria Rita Ciceri
**Author**	Martina Gnerre
**Author**	Raffaello Nemni
**Author**	Monia Cabinio
**Author**	Francesca Baglio
**Author**	Maria Caterina Silveri
**Abstract**	BACKGROUND AND PURPOSE: Non-motor impairment such as emotion recognition deficit in both facial and vocal expressions has been previously reported in Parkinson's disease (PD). We investigated whether the decoding of emotional prosody is impaired in PD and whether this deficit is related to striatal damage. METHODS: Fifteen PD patients and 15 healthy controls (HCs) were requested to listen to six audio tracks and to recognize the emotions expressed by a professional actor while reading a meaning-neutral sentence. All subjects also received a structural MRI examination. Volumetric measurements were extracted for the striatum, a key region involved in emotional processing and typically impaired in PD. RESULTS: Decoding sadness conveyed by voice was impaired in PD compared with HC and was related to the volume of the dorsal striatum bilaterally. CONCLUSIONS: The dorsal striatum is involved in the decoding of vocal negative emotions in PD.
**Date**	2021 May
**Language**	eng
**Extra**	Place: Italy
**Volume**	42
**Pages**	2085-2089
**Publication**	Neurological sciences : official journal of the Italian Neurological Society and of the Italian Society of Clinical Neurophysiology
**DOI**	10.1007/s10072-020-04959-5
**Issue**	5
**Journal Abbr**	Neurol Sci
**ISSN**	1590-3478 1590-1874
**PMID**	33411203
**Date Added**	6.7.2025, 19:09:38
**Modified**	5.9.2025, 14:33:23

Notes:

Included

sample characteristics

size: 15 PD and 15 HC

Parkinson's Disease type and duration: idiopathic PD, Mduration = 3.48 SD= 1.78

Medication: on medication

Hoehn-Yahr: M= 1.79 SD= 0.38

UPDRS-3: M= 27.80 SD= 10.60

Gender (male): 9 males (60%)

averaged ages (SD, range): M= 69.93 SD= 7.12

other neurological disease (tumor, stroke, etc.): None

other major psychopathology: None

origin country (or ethnicity): Italy

method observational and imaging

instruments used in order to quantify the variables

Social cognition aspect: emotion recognition

Name of the task: NA

type of stimulus [face/voice etc., Ekman faces/other etc.]: ekman faces

task condition: anger, happiness, fear, sadness, disgust, and neutral

operationalization: accuracy

Name of the task: NA

type of stimulus [face/voice etc., Ekman faces/other etc.]: Participants listened through headphones to the standard sentence "*Non è possibile, non ora*" ("It's not possible, not now") uttered by the same male actor in six different overtones

task condition: anger, happiness, fear, sadness, disgust, and neutral

operationalization: they had to point to the label of the emotion corresponding to the stimulus, which was randomly positioned on a list of eleven labels (i.e., the five emotions, the neutral item, and five additional distractors: compassion, doubt, irony, jealousy, and nostalgia).

One point was given for each correct recognition and 0 points for any false recognition. To account for possible response biases, the scores were transformed into unbiased Hu *scores* (Hu scores are calculated on the basis of both the number of correct uses (correct recognition) and incorrect uses of the label (false alarm). Hu scores range from 0 to 1.57 (perfect score)).

MRI: (1.5 T Siemens Magnetom Avanto) including (1) a 3D T1 MPRAGE scan (TR/TE = 1900/3.37 ms, FoV = 192 × 256 mm^2, voxel 1 mm^3, 176 axial slices) to obtain cortical and subcortical measurements and (2) conventional anatomical scans (proton density—T2, FLAIR) to exclude patients with gross brain changes and/or white matter hyperintensities outside the normal range. 3D T1 images were processed with Freesurfer's recon-all (https://surfer.nmr.mgh.harvard.edu/) and ENIGMA guidelines (https://enigma.ini.usc.edu/protocols/imaging-protocols) for quality check. Manual corrections were performed if needed.

Subcortical volumes were extracted for the total dorsal striatum (caudate + putamen, left + right) (Fig. 1(B)) and the total hippocampus (right + left) as control region. To account for different brain dimensions, total gray volume was also computed and used to normalize the subcortical volumes extracted (subcortical volume/total gray volume).

Main findings related to the review's scope

No sig difference in Ekman faces test between PD and HC

Hu scores: only on sadness, PD scored significantly lower than HC.

The point biserial correlation coefficient (r_{pb}) between the recognition of sadness and the volume of the bilateral striatum was significant in the PD group ($r_{pb} = 0.549$, $p = 0.034$) but not in the HC group ($r_{pb} = -0.034$; $p = 0.909$) (Fig. 1(C)).

Tags: Emotion recognition, Imaging, behavioral

The spectrum of embodied intersubjective synchrony in empathy: from fully embodied to externally oriented engagement in Parkinson's disease

**Item Type**	Journal Article
**Author**	Antonia Zepeda
**Author**	Alejandro Troncoso
**Author**	Daniela Pizarro
**Author**	Constanza Baquedano
**Author**	Rodrigo Gomez
**Author**	Silvia Barria
**Author**	Kevin Blanco
**Author**	David Martinez-Pernia
**Date**	2025-05-09
**Volume**	16
**Publication**	FRONTIERS IN PSYCHOLOGY
**DOI**	10.3389/fpsyg.2025.1570124
**ISSN**	1664-1078
**Date Added**	14.7.2025, 14:48:38

Modified 5.9.2025, 15:02:58

Notes:

Not Included: Only PD-group (no controls). Also mostly qualitative analysis

Tags: EXCLUDED

The Structure of Social Cognition: In(ter)dependence of Sociocognitive Processes

Item Type Journal Article

Author Francesca Happé
Author Jennifer L. Cook
Author Geoffrey Bird

Abstract Social cognition is a topic of enormous interest and much research, but we are far from having an agreed taxonomy or factor structure of relevant processes. The aim of this review is to outline briefly what is known about the structure of social cognition and to suggest how further progress can be made to delineate the in(ter)dependence of core sociocognitive processes. We focus in particular on several processes that have been discussed and tested together in typical and atypical (notably autism spectrum disorder) groups: imitation, biological motion, empathy, and theory of mind. We consider the domain specificity/generality of core processes in social learning, reward, and attention, and we highlight the potential relevance of dual-process theories that distinguish systems for fast/automatic and slow/effortful processing. We conclude with methodological and conceptual suggestions for future progress in uncovering the structure of social cognition.

Date 2017-01-03
Language en
Short Title The Structure of Social Cognition
Library Catalog DOI.org (Crossref)
URL https://www.annualreviews.org/doi/10.1146/annurev-psych-010416-044046
Accessed 19.1.2026, 7:10:46
Volume 68
Pages 243-267
Publication Annual Review of Psychology
DOI 10.1146/annurev-psych-010416-044046
Issue 1
Journal Abbr Annu. Rev. Psychol.
ISSN 0066-4308, 1545-2085
Date Added 19.1.2026, 7:10:46
Modified 19.1.2026, 7:10:46

THEORY OF MIND ABILITIES IN NEURODEGENERATIVE DISEASES: AN UPDATE AND A CALL TO INTRODUCE MENTALIZING TASKS IN STANDARD NEUROPSYCHOLOGICAL ASSESSMENTS

Item Type Journal Article
Author Mauro Adenzato
Author Michele Poletti
Abstract There is fast-growing interest in the study of Theory of Mind (ToM) abilities in neurodegenerative diseases. In a previous work, we reviewed all the evidence of altered ToM abilities in patients with neurodegenerative diseases in the literature published until then. In the present paper, we extend that analysis by integrating our conclusions with the most updated evidence that is now available. This new analysis allows for a clarification of some pending questions, such as at which stage ToM deficits begin to appear in dementing disorders, what is the relationship between executive functioning and ToM abilities in patients with Parkinson's disease, and how can ToM tasks help clinicians to discriminate between different neurodegenerative disorders. Furthermore, we now provide the first review of all articles on ToM abilities in patients with multiple sclerosis. The data discussed here strongly suggest overall that a neuropsychological assessment of patients with neurodegenerative diseases should routinely include an accurate investigation of ToM abilities. Increasing evidence has shown that different ToM tasks may help clinicians in the diagnostic process and caregivers in understanding the behavioural problems that are often shown by their suffering relatives.
Date 2013-10
Language English
Extra Place: VIA ARCHIMEDE 179, ROME, 00197, ITALY Type: Article
Volume 10
Publisher GIOVANNI FIORITI EDITORE
Pages 226-234
Publication CLINICAL NEUROPSYCHIATRY
Issue 5
ISSN 1724-4935
Date Added 14.7.2025, 14:50:38
Modified 5.9.2025, 14:24:56

Notes:

Not Included: not a systematic review
Tags: EXCLUDED

Theory of mind and decision-making processes are impaired in Parkinson's disease.

Item Type Journal Article
Author Chunhua Xi
Author Youling Zhu

Author Yanfang Mu
Author Bing Chen
Author Bin Dong
Author Huaidong Cheng
Author Panpan Hu
Author Chunyan Zhu
Author Kai Wang

Abstract Prefrontal cortex plays a vital role in the theory of mind (ToM) and decision making, as shown in functional brain imaging and lesion studies. Considering the primary neuropathology of Parkinson's disease (PD) involving the frontal lobe system, patients with PD are expected to exhibit deficits in ToM and social decision making. The aim of this study was to investigate affective ToM and decision making in patients with PD and healthy controls (HC) in a task assessing affective ToM (Reading the Mind in the Eyes, RME) and two decision-making tasks (Iowa Gambling Task, IGT; Game of Dice Task, GDT). Consistent with previous findings, patients with PD were impaired in the affective ToM task, and when making decisions under ambiguity and in risk situations. The score of emotion recognition in the RME task was negatively correlated with the severity of the disease and positively correlated with the total number of advantageous cards chosen in the IGT. However, the final capital in the GDT was correlated with memory impairment. The present study implies that affective ToM and decision making under ambiguity may share similar neural mechanisms, while decision making under ambiguity and decision making under risk may involve processing within different neural networks.

Date 2015 Feb 15
Language eng
License Copyright © 2014 Elsevier B.V. All rights reserved.
Extra Place: Netherlands
Volume 279
Pages 226-233
Publication Behavioural brain research
DOI 10.1016/j.bbr.2014.11.035
Journal Abbr Behav Brain Res
ISSN 1872-7549 0166-4328
PMID 25435317
Date Added 6.7.2025, 19:09:38
Modified 5.9.2025, 15:01:08

Notes:

Included

Sample characteristics

Size: 15 PD, 15 HC

PD-type: NA (early to moderatly affected)

PD-duration: M = 4.33, SD 0 5.05

Medication: All patients received typical dopaminergic medication (levodopa, dopamine-agonists)

Hoehn-Yahr: 1.97 ± 0.67

UPDRS-3: 15.87 ± 8.96

Gender (male): 7 (47%)

Age: 60.73 ± 11.79

Other neurological disease (tumor, stroke, etc.): None

Other major psychopathology: no MDD

Origin country (or ethnicity): China

method observational

instruments used in order to quantify the variables

Social cognition aspect: affective ToM

Name of the task: Reading the Mind in the Eyes task

Type of stimulus [face/voice etc., Ekman faces/other etc.]: 34 photographs of Asians exhibiting various facial expressions, showing only the eye region (blackand-white photographs

Task condition: For each eye region, participants were asked to choose the word from a list that could describe the complicated emotional expression (mind reading); one correct and three distractor words were presented for each item. There were no time limits. At the same time, participants were asked to judge the gender of each person in the photographs as a control task

Operationalization: Number of correct answers (The maximum score a participant could receive on the RME and gender recognition task was 34.)

Main findings related to the review's scope

Reading the Mind in the Eyes task

The PD group performed worse than the HC group in emotion recognition (mind reading) ($t(28) = 4.79$, $p < 0.001$).

Tags: ToM, behavioral

Theory of Mind and Empathy as Multidimensional Constructs: Neurological Foundations

Item Type Journal Article
Author Jonathan Dvash
Author Simone G. Shamay-Tsoory
Date 10/2014
Language en
Short Title Theory of Mind and Empathy as Multidimensional Constructs
Library Catalog DOI.org (Crossref)
URL https://journals.lww.com/00011363-201410000-00003
Accessed 19.1.2026, 7:30:00
Volume 34
Pages 282-295
Publication Topics in Language Disorders
DOI 10.1097/TLD.0000000000000040
Issue 4
ISSN 0271-8294
Date Added 19.1.2026, 7:30:00
Modified 19.1.2026, 7:30:00

Theory of mind and executive functions in normal human aging and Parkinson's disease.

Item Type Journal Article
Author J. Saltzman
Author E. Strauss
Author M. Hunter
Author S. Archibald
Abstract Although the majority of research in theory of mind (TOM) has focused on young children or individuals with autism, recent investigations have begun to look at TOM throughout the lifespan and in other neurological and psychiatric populations. Some have suggested that TOM may represent a dissociable, modular brain system that is related to, but separable, from other brain functions including executive functions (EF). Recently, studies have shown that TOM performance can be compromised following an acquired brain insult (e.g., damage to the right hemisphere). However, the relationship of such impaired TOM performance to other brain functions in these cases has not been explored. This study investigated the effects of both normal human aging and Parkinson's disease on TOM. The relationship of TOM performance and EF in these groups was also examined. The results suggested that although TOM performance appeared compromised in the group of individuals with Parkinson's disease, the elderly control participants were relatively unimpaired relative to younger individuals. Significant relationships between several measures of TOM and EF were also found. The implications of these findings, and also the finding that failure on one measure of TOM did not necessarily predict failure on all measures of TOM, are discussed.

**Date**	2000 Nov
**Language**	eng
**Extra**	Place: England
**Volume**	6
**Pages**	781-788
**Publication**	Journal of the International Neuropsychological Society : JINS
**DOI**	10.1017/s1355617700677056
**Issue**	7
**Journal Abbr**	J Int Neuropsychol Soc
**ISSN**	1355-6177
**PMID**	11105468
**Date Added**	6.7.2025, 19:09:41
**Modified**	5.9.2025, 14:54:53

Notes:

Included

Sample characteristics

Size: 11 PD, 8 HC (elderly), 9 HC (university-aged)

PD-type: Idiopathic PD

PD-duration: NA

Medication: All patients were being treated with Sinemet at the time of the study

Hoehn-Yahr: M = 2.5, Range = 2-3

UPDRS-3: NA

Gender (male): 6 (55%)

Age: M = 70.98, SD = 13.43, Range = 48-84.83

Other neurological disease (tumor, stroke, etc.): Persons with chronic medical problems (e.g., hypothyroidism, hypertension) were included as long as it was clear that their medical condition was well controlled, and not believed to be adversely affecting their level of cognition

Other major psychopathology: One participant with Parkinson's disease achieved a moderately elevated score on the GDS, and was being treated with antidepressant medication.

Origin country (or ethnicity): Canada

method behavioural

instruments used in order to quantify the variables

Social cognition aspect: ToM

Name of the task: NA

Type of stimulus [face/voice etc., Ekman faces/other etc.]: Participants heard two short false-belief stories: one first and one second-order attribution task

Task condition: Following each story, participants were asked three types of questions

1. Memory question
2. Predicition question (regarding behaviour of characters in the story)
3. Justification question (asked why they made the prediction

Operationalization: Participants received a score of zero or 1 (incorrect or correct) for each question.

Social cognition aspect: ToM

Name of the task: Droodles Task

Type of stimulus [face/voice etc., Ekman faces/other etc.]: participants were shown a cartoon-type picture that was taped inside an 8.5 3 11 cm filing folder

Task condition: For the first folder picture, participants were shown only the cut-out window portion of a picture, and asked to provide as many possible guesses as to the true picture content. For the remaining four folders pictures, participants were first shown and asked to describe the whole picture.

Then, the picture was covered so that only a small portion was visible through a cut-out window. Participants were asked to predict what another person (Bill, who has not seen the entire picture) would think that picture was. Then, participants were asked to predict what a second person (Roberta) would think the picture was, if she didn't think the same thing as Bill.

Operationalization: Participants received a score of zero or 1 (incorrect or correct) for their predictions of Bill and of Roberta, for a total score of 4 points for Bill's responses (first-order attribution), and 4 points for Roberta's responses (interpretation).

Social cognition aspect: ToM

Name of the task: Knower/Guesser ("egg cup") task

Type of stimulus [face/voice etc., Ekman faces/other etc.]: They were told that a paper clip would be hidden underneath one of the cups. While the clip was being hidden, a cardboard screen was placed in front of the cups, such that the participant could see that the clip was being hidden, but not where it was hidden. Participants were told that after the clip was hidden the screen would be removed. At this point, the examiner and her confederate (another graduate student) simultaneously pointed to the location where each thought the clip was hiding. The participants were instructed that the examiner and the confederate would point honestly and would not try to deceive them in any way. Participants were informed that, in order to make the task more difficult for the confederate, she would cover her eyes with a blindfold, while the clip was being hidden. The participants were then given eight trials in which they had to figure out the location of the clip. If at any point the participant verbally indicated that they understood that the clip was always in the location where the examiner was pointing, the procedure was terminated and the participant was given credit for the remaining trials. At the end of eight trials, participants were asked how they knew where the clip was hiding, or if they used any strategies for locating it.

Operationalization: Participants were scored on the number of correct guesses they made.

A summary score indicative of one's overall performance on the four measures of theory of mind, was assigned to each participant (TOM composite score). Participants received 1 point for each theory of mind task they successfully completed (maximum 5 4 points)

Main findings related to the review's scope

Overall, participants with Parkinson's disease scored significantly lower on the TOM composite score than their age-matched counterparts, suggesting that they had difficulty on a greater number of these tasks.

Specifically, the false-belief stories and "spy" model were areas of relative difficulty for the participants with Parkinson's disease; they were less able to make correct predictions based on inferences about a story character's belief, and they had more difficulty planning a course of action that could deceive another person.

In contrast, as compared to their age matched controls, participants with Parkinson's disease were equally able to recognize that one individual might hold a different interpretation than another (Droodles), and they were able to recognize that seeing can lead to knowing (Knower0Guesser).

Tags: ToM, behavioral

Theory of mind and joint action in Parkinson's disease.

Item Type Journal Article
Author Marco Fabbri
Author Carmine Vitale
Author Sofia Cuoco
Author Alessia Beracci
Author Rosanna Calabrese
Author Maria Cordella
Author Regina Mazzotta
Author Paolo Barone
Author Maria Teresa Pellecchia
Author Gabriella Santangelo
Abstract It has been suggested that the Theory of Mind (ToM) may rely on more basic processes of social cognition, such as action control (e.g., joint action), even if little is known about this relationship. The relationship between ToM and joint action can be studied in patients with Parkinson's disease (PD), because they are characterized not only by a deficit in ToM (and in its cognitive and affective subcomponents) but also by a deficit in the inhibition of competing responses. Sixty PD patients and 60 matched healthy controls (HCs) performed a go/no-go Flanker task in both joint and individual conditions. Cognitive (Advanced Test or AT) and affective (Emotion Attribution Task or EAT) ToM also were measured. Thirty-five PD patients and matched HCs also performed the standard Flanker task, as a control measure. In patients, only individuals with high AT scores exhibited a joint Flanker effect, whereas in HCs the joint effect was found irrespectively of AT score. Patients with low EAT scores showed a greater interference effect than patients with high scores, whereas the opposite pattern was found for HCs. In regression analysis AT and EAT scores predicted the Flanker effect in the joint condition only. In the standard task, both groups showed a Flanker effect. The role of different fronto-striatal circuits, especially in PD patients, could explain the different involvement of cognitive and affective ToM in joint tasks. The Flanker effect is discussed considering the referential coding account and the attention-focus account as possible candidates to explain joint action effects.
Date 2018 Dec
Language eng
Extra Place: United States
Volume 18
Pages 1320-1337
Publication Cognitive, affective & behavioral neuroscience
DOI 10.3758/s13415-018-0642-0
Issue 6
Journal Abbr Cogn Affect Behav Neurosci
ISSN 1531-135X 1530-7026
PMID 30259349
Date Added 6.7.2025, 19:09:40
Modified 5.9.2025, 14:35:27

Notes:

Not Included: did not report on relevant results
Tags: EXCLUDED

Theory of mind deficits in Parkinson's disease are not modulated by dopaminergic medication.

Item Type Journal Article
Author Tatiana Usnich
Author Elena Krasivskaya
Author Fabian Klostermann
Abstract INTRODUCTION: Patients with Parkinson's disease (PD) exhibit deficits in social cognition, particularly with respect to Theory of Mind (ToM) capacities. It is unclear whether they are associated with PD-related dopamine deficiency and modulated by levodopa replacement therapy. METHODS: A total of 15 persons with PD and 13 healthy controls (HC) participated in the study. They performed different neuropsychological tasks, including the Faux Pas Recognition Test (FPRT), assessing different dimensions of cognitive ToM (e.g., detection, inappropriateness, intentions), and the Reading the Mind in the Eyes Test (RMET) as an index of affective ToM. Persons with PD were tested twice, once under their regular treatment and another time after at least 18 h of levodopa withdrawal (MED-ON and MED-OFF, respectively). On either occasion, serum drug levels and motor symptom severity [Unified Parkinson's Disease Rating Scale (UPDRS)] were measured. RESULTS: MED-ON and MED-OFF conditions in patients with PD were confirmed by higher serum drug levels in the former than in the latter state and a corresponding amelioration of the motor deficit. In so doing, no performance difference in any ToM-related task was identified as a function of the levodopa therapy. Generally, patients performed worse than controls in both affective and cognitive ToM tests. CONCLUSION: Patients with PD have deficits in cognitive and affective ToM. Dopamine replacement, effective for improving the motor condition, does not appear to counteract these dysfunctions.
Date 2023
Language eng
License Copyright © 2023 Usnich, Krasivskaya and Klostermann.
Extra Place: Switzerland
Volume 14
Pages 1208638
Publication Frontiers in neurology
DOI 10.3389/fneur.2023.1208638
Journal Abbr Front Neurol
ISSN 1664-2295
PMID 37822526
PMCID PMC10562626
Date Added 6.7.2025, 19:09:40
Modified 5.9.2025, 14:59:15

Notes:

Included

Sample characteristics

Size: 15 PD, 13 HC

PD-type: NA

PD-duration: 4.13 (± 3.83)

Medication: measured ON and OFF

Hoehn-Yahr: Median = 2.0 (1.0–4.0)

UPDRS-3: ON: 22.40 (±5.539); OFF: 32.27 (±9.505)

Gender (male): 10 (66.7%)

Age: 75.33 (± 8.98)

Other neurological disease (tumor, stroke, etc.): None

Other major psychopathology: None

Origin country (or ethnicity): Germany

method observational

instruments used in order to quantify the variables

Social cognition aspect: affective ToM

Name of the task: Reading the Mind in the Eyes Task (RMET)

Type of stimulus [face/voice etc., Ekman faces/other etc.]: photos of the periocular eye region of different persons (one photo per person).

Task condition: The decision is made from four predefined options (e.g., angry, sad, friendly, and flirty). 2x18 picture for ON and OFF test

Operationalization: Correct answers

Social cognition aspect: ToM

Name of the task: Faux Pas Recognition Test (FPRT)

Type of stimulus [face/voice etc., Ekman faces/other etc.]: 20 short stories in which persons communicate with each other

Task condition: Per story, one has to answer (i) whether the misconduct was present (Faux Pas Detection), and, if this was the case, (ii) why it is inappropriate (Faux Pas Inappropriateness), (iii) which goal it pursued (Faux Pas Intention), (iv) whether it was formulated accidentally or on purpose (Faux Pas Belief), and (v) which emotions it triggered in the interlocutors (Empathy). 10 stories per MED-ON and MED-OFF, respectively (containing 5 stories with and without a faux pas each)

Operationalization: Correct answers. ; scores are expressed as the percentage ratio of reached to maximally possible points

Main findings related to the review's scope

FPRT:

The faux pas detection rate was lower in patients under MED-OFF than in controls.

This statistical group difference vanished when patients were in the MED-ON condition.

Concerning all other cognitive theory of mind dimensions (understanding inappropriateness, intentions, belief, and empathy), patients, be they under MED-ON or MED-OFF, performed worse than controls.

Within patients, no performance differences were identified between the MED-ON and MED-OFF conditions

RMET

results were lower in the patients under MED-ON than in controls [t(df)=2.453 (26); p=0.021]. However, under MED-OFF, this group difference failed to be significant, with the difference between MED-ON and MED-OFF performances being marginal

Tags: ToM, behavioral

Theory of mind deficits in Parkinson's disease: a product of executive dysfunction?

Item Type Journal Article

Author Clare M. Eddy
Author Sarah R. Beck
Author Ian J. Mitchell
Author Peter Praamstra
Author Hardev S. Pall

Abstract OBJECTIVE: Patients with Parkinson's disease (PD) can perform poorly on tasks involving theory of mind (ToM): the ability to reason about mental states. We investigated whether patients' ToM deficits were independent of executive dysfunction. METHOD: Experiment 1 aimed to establish that ToM deficits were present, and 2 following experiments manipulated the working memory (WM) demands of the ToM task. RESULTS: In Experiment 1, 15 patients with PD performed significantly more poorly than controls on a false belief vignette task but not on a faux pas task. Errors were related to poor verbal fluency. In Experiment 2, 24 patients with PD made fewer errors on shorter false belief vignettes than the original FBT, and errors on the latter were related to WM impairment. In Experiment 3, the FBT was presented as a comic strip visible throughout questioning, reducing WM demands. Patients (n = 24) made memory errors but no false belief errors on the comic strip. They exhibited no verbal fluency or WM impairments, but did exhibit deficits on a black-and-white Stroop task. False belief errors were not correlated with executive performance. CONCLUSIONS: PD patients made very few ToM errors that were independent of errors on memory questions, so in this sample, ToM deficits per se appear unlikely. However, patients still made errors on ToM tasks when associated incidental WM demands were considerably reduced, highlighting the need for future investigations of ToM in PD to account for the role of more general cognitive restrictions exhibited by even some medicated, early stage patients.

**Date**	2013 Jan
**Language**	eng
**Extra**	Place: United States
**Volume**	27
**Pages**	37-47
**Publication**	Neuropsychology
**DOI**	10.1037/a0031302
**Issue**	1
**Journal Abbr**	Neuropsychology
**ISSN**	1931-1559 0894-4105
**PMID**	23356595
**Date Added**	6.7.2025, 19:09:41
**Modified**	5.9.2025, 14:34:47

Notes:

Included

EXP1

sample characteristics

size: 15 PD and 10 HC

Parkinson's Disease type and duration: Mduration = 9.53 years (SD=5.85, range 2–19 years).

Medication: on medication

Hoehn-Yahr: NA

UPDRS-3: NA

Gender (male): 10 males (67%)

averaged ages (SD, range): M= 65.6 years (SD=9.60)

other neurological disease (tumor, stroke, etc.): no dementia

other major psychopathology: NA

origin country (or ethnicity): United Kingdom

method (Review, meta-analysis or observational and/or self-reported):

instruments used in order to quantify the variables

Social cognition aspect: ToM

Name of the task: Faux pas test.

type of stimulus [face/voice etc., Ekman faces/other etc.]: Participants were read eight vignettes. Four test vignettes described a potentially offensive faux pas. For example, Jill has moved house and bought new curtains, and Lisa says the curtains are horrible. Four control stories involved no faux pas.

operationalization: accuracy

Name of the task: False belief vignette test.

type of stimulus [face/voice etc., Ekman faces/other etc.]: Participants were read four unexpected transfer style vignettes. Example: “Andrew is in bed. Susie goes to the shops and while she’s away, Andrew goes to the school.” Each vignette was read twice and followed by four questions: Counterfactual, “Where would Andrew be if he hadn’t gone to the school?” False belief, “Where does Susie think Andrew is?” Memory, “Where was Andrew at the start?” Reality, “Where is Andrew now?” There were two forced-choice responses, the target’s original and current locations. Vignettes were presented in a set order, but questions were counterbalanced in pairs (first and second false belief or counterfactual; third and fourth memory or reality).

operationalization: accuracy

-

Main findings related to the review's scope

Patients did not perform significantly differently from controls when asked to recognize faux pas.

Patients made an average of 1.46 errors on the false belief vignette test and performed significantly more poorly than controls (U 56.0, p .001), who performed at ceiling.

EXP2

sample characteristics

size: 24 PD and 28 HC

Parkinson's Disease type and duration: Mduration = 8.02 years (SD 4.94, range 2–20 years).

Medication: on medication

Hoehn-Yahr: NA

UPDRS-3: NA

Gender (male): 18 males (75%)

averaged ages (SD, range): M= 66 years (SD=8.99, range 49–86 years)

other neurological disease (tumor, stroke, etc.): no dementia

other major psychopathology: NA

origin country (or ethnicity): United Kingdom

method (Review, meta-analysis or observational and/or self-reported):

instruments used in order to quantify the variables

Social cognition aspect: ToM

Name of the task: ToM vignettes.

type of stimulus [face/voice etc., Ekman faces/other etc.]:

Short, noninferential "deceptive box" style vignettes. Each deceptive box vignette described one character telling another character a lie to deceive them about the identity of an item. Example: "It is Arthur and Beryl's anniversary. Beryl tells Arthur that she has bought him a CD. In fact, she has bought him tickets to a concert. Arthur doesn't know that Beryl is going to surprise him, so he believes Beryl." These vignettes were followed by false belief (e.g., "What does Arthur think Beryl has bought him for his birthday?") and reality ("What has Beryl really bought for Arthur?") questions, with two forced choice options.

Short, "unexpected transfer" vignettes. The unexpected transfer vignettes had a similar structure to those in the false belief vignette test but were shorter. Example: "Judy leaves some cigarettes on the coffee table. While she is in bed asleep, her son takes the cigarettes and puts them in his schoolbag." Participants were asked false belief (e.g., "Where does Judy think the cigarettes are?") and reality (e.g., "Where are the cigarettes really?") ques tions, and two forced-choice options were given.

False belief vignette task. The false belief vignette task was the same as that in Experiment 1.

operationalization: accuracy

-

Main findings related to the review's scope

ONLY ON PD (NO HC)

significantly more errors were made on the false belief vignette test than on the short unexpected transfer vignettes, z 3.42, p .001, and deceptive box vignettes, z 2.16, p .031.

AND IN EXP3 – no sig results

Tags: ToM, behavioral

Theory of mind in mild cognitive impairment and Parkinson's disease: The role of memory impairment.

**Item Type**	Journal Article
**Author**	Gianpaolo Maggi
**Author**	Chiara Giacobbe
**Author**	Carmine Vitale
**Author**	Marianna Amboni
**Author**	Ignacio Obeso
**Author**	Gabriella Santangelo
**Abstract**	BACKGROUND: Social cognition is impaired in Parkinson's disease (PD). Whether social cognitive impairment (iSC) is a by-product of the underlying cognitive deficits in PD or a process independent of cognitive status is unknown. To this end, the present study was designed to investigate the weight of specific cognitive deficits in social cognition, considering different mild cognitive impairment subtypes of PD (PD-MCI). METHODS: Fifty-eight PD patients underwent a neuropsychological battery assessing executive functions, memory, language, and visuospatial domains, together with social cognitive tests focused on theory of mind (ToM). Patients were divided into subgroups according to their clinical cognitive status: amnestic PD-MCI (PD-aMCI, n = 18), non-amnestic PD-MCI (PD-naMCI, n = 16), and cognitively unimpaired (PD-CU, n = 24). Composite scores for cognitive and social domains were computed to perform mediation analyses. RESULTS: Memory and language impairments mediated the effect of executive functioning in social cognitive deficits in PD patients. Dividing by MCI subgroups, iSC occurred more frequently in PD-aMCI (77.8%) than in PD-naMCI (18.8%) and PD-CU (8.3%). Moreover, PD-aMCI performed worse than PD-CU in all social cognitive measures, whereas PD-naMCI performed worse than PD-CU in only one subtype of the affective and cognitive ToM tests. CONCLUSIONS: Our findings suggest that ToM impairment in PD can be explained by memory dysfunction that mediates executive control. ToM downsides in the amnesic forms of PD-MCI may suggest that subtle changes in social cognition could partly explain future transitions into dementia. Hence, the evaluation of social cognition in PD is critical to characterize a possible behavioral marker of cognitive

decline.

**Date**	2024 Feb
**Language**	eng
**License**	© 2023. The Author(s).
**Extra**	Place: United States
**Volume**	24
**Pages**	156-170
**Publication**	Cognitive, affective & behavioral neuroscience
**DOI**	10.3758/s13415-023-01142-z
**Issue**	1
**Journal Abbr**	Cogn Affect Behav Neurosci
**ISSN**	1531-135X 1530-7026
**PMID**	38049608
**PMCID**	PMC10827829
**Date Added**	6.7.2025, 19:09:40
**Modified**	5.9.2025, 14:44:38

Notes:

Included

sample characteristics

size: 58 PD, divided into PD-CU= Parkinson's Disease, cognitively unimpaired (n=24); PD-aMCI= PD patients with amnestic MCI (n=18); PD-naMCI= PD patients with non amnestic MCI (n=16)

Parkinson's Disease type and duration: idiopathic PD; Mduration = 10.23 SD = 6.07

Medication: NA

Hoehn-Yahr: M = 2.34 SD = 0.66

UPDRS-3: M=15.49 SD = 8.33

Gender (male): 38 males (66%)

averaged ages (SD, range): M = 64.57, SD = 8.68

other neurological disease (tumor, stroke, etc.): None

other major psychopathology: None

origin country (or ethnicity): Italy

method observational

instruments used in order to quantify the variables

Social cognition aspect: ToM

Name of the task: The Reading the Mind in the Eyes Test (RMET - Affective ToM) **and** the Emotion Attribution Task (EAT - Affective ToM) **and** the Strange Stories (ATT - cognitive ToM) **and** the Theory of Mind Picture Stories Task (TMPS - cognitive ToM)

type of stimulus [face/voice etc., Ekman faces/other etc.]:

RMET - eye region of adult faces

EAT - short stories eliciting the attribution of several emotions

ATT - short stories depicting naturalistic situations in which two or more characters interact in familiar or social contexts

TMPS - series of six cartoon picture stories, different scenarios are presented that show reciprocity, deception, and cheating.

operationalization: patients reporting a composite score in social cognition tests <1.5 SD of the group average were classified as presenting social cognitive impairment (PD-iSC). Mean while, patients reporting a score >1.5 SD were classified as unimpaired social cognitive impairment (PD-uSC).

Main findings related to the review's scope

Thirty-nine PD patients did not present social cognitive impairment and were classified as PD-uSC, whereas 19 patients were assigned to PD-iSC group.

significant relationship between the occurrence of social cognitive impairment within the three groups: more social impairments in the PD-aMCI group

RMET: PD-aMCI had poorer performance, compared to HC. no other sig dif.

EAT: both PD-aMCI and PD-naMCI scored lower than PD-CU

ATT:PD-aMCI had poorer performance, compared to HC. no other sig dif.

TMPS: PD-aMCI and PD naMCI groups reported lower scores than PD-CU

Tags: ToM, behavioral

Theory of Mind in multiple system atrophy: comparison with Parkinson's disease and healthy subjects.

**Item Type**	Journal Article
**Author**	Gabriella Santangelo
**Author**	Sofia Cuoco
**Author**	Marina Picillo
**Author**	Roberto Erro
**Author**	Massimo Squillante
**Author**	Giampiero Volpe
**Author**	Autilia Cozzolino
**Author**	Giulio Cicarelli
**Author**	Paolo Barone
**Author**	Maria Teresa Pellecchia
**Abstract**	Theory of Mind is defined as the ability to attribute mental state and emotions to other people and is relevant to social relationships. The cortical and subcortical regions involved in Theory of Mind are damaged by neurodegenerative processes of Parkinsonian syndromes, so the aim of the present study was to explore, for the first time, possible deficits of Theory of Mind and their cognitive correlates in multiple system atrophy (MSA). Twenty-six patients with MSA, 25 patients with Parkinson's disease (PD) and 25 healthy subjects were enrolled. Cognitive and affective subcomponents of Theory of Mind, executive functions, long-term memory and apathy were evaluated. The three groups did not differ on demographic variables. MSA and PD groups performed worse than healthy subjects on both cognitive (advanced test of ToM) and affective (emotion attribution task) ToM tasks, but no significant difference was found between patients' groups. However, when using another affective ToM task (Eyes Test), MSA group had poorer performance than healthy subjects and Parkinsonian patients, whereas Parkinsonian patients had similar performance to healthy subjects. Regression analysis revealed an association between poor cognitive flexibility and dysfunctional cognitive component of Theory of Mind. Deficit of cognitive and affective components of Theory of Mind occurred in MSA. Dysfunction of cognitive component was related to executive dysfunction (i.e. cognitive rigidity). These findings might suggest the usefulness of an early evaluation of social cognition in MSA to identify individuals with impaired Theory of Mind who are at risk of social withdrawal, and reduced quality of life.
**Date**	2020 Jun
**Language**	eng

Extra Place: Austria
Volume 127
Pages 915-923
Publication Journal of neural transmission (Vienna, Austria : 1996)
DOI 10.1007/s00702-020-02181-3
Issue 6
Journal Abbr J Neural Transm (Vienna)
ISSN 1435-1463 0300-9564
PMID 32248368
Date Added 6.7.2025, 19:09:40
Modified 5.9.2025, 14:55:09

Notes:

Included

Sample characteristics

Size: 25 PD, 25 HC, 25 MSA (multi system atrophy).All groups matched for age, education, sex.

PD-type: Idiopathic PD

PD-duration: M = 7.6, SD = 5.9

Medication: LEDD mentioned

Hoehn-Yahr: NA

UPDRS-3: M = 18.5, SD = 9.2

Gender (male): NA

Age: M = 64.2, SD = 7.8

Other neurological disease (tumor, stroke, etc.): NA

Other major psychopathology: MoCA had to be > 15.5

Origin country (or ethnicity): Italy

method (Review, meta-analysis or observational and/or self-reported):

instruments used in order to quantify the variables

Social cognition aspect: ToM (cognitive subcomponent)

Name of the task: Advanced Test of ToM (AT)

Type of stimulus [face/voice etc., Ekman faces/other etc.]: 13 stories describing naturalistic situations where the characters interacted with each other.

Task condition: The subject had to explain why the characters behaved as they did

Operationalization: ; the total score ranges from 0 (worst performance) to 13 (best performance)

Social cognition aspect: ToM (affective subcomponent)

Name of the task: Emotion Attribution Task (EAT)

Type of stimulus [face/voice etc., Ekman faces/other etc.]: 35 short stories describing emotional situations (e.g., an employee apprehending to receive an extra salary)

Task condition: the subject has to explain what the main protagonists might feel in that situation.

Moreover, five stories elicit attribution of sadness, five of fear, five of embarrassment, five of disgust, five of happiness, five of anger, and five of envy.

Operationalization: ; The total score ranges from 0 (worst performance) to 35 (best performance).

The partial scores for each emotion range from 0 (worst performance) to 5 (best performance)

Social cognition aspect: ToM

Name of the task: The “Reading the Mind in the Eyes” Test (Eyes Test)

Type of stimulus [face/voice etc., Ekman faces/other etc.]: 36 still pictures of the eye regions illustrating emotionally charged

Task condition: the subject has to match the semantic definition of a mental state (e.g., “worried”, “annoyed”) to the picture of the eyeregion expression displayed in the screen

Moreover, five stories elicit attribution of sadness, five of fear, five of embarrassment, five of disgust, five of happiness, five of anger, and five of envy.

Operationalization: ; The total score ranges from 0 (worst performance) to 35 (best performance).

The partial scores for each emotion range from 0 (worst performance) to 5 (best performance)

Main findings related to the review's scope

EAT

PD poorer performance than HC: PD versus HC: U test = 141.5, p = 0.001)

AT

PD poorer performance than HC: PD versus HC: U test = 115.5, p < 0.001)

Eyes Test

PD patients had similar performance to the HCs (U test=225.5, p=0.090)
Tags: ToM, behavioral

Theory of Mind in normal ageing and neurodegenerative pathologies.

Item Type Journal Article
Author Jennifer Kemp
Author Olivier Després
Author François Sellal
Author André Dufour
Abstract This paper reviews findings in three subcomponents of social cognition (i.e., Theory of Mind, facial emotion recognition, empathy) during ageing. Changes over time in social cognition were evaluated in normal ageing and in patients with various neurodegenerative pathologies, such as Alzheimer's disease, mild cognitive impairment, frontal and temporal variants of frontotemporal lobar degeneration and Parkinson's disease. Findings suggest a decline in social cognition with normal ageing, a decline that is at least partially independent of a more general cognitive or executive decline. The investigation of neurodegenerative pathologies showing specific deficits in Theory of Mind in relation to damage to specific cerebral regions led us to suggest a neural network involved in Theory of Mind processes, namely a fronto-subcortical loop linking the basal ganglia to the regions of the frontal lobes.
Date 2012 Apr
Language eng

License Copyright Â© 2011 Elsevier B.V. All rights reserved.
Extra Place: England
Volume 11
Pages 199-219
Publication Ageing research reviews
DOI 10.1016/j.arr.2011.12.001
Issue 2
Journal Abbr Ageing Res Rev
ISSN 1872-9649 1568-1637
PMID 22186031
Date Added 6.7.2025, 19:09:39
Modified 5.9.2025, 14:41:44

Notes:

Not Included: Not a systematic review

Tags: EXCLUDED

Theory of mind in Parkinson's disease and related basal ganglia disorders: a systematic review.

Item Type Journal Article
Author Maren Elisabeth Bodden
Author Richard Dodel
Author Elke Kalbe
Abstract Theory of mind (ToM), defined as the ability to infer other people's mental states, is a crucial prerequisite of human social interaction and a major topic of interest in the recent neuroscientific research. It has been proposed that ToM is mediated by a complex neuroanatomical network that includes the medial prefrontal cortex, the anterior gyrus cinguli, the sulcus temporalis superior, the temporal poles, and the amygdala. Various neurological and psychiatric diseases are accompanied by aspects of dysfunctional ToM processing. In this review, the association between basal ganglia, involved in the organization of complex cognitive and emotional behavior, and ToM processing is discussed. The purpose of this review is to provide an overview of research on ToM abilities in basal ganglia disorders, especially Parkinson's Disease and related disorders.
Date 2010 Jan 15
Language eng
Extra Place: United States
Volume 25
Pages 13-27
Publication Movement disorders : official journal of the Movement Disorder Society
DOI 10.1002/mds.22818
Issue 1
Journal Abbr Mov Disord

ISSN 1531-8257 0885-3185
PMID 19908307
Date Added 6.7.2025, 19:09:40
Modified 5.9.2025, 14:29:08

Notes:

Included - systematic review

sample characteristics

In this review, we compassed the most prevalent basal ganglia disorders including Parkinson's disease, atypical Parkinson syndromes (multiple system atrophy, progressive supranuclear palsy, corticobasal ganglionic degeneration, Lewy body disease), restless legs syndrome and Huntington's disease. To identify studies that examine ToM functions in those disorders, we performed a systematic literature search in the databases Pubmed, Psyndex, and Psychinfo using a search strategy including the keywords "Theory of Mind," "social cognition," "empathy," "mindreading," "mentalizing," or "perspective taking" in combination with "Parkinson," "multiple system atrophy" or "MSA," "progressive supranuclear palsy" or "PSP," "corticobasal ganglionic degeneration" or "CBD," "Lewy body," "restless legs" or "RLS" or "Huntington." We also checked the reference lists of each relevant study that resulted from this search for further appropriate articles. There was no restriction on year of publication or language of the study. Articles published through March 2009 were included. 16 articles were identified as relevant.

Main findings related to the review's scope

ToM is impaired in PD were reported in studies using non-verbal card-sequencing tasks, false belief and first and second order ToM short stories,**41** faux pas recognition stories,**12** the "Reading the Mind in the Eyes Test" (RMET)**53**, **37** and visual material

ToM dysfunctions are related to specific pathologies that differ between PD subtypes

Tags: ToM, behavioral

Theory of mind in Parkinson's disease: A meta-analysis.

Item Type Journal Article

Author Emre Bora
Author Mark Walterfang
Author Dennis Velakoulis

Abstract Non-motor symptoms of Parkinson's disease (PD) include cognitive deficits and impairment in the recognition of emotions. Recently, a number of studies have investigated theory of mind (ToM) deficits in PD. In this meta-analysis of 18 studies, the ToM performance of 487 non-demented individuals with Parkinson's disease (PD) was compared with 459 healthy controls. Meta-regression analyses were conducted to investigate the relationship between ToM deficits and demographical and clinical features in PD, including medication, stage of the illness and neurocognition. PD was associated with significant ToM impairment (d = 0.83) in both verbal and visual tasks. In individual task analyses, PD patients performed worse than controls in both

faux pas recognition (d = 0.55) and reading the mind from the eyes (d = 0.67) tests. Severity of ToM impairment was significantly associated with cognitive impairment. Impairments in social cognitive abilities, including ToM, may be important contributors to the functional impairments observed in PD.

Date 2015 Oct 1
Language eng
License Copyright © 2015 Elsevier B.V. All rights reserved.
Extra Place: Netherlands
Volume 292
Pages 515-520
Publication Behavioural brain research
DOI 10.1016/j.bbr.2015.07.012
Journal Abbr Behav Brain Res
ISSN 1872-7549 0166-4328
PMID 26166188
Date Added 6.7.2025, 19:09:38
Modified 5.9.2025, 14:29:20

Notes:

Included - meta-analysis

sample characteristics

A literature search was conducted using the databases Pubmed, PsycINFO and Scopus to identify the relevant studies (January 1990–February 2015) using the combination of keywords as follows: Parkinson, [redacted], [redacted] and [redacted]. Reference lists of published reports were also reviewed for additional studies. Inclusion criteria were studies that: (1) Examined ToM abilities; (2) reported sufficient data to calculate the effect size and standard error of the ToM measure including results of parametric statistics (i.e., *t* and *F* values); (3) compared the neurocognitive performances of patients with non-demented PD and healthy controls.

A total of 18 studies (23 patient-control comparisons) consisting of 487 patient with PD (53.0% males) and 459 healthy controls (41.8% males) patients were included in the meta-analysis

PD patients included in studies had mild to moderate severity of the disease. There was no significant difference in age between PD patients and controls (d = 0.12, CI = −0.02 to 026, Z = 1.68, p = 0.09). In the PD group, the mean duration of disease was 5.84 years; mean Hoehn and Yahr (H–Y) rating scale score was 2.13 the mean mini mental state examination (MMSE) score was 28.1. 466 out of 487 PD patients were on levadopa and [redacted] and the mean levadopa equivalent daily dose (LEDD) was 652. PD patients included in studies had mild (mean H–Y = 1–2) to moderate (mean H–Y = 2–3) severity of the disease.

Main findings related to the review's scope

Highlights

•Parkinson's disease is associated with significant ToM impairment (d = 0.83).

•ToM impairment is evident in both affective and cognitive tasks.

•ToM impairment is associated with executive dysfunction.

there was consistent evidence of ToM impairment (hypothesis 1) which was more severe in later stages of the illness (hypothesis 3). Our second hypothesis was only partly supported as PD was associated with more severe deficits in cognitive tasks in comparison to affective tasks but there was no evidence for more severe impairment in visual tasks. Our findings suggest that ToM is significantly impaired in PD both for verbal and visual tasks including RMET and faux pas recognition. The magnitude of the main effect of diagnosis on ToM was large (d = 0.83). ToM impairment was correlated with executive functioning as measured by verbal fluency.

Tags: ToM, behavioral

Theory of mind in Parkinson's disease: evidences in drug-naïve patients and longitudinal effects of dopaminergic therapy.

Item Type Journal Article
Author Eleonora Del Prete
Author Pierpaolo Turcano

Author Elisa Unti
Author Giovanni Palermo
Author Cristina Pagni
Author Daniela Frosini
Author Ubaldo Bonuccelli
Author Roberto Ceravolo
Abstract Theory of mind (ToM) is the ability to attribute mental states to one self and others and to understand that others have beliefs different from one's own. Different subcomponents of ToM have also been identified: cognitive and affective. Cognitive ToM refers to the capacity to infer others' beliefs and intentions, while affective ToM implies the ability to appreciate others' emotional states. The aim of this study was to explore ToM in drug-naïve Parkinson's disease (PD) patients and to investigate the effects of chronic dopaminergic therapy on different subcomponents of ToM during a 3 months and 1 year of follow-up. We examined 16 PD patients in three conditions: before (un-medicated) and after dopaminergic therapy (medicated 3 months: T1 and medicated 1 year: T2). We also compared our PD's ToM abilities with 11 healthy individuals. ToM was explored with 5 different tasks: Faux Pas Test, Picture Sequencing Task Capture Story, Emotion Attribution Task, Strange Stories Task, and Karolinska Directed Emotional Faces. Our study confirms that PD patients present deficits in cognitive components of ToM and preserved performances in the affective ones in early stages of disease. We also find a significant effect of dopaminergic therapy on ToM already after 3 months with a good persistency after 1 year of treatment.
Date 2020 Oct
Language eng
Extra Place: Italy
Volume 41
Pages 2761-2766
Publication Neurological sciences : official journal of the Italian Neurological Society and of the Italian Society of Clinical Neurophysiology
DOI 10.1007/s10072-020-04374-w
Issue 10
Journal Abbr Neurol Sci
ISSN 1590-3478 1590-1874
PMID 32277390
Date Added 6.7.2025, 19:09:40
Modified 5.9.2025, 14:32:48

Notes:

Included

sample characteristics

size: 16 PD and 11 HC

Parkinson's Disease type and duration: drug-naïve PD, Mduration = 8 SD=4

Medication: off medication (T0) and on medication (T1/T2)

Hoehn-Yahr: M=1.4 SD=0.5 (1-2)

UPDRS-3: NA

Gender (male): NA

averaged ages (SD, range): M=66.2 SD=10.8

other neurological disease (tumor, stroke, etc.): no dementia

other major psychopathology: NA

origin country (or ethnicity): Italy

method observational

instruments used in order to quantify the variables

Social cognition aspect: ToM

Name of the task: Faux Pas Test ("cognitive" and "affective" ToM task)

type of stimulus [face/voice etc., Ekman faces/other etc.]: stories that may or may not contain a faux pas. In the stories containing a faux pas, the character committing the faux pas was unaware that he/she had said something inappropriate, whereas the person in the story hearing it might have felt hurt or insulted. We used a shortened version of the test, comprising 5 stories with a faux pas and 5 control scenarios without a faux pas

operationalization: scoring system as already used in Peron's manuscriptName of the task: Picture Sequencing Task Capture Story [13] ("cognitive" ToM task)

type of stimulus [face/voice etc., Ekman faces/other etc.]: four pictures describing a story. The pictures were randomly placed on a table and the subjects were asked to rearrange them to depict a logical and chronological order of events.

operationalization: One point was given after choosing cards' correct order; an extra point was also given for providing the correct answer to each of the questions.

task: Emotion Attribution Task ("affective" ToM task)

type of stimulus [face/voice etc., Ekman faces/other etc.]: four pictures describing a story. The pictures were randomly placed on a table and the subjects were asked to rearrange them to depict a logical and chronological order of events.

Condition: 3 of fear, 3 of disgust, 3 of happiness, and 3 of anger

operationalization: The total score ranges from 0 (worst performance) to 15 (best performance).

task: Strange Stories Task ("cognitive" ToM task)

type of stimulus [face/voice etc., Ekman faces/other etc.]: 13 stories describing naturalistic situations in which 2 or more characters interacted with each other as in familiar or social contexts. The subject was asked to explain why a character says something that is not literally true

operationalization: the total score ranges from 0 (worst performance) to 13 (best performance).

Main findings related to the review's scope

Faux Pas Test: PD patients performed worse than HC both explanation score and correct hits score.

Strange Stories Task: no significant differences were found between HC and PD at T0 and T1 or T2.

No other relevant results were reported.
Tags: ToM, behavioral

Theory of Mind in Parkinson's disease.

Item Type Journal Article
Author Morris Freedman
Author Donald T. Stuss
Abstract Theory of Mind is an important concept within social cognition and refers to the ability to attribute mental states to oneself and others. Other terms for this concept include mentalizing and mind reading. Deficits in Theory of Mind may contribute to behavioral abnormalities, such as paranoia and delusions that are common in dementia. There are several experimental tasks for measuring Theory of Mind. A classical example is the false belief test. Examples of other measures include tests of understanding metaphor, sarcasm, irony, deception, and faux pas, determining what a person is thinking or feeling from photographs of the eye region, and visual perspective taking. There are several anatomical areas related to Theory of Mind. These include regions within the frontal and temporal lobes, and temporoparietal junction. There is a small but emerging literature on Theory of Mind in Parkinson's disease (PD). The data suggest that Theory of Mind is impaired in PD and that the deficits precede the development of dementia. Future studies are needed to better define the nature of the Theory of Mind deficits in PD, as well as the impact of these deficits on clinical disability in this disorder.
Date 2011 Nov 15
Language eng
License Copyright © 2011 Elsevier B.V. All rights reserved.
Extra Place: Netherlands
Volume 310
Pages 225-227
Publication Journal of the neurological sciences
DOI 10.1016/j.jns.2011.06.004
Issue 1-2
Journal Abbr J Neurol Sci

ISSN 1878-5883 0022-510X
PMID 21705020
Date Added 6.7.2025, 19:09:40
Modified 5.9.2025, 14:35:52

Notes:

Not Included: not an empirical paper
Tags: EXCLUDED

Theory of Mind in Parkinson's disease.

Item Type Journal Article
Author Michele Poletti
Author Ivan Enrici
Author Ubaldo Bonuccelli
Author Mauro Adenzato
Abstract The ability to infer other people's mental states (i.e. Theory of Mind, ToM) is a major topic of interest in various neurological and psychiatric disorders. However, it is only recently that there has been an assessment of cognitive and affective components of ToM ability in neurodegenerative disorders. In this review, we examine studies investigating the ToM ability in Parkinson's disease (PD). Taken together, these studies provide preliminary evidence that ToM difficulties may occur in PD patients. In particular, these difficulties principally involve the cognitive component of ToM in the early stages of the disease. The spatio-temporal progression of dopamine depletion supports the hypothesis that the affective component may only be affected in the advanced stages of the disease. The relationships between executive functioning, dopaminergic therapies, and ToM in PD as well as the relationships between frontostriatal circuits and ToM processing are discussed.
Date 2011 Jun 1
Language eng
License Copyright © 2011 Elsevier B.V. All rights reserved.
Extra Place: Netherlands
Volume 219
Pages 342-350
Publication Behavioural brain research
DOI 10.1016/j.bbr.2011.01.010
Issue 2
Journal Abbr Behav Brain Res
ISSN 1872-7549 0166-4328
PMID 21238496
Date Added 6.7.2025, 19:09:40
Modified 5.9.2025, 14:51:56

Notes:

No Included: Not a systematic Review
Tags: EXCLUDED

Theory of mind performance in Parkinson's disease is associated with motor and cognitive functions, but not with symptom lateralization.

Item Type Journal Article
Author Lisa Nobis
Author Katharina Schindlbeck
Author Felicitas Ehlen
Author Hannes Tiedt
Author Charlotte Rewitzer
Author Annelien A. Duits
Author Fabian Klostermann
Abstract Next to the typical motor signs, Parkinson's disease (PD) goes along with neuropsychiatric symptoms, amongst others affecting social cognition. Particularly, Theory of Mind (ToM) impairments have mostly been associated with right hemispherical brain dysfunction, so that it might prevail in patients with left dominant PD. Fourty-four PD patients, twenty-four with left and twenty with right dominant motor symptoms, engaged in the Reading the Mind in the Eyes (RME) and the Faux Pas Detection Test (FPD) to assess affective and cognitive ToM. The results were correlated with performance in further cognitive tests, and analyzed with respect to associations with the side of motor symptom dominance and severity of motor symptoms. No association of ToM performance with right hemispheric dysfunction was found. RME results were inversely correlated with motor symptom severity, while FPD performance was found to correlate with the performance in verbal fluency tasks and the overall cognitive evaluation. Affective ToM was found associated with motor symptom severity and cognitive ToM predominantly with executive function, but no effect of PD lateralization on this was identified. The results suggest that deficits in social cognition occur as a sequel of the general corticobasal pathology in PD, rather than as a result of hemisphere-specific dysfunction.
Date 2017 Sep
Language eng
Extra Place: Austria
Volume 124
Pages 1067-1072
Publication Journal of neural transmission (Vienna, Austria : 1996)
DOI 10.1007/s00702-017-1739-2
Issue 9
Journal Abbr J Neural Transm (Vienna)
ISSN 1435-1463 0300-9564
PMID 28584926
Date Added 6.7.2025, 19:09:40
Modified 5.9.2025, 14:48:07

Notes:

Not Included: Only PD group.

Comparison between left and right dominant PD

No association of ToM performance with right hemispheric dysfunction was found. RME results were inversely correlated with motor symptom severity, while FPD performance was found to correlate with the performance in verbal fluency tasks and the overall cognitive evaluation. Affective ToM was found associated with motor symptom severity and cognitive ToM predominantly with executive function, but no effect of PD lateralization on this was identified. **The results suggest that deficits in social cognition occur as a sequel of the general corticobasal pathology in PD, rather than as a result of hemisphere-specific dysfunction.**

Tags: EXCLUDED

Theory of mind, empathy and eye gaze strategies during observation of a work of art: neurodegenerative pathologies.

Item Type Journal Article

Author Kevin Polet
Author Solange Hesse
Author Adeline Morisot
Author Benoît Kunmann
Author Sandrine Louchart de la Chapeue
Author Galina Iakimova
Author Alain Pesce

Abstract OBJECTIVE: Theory of mind (ToM) and empathy are severely impaired in the behavioural variant of frontotemporal dementia (bvFTD) and more mildly in Alzheimer's (AD) and Parkinson's diseases (PD). Such impairments are associated with behavioural disorders. Modification of visual scanning strategies of complex visual scenes is also found in these pathologies. We hypothesised that these patients applied atypical gaze strategies when observing social events, which does not allow them to properly process social cues and results in the production of erroneous inferences and lack of empathy towards others. METHODS: Fifty-five participants were divided into four groups: five bvFTD subjects, 19 AD subjects, 17 PD subjects and 14 matched control subjects. ToM and empathy were assessed by eye movement recording (eye-tracking) and by a questionnaire during a painting observation. Scores obtained were compared between each group and to social cognition reference tests and correlated to the NeuroPsychiatric Inventory. RESULTS: Our paradigm was suitable for assessing cognitive ToM while it lacked sensitivity for empathy assessment. Severe ToM impairment was highlighted in bvFTD, while milder difficulties were observed in AD and for PD. Subjects with bvFTD and AD produced erroneous inferences from cognitive mental states. ToM performances were linked to visual exploration strategies of the painting. Atypical visual observation was highlighted in the bvFTD and AD groups causing a time delay in taking the perspective of the other person. Finally, we highlighted that social cognition performances, gaze strategies and behavioural disorders were correlated. CONCLUSION: Observing a painting in association with eye-tracking technology can be a good support for social cognition assessment. We highlighted a link between

atypical visual scanning strategies, ToM impairment and behavioural disorders in these pathologies. ToM skills could be improved by training in the search for visual social cues. Therefore, this kind of remediation could have positive effects on behavioural disorders.

**Date**	2022 Feb 14
**Language**	eng
**Extra**	Place: France
**Publication**	Geriatrie et psychologie neuropsychiatrie du vieillissement
**DOI**	10.1684/pnv.2022.1017
**Journal Abbr**	Geriatr Psychol Neuropsychiatr Vieil
**ISSN**	2115-7863
**PMID**	35165082
**Date Added**	6.7.2025, 19:09:39
**Modified**	5.9.2025, 14:51:33

Notes:

Not included: no access

Tags: EXCLUDED

Theory of the mind in subjects with Parkinson's disease: A case-control study

**Item Type**	Journal Article
**Author**	Genny Giselle Arciniega-Martinez
**Author**	Adib Jorge de Sarachaga
**Author**	Jackeline Estephanie Simancas-Ruiz
**Author**	Marisa Selene Escobar-Barrios
**Author**	Elsa Edith Carreon-Bautista
**Author**	Linney Fabiola Balboa-Alegría
**Author**	Amin Cervantes-Arriaga
**Author**	Mayela Rodriguez-Violante
**Date**	2016
**URL**	https://www.sciencedirect.com/science/article/pii/S1353802015005581
**Volume**	22
**Pages**	e58
**Publication**	Parkinsonism & Related Disorders
**DOI**	https://doi.org/10.1016/j.parkreldis.2015.10.122
**ISSN**	1353-8020
**Date Added**	6.7.2025, 19:12:34
**Modified**	6.7.2025, 19:12:34

Notes:

Not Included: not an empirical paper
Tags: EXCLUDED

Thinking versus feeling: How interoception and cognition influence emotion recognition in behavioural-variant frontotemporal dementia, Alzheimer's disease, and Parkinson's disease.

**Item Type**	Journal Article
**Author**	Jessica L. Hazelton
**Author**	Sol Fittipaldi
**Author**	Matias Fraile-Vazquez
**Author**	Marion Sourty
**Author**	Agustina Legaz
**Author**	Anna L. Hudson
**Author**	Indira Garcia Cordero
**Author**	Paula C. Salamone
**Author**	Adrian Yoris
**Author**	Agustín Ibañez
**Author**	Olivier Piguet
**Author**	Fiona Kumfor
**Abstract**	Disease-specific mechanisms underlying emotion recognition difficulties in behavioural-variant frontotemporal dementia (bvFTD), Alzheimer's disease (AD), and Parkinson's disease (PD) are unknown. Interoceptive accuracy, accurately detecting internal cues (e.g., one's heart beating), and cognitive abilities are candidate mechanisms underlying emotion recognition. One hundred and sixty-eight participants (52 bvFTD; 41 AD; 24 PD; 51 controls) were recruited. Emotion recognition was measured via the Facial Affect Selection Task or the Mini-Social and Emotional Assessment Emotion Recognition Task. Interoception was assessed with a heartbeat detection task. Participants pressed a button each time they: 1) felt their heartbeat (Interoception); or 2) heard a recorded heartbeat (Exteroception-control). Cognition was measured via the Addenbrooke's Cognitive Examination-III or the Montreal Cognitive Assessment. Voxel-based morphometry analyses identified neural correlates associated with emotion recognition and interoceptive accuracy. All patient groups showed worse emotion recognition and cognition than controls (all P's ≤ .008). Only the bvFTD showed worse interoceptive accuracy than controls (P < .001). Regression analyses revealed that in bvFTD worse interoceptive accuracy predicted worse emotion recognition (P = .008). Whereas worse cognition predicted worse emotion recognition overall (P < .001). Neuroimaging analyses revealed that the insula, orbitofrontal cortex, and amygdala were involved in emotion recognition and interoceptive accuracy in bvFTD. Here, we provide evidence for disease-specific mechanisms for emotion recognition difficulties. In bvFTD, emotion recognition impairment is driven by inaccurate perception of the internal milieu. Whereas, in AD and PD, cognitive impairment likely underlies emotion recognition deficits. The current study furthers our theoretical understanding of emotion and highlights the need for targeted interventions.
**Date**	2023 Jun
**Language**	eng

License Copyright © 2023 The Author(s). Published by Elsevier Ltd.. All rights reserved.
Extra Place: Italy
Volume 163
Pages 66-79
Publication Cortex; a journal devoted to the study of the nervous system and behavior
DOI 10.1016/j.cortex.2023.02.009
Journal Abbr Cortex
ISSN 1973-8102 0010-9452
PMID 37075507
PMCID PMC11177281
Date Added 6.7.2025, 19:09:35
Modified 5.9.2025, 14:38:28

Notes:

Included

sample characteristics

size: 52 behavioural-variant frontotemporal dementia; 39 Alzheimer's disease; 22 Parkinson's disease; 50 controls

Parkinson's Disease type and duration: NA, NA

Medication: NA

Hoehn-Yahr: NA

UPDRS-3: NA

Gender (male): 12 males (55%)

averaged ages (SD, range): M=72.23 SD= 6.78

other neurological disease (tumor, stroke, etc.): None

other major psychopathology: None

origin country (or ethnicity): multi-centre: Australia Argentina and Chile

method observational and imaging

instruments used in order to quantify the variables

Social cognition aspect: emotion recognition

Name of the task: the Facial Affect Selection Task (FAST) **or** Emotion Recognition subtest of the Mini-Social and Emotional Assessment (SEA).

type of stimulus [face/voice etc., Ekman faces/other etc.]:

FAST - seven faces of the same individual posing several different emotional expressions

SEA - NA

task condition: fear, sadness, disgust, surprise, anger, happiness and neutral

operationalization: To account for potential differences in the tests used, z-scores were calculated relative to controls scores on the FAST or the mini-SEA.

MRI: Whole-brain structural MRI data were obtained using the standard imaging protocols of each research centre (see Supplementary Table 1). MRI images were collected from four MRI scanners (1: Phillips Ingenia 3.0 T, 224 × 224, 160 slices, echo time/repetition time = 3.8/8.3 ms; 2: Phillips Intera 1.5 T, 256 × 256, 196 slices, echo time/repetition time = 3.4/7.49 ms; 3: GE 3 T 256 × 256, 200 slices, echo time/repetition time = 2.5/6.7 ms; Scanner 4: Siemens Skyra 3.0 T, 256 × 256, 192 slices, echo time/repetition time = 2/2.4 ms) with 1 mm3 isotropic resolution and a flip angle α = 8°.

MRI scans were available for 138 participants (40 healthy controls, 34 AD patients, 46 bvFTD patients

and 18 PD patients).

MRI data were analysed using the FSL suite

First, structural images were brain-extracted using BET, then tissue segmentation was conducted via automatic segmentation (Zhang, Brady, & Smith, 2001). Next, grey matter partial volume maps were aligned to Montreal Neurological Institute (MNI) standard space (MNI152) using non-linear registration (FNIRT) which uses a b-spline representation of the registration warp field (Rueckert et al., 1999). A study specific template was created using participants representative of each research centre and diagnostic group, and the native grey matter images were non-linearly re-registered. Modulation of the registered partial volume maps were carried out by dividing them by the Jacobian of the warp field. Finally, the modulated, segmented images were smoothed with an isotropic Gaussian kernel with a sigma of 3 mm.

Main findings related to the review's scope

all patient groups performed worse than controls (bvFTD vs controls: $P < .001$; AD vs controls: $P < .001$; PD vs controls: $P = .008$), with no differences observed between patient groups.

MRI: No significant clusters were identified in PD.

Tags: Emotion recognition, Imaging, behavioral

To study the effect of a newly developed emotion detection and grading system software for identifying and grading expressions of patients with Parkinson's disease

**Item Type**	Journal Article
**Author**	Sushant Sarang
**Author**	Bhakti Sonawane
**Author**	Priyanka Sharma
**Author**	Rashmi Yeradkar
**Date**	2023-08-09
**Publication**	MULTIMEDIA TOOLS AND APPLICATIONS
**DOI**	10.1007/s11042-023-16156-5
**ISSN**	1380-7501
**Date Added**	14.7.2025, 14:48:39
**Modified**	5.9.2025, 14:55:43

Notes:

Not Included: Not on SC

Tags: EXCLUDED

Transcranial direct current stimulation enhances theory of mind in Parkinson's disease patients with mild cognitive impairment: a randomized, double-blind, sham-controlled study.

**Item Type**	Journal Article
**Author**	Mauro Adenzato
**Author**	Rosa Manenti
**Author**	Ivan Enrici
**Author**	Elena Gobbi
**Author**	Michela Brambilla
**Author**	Antonella Alberici
**Author**	Maria Sofia Cotelli
**Author**	Alessandro Padovani
**Author**	Barbara Borroni
**Author**	Maria Cotelli
**Abstract**	BACKGROUND: Parkinson's Disease (PD) with mild cognitive impairment (MCI) (PD-MCI) represents one of the most dreaded complications for patients with PD and is associated with a higher risk of developing dementia. Although transcranial direct current stimulation (tDCS) has been demonstrated to improve motor and non-motor symptoms in PD, to date, no study has investigated the effects of tDCS on Theory of Mind (ToM), i.e., the ability to understand and predict other people's behaviours, in PD-MCI. METHODS: In this randomized, double-blind, sham-controlled study, we applied active tDCS over the medial frontal cortex (MFC) to modulate ToM performance in twenty patients with PD-MCI. Twenty matched healthy controls (HC) were also enrolled and were asked to perform the ToM task without receiving tDCS. RESULTS: In the patients with PD-MCI, i) ToM performance was worse than that in the HC, ii) ToM abilities were poorer in those with fronto-executive difficulties, and iii) tDCS over the MFC led to significant shortening of latency for ToM tasks. CONCLUSIONS: We show for the first time that active tDCS over the MFC enhances ToM in patients with PD-MCI, and suggest that non-invasive brain stimulation could be used to ameliorate ToM deficits observed in these patients.
**Date**	2019
**Language**	eng
**Extra**	Place: England
**Volume**	8
**Pages**	1
**Publication**	Translational neurodegeneration
**DOI**	10.1186/s40035-018-0141-9
**Journal Abbr**	Transl Neurodegener
**ISSN**	2047-9158
**PMID**	30627430
**PMCID**	PMC6322239
**Date Added**	6.7.2025, 19:09:41
**Modified**	5.9.2025, 14:24:50

Notes:

Included

sample characteristics

size: 20 PD-MCI and 20 matched HC (by gender, age, education).

PD-type: PD-MCI

PD-duration: NA

Medication: All patients had been on stable pharmacological therapy for at least 6 months prior to entering the study.

Hoehn-Yahr: M = 1.8, SD = 0.7

UPDRS-3: M = 24.1, SD = 9.5

Gender: 10 male (50%)

Age (SD, Range): M = 65.5 (SD = 8.4)

Other neurological disease (tumor, stroke, etc.): None

Other major psychopathology: None

averaged ages (SD, range): not described.

origin country (or ethnicity): Italy

method observational

instruments used in order to quantify the variables

Social cognition aspect: Theory of mind

Name of the task: Mind in the Eyes task and Attribution of Intentions task

type of stimulus [face/voice etc., Ekman faces/other etc.]: Mind in the Eyes task – eye strips

Attribution of Intentions task – video version (short videos lasting 1500 milliseconds)

Attribution of Intentions task – a) the Private Intention condition (PInt), in which participants were required to recognize another person's intention while watching his/her isolated actions. b) the Communicative Intention condition (CInt), in which participants were required to recognize another person's communicative intention during a social interaction

operationalization:

Mind in the Eyes task – sum of correct responses

Attribution of Intentions task – accuracy rate and RT

Main findings related to the review's scope

Mind in the Eyes task – PD-MCI group performed worse than the HC group in this task

Attribution of Intentions task – PD-MCI group had less accuracy in this task than the HC group did

PD-MCI group was slower than the HC group in both CInt and PInt conditions
Tags: ToM, behavioral

Unaltered emotional experience in Parkinson's disease: Pupillometry and behavioral evidence.

Item Type Journal Article
Author Rachel Schwartz
Author Kathrin Rothermich
Author Sonja A. Kotz
Author Marc D. Pell
Abstract INTRODUCTION: Recognizing emotions in others is a pivotal part of socioemotional functioning and plays a central role in social interactions. It has been shown that individuals suffering from Parkinson's disease (PD) are less accurate at identifying basic emotions such as fear, sadness, and happiness; however, previous studies have predominantly assessed emotion processing using unimodal stimuli (e.g., pictures) that do not reflect the complexity of real-world processing demands. Dynamic, naturalistic stimuli (e.g., movies) have been shown to elicit stronger subjective emotional experiences than unimodal stimuli and can facilitate emotion recognition. METHOD: In this experiment, pupil measurements of PD patients and matched healthy controls (HC) were recorded while they watched short film clips. Participants' task was to identify the emotion elicited by each clip and rate the intensity of their emotional response. We explored (a) how PD affects subjective emotional experience in response to dynamic, ecologically valid film stimuli, and (b) whether there are PD-related changes in pupillary response, which may contribute to the differences in emotion processing reported in the literature. RESULTS: Behavioral results showed that identification of the felt emotion as well as perceived intensity varies by emotion, but no significant group effect was found. Pupil measurements revealed differences in dilation depending on the emotion evoked by the film clips (happy, tender, sadness, fear, and neutral) for both groups. CONCLUSIONS: Our results suggest that differences in emotional response may be negligible when PD patients and healthy controls are presented with dynamic, ecologically valid emotional stimuli. Given the limited data available on pupil response in PD, this study provides new evidence to suggest that the PD-related deficits in emotion processing reported in the literature may not translate to real-world differences in physiological or subjective emotion processing in early-stage PD patients.
Date 2018 Apr
Language eng
Extra Place: England
Volume 40
Pages 303-316
Publication Journal of clinical and experimental neuropsychology
DOI 10.1080/13803395.2017.1343802
Issue 3
Journal Abbr J Clin Exp Neuropsychol
ISSN 1744-411X 1380-3395

PMID 28669253
Date Added 6.7.2025, 19:09:37
Modified 5.9.2025, 14:56:15

Notes:

Included

Sample characteristics

Size: 17 PD, 20 HC (age and education machted)

PD-type: Idiopathic PD

PD-duration: M = 9.9, Sd = 6.2

Medication: NA

Hoehn-Yahr: NA

UPDRS-3: NA

Gender (male): 8 (47%)

Age: M = 64.7, Sd = 6.8

Other neurological disease (tumor, stroke, etc.): NA

Other major psychopathology: NA

Origin country (or ethnicity): Canada

method behavioural

instruments used in order to quantify the variables

Social cognition aspect: empathy

Name of the task: NA

Type of stimulus [face/voice etc., Ekman faces/other etc.]: we developed a set of film stimuli that could reliably elicit target emotions in adults between the ages of 50 and 80 years. We focused on four emotions for an experimental design balanced in terms of valence (two negative (sadness, fear), two positive(happiness, tenderness)).

Task condition:11 flim clips were used ((between 1:05 min and 2:05 min)

Operationalization: Accuracy if a participant felt the correct emotion. The experiened intensity (0-6)

Main findings related to the review's scope

Including group as a fixed effect did not significantly improve the model, $p > .9$, $b = -0.007$, R2 (group) = .0001,

Tags: empathy, behavioral

Understanding disrupted motivation in Parkinson’s disease through a value-based decision-making lens

**Item Type**	Journal Article
**Author**	Campbell Le Heron
**Author**	Lee-Anne Morris
**Author**	Sanjay Manohar
**Abstract**	Neurobehavioural disturbances such as loss of motivation have profound effects on the lives of many people living with Parkinson’s disease (PD), as well as other brain disorders. The field of decision-making neuroscience, underpinned by a plethora of work across species, provides an important framework within which to investigate apathy in clinical populations. Here we review how changes in a number of different processes underlying value-based decision making may lead to the common phenotype of apathy in PD. The application of computational models to probe both behaviour and neurophysiology show promise in elucidating these cognitive processes crucial for motivated behaviour. However, observations from the clinical management of PD demand an expanded view of this relationship, which we aim to delineate. Ultimately, effective treatment of apathy may depend on identifying the pattern in which decision making and related mechanisms have been disrupted in individuals living with PD.
**Date**	2025
**URL**	https://www.sciencedirect.com/science/article/pii/S0166223625000396
**Volume**	48
**Pages**	297-311
**Publication**	Trends in Neurosciences
**DOI**	https://doi.org/10.1016/j.tins.2025.02.008
**Issue**	4
**ISSN**	0166-2236
**Date Added**	6.7.2025, 19:12:36
**Modified**	5.9.2025, 14:39:01

Notes:

Not Included: not an empirical paper

Tags: EXCLUDED

Understanding facial emotion perception in Parkinson's disease: the role of configural processing.

**Item Type**	Journal Article
**Author**	Pauline Narme
**Author**	Anne-Marie Bonnet
**Author**	Bruno Dubois
**Author**	Laurence Chaby

Abstract Parkinson's disease (PD) has been frequently associated with facial emotion recognition impairments, which could adversely affect the social functioning of those patients. Facial emotion recognition requires processing of the spatial relations between facial features, known as the facial configuration. Few studies, however, have investigated this ability in people with PD. We hypothesized that facial emotion recognition impairments in patients with PD could be accounted for by a deficit in configural processing. To assess this hypothesis, three tasks were proposed to 10 patients with PD and 10 healthy controls (HC): (i) a facial emotion recognition task with upright faces, (ii) a similar task with upside-down faces, to explore the face inversion effect, and (iii) a configural task to assess participants' abilities to detect configural modifications made on a horizontal or vertical axis. The results showed that when compared with the HC group, the PD group had impaired facial emotion recognition, in particular for faces expressing anger and fear, and exhibited reduced face inversion effect for these emotions. More importantly, the PD group's performance on the configural task to detect vertical modifications was lower than the HC group's. Taken together, these results suggest the presence of a configural processing alteration in patients with PD, especially for vertical, second-order information. Furthermore, configural performance was positively correlated with emotion recognition for anger, disgust, and fear, suggesting that facial emotion recognition could be related, at least partially, to configural processing.

Date 2011 Oct
Language eng
License Copyright © 2011 Elsevier Ltd. All rights reserved.
Extra Place: England
Volume 49
Pages 3295-3302
Publication Neuropsychologia
DOI 10.1016/j.neuropsychologia.2011.08.002
Issue 12
Journal Abbr Neuropsychologia
ISSN 1873-3514 0028-3932
PMID 21856319
Date Added 6.7.2025, 19:09:38
Modified 5.9.2025, 14:47:53

Notes:

Included

Sample characteristics

Size: 10 PD, 10 HC(matched for age and education level)

PD-type: NA

PD-duration: M = 9.8, SD = 3.7

Medication: ON state

Hoehn-Yahr: Range = 1-3, M = 2.1

UPDRS-3: NA

Gender (male): 8 (80%)

Age: M = 63.2, SD = 8.3

Other neurological disease (tumor, stroke, etc.): None

Other major psychopathology: None

Origin country (or ethnicity): France

method (Review, meta-analysis or observational and/or self-reported):

instruments used in order to quantify the variables

Social cognition aspect: Facial expression recognöition

Name of the task: NA

Type of stimulus [face/voice etc., Ekman faces/other etc.]: Ten black and white photographs of faces (five female) were taken from the Ekman series. The images were cropped so that only the facial regions were shown,

Task condition: Each face was presented expressing four basic emotions (happiness, fear, disgust, and anger), plus a neutral expression for a total of 50 photographs.

Operationalization: The participants were required to (i) identify the emotions expressed on a set of 50 upright faces (Fig. 1A), and (ii) identify the emotions expressed on a set of 50 upside-down faces (this task was used to assess configural processing of facial emotion and to calculate the FIE

>> Correct answers

Main findings related to the review's scope

Facial expression recognition task

a lower accuracy for the PD group than the HC group in the upright condition only (for upright faces, 81.0 ± 3.1% versus 94.2 ± .9%, U = 11, z = −2.95, p = .003; for upside-down faces, 65.4 ± 3.6% versus 62.0 ± 3.9%, U = 44, z = 0.45, p = .7). For upright faces, mean accuracy was significantly lower for anger in PD (56 ± 27.2) than HC (90 ± 8.2, U = 6.5, z = −3.29, p = .001; all other comparisons p > .1). For upsidedown faces, not any significant group difference was found (all p > .1).

Tags: Emotion recognition, behavioral

Understanding the role of configural processing in face emotion recognition in Parkinson's disease.

Item Type Journal Article
Author Rosanna Cousins
Author Anne Pettigrew
Author Olivia Ferrie
Author J. Richard Hanley
Abstract This investigation examined whether impairment in configural processing could explain deficits in face emotion recognition in people with Parkinson's disease (PD). Stimuli from the Radboud Faces Database were used to compare recognition of four negative emotion expressions by older adults with PD (n = 16) and matched controls (n = 17). Participants were tasked with categorizing emotional expressions from upright and inverted whole faces and facial composites; it is difficult to derive configural information from these two types of stimuli so featural processing should play a larger than usual role in accurate recognition of emotional expressions. We found that the PD group were impaired relative to controls in recognizing anger, disgust and fearful expressions in upright faces. Then, consistent with a configural processing deficit, participants with PD showed no composite effect when attempting to identify facial expressions of anger, disgust and fear. A face inversion effect, however, was observed in the performance of all participants in both the whole faces and facial composites tasks. These findings can be explained in terms of a configural processing deficit if it is assumed that the disruption caused by facial composites was specific to configural processing, whereas inversion reduced performance by making it difficult to derive both featural and configural information from faces.
Date 2021 Apr
Language eng
License © 2020 The British Psychological Society.
Extra Place: England
Volume 15 Suppl 1
Pages 8-26
Publication Journal of neuropsychology
DOI 10.1111/jnp.12210
Journal Abbr J Neuropsychol
ISSN 1748-6653 1748-6645
PMID 32323929
Date Added 6.7.2025, 19:09:36
Modified 5.9.2025, 14:31:57

Notes:

Included

sample characteristics

size: 16 PD and 17 HC (matched for age and current verbal intelligence at time of testing consented to take part).

Parkinson's Disease type and duration: idiopathic, Mduration= 6.88 (SD=5.19) 0.25–15

Medication: on medication

Hoehn-Yahr: M=2.06 SD=0.66 1-3

UPDRS-3: M= NA

Gender (male): 10 males (62.5%)

averaged ages (SD, range): M= 70.13 (SD=10.38) 47–85

other neurological disease (tumor, stroke, etc.): None

other major psychopathology: None

origin country (or ethnicity): England

method observational

instruments used in order to quantify the variables

Social cognition aspect:

Name of the task: NA, the Radboud Faces Database

type of stimulus [face/voice etc., Ekman faces/other etc.]: The faces of 10 Caucasian people (five male) were selected. Four colour photographs of each face were used, making a total of 40 photographs

task condition: anger, disgust, fear, sadness

operationalization: accuracy

type of stimulus [face/voice etc., Ekman faces/other etc.]: For the composite stimuli, the 40 photographs were cut horizontally across the middle of the face, directly under the nose so that the eyes and the mouth were in separate parts: the eyes and the mouth are most important for expression recognition (Tanaka & Simonyi, **2016**). Composites were made from the same models for 12 different eyes/mouth emotion combinations:

task condition: anger/disgust, anger/fear and anger/sadness; disgust/anger, disgust/fear and disgust/sadness; fear/anger, fear/disgust and fear/sadness; and sadness/anger, sadness/disgust and sadness/fear.

The aligned composites were edited where necessary – using Adobe Photoshop – to occlude differences in shade and ensure there were no gaps, or sharp edges at the jawline or on the nose. Non-aligned composites were made following the principle of moving the top half to the left or to the right until the model's jawline was directly under the middle or the nose, and with no gap between the two parts.

Operationalization: the emotion to be identified was top 48 times and bottom 48 times.

Main findings related to the review's scope

PD group were impaired relative to controls in recognizing anger, disgust and fearful expressions in upright faces. Then, consistent with a configural processing deficit, participants with PD showed no composite effect when attempting to identify facial expressions of anger, disgust and fear. A face inversion effect, however, was observed in the performance of all participants in both the whole faces and facial composites tasks.

Full-face emotion expression recognition

For upright faces, the PD group exhibited impairment in recognition of Anger (U = 59.5; p = .002), Disgust (U = 69.0; p = .007) and Fear

The difference between the groups for Sadness was not statistically significant.

For inverted faces, controls were significantly more accurate than people with Parkinson's for Disgust (U = 68.0; p = .007) and Sadness.

The composite Paradigm

In the conventional upright condition, when considering the four negative emotion expressions together, the control group was significantly more accurate when composite face stimuli were not aligned than when aligned. For the PD group, this composite effect was absent.

Emotion specific analyses found no composite effect for anger, disgust or fearful for the PD group, although for sadness recognition was better for non-aligned composites

Examination of the inverted composite stimuli confirmed that there were no composite effects at all.

Tags: Emotion recognition, behavioral

Understanding the role of social cognition in neurodegenerative Disease: A scoping review on an overlooked problem.

Item Type Journal Article

Author Giuseppa Maresca
Author Maria Grazia Maggio
Author Desiree Latella
Author Antonino Naro
Author Simona Portaro
Author Rocco Salvatore Calabrò

Abstract Social cognition (SC) is the set of socio-cognitive processes that guide automatic and voluntary behaviors by modulating behavioral responses, it includes both cognitive (Theory of the mind - ToM) and affective aspects (Empathy). SC also includes representations of internal somatic states, self-knowledge, perception of others, communication with others and interpersonal motivations. SC is relevant in daily life

and reflects the neural complexity of social processing. The purpose of this scoping review is to evaluate the role of SC in neurological disorders, also considering the pathophysiological mechanisms underlying SC and potential assessment tools. The included studies were carried out between 2010 and 2019 and were found on PubMed, Scopus, Cochrane, and Web of Sciences databases, using the combined terms "social cognition"; "dementia"; "multiple sclerosis"; "parkinson", "amyotrophic lateral sclerosis", "neurodegenerative disease". Our review has shown that different SC domains are affected by several neurological conditions, with regards to dementia and amyotrophic lateral sclerosis. Further studies are needed to investigate the association between cognitive and social deficits, for a better management of patients with neurological disorders.

Date 2020 Jul
Language eng
License Copyright © 2020 Elsevier Ltd. All rights reserved.
Extra Place: Scotland
Volume 77
Pages 17-24
Publication Journal of clinical neuroscience : official journal of the Neurosurgical Society of Australasia
DOI 10.1016/j.jocn.2020.05.013
Journal Abbr J Clin Neurosci
ISSN 1532-2653 0967-5868
PMID 32389547
Date Added 6.7.2025, 19:09:41
Modified 5.9.2025, 14:44:55

Notes:

Not Included: not a systematic Review

Tags: EXCLUDED

Use of a Graphic Memoir to Enhance Clinicians' Understanding of and Empathy for Patients with Parkinson Disease.

Item Type Journal Article
Author Kimberly R. Myers
Author Daniel R. George
Author Xeumei Huang
Author Michael D. F. Goldenberg
Author L. J. Van Scoy
Author Erik Lehman
Author Michael J. Green
Abstract CONTEXT: Parkinson disease (PD) can be physically, emotionally, and financially burdensome. Understanding its impact from the patient's perspective is an important way to sensitize clinicians to the challenges of living with PD. OBJECTIVE: To evaluate whether a book-length graphic memoir (an illness story in comic form) can

help clinicians appreciate PD from the patient's perspective. DESIGN: A convergent mixed-methods study of clinicians working in a multidisciplinary movement disorders clinic. Participants read My Degeneration and completed preintervention and post-intervention questionnaires. They also attended a book group discussion. Quantitative findings were compared before and after the intervention, and qualitative data were analyzed for themes. MAIN OUTCOME MEASURES: Clinicians': 1) confidence in understanding patients' experiences with PD, 2) knowledge about PD, and 3) empathy toward patients and families. RESULTS: After reading the book, participants' confidence in understanding patients' experiences with PD increased significantly in the areas of stigma and disease impact on patients and families. Clinical knowledge was unchanged. Qualitative analysis revealed 3 main themes: 1) the book provides a meaningful way for clinicians to learn about the experience of living with PD; 2) the medium of comics engages clinicians in ways different from other mediums; and 3) benefits of the book may extend beyond the clinical team. CONCLUSION: Clinicians who read My Degeneration gained insight into the psychosocial effects of PD on patients and their loved ones. The book helped facilitate deeper understanding of patients' experiences living with PD and fostered greater empathy and self-reflection.

**Date**	2019
**Language**	eng
**Extra**	Place: United States
**Volume**	24
**Publication**	The Permanente journal
**DOI**	10.7812/TPP/19.060
**Journal Abbr**	Perm J
**ISSN**	1552-5775 1552-5767
**PMID**	31710837
**PMCID**	PMC6844551
**Date Added**	6.7.2025, 19:09:39
**Modified**	5.9.2025, 14:47:46

Notes:

Not Included: No comparison goup. Only pre-post data after training intervention in PD

Tags: EXCLUDED

Visual dysfunction in Parkinson's disease

**Item Type**	Journal Article
**Author**	Rimona S. Weil
**Author**	Anette E. Schrag
**Author**	Jason D. Warren
**Author**	Sebastian J. Crutch
**Author**	Andrew J. Lees
**Author**	Huw R. Morris

Abstract Visual symptoms are frequently reported in Parkinson's disease. Weil et al.. relate visual changes to underlying brain regions, and consider mechanisms for visual hallucinations. They examine links between visual changes and other features of Parkinson's disease and discuss the role of visual dysfunction as a marker of dementia.Visual symptoms are frequently reported in Parkinson's disease. Weil et al.. relate visual changes to underlying brain regions, and consider mechanisms for visual hallucinations. They examine links between visual changes and other features of Parkinson's disease and discuss the role of visual dysfunction as a marker of dementia.Patients with Parkinson's disease have a number of specific visual disturbances. These include changes in colour vision and contrast sensitivity and difficulties with complex visual tasks such as mental rotation and emotion recognition. We review changes in visual function at each stage of visual processing from retinal deficits, including contrast sensitivity and colour vision deficits to higher cortical processing impairments such as object and motion processing and neglect. We consider changes in visual function in patients with common Parkinson's disease-associated genetic mutations including GBA and LRRK2. We discuss the association between visual deficits and clinical features of Parkinson's disease such as rapid eye movement sleep behavioural disorder and the postural instability and gait disorder phenotype. We review the link between abnormal visual function and visual hallucinations, considering current models for mechanisms of visual hallucinations. Finally, we discuss the role of visuo-perceptual testing as a biomarker of disease and predictor of dementia in Parkinson's disease.

Date 2016-11
Language English
Extra Place: GREAT CLARENDON ST, OXFORD OX2 6DP, ENGLAND Type: Review
Volume 139
Publisher OXFORD UNIV PRESS
Pages 2827-2843
Publication BRAIN
DOI 10.1093/brain/aww175
Issue 11
ISSN 0006-8950
Date Added 14.7.2025, 14:50:36
Modified 5.9.2025, 15:00:51

Notes:

Not Included: Does not study SC aspects

Tags: EXCLUDED

Visual exploration of emotional facial expressions in Parkinson's disease

Item Type Journal Article

Author Uraina S. Clark
Author Sandy Neargarder
Author Alice Cronin-Golomb

Abstract Parkinson's disease (PD) is associated with impairments in facial emotion recognition as well as visual and executive dysfunction. We investigated whether facial emotion categorization impairments in PD are attributable to visual scanning abnormalities by recording the eye movements of 16 non-demented PD and 20 healthy control (HC) participants during an emotion recognition task. We examined the influence of several factors that can affect visual scanning, including oculomotor, basic visual, and cognitive abilities (executive function). Increases in the number and duration of fixations in the top regions of surprise facial expressions were related to increases in recognition accuracy for this emotion in PD participants with left-sided motor-symptom onset. Compared to HC men, HC women spent less time fixating on fearful expressions. PD participants displayed oculomotor abnormalities (antisaccades), but these were unrelated to scanning patterns. Performance on visual measures (acuity, contrast sensitivity) correlated with scanning patterns in the PD group only. Poorer executive function was associated with longer fixation times in PD and with a greater number of fixations in HC. Our findings indicate a specific relation between facial emotion categorization impairments and scanning of facial expressions in PD. Furthermore, PD and HC participants' scanning behaviors during an emotion categorization task were driven by different perceptual processes and cognitive strategies. Our results underscore the need to consider differences in perceptual and cognitive abilities in studies of visual scanning, particularly when examining this ability in patient populations for which both vision and cognition are impaired. (C) 2010 Elsevier Ltd. All rights reserved.

Date 2010-06
Language English
Extra Place: THE BOULEVARD, LANGFORD LANE, KIDLINGTON, OXFORD OX5 1GB, ENGLAND Type: Article
Volume 48
Publisher PERGAMON-ELSEVIER SCIENCE LTD
Pages 1901-1913
Publication NEUROPSYCHOLOGIA
DOI 10.1016/j.neuropsychologia.2010.03.006
Issue 7
ISSN 0028-3932
Date Added 14.7.2025, 14:50:41
Modified 5.9.2025, 14:31:15

Notes:

Included

sample characteristics

size: 16 PD and 20 HC (match in age and education)

Parkinson's Disease type and duration: idiopathic PD, Mduration=5.9 SD=3.16

Medication: on medication

Hoehn-Yahr: Md=2 (2-3)

UPDRS-3: NA

Gender (male): 8 males (50%)

averaged ages (SD, range): M= 59.7 SD=6.7

other neurological disease (tumor, stroke, etc.): None

other major psychopathology: None

origin country (or ethnicity): USA

method observational

instruments used in order to quantify the variables

Social cognition aspect: Emotion recognition

Name of the task: NA

type of stimulus [face/voice etc., Ekman faces/other etc.]: 70 black-and-white photographs taken from the Ekman and Friesen Pictures of Facial Affect database

task condition: Angry, Disgust, Fear, Happy, Sad and Surprise, plus Neutral

operationalization: Accuracy and Eye-movement

Eye movement recordings: In Experiments 1 and 2, eye movements were monitored with the ViewPoint EyeTracker (Version 2.11; Arrington Research, Inc., Scottsdale, AZ), which was run on a Macintosh G4 desktop computer. Software included with the eye tracking system was used to present stimuli and record data. The system has a sampling rate of 30 Hz. Eye movement recordings were captured using a camera (mounted to an adjustable stand) with an attached infrared light source, which allowed eye movement recordings using a pupil location technique.

Main findings related to the review's scope

Accuracy: compared to HC, PD participants were impaired at recognizing facial expressions of Anger and Surprise

Eye-movement: The PD and HC groups did not differ significantly in the total number of fixations made to the seven emotional facial expressions, total time spent fixating on the emotional facial

expressions, or mean fixation durations

no significant group difference in the number or duration of fixations made to the top vs. the bottom regions of facial expressions

Tags: Emotion recognition, behavioral

Visuo-constructional functions in patients with mild cognitive impairment, Alzheimer’s disease, and Parkinson’s disease

**Item Type**	Journal Article
**Author**	Johann Lehrner
**Author**	Harald Krakhofer
**Author**	Claus Lamm
**Author**	Stefan Macher
**Author**	Doris Moser
**Author**	Stefanie Klug
**Author**	Peter Dal-Bianco
**Author**	Walter Pirker
**Author**	Eduard Auff
**Author**	Gisela Pusswald
**Date**	10/2015
**Language**	en
**Library Catalog**	DOI.org (Crossref)
**URL**	http://link.springer.com/10.1007/s40211-015-0141-2
**Accessed**	18.1.2026, 23:01:05
**Volume**	29
**Pages**	112-119
**Publication**	neuropsychiatrie
**DOI**	10.1007/s40211-015-0141-2
**Issue**	3
**Journal Abbr**	Neuropsychiatr
**ISSN**	0948-6259, 2194-1327
**Date Added**	18.1.2026, 23:01:05
**Modified**	18.1.2026, 23:01:05

Visuo-spatial interference affects the identification of emotional facial expressions in unmedicated Parkinson's patients

**Item Type**	Journal Article
**Author**	Beatriz Garcia-Rodriguez
**Author**	Carmen Casares Guillen
**Author**	Rosa Jurado Barba

Author Gabriel Rubio Valladolid
Author Jose Antonio Molina Arjona
Author Heiner Ellgring
Abstract There is evidence that visuo-spatial capacity can become overloaded when processing a secondary visual task (Dual Task, DT), as occurs in daily life. Hence, we investigated the influence of the visuo-spatial interference in the identification of emotional facial expressions (EFEs) in early stages of Parkinson's disease (PD). We compared the identification of 24 emotional faces that illustrate six basic emotions in, unmedicated recently diagnosed PD patients (16) and healthy adults (20), under two different conditions: a) simple EFE identification, and b) identification with a concurrent visuo-spatial task (Corsi Blocks). EFE identification by PD patients was significantly worse than that of healthy adults when combined with another visual stimulus. Published by Elsevier B.V.
Date 2012-02-15
Language English
Extra Place: RADARWEG 29, 1043 NX AMSTERDAM, NETHERLANDS Type: Article; Proceedings Paper
Volume 313
Publisher ELSEVIER
Pages 13-16
Publication JOURNAL OF THE NEUROLOGICAL SCIENCES
DOI 10.1016/j.jns.2011.09.041
Issue 1-2
ISSN 0022-510X
Date Added 14.7.2025, 14:50:40
Modified 5.9.2025, 14:36:12

Notes:

Included

sample characteristics

size: 16 PD and 20 HC (matched with respect to age, gender ratio, years of education, Mini-Mental State Examination, Blessed Scale and Geriatric Depression Scale)

Parkinson's Disease type and duration: *de novo* outpatients with a clinical diagnosis of idiopathic PD

Medication: off medication

Hoehn-Yahr: NA max=2

UPDRS-3: NA

Gender (male): 9 males (56%)

averaged ages (SD, range): M= 73.19 SD=9.21 (49–86)

other neurological disease (tumor, stroke, etc.): no dementia

other major psychopathology: none

origin country (or ethnicity): Spain

method observational

instruments used in order to quantify the variables

Social cognition aspect: emotion recognition

Name of the task: NA. forced-choice recognition task was used for Emotional Facial Expression identification

type of stimulus [face/voice etc., Ekman faces/other etc.]: virtual faces on a computer screen that represented the six basic emotions. the emotions were represented by four virtual characters created by 3D imaging (two male and two female avatars). The expressions were generated by an experienced *Facial Action Coding System* [47], [48] coder (JHE) by activating the virtual muscles with respect to single Action Units.

task condition: happiness, sadness, surprise, anger, fear and disgust

operationalization: emotional facial expression (EFE) performance was tested under two conditions: a) simple EFE identification; and b) EFE identification concurrent with the Corsi task. >> number of correct responses provided for all emotional faces.

Main findings related to the review's scope

all participants identified more emotional stimuli in the simple EFE identification condition than when required to perform a concurrent secondary task

PD perfomed worse than HC

The dual task had greater cost for the PD than HC

Tags: Emotion recognition, behavioral

Vocal emotion decoding in the subthalamic nucleus: An intracranial ERP study in Parkinson's disease

**Item Type**	Journal Article
**Author**	Julie Péron
**Author**	Olivier Renaud
**Author**	Claire Haegelen
**Author**	Lucas Tamarit
**Author**	Valérie Milesi
**Author**	Jean-François Houvenaghel
**Author**	Thibaut Dondaine
**Author**	Marc Vérin
**Author**	Paul Sauleau
**Author**	Didier Grandjean
**Abstract**	Using intracranial local field potential (LFP) recordings in patients with Parkinson's disease (PD) undergoing deep brain stimulation (DBS), we explored the electrophysiological activity of the subthalamic nucleus (STN) in response to emotional stimuli in the auditory modality. Previous studies focused on the influence of visual stimuli. To this end, we recorded LFPs within the STN in response to angry, happy, and neutral prosodies in 13 patients with PD who had just undergone implantation of DBS electrodes. We observed specific modulation of the right STN in response to anger and happiness, as opposed to neutral prosody, occurring at around 200–300ms post-onset, and later at around 850–950ms post-onset for anger and at around 3250–3350ms post-onset for happiness. Taken together with previous reports of modulated STN activity in response to emotional visual stimuli, the present results appear to confirm that the STN is involved in emotion processing irrespective of stimulus valence and sensory modality.
**Date**	2017
**URL**	https://www.sciencedirect.com/science/article/pii/S0093934X16301559
**Volume**	168
**Pages**	1-11
**Publication**	Brain and Language
**DOI**	https://doi.org/10.1016/j.bandl.2016.12.003
**ISSN**	0093-934X
**Date Added**	6.7.2025, 19:12:35
**Modified**	5.9.2025, 14:50:48

Notes:

Not Included: Only PD-group post DBS-OP

Vocal emotion decoding in the subthalamic nucleus: An intracranial ERP study in Parkinson's disease

Tags: EXCLUDED

Vocal emotion processing in Parkinson's disease: reduced sensitivity to negative emotions.

Item Type Journal Article

Author Chinar Dara
Author Laura Monetta
Author Marc D. Pell

Abstract To document the impact of Parkinson's disease (PD) on communication and to further clarify the role of the basal ganglia in the processing of emotional speech prosody, this investigation compared how PD patients identify basic emotions from prosody and judge specific affective properties of the same vocal stimuli, such as valence or intensity. Sixteen non-demented adults with PD and 17 healthy control (HC) participants listened to semantically-anomalous pseudo-utterances spoken in seven emotional intonations (anger, disgust, fear, sadness, happiness, pleasant surprise, neutral) and two distinct levels of perceived emotional intensity (high, low). On three separate occasions, participants classified the emotional meaning of the prosody for each utterance (identification task), rated how positive or negative the stimulus sounded (valence rating task), or rated how intense the emotion was expressed by the speaker (intensity rating task). Results indicated that the PD group was significantly impaired relative to the HC group for categorizing emotional prosody and showed a reduced sensitivity to valence, but not intensity, attributes of emotional expressions conveying anger, disgust, and fear. The findings are discussed in light of the possible role of the basal ganglia in the processing of discrete emotions, particularly those associated with negative vigilance, and of how PD may impact on the sequential processing of prosodic expressions.

Date 2008 Jan 10
Language eng
Extra Place: Netherlands
Volume 1188
Pages 100-111
Publication Brain research
DOI 10.1016/j.brainres.2007.10.034
Journal Abbr Brain Res
ISSN 0006-8993
PMID 18022608
Date Added 6.7.2025, 19:09:39
Modified 5.9.2025, 14:32:34

Notes:

Included

sample characteristics

size: 16 PD and 17 HC (matched for age and education)

Parkinson's Disease type and duration: idiopathic PD, Mduration = 8.2 years (S.D.=3.6, Range=3.0–27.3).

Medication: On medication

Hoehn-Yahr: M=2.7

UPDRS-3: NA

Gender (male): 9 males (57%)

averaged ages (SD, range): M= 66.0 (S.D.=9.0)

other neurological disease (tumor, stroke, etc.): NA

other major psychopathology: NA

origin country (or ethnicity): NA

method observational

instruments used in order to quantify the variables

Social cognition aspect: Emotion recognition

Name of the task: NA

type of stimulus [face/voice etc., Ekman faces/other etc.]: emotional prosody in various ways, with or without congruent semantic cues. In the three main tasks, language-like "pseudo-utterances" (e.g., Someone migged the pazing) were presented which were semantically anomalous but which communicated specific emotions unambiguously through the prosody. In the remaining task (prosody-semantic emotion identification), utterances of similar length and complexity were presented which contained both semantic and prosodic cues which communicated the intended emotion (e.g.,for sad: I didn't make the team). Utterances in all tasks were approximately 6–10 syllables in length and, once recorded, ranged between 1.2 and 2.5 s in duration when spoken naturally to communicate different target emotions. All stimuli were taken from a database of emotionally-inflected utterances in English which is described in detail elsewhere (Pell, 2002; Pell et al., in review). Stimuli were digitally recorded in a sound attenuated chamber by six male and six female actors to express seven distinct emotions

task condition: anger, disgust, fear, sadness, neutral, happiness, and pleasant surprise

operationalization:

In the **pure prosody** emotion identification task, participants listened to each utterance and then identified the emotion of the speaker based on their prosodic features in a seven forced-choice response format (alternatives=anger, disgust, fear, sadness, neutral, happiness, pleasant surprise).

In the **affective valence** rating task, participants listened to the utterances on a different occasion and

rated how positive or negative the speaker sounds in reference to a continuous scale (where −3=very negative and +3=very positive).

In the **affective intensity** rating task, participants rated the strength of the emotion produced by the speaker following each of the utterances (where 1=not at all strong and 5=very strong).

Main findings related to the review's scope

Pure-prosody identification (only prosody conveyed emotional meanings)**:**

Group (2) x emotions (7)

PD group made more errors overall than the HC group

neutral utterances (M=0.81) were identified more accurately by both groups than all other emotions

No sig interaction

Group (2) x valance (2) x intensity (2)

PD group performed less accurately than the HC group overall.

all participants were more accurate to identify negative emotions when they were high (M=0.67) versus low (M=0.44) in intensity, whereas accuracy rates did not differ for positive emotions of high and low intensity.

No sig interaction with group

Valence rating:

Group (2) x emotions (7)

for three of the emotions–anger, disgust, and fear–the PD group differed from the HC group by assigning significantly higher valence ratings to these emotions

the mean ratings for the PD group fell on the positive side of the rating scale, in contrast to what was observed for the HC group.

The groups did not differ in how they rated sadness, happiness, pleasant surprise, or neutral utte rances.

Group (2) x valance (2) x intensity (2)

PD group assigned higher (i.e., more positive) valence ratings to the class of negative emotions than the HC group, whereastherewasnodifferenceinhowthetwogroups assigned ratings to the class of positive emotions.

Intensity rating:

Group (2) x emotions (7)

In general, the participants rated anger, fear, and surprise as mostintense, greater thandisgust, which in turn was more intense than happiness and sadness, which in turn exceeded neutral.

No group or interaciton effects

Group (2) x valance (2) x intensity (2)

No group or interaction with group were found.
Tags: Emotion recognition, behavioral

[Voxel-Based Morphometry and Social Cognitive Function in Parkinson's Disease].

Item Type Journal Article
Author Mutsutaka Kobayakawa
Abstract In recent years, voxel-based morphometry (VBM) has been increasingly used to investigate local brain structures in Parkinson's disease (PD). Compared to healthy participants, PD patients tend to show reduced brain volume in limbic and paralimbic areas in early disease stages, while the neocortical areas, such as the temporal and frontal cortices, are affected in advanced patients. Moreover, there are many studies showing correlation between cognitive performance and local brain volume. In the last decade, emotional and social cognitive function, such as facial emotion recognition, emotional decision-making, and theory of mind, have been the target of VBM analysis in PD. These studies facilitate understanding of the nature of communication and behavioral disorders that some PD patients display. However, there are discrepancies in VBM results between studies. Future studies need to employ larger sample sizes and combined analysis of brain perfusion or network connectivity in order to confirm brain structural changes and their effects on social cognitive function in PD patients.
Date 2017 Nov
Language jpn
Extra Place: Japan
Volume 69
Pages 1323-1329
Publication Brain and nerve = Shinkei kenkyu no shinpo
DOI 10.11477/mf.1416200911
Issue 11
Journal Abbr Brain Nerve
ISSN 1881-6096
PMID 29172197
Date Added 6.7.2025, 19:09:34
Modified 5.9.2025, 14:41:53

Notes:

Not Included: Not in English
Tags: EXCLUDED

What Do We Know about Theory of Mind Impairment in Parkinson's Disease?

Item Type Journal Article

Author Clara Trompeta
Author Beatriz Fernández Rodríguez
Author Carmen Gasca-Salas
Abstract Theory of mind (ToM) is a social cognitive skill that involves the ability to attribute mental states to self and others (what they think (cognitive ToM) and feel (affective ToM)). We aim to provide an overview of previous knowledge of ToM in Parkinson's disease (PD). In the last few years more attention has been paid to the study of this construct as a non-motor manifestation of PD. In advanced stages, both components of ToM (cognitive and affective) are commonly impaired, although in early PD results remain controversial. Executive dysfunction correlates with ToM deficits and other cognitive domains such as language and visuospatial function have also been related to ToM. Recent studies have demonstrated that PD patients with mild cognitive impairment show ToM deficits more frequently in comparison with cognitively normal PD patients. In addition to the heterogeneity of ToM tests administered in different studies, depression and dopaminergic medication may also be acting as confounding factors, but there are still insufficient data to support this. Neuroimaging studies conducted to understand the underlying networks of cognitive and affective ToM deficits in PD are lacking. The study of ToM deficit in PD continues to be important, as this may worsen quality of life and favor social stigma. Future studies should be considered, including assessment of the patients' cognitive state, associated mood disorders, and the role of dopaminergic deficit.
Date 2021 Sep 24
Language eng
Extra Place: Switzerland
Volume 11
Publication Behavioral sciences (Basel, Switzerland)
DOI 10.3390/bs11100130
Issue 10
Journal Abbr Behav Sci (Basel)
ISSN 2076-328X
PMID 34677223
PMCID PMC8533307
Date Added 6.7.2025, 19:09:40
Modified 5.9.2025, 14:58:54

Notes:

Not Included: not a systematic review

Seems really interesting though!

Tags: EXCLUDED

What Happens When I Watch a Ballet and I Am Dyskinetic? A fMRI Case Report in Parkinson Disease.

Item Type Journal Article
Author Sara Palermo

Author Rosalba Morese
Author Maurizio Zibetti
Author Alberto Romagnolo
Author Edoardo Giovanni Carlotti
Author Andrea Zardi
Author Maria Consuelo Valentini
Author Alessandro Pontremoli
Author Leonardo Lopiano
Abstract BACKGROUND: The identical sets of neurons - the mirror neuron system (MNS) - can be activated by simply observing specific, specific movements, decoded behaviors and even facial expressions performed by other people. The same neurons activated during observation are those recruited during the same movements and actions. Hence the mirror system plays a central role in observing and executing movements. Little is known about MNS in a neurodegenerative motor disorder, such as Parkinson's Disease (PD) is. METHODS: We explored the neural correlates potentially involved in empathy and embodiment in PD through complex action observation of complex behaviors like the choreutical arts. An integrated multidisciplinary assessment (neurological, neuropsychiatric, and neuropsychological) was used for the selection of the PD candidate for the neuroimaging experimental acquisition. For the first time in literature the famous Calvo-Merino's paradigm was administered to a PD subject. KEY POINTS: Functional magnetic resonance imaging (fMRI) exploratory analysis shows the recruitment of the left thalamus, the right dorsolateral prefrontal cortex, and the bilateral superior precentral gyrus (one of the main hubs of the MNS). If the observed choreic movement becomes part of the observer's motor repertoire experience, mirror neurons might activate stimulating affective empathy and making the understanding of movement an own proper body experience (cognitive embodiment). MAIN LESSONS: Our study sheds light on a possible use of complex action observation to improve or slow the deterioration of motor abilities and levodopa-induced dyskinesias in PD patients. Indeed, the modulation of the neural area involved in complex action observation could be considered a promising target for neuro-rehabilitative intervention mediated by the elicitation of the MNS.
Date 2020
Language eng
License Copyright © 2020 Palermo, Morese, Zibetti, Romagnolo, Carlotti, Zardi, Valentini, Pontremoli and Lopiano.
Extra Place: Switzerland
Volume 11
Pages 1999
Publication Frontiers in psychology
DOI 10.3389/fpsyg.2020.01999
Journal Abbr Front Psychol
ISSN 1664-1078
PMID 32849162
PMCID PMC7426453
Date Added 6.7.2025, 19:09:42
Modified 5.9.2025, 14:48:47

Notes:

Not Included: Case-report study
Tags: EXCLUDED

White matter hyperintensities associated with small vessel disease impair social cognition beside attention and memory

**Item Type**	Journal Article
**Author**	Jana Kynast
**Author**	Leonie Lampe
**Author**	Tobias Luck
**Author**	Stefan Frisch
**Author**	Katrin Arelin
**Author**	Karl-Titus Hoffmann
**Author**	Markus Loeffler
**Author**	Steffi G. Riedel-Heller
**Author**	Arno Villringer
**Author**	Matthias L. Schroeter
**Abstract**	Age-related white matter hyperintensities (WMH) are a manifestation of white matter damage seen on magnetic resonance imaging (MRI). They are related to vascular risk factors and cognitive impairment. This study investigated the cognitive profile at different stages of WMH in a large community-dwelling sample; 849 subjects aged 21 to 79 years were classified on the 4-stage Fazekas scale according to hyperintense lesions seen on individual T2-weighted fluid-attenuated inversion recovery MRI scans. The evaluation of cognitive functioning included seven domains of cognitive performance and five domains of subjective impairment, as proposed by the DSM-5. For the first time, the impact of age-related WMH on Theory of Mind was investigated. Differences between Fazekas groups were analyzed non-parametrically and effect sizes were computed. Effect sizes revealed a slight overall cognitive decline in Fazekas groups 1 and 2 relative to healthy subjects. Fazekas group 3 presented substantial decline in social cognition, attention and memory, although characterized by a high inter-individual variability. WMH groups reported subjective cognitive decline. We demonstrate that extensive WMH are associated with specific impairment in attention, memory, social cognition, and subjective cognitive performance. The detailed neuropsychological characterization of WMH offers new therapeutic possibilities for those affected by vascular cognitive decline.
**Date**	2018-06
**Language**	English
**Extra**	Place: 2455 TELLER RD, THOUSAND OAKS, CA 91320 USA Type: Article
**Volume**	38
**Publisher**	SAGE PUBLICATIONS INC
**Pages**	996-1009
**Publication**	JOURNAL OF CEREBRAL BLOOD FLOW AND METABOLISM
**DOI**	10.1177/0271678X17719380
**Issue**	6
**ISSN**	0271-678X
**Date Added**	14.7.2025, 14:50:34

Modified 5.9.2025, 14:42:33

Notes:

Not Included: Not on Parkinson

However, on WM on large sample.
Tags: EXCLUDED

White matter microstructures in Parkinson's disease with and without impulse control behaviors.

Item Type Journal Article
Author Haruka Takeshige-Amano
Author Taku Hatano
Author Koji Kamagata
Author Christina Andica
Author Wataru Uchida
Author Masahiro Abe
Author Takashi Ogawa
Author Yasushi Shimo
Author Genko Oyama
Author Atsushi Umemura
Author Masanobu Ito
Author Masaaki Hori
Author Shigeki Aoki
Author Nobutaka Hattori
Abstract BACKGROUND: Impulse control behaviors (ICBs) in Parkinson's disease (PD) are thought to be caused by an overdose of dopaminergic therapy in the relatively spared ventral striatum, or by hypersensitivity of this region to dopamine. Alterations in brain networks are now also thought to contribute to the development of ICBs. OBJECTIVE: To comprehensively assess white matter microstructures in PD patients with ICBs using advanced diffusion MRI and magnetization transfer saturation (MT-sat) imaging. METHODS: This study included 19 PD patients with ICBs (PD-ICBs), 18 PD patients without ICBs (PD-nICBs), and 20 healthy controls (HCs). Indices of diffusion tensor imaging (DTI), diffusion kurtosis imaging, neurite orientation dispersion and density imaging, and MT-sat imaging were evaluated using tract-based spatial statistics (TBSS), regions of interest (ROIs), and tract-specific analysis (TSA). RESULTS: Compared with HCs, PD-nICBs had significant alterations in many major white matter tracts in most parameters. In contrast, PD-ICBs had only partial changes in several parameters. Compared with PD-ICBs, TBSS, ROI, and TSA analyses revealed that PD-nICBs had lower axial kurtosis, myelin volume fraction, and orientation dispersion index in the uncinate fasciculus and external capsule, as well as in the retrolenticular part of the internal capsule. These are components of the reward system and the visual and emotional perception areas, respectively. INTERPRETATION: Myelin and axonal changes in fibers related to the reward system and visual emotional recognition might be more prominent in PD-nICBs than

in PD-ICBs.

Date 2022 Mar
Language eng
License © 2022 The Authors. Annals of Clinical and Translational Neurology published by Wiley Periodicals LLC on behalf of American Neurological Association.
Extra Place: United States
Volume 9
Pages 253-263
Publication Annals of clinical and translational neurology
DOI 10.1002/acn3.51504
Issue 3
Journal Abbr Ann Clin Transl Neurol
ISSN 2328-9503
PMID 35137566
PMCID PMC8935280
Date Added 6.7.2025, 19:09:37
Modified 5.9.2025, 14:58:16

Notes:

Not Included: Does not experimentally study SC

Still interesting though due to the imaging
Tags: EXCLUDED

White matter pathways and social cognition

Item Type Journal Article
Author Yin Wang
Author Athanasia Metoki
Author Kylie H. Alm
Author Ingrid R. Olson
Abstract There is a growing consensus that social cognition and behavior emerge from interactions across distributed regions of the "social brain". Researchers have traditionally focused their attention on functional response properties of these gray matter networks and neglected the vital role of white matter connections in establishing such networks and their functions. In this article, we conduct a comprehensive review of prior research on structural connectivity in social neuroscience and highlight the importance of this literature in clarifying brain mechanisms of social cognition. We pay particular attention to three key social processes: face processing, embodied cognition, and theory of mind, and their respective underlying neural networks. To fully identify and characterize the anatomical architecture of these networks, we further implement probabilistic tractography on a large sample of diffusion-weighted imaging data. The combination of an in-depth literature review and the empirical investigation gives us an unprecedented, well-defined landscape of white matter pathways underlying major social brain networks. Finally, we discuss current problems in the field, outline

suggestions for best practice in diffusion-imaging data collection and analysis, and offer new directions for future research.

Date 2018
URL https://www.sciencedirect.com/science/article/pii/S0149763417306474
Volume 90
Pages 350-370
Publication Neuroscience & Biobehavioral Reviews
DOI https://doi.org/10.1016/j.neubiorev.2018.04.015
ISSN 0149-7634
Date Added 6.7.2025, 19:12:36
Modified 5.9.2025, 15:00:42

Notes:

Not Included: not specifically on PD

Though interesting Information on PD is included!
Tags: EXCLUDED

White matter pathways associated with empathy in females: A DTI investigation

Item Type Journal Article
Author Stephanie N. Steinberg
Author Neami B. Tedla
Author Erin Hecht
Author Diana L. Robins
Author Tricia Z. King
Abstract Empathy is a component of social cognition that allows us to understand, perceive, experience, and respond to the emotional state of others. In this study, we seek to build on previous research that suggests that sex and hormone levels may impact white matter microstructure. These white matter microstructural differences may influence social cognition. We examine the fractional anisotropy (FA) of white matter pathways associated with the complex human process of empathy in healthy young adult females during the self-reported luteal phase of their menstrual cycle. We used tract-based spatial statistics to perform statistical comparisons of FA and conducted multiple linear regression analysis to examine the strength of association between white matter FA and scores on the Empathy Quotient (EQ), a self-report questionnaire in which individuals report how much they agree or disagree with 60 statements pertaining to their empathic tendencies. Results identified a significant negative relationship between EQ scores and FA within five clusters of white matter: in the left forceps minor/body of the corpus callosum, left corticospinal tract, intraparietal sulcus/primary somatosensory cortex, superior longitudinal fasciculus, and right inferior fronto-occipital fasciculus/forceps minor. These consistent findings across clusters suggest that lower self-reported empathy is related to higher FA across healthy young females in specific white matter regions during the menstrual luteal phase. Future research should seek to examine if self-reported empathy varies across

the menstrual cycle, using blood samples to confirm cycle phase and hormone levels.

Date 2022
URL https://www.sciencedirect.com/science/article/pii/S0278262622000604
Volume 162
Pages 105902
Publication Brain and Cognition
DOI https://doi.org/10.1016/j.bandc.2022.105902
ISSN 0278-2626
Date Added 6.7.2025, 19:12:36
Modified 5.9.2025, 14:57:38

Notes:

Not Included: No PD group

Tags: EXCLUDED

Working memory and facial expression recognition in patients with Parkinson's disease.

Item Type Journal Article
Author Laura Alonso-Recio
Author Pilar Martín-Plasencia
Author Ángela Loeches-Alonso
Author Juan M. Serrano-Rodríguez
Abstract Facial expression recognition impairment has been reported in Parkinson's disease. While some authors have referred to specific emotional disabilities, others view them as secondary to executive deficits frequently described in the disease, such as working memory. The present study aims to analyze the relationship between working memory and facial expression recognition abilities in Parkinson's disease. We observed 50 patients with Parkinson's disease and 49 healthy controls by means of an n-back procedure with four types of stimuli: emotional facial expressions, gender, spatial locations, and non-sense syllables. Other executive and visuospatial neuropsychological tests were also administered. Results showed that Parkinson's disease patients with high levels of disability performed worse than healthy individuals on the emotional facial expression and spatial location tasks. Moreover, spatial location task performance was correlated with executive neuropsychological scores, but emotional facial expression was not. Thus, working memory seems to be altered in Parkinson's disease, particularly in tasks that involve the appreciation of spatial relationships in stimuli. Additionally, non-executive, facial emotional recognition difficulty seems to be present and related to disease progression.
Date 2014 May
Language eng
Extra Place: England
Volume 20
Pages 496-505
Publication Journal of the International Neuropsychological Society : JINS

DOI 10.1017/S1355617714000265
Issue 5
Journal Abbr J Int Neuropsychol Soc
ISSN 1469-7661 1355-6177
PMID 24713515
Date Added 6.7.2025, 19:09:37
Modified 5.9.2025, 14:25:56

Notes:

Not Included: not on SC
Tags: EXCLUDED

Young-Onset Dementia and Neurodegenerative Disorders of the Young With an Emphasis on Clinical Manifestations

Item Type Journal Article
Author Kaynaat Fatima
Author Ashok M. Mehendale
Author Himabindu Reddy
Abstract Young-onset dementia (YOD) refers to a neurological ailment primarily affecting people below 65 years of age in roughly about 8% of cases found through various researches. The high rate of prevalence of secondary dementias among older patients proves that younger people show a better prognosis of the conditions causing dementia than older people. However, effective interventions have to be usually provided early in the course of cognitive decline to help facilitate cognitive improvement. The risk of development of prodromal dementia is high if there is a development of psychoses in middle-aged or older people. When there is a development of psychoses in middle to late life, the likelihood of this indicates prodromal dementia is high. The clinical presentation is quite variable and often subtle in frontotemporal dementia (FTD) but may be dominated by personality change, behavioral disturbances, motivation, or the loss of empathy. There is great heterogeneity in the probable causes of dementia in young age as compared to dementia in old age, and some observed differences also exist in the course and characteristics of the disease. These causes may range from the most probable cause such as Alzheimer's disease (AD) to causes with low probability, such as metabolic disorders and prion diseases. The symptoms of young-onset dementia include a gradual development of personality and behavioral changes over a period of years. However, in the initial stages of young-onset dementia, this change can be attributed to various issues, such as depression, marital problems, and menopause. Other neurodegenerative diseases such as Huntington's disease show presentations such as changes in personality, chorea, and depression that can be observed in patients in their early adulthood. A few other neurodegenerative disorders are myoclonic epilepsy with ragged red fibers (MERRF) and mitochondrial encephalopathy, lactic acidosis, and stroke-like episodes (MELAS) with presentations such as characterized muscle weakness, poor growth, problems with vision and hearing, and the involvement of the multi-organ system, including the central nervous system to name a few. There is also the prevalence of juvenile parkinsonism in the community, which represents a group

of clinicopathological entities present before the age of 21. Young-onset Parkinson's disease (PD) (YOPD) appears to have the same pathological presentation as late-onset Parkinson's disease (LOPD). Recent researches have proved that "gene therapy" can be useful in the treatment and in preventing the progression of symptoms in cases of neurodegenerative diseases.

Date 2022-10-07
Language English
Extra Place: CAMPUS, 4 CRINAN ST, LONDON, N1 9XW, ENGLAND Type: Review
Volume 14
Publisher SPRINGERNATURE
Publication CUREUS JOURNAL OF MEDICAL SCIENCE
DOI 10.7759/cureus.30025
Issue 10
Date Added 14.7.2025, 14:50:28
Modified 5.9.2025, 14:35:38

Notes:

Not Included: not an empirical paper

Tags: EXCLUDED
