# Supplementary material for: Social cognition in Parkinson’s disease: a comprehensive systematic review and integrative conceptual framework
Source: Front Aging Neurosci. 2026 Jun 29;18:1863728. doi: 10.3389/fnagi.2026.1863728 (PMC13357429; doi:10.3389/fnagi.2026.1863728)
Supplement: Supplementary file 2 [file Data_Sheet_2.docx]

**Risk-of-Bias Assessment and Quality of Evidence**

**Summary**

Overall, the methodological quality of the primary empirical studies was generally moderate to high. Across the empirical studies, most appraisal ratings were either “Yes” or “Mostly Yes”, with “Yes” accounting for 68% of all item-level judgments and “Mostly Yes” for 29%. The strongest domains were the validity of PD diagnosis, where 82% of studies reported that PD diagnosis was established using a valid and reliable method. Another strong domain was the validity/reliability of SC or neuroimaging outcome measures, where 85% measured the outcomes using validated, standardized, or well-established tools. Confounding factors were also commonly identified, with 79% of studies identified and measured the main confounders relevant to its design, sample, and outcome measures. Alongside 71% of the studies that appropriately handled them in the statistical analyses or the design of the study. However, several recurring limitations were evident. Description of study subjects and setting was often incomplete, with only 55% of studies described both the PD and control groups in adequate demographic and clinical detail, and 42% of them provided a generally adequate description of the sample and setting, but some details were missing. This reflecting inconsistent reporting of key PD clinical characteristics such as disease duration, H&Y stage, UPDRS-III, medication status, LEDD, and ON/OFF state. The clearest weakness concerned clinical comparability between groups: only 27% of studies were rated “Yes” for comparability on clinical or disease-relevant variables, whereas 61% were rated “Mostly Yes”. This suggests that although most studies considered important clinical variables, comparability regarding mood symptoms, global cognition, disease severity, medication status, and other potential confounders was often only partially established.

In contrast, the included reviews and meta-analyses showed more substantial methodological limitations. Review questions and inclusion criteria were usually adequate, with 60% that formulated clearly the study question around its PICO (Population, Intervention, Comparator, Outcome) elements, and 73% had identifiable inclusion criteria matching the review question. Search strategies were also generally acceptable, with 67% rated “Yes”, although search resources were less consistently adequate: only 40% were rated “Yes”, while 33% were rated “No”. The most consistent weaknesses were the absence of formal critical appraisal and duplicate appraisal procedures, with 87% having an inappropriate appraisal criteria or whether critical appraisal was conducted. Methods to minimize data extraction errors were also frequently insufficiently reported, with 67% of them were unclear on weather duplicates and independently were dealt with (or how). Lastly, publication bias was assessed in only 40% of reviews. Taken together, these findings indicate that the primary empirical evidence base is relatively strong but limited by incomplete clinical characterization and heterogeneous control of confounding, whereas the secondary evidence base is more vulnerable to methodological limitations related to study appraisal, data extraction procedures, and publication-bias assessment.

| Study | Q1 | Q2 | Q3 | Q4a | Q4b | Q5 | Q6 | Q7 | Q8 |
| --- | --- | --- | --- | --- | --- | --- | --- | --- | --- |
| (Adamaszek et al., 2019) | Mostly Yes | Yes | Yes | Yes | Mostly Yes | Yes | Yes | Yes | Yes |
| (Adenzato et al., 2019) | Yes | Yes | Yes | Yes | Yes | Yes | Yes | Yes | Yes |
| (Adolphs et al., 1998) | Yes | Mostly Yes | Yes | Yes | Mostly Yes | Yes | Yes | Yes | Yes |
| (Aiello et al., 2014) | Mostly Yes | Mostly Yes | Yes | Yes | Mostly Yes | Yes | Mostly Yes | Yes | Yes |
| (Albuquerque et al., 2016) | Yes | Mostly Yes | Yes | Yes | Mostly Yes | Yes | Yes | Yes | Yes |
| (Alonso-Recio et al., 2021) | Yes | Mostly Yes | Yes | Yes | Yes | Yes | Yes | Yes | Yes |
| (Alonso-Recio, Martín, et al., 2014) | Yes | Mostly Yes | Yes | Yes | Mostly Yes | Yes | Yes | Yes | Yes |
| (Alonso-Recio, Serrano, et al., 2014) | Yes | Mostly Yes | Yes | Yes | Mostly Yes | Yes | Yes | Yes | Yes |
| (Anderson et al., 2013) | Mostly Yes | Yes | Yes | Mostly Yes | Mostly Yes | Yes | Yes | Yes | Yes |
| (Anzani et al., 2024) | Yes | Mostly Yes | Yes | Yes | Yes | Yes | Yes | Yes | Yes |
| (Argaud et al., 2016) | Mostly Yes | Yes | Yes | Yes | Mostly Yes | Yes | Yes | Yes | Yes |
| (Ariatti et al., 2008) | Yes | Yes | Yes | Mostly Yes | Mostly Yes | Yes | Yes | Yes | Yes |
| (Assogna et al., 2010) | Yes | Mostly Yes | Yes | Yes | Mostly Yes | Yes | Yes | Yes | Yes |
| (Baggio et al., 2012) | Yes | Yes | Yes | Yes | Mostly Yes | Yes | Yes | Yes | Yes |
| (Bauer et al., 2023) | Mostly Yes | No | Yes | Mostly Yes | Unclear | Yes | Yes | Yes | Yes |
| (Beatty et al., 1989) | Yes | Mostly Yes | Yes | Mostly Yes | Mostly Yes | Yes | Yes | Yes | Yes |
| (Bediou et al., 2012) | Mostly Yes | No | Yes | Mostly Yes | Unclear | Mostly Yes | Mostly Yes | Yes | Yes |
| (Bek et al., 2020) | Yes | Yes | Yes | No | Mostly Yes | Yes | Yes | Yes | Yes |
| (Benke et al., 1998) | Yes | Yes | Yes | Mostly Yes | Mostly Yes | Yes | Yes | Mostly Yes | Yes |
| (Blonder et al., 1989) | Yes | Mostly Yes | Yes | Yes | Mostly Yes | Yes | Yes | Yes | Yes |
| (Bodden, Mollenhauer, et al., 2010) | Yes | Mostly Yes | Yes | Yes | Mostly Yes | Yes | Yes | Yes | Yes |
| (Bologna et al., 2016) | Yes | Yes | Yes | Yes | Yes | Yes | Yes | Yes | Yes |
| (Borod et al., 1990) | Yes | No | Yes | Mostly Yes | Yes | Yes | Yes | Yes | Yes |
| (Breitenstein et al., 2001) | Yes | Yes | Yes | Yes | Yes | Yes | Yes | Yes | Yes |
| (Breitenstein et al., 1998) | Yes | Mostly Yes | Yes | Yes | Yes | Yes | Yes | Yes | Yes |
| (Burgio et al., 2024) | Yes | Unclear | Yes | No | Mostly Yes | Yes | Yes | Yes | Yes |
| (Buxton et al., 2013) | Yes | Yes | Yes | Yes | Mostly Yes | Yes | Yes | Yes | Yes |
| (Caballero et al., 2022) | Mostly Yes | Mostly Yes | Mostly Yes | Yes | Mostly Yes | Yes | Yes | Yes | Yes |
| (Chiang et al., 2024) | Yes | Yes | Yes | Yes | Mostly Yes | Mostly Yes | Mostly Yes | Yes | Yes |
| (Chuang et al., 2022) | Yes | Yes | Yes | Mostly Yes | Mostly Yes | Yes | Yes | Yes | Yes |
| (Ciccarelli et al., 2022) | Yes | Yes | Yes | Yes | Mostly Yes | Yes | Yes | Yes | Yes |
| (Clark et al., 2008) | Yes | Yes | Yes | Yes | Mostly Yes | Yes | Yes | Yes | Yes |
| (Clark et al., 2010) | Yes | Mostly Yes | Yes | Yes | Mostly Yes | Yes | Yes | Yes | Yes |
| (Cohen et al., 2010) | Yes | Mostly Yes | Yes | Mostly Yes | Mostly Yes | Yes | Yes | Yes | Yes |
| (Costa et al., 2013) | Yes | Mostly Yes | Yes | Mostly Yes | Mostly Yes | Yes | Yes | Yes | Yes |
| (Coundouris et al., 2022) | Mostly Yes | Mostly Yes | Yes | Yes | Mostly Yes | Yes | Yes | Yes | Yes |
| (Cousins et al., 2021) | Yes | Mostly Yes | Mostly Yes | Mostly Yes | Mostly Yes | Yes | Yes | Yes | Yes |
| (Czernecki et al., 2021) | Yes | Yes | Yes | Mostly Yes | Mostly Yes | Yes | Yes | Yes | Yes |
| (Dan et al., 2019) | Yes | Yes | Yes | Yes | Yes | Yes | Yes | Yes | Yes |
| (Dara et al., 2008) | Yes | Yes | Yes | Yes | Mostly Yes | Yes | Yes | Yes | Yes |
| (De Risi et al., 2018) | Yes | Yes | Yes | Yes | Mostly Yes | Yes | Yes | Yes | Yes |
| (Del Prete et al., 2020) | Yes | Mostly Yes | Yes | Mostly Yes | Mostly Yes | Yes | Yes | Yes | Mostly Yes |
| (Delaveau et al., 2009) | Yes | Yes | Yes | Mostly Yes | Mostly Yes | Yes | Yes | Yes | Yes |
| (Di Tella et al., 2021) | Yes | Yes | Yes | Yes | Yes | Yes | Yes | Mostly Yes | Yes |
| (Di Tella et al., 2025) | Yes | Mostly Yes | Yes | Mostly Yes | Mostly Yes | Yes | Yes | Yes | Yes |
| (Díez-Cirarda et al., 2015) | Yes | Yes | Yes | Yes | Yes | Yes | Yes | Yes | Yes |
| (Dodich et al., 2022) | Yes | Yes | Yes | Mostly Yes | Yes | Yes | Yes | Yes | Yes |
| (Dujardin et al., 2004) | Yes | Yes | Yes | Yes | Mostly Yes | Yes | Yes | Yes | Yes |
| (Eddy et al., 2013) | Mostly Yes | Mostly Yes | Yes | Mostly Yes | Unclear | Yes | Yes | Yes | Yes |
| (Enrici et al., 2015) | Yes | Yes | Yes | Yes | Mostly Yes | Yes | Yes | Yes | Yes |
| (Enrici et al., 2017) | Yes | Yes | Yes | Yes | Yes | Yes | Yes | Yes | Yes |
| (Eriksson et al., 2022) | Mostly Yes | Yes | Yes | Mostly Yes | Mostly Yes | Yes | Mostly Yes | Yes | Yes |
| (Esteves et al., 2018) | Yes | Mostly Yes | Yes | Mostly Yes | Yes | Yes | Yes | Yes | Yes |
| (Euteneuer et al., 2009) | Yes | Yes | Yes | Yes | Mostly Yes | Yes | Mostly Yes | Yes | Yes |
| (Fernández-Fernández et al., 2024) | Yes | Yes | Yes | Yes | Mostly Yes | Yes | Yes | Yes | Yes |
| (Fleury et al., 2014) | Yes | Mostly Yes | Yes | Yes | Mostly Yes | Yes | Yes | Yes | Mostly Yes |
| (Foley et al., 2019) | Mostly Yes | Mostly Yes | Yes | Yes | Mostly Yes | Yes | Yes | Yes | Yes |
| (Foul et al., 2024) | Yes | Mostly Yes | Yes | Mostly Yes | Mostly Yes | Yes | Yes | Yes | Yes |
| (Funghi et al., 2025) | Yes | Yes | Yes | Mostly Yes | Mostly Yes | Yes | Yes | Yes | Yes |
| (Garcia-Rodriguez et al., 2012) | Yes | Mostly Yes | Yes | Yes | Yes | Yes | Yes | Mostly Yes | Yes |
| (Garrido-Vasquez et al., 2013) | Yes | Yes | Yes | Yes | Mostly Yes | Yes | Yes | Yes | Yes |
| (Glozman et al., 2003) | Unclear | Mostly Yes | Unclear | Mostly Yes | Unclear | Mostly Yes | Mostly Yes | No | Mostly Yes |
| (Haeske-Dewick, 1996) | Yes | Mostly Yes | Unclear | Mostly Yes | Mostly Yes | Mostly Yes | Yes | Mostly Yes | Yes |
| (Han et al., 2025) | Yes | Yes | Yes | Yes | Mostly Yes | Yes | Mostly Yes | Mostly Yes | Yes |
| (Hazelton et al., 2023) | Yes | Unclear | Yes | Mostly Yes | Mostly Yes | Mostly Yes | Yes | Yes | Yes |
| (Heller et al., 2018) | Yes | Yes | Yes | Yes | Mostly Yes | Yes | Yes | Yes | Yes |
| (Herrera et al., 2011) | Yes | Mostly Yes | Yes | Yes | Yes | Yes | Yes | Mostly Yes | Yes |
| (Hipp et al., 2014) | Yes | Yes | Yes | Mostly Yes | Mostly Yes | Yes | Yes | Yes | Yes |
| (Hirai et al., 2019) | Yes | Mostly Yes | Yes | Mostly Yes | Mostly Yes | Mostly Yes | Yes | Yes | Yes |
| (Ho et al., 2020) | Yes | Mostly Yes | Yes | Mostly Yes | Mostly Yes | Yes | Mostly Yes | Mostly Yes | Yes |
| (Hu et al., 2021) | Yes | Yes | Yes | Yes | Mostly Yes | Yes | Mostly Yes | Mostly Yes | Yes |
| (Ibañez et al., 2021) | Yes | Mostly Yes | Yes | Yes | Yes | Mostly Yes | Yes | Yes | Yes |
| (Ibarretxe-Bilbao et al., 2009) | Yes | Yes | Yes | Yes | Yes | Yes | Yes | Yes | Yes |
| (Ille et al., 2016) | Yes | Yes | Yes | Yes | Yes | Yes | Yes | Yes | Yes |
| (Irmen et al., 2017) | Yes | Yes | Yes | Yes | Yes | Yes | Yes | Mostly Yes | Yes |
| (Jacobs et al., 1995) | Unclear | No | Unclear | Mostly Yes | Unclear | Mostly Yes | Mostly Yes | Yes | Mostly Yes |
| (Jin et al., 2017) | Yes | Yes | Yes | Yes | Yes | Yes | Mostly Yes | Yes | Yes |
| (Kalampokini et al., 2018) | Yes | Yes | Yes | Yes | Mostly Yes | Yes | Yes | Yes | Yes |
| (Kan et al., 2002) | Mostly Yes | Mostly Yes | Unclear | Mostly Yes | Unclear | Mostly Yes | Yes | Yes | Mostly Yes |
| (Kuehne et al., 2023) | Yes | Mostly Yes | Yes | Yes | Yes | Mostly Yes | Yes | Yes | Yes |
| (Laskowska et al., 2015) | Mostly Yes | Mostly Yes | Yes | Yes | Mostly Yes | Yes | Mostly Yes | Mostly Yes | Yes |
| (Lawrence et al., 2007) | Yes | Mostly Yes | Yes | Yes | Mostly Yes | Mostly Yes | Yes | Yes | Mostly Yes |
| (Le Jeune et al., 2008) | Yes | Yes | Yes | Yes | Yes | Yes | Yes | Yes | Yes |
| (Li et al., 2022) | Yes | Mostly Yes | Yes | Mostly Yes | Yes | Yes | Yes | Yes | Yes |
| (Lima et al., 2013) | Yes | Yes | Yes | Yes | Yes | Yes | Yes | Yes | Yes |
| (Lin et al., 2016) | Mostly Yes | Yes | Yes | Mostly Yes | Mostly Yes | Mostly Yes | Yes | Mostly Yes | Mostly Yes |
| (Longo et al., 2025) | Mostly Yes | Mostly Yes | Yes | Mostly Yes | Mostly Yes | Mostly Yes | Mostly Yes | Yes | Yes |
| (Lundqvist et al., 2017) | Mostly Yes | Mostly Yes | Yes | Yes | Yes | Yes | Yes | Yes | Mostly Yes |
| (Maggi et al., 2024) | Yes | Yes | Yes | Mostly Yes | Mostly Yes | Yes | Yes | Yes | Yes |
| (Marneweck & Hammond, 2014) | Mostly Yes | Yes | Yes | Yes | Mostly Yes | Yes | Yes | Yes | Yes |
| (Marneweck et al., 2014) | Mostly Yes | Mostly Yes | Yes | Mostly Yes | Mostly Yes | Yes | Yes | Yes | Yes |
| (Martinez et al., 2018) | Yes | Mostly Yes | Yes | Mostly Yes | Mostly Yes | Mostly Yes | Mostly Yes | Yes | Mostly Yes |
| (Martínez-Corral et al., 2010) | Yes | Yes | Yes | Yes | Yes | Yes | Yes | Yes | Yes |
| (A. Martins et al., 2008) | Yes | Mostly Yes | Yes | Yes | Mostly Yes | Mostly Yes | Mostly Yes | Mostly Yes | Mostly Yes |
| (M. I. Martins et al., 2024) | Yes | Mostly Yes | Yes | Yes | Mostly Yes | Mostly Yes | Mostly Yes | Yes | Mostly Yes |
| (Mattavelli et al., 2021) | Mostly Yes | Yes | Unclear | Yes | Mostly Yes | Yes | Mostly Yes | Yes | Yes |
| (McIntosh et al., 2014) | Yes | Mostly Yes | Yes | Yes | Mostly Yes | Mostly Yes | Yes | Yes | Mostly Yes |
| (McKinlay et al., 2013) | Yes | Mostly Yes | Yes | Mostly Yes | Mostly Yes | Yes | Yes | Yes | Yes |
| (McNamara et al., 2010) | Yes | Mostly Yes | Yes | No | Mostly Yes | Yes | Mostly Yes | Mostly Yes | Mostly Yes |
| (Mengelberg & Siegert, 2003) | Yes | Mostly Yes | Yes | Unclear | Mostly Yes | Mostly Yes | Mostly Yes | Yes | Mostly Yes |
| (Menozzi et al., 2025) | Yes | Yes | Mostly Yes | Mostly Yes | Unclear | Mostly Yes | Yes | Mostly Yes | Mostly Yes |
| (Mimura et al., 2006) | Yes | Mostly Yes | Unclear | Yes | Mostly Yes | Yes | Mostly Yes | Yes | Mostly Yes |
| (Mitchell & Boucas, 2009) | Yes | Mostly Yes | Yes | Mostly Yes | Mostly Yes | Mostly Yes | Mostly Yes | Mostly Yes | Mostly Yes |
| (Mondillon et al., 2012) | Yes | Yes | Yes | Yes | Yes | Yes | Yes | Yes | Mostly Yes |
| (Monetta et al., 2009) | Yes | Mostly Yes | Yes | Yes | Mostly Yes | Yes | Mostly Yes | Yes | Mostly Yes |
| (Narme et al., 2011) | Yes | Mostly Yes | Yes | Yes | Yes | Yes | Yes | Yes | Yes |
| (Narme et al., 2013) | Yes | Yes | Yes | Yes | Mostly Yes | Yes | Yes | Yes | Yes |
| (Orduz-Bastidas et al., 2020) | Mostly Yes | Mostly Yes | Mostly Yes | Mostly Yes | Unclear | Mostly Yes | Mostly Yes | Yes | Mostly Yes |
| (Orso et al., 2020) | Yes | Yes | Yes | Yes | Yes | Yes | Yes | Yes | Yes |
| (Ozzoude et al., 2022) | Yes | Mostly Yes | Yes | No | No | Yes | Mostly Yes | Yes | Yes |
| (Palmeri et al., 2020) | Yes | Mostly Yes | Yes | Mostly Yes | Mostly Yes | Yes | Mostly Yes | Yes | Mostly Yes |
| (Paulmann & Pell, 2010) | Yes | Yes | Yes | Yes | Mostly Yes | Yes | Mostly Yes | Yes | Yes |
| (Pell & Leonard, 2003) | Yes | Yes | Yes | Yes | Mostly Yes | Yes | Mostly Yes | Yes | Yes |
| (Pell et al., 2014) | Yes | Yes | Yes | Yes | Mostly Yes | Yes | Mostly Yes | Yes | Yes |
| (Pell & Leonard, 2005) | Yes | Yes | Yes | Yes | Mostly Yes | Yes | Yes | Yes | Yes |
| (Péron, Biseul, et al., 2010) | Yes | Yes | Yes | Mostly Yes | Mostly Yes | Yes | Yes | Yes | Mostly Yes |
| (Peron et al., 2014) | Yes | Yes | Yes | Yes | Mostly Yes | Yes | Yes | Yes | Yes |
| (Peron et al., 2010) | Yes | Yes | Yes | Yes | Mostly Yes | Yes | Yes | Yes | Yes |
| (Péron, Le Jeune, et al., 2010) | Yes | Yes | Yes | Yes | Yes | Yes | Yes | Yes | Yes |
| (Péron et al., 2009) | Yes | Mostly Yes | Yes | Mostly Yes | Mostly Yes | Yes | Mostly Yes | Yes | Mostly Yes |
| (Pietschnig et al., 2016) | Yes | Yes | Yes | Mostly Yes | No | Yes | Yes | Yes | Yes |
| (Pläschke et al., 2017) | Mostly Yes | Mostly Yes | Yes | Mostly Yes | Unclear | Mostly Yes | Mostly Yes | Mostly Yes | Yes |
| (Poletti et al., 2013) | Mostly Yes | Mostly Yes | Mostly Yes | Mostly Yes | No | Yes | Yes | Yes | Mostly Yes |
| (Pontieri et al., 2012) | Mostly Yes | Yes | Yes | Yes | Mostly Yes | Yes | Mostly Yes | Yes | Mostly Yes |
| (Rabini et al., 2024) | Mostly Yes | Mostly Yes | Mostly Yes | No | Unclear | Mostly Yes | Mostly Yes | No | Mostly Yes |
| (Raffo De Ferrari et al., 2015) | Yes | Yes | Yes | Yes | Mostly Yes | Yes | Yes | Yes | Mostly Yes |
| (Ricciardi et al., 2015) | Mostly Yes | Yes | Yes | Yes | Yes | Yes | Yes | Yes | Yes |
| (Ricciardi et al., 2017) | Yes | Yes | Yes | Yes | Yes | Yes | Yes | Yes | Mostly Yes |
| (Righi et al., 2023) | Yes | Yes | Yes | Yes | Yes | Yes | Yes | Yes | Yes |
| (Robert et al., 2014) | Yes | Yes | Yes | NA | NA | Yes | Yes | Yes | Mostly Yes |
| (Rodríguez-Antigüedad et al., 2024) | Yes | Yes | Yes | NA | NA | Yes | Yes | Yes | Yes |
| (Romosan et al., 2019) | Yes | Mostly Yes | Yes | Yes | No | Yes | Yes | Yes | Yes |
| (Rosen et al., 2013) | Yes | Yes | Mostly Yes | Yes | No | Yes | Mostly Yes | Yes | Yes |
| (Rosen et al., 2015) | Yes | Yes | Yes | Yes | No | Yes | Mostly Yes | Yes | Yes |
| (Rossetto et al., 2018) | Yes | Yes | Yes | Mostly Yes | Mostly Yes | Yes | Mostly Yes | Yes | Yes |
| (Saenz et al., 2013) | Mostly Yes | Yes | Unclear | Yes | No | Yes | Mostly Yes | Mostly Yes | Yes |
| (Saffarian et al., 2019) | Yes | Mostly Yes | Unclear | Yes | Mostly Yes | Mostly Yes | Mostly Yes | Yes | Mostly Yes |
| (Salfi et al., 2024) | Yes | Yes | Unclear | Yes | Mostly Yes | Yes | Yes | Yes | Yes |
| (Saltzman et al., 2000) | Mostly Yes | Mostly Yes | Mostly Yes | Yes | Mostly Yes | Mostly Yes | Yes | Yes | Mostly Yes |
| (Santangelo et al., 2020) | Yes | Mostly Yes | Yes | Yes | Mostly Yes | Yes | Yes | Yes | Yes |
| (Santangelo et al., 2012) | Yes | Yes | Yes | Yes | Mostly Yes | Yes | Yes | Yes | Yes |
| (Sarasso et al., 2021) | Yes | Yes | Mostly Yes | Yes | Mostly Yes | Yes | Yes | Mostly Yes | Mostly Yes |
| (Schröder et al., 2006) | Yes | Yes | Mostly Yes | Yes | Yes | Mostly Yes | Yes | Mostly Yes | Mostly Yes |
| (Schwartz et al., 2018) | Mostly Yes | Mostly Yes | Yes | Yes | Mostly Yes | Mostly Yes | Mostly Yes | Mostly Yes | Mostly Yes |
| (Sedda et al., 2017) | Yes | Mostly Yes | Yes | Yes | Mostly Yes | Mostly Yes | Mostly Yes | Yes | Yes |
| (Seubert-Ravelo et al., 2021) | Yes | Yes | Yes | Yes | Mostly Yes | Yes | Yes | Yes | Yes |
| (Shafiei et al., 2020) | Yes | Mostly Yes | Yes | Yes | Yes | Mostly Yes | Yes | Mostly Yes | Mostly Yes |
| (Sidoroff et al., 2024) | Yes | Yes | Mostly Yes | Yes | Mostly Yes | Yes | Mostly Yes | Yes | Mostly Yes |
| (Silveri et al., 2024) | Mostly Yes | Mostly Yes | Yes | Mostly Yes | Mostly Yes | Yes | Yes | Yes | Yes |
| (Siquier & Andrés, 2022) | Mostly Yes | Yes | Yes | Yes | No | Yes | Yes | Yes | Yes |
| (Slomp et al., 2024) | Yes | Yes | Yes | Yes | Mostly Yes | Mostly Yes | Mostly Yes | Yes | Yes |
| (Smith et al., 2010) | Yes | Yes | Yes | Yes | Mostly Yes | Yes | Yes | Mostly Yes | Yes |
| (Sprengelmeyer et al., 2003) | Mostly Yes | Yes | Mostly Yes | Yes | Mostly Yes | Yes | Mostly Yes | Yes | Yes |
| (St. Clair et al., 1998) | Yes | Mostly Yes | Yes | Yes | Mostly Yes | Mostly Yes | Mostly Yes | Mostly Yes | Mostly Yes |
| (Stirnimann et al., 2018) | Yes | Yes | Yes | Mostly Yes | Mostly Yes | Yes | Yes | Yes | Yes |
| (Suzuki et al., 2006) | Mostly Yes | Mostly Yes | Mostly Yes | Yes | Yes | Mostly Yes | Yes | Yes | Yes |
| (Tessitore et al., 2002) | Mostly Yes | Mostly Yes | Mostly Yes | Yes | Mostly Yes | Mostly Yes | Yes | Mostly Yes | Yes |
| (Thomasson et al., 2022) | Yes | Yes | Mostly Yes | Yes | Mostly Yes | Yes | Yes | Yes | Yes |
| (Trompeta et al., 2023) | Yes | Yes | Yes | Yes | Yes | Yes | Yes | Yes | Mostly Yes |
| (Tsuruya et al., 2011) | Yes | Mostly Yes | Mostly Yes | Yes | Yes | Mostly Yes | Yes | Yes | Mostly Yes |
| (Ulusoy et al., 2015) | Yes | Yes | Mostly Yes | Yes | Unclear | Mostly Yes | Yes | Yes | Mostly Yes |
| (Usnich et al., 2023) | Mostly Yes | Yes | Yes | Yes | Yes | Yes | Yes | Yes | Mostly Yes |
| (Vélez Feijó et al., 2008) | Yes | Mostly Yes | Yes | Yes | Mostly Yes | Yes | Yes | Mostly Yes | Mostly Yes |
| (Ventura et al., 2012) | Yes | Yes | Mostly Yes | Mostly Yes | Yes | Yes | Yes | Yes | Mostly Yes |
| (Voruz et al., 2024) | Yes | Yes | Yes | Yes | Yes | Yes | Yes | Yes | Yes |
| (Voruz et al., 2020) | Yes | Yes | Yes | Yes | Yes | Yes | Yes | Yes | Yes |
| (Wabnegger et al., 2015) | Mostly Yes | Yes | Mostly Yes | Yes | Yes | Mostly Yes | Yes | Yes | Mostly Yes |
| (Waldthaler et al., 2019) | Yes | Yes | Yes | Yes | Yes | Yes | Yes | Yes | Yes |
| (Wasser et al., 2018) | Mostly Yes | Mostly Yes | Mostly Yes | Mostly Yes | Mostly Yes | Mostly Yes | Mostly Yes | Yes | Mostly Yes |
| (Wieser et al., 2012) | Yes | Yes | Mostly Yes | Yes | Mostly Yes | Yes | Mostly Yes | Yes | Mostly Yes |
| (Xi et al., 2015) | Mostly Yes | Yes | Yes | Yes | No | Yes | Mostly Yes | Yes | Mostly Yes |
| (Yip et al., 2003) | Mostly Yes | Mostly Yes | Mostly Yes | Yes | Mostly Yes | Yes | Yes | Yes | Mostly Yes |
| (Yu et al., 2018) | Yes | Yes | Yes | Mostly Yes | Yes | Yes | Mostly Yes | Yes | Mostly Yes |
| (Yu et al., 2012) | Yes | Yes | Yes | Yes | Yes | Yes | Yes | Yes | Mostly Yes |
| *Note.* Assessment was based on guidelines stated in Barker et al. (2023):  **Q1. Were the inclusion and exclusion criteria for the PD and control groups clearly defined, including the PD population/subgroup included?**  This item assesses whether the study clearly specified who was eligible for inclusion and exclusion in both the PD and control groups. Particular attention should be given to whether the study explicitly defined the type of PD included, such as idiopathic PD, early-stage PD, de novo PD, medicated PD, PD without dementia, PD-MCI, or advanced PD, rather than referring only generally to patients with PD.”   - Yes: The study clearly defined inclusion and exclusion criteria for both groups, including a sufficiently explicit description of the PD population included. Relevant exclusions were also stated, such as dementia, major neurological or psychiatric disorders, severe depression, psychosis, visual or auditory impairment, DBS, or medications likely to affect cognition or task performance. - Mostly Yes: The study provided generally clear inclusion and exclusion criteria and gave some meaningful information about the PD population, but one or more non-critical details were missing. For example, the study specified idiopathic PD and major exclusions, but did not fully describe medication-related exclusions or sensory screening. - Unclear: The study provided limited information about eligibility criteria. For example, it stated that patients with PD and controls were recruited, but did not clearly define the type of PD, the diagnostic basis, or key exclusion criteria. - No: The study did not provide meaningful inclusion or exclusion criteria, or the definition of the PD and/or control groups was too vague to determine who was eligible for participation.   **Q2. Were the study subjects and setting described in sufficient detail, including key demographic variables and PD clinical characteristics such as H&Y and UPDRS-III?**  This item assesses whether the sample and study context were described in enough detail to understand the characteristics of the participants and the setting in which the study was conducted. This includes both the PD and control groups. For the PD group, particular attention should be given to clinical disease characteristics, including Hoehn & Yahr stage and UPDRS-III / MDS-UPDRS Part III, when available. Relevant information includes sample size, age, sex, education, recruitment source, setting, global cognition, mood symptoms, and sensory or language-related characteristics. For the PD group, relevant variables also include disease duration, disease severity, motor severity, medication status, ON/OFF state, LEDD, H&Y, and UPDRS-III.   - Yes: The study described both the PD and control groups in adequate demographic and clinical detail. For the PD group, key disease characteristics were reported, including H&Y and UPDRS-III or equivalent motor severity information. The recruitment setting or source was also described. - Mostly Yes: The study provided a generally adequate description of the sample and setting, but some details were missing. For example, the study reported age, sex, education, disease duration, and either H&Y or UPDRS-III, but not both. Alternatively, the study described the PD group well but provided somewhat limited information about the control group. - Unclear: The study provided only partial descriptive information, such as age and sex, but insufficient detail about disease characteristics, control group characteristics, or recruitment setting. - No: The study provided little or no meaningful description of the participants or setting, making it difficult to understand the sample or judge its relevance.   **Q3. Was the diagnosis of PD measured in a valid and reliable way?**  This item assesses whether PD diagnosis was established using a valid and reliable method. The focus is on whether participants were appropriately classified as having Parkinson's disease.   - Yes: PD diagnosis was based on recognized clinical diagnostic criteria, such as UK Brain Bank criteria, MDS criteria, or an equivalent standard, or was established by a neurologist or movement disorders specialist using a clearly described clinical diagnostic process. - Mostly Yes: The study indicated that participants had a clinical diagnosis of PD and were recruited from a relevant clinical source, such as a neurology or movement disorders clinic, but did not fully specify the diagnostic criteria used. - Unclear: The study stated that participants had PD but did not provide enough information about how the diagnosis was established, who made the diagnosis, or whether standard diagnostic criteria were used. - No: PD status was based only on self-report, an unclear source, or a non-standard method, with no evidence that diagnosis was clinically verified.   **Q4a. Were the groups comparable on key background/demographic variables?**  This asks whether the PD and comparison groups were comparable on non-clinical background characteristics that could influence social cognition task performance.  Core variables: age, sex/gender, education, language/native language (when relevant to verbal or prosody tasks), hearing/vision status (when relevant to auditory/visual emotion tasks).   - Yes: The groups were matched or shown to be comparable on key demographic. - Mostly Yes: The groups were comparable on most major variables, but there were minor differences or some missing comparability information. - No: The groups differed substantially on one or more key variables likely to affect social cognition, and these differences were not addressed. Alternatively, no meaningful attempt was made to match, compare, or describe group comparability. - Unclear: The study did not provide enough information to determine whether the PD and control groups were comparable.   **Q4b. Were the groups comparable on key clinical or disease-relevant variables other than PD status?**  This asks whether the PD and comparison groups were comparable on clinical variables that could confound social cognition performance, apart from the presence/absence of PD itself. Important: for healthy controls, many PD-specific variables are naturally “not applicable.” So, this question should focus on whether the groups differed on clinical factors that both groups could have, and whether the PD group had clinical features that might bias interpretation.  Relevant clinical/comorbidity variables: depression / mood symptoms, dementia or MCI status or general cognitive screening (for example MMSE/MoCA, when relevant)psychiatric illness, other neurological illness, major medical illness, medication that could affect cognition or emotion processing. For pathological control groups: disease severity, cognitive status, mood, and relevant clinical characteristics. PD-specific variables are not “comparability” variables when comparing PD to healthy controls, but they matter if comparing PD subgroups or PD vs another clinical group: disease duration, H&Y, UPDRS-III, LEDD / medication status, ON/OFF state, side of onset / motor phenotype   - Yes: The groups were matched or shown to be comparable on key clinical or disease-relevant variables. - Mostly Yes: The groups were comparable on most major variables, but there were minor differences or some missing comparability information. - No: The groups differed substantially on one or more key variables likely to affect social cognition, and these differences were not addressed. Alternatively, no meaningful attempt was made to match, compare, or describe group comparability. - Unclear: The study did not provide enough information to determine whether the PD and control groups were comparable.   **Q5. Were relevant confounding factors identified?**  This item assesses whether the study recognized and measured factors that could confound the relationship between PD and social cognition outcomes.  Potential confounders include age, sex, education, global cognition, executive functioning, depression, anxiety, apathy, disease duration, disease severity, medication status, LEDD, ON/OFF state, visual or auditory impairment, language ability, psychiatric comorbidity, and task-specific cognitive demands. In neuroimaging studies, relevant confounders may also include head motion, brain atrophy, scanner parameters, preprocessing choices, and correction for multiple comparisons.   - Yes: The study identified and measured the main confounders relevant to its design, sample, and outcome measures. - Mostly Yes: The study identified and measured several important confounders but omitted one or more potentially relevant factors. The omissions were not severe enough to undermine the overall interpretability of the study. - No: The study did not identify or measure relevant confounding factors, despite their likely importance for interpreting social cognition outcomes in PD. - Unclear: Some potentially relevant variables were reported, but it was not clear whether they were considered as confounders or whether the study adequately recognized their possible influence.   **Q6. Were appropriate strategies used to deal with confounding factors?**  This item assesses whether the study used design-based or statistical strategies to reduce the influence of confounding factors that were detected or considered.  Relevant strategies may include matching, restriction, exclusion criteria, stratification, covariate adjustment, ANCOVA, regression models, mixed-effects models, partial correlations, sensitivity analyses, or subgroup analyses. For example, a study may control for age, education, global cognition, depression, or disease severity when comparing PD and control groups.   - Yes: The study used appropriate strategies to address the main confounding factors relevant to the research question and study design. - Mostly Yes: The study addressed some important confounders but did not fully account for all relevant factors. For example, it controlled for age and education but not mood symptoms or global cognition, despite their potential relevance. - No: The study did not use meaningful strategies to address confounding, despite identifying or demonstrating relevant group differences. - Unclear: The study mentioned possible confounders or reported group differences, but it was not clear whether or how these were handled analytically.   **Q7. Were social cognition and/or neuroimaging outcomes measured in a valid and reliable way?**  This item assesses whether the study's outcome measures were appropriate, valid, and reliable. Outcomes include behavioral, self-report, and neuroimaging measures of social cognition. Relevant social cognition domains include emotion recognition, Theory of Mind, and social problem-solving. Measures may include facial or vocal emotion recognition tasks, Reading the Mind in the Eyes Test, Faux Pas Test, Strange Stories, Hinting Task, Movie for the Assessment of Social Cognition, validated social problem-solving tasks, or other clearly described and justified tools. Neuroimaging outcomes may include MRI, fMRI, or PET measures, if acquisition, preprocessing, analysis, and outcome definition were adequately described.   - Yes: The outcomes were measured using validated, standardized, or well-established tools, or the study provided a clear and convincing justification for the measures used. Scoring procedures, task characteristics, and outcome definitions were sufficiently described. For neuroimaging studies, acquisition and analysis methods were appropriate and clearly reported. - Mostly Yes: The outcome measures were generally appropriate and sufficiently described, but some information was missing. For example, the study used a recognized task but did not fully report psychometric properties, cultural adaptation, or scoring details. For neuroimaging studies, the main imaging protocol was adequate, but some preprocessing or quality-control details were limited. - No: The outcome was measured using an inadequately described, non-standard, or poorly justified tool. Scoring was unclear, validity was doubtful, or the outcome measure did not adequately capture emotion recognition, Theory of Mind, social problem-solving, or the relevant neuroimaging construct. - Unclear: The study provided insufficient information about the outcome measure, task, scoring method, validation, or imaging pipeline to determine whether the outcome was measured validly and reliably.   **Q8. Was appropriate statistical analysis used?**  This item assesses whether the statistical analyses were appropriate for the study design, data structure, outcome type, and research question.  Relevant considerations include the suitability of group comparison tests, handling of non-normal data, adjustment for covariates, correction for multiple comparisons, reporting of effect sizes and confidence intervals when possible, and appropriate interpretation of results. For neuroimaging studies, this also includes appropriate correction for multiple comparisons, ROI or whole-brain analysis justification, motion control, and transparent reporting of statistical thresholds.   - Yes: The statistical analyses were appropriate for the study design and outcomes. The study used suitable group comparisons or models, addressed relevant assumptions, controlled for key covariates when needed, and applied appropriate correction for multiple comparisons where relevant. - Mostly Yes: The statistical analyses were generally appropriate, but some details were incomplete or some limitations were present. For example, the study used suitable group comparisons but did not report effect sizes, or controlled for some but not all relevant covariates. - No: The statistical analyses were inappropriate for the design or data, failed to address major confounders or multiple comparisons, or supported conclusions that were not justified by the analyses. - Unclear: The statistical approach was insufficiently described, making it difficult to determine whether the analyses were appropriate. For example, the paper reported that statistical comparisons were conducted, without specifying the tests, covariates, or correction procedures. | | | | | | | | | |

| Study | Q1 | Q2 | Q3 | Q4 | Q5 | Q6 | Q7 | Q8 | Q9 | Q10 | Q11 |
| --- | --- | --- | --- | --- | --- | --- | --- | --- | --- | --- | --- |
| (Arioli et al., 2022) | Yes | Yes | Yes | No | No | No | Unclear | Yes | No | Yes | Yes |
| (Bodden, Dodel, et al., 2010) | Mostly Yes | Yes | Yes | Yes | No | No | Unclear | Yes | No | Yes | Yes |
| (Bora et al., 2015) | Yes | Yes | Yes | Yes | No | No | Unclear | Yes | Yes | Yes | Yes |
| (Coundouris et al., 2019) | Yes | Yes | Yes | Yes | No | No | Unclear | Yes | Yes | Yes | Yes |
| (Coundouris et al., 2020) | Yes | Yes | Yes | Yes | No | No | Yes | Yes | Yes | Yes | Yes |
| (Desmarais et al., 2018) | Mostly Yes | No | Yes | Yes | No | No | Unclear | Mostly Yes | No | Yes | Yes |
| (Doskas et al., 2024) | Mostly Yes | Mostly Yes | Mostly Yes | No | No | No | Unclear | Mostly Yes | No | Yes | Yes |
| (Gothwal et al., 2022) | Mostly Yes | Mostly Yes | Mostly Yes | No | No | No | Unclear | Mostly Yes | No | Yes | Yes |
| (Gray & Tickle-Degnen, 2010) | Yes | Yes | Yes | Mostly Yes | No | No | Yes | Yes | Yes | Yes | Yes |
| (Hazelton et al., 2025) | Yes | Yes | Yes | Mostly Yes | No | No | Yes | Yes | Yes | Yes | Yes |
| (Ibarretxe-Bilbao et al., 2011) | Mostly Yes | Yes | Mostly Yes | No | No | No | Unclear | Mostly Yes | No | Yes | Yes |
| (Moonen et al., 2017) | Yes | Yes | Yes | Mostly Yes | Yes | Yes | Yes | Yes | No | Yes | Yes |
| (Peron et al., 2012) | Mostly Yes | Mostly Yes | Mostly Yes | Mostly Yes | No | No | Unclear | Mostly Yes | No | Yes | Yes |
| (Stafford et al., 2023) | Yes | Yes | Yes | Yes | Yes | Yes | Yes | Yes | Yes | Yes | Yes |
| (Voruz et al., 2025) | Yes | Yes | Mostly Yes | No | No | No | Unclear | Yes | No | Yes | Yes |
| *Note.* Assessment was fully aligned with the guidelines stated in Aromataris et al. (2015). | | | | | | | | | | | |
