# Supplementary material for: Social cognition in Parkinson’s disease: a comprehensive systematic review and integrative conceptual framework
Source: Front Aging Neurosci. 2026 Jun 29;18:1863728. doi: 10.3389/fnagi.2026.1863728 (PMC13357429; doi:10.3389/fnagi.2026.1863728)
Supplement: Supplementary file 3 [file Data_Sheet_3.docx]

**Summary of empirical findings – neural correlates of emotion recognition in patients with Parkinson’s Disease (PD)**

- The findings of a systematic review by **Ibarretxe-Bilbao et al. (2011)**, which included articles published in **MEDLINE** between **1998 and 2011**, disclosed that deficient emotion recognition in patients with PD were frequently associated with:
  Lower **gray matter volumes** in the **orbitofrontal cortex** and the **amygdala**, although not as a consistent finding.
- A systematic review by **Moonen et al. (2017)**, which addressed **emotional processing** in a broader sense (beyond emotion recognition alone), indicated that emotional processing relies on **two major neural pathways**, namely the ventral and dorsal systems. While the **ventral (lower) system is** responsible for **emotion perception** and the generation of **automatic emotional responses** (including the **amygdala**, **ventral striatum**, **ventral ACC**, **OFC**, **VLPFC**, **hypothalamus**, etc.), the **dorsal system** is responsible for **top-down emotional regulation** (i.e., **cognitive control over emotional states**, which are modulated by the **DLPFC**, **dorsal ACC**, and **hippocampus**).
  In PD, processing deficiencies are related to the dopamine-dependent **ventral system**, that are likely to yield **autonomic and perceptual deficits** in emotional responses, while the dorsal system may compensate for this impairment through **cognitive control**—that is, the brain’s attempt to “maintain” emotional processing at a normal level by recruiting cognitive control processes known to be modulated by **prefrontal regions**.
- A **meta-analysis by Hazelton et al. (2025)**, based on studies published up to **2023**, examined **neuroimaging studies of interoception, emotion, or social cognition in neurodegenerative diseases**, and included **10 studies**.
  It addressed emotion in a broader sense (**recognition of emotions in others or experience of one’s own emotions**) and found that neurofunctional **activations related to emotion processing in PD** involved the **frontal poles** and the **central opercular cortices bilaterally**, as well as the **left planum temporale** and the **postcentral gyrus**.
- **Trompeta et al. (2023)** found **no association** between **emotion recognition** and **metabolic activity** by employing resting-state PET.
- **Hazelton et al. (2023) conducted whole-brain VBM analysis and** found **no relationship** between **emotion recognition** and **gray matter volume**.
- **Robert et al. (2014)** studied patients with PD solely (without a healthy control or comparison group) using PET imaging and found that **higher overall emotion recognition accuracy** was associated with **higher metabolism** in the right precuneus and the left inferior occipital gyrus, and with **lower metabolism** in the posterior cingulate cortex and the superior frontal gyri bilaterally.
- **Funghi et al. (2025)** investigated patients with PD only (without a healthy control group). Their findings revealed that compared to patients without intact emotion recognition those **with emotion recognition impairment** (based on **Italian normative data**) showed **lower functional connectivity** between the **right and the left amygdala**, as well as between the **left amygdala and the left anterior temporal cortex**, and between the **right amygdala and the left anterior temporal cortex**. Across the whole patient sample, a positive correlation was found between **emotion recognition scores** and **connectivity** strengths between the **left and right amygdala** as well as between the **left amygdala** and a distributed fronto-temporo-parietal network including the:
  o Left Temporal Pole
  o Bilateral Inferior Temporal Gyrus, posterior division
  o Left Middle Temporal Gyrus, posterior division
  o Bilateral Temporal Pole
  o Left Supramarginal Gyrus, posterior division
  and between the **right amygdala** and:
  o Left/Right Precentral Gyrus
  o Left Precentral and Postcentral Gyrus
  o Right Postcentral Gyrus
  o Right Inferior Temporal Gyrus, posterior division
  Furthermore, in the total sample, **better emotion recognition** was associated with **higher gray matter volumes** in the right anterior temporal cortex as well as in bilateral structures of dorsomedial prefrontal cortex, primary and secondary somatosensory cortex and the amygdala.
- **Li et al. (2022)** found **no differences** in cortical thickness between **HC** and **PD**, but across groups, **longer reaction time medians** (across emotional conditions) were associated with **lower cortical thickness** in the left caudal middle frontal cortex, the left precentral cortex, the left superior frontal cortex, the right temporal pole and the right superior frontal cortex. Furthermore, **higher accuracy rates** for **happy faces specifically** were associated with **greater cortical thickness** in the **left superior temporal** region.
- **Tessitore et al. (2002) report that** compared to **HC**, PD showed **reduced neurofunctional (BOLD) activity** in a **face-matching task** with **negative affect (angry/fearful)** faces in
  o Right amygdala
  o Bilateral posterior fusiform gyrus
  In addition, PD patients showed **increased BOLD activity** in the emotional task (compared to the control task) in:
  o Bilateral amygdala
  o Bilateral ventral prefrontal cortex
  o Bilateral inferior frontal gyrus
  o Anterior cingulate cortex
  o Bilateral posterior fusiform gyrus
  o Bilateral inferior occipital gyrus
- **Fleury et al. (2014):** found **no difference** in BOLD activity between **PD** and **HC**. However, among PD patients, **greater BOLD activity** was observed in the **incongruent vs. congruent condition** (in a **Stroop-like face-word task**) for **fearful faces** in:
  o Right middle frontal cortex
  o Left middle and right inferior temporal cortex
- **Rodríguez-Antigüedad et al. (2024):** without a control or comparison group, found that **higher overall emotion recognition accuracy** was associated with **greater cortical thickness** in:
  o Left superior frontal gyrus
  When examining each emotion separately, **positive correlations** were found between **cortical thickness** and **accuracy in anger recognition** in:
  o Bilateral superior frontal gyrus
  o Left middle temporal gyrus
  o Right superior parietal cortex
  o Right precuneus
  and **positive correlations** between **cortical thickness** and **accuracy in disgust recognition** in:
  o Left superior frontal gyrus
  o Right fusiform gyri
  No significant correlations were found for the other emotions.
- **Burgio et al. (2024):** conducted an **ROI-only analysis**. In the **matching task**, they found **no association** between **VBM** and the **LISAS** score (a composite of reaction time and accuracy; higher scores indicate worse performance).
  In an **emotional memory task** (which included a learning phase of emotional faces followed by a recognition phase—choosing which of two faces had appeared in the learning phase), a relationship was found between **VBM** and **LISAS** for **fear** only (though insufficiently detailed).
  Better task performance was associated with **increased gray matter volume** in the:
  o Striatum
  o Right temporal regions
- **Wabnegger et al. (2015):** compared with controls, **PD** showed **decreased BOLD activation** for **sad vs. neutral faces** in:
  o Right putamen
  o Right inferior frontal gyrus
  In contrast, compared with controls, PD showed **stronger BOLD activation** in:
  o Right secondary somatosensory cortex for **angry vs. neutral** faces
  o Right inferior parietal lobule and left secondary somatosensory cortex for **disgusted vs. neutral** faces
  o Left inferior parietal lobule and right orbitofrontal cortex for **fearful vs. neutral** faces
  o Bilateral somatosensory cortex for **sad vs. neutral** faces
  Based on these results, they focused on the **inferior parietal** and **secondary somatosensory cortices**, finding that **stronger BOLD activation** in the **secondary somatosensory cortices** was associated with **greater accuracy and higher intensity ratings** for **fear and disgust**, whereas **stronger activation** in the **left inferior parietal** was associated with **higher intensity ratings** for **anger**.
- **Dan et al. (2019):** compared to HC, PD showed **stronger BOLD activation** when matching **negative faces** (fear and anger) in:
  o Bilateral calcarine sulci
  o Lingual gyri
- **Ibarretxe-Bilbao et al. (2009) found that** in patients with PD (compared to HC) overall emotion recognition scores were positively correlated to **gray matter volume in the bilateral orbitofrontal cortex**.
- **Baggio et al. (2012)** report that higher overall emotion recognition accuracy score was associated with **increased gray matter volumes** in the Dorsal ACC. A more fine-grained analyses of specific emotional expressions revealed that higher accuracy in recognizing **sad faces** was associated with **increased gray matter volumes** in:
  o Right lateral and medial orbitofrontal cortex
  o Right amygdala
  o Right postcentral gyrus,
  while higher accuracy in recognizing **angry faces** was associated with **increased gray matter volumes** in:
  o Bilateral ventral striatum (nuclei accumbens)
  o Right occipital fusiform gyrus
  o Subgenual cortex
  and higher accuracy in recognizing **disgusted faces** was associated with **increased gray matter volumes** in Dorsal ACC.
  Overall and compared to HC, patients with PD (as a group) exhibited deficient emotion recognition skills (indexed by lower accuracy rates) that were associated with **decreased gray matter volumes**.
  Regarding structural brain connectivity (as indexed by **Fractional Anisotropy/FA)**, better **sad face recognition** was related to **higher FA** in:
  o Bilateral inferior fronto-occipital fasciculus, including the right forceps minor
  o Corpus callosum to the left centrum semiovale
  o Left inferior longitudinal fasciculus
  Overall, patients with PD showed **lower FA** (reflecting poorer structural connectivity) than HC.
- **Di Tella et al. (2021) report a positive correlation between sad face recognition** and **gray matter volumes** in the **bilateral dorsal striatum (caudate and putamen)**.
- **Stirnimann et al. (2018)** examined the relationship between **PET metabolism** and **emotional prosody by** differentiating motor symptom side (**RPD/LPD**). While no significant associations were found for **RPD**, for **LPD**, **poorer discrimination scores** (smaller differences between correct emotion ratings and the other incorrect emotion ratings) were associated with **lower brain metabolism** for **happy prosody** in:
  o Right orbitofrontal cortex

**Converging evidence across studies:**
o **Bilateral superior frontal gyrus** (Li et al., 2022; Robert et al., 2014; Rodríguez-Antigüedad et al., 2024)
o **Bilateral amygdala** (Baggio et al., 2012; Funghi et al., 2025; Ibarretxe-Bilbao et al., 2011; Tessitore et al., 2002)
o The **temporal cortex** may be **emotion-specific** (Fleury et al., 2014; Li et al., 2022), and the **striatum** (including the **nuclei accumbens, caudate, and putamen**) may be involved particularly in **negative emotions** (fear, anger, sadness) (Baggio et al., 2012; Burgio et al., 2024; Di Tella et al., 2021).
o The **right precuneus** may be related to **emotion recognition** (Robert et al., 2014; Rodríguez-Antigüedad et al., 2024), as well as the **fusiform gyrus** (Baggio et al., 2012; Rodríguez-Antigüedad et al., 2024; Tessitore et al., 2002).
o **Bilateral orbitofrontal cortex** (consistent with the systematic review of Ibarretxe-Bilbao et al., 2011, and the findings by Baggio et al., 2012; Ibarretxe-Bilbao et al., 2009), though its **specific role remains unclear**, as the **right side** was linked to **happy prosody** (Stirnimann et al., 2018) but also to **fearful or sad face recognition** (Baggio et al., 2012; Wabnegger et al., 2015).
o The **postcentral gyrus** may also play a key role in emotion recognition, as **Baggio et al. (2012)** found that **lower gray matter volumes** there were linked to **poorer sad face recognition**, and **Funghi et al. (2025)** found that **lower connectivity** between the postcentral gyrus and the **amygdala** was associated with **poorer overall emotion recognition**.
